# Isothiourea-Catalyzed [2 + 2] Cycloaddition of C(1)-Ammonium Enolates and *N*-Alkyl Isatins

**DOI:** 10.1021/acs.orglett.2c02170

**Published:** 2022-07-18

**Authors:** Yusra Abdelhamid, Kevin Kasten, Joanne Dunne, Will C. Hartley, Claire M. Young, David B. Cordes, Alexandra M. Z. Slawin, Sean Ng, Andrew D. Smith

**Affiliations:** † EaStCHEM, School of Chemistry, University of St. Andrews, North Haugh, St. Andrews, U.K., KY16 9ST; ‡ Syngenta, Jealott’s Hill International Research Centre, Bracknell, Berkshire RG42 6EY, U.K.

## Abstract

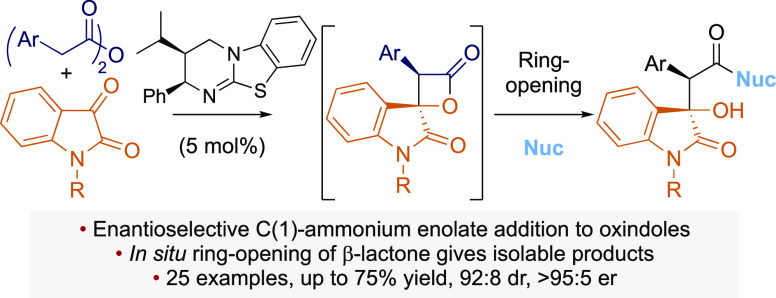

Enantioselective [2 + 2] cycloaddition of C(1)-ammonium enolates generated catalytically using the isothiourea HyperBTM with *N*-alkyl isatins gives spirocyclic β-lactones. *In situ* ring opening with an amine nucleophile generates isolable highly enantioenriched products in up to 92:8 dr and in >99:1 er.

β-Lactones are versatile synthetic building blocks and significant components of many bioactive natural products.^1,2^ As a consequence, a range of enantioselective synthetic methods for their preparation has been developed, with both Lewis acid and Lewis base catalyzed approaches common.^3^ In terms of Lewis base catalysis using tertiary amines, the use of cinchona alkaloids and chiral DMAP derivatives has been extensively used to promote β-lactone formation through the generation of an intermediate C(1)-ammonium enolate.^4^ Although versatile, these methods typically rely on the generation of reactive monosubstituted ketenes (formed *in situ* from acyl chlorides) or isolable but sensitive disubstituted ketenes as starting materials.^5^ In an alternative approach, Romo introduced the NCAL (nucleophile-catalyzed aldol-lactonization) process to prepare β-lactones from keto-acids (Scheme 1a).^6^ Key to this protocol was the development of carboxylic acids as the C(1)-ammonium enolate precursor, with a modified Mukaiyama reagent used for *in situ* generation of a reactive ester. Addition of either a cinchona alkaloid or isothiourea catalyst was used to generate the desired C(1)-ammonium enolate, with subsequent *intramolecular* formal [2 + 2]-cycloaddition onto the pendant carbonyl giving highly enantioenriched β-lactones. Building on this work, we previously demonstrated the use of symmetric arylacetic anhydrides as alternative C(1)-ammonium enolate precursors.^7^ These anhydrides are generally readily prepared from the parent carboxylic acid, are easy to handle, and can be used in conjunction with isothiourea catalysts without requiring the excess base that is a recognized limitation of alternative protocols using carboxylic acids as starting materials. This approach was applied to the HyperBTM-catalyzed enantioselective *intermolecular* formation of β-lactones with perfluoroalkyl ketones and arylacetic anhydrides (Scheme 1b). Mechanistic studies using natural abundance ^13^C kinetic isotope effect experiments, together with computational analyses, indicated the operation of a concerted asynchronous [2 + 2] cycloaddition.^8^ To date, the isothiourea-catalyzed intermolecular [2 + 2] cycloaddition approach has not been demonstrated using cyclic ketones as substrates; in this manuscript the application of *N*-protected isatins as electrophiles to initially generate spirooxindole β-lactones is investigated.^9^ Attempted isolation of the β-lactones led to spontaneous decarboxylation, but postcatalysis addition of an amine nucleophile led to β-lactone ring opening to give isolable highly enantioenriched products (Scheme 1c). Mechanistic studies are consistent with *in situ* epimerization of the initially formed β-lactone, leading to a mixture of β-hydroxy amide diastereoisomers in highly enantioenriched form (up to 92:8 dr, >99:1 er for both diastereoisomers).

**Scheme 1:**
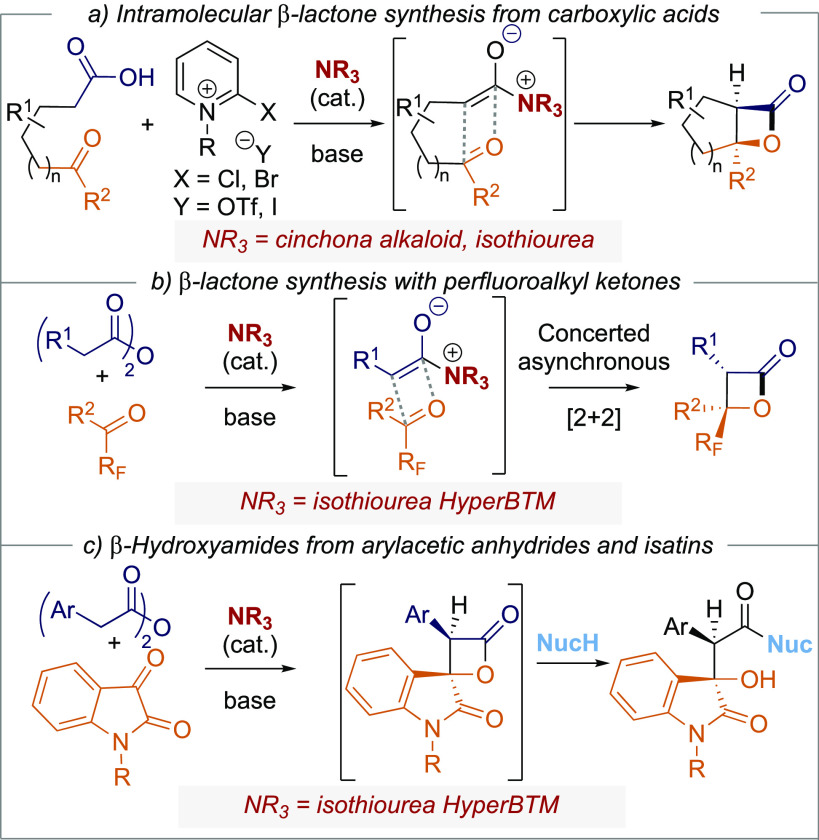
Tertiary Amine-Catalyzed β-Lactone Syntheses: (a) Romo’s NCAL Intramolecular β-Lactone Synthesis; (b) Previous Work: β-Lactone Synthesis with Perfluoroalkyl Ketones; (c) This Work: β-Lactone Synthesis with Isatins Followed by Ring Opening

Initial studies began with the reaction of (2*R*,3*S*)-HyperBTM **1** (5 mol %) with phenylacetic anhydride **2** and *N*-benzyl isatin **3**, in CH_2_Cl_2_. Although β-lactone **5** could be observed by ^1^H NMR spectroscopic analysis of the crude reaction product, attempted chromatographic purification resulted in the isolation of alkene **4** in 58% yield [66:34 (*E*)/(*Z*)], consistent with decarboxylation of β-lactone **5**.^10^ As an alternative, the crude reaction mixture was treated *in situ* with excess benzylamine (3.0 equiv) to give the isolable β-hydroxyamide derivative **6** (76:24 dr).^11^ Chromatographic purification gave the separable diastereoisomers in 81% combined yield and >99:1 er for each diastereomer (Scheme 2a). *In situ* formation of a mixed anhydride, using phenylacetic acid and pivaloyl chloride, and subsequent HyperBTM-catalyzed cycloaddition followed by ring opening led to slightly decreased levels of diastereoselectivity (69:31 dr, >99:1 er) and product yield (57%). Further attempted optimization through variation in catalyst, solvent, and reaction temperature gave no significant improvement in either product dr or yield (see Supporting Information (SI) for full information) with consistent high enantioselectivity observed. Intrigued by the observation that both product diastereoisomers were highly enantioenriched (>99:1 er), further investigations probed if the stereochemical outcome (76:24 dr, >99:1 er) was intrinsic to the catalyzed process, or alternatively a consequence of *in situ* epimerization of the β-lactone **5** or β-hydroxyamide product **6**. Control experiments showed that retreatment of a single diastereoisomer of β-hydroxyamide **6** (>95:5 dr, 99:1 er) to the reaction conditions, or with excess ^*i*^Pr_2_NEt, led to no change in dr or er. *In situ* reaction monitoring at room temperature using ^1^H NMR spectroscopy allowed the concentration and dr of β-lactone **5** to be quantified over the reaction course (Scheme 2b). After 10 min, a single diastereoisomer of β-lactone **5** (60% conversion of isatin **3**) was observed, with the dr gradually reducing with time to 85:15 dr after 2 h (∼80% conversion). Extending the reaction time to 16 h gave the β-lactone **5** in 70:30 dr and reduced yield (70%) due to the observation of (*E*)/(*Z*) alkenes **4** from decarboxylation in the reaction mixture. These results are consistent with an initial highly stereoselective catalytic process giving β-lactone **5** in high diastereo- and enantioselectivity, with *in situ* epimerization giving a mixture of diastereoisomers.^12^ To further probe this process, variation of the reaction time before addition of benzylamine, plus the use of alternative amine nucleophiles for derivatization, was investigated (Scheme 2c). At 0 °C, addition of benzylamine after a 30-min reaction time gave product **6** in an improved 95:5 dr and >99:1 er but with reduced yield (36%) compared to the standard 3 h reaction time (81%, 76:24 dr, >99:1 er). The use of morpholine and pyrrolidine gave the corresponding products **7** and **8** respectively in uniformly excellent enantioselectivity (>99:1 er), but varying diastereoselectivity (85:15 and 68:32 dr respectively). The variation in dr presumably reflects competition between the basicity (promoting epimerization alongside ^*i*^Pr_2_NEt) and nucleophilicity (promoting ring opening) of these amines and their relative reaction rates. Consistent with these observations, X-ray crystal structure analysis allowed the relative and absolute configuration of the product minor diastereoisomer **9** to be unambiguously determined (Scheme 2d).^13^ The observed (*S*)-configuration at C(2) is opposite to that expected based upon the established selectivity of (2*R*,3*S*)-HyperBTM in C(1)-ammonium enolate reactions,^14^ consistent with epimerization of β-lactone **5**.

**Scheme 2:**
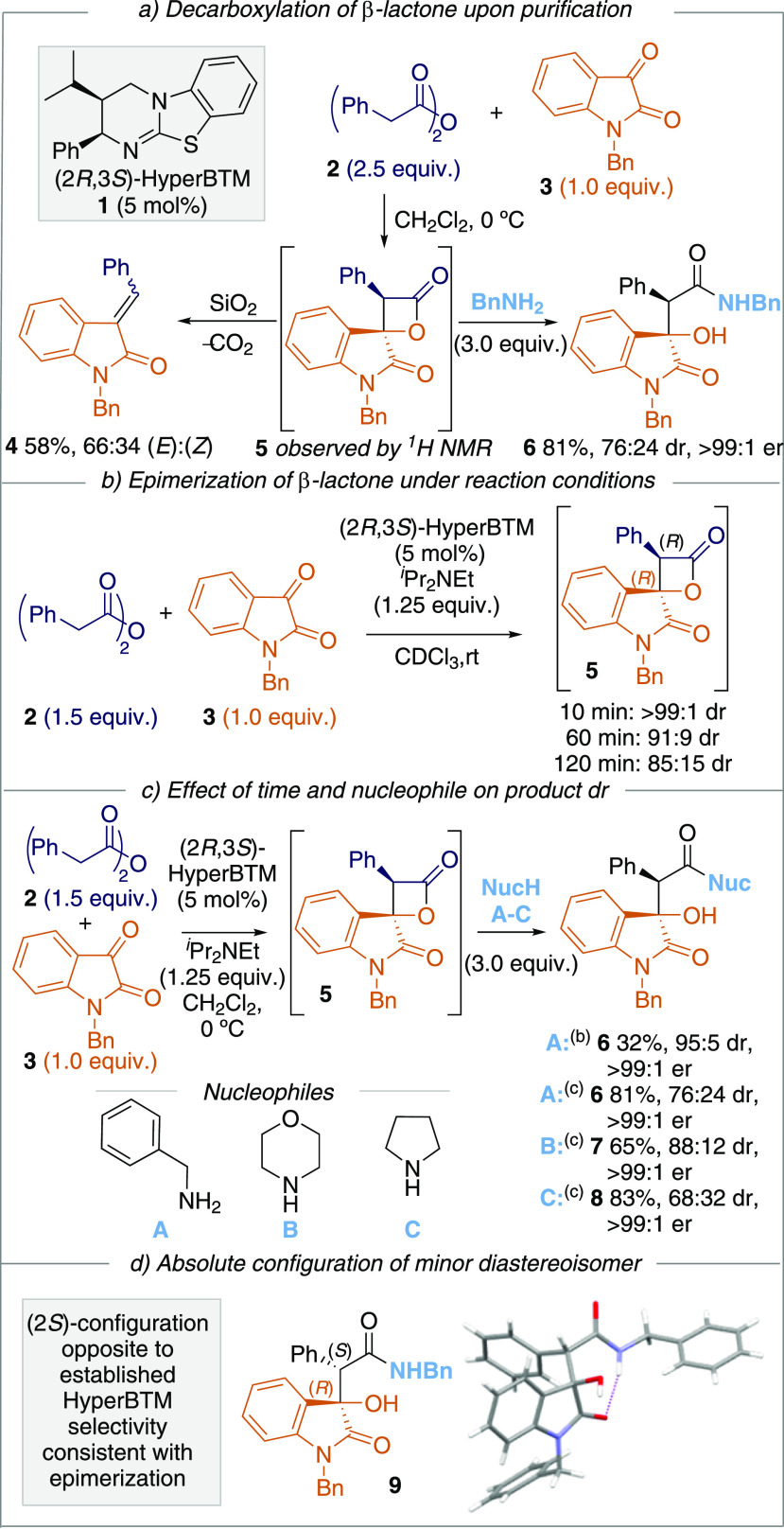
Optimization and β-Lactone Epimerization Yield of isolated products. Reported er of major diastereoisomer (er always >99:1 for minor diastereoisomer). ^1^H NMR of the crude reaction product was used to determine dr. (b) 30 min reaction time before addition of amine; (c) 3 h reaction time before addition of amine.

Following these observations, the scope and limitations of this process were examined through variation of the isatin, anhydride, and ring-opening nucleophile reaction components (Figure 1). Alternative *N*-substituents within the isatin were tolerated, with *N*-methyl, *N*-allyl protected isatins giving the corresponding products **10** and **11** in good yields and consistently high enantioselectivity (>99:1 er). Interestingly, using *N*-Boc protected isatin led to product **12** in >99:1 er as a result of ring opening of the β-lactone and the oxindole presumably facilitated by the *N*-Boc substituent.^15^

**Figure 1:**
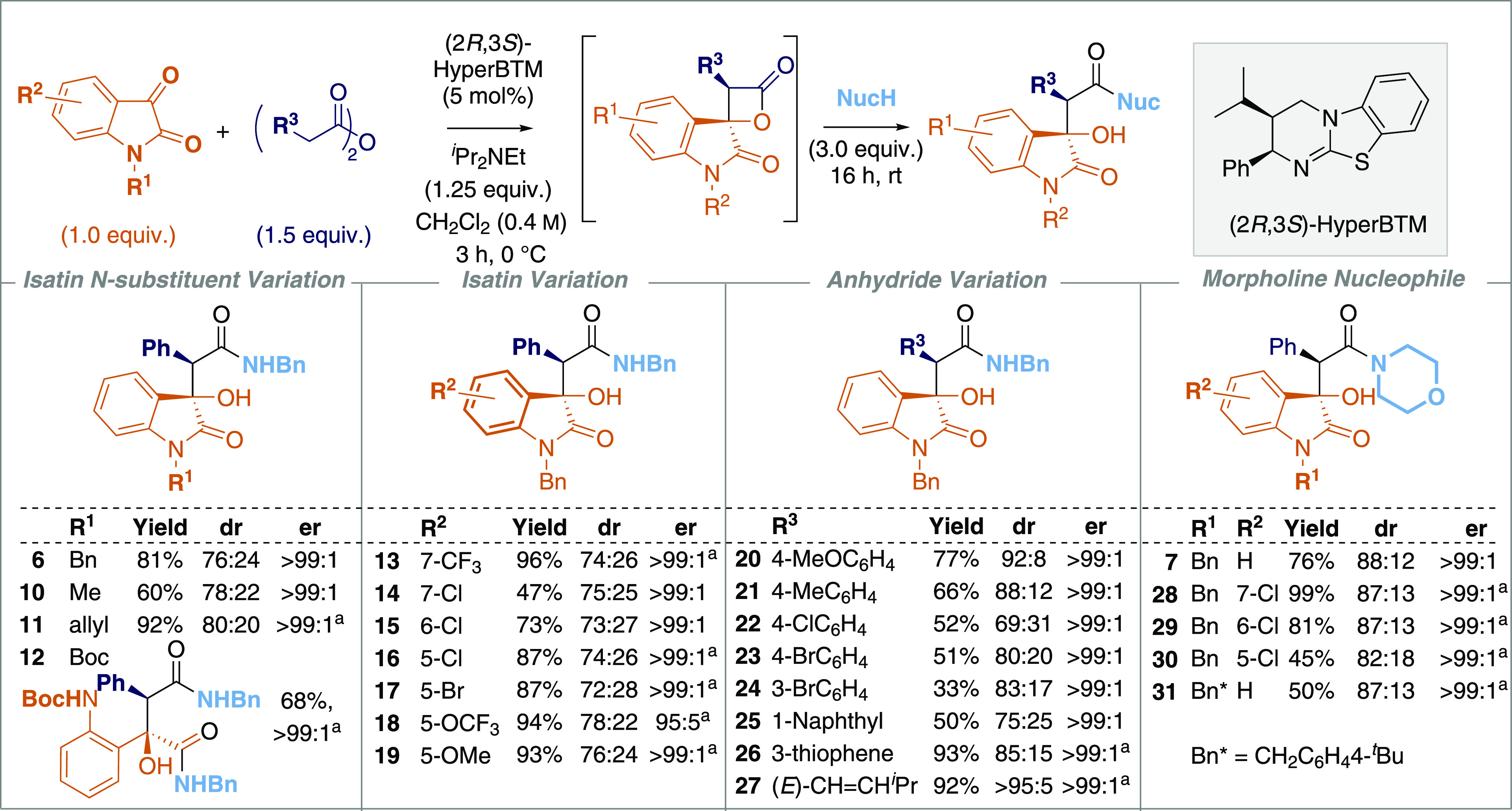
Scope of the reaction. Combined yield of isolated diastereoisomers. Reported er of major diastereoisomer. Reaction performed on 0.40 mmol scale under air atmosphere. ^1^H NMR of the crude reaction product used to determine dr. ^*a*^(2*S*,3*R*)-HyperBTM used, product has opposite absolute configuration to that shown.

Variation of the isatin component was expanded to incorporate 5-, 6-, and 7-substituted isatins, as well as variation of the *N*-substituent. Substitution at the 5-, 6-, and 7-position was consistently tolerated, with good product yields observed for both electron-withdrawing and electron-donating substituents, giving products **13**–**19** with excellent enantioselectivity (>99:1 er) and consistent diastereoselectivity (from 72:28 to 78:22 dr). Unfortunately, 4-substitution of the isatin was not tolerated, with 4-Cl isatin giving <10% conversion to products. The poor conversion in this case is ascribed to the spatial proximity to the reaction center, disfavoring nucleophilic addition.

A selection of anhydrides was next synthesized from the parent carboxylic acids and tested in this cycloaddition/ring-opening process. Notably, incorporating the 4-MeOC_6_H_4_-substituent within the anhydride gave product **20** in 77% yield with 92:8 dr and >99:1 er. The higher dr (compared to **6**) presumably reflects the electron-donating nature of the aryl substituent that reduces the acidity of the C(3)–H within the β-lactone intermediate. This effect was also observed for the 4-MeC_6_H_4_-substrate **21** (88:12 dr, >99:1 er). Halogen-containing phenylacetic anhydrides were tolerated, giving the corresponding products **22**–**24** in variable diastereoselectivity (from 69:31 to 83:17 dr) but with excellent enantiocontrol. A 1-naphthyl-derivative **25** was prepared in 50% yield and >99:1 er, while extension to a 3-thiophene derivative **26** was also tolerated (85:15 dr, >99:1 er). While the use of simple alkyl anhydride derivatives did not generate any product (see SI for further information), the use of an (*E*)-alkenyl substituent was tolerated, giving **27** in excellent yield, dr, and er (91%, >95:5 dr, >99:1 er) that was amenable to scale-up to 10 mmol scale, giving >4.1 g of product. Finally, five examples using morpholine as the derivatizing agent were demonstrated (**7**, **28**–**31**). Generally, good yields and higher diastereocontrol were observed than in the corresponding benzylamides, with excellent enantiocontrol maintained (>99:1 er). Reduced yield was observed for the 5-chloro **30** and *N*-(*para*-*tert*-butylbenzyl) **31** derivatives, with the low solubility of **30** complicating the purification process.

Based upon these observations, alongside previous work in this area, a catalytic cycle is proposed (Figure 2). Initial addition of (2*R*,3*S*)-HyperBTM **1** to the phenylacetic anhydride **2** results in the formation of acyl ammonium ion pair **32**. Deprotonation at C(2)- gives the corresponding (*Z*)-ammonium enolate **33**,^16^ with a stabilizing 1,5-O···S chalcogen bonding interaction (n_O_ to σ*_S–C_)^17−19^ providing a conformational bias and ensuring coplanarity between the 1,5-O- and S-atoms. The observed product configuration is consistent with that observed in the related [2 + 2]-cycloaddition of C(1)-ammonium enolates and trifluoromethylketones,^8^ so by analogy a similar concerted asynchronous [2 + 2]-cycloaddition pathway via transition state assembly **34** to give **35** is proposed. Subsequent catalyst release generates the β-lactone **5** in high diastereo- and enantioselectivity. *In situ* epimerization of the lactone at C(3)- leads to a mixture of β-lactone diastereoisomers, with the subsequent addition of an amine nucleophile promoting ring opening to give the isolable β-hydroxyamide products.

**Figure 2:**
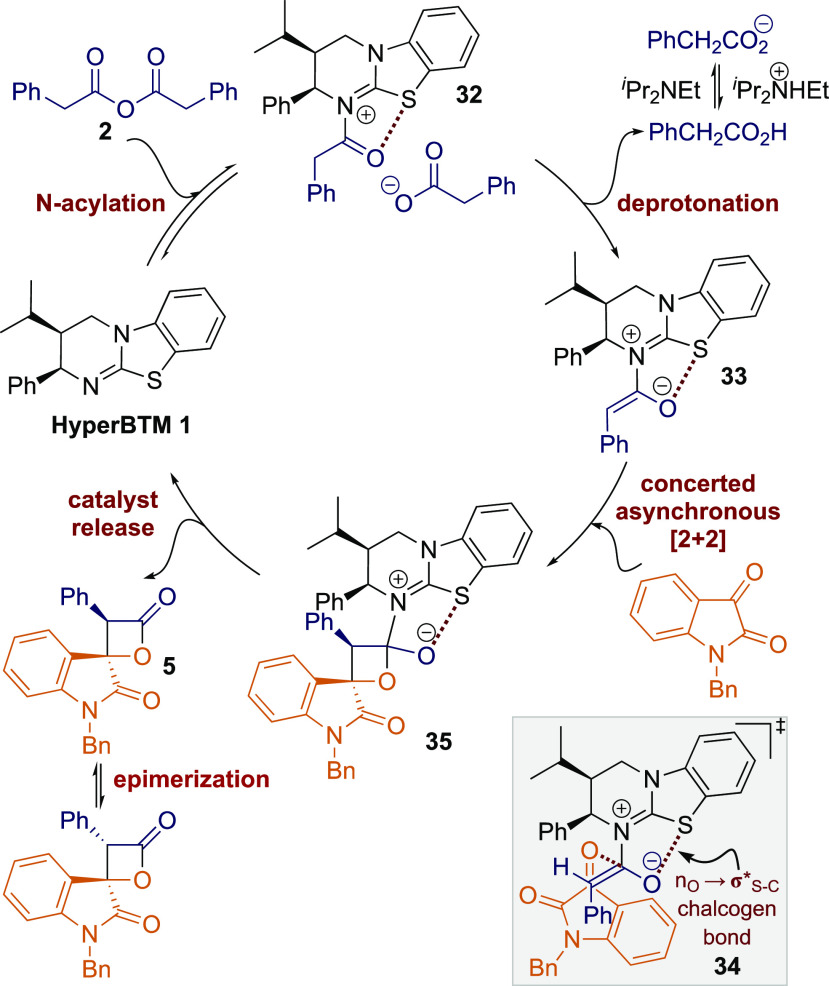
Proposed catalytic cycle for the intermolecular [2 + 2] cycloaddition to form β-lactones.

In summary, a procedure for the generation of highly enantioenriched β-hydroxyamides (>99:1 er) has been developed. This protocol involves the *in situ* preparation of β-lactones from isatins and 2-arylacetic anhydrides using the isothiourea HyperBTM **1** to promote a concerted asynchronous [2 + 2]-cycloaddition, followed by *in situ* ring opening with a nucleophile. Mechanistic studies suggest a base-promoted epimerization leads to a reduction in the diastereoselectivity of the initially formed β-lactone product, giving rise to a diastereoisomeric mixture of isolable products each in high er (>99:1 er). Further work from this laboratory is focused upon alternative application of isothioureas and other Lewis bases in enantioselective catalysis.

## Supporting Information Available

Full experimental procedures, characterization data, NMR spectra and HPLC chromatograms for all new compounds, as well as crystallographic data for **9** (CCDC 2153991) (PDF)

## Accession Codes

CCDC 2153991 contains the supplementary crystallographic data for this paper. These data can be obtained free of charge via www.ccdc.cam.ac.uk/data_request/cif, or by emailing data_request@ccdc.cam.ac.uk, or by contacting The Cambridge Crystallographic Data Centre, 12 Union Road, Cambridge CB2 1EZ, UK; fax: +44 1223 336033.

## References

[ref1] aMukherjee S.; Biju A. Recent advances in the organocatalytic enantioselective synthesis of functionalized β-lactones. Chem.—Asian J. 2018, 13, 2333 10.1002/asia.201800902. 30033573

[ref2] aPommier A.; Pons J.-M. The synthesis of natural 2-oxetanones. Synthesis 1995, 1995, 729–744. 10.1055/s-1995-4011.

[ref3] aSchneider C. Catalytic, enantioselective syntheses of β-lactones-versatile synthetic building blocks in organic chemistry. Angew. Chem., Int. Ed. 2002, 41, 744–746. 10.1002/1521-3773(20020301)41:5<744::AID-ANIE744>3.0.CO;2-V. 12491322

[ref4] aWynberg H.; Staring E. G. J. Asymmetric synthesis of (*S*)- and (*R*)-malic acid from ketene and chloral. J. Am. Chem. Soc. 1982, 104, 166–168. 10.1021/ja00365a030.

[ref5] aWilson J. E.; Fu G. C. Asymmetric synthesis of highly substituted β-lactones by nucleophile-catalyzed [2 + 2] cycloadditions of disubstituted ketenes with aldehydes. Angew. Chem., Int. Ed. 2004, 43, 6358–6360. 10.1002/anie.200460698. 15558690

[ref6] aCortez G. S.; Tennyson R. L.; Romo D. Intramolecular, nucleophile-catalyzed aldol-lactonization (NCAL) reactions: catalytic, asymmetric synthesis of bicyclic β-lactones. J. Am. Chem. Soc. 2001, 123, 7945–7946. 10.1021/ja016134+. 11493084

[ref7] Morrill L. C.; Ledingham L. A.; Couturier J.-P.; Bickel J.; Harper A. D.; Fallan C.; Smith A. D. 2-Arylacetic anhydrides as ammonium enolate precursors. Org. Biomol. Chem. 2014, 12, 624–636. 10.1039/C3OB41869C. 24292454

[ref8] aBarrios Antunez D.; Greenhalgh M. D.; Brueckner A. C.; Walden D. M.; Elías-Rodríguez P.; Roberts P.; Young B.; West T. W.; Slawin A. M. Z.; Cheong P. H-Y.; Smith A. D. Catalytic enantioselective synthesis of perfluoroalkyl-substituted β-lactones *via* a concerted asynchronous [2 + 2] cycloaddition: a synthetic and computational study. Chem. Sci. 2019, 10, 6162–6173. 10.1039/C9SC00390H. 31360423 PMC6585878

[ref9] For a related [2 + 2]-cycloaddition to generate spirocyclic β-lactams using isothioureas, see: Jin J.-H.; Zhao J.; Yang W.-L.; Deng W.-P. Asymmetric Synthesis of Spirooxindole β-lactams via Isothiourea-catalyzed Mannich/lactamization Reaction of Aryl Acetic Acids with Isatin-derived Ketimines. Adv. Synth. Catal. 2019, 361, 1592–1596. 10.1002/adsc.201801621.

[ref10] aNoyce D. S.; Banitt E. H. The Stereochemistry of the Decarboxylation of β-Lactones to Form Olefins. J. Org. Chem. 1966, 31, 4043–4047. 10.1021/jo01350a037.

[ref11] Attempted ring opening of the β-lactone **5** with MeOH led to a complex product distribution so this was not followed further.

[ref12] bJi D.-S.; Liang H.; Yang K.-X.; Feng Z.-T.; Luo Y.-C.; Xu G.-Q.; Gu Y.; Xu P.-F. Solvent directed chemically divergent synthesis of β-lactams and α-amino acid derivatives with chiral isothiourea. Chem. Sci. 2022, 13, 1801–1807. 10.1039/D1SC06127E. 35282623 PMC8826511

[ref13] CCDC 2153991 contains crystallographic data for the minor (2*S*,3*R*)-diastereoisomer **9**.

[ref14] aMorrill L. C.; Lebl T.; Slawin A. M. Z.; Smith A. D. Catalytic asymmetric α-amination of carboxylic acids using isothioureas. Chem. Sci. 2012, 3, 2088–2093. 10.1039/c2sc20171b.

[ref15] aFranke A. Synthesis and reactions of (o-acylamino)phenylglyoxylic amides. Liebigs. Annalen der Chemie 1982, 1982, 794–804. 10.1002/jlac.198219820420.

[ref16] Wang C.; Li S.-J.; Zhang M.; Wei D.; Ding L. Origin of stereoselectivity in an isothiourea catalyzed Michael addition reaction of aryl ester with vinyl sulfone. New. J. Chem. 2020, 44, 17906–17911. 10.1039/D0NJ03540H.

[ref17] aBirman V. B.; Li X.; Han Z. Nonaromatic Amidine Derivatives as Acylation Catalysts. Org. Lett. 2007, 9, 37–40. 10.1021/ol0623419. 17192079

[ref18] aMukherjee A. J.; Zade S. S.; Singh H. B.; Sunoj R. B. Organoselenium Chemistry: Role of Intramolecular Interactions. Chem. Rev. 2010, 110, 4357–4416. 10.1021/cr900352j. 20384363

[ref19] aBleiholder C.; Gleiter R.; Werz D. B.; Köppel H. Theoretical Investigations on Heteronuclear Chalcogen–Chalcogen Interactions: On the Nature of Weak Bonds between Chalcogen Centers. Inorg. Chem. 2007, 46, 2249–2260. 10.1021/ic062110y. 17311376

