# Supplementary material for: Isothiourea-Catalyzed [2 + 2] Cycloaddition of C(1)-Ammonium Enolates and N-Alkyl Isatins
Source: Org Lett. 2022 Jul 18;24(29):5444–9. doi: 10.1021/acs.orglett.2c02170 (PMC9490795; doi:10.1021/acs.orglett.2c02170)
Supplement: Supplementary file 1 — ol2c02170_si_001.pdf [file ol2c02170_si_001.pdf]

Supporting information

Isothiourea-Catalyzed [2+2] Cycloaddition of C(1)-Ammonium Enolates and *N*-Alkyl Isatins

Yusra Abdelhamid,[a] Kevin Kasten,[a] Joanne Dunne,[a] Will. C. Hartley,[a] Claire M. Young,[a] David. B. Cordes,[a] Alexandra M. Z. Slawin,[a] Sean Ng[b] and Andrew D. Smith*

a. EaStCHEM, School of Chemistry, University of St Andrews, North Haugh, St Andrews, UK, KY16 9ST.

b. Syngenta, Jealott's Hill International Research Centre, Bracknell, Berkshire, RG42 6EY, UK

**Corresponding Author:* ads10@st-andrews.ac.uk

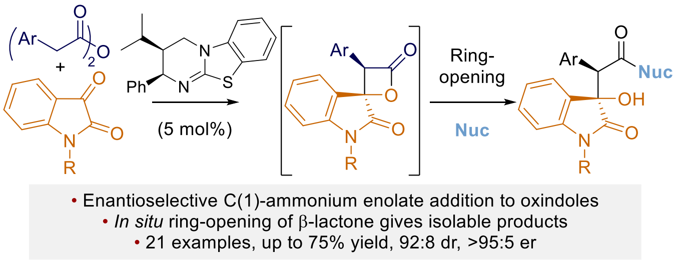

ABSTRACT: Enantioselective [2+2] cycloaddition of C(1)-ammonium enolates generated catalytically using the isothiourea HyperBTM with *N*-alkyl isatins gives spirocyclic β-lactones. *In situ* ring-opening with an amine nucleophile generates isolable highly enantioenriched products in up to 92:8 dr and in >99:1 er.

Table of Contents

I. General Procedures 4

II. Optimisation of Reaction Conditions 6

a) **Scheme 2a** 7

b) **Scheme 2b** 10

III. Syntheses of 3-hydroxyoxindoles 12

a) ***N*-benzyl-2-(1-benzyl-3-hydroxy-2-oxoindolin-3-yl)-2-phenylacetamide (6 and 9)** 12

b) ***N*-benzyl-2-(1-benzyl-7-chloro-3-hydroxy-2-oxoindolin-3-yl)-2-phenylacetamide (7)** 27

c) **1-Benzyl-4-chloro-3-hydroxy-3-(2-pyrrolidino-2-oxo-1-phenylethyl)indolin-2-one (8)** 33

d) ***N'*-Benzyl,*N*-methyl 2-(3-hydroxy-2-oxoindolin-3-yl)-2-phenylacetamide (10)** 39

e) ***N*-benzyl-2-(1-allyl-3-hydroxy-2-oxoindolin-3-yl)-2-phenylacetamide (11)** 51

f) ***N,N'*-dibenzyl 2-(*N*-(*tert*-butyloxycarbonyl)anilin-2-yl)-2-hydroxy-3-phenylsuccinamide (12)** 63

g) ***N,N'*-dibenzyl-2-(3-hydroxy-2-oxo-7-trifluoromethylindolin-3-yl)-2-phenylacetamide (13)** 75

h) ***N*-benzyl-2-(1-benzyl-7-chloro-3-hydroxy-2-oxoindolin-3-yl)-2-phenylacetamide (14)** 88

i) ***N,N'*-Dibenzyl-2-(6-chloro-3-hydroxy-2-oxoindolin-3-yl)-2-phenylacetamide (15)** 99

j) ***N*-benzyl-2-(1-benzyl-5-chloro-3-hydroxy-2-oxoindolin-3-yl)-2-phenylacetamide (16)** 110

k) ***N,N'*-dibenzyl-2-(5-bromo-3-hydroxy-2-oxoindolin-3-yl)-2-phenylacetamide (17)** 116

l) ***N,N'*-dibenzyl-2-(3-hydroxy-2-oxo-5-trifluoromethoxyindolin-3-yl)-2-phenylacetamide (18)** 122

m) ***N,N'*-Dibenzyl-2-(3-hydroxy-5-methoxy-2-oxoindolin-3-yl)-2-phenylacetamide (19)** 129

n) ***N,N'*-Dibenzyl-2-(3-hydroxy-2-oxoindolin-3-yl)-2-*p*-anisylacetamide (20)** 140

o) ***N,N'*-Dibenzyl-2-(3-hydroxy-2-oxoindolin-3-yl)-2-*p*-tolylacetamide (21)** 146

p) ***N,N'*-Dibenzyl-2-(3-hydroxy-2-oxoindolin-3-yl)-2-*p*-chlorophenylacetamide (22)** 159

q) ***N,N'*-Dibenzyl-2-(3-hydroxy-2-oxoindolin-3-yl)-2-*p*-bromophenylacetamide (23)** 172

r) ***N,N'*-Dibenzyl-2-(3-hydroxy-2-oxoindolin-3-yl)-2-*m*-bromophenylacetamide (24)** 185

s) ***N,N'*-dibenzyl-2-(3-hydroxy-2-oxindolin-3-yl)-2-naphth-1-ylacetamide (25)** 198

t) ***N*-Benzyl 2-(1-benzyl-3-hydroxyoxindol-3-yl)-2-thiophen-3-ylacetamide (26)** 205

u) ***N*-Benzyl 2-(1-benzyl-3-hydroxyoxindol-3-yl)-5-methylhex-3-enamide (27)** 211

v) **1-Benzyl-7-chloro-3-hydroxy-3-(2-morpholino-2-oxo-1-phenylethyl)indolin-2-one (28)** 218

w) **1-Benzyl-6-chloro-3-hydroxy-3-(2-morpholino-2-oxo-1-phenylethyl)indolin-2-one (29)** 224

x) **1-Benzyl-5-chloro-3-hydroxy-3-(2-morpholino-2-oxo-1-phenylethyl)indolin-2-one (30)** 230

y) **1-(*p*-^{t}Butylbenzyl)-3-hydroxy-3-(2-morpholino-2-oxo-1-phenylethyl)indolin-2-one (31)** 236

IV. Syntheses of Anhydrides 242

a) **Phenylacetic anhydride (S1)** 242
b) ***para*-Anisylacetic anhydride (S2)** 244
c) ***para*-Tolylacetic anhydride (S3)** 246
d) ***para*-Chlorophenylacetic anhydride (S4)** 248
e) **(*para*-Bromophenyl)acetic anhydride (S5)** 250
f) ***meta*-Bromophenylacetic anhydride (S6)** 252
g) **Hydratropic Anhydride (kk47-S7)** 254
V. Syntheses of Isatins 256
a) ***N*-Benzylisatin (S8)** 256
b) ***N*-Benzyl-7-trifluoromethylisatin (S9)** 258
c) ***N*-Benzyl 7-Chloroisatin (S10)** 261
d) ***N*-Benzyl 6-Chloroisatin (S11)** 263
e) ***N*-Benzyl-5-chloroisatin (S12)** 265
f) ***N*-Benzyl-4-chloroisatin (S13)** 267
g) ***N*-Benzyl-5-bromoisatin (S14)** 269
h) ***N*-Benzyl-5-trifluoromethoxyisatin (S15)** 271
i) ***N*-Benzyl-5-methoxyisatin (S16)** 274
j) ***N*-(*para*-*tert*-Butylbenzyl)isatin (S17)** 276
k) ***N*-Methylisatin (S18)** 278
l) ***N*-Allylisatin (S19)** 280
m) ***N*-*tert*-Butoxycarbonylisatin (S20)** 282
VI. Unsuccessful examples 284
VII. References 285

I. General Procedures

Reactions involving moisture sensitive reagents were carried out in flame-dried glassware under an inert atmosphere (Ar or N_2) using standard vacuum line techniques. Anhydrous solvents (Et_2O, CH_2Cl_2, THF and PhMe) were obtained after passing through an alumina column (Mbraun SPS-800) or purchased in a sealed bottled under inert atomsphere. Organometallic reagents were titrated before use according to literature procedures.[1] Room temperature (r.t.) refers to 18 ± 3 °C, Petrol refers to petroleum ether with the boiling range of 40 – 60 °C, brine refers to saturated aqueous sodium chloride solution, ether refers to diethylether (Et_2O). All chemicals and solvents used were purchased by pertinent brands (Sigma Aldrich, Alfa Aesar, Acros, Apollo Scientific, TCI, STREM) and used without further purification unless stated. For reactions conducted during the day following cooling baths were applied: 0 °C (ice/water), −10 °C (ice/acetone), −20 °C (ice/NaCl), −45 °C (CO_2(s) or N_2(l)/MeCN), −60 °C (CO_2(s) or N_2(l)/$CHCl_3$) and −78 °C (CO_2(s)/acetone). Temperatures of 0 °C to −78 °C for overnight reactions were obtained using an immersion cooler (HAAKE EK 90). Reactions involving heating were performed using DrySyn blocks or oil baths and a contact thermocouple. Under reduced pressure refers to the use of either a Büchi Rotavapor R-200 with a Büchi V491 heating Bath and Büchi V-800 vacuum controller, a Büchi Rotavapor R-210 with a Büchi V-491 heating bath and Büchi V-850 vacuum controller, a Heidolph Laborota 4001 with vacuum controller, an IKA RV10 rotary evaporator with an IKA HB10 heating bath and ILMVAC vacuum controller, or an IKA RV10 rotary evaporator with an IKA HB10 heating bath and Vacuubrand CVC3000 vacuum controller. Rotary evaporator condensers are fitted to Julabo FL601 Recirculating Coolers filled with ethylene glycol and set to −6 °C.

Analytical thin layer chromatography (TLC)[2] was performed on pre-coated aluminium plates (Kieselgel 60 F_{254} silica) plates purchased from Merck. Visualisation was achieved using ultraviolet light (254 nm) and staining with aqueous $KMnO_4$ or ethanolic vanillin solution followed by heating. Flash column chromatography was performed in glass columns fitted with porosity 3 sintered discs over Silica gel 60 (0.043 – 0.060 mm) using standard techniques as reported in literature with the solvent system stated.[3] Automated chromatography was performed on a Biotage® Selekt™ SEL-2SV with a 200 – 400 nm UV-detector using the method stated and Biotage® Sfär™ Silica HC D or Biotage® Sfär™ Silica D columns.

HPLC analyses were obtained on either a Shimadzu HPLC consisting of a DGU-20A5 degassing unit, LC-20AT liquid chromatography pump, SIL-20AHT autosampler, CMB-20A communications bus module, SPD-M20A diode array detector and a CTO-20A column oven or a Shimadzu HPLC consisting of a DGU-20A5R degassing unit, LC-20AD liquid chromatography pump, SIL-20AHT autosampler, SPD-20A UV/Vis detector and a CTO-20A column oven. Separation was achieved using either DAICEL CHIRALCEL OD-H and OJ-H columns or DAICEL CHIRALPAK AD-H, AS-H, IA, IB, IC and ID columns using the method stated. HPLC traces of enantiomerically enriched compounds were compared with authentic racemic spectra. Racemic compounds were synthesised under analogous reaction conditions using achiral or racemic catalysts where necessary.

Optical rotations were determined using a Perkin Elmer Precisely/Model-341 Polarimeter with a Na/Hal lamp (Na D line, 589 nm) at 20 °C.

Infrared spectra were recorded on a Shimadzu IRAffinity-1 Fourier transform IR spectrophotometer fitted with a Specac Quest ATR accessory (diamond puck). Spectra were recorded of either thin films or solids, with characteristic absorption wavenumbers (ν_{max}) reported in cm^{-1}.

^1H, ^{13}C, ^{19}F and ^{32}P nuclear magnetic resonance (NMR) spectra were recorded with Bruker Avance™ 300 Cryomagnet with a BBFO probe, Bruker Avance II™ 400 Ultrashield with a BBFO probe, Bruker Avance™ 500 Ultrashield with a SmartProbe BBFO+ probe or Bruker Avance III™ 500 Ascend™ with a CryoProbe Prodigy BBO probe using deuterated solvents ($CDCl_3$, CD_2Cl_2, D_2O, CD_3OD, CD_3CN, $(CD_3)_2SO$, $(CD_3)_2CO$, $C_6D_5CD_3$) purchased from Sigma-Aldrich. Chemical shifts (δ) are quoted in ppm

and referenced to residual solvent signals reported in literature.[4] $^{13}C\{^1H\}$ and $^{19}F\{^1H\}$ spectra were acquired using a proton broadband decoupling sequence. ^{13}C were recorded with DEPTQ or UDEFT sequences. Couplings were indicated by the use of conventional agreed abbreviations: s (singlet), d (doublet), t (triplet), q (quartet), m (multiplet), dd (doublet of doublets), td (triplet of doublets), etc. Coupling constants (*J*) are denoted with the number of bonds involved in the upper left and with the atoms coupling in the bottom right edge of the symbol, e.g. $^3J_{HH}$. The abbreviation *Ar* denotes aromatic and app denotes apparent.[5] NMR peak assignments were confirmed using 2D ^1H correlated spectroscopy (COSY), ^1D selective ^1H nuclear Overhauser effect spectroscopy (NOESY), 2D ^1H–^{13}C heteronuclear multiple-bond correlation spectroscopy (HMBC), and 2D ^1H–^{13}C heteronuclear single quantum coherence (HSQC) where necessary. For analysis of NMR-spectra MestReNova and tools therein were used.[6] For Karplus analysis transformed equation 2 was used.

Melting points were recorded on an Electrothermal 9100 melting point apparatus and are not corrected; (dec) refers to decomposition.

Mass spectrometry (*m*/*z*) data were acquired using ThermoFisher Exactive Orbitrap mass spectrometer or Micromass GCT (TOF) mass spectrometer with solids probe. Ionisation techniques used are indicated for each compound. Values are quoted as a ratio of mass to charge (*m*/*z*) in Daltons [Da].[7]

Common chemical abbreviations were used to indicate chemical groups or environments such as Ph (phenyl), *Ar* (aromatic, not confuse with Argon), Bn (benzyl), Et (ethyl), Me (methyl). To indicate atoms numbering schemes are displayed with the spectrum and deviate from IUPAC numbering for clarity. For names and numbering concerning stereodiscriptors IUPAC nomenclature was applied.[8]

II. Optimisation of Reaction Conditions

General procedure for the isothiourea-catalysed [2+2] cycloddition of phenylacetic anhydride and *N*-benzylisatin: To a 25 ml round bottmed flask was added *N*-benzylisatin (1.0 equiv, 0.250 mmol, 59.3 mg) and the respective amounts of (2*R*, 3*S*)-HyperBTM and phenylacetic anhydride according to the table below. The corresponding solvent was added in the specified amount followed by the addition of Hünig's base. The reaction was stirred at the specified time and temperature and was then quenched by the addition of benzylamine. To this mixture was added 500 µl of a 0.1 M solution of 1,3,5-trimethoxybenzene in CH_2Cl_2. The solvent was removed *in vacuo* and the residue was submitted to ^{1}H NMR analysis.

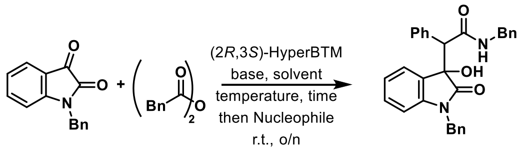

entry	Catalyst [mol%]	$BnNH_2$ [eqiuv]	anhydride [equiv]	Hünig's base [equiv]	solvent	molarity [M]	[2+2] time [h]	temperature [°C]	d.r. (2'*R*,3*R*):(2'*S*,3*R*)	Yield [%] (2'*R*,3*R*)/(2'*S*,3*R*)	e.r. (2'*R*,3*R*):(2'*S*,3*R*)
1	5	1	2.5	1.25	CH_2Cl_2	0.04	24	r.t.	76:24	32 / N/A	>99:1 / N/A
2	5	3	2.5	1.25	CH_2Cl_2	0.04	24	r.t.	76:24	66 / 21	>99:1 / >99:1
3	5	3	2.5	1.25	CH_2Cl_2	0.04	3	r.t.	69:31	63 / 25	>99:1 / >99:1
4	5	3	2.5	1.25	CH_2Cl_2	0.04	24	0	70:30	50 / N/A	>99:1 / N/A
5	5	3	2.5	1.25	CH_2Cl_2	0.04	20	0	67:33	57 / 22	98:2 / >99:1
6	5	3	2.5	1.25	CH_2Cl_2	0.04	3	0	67:33	47 / 23	>99:1 / >99:2
7	5	3	2.5	1.25	CH_2Cl_2	0.04	1	0	83:17	57 / 12	>99:1 / >99:1
8	5	3	2.5	1.25	CH_2Cl_2	0.04	3	-20	82:18	72 / 16	>99:1 / >99:1
9	5	3	1.5	1.25	CH_2Cl_2	0.04	1	0	80:20	59 / 15	>99:1 / >99:1
10	5	3	1.5	1.25	CH_2Cl_2	0.04	2	0	77:23	64 / 19	>99:1 / >99:1
11	5	3	1.5	1.25	CH_2Cl_2	0.04	4	0	76:24	64 / 20	>99:1 / >99:1
12	5	3	1.5	1.25	CH_2Cl_2	0.04	6	0	74:26	61 / 21	>99:1 / >99:1
13	5	3	1.5	1.25	CH_2Cl_2	0.04	3	0	76:24	66 / 21	>99:1 / >99:1
14	5	3	1.0	1.25	CH_2Cl_2	0.04	3	0	76:24	56 / 17	>99:1 / >99:1
15	1	3	1.5	1.25	CH_2Cl_2	0.04	3	0	76:24	44 / 14	>99:1 / >99:1
16	5	3	1.5	0.00	CH_2Cl_2	0.04	3	0	>95: 5	8 / N/A	97:3 / N/A
17	5	3	1.5	1.25	Anh. CH_2Cl_2	0.04	3	0	78:22	62 / 17	>99:1 / >99:1
18	5	3	1.5	1.25	Anh. THF	0.04	3	0	69:31	41 / 18	96:4 / N/A
19	5	3	1.5	1.25	EtOAc	0.04	3	0	80:20	62 / 18	98:2 / N/A
20	5	3	1.5	1.25	PhMe	0.10	3	0	78:22	54 / 15	>99:1 / >99:1
21	5	3	1.5	1.25	PhMe	0.25	3	0	71:29	31 / 13	>99:1 / >99:1
22	5	3	1.5	1.25	PhMe	0.50	3	0	66:34	25 / 13	>99:1 / >99:1
23	5	3	1.5	1.25	PhMe	1.00	3	0	72:28	16 / 6	>99:1 / >99:1

a) Scheme 2a

Attempted Preparation of (3*R*,3'*R*)-1-benzyl-3'-phenylspiro[oxindole-3,2-oxetan[4]one]:

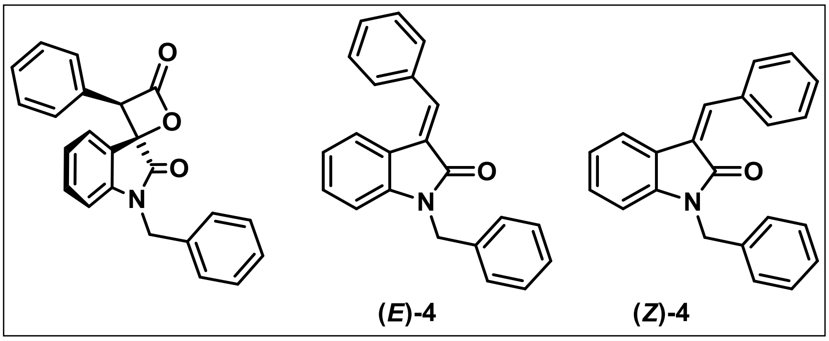

To a 25 ml round bottomed flask charged with *N*-benzylisatin (37.0 mg, 0.156 mmol) and phenylacetic anhydride (100.0 mg, 0.393 mmol) was added CH_2Cl_2 (5.50 ml, 0.03 M). To this solution was added (2*R*,3*S*)-HyperBTM (2.4 mg, 0.008 mmol) and Hünig's base (34.0 µL, 0.195 mmol) The solution was stirred at room temperature for 4 hours and was then concentrated *in vacuo* to yield the crude product which was purified by silica column chromatography (10% Et_2O in Petrol) to give the two alkenes as yellow oils (**(*E*)-4**: 18.5 mg, 38%; **(*Z*)-4**: 9.8 mg, 20%, co-eluted with 1,3,5-trimethoxybenzene) with data in accordance with the literature.[9]

4 (*E*)-1-benzyl-3-benzylideneindolin-2-one ((*E*)-4)

^{1}H NMR (400 MHz, $CDCl_3$) δ_H 7.95 (1H, s, C=C*H*-Ph), 7.71 – 7.63 (2H, m, ArC^4*H*, PhC*H*), 7.52 – 7.41 (3H, m, PhC*H*), 7.39 – 7.24 (5H, m, PhC*H*), 7.16 (1H, app td, $^3J_{HH}$ = 7.9 Hz, 7.7 Hz, $^4J_{HH}$ = 1.2 Hz, ArC^6*H*), 6.85 (1H, app td, $^3J_{HH}$ = 7.7 Hz, $^4J_{HH}$ = 1.0 Hz, ArC^5*H*), 6.74 (1H, d, $^3J_{HH}$ = 7.9 Hz, ArC^7*H*), 5.01 (2H, s, C*H*$_2$).

(*Z*)-1-benzyl-3-benzylideneindolin-2-one ((*Z*)-4)

^{1}H NMR (400 MHz, $CDCl_3$) δ_H 8.35 – 8.29 (2H, m, C=CH-PhC^{2,6}*H*), 7.60 (1H, s, C=C*H*-Ph), 7.55 (1H, app dt, $^3J_{HH}$ = 7.7 Hz, $^4J_{HH}$ = 1.2 Hz, $^5J_{HH}$ = 0.8 Hz, Ar*H*), 7.50 – 7.40 (3H, m, PhC*H*), 7.37 – 7.22 (5H, m, PhC*H*), 7.19 (1H, app td, $^3J_{HH}$ = 7.7 Hz, $^4J_{HH}$ = 1.2 Hz, ArC^6*H*), 7.04 (1H, app td, $^3J_{HH}$ = 7.7, $^4J_{HH}$ = 1.0 Hz, ArC^5*H*), 6.73 (1H, app dt, $^3J_{HH}$ = 7.7 Hz, $^4J_{HH}$ = 1.0 Hz, $^5J_{HH}$ = 0.8 Hz, ArC^7*H*), 5.00 (2H, s, C*H*$_2$).

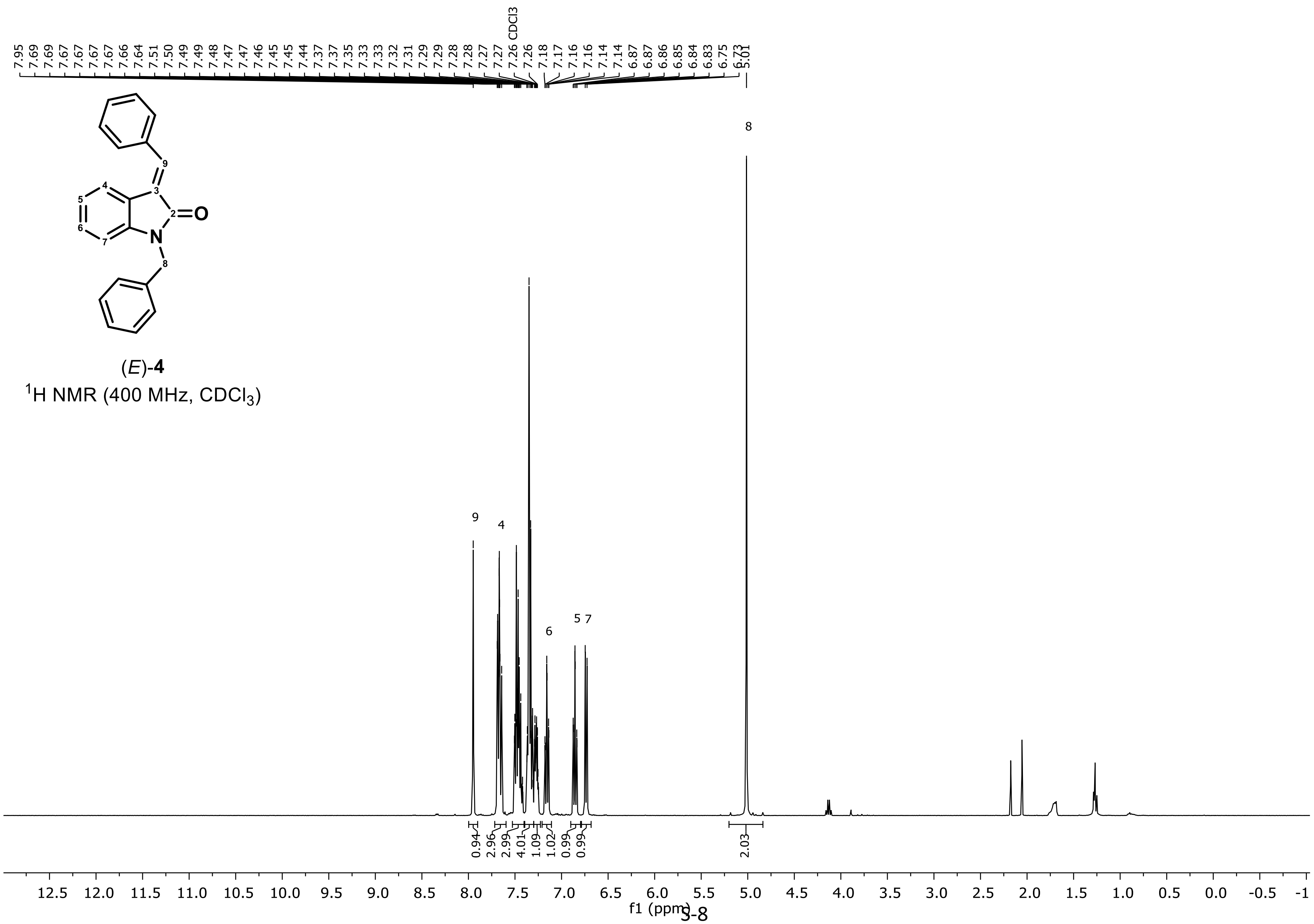

(*E*)-**4**

^{1}H NMR (400 MHz, $CDCl_3$)

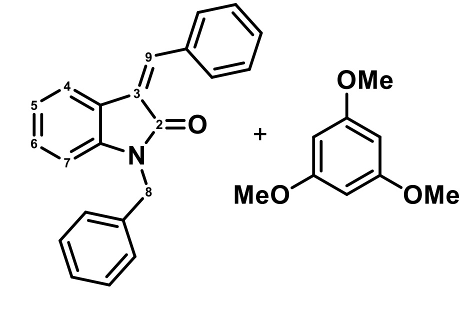

(Z)-**4**

^{1}H NMR (400 MHz, $CDCl_3$)

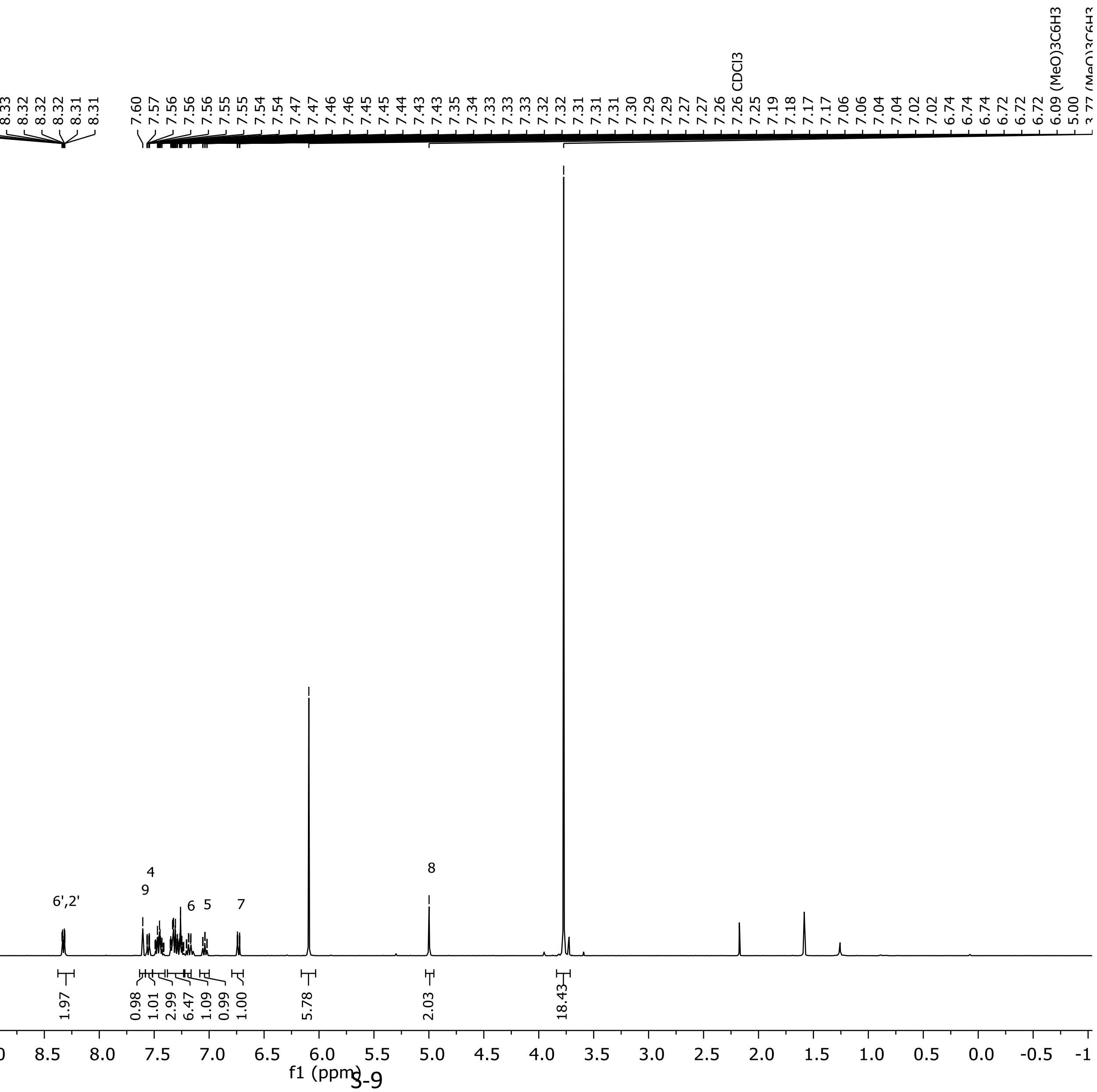

***b)* Scheme 2b**

Reaction Monitoring

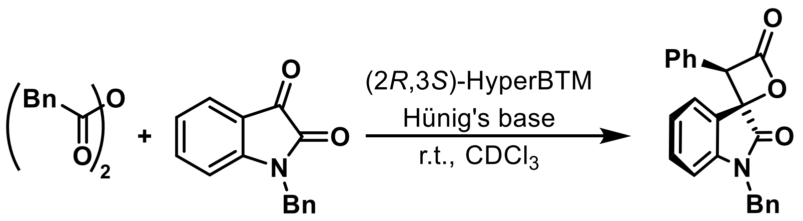

run I				run II			
entry	time [min]	Yield [%]	d.r.	entry	time [min]	Yield [%]	d.r.
1	10	68	100: 0	1	10	68	100: 0
2	21	71	100: 0	2	63	75	95: 5
3	51	76	91: 9	3	82	74	91: 9
4	63	76	89:11	4	103	76	89:11
5	80	76	88:12	5	135	77	87:13
6	121	75	85:15	6	165	75	85:15
7	145	75	83:17	7	190	74	84:16
8	169	76	82:18	8	1003	70	69:31

To a 25 ml round bottomed flask was added phenylacetic anhydride (95.3 mg, 0.375 mmol), *N*-benzylisatin (59.3 mg, 0.250 mmol), (2*R*,3*S*)-HyperBTM (3.9 mg, 0.012 mmol) and 1,3,5-trimethoxybenzene (16.8 mg, 0.1 mmol). $CDCl_3$ (6.0 ml, 0.04 M) and Hünig's base (54.0 µl, 0.312 mmol) were added at room temperature and 0.6 ml was added to an NMR tube. The NMR tube was loaded into an NMR spectrometer at 297 K. The sample was locked, shimmed and a ^{1}H spectrum was acquired (ns = 8, sweep width 14 ppm, spectral centre 6.5 ppm). Spectra were acquired at intervals and processed using MNova. The yield and d.r. of the lactone were recorded, and MS Excel was used to analyse these numbers.

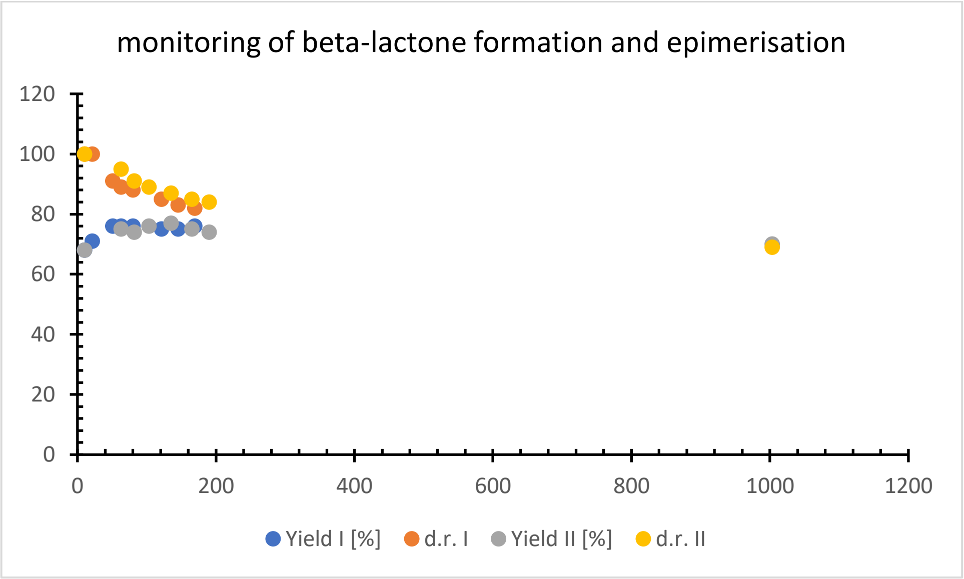

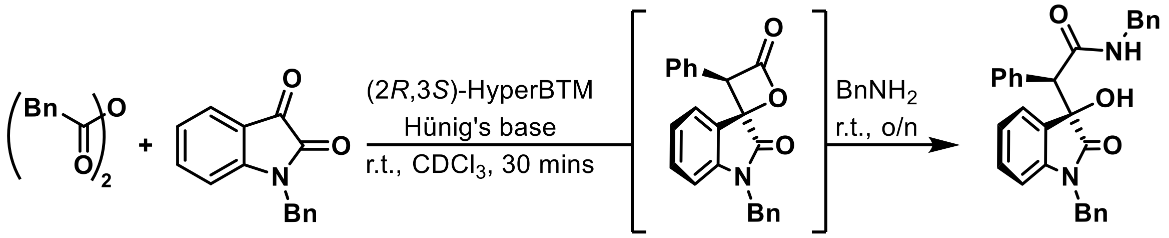

To a 25 ml round bottomed flask was added phenylacetic anhydride (95.3 mg, 0.375 mmol), *N*-benzylisatin (59.3 mg, 0.250 mmol), (3*R*,3*S*)-HyperBTM (3.9 mg, 0.012 mmol) and 1,3,5-trimethoxybenzene (16.8 mg, 0.1 mmol). $CDCl_3$ (6.0 ml, 0.04 M) and Hünig's base (54.0 µl, 0.312 mmol) were added at room temperature and 0.6 ml was added to an NMR tube. The NMR tube was loaded into an NMR spectrometer at 297 K. The sample was locked, shimmed and a ^{1}H spectrum acquired. (ns = 8, sweep width 14 ppm, spectral centre 6.5 ppm). After 30 minutes, the sample was returned to the round bottom flask and benzylamine (82.0 µl, 0.75 mmol) was added. Again, 0.6 ml of the solution were added to an NMR tube and spectra recorded at regular intervals. The yield and d.r. of the lactone were recorded and the presence of the ring-opened amine observed.

Reaction quench at 30 minutes

To a 25 ml round bottomed flask was added phenylacetic anhydride (95.3 mg, 0.375 mmol), *N*-benzylisatin (59.3 mg, 0.250 mmol), and (2*R*,3*S*)-HyperBTM (3.9 mg, 0.012 mmol). The flask was cooled to 0 °C and CH_2Cl_2 (6.0 ml, 0.04 M) and Hünig's base (54.0 µl, 0.312 mmol) were added and the reaction stirred for 30 minutes. Benzylamine (82.0 µl, 0.75 mmol) was added and the reaction stirred at room temperature for 16 h. ^{1}H NMR of the crude material only showed a single diastereomer (d.r. >95:5). The product was isolated (Biotage, 10 g SiO_2 column, EtOAc in petroleum ether 0-20% (5 CV), 20-25% (3 CV), 25-30% (6 CV), 35-60% (3 CV)) to give a single diastereomer in 34% yield.

III. Syntheses of 3-hydroxyoxindoles

a) ***N*-benzyl-2-(1-benzyl-3-hydroxy-2-oxoindolin-3-yl)-2-phenylacetamide (6 and 9)**

To a 25 ml round bottomed flask was added phenylacetic anhydride (95.3 mg, 0.375 mmol), *N*-benzylisatin (59.3 mg, 0.250 mmol), and (2*R*,3*S*)-HyperBTM (3.9 mg, 0.012 mmol). The mixture was cooled to 0 °C and CH_2Cl_2 (6.0 ml, 0.04 M) and Hünig's base (54 µl, 0.312 mmol) were added. The mixture was stirred at 0 °C for 3 h. Benzylamine (82 µl, 0.750 mmol) was added at 0 °C and the reaction was left to be stirred overnight at room temperature. 1,3,5-trimethoxybenzene (0.1 M soln in CH_2Cl_2, 500 µl, 0.05 mmol) was added and the solvent was removed under reduced pressure. Purification by column chromatography (20% – 25% EtOAc in Pentane) gave the title compound in two fractions (major diastereomer as white solid (71.2 mg, 62%) and minor diastereomer as white solid (22.4 mg, 19%); combined (93.6 mg, 0.202 mmol, 81%, 76:24 d.r.).

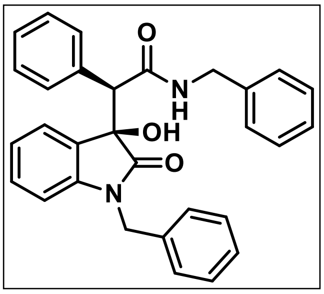

Major (2''''*R*,3*R*)-**6**: **R*f*** 0.31 (PET:EtOAc 6:4); **Chiral HPLC analysis** Chiralpak IB (95:5 hexane:IPA, flow rate 2 ml·min^{-1}, 211 nm, 40 °C) t_R (2''''*R*, 3*R*)-**6**: 20.4 min, t_R (2''''*S*, 3*S*)-**6**: 28.4 min, >99:1 er; $\alpha_D^{20} = -0.227$ (*c* 3.12, $CHCl_3$); ***ν*max** (thin film): 3337 (O–H), 3059 (N–H), 3030 (N–H), 1717 (C=O lactam), 1647 (C=C), 1612 (C=O amide), 1468 (CH2), 1175 (C-O alcohol); **^{1}H NMR** (500 MHz, $CDCl_3$) δ_H 7.49 (1H, d, $^3J_{HH}$ = 7.4 Hz, ArC^4*H*), 7.36 – 7.24 (4H, m, PhC$^{3'',4'',5''}$*H*, PhC$^{4'''}$*H*), 7.21 (2H, app d, $^3J_{HH}$ = 7.5 Hz, PhC$^{2''',6'''}$*H*), 7.18 – 7.11 (4H, m, ArC^6*H*, PhC$^{4'}$*H*, PhC$^{3''',5'''}$*H*), 7.09 (2H, app t, $^3J_{HH}$ = 7.4 Hz, PhC$^{3',5'}$*H*), 7.00 (1H, dd, $^3J_{HH}$ = 7.7 Hz, 7.4 Hz, ArC^5*H*), 6.91 (2H, app d, $^3J_{HH}$ = 7.7 Hz, PhC$^{2'',6''}$*H*), 6.83 (1H,s, O*H*), 6.54 (2H, app d, $^3J_{HH}$ = 7.4 Hz, PhC$^{2',6'}$*H*), 6.41 (1H, d, $^3J_{HH}$ = 7.8 Hz, ArC^7*H*), 5.91 (1H, t, $^3J_{HH}$ = 6.0 Hz, N*H*CH_2-Ph), 4.92 (1H, d, $^2J_{HH}$ = 16.0 Hz, NC$H_a$$H_b$-Ph), 4.53 (1H, dd, $^2J_{HH}$ = 15.0 Hz, $^3J_{HH}$ = 6.0 Hz, NHC$H_a$$H_b$-Ph), 4.48 (1H, dd $^2J_{HH}$ = 15.0 Hz, $^3J_{HH}$ = 6.0 Hz, NHC$H_a$$H_b$-Ph), 4.36 (1H, s, C*H*-Ph), 4.31 (1H, d, $^2J_{HH}$ = 16.0 Hz, NC$H_a$$H_b$-Ph); **^{13}C {^{1}H} NMR** (101 MHz, $CDCl_3$) δ_C 175.1 (N*C*(O)), 173.1 (NH*C*(O)), 143.2 (Ar*C*^{7a}), 137.6 (Ph*C*$^{1'''}$$CH_2$NH), 135.0 (Ph*C*$^{1'}$$CH_2$N), 132.9 (Ph*C*$^{1''}$CH), 130.6 (Ph*C*$^{2'',6''}$H), 129.9 (Ar*C*^{6}H), 129.5 (Ar*C*^{3a}), 128.7 (Ph*C*$^{3'',5''}$H), 128.6 (Ph*C*$^{4''}$H), 128.8_3 and 128.8_0 (Ph*C*$^{3'',5''}$H and Ph*C*$^{3''',5'''}$H), 127.7 (Ph*C*$^{4'''}$H), 127.6 (Ph*C*$^{2''',6'''}$H), 127.3 (Ph*C*$^{4'}$H), 126.6 (Ph*C*$^{2',6'}$H), 126.0 (Ar*C*^{4}H), 123.2 (Ar*C*^{5}H), 109.5 (Ar*C*^{7}H), 79.0 (*C*-OH), 57.1 (*C*H-Ph), 43.7_9 (NH*C*H_2-Ph), 43.7_5 (N*C*H_2-Ph); **HRMS** (ESI$^+$) *m/z* $[M+H]^+$ calcd for $C_{30}H_{27}N_2O_3$ 463.2016, found 463.2007 (−2.0 ppm).

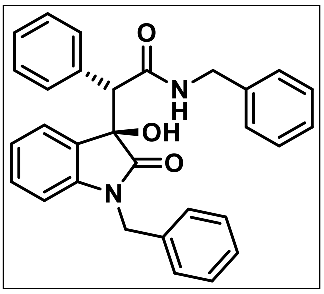

Minor (2''''*S*,3*R*)-**9**: **R*f*** 0.17 (PET:EtOAc 6:4); **Chiral HPLC analysis** Chiralpak IB (95:5 hexane:IPA, flow rate 2 ml·min^{-1}, 211 nm, 40 °C) t_R (2''''*S*, 3*R*)-**9**: 22.2 min, t_R (2''''*R*, 3*S*)-**9**: 31.3 min, >99:1 er**;** $\alpha_D^{20} = -1.069$ (*c* 0.29, $CHCl_3$); ***ν*max** (thin film) 3291 (O–H), 3061 (N–H), 3030 (N–H), 2922 (C–H), 1699 (C=O lactam), 1612 (C=O amide), 1468 (CH2), 1177 (C-O alcohol); **^{1}H NMR** (400 MHz, $CDCl_3$) δ_H 7.36 (1H, dd, $^3J_{HH}$ = 7.4 Hz, $^4J_{HH}$ = 1.3 Hz, ArC^4*H*), 7.35 – 7.25 (5H, m, PhC$^{4'}$*H*, PhC$^{2''',3''',5''',6'''}$*H*), 7.25 – 7.20 (3H, m, PhC$^{3',5'}$*H*, PhC$^{4'''}$*H*), 7.19 – 7.12 (1H, m, PhC$^{4''}$*H*), 7.10 – 7.01 (7H, m, ArC^6*H*, PhC$^{2',6'}$*H*, PhC$^{2'',3'',5'',6''}$*H*), 6.98 (1H, ddd, $^3J_{HH}$ = 7.7 Hz, 7.4 Hz, $^4J_{HH}$ = 1.1 Hz, ArC^5*H*), 6.94 (1H, s, O*H*), 6.71 – 6.60 (1H, m, N*H*), 6.42 (1H, app d, $^3J_{HH}$ = 7.8 Hz, ArC^7*H*), 4.84 (1H, d, $^2J_{HH}$ = 15.8 Hz, NC$H_a$$H_b$-Ph), 4.65 (1H, d, $^2J_{HH}$ = 15.8 Hz, NC$H_a$$H_b$-Ph), 4.57 (1H, dd, $^2J_{HH}$ = 15.0 Hz, $^3J_{HH}$ = 5.9 Hz, NHC$H_a$$H_b$-Ph), 4.51 (1H, dd, $^2J_{HH}$ = 15.0 Hz, $^3J_{HH}$ = 5.7 Hz, NHC$H_a$$H_b$-Ph), 4.26 (1H, s, C*H*-Ph); **^{13}C {^{1}H} NMR** (126 MHz, $CDCl_3$) δ_C 176.7 (N*C*(O)NBn), 172.3 (*C*(O)NHBn), 142.8 (Ar*C*^{7a}), 137.7 (Ph*C*$^{1'''}$$CH_2$NH), 135.3 (Ph*C*$^{1'}$$CH_2$N), 132.9 (Ph*C*$^{1''}$CH), 130.2 (Ph*C*$^{2'',6''}$H), 129.9 (Ar*C*^{6}H), 129.2 (Ar*C*^{3a}), 128.9_0, 128.8_5 and 128.7_8 (Ph*C*$^{3',5'}$H, Ph*C*$^{3'',5''}$H and Ph*C*$^{3''',5'''}$H), 128.3 (Ph*C*$^{4''}$H), 127.8 (Ph*C*$^{2''',6'''}$H), 127.6_6 and 127.6_4 (Ph*C*$^{4'}$H and Ph*C*$^{4'''}$H), 127.4 (Ph*C*$^{2',6'}$H), 124.1 (Ar*C*^{4}H), 123.1 (Ar*C*^{5}H), 109.4 (Ar*C*^{7}H), 78.7 (*C*-OH), 57.6 (*C*H-Ph), 43.9_5 and 43.9_3 (N*C*H_2-Ph and NH*C*H_2-Ph); **HRMS** (ESI$^+$) *m/z* $[M+Na]^+$calcd for $C_{30}H_{26}N_2NaO_3$ 485.1836, found 485.1827 (−1.9 ppm).

(±)-anti-**6**

PDA Ch1 211nm

Peak#	Ret. Time	Area%
1	20.627	49.968
2	28.079	50.032
Total		100.000

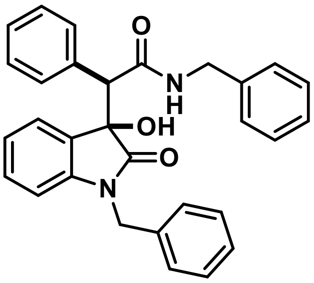

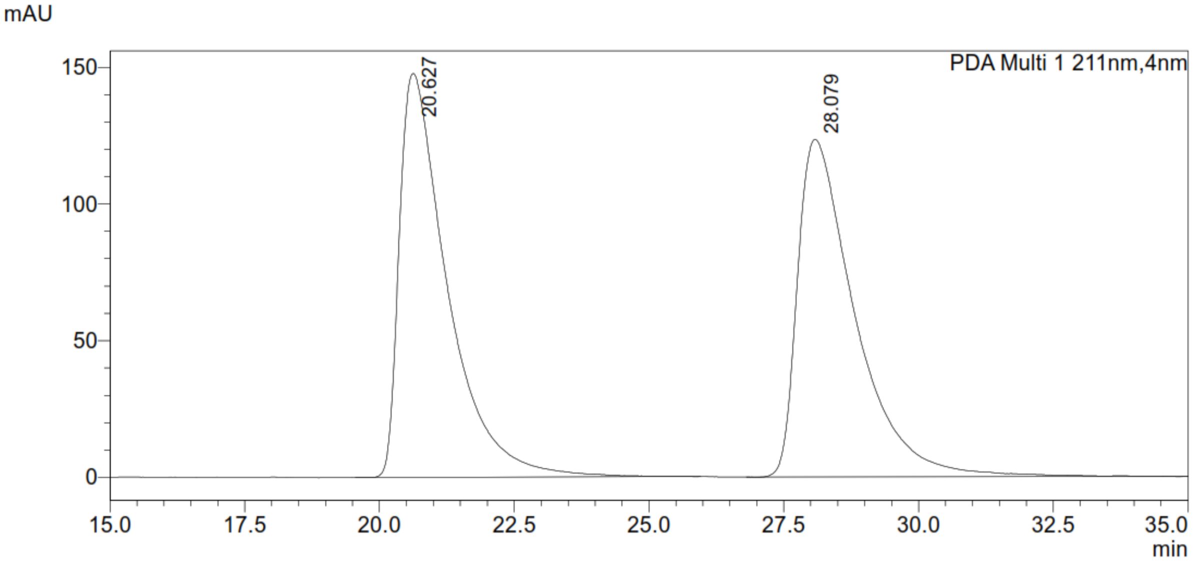

(–)-(2''''*R*,3*R*)-**6**

PDA Ch1 211nm

Peak#	Ret. Time	Area%
1	20.254	99.272
2	28.244	0.728
Total		100.000

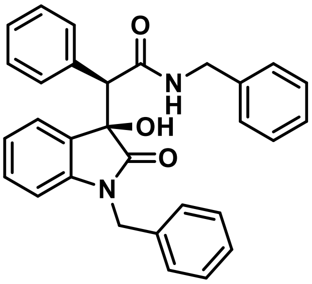

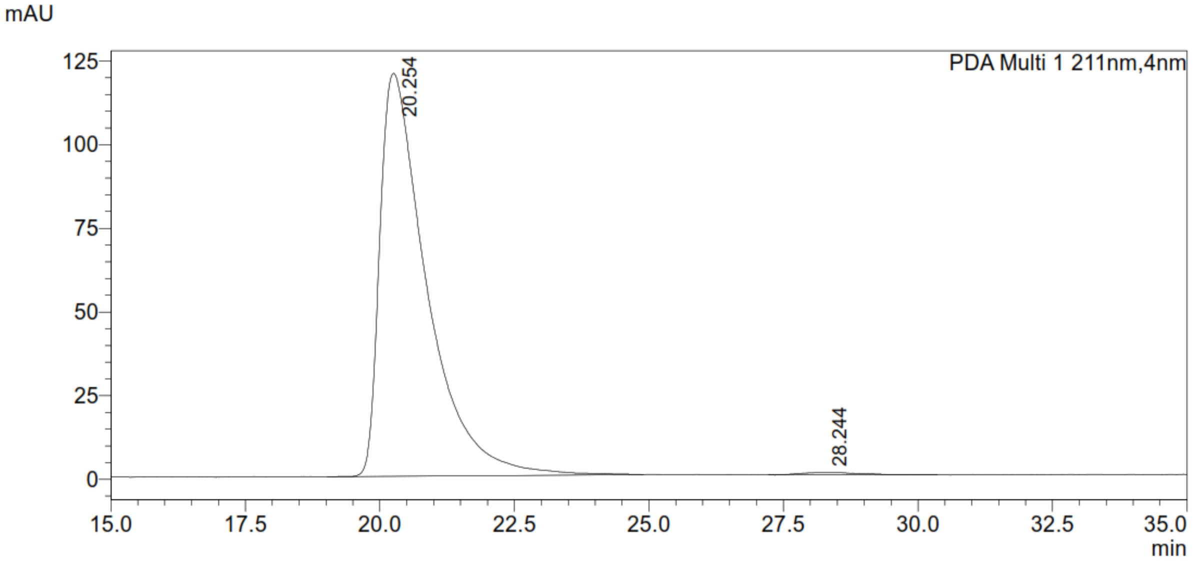

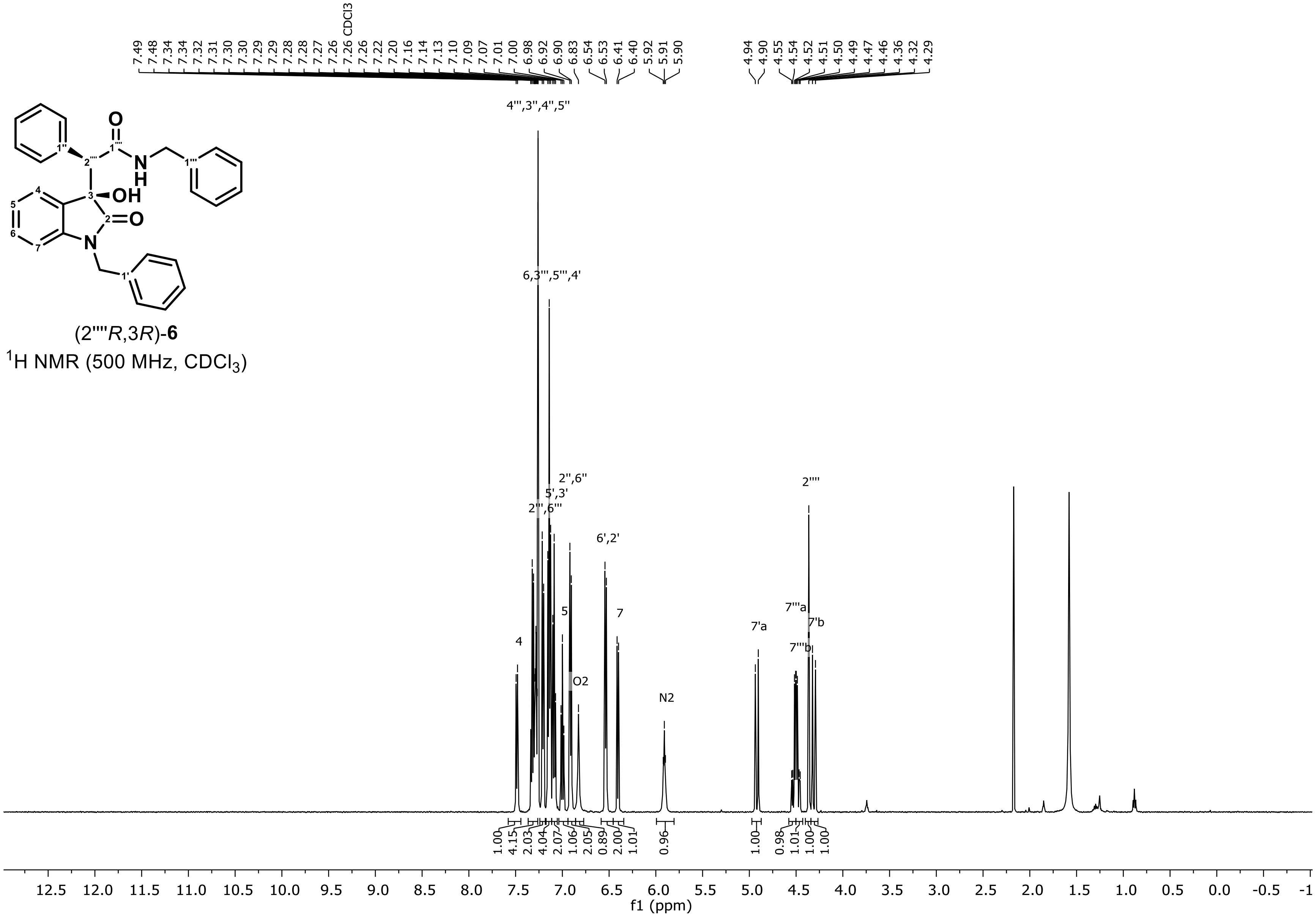

(2""*R*,3*R*)-**6**

^{1}H NMR (500 MHz, $CDCl_3$)

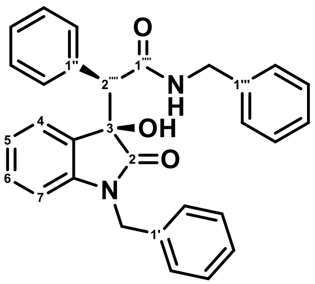

(2''''*R*,3*R*)-**6**

^{13}C {^{1}H} NMR (126 MHz, $CDCl_3$)

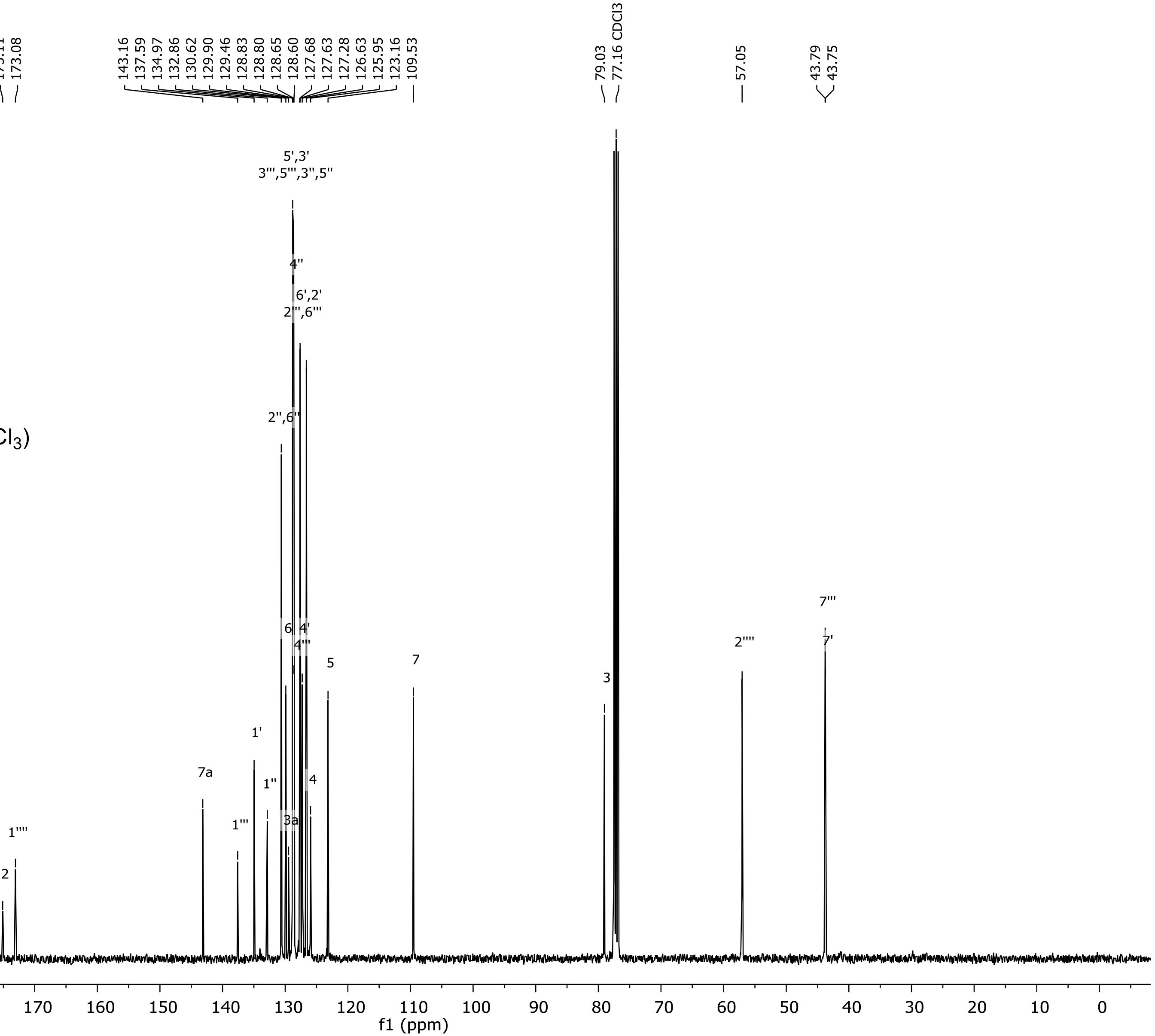

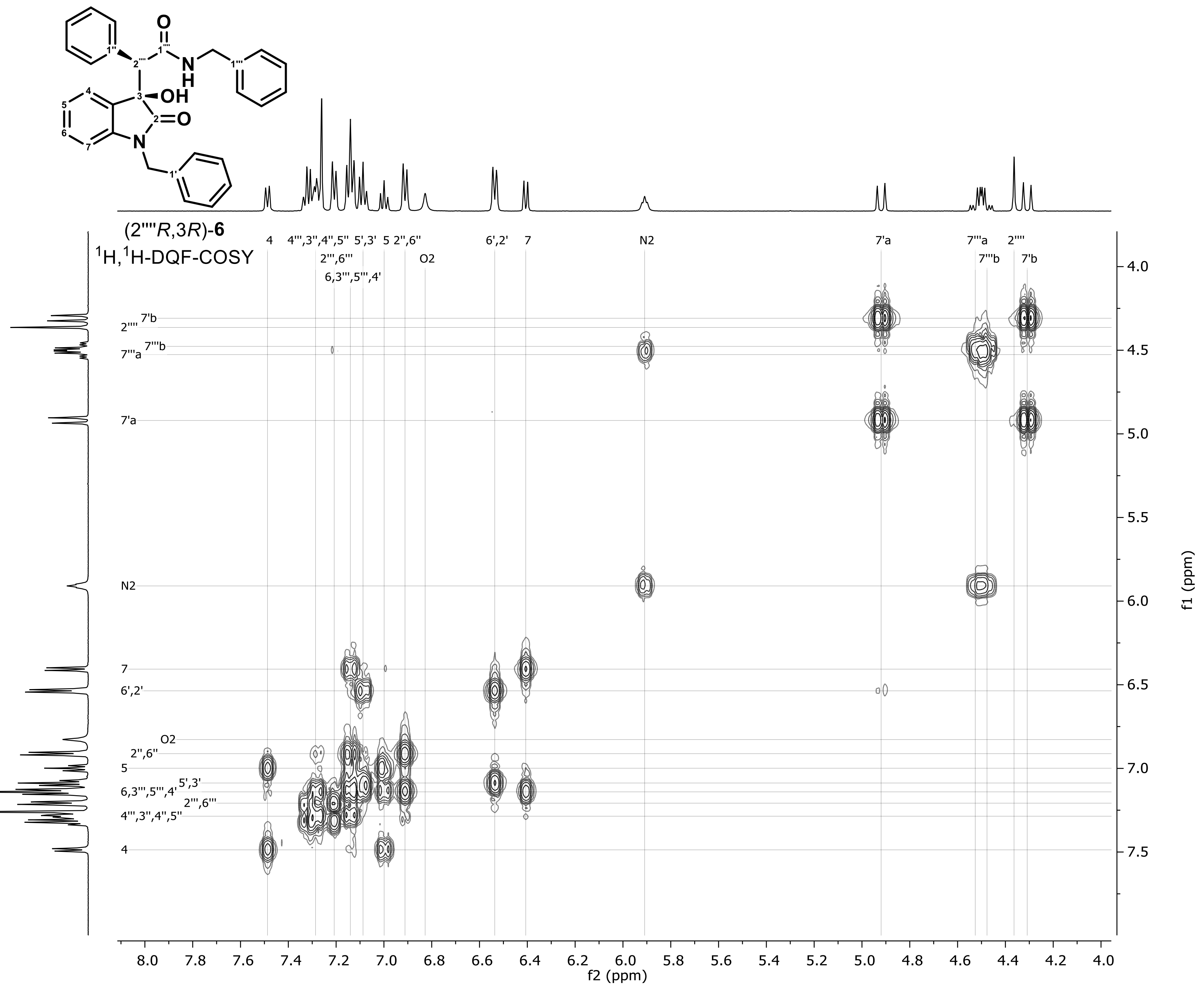
(2''''R,3R)-6
1H,1H-DQF-COSY
f2 (ppm)
f1 (ppm)

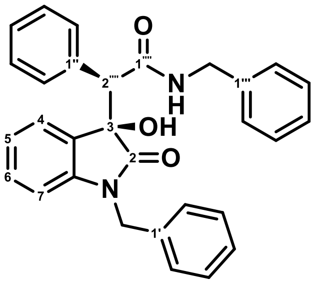

(2''''*R*,3*R*)-**6**

^{1}H,^{13}C-gs-HSQC w/ME

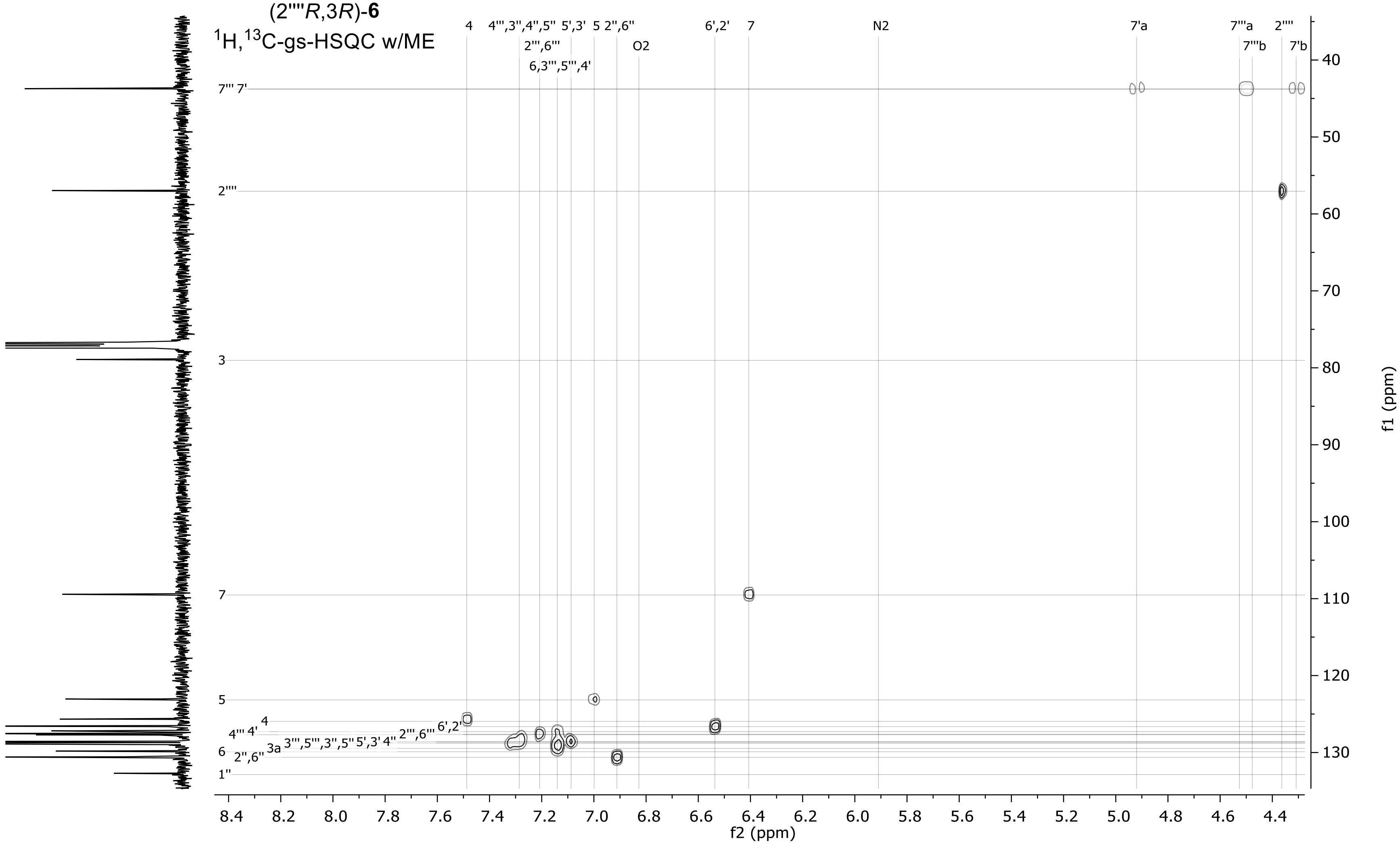

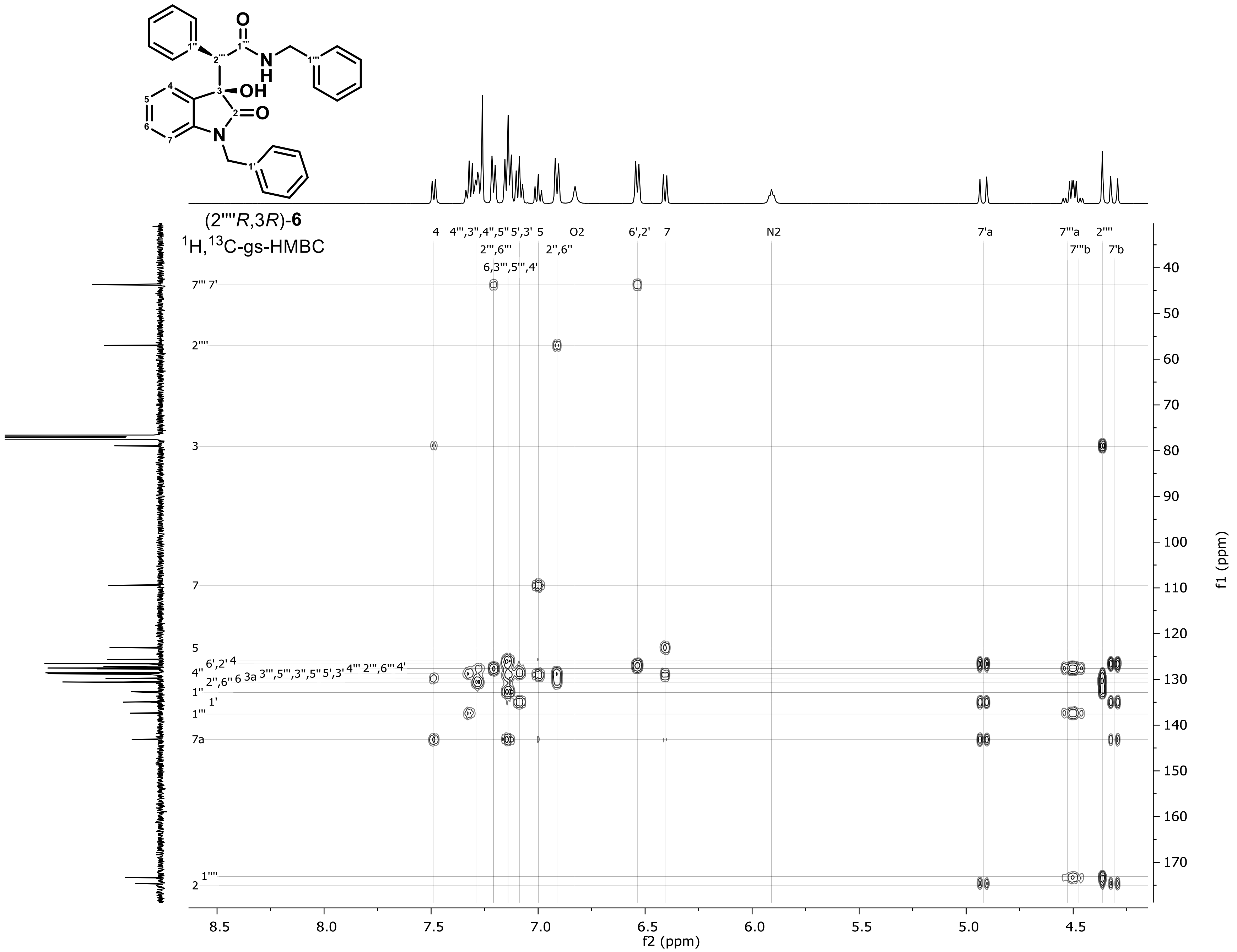

(2''''R,3R)-**6**

^{1}H,^{13}C-gs-HMBC

(±)-syn-**9**

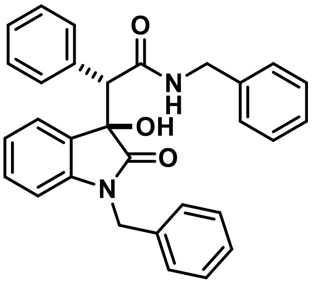

PDA Ch1 211nm

Peak#	Ret. Time	Area%
1	22.026	49.291
2	30.333	50.709
Total		100.000

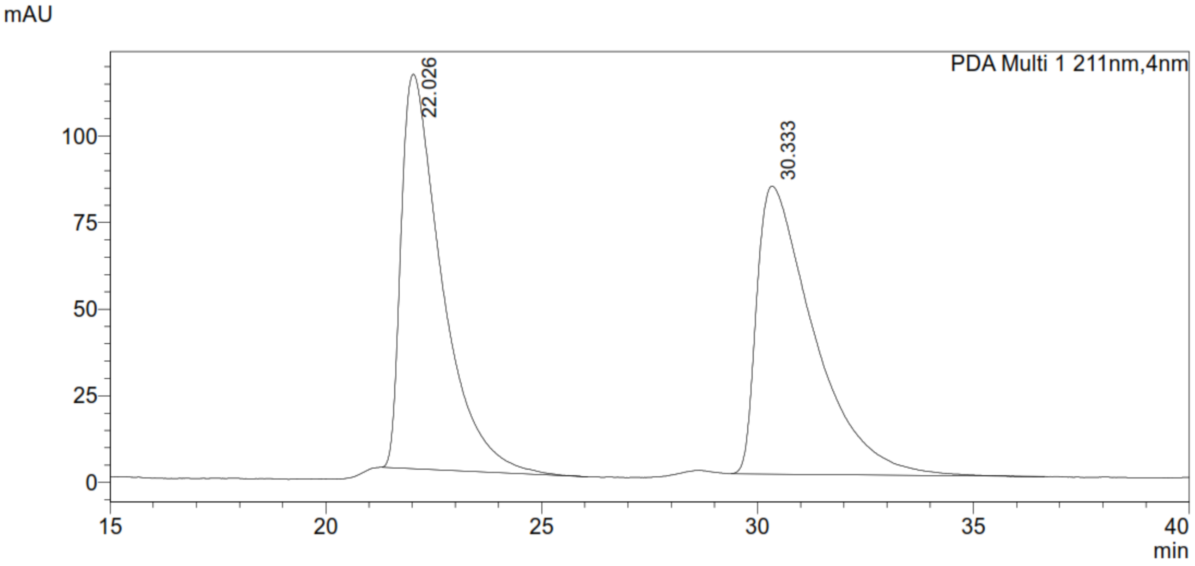

(–)-(2'''*S*,3*R*)-**9**

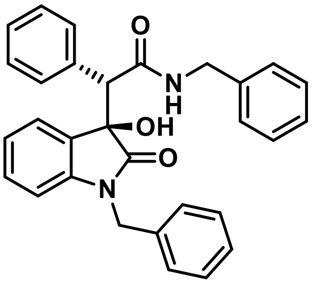

PDA Ch1 211nm

Peak#	Ret. Time	Area%
1	21.923	97.842
2	31.605	2.158
Total		100.000

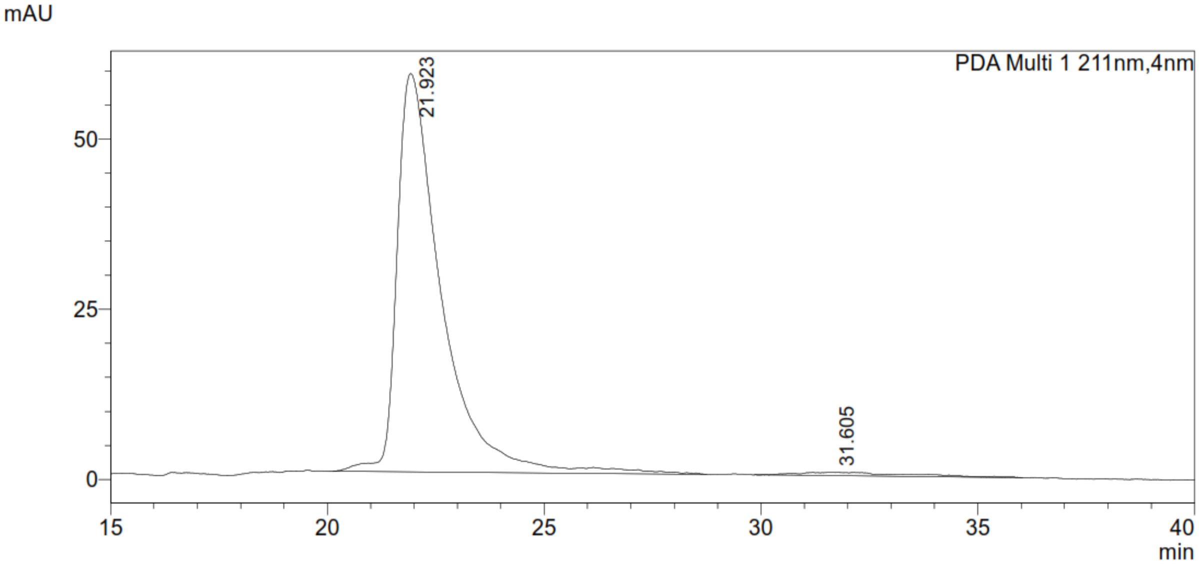

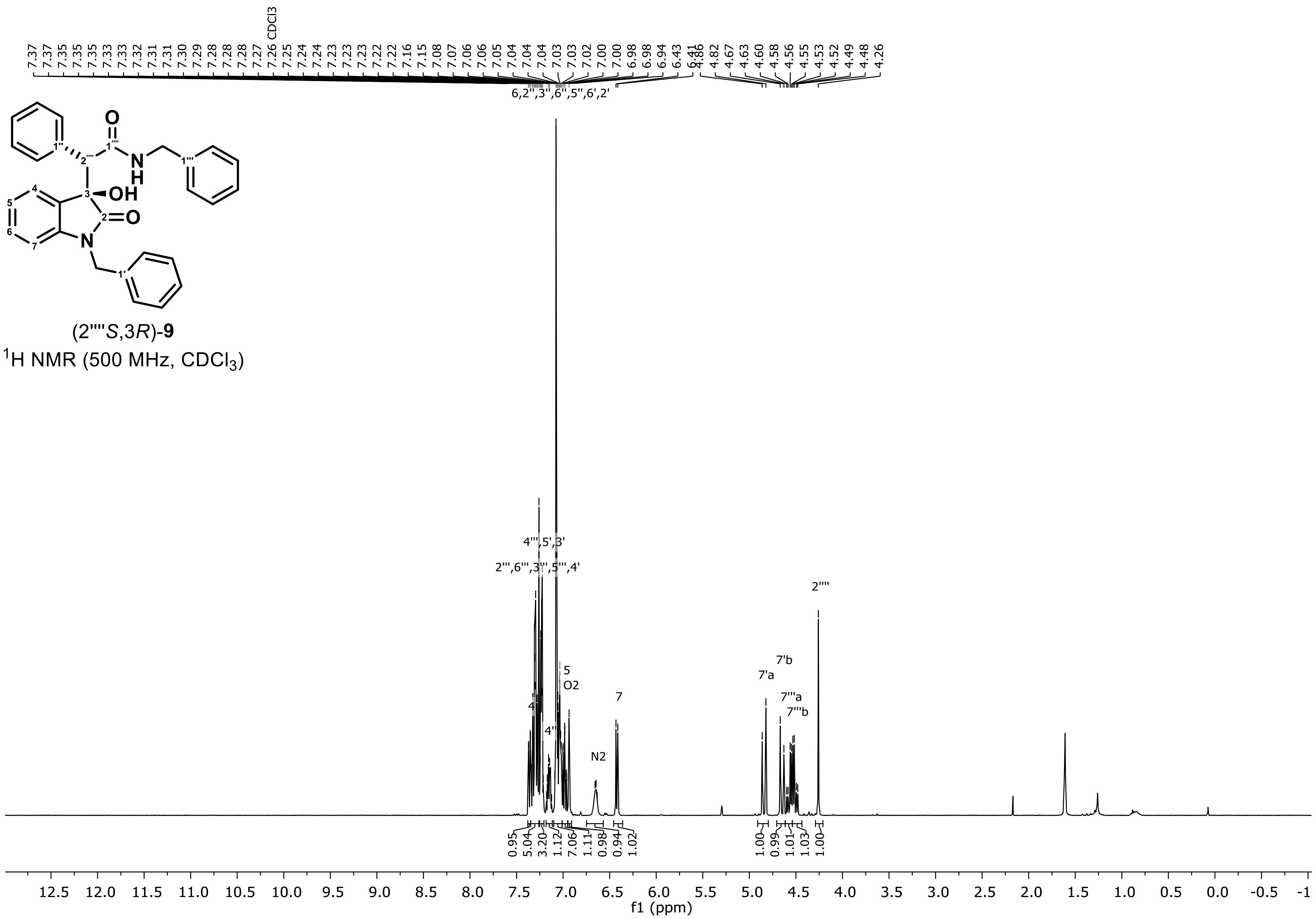

(2''''S,3R)-**9**

^{1}H NMR (500 MHz, $CDCl_3$)

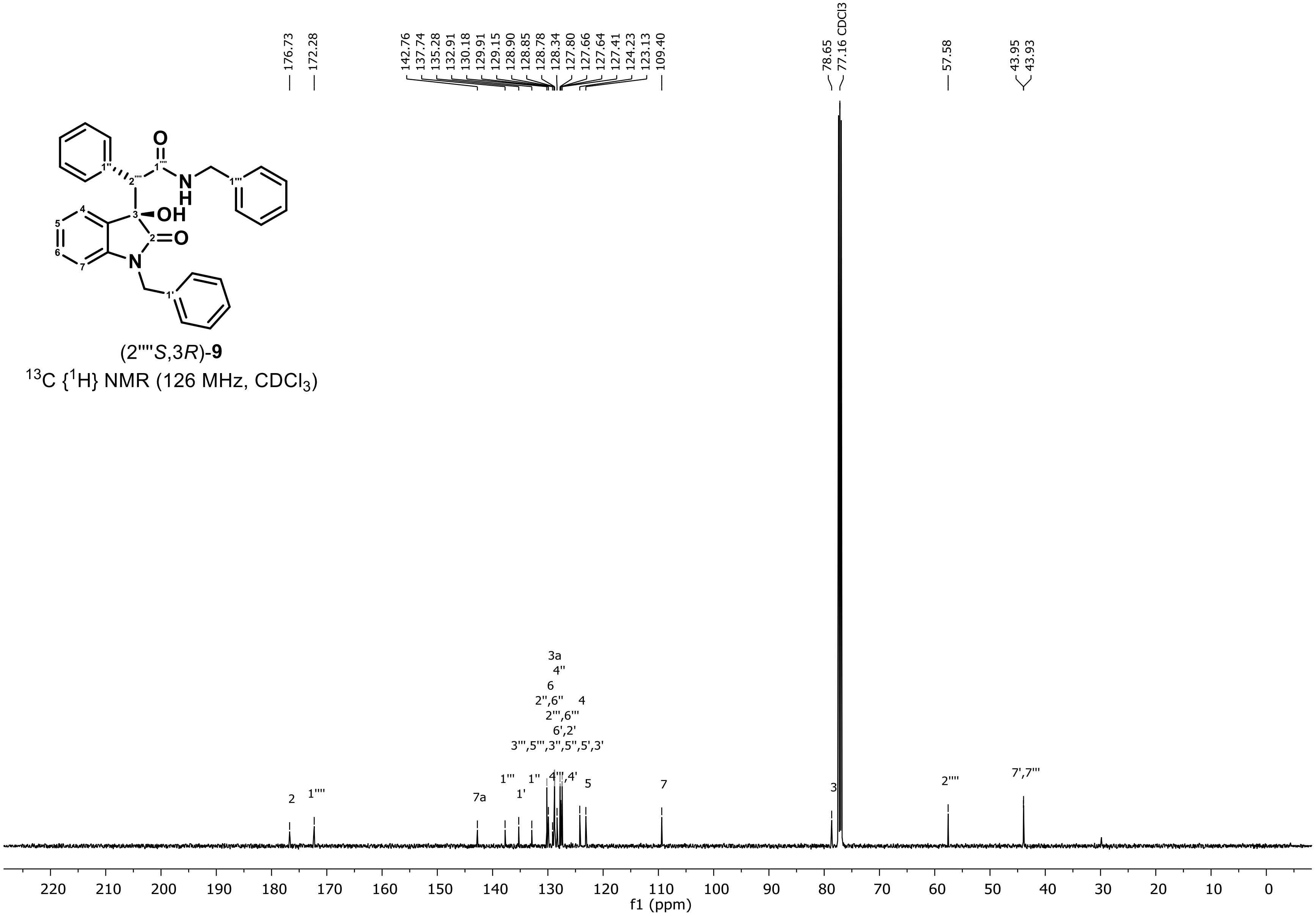
176.73
172.28
142.76
137.74
135.28
132.91
130.18
129.91
129.15
128.90
128.85
128.78
128.34
127.80
127.66
127.64
127.41
124.23
123.13
109.40
78.65
77.16 CDCl3
57.58
43.95
43.93
(2''''S,3R)-9
^{13}C {^{1}H} NMR (126 MHz, $CDCl_3$)
f1 (ppm)

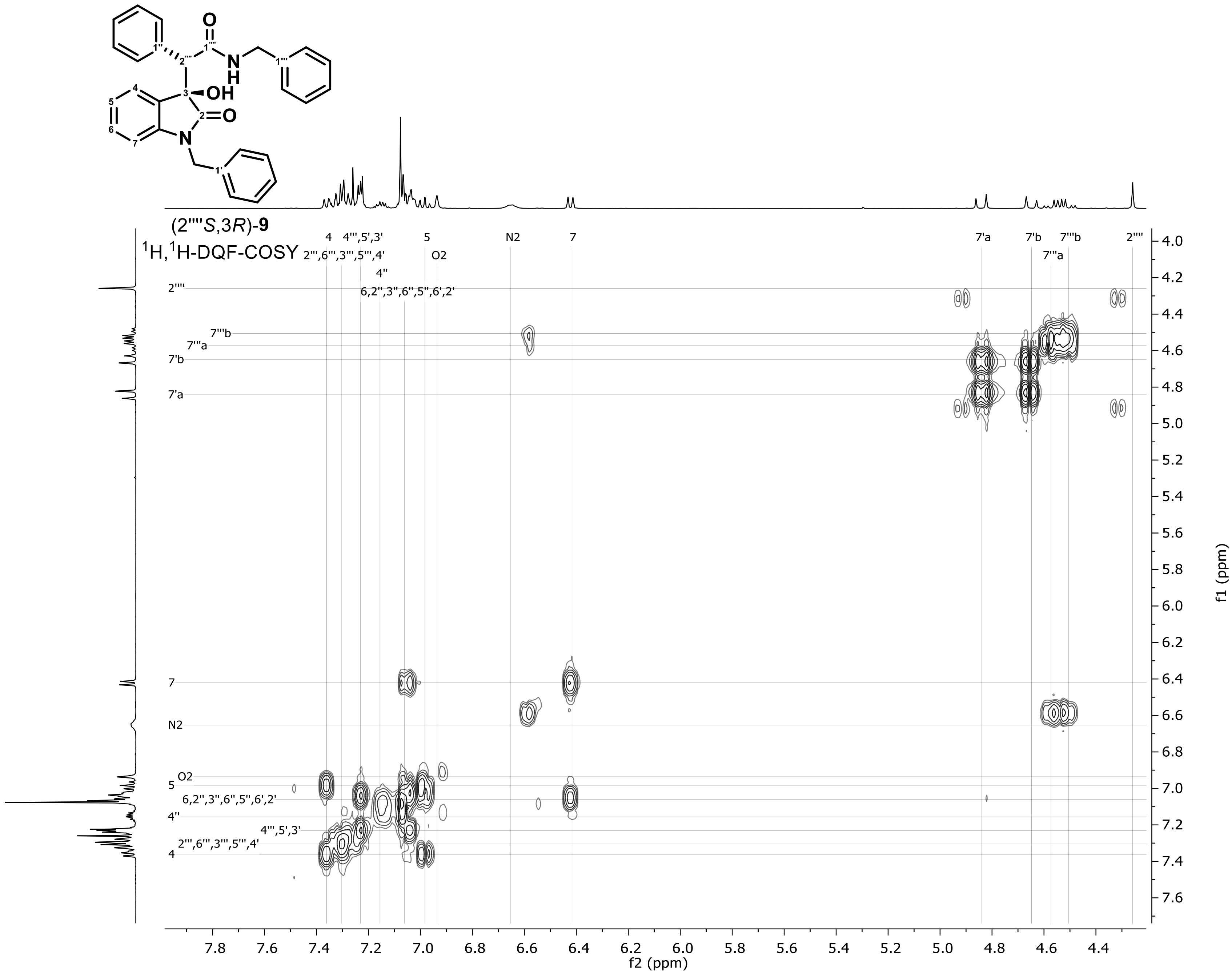
(2''''S,3R)-9
^{1}H,^{1}H-DQF-COSY
f2 (ppm)
f1 (ppm)

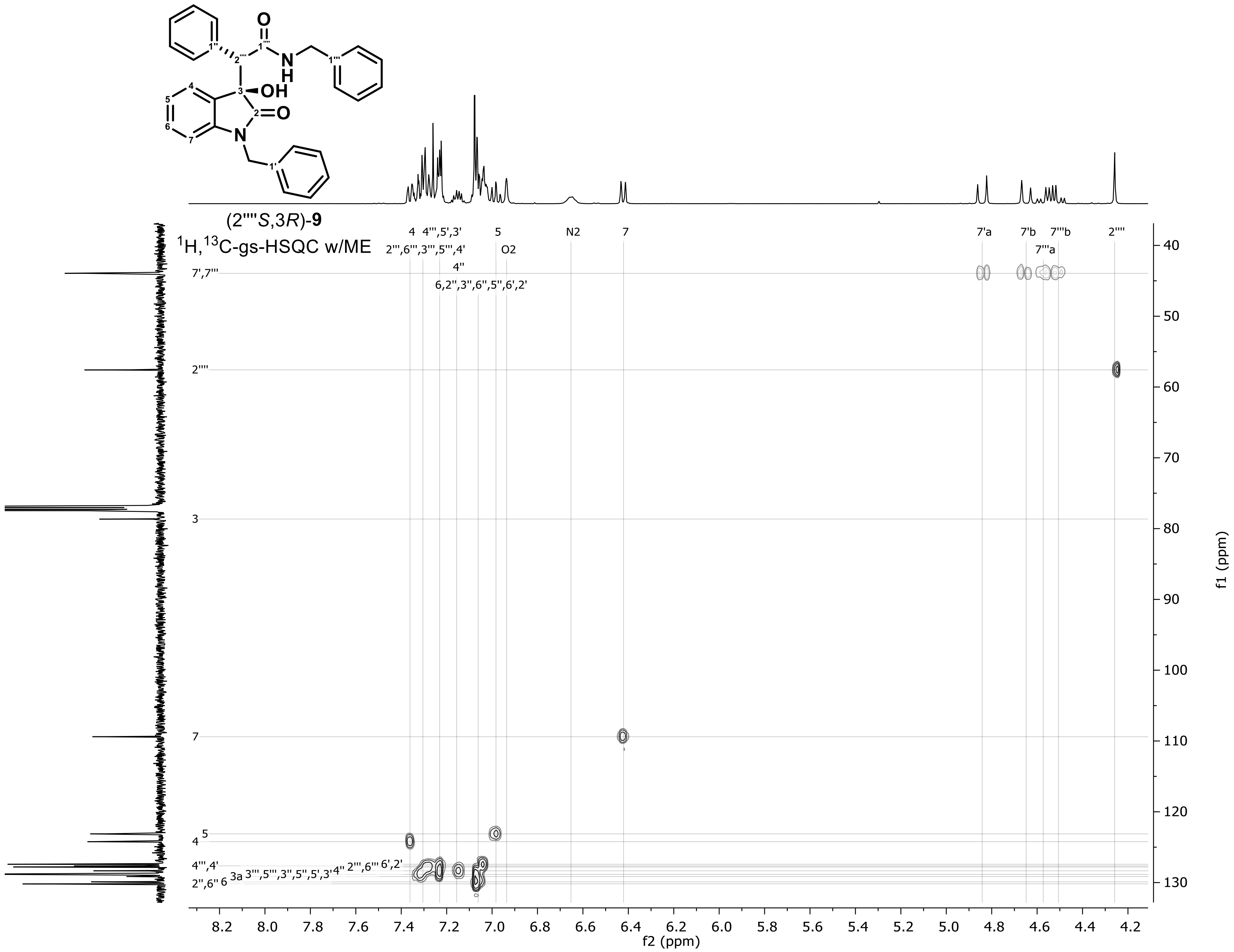
(2''''S,3R)-9
1H,13C-gs-HSQC w/ME
f2 (ppm)
f1 (ppm)

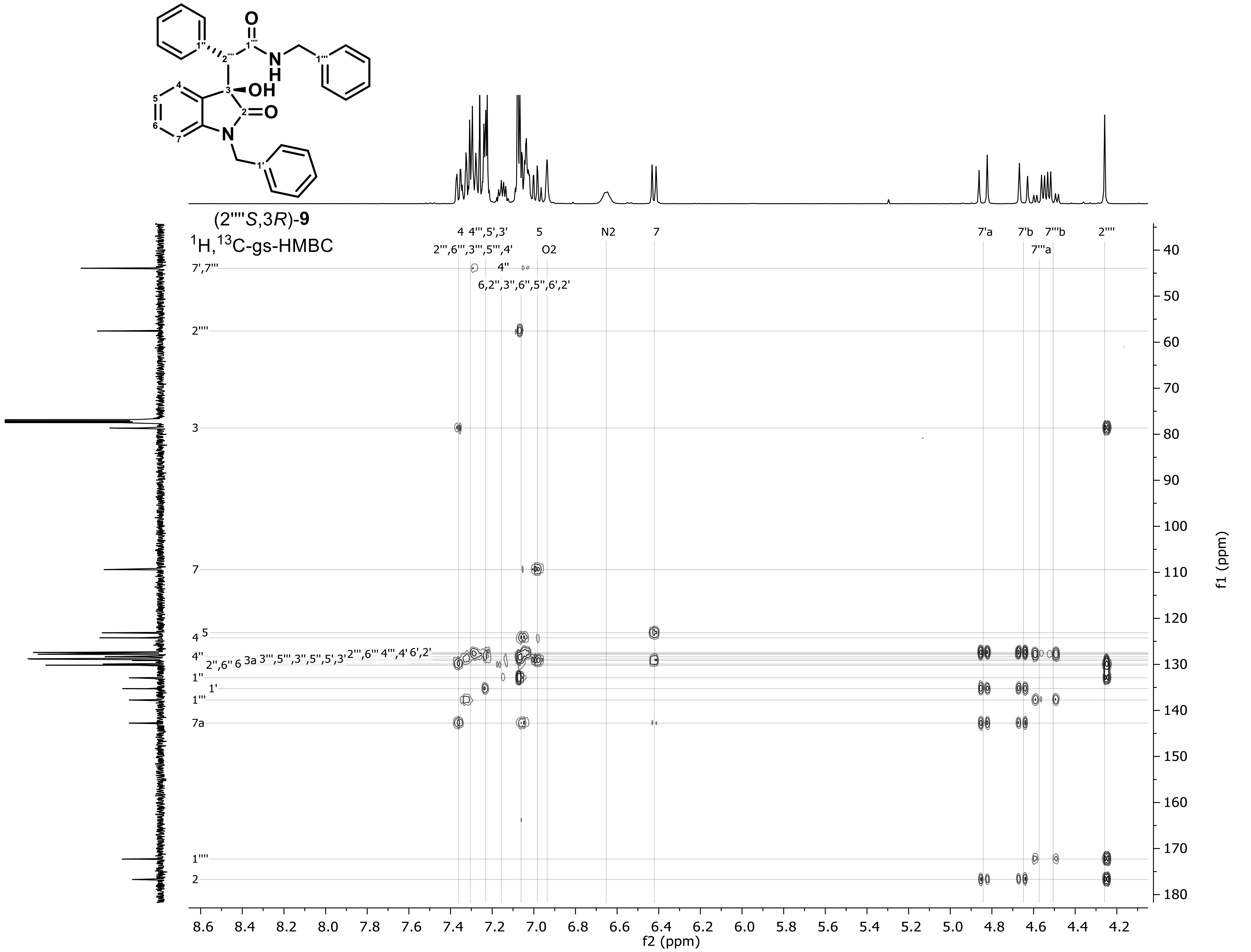
(2''''S,3R)-9
1H,13C-gs-HMBC
f2 (ppm)
f1 (ppm)

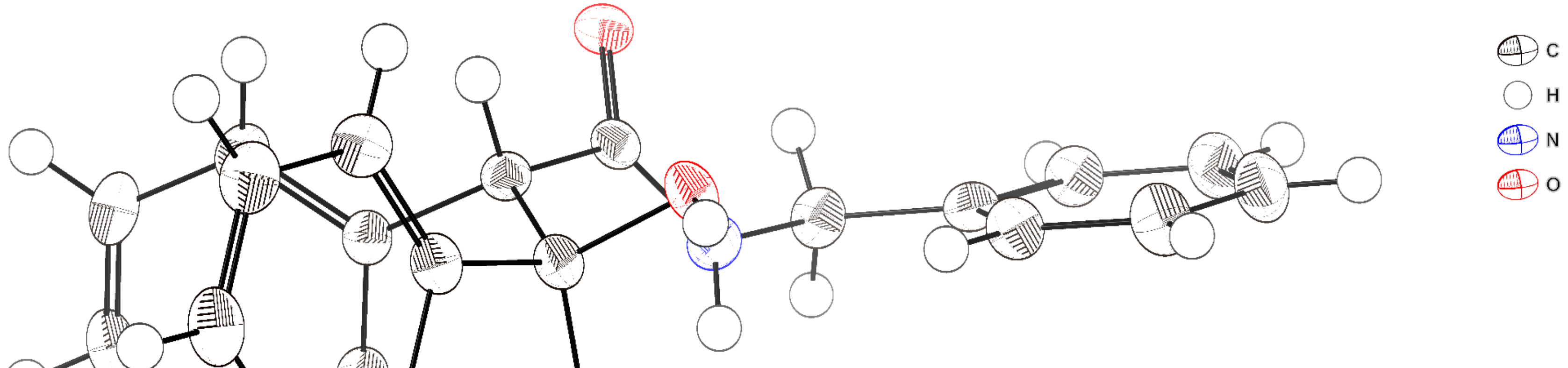

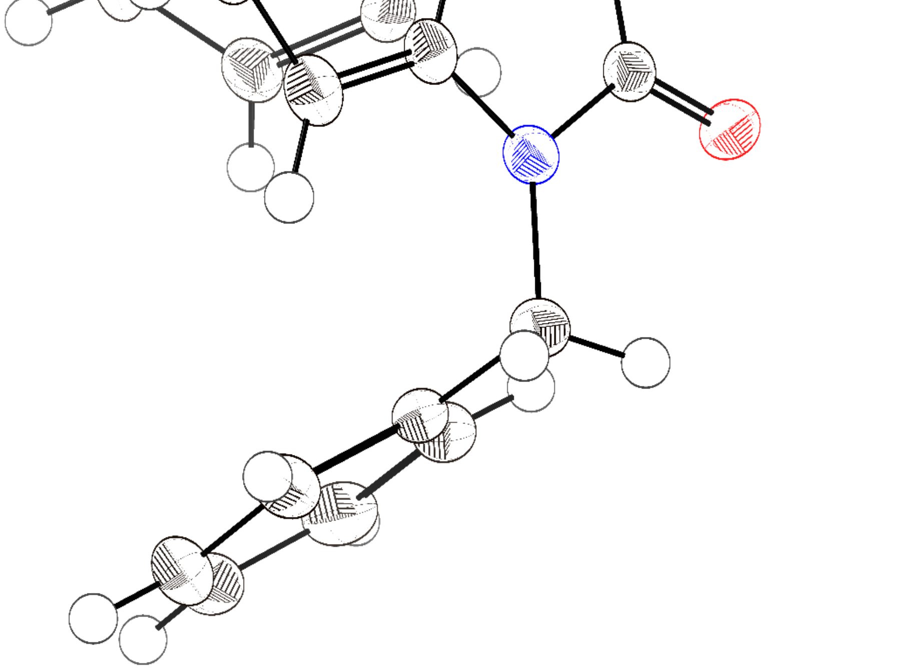

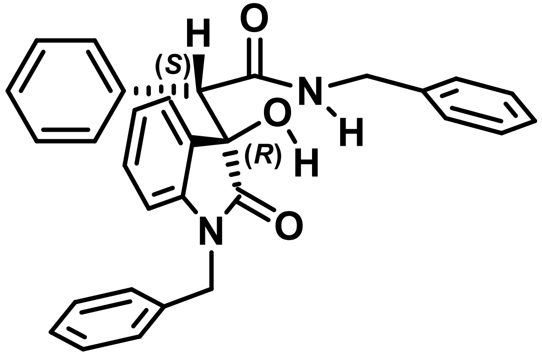

(2''''*S*,3*R*)-**9**

Single Crystal Refraction Analysis of (2''''*S*,3*R*)-**9** with ellipsoids shown at 40% probability

The crystal of compound **9** was prepared by diffusion of hexane into a dichloromethane solution. X-ray diffraction data for compound 9 were collected at 173 K using a Rigaku MM-007HF High Brilliance RA generator/confocal optics with XtaLAB P100 diffractometer [Cu Kα radiation (λ = 1.54187 Å)]. Intensity data were collected using both ω and ϕ steps accumulating area detector images spanning at least a hemisphere of reciprocal space. Data were collected using CrystalClear[10] and processed (including correction for Lorentz, polarization and absorption) using CrysAlisPro.[11] The structure was solved by charge-flipping methods (Superflip[12]) and refined by full-matrix least-squares against F2 (SHELXL-2018/3[13]). Non-hydrogen atoms were refined anisotropically, and alkyl and aryl hydrogen atoms were refined using a riding model. Hydrogen atoms bound to nitrogen and oxygen were located from the difference Fourier map and refined isotropically, subject to a distance restraint. All calculations were performed using the Olex2[14] interface. Deposition number 2153991contains the supplementary crystallographic data for this paper. These data are provided free of charge by the joint Cambridge Crystallographic Data Centre and Fachinformationszentrum Karlsruhe Access Structures service.
www.ccdc.cam.ac.uk/structures

Empirical formula	$C_{30}H_{26}N_2O_3$
Formula Weight [Da]	462.53904
Crystal Description	Orthorhombic
Space Group	$P2_12_12_1$
a [Å]	10.08426(12)
b [Å]	13.12584(14)
c [Å]	17.8763(2)
U [Å]3	2366.18(5)
T [K]	173
Z	4
Reflections Collected	25016
Unique Reflections	4314
R_{int}	0.0278
R_1 [$I > 2\sigma(I)$]	0.0265
wR_2 (all Data)	0.0677
Flack parameter	0.05(5)

b) ***N*-benzyl-2-(1-benzyl-7-chloro-3-hydroxy-2-oxoindolin-3-yl)-2-phenylacetamide (7)**

To a 25 mL round bottomed flask was added phenylacetic anhydride (95.3 mg, 0.375 mmol), *N*-benzylisatin (59.3 mg, 0.250 mmol), and (2*R*,3*S*)-HyperBTM (3.9 mg, 0.012 mmol). The mixture was cooled to 0 °C and CH_2Cl_2 (6.0 mL, 0.04 M) and Hünig's base (54.0 µL, 0.312 mmol) were added. The mixture was stirred at 0 °C for 3 h. Morpholine (65.0 µL, 0.750 mmol) was added and the reaction was stirred overnight at room temperature. 1,3,5-Trimethoxybenzene (0.1 M solution in CH_2Cl_2) added as internal standard and d.r recorded (82:18). The solvent was removed under reduced pressure and the reaction purified by column chromatography (CH_2Cl_2:Et_2O 85:15) to give a single fraction of major diastereomer and a mixed fraction of diastereomers as white solids which were combined for analysis (74 mg, 0.16 mmol, 65%)

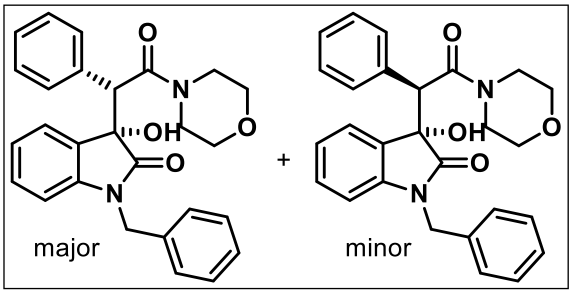

Major (2′′′′*S*,3*S*)-**7** and **minor** (2′′′′*R*,3*S*)-**7**: **m.p.** 207 °C dec; **Chiral HPLC analysis** (Chiralcel® AD-H 85:15 Hexane:IPA, flow rate 2.0 ml·min^{-1}, 211 nm, 40 °C) t_R (2*S*,3*S*)-**7** 14.1 min, t_R (2*R*,3*R*)-**7** 24.6 min, 99:1 e.r.; t_R (2*R*,3*S*)-**7** 12.3 min, t_R (2*S*,3*R*)-**7** 52.9 min – not detected; >95:5 e.r.; $\boldsymbol{\alpha_D^{20}} = -16.0$ (*c* 0.50, $CHCl_3$); $\boldsymbol{\nu_{max}}$ (thin film) 3330 (m, broad, NH, OH), 3061 (w), 3030 (w), 2963 (w), 2860 (w), 1722 (s, C=O lactam), 1713 (s, C=O, lactam), 1645 (m), 1614 (s, C=O, amide), 1497 (m), 1468 (m), 1454 (w), 1435 (w), 1373 (w), 1302 (w), 1256 (w), 1227 (m), 1177 (w), 1115 (w), 1078 (w), 1032 (w), 754 (m); **^{1}H NMR** (400 MHz, $CDCl_3$, 96:4 mixture of diastereomers, only diagnostic signals for minor diastereomer resolved) δ_H 7.37 (1.00H, d, $^3J_{HH}$ = 7.6 Hz, ArC^4*H*), 7.25 – 7.19 (3.84H, m, PhC$^{3',4',5'}$*H*, PhC$^{4''}$*H*), 7.19 – 7.14 (1.92H, m, PhC$^{3'',5''}$*H*), 7.14 – 7.10 (1.92H, m, PhC$^{2'',6''}$*H*), 7.07 (0.96H, app td, $^3J_{HH}$ = 7.8 Hz, 7.6 Hz, $^4J_{HH}$ = 1.3 Hz, ArC^6*H*), 7.03 – 6.96 (1.92H, m, PhC$^{2',6'}$*H*), 6.90 (0.96H, app td, $^3J_{HH}$ = 7.6 Hz, $^4J_{HH}$ = 1.1 Hz, ArC^5*H*), 6.59 (0.04H, d, $^3J_{HH}$ = 7.9 Hz, ArC^7*H*), 6.47 (0.96H, d, $^3J_{HH}$ = 7.8 Hz, ArC^7*H*), 5.99 (0.96H, s, O*H*), 4.99 (0.04H, d, $^2J_{HH}$ = 15.8 Hz, PhC$^{1'}$-CH_aH$_b$), 4.91 (0.96H, d, $^2J_{HH}$ = 15.9 Hz, PhC$^{1'}$-CH_aH$_b$), 4.66 (1.00H, d, $^2J_{HH}$ = 15.9 Hz, PhC$^{1'}$-CH$_a$$H_b$), 4.40 (0.04H, s, PhC$^{1''}$-C*H*), 4.33 (0.96H, s, PhC$^{1''}$-C*H*), 3.83 – 3.62 (2.88H, m, OCH_cH$_d$, NCH$_a$H$_b$), 3.56 (0.96H, ddd, $^2J_{HH}$ = 10.5 Hz, $^3J_{HH}$ = 6.4 Hz, 3.0 Hz, OCH$_c$$H_d$), 3.46 (0.96H, ddd, $^2J_{HH}$ = 11.4 Hz, $^3J_{HH}$ = 6.1 Hz, 3.0 Hz, OCH_aH$_b$), 3.36 (0.96H, ddd, $^2J_{HH}$ = 13.4 Hz, $^3J_{HH}$ = 7.1 Hz, 3.0 Hz, NCH_cH$_d$), 3.19 (0.96H, ddd, $^2J_{HH}$ = 13.4 Hz, $^3J_{HH}$ = 6.1 Hz, 3.0 Hz, NCH$_c$$H_d$), 3.04 (0.96H, ddd, $^2J_{HH}$ = 11.4 Hz, $^3J_{HH}$ = 7.0 Hz, 3.0 Hz, OCH$_a$$H_b$); **^{13}C{^{1}H} NMR** (101 MHz, $CDCl_3$, 96:4 mixture of diastereomers, minor diastereomer not resolved) δ_C 176.1 (*C*(O)NBn), 171.0 (*C*(O)N<(CH_2)$_4$>O), 142.8 (Ar*C*^{7a}), 135.4 (Ph*C*$^{1'}$), 133.1 (Ph*C*$^{1''}$), 130.0 (Ph*C*$^{2'',6''}$H), 129.6 (Ar*C*^{6}H), 128.8 and 128.7 (Ph*C*$^{3',5'}$H and Ph*C*$^{3'',5''}$H), 128.6 (Ar*C*^{3a}), 128.1 (Ph*C*$^{4'}$H), 127.6 (Ph*C*$^{4''}$H), 127.4 (Ar*C*^{4}H), 127.2 (Ph*C*$^{2',6'}$H), 122.8 (Ar*C*^{5}H), 109.2 (Ar*C*^{7}H), 79.0 (*C*-OH), 66.8 (O*C*H$_c$H$_d$), 66.1 (O*C*H$_a$H$_b$), 53.8 (PhC$^{1''}$-*C*H), 46.6 (N*C*H$_c$H$_d$), 43.9 (PhC$^{1'}$-*C*H$_2$), 42.6 (N*C*H$_a$H$_b$); ***m/z*** **(ESI$^+$):** 91 ([C_7H_7]$^+$ 47%), 123 (13%), 161 (18%), 229 (56%), 260 ([M-$C_{12}H_{15}NO_2$+Na]$^+$ 42%), 465 ([M+Na]$^+$ 100%), 466 ([M(^{13}C)+Na]$^+$ 30%), 467 ([M($^{13}C_2$)+Na]$^+$ 5%), 481 ([M+K]$^+$ 5%), 907 ([2M+Na]$^+$ 13%); **HRMS** (ESI$^+$) *m/z* calcd for [M+Na]$^+$ $C_{27}H_{26}O_4N_2Na$ 465.1785, found 465.1782 (−0.5 ppm).

(±)-anti-**7** and (±)-syn-**7**

PDA Ch1 211nm

Peak#	Ret. Time	Area%
1	12.386	5.466
2	14.076	44.854
3	24.605	44.470
4	53.282	5.210
Total		100.000

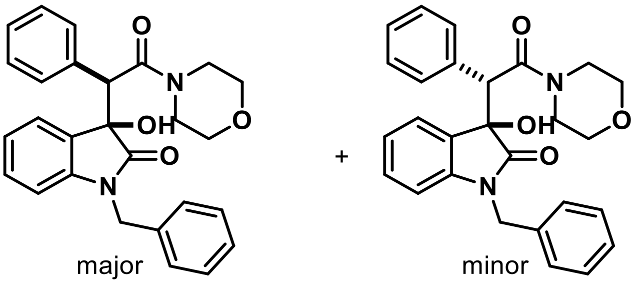

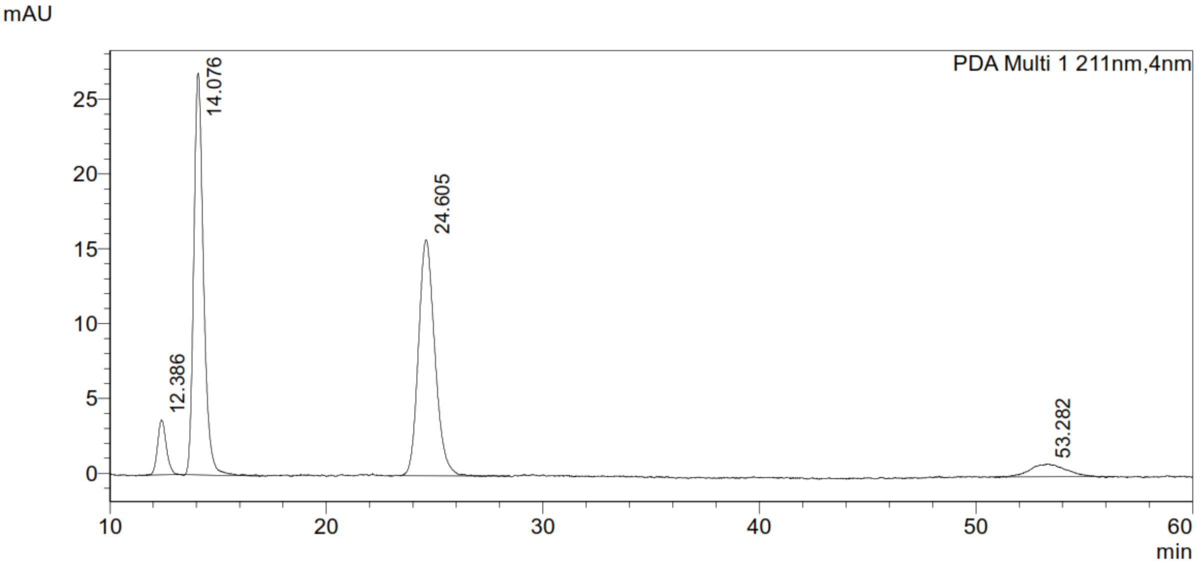

(2''''*S*,3*S*)-**7** and (2''''*R*,3*S*)-**7**

PDA Ch1 211nm

Peak#	Ret. Time	Area%
1	12.341	1.154
2	14.083	97.358
3	24.809	1.423
4	52.875	0.066
Total		100.000

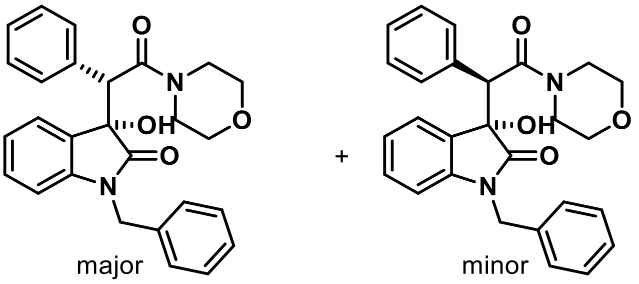

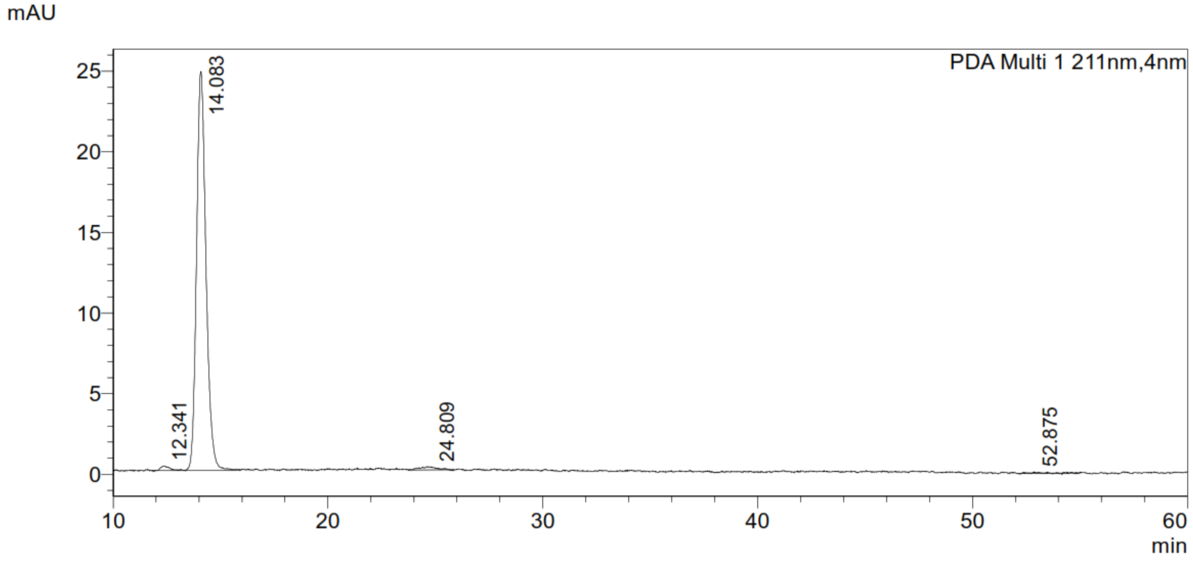

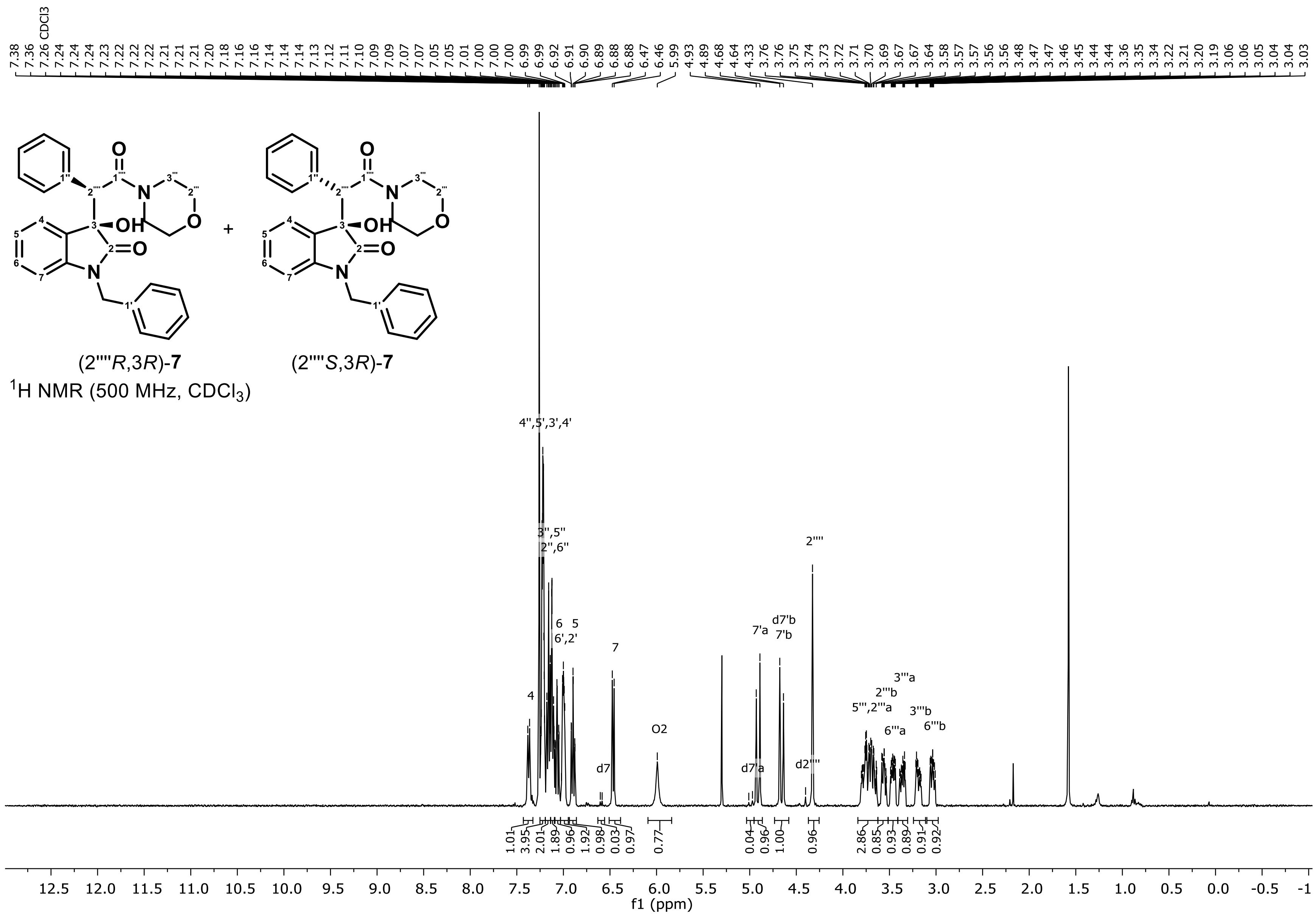
(2''''R,3R)-7
(2''''S,3R)-7
1H NMR (500 MHz, CDCl3)
4'',5',3',4'
3'',5''
2'',6''
6 5
6',2'
7
4
d7
O2
7'a
d7'b
7'b
d7'a
2''''
d2''''
3'''a
2'''b
5''',2'''a
6'''a
3'''b
6'''b
f1 (ppm)

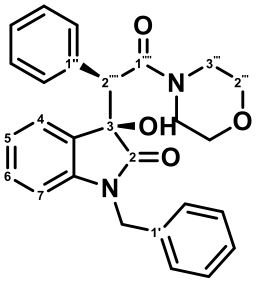

(2''''R,3R)-**7**
^{13}C {^{1}H} NMR (101 MHz, $CDCl_3$)

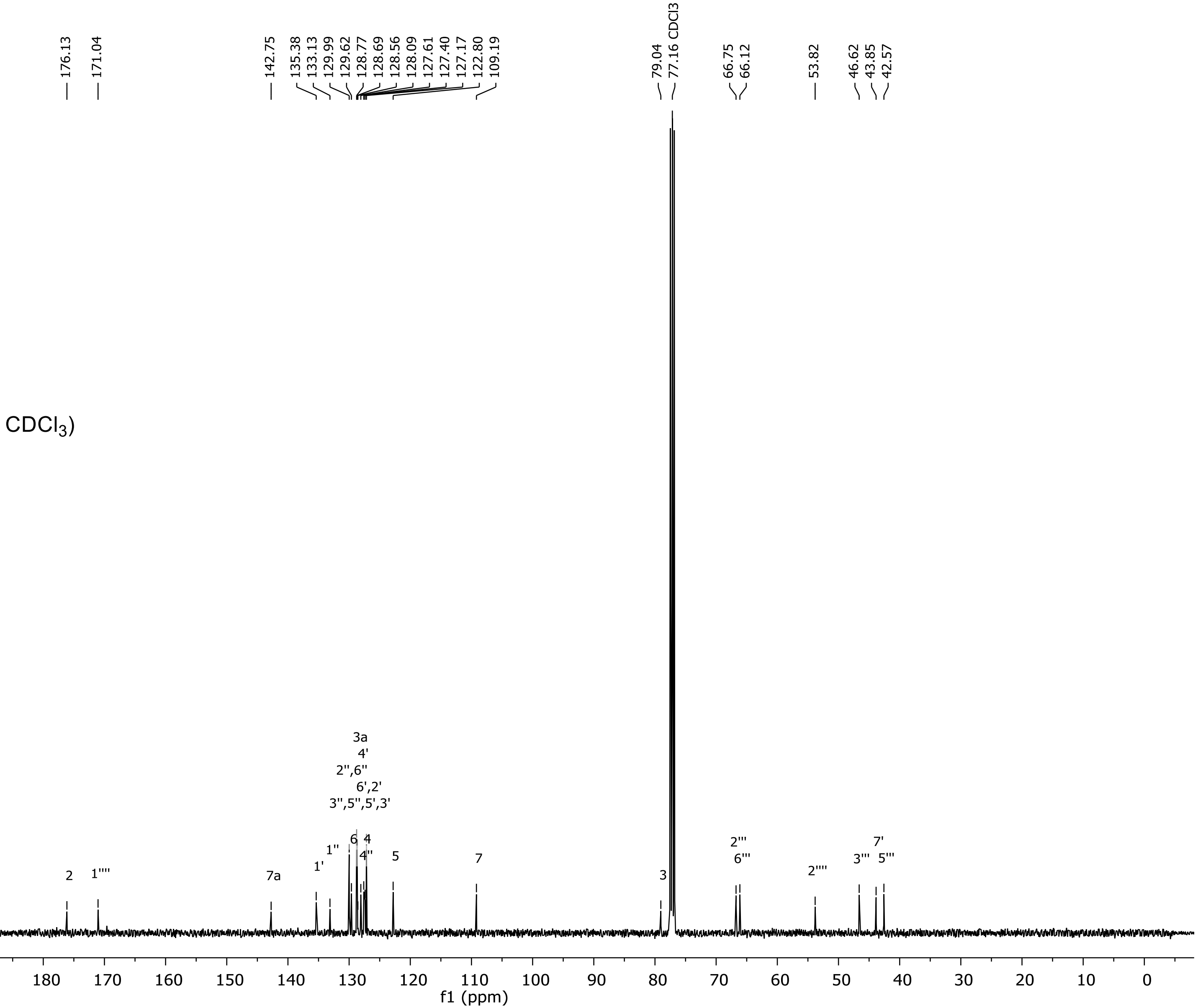

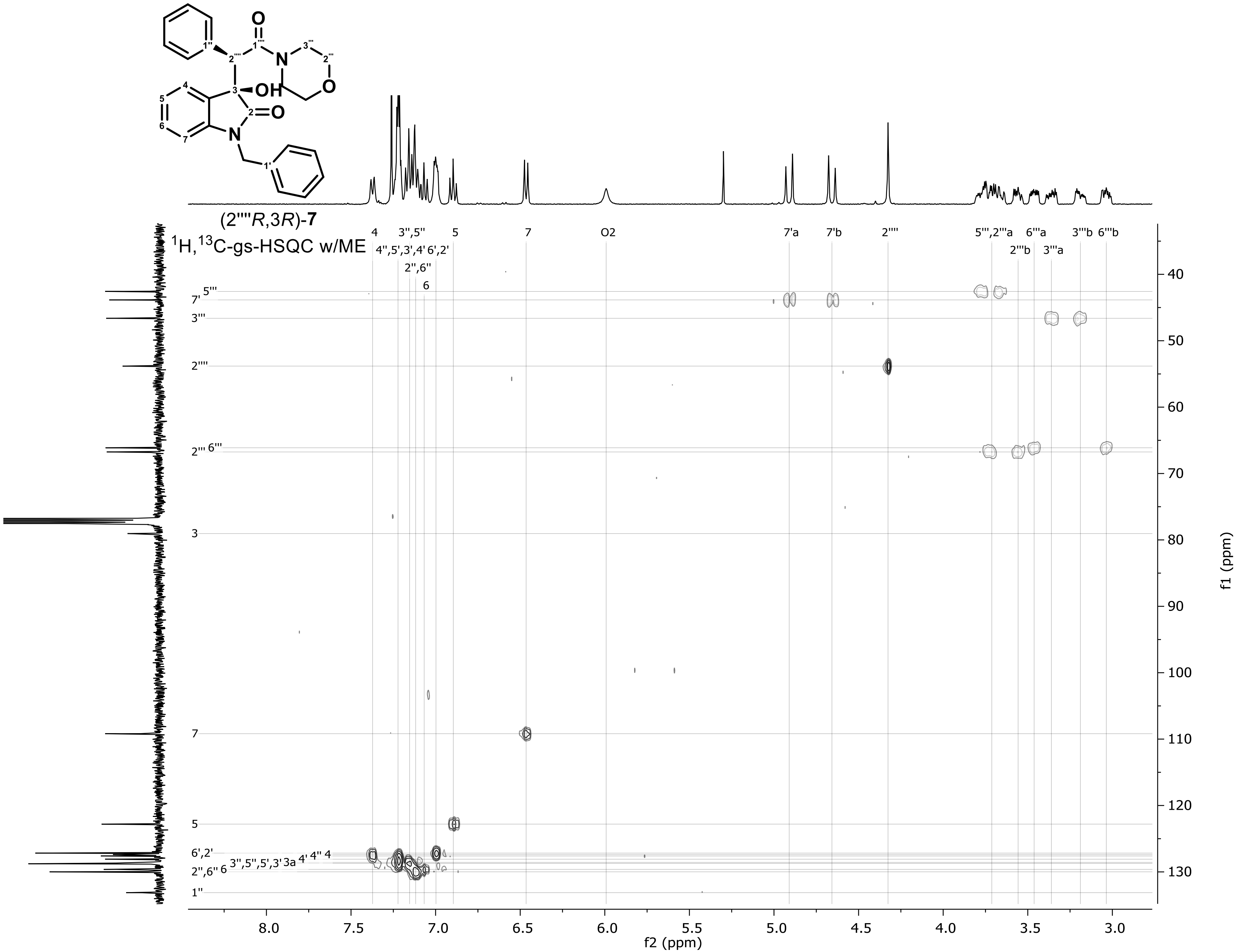

(2''''R,3R)-7
1H,13C-gs-HSQC w/ME
f2 (ppm)
f1 (ppm)

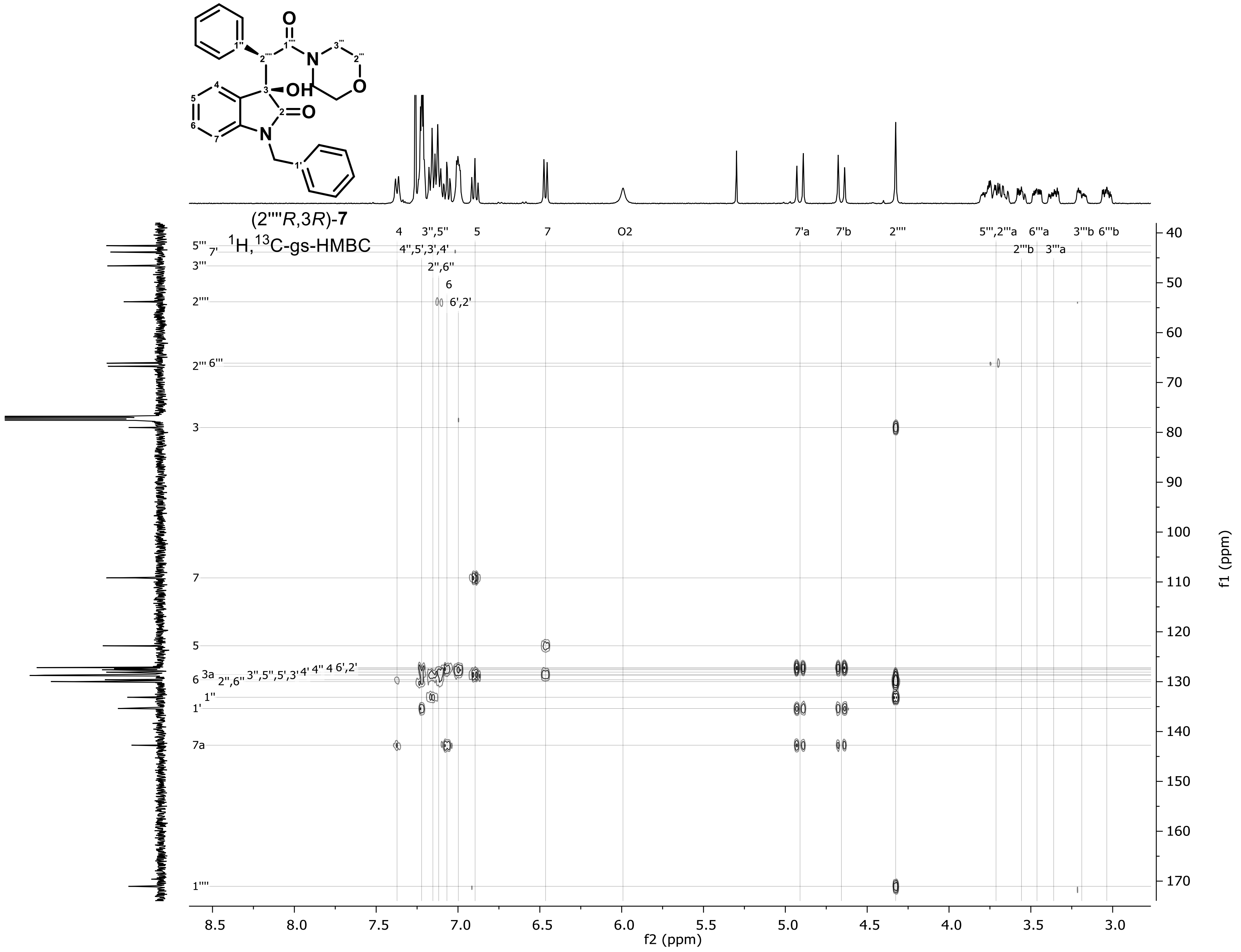
(2''''R,3R)-7
1H,13C-gs-HMBC
4
3'',5''
5
7
O2
7'a
7'b
2''''
5''',2'''a
6'''a
3'''b
6'''b
4'',5',3',4'
2'''b
3'''a
2'',6''
6
6',2'
5'''
7'
3'''
2''''
2''' 6'''
3
7
5
6
3a
2'',6''
3'',5'',5',3'
4'
4''
4
6',2'
1''
1'
7a
1''''
f1 (ppm)
f2 (ppm)
8.5
8.0
7.5
7.0
6.5
6.0
5.5
5.0
4.5
4.0
3.5
3.0
40
50
60
70
80
90
100
110
120
130
140
150
160
170

c) 1-Benzyl-4-chloro-3-hydroxy-3-(2-pyrrolidino-2-oxo-1-phenylethyl)indolin-2-one (8)

To a 25 ml round bottomed flask was added phenylacetic anhydride (95.3 mg, 0.375 mmol), *N*-benzylisatin (59.3 mg, 0.250 mmol), and (2*S*,3*R*)-HyperBTM (3.9 mg, 0.012 mmol). The mixture was cooled to 0 °C and CH_2Cl_2 (6.0 ml, 0.04 M) and Hünig's base (54 µl, 0.312 mmol) were added. The mixture was stirred at 0 °C for 3 h. Pyrrolidine (63 µl, 0.750 mmol) was added and the reaction was left to be stirred overnight at room temperature. The solvent was removed under reduced pressure. Purification by column chromatography (CH_2Cl_2:Acetone 100% → 4:1) gave the title compound as a mixture of diastereomers as brown semi-solid (88.1 mg, 0.207 mmol, 83%).

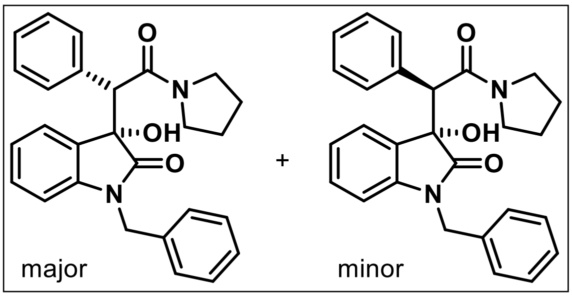

Major (2*S*,3*S*)-**8** and **minor** (2*R*,3*S*)-**8** (analysed as 72:28 mixture of diastereomers): $\mathbf{R_f}$ 0.46 (CH_2Cl_2:EtOAc 7:3); **Chiral HPLC analysis** (Chiralcel® AD-H 85:15 Hexane:IPA, flow rate 2.0 ml·min^{-1}, 211 nm, 40 °C) t_R (2*S*,3*S*)-**8** 9.7 min, t_R (2*R*,3*R*)-**8** 18.1 min, >99:1 e.r.; t_R (2*R*,3*S*)-**8** 11.4 min, t_R (2*S*,3*R*)-**8** 15.6 min; >99:1 e.r.; $\alpha_D^{20} = -0.408$ (*c* 1.0, $CHCl_3$); $\boldsymbol{\nu_{max}}$ (film) 3379 (w, OH), 3061 (w, CH), 3030 (w, CH), 2971 (w, CH), 2953 (w, CH), 2928 (w, CH), 2876 (w, CH), 1717 (s, C=O lactam), 1612 (s, C=O amide), 1489 (m), 1468 (s), 1454 (s), 1439 (s), 1368 (m), 1298 (w), 1287 (w), 1227 (w), 1175 (m), 1117 (w), 1078 (m), 1034 (w), 1013 (w), 972 (w), 912 (m), 866 (w), 754 (m); **^{1}H NMR** (400 MHz, $CDCl_3$) δ_H 7.38 – 7.11 (8.84H, m, ArC^6*H* (minor), PhC$^{3',4',5'}$H (major), PhC$^{2',3',4',5',6'}$*H* (minor), PhC$^{2'',3''4'',5'',6''}$*H* (major and minor)), 7.10 – 6.97 (3.16H, m, ArC^{4,6}*H* and PhC$^{2',6'}$*H* (major), ArC^4*H* (minor)), 6.93 (0.28H, app td, $^3J_{HH}$ = 7.5 Hz, $^4J_{HH}$ = 1.0 Hz, ArC^5*H*), 6.84 (0.72H, app td, $^3J_{HH}$ = 7.6 Hz, $^4J_{HH}$ = 1.0 Hz, ArC^5*H*), 6.67 (0.72H, s, O*H*), 6.60 (0.28H, dd, $^3J_{HH}$ = 7.8 Hz, $^4J_{HH}$ = 1.0 Hz, ArC^7*H*), 6.50 (0.72H, dd, $^3J_{HH}$ = 8.2 Hz, $^4J_{HH}$ = 1.0 Hz, ArC^7*H*), 5.54 (0.28H, s, O*H*), 4.95 (0.28H, d, $^2J_{HH}$ = 15.8 Hz, PhC1′-C*H*$_a$H$_b$N), 4.93 (0.72H, d, $^2J_{HH}$ = 15.8 Hz, PhC$^{1'}$-C*H*$_a$H$_b$N), 4.66 (0.72H, d, $^2J_{HH}$ = 15.8 Hz, PhC$^{1'}$-CH$_a$*H*$_b$N), 4.65 (0.28H, d, $^2J_{HH}$ = 15.8 Hz, PhC$^{1'}$-CH$_a$*H*$_b$N), 4.10 (0.28H, s, PhC$^{1''}$-C*H*), 4.04 (0.72H, s, PhC$^{1''}$-C*H*), 3.69 (0.72H, app dt, $^2J_{HH}$ = 12.8 Hz, $^3J_{HH}$ = 6.5 Hz, NC*H*$_c$H$_d$CH$_2$), 3.55 (0.28H, app dt, $^2J_{HH}$ = 12.6 Hz, $^3J_{HH}$ = 6.4 Hz, NC*H*$_c$H$_d$CH$_2$), 3.51 – 3.41 (1H, m, NC*H*$_c$H$_d$CH$_2$), 3.38 (0.72H, app dt, $^2J_{HH}$ = 10.0 Hz, $^3J_{HH}$ = 6.1 Hz, NC*H*$_a$H$_b$CH$_2$), 3.26 (0.28H, app dt, $^2J_{HH}$ = 10.4 Hz, $^3J_{HH}$ = 6.2 Hz, NC*H*$_a$H$_b$CH$_2$), 2.95 (0.72H, app dt, $^2J_{HH}$ = 10.0 Hz, $^3J_{HH}$ = 6.5 Hz, NCH$_a$*H*$_b$CH$_2$), 2.86 (0.28H, app dt, $^2J_{HH}$ = 10.4 Hz, $^3J_{HH}$ = 6.3 Hz, NCH$_a$*H*$_b$CH$_2$), 1.96 – 1.62 (4H, m, N(CH$_2$C*H*$_2$)$_2$); **^{13}C {^{1}H} NMR** (101 MHz, $CDCl_3$) δ_C 176.6 (C(O)NBn (major), 175.4 (*C*(O)NBn (minor)), 171.0 (*C*(O)N(CH$_2$)$_4$ (major)), 170.0 (*C*(O)N(CH$_2$)$_4$ (minor)), 143.0 (Ar*C*^{7a} (minor)), 142.7 (Ar*C*^{7a} (major)), 135.8 (Ph*C*$^{1''}$ (minor)), 135.5 (PhC$^{1'}$ (major)), 133.1 (Ph*C*$^{1''}$ (minor)), 133.0 (Ph*C*$^{1''}$ (major)), 130.5 (Ar*C*^{3a} (minor)), 130.4 (Ph*C*$^{2'',6''}$H (major)), 130.3 (Ph*C*$^{2'',6''}$H (minor)), 129.6 (Ar*C*^{6}H (minor)), 129.5 (Ar*C*^{6}H (major)), 128.8 and 128.4 (Ph*C*$^{3',5'}$H and Ph*C*$^{3'',5''}$H (major)), 128.5 (Ar*C*^{3a} (major)), 128.1 (Ph*C*H), 128.0 (Ph*C*H), 127.6 (Ph*C*H), 127.5 (Ph*C*H), 127.3 (Ph*C*$^{2',6'}$H), 127.2 (Ph*C*$^{2',6'}$H (major)), 124.4 (Ar*C*^{4}H (minor)), 122.7 (Ar*C*^{5}H (major)), 122.6 (Ar*C*^{5}H (minor)), 109.4 (Ar*C*^{7}H (minor)), 109.1 (Ar*C*^{7}H (major)), 78.9 (*C*-OH (major)), 77.7 (*C*-OH (minor)), 56.4 (PhC$^{1''}$-*C*H (minor)), 54.7 (PhC$^{1''}$-*C*H (major)), 46.9 (N*C*H$_a$H$_b$ (major)), 46.7 (N*C*H$_a$H$_b$ (minor)), 46.3 (N*C*H$_c$H$_d$ (major)), 46.2 (N*C*H$_c$H$_d$ (minor)), 43.9 (PhC$^{1'}$-*C*H$_2$N (minor)), 43.8 (PhC$^{1'}$-*C*H$_2$N (major)), 26.1 (N(CH$_c$H$_d$*C*H$_2$)$_2$ (major)), 26.0 (N(CH$_c$H$_d$*C*H$_2$)$_2$ (minor)), 24.2 (N(CH$_a$H$_b$*C*H$_2$)$_2$ (major and minor)); ***m/z*** (ESI$^+$) 260 (21%), 427 ([M+H]$^+$ 8%), 437 (3%), 449 ([M+Na]$^+$ 100%), 450 ([M(^{13}C)+Na]$^+$ 27%), 451 ([M(^{13}C$_2$)+Na]$^+$ 4%), 875 ([2M+Na]$^+$ 33%); **HRMS** (ESI$^+$) *m/z* calcd for [M+Na]$^+$ $C_{27}H_{26}N_2O_3Na$ 449.1836, found 449.1825 (−2.5 ppm).

(±)-anti-**8** and (±)-syn-**8**

PDA Ch1 211nm

Peak#	Ret. Time	Area%
1	9.732	35.941
2	11.427	14.806
3	15.601	14.333
4	18.105	34.920
Total		100.000

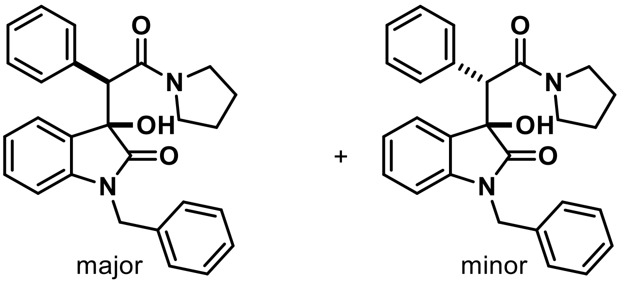

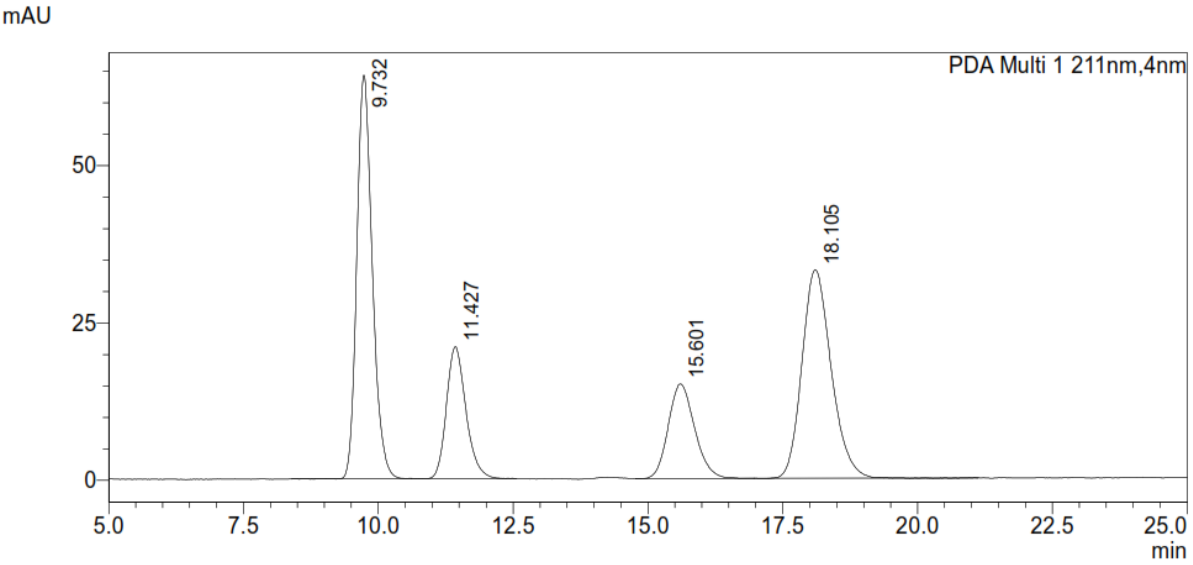

(2''''*S*,3*S*)-**8** and (2''''*R*,3*S*)-**8**

PDA Ch1 211nm

Peak#	Ret. Time	Area%
1	9.677	69.718
2	11.359	29.937
3	15.570	0.083
4	18.015	0.262
Total		100.000

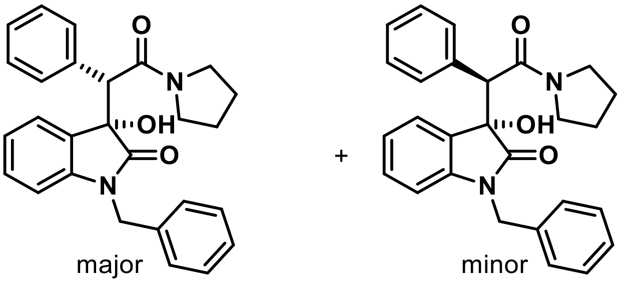

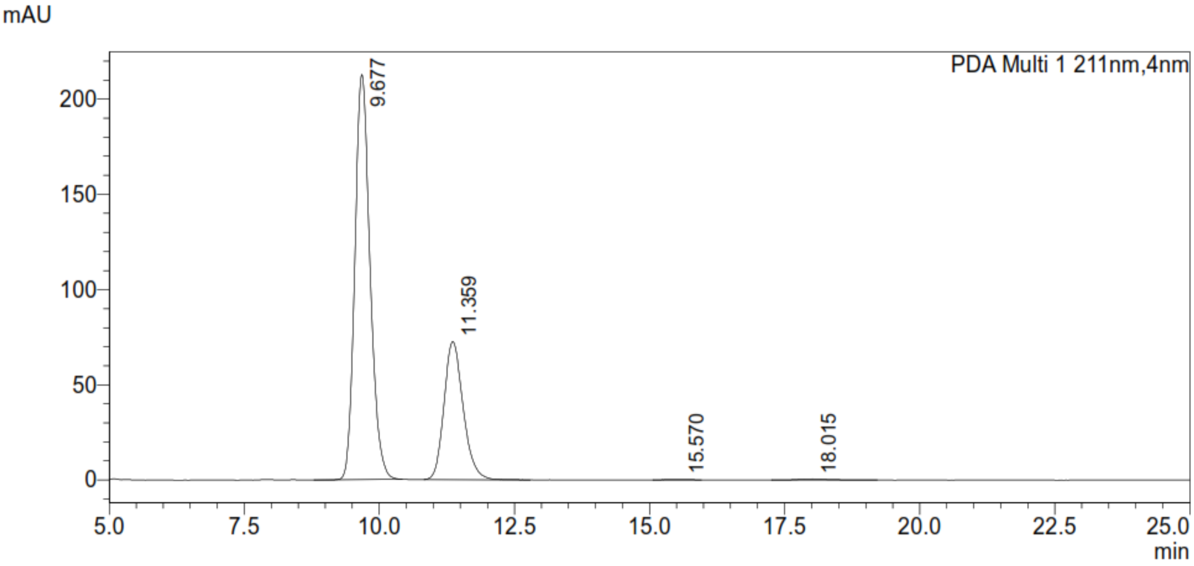

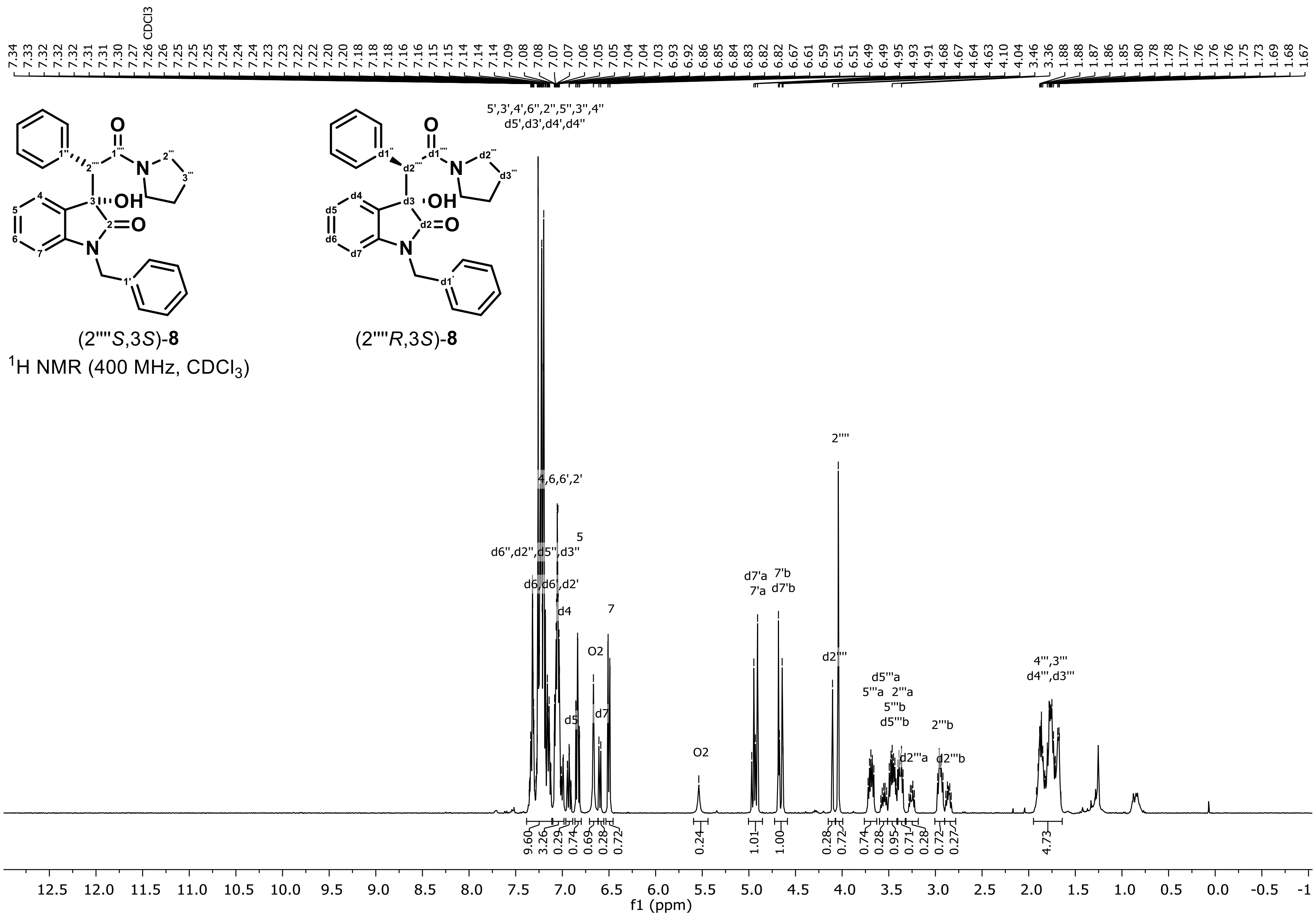

(2''''*S*,3*S*)-**8**

(2''''*R*,3*S*)-**8**

^{1}H NMR (400 MHz, $CDCl_3$)

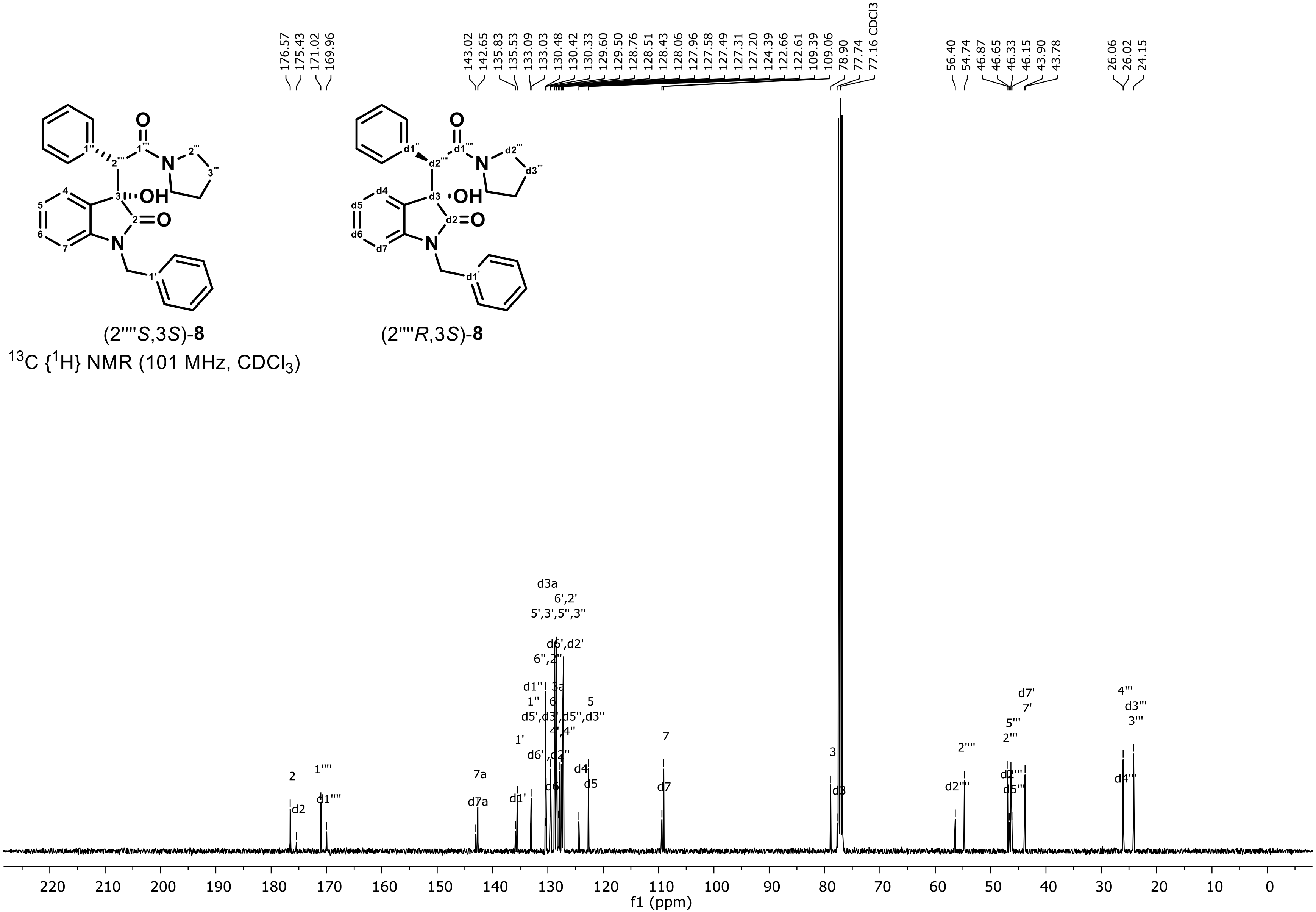

(2''''S,3S)-**8**

(2''''R,3S)-**8**

$^{13}C\ \{^{1}H\}$ NMR (101 MHz, $CDCl_3$)

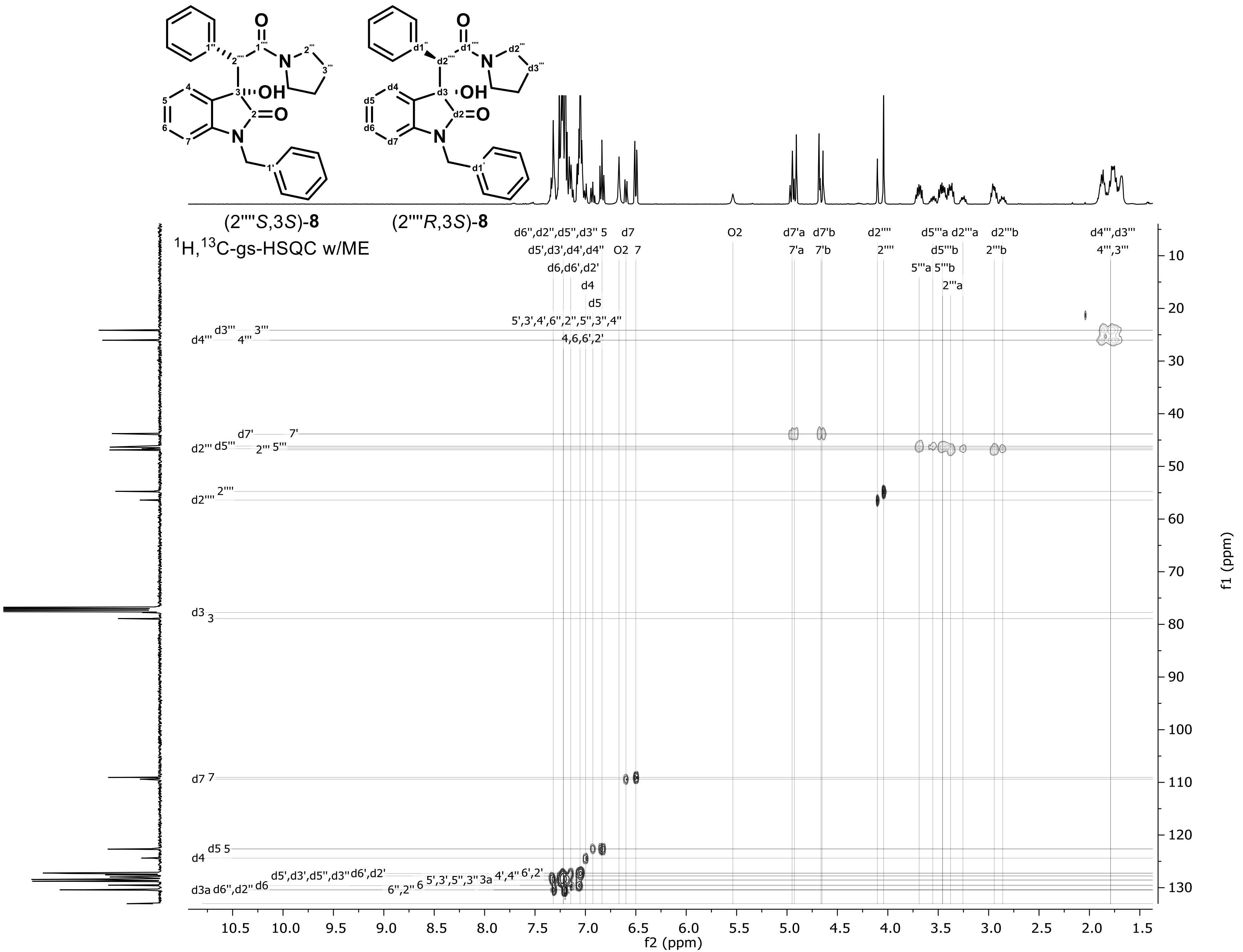
(2''''S,3S)-8
(2''''R,3S)-8
1H,13C-gs-HSQC w/ME
f2 (ppm)
f1 (ppm)

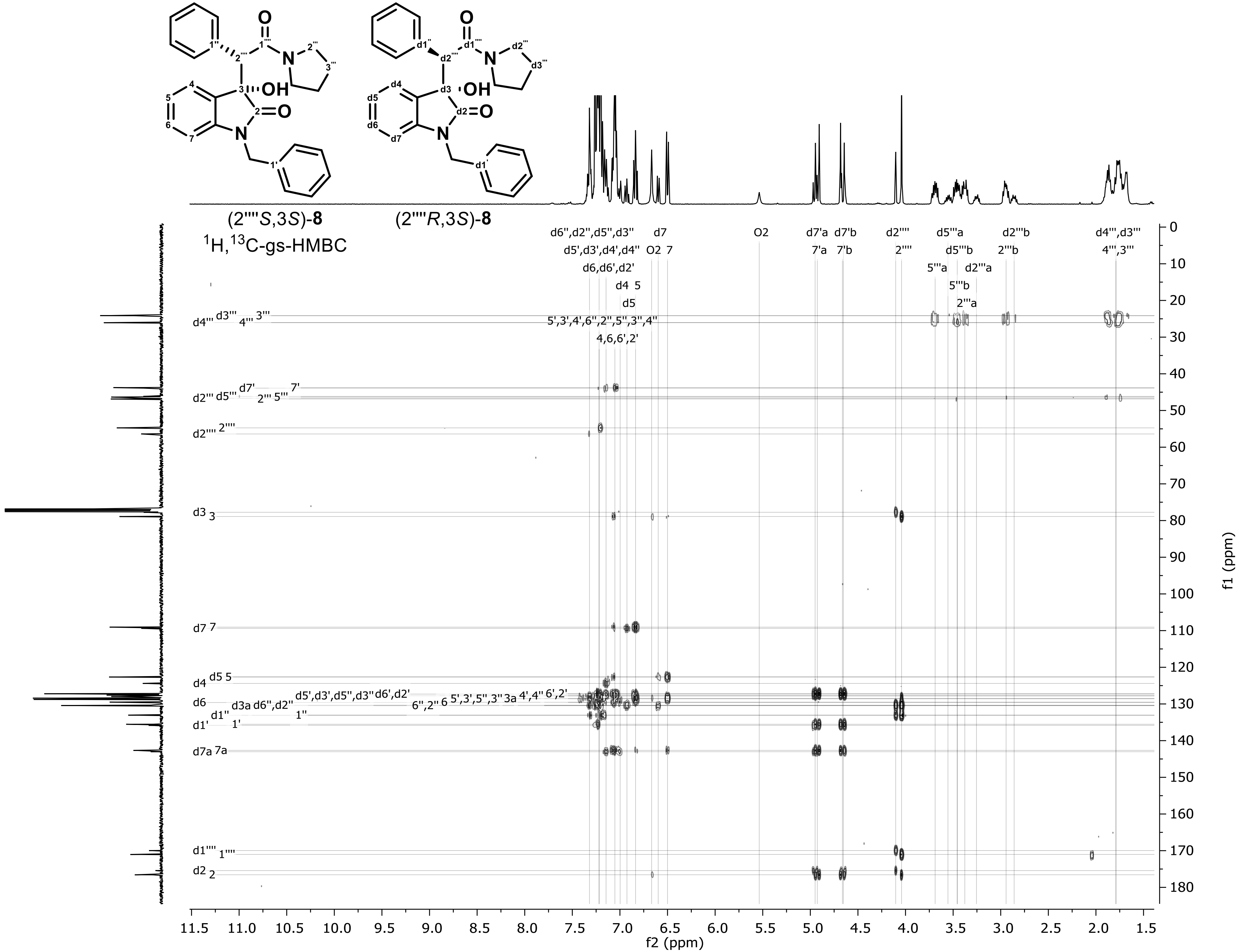
(2''''S,3S)-8
(2''''R,3S)-8
^{1}H,^{13}C-gs-HMBC
f2 (ppm)
f1 (ppm)

d) *N'*-Benzyl,*N*-methyl 2-(3-hydroxy-2-oxoindolin-3-yl)-2-phenylacetamide (10)

To a 25 ml round bottomed flask was added phenylacetic anhydride (95.3 mg, 0.375 mmol), *N*-methylisatin (46.8 mg, 0.250 mmol), and (2*R*,3*S*)-HyperBTM (3.9 mg, 0.012 mmol). The mixture was cooled to 0 °C and CH_2Cl_2 (6.0 ml, 0.04 M) and Hünig's base (54 µl, 0.312 mmol) were added. The mixture was stirred at 0 °C for 3 h. Benzylamine (82 µl, 0.750 mmol) was added and them reaction was left to be stirred overnight at room temperature. 1,3,5-Trimethoxybenzene (0.1 M soln in CH_2Cl_2 500 µl, 0.05 mmol) and the solvent was removed. Purification by column chromatography (Pentanes : EtOAc 4:1 → 7:3) to give the title compound in two fractions (major diastereomer (48 mg, 50%) and minor diastereomer as white solids (10 mg, 10%); combined (58 mg, 0.150 mmol, 60%, 78:22 d.r.).

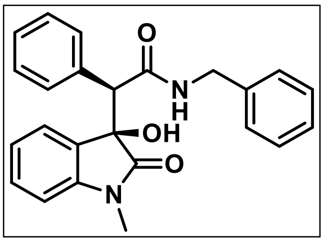

Major (2'''*R*,3*R*)-**10**: **Chiral HPLC analysis** Chiralpak AD-H (92:8 hexane:IPA, flow rate 1.5 ml·min^{-1}, 211 nm, 40 °C) t_R (2'''*S*,3*S*)-**10**: 20.4 min, t_R (2'''*R*,3*R*)-**10**: 23.9 min, <1:99; $\boldsymbol{\alpha_D^{20}} = +\ 59.5$ (*c* 2.74, $CHCl_3$); $\boldsymbol{\nu_{max}}$ (thin film) 3319 (m(br), OH, NH), 3059 (w), 1721 (s), 1647 (m), 1614 (s), 1541 (m), 1495 (m), 1472 (m), 1454 (m), 1423 (w), 1373 (m), 1348 (m), 1260 (w), 1227 (w), 1107 (m), 1092 (m), 1022 (w), 980 (w), 752 (s); **^{1}H NMR** (400 MHz, $CDCl_3$) δ_H: 7.35 – 7.23 (5H, m, ArC^{4,6}*H*, CH-PhC$^{3',5'}$*H*, NHCH$_2$-PhC$^{3'',5''}$*H*), 7.22 – 7.16 (3H, m, NHCH$_2$-PhC$^{2'',4'',6''}$*H*), 7.14 – 7.07 (2H, m, CH-PhC$^{3',5'}$*H*), 6.97 (1H, app t, $^3J_{HH}$ = 7.5 Hz, ArC^5*H*), 6.66 (1H, s, O*H*), 6.89 – 6.94 (2H, m, CH-PhC$^{2',6'}$*H*), 6.16 (1H, s(br), N*H*), 6.57 (1H, d, $^3J_{HH}$ = 7.8 Hz, ArC^7*H*), 4.49 (1H, dd, $^2J_{HH}$ = 15.1 Hz, $^3J_{HH}$ = 6.1 Hz, NHCH_aH$_b$-Ph), 4.45 (1H, dd, $^2J_{HH}$ = 15.1 Hz, $^3J_{HH}$ = 5.8 Hz, NHCH$_a$$H_b$-Ph), 4.08 (1H, s, C*H*-Ph), 2.79 (3H, s, NCH_3); **^{13}C {^{1}H} NMR** (101 MHz, $CDCl_3$) δ_C: 175.2 (*C*(O)NMe), 173.0 (*C*(O)NHBn), 143.8 (Ar*C*^{7a}), 137.6 (NHCH$_2$-Ph*C*$^{1''}$), 132.9 (CH-Ph*C*$^{1'}$), 130.2 (CH-Ph*C*$^{2',6'}$H), 129.9 (Ar*C*^{6}H), 128.8 (NHCH$_2$-Ph*C*$^{3'',5''}$H), 128.5 (Ar*C*^{3a}H), 128.4 (NHCH$_2$-Ph*C*$^{4''}$H), 128.3 (CH-Ph*C*$^{3',5'}$H), 127.6$_8$ (NHCH$_2$-Ph*C*$^{2',6'}$H), 127.6$_5$ (CH-Ph*C*$^{4'}$H), 126.0 (Ar*C*^{4}H), 122.9 (Ar*C*^{5}H), 108.1 (Ar*C*^{7}H), 79.1 (*C*-OH), 56.9 (*C*H-Ph), 43.7 (NH*C*H$_2$-Ph), 25.8 (N*C*H$_3$); **HRMS** (ESI$^+$) *m*/*z* calcd for $[M]^+$ $C_{24}H_{23}N_2O_3$ 387.1703, found 387.1696 (−1.8 ppm).

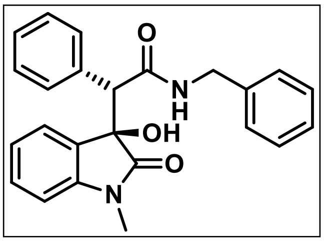

Minor (2'''*S*,3*R*)-**10**: **Chiral HPLC analysis** Chiralpak IB (95:5 hexane:IPA, flow rate 2 ml·min^{-1}, 211 nm, 40 °C) t_R (2'''*S*,3*R*)-**10**: 23.1 min, t_R (2'''*R*,3*S*)-**10**: 31.5 min, >99:1; $\boldsymbol{\alpha_D^{20}} = -18.0$ (*c* 0.44, $CHCl_3$); $\boldsymbol{\nu_{max}}$ (thin film) 3316 (m (br), OH, NH), 3086 (w), 3061 (w), 3030 (w), 3011 (w), 2932 (w), 1721 (s), 1709 (s), 1645 (m), 1614 (s), 1547 (m), 1530 (m), 1495 (m), 1472 (m), 1454 (m), 1423 (w), 1373 (m), 1348 (m), 1302 (w), 1258 (w), 1227 (m), 1157 (w), 1134 (w), 1107 (m), 1094 (m), 1024 (w), 980 (w), 752 (s); **^{1}H NMR** (400 MHz, $CDCl_3$) δ_H 7.35 – 7.31 (3H, m, ArC4H, PhC$^{3'',5''}$*H*), 7.31 – 7.25 (3H, m, PhC$^{2'',4'',6''}$*H*), 7.17 (1H, ddd, $^3J_{HH}$ = 7,8 Hz, 7,7 Hz, $^4J_{HH}$ = 1.3 Hz, ArC^6*H*), 7.18 – 7.10 (3H, m, PhC$^{3',4',5'}$*H*), 7.10 – 7.05 (2H, m, PhC$^{2',6'}$*H*), 7.02 (1H, ddd, $^3J_{HH}$ = 7.7 Hz, 7.5 Hz, $^4J_{HH}$ = 1.0 Hz, ArC^5*H*), 6.99 (1H, s(br), O*H*), 6.48 (1H, s(br), N*H*), 6.52 (1H, d, $^3J_{HH}$ = 7.8 Hz, ArC^7*H*), 4.57 (1H, dd, $^2J_{HH}$ = 15.0 Hz, $^3J_{HH}$ = 5.8 Hz, NHCH_aH$_b$-Ph), 4.52 (1H, dd, $^2J_{HH}$ = 15.0 Hz, $^3J_{HH}$ = 5.8 Hz, NHCH$_a$$H_b$-Ph), 4.18 (1H, s, C*H*-Ph), 3.03 (3H, s, NCH_3); **^{13}C{^{1}H} NMR** (101 MHz, $CDCl_3$) δ_C 176.6 (*C*(O)NMe), 172.3 (*C*(O)NHBn), 143.3 (Ar*C*^{7a}), 137.7 (Ph*C*$^{1''}$CH$_2$NH), 132.8 (Ph*C*$^{1'}$CH), 130.0 (Ph*C*$^{2',6'}$H), 129.9 (Ar*C*^{6}H), 129.1 (Ar*C*^{3a}), 128.8 (Ph*C*$^{3'',5''}$H), 128.6 (Ph*C*$^{3',5'}$H), 128.4 (Ph*C*$^{4'}$H), 127.8 (Ph*C*$^{2'',6''}$H), 127.6 (Ph*C*$^{4''}$H), 124.1 (Ar*C*^{4}H), 123.1 (Ar*C*^{5}H), 108.1 (Ar*C*^{7}H), 78.8 (*C*-OH), 57.7 (*C*H-Ph), 43.9 (NH*C*H$_2$-Ph), 26.0 (N*C*H$_3$); **HRMS** (ESI$^+$) *m*/*z* calcd for $[M]^+$ $C_{24}H_{23}N_2O_3$ 387.1703, found 387.1696 (−1.8 ppm).

(±)-anti-**10**

PDA Ch1 211nm

Peak#	Ret. Time	Area%
1	20.236	49.854
2	23.785	50.146
Total		100.000

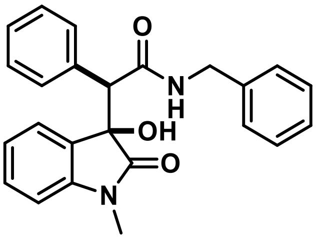

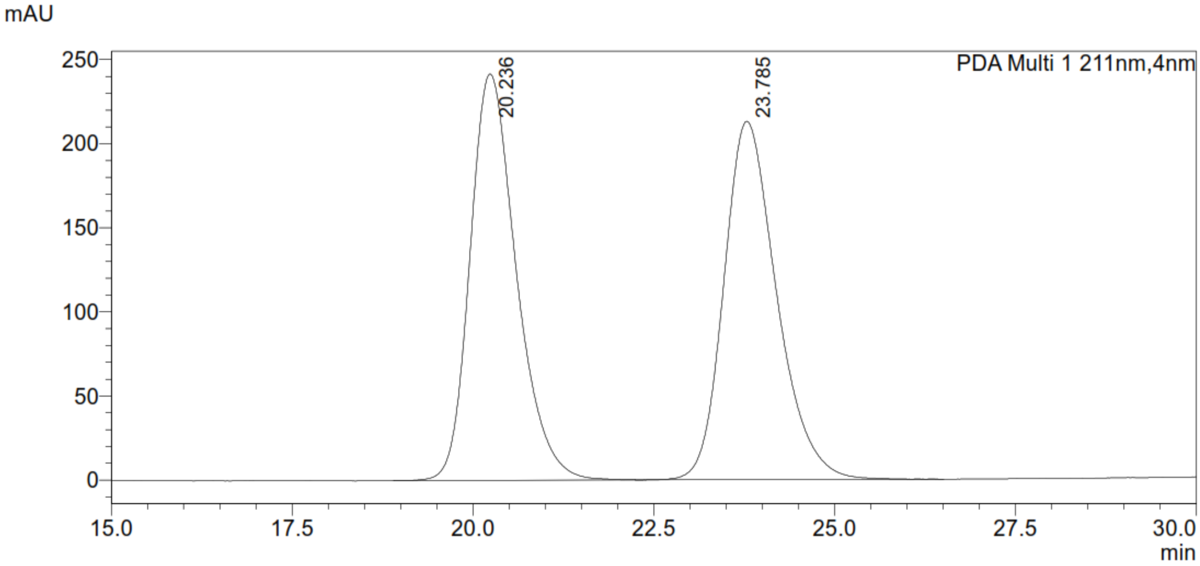

(+)-(2'''*R*,3*R*)-**10**

PDA Ch1 211nm

Peak#	Ret. Time	Area%
1	20.404	0.252
2	23.854	99.748
Total		100.000

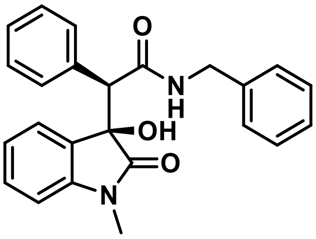

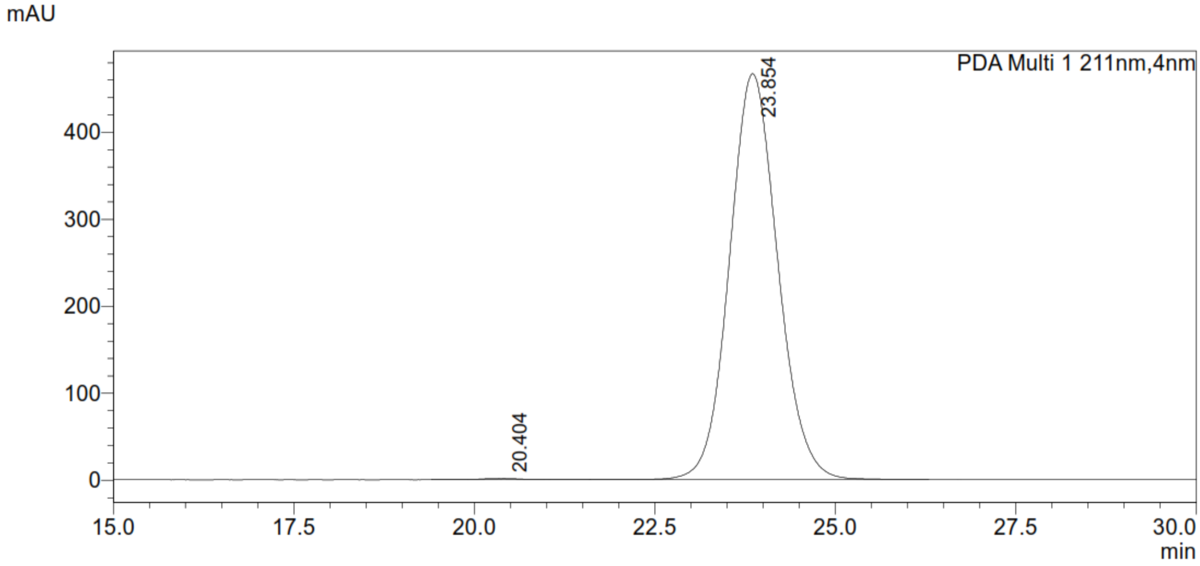

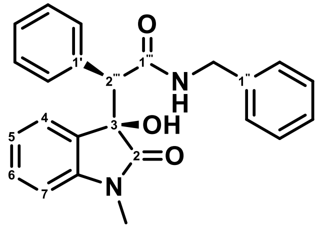

(2'''*R*,3*R*)-**10**

^{1}H NMR (400 MHz, $CDCl_3$)

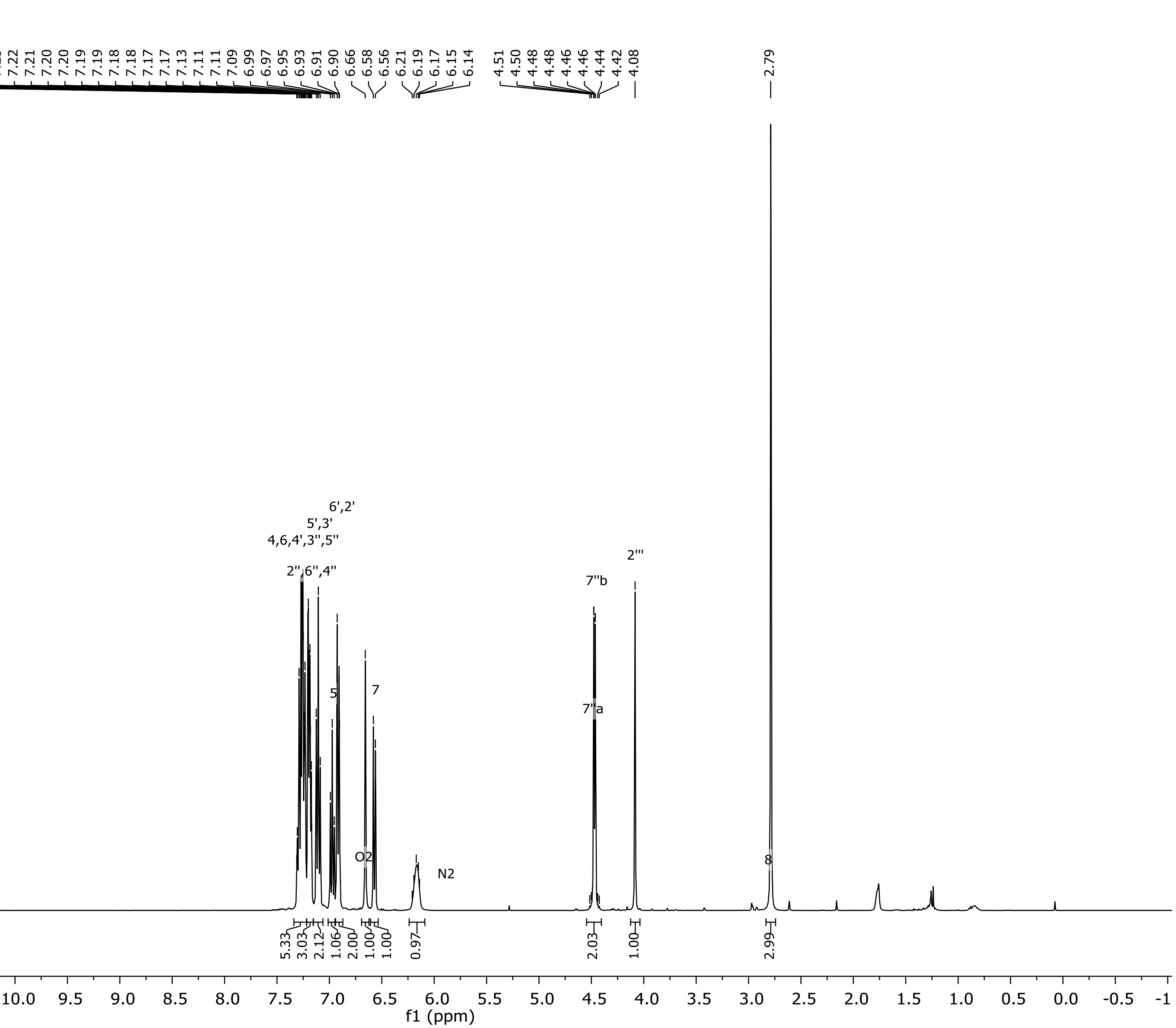

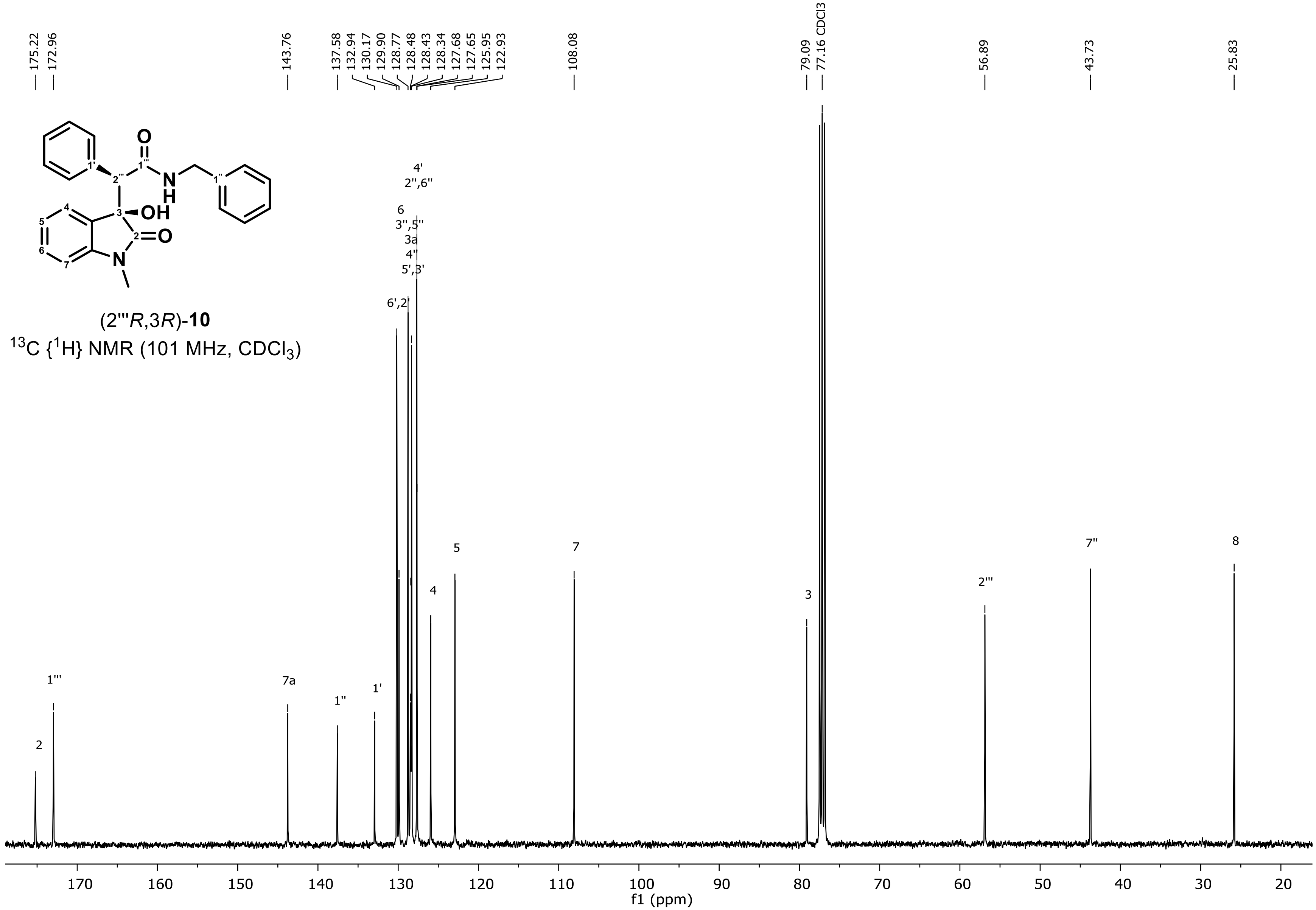

175.22
172.96
143.76
137.58
132.94
130.17
129.90
128.77
128.48
128.43
128.34
127.68
127.65
125.95
122.93
108.08
79.09
77.16 CDCl3
56.89
43.73
25.83
(2'''R,3R)-10
$^{13}C\ \{^{1}H\}$ NMR (101 MHz, $CDCl_3$)
f1 (ppm)

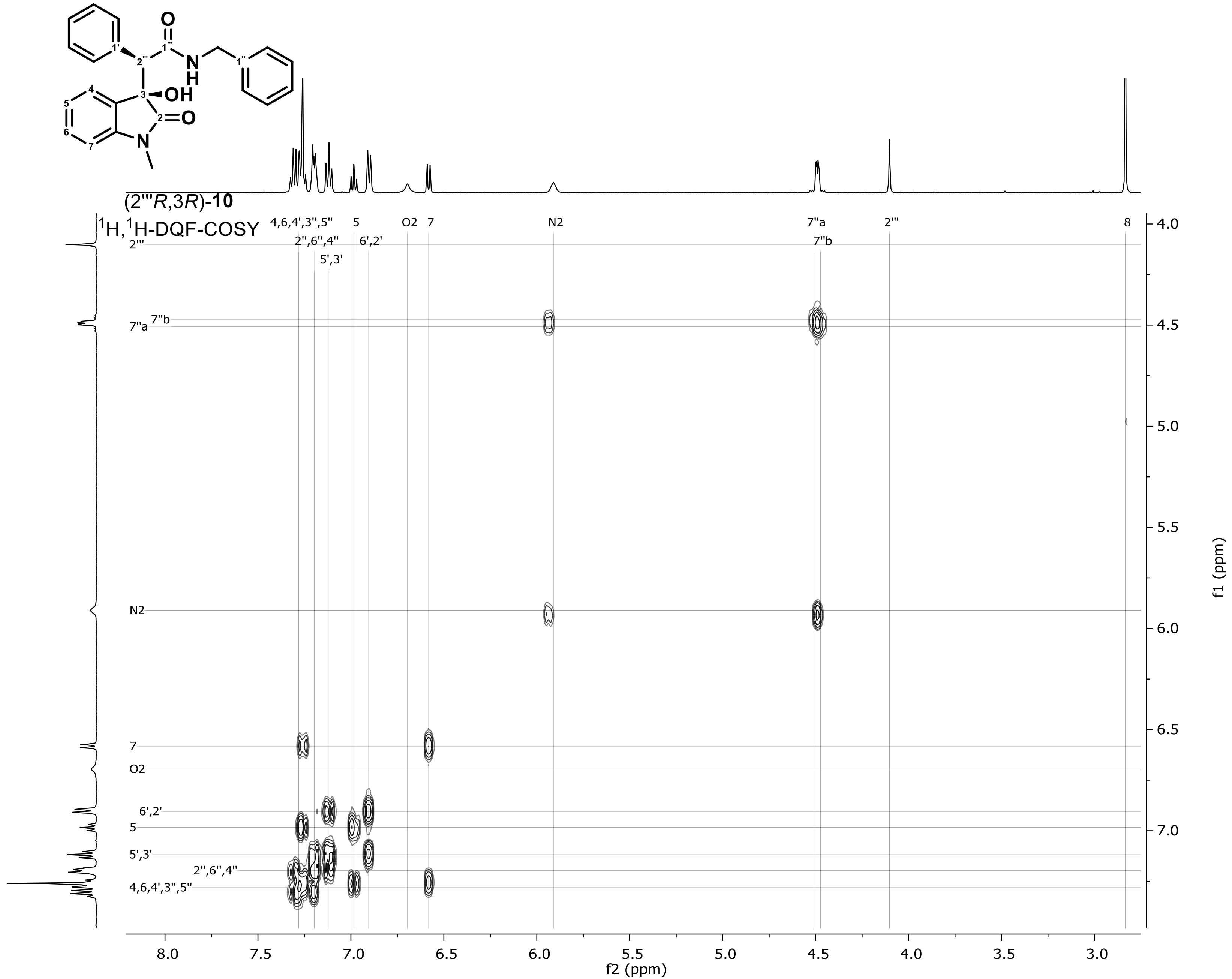
(2'''R,3R)-10
1H,1H-DQF-COSY
4,6,4',3'',5''
2'',6'',4''
5',3'
5
6',2'
O2
7
N2
7''a
7''b
2'''
8
f1 (ppm)
f2 (ppm)

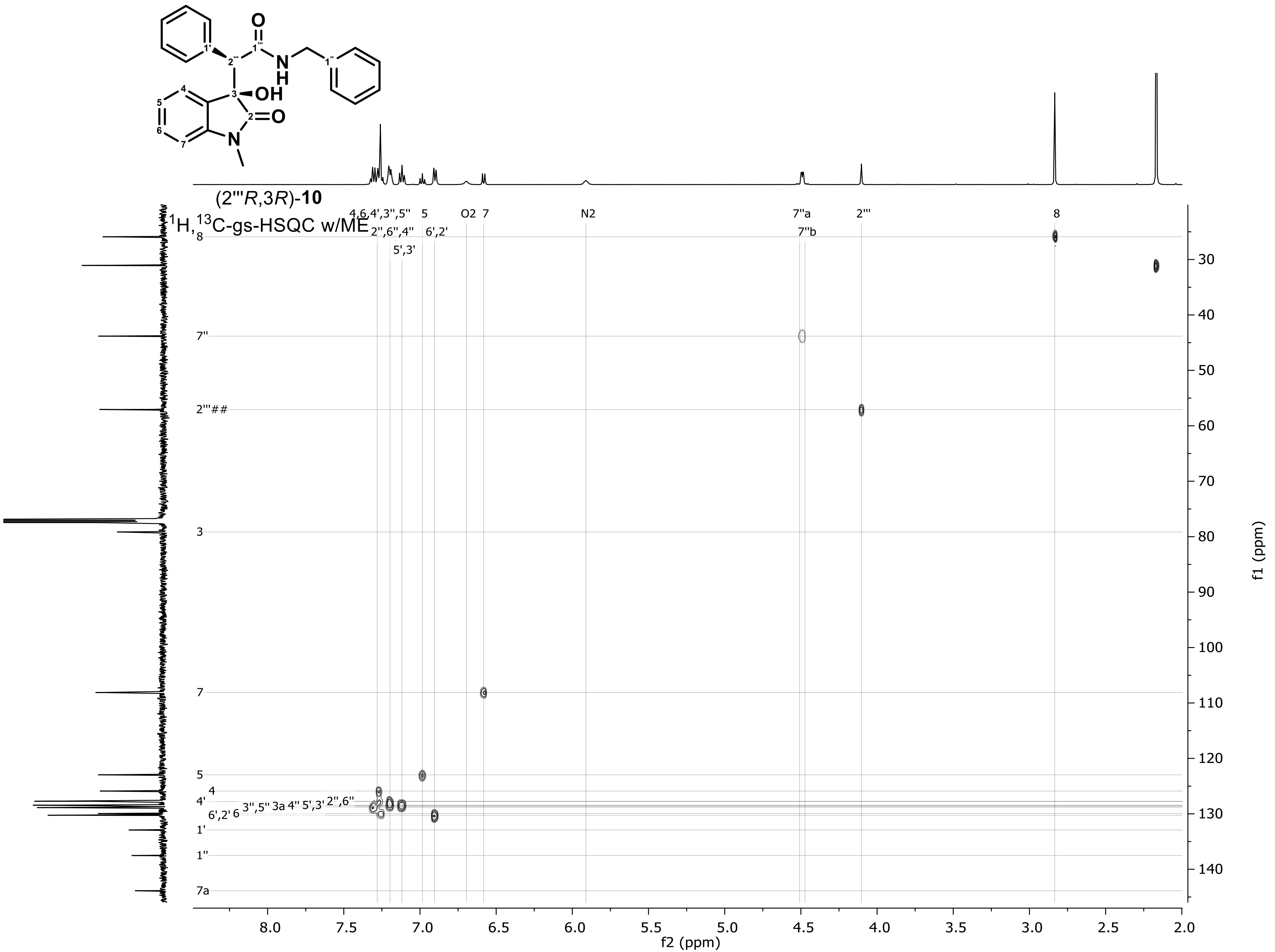
(2'''R,3R)-10
^{1}H,^{13}C-gs-HSQC w/ME
f2 (ppm)
f1 (ppm)

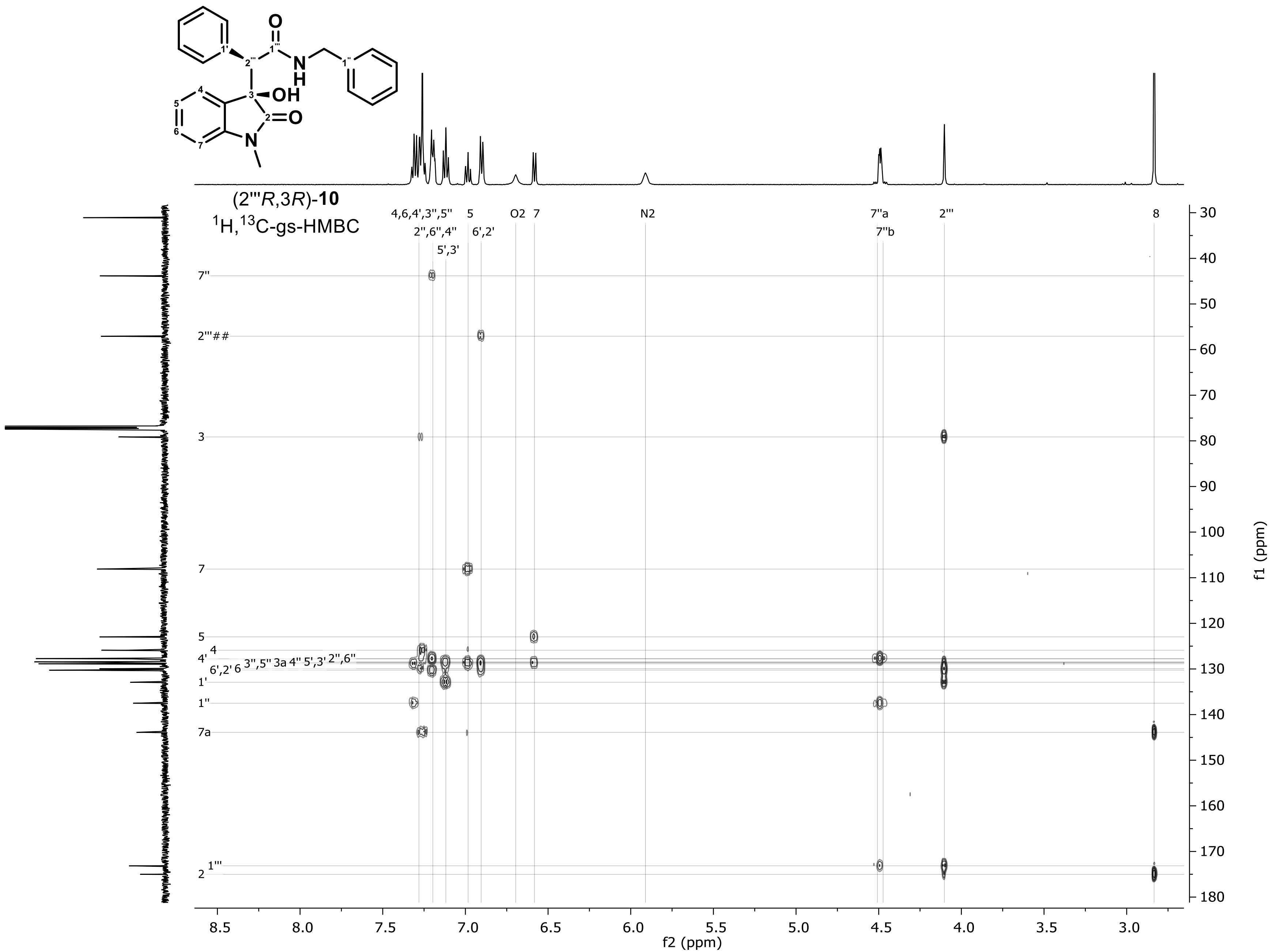

(2'''R,3R)-10
^{1}H,^{13}C-gs-HMBC
4,6,4',3",5"
2",6",4"
5',3'
5
6',2'
O2
7
N2
7"a
7"b
2'''
8
7"
2'''##
3
7
5
4
4'
6',2' 6 3",5" 3a 4" 5',3' 2",6"
1'
1"
7a
1'''
2
f2 (ppm)
f1 (ppm)

(±)-syn-**10**

PDA Ch1 211nm

Peak#	Ret. Time	Area%
1	23.381	48.690
2	32.302	51.310
Total		100.000

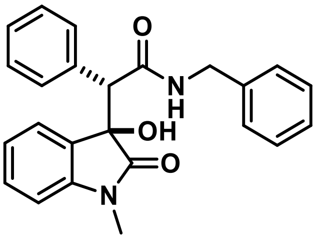

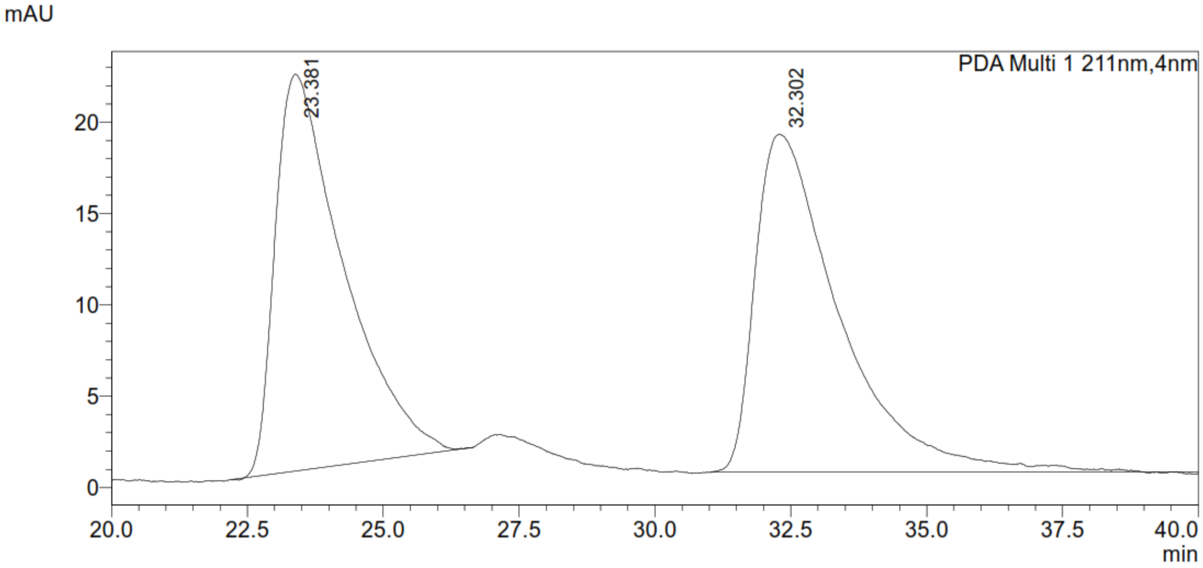

(–)-(2'''*S*,3*R*)-**10**

PDA Ch1 211nm

Peak#	Ret. Time	Area%
1	23.128	100.232
2	31.477	-0.232
Total		100.000

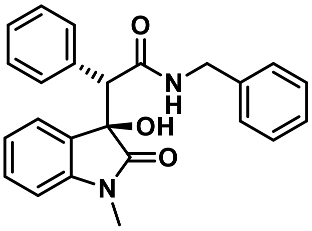

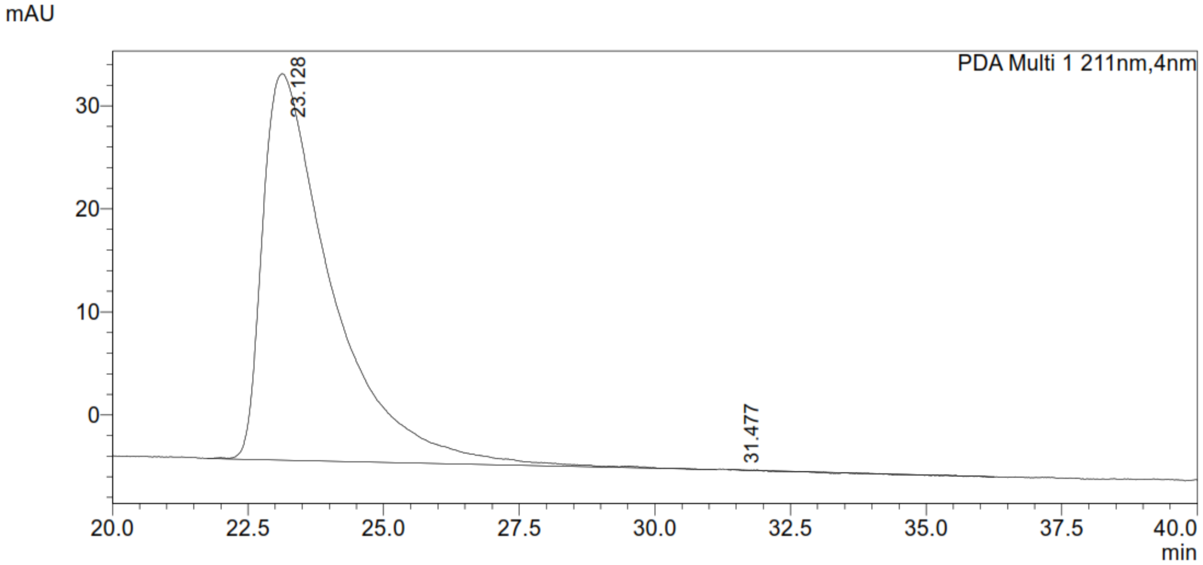

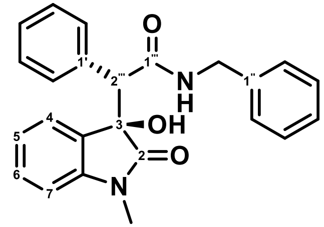

(2'''*S*,3*R*)-**10**

^{1}H NMR (400 MHz, $CDCl_3$)

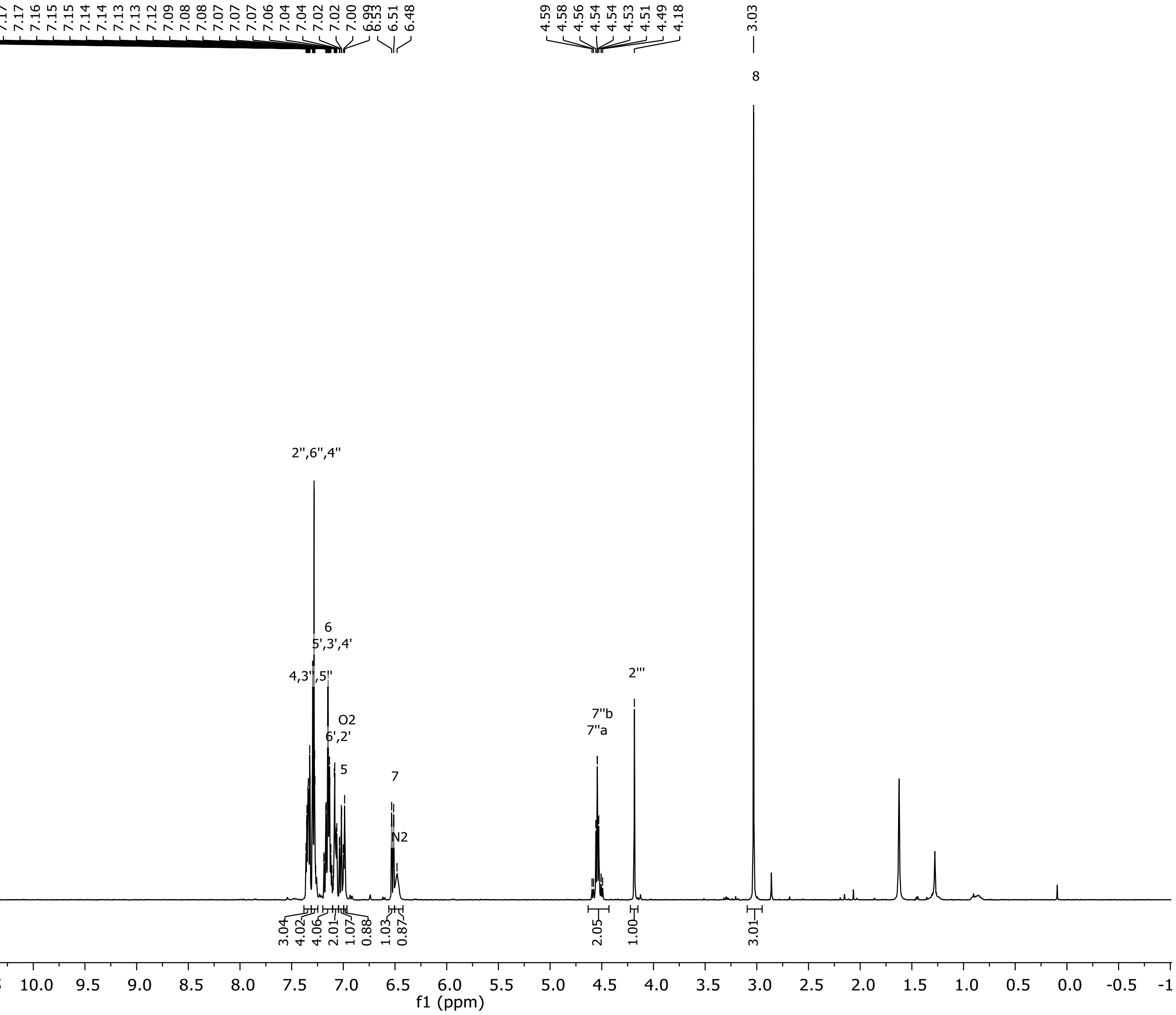

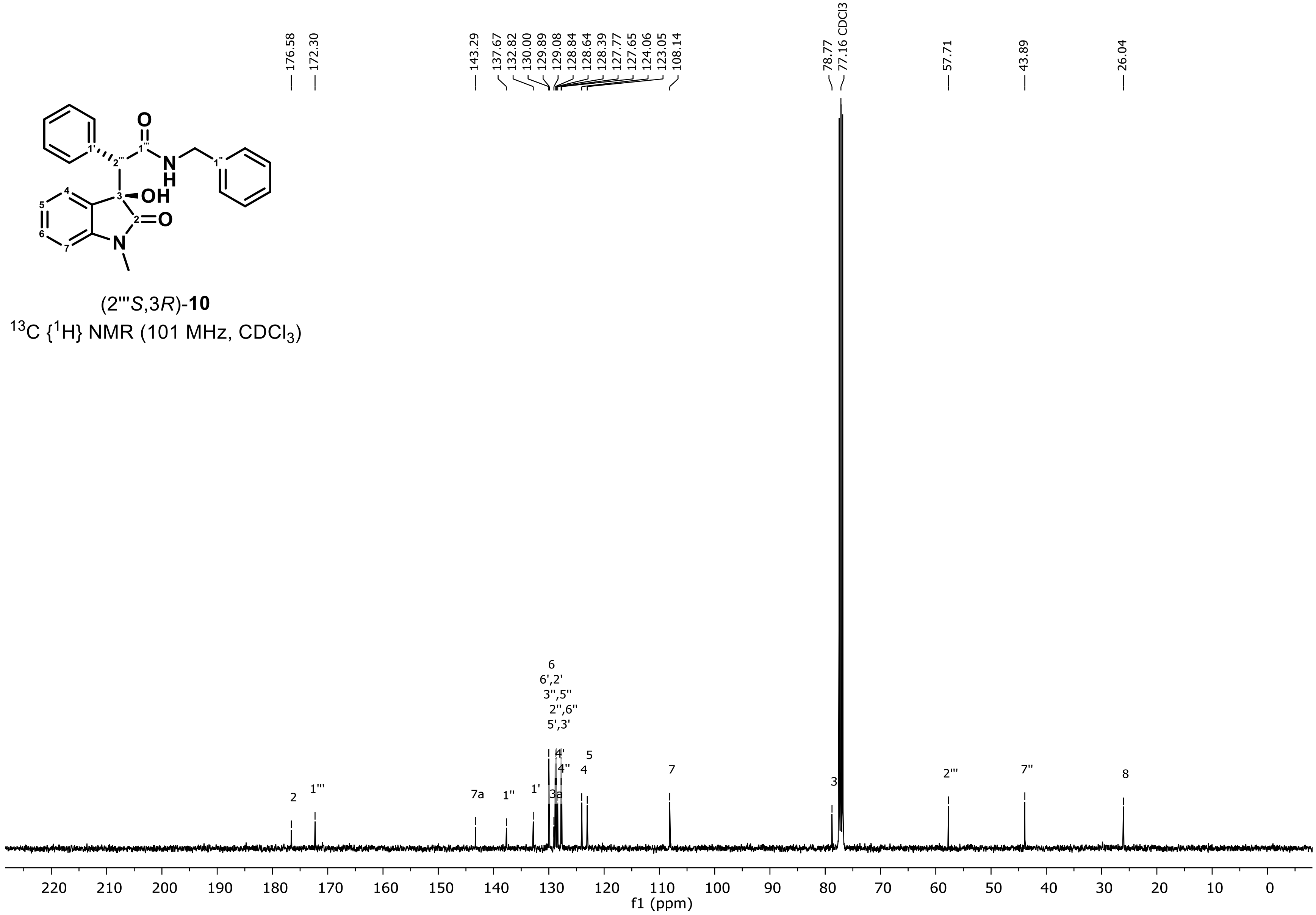

(2'''*S*,3*R*)-**10**

$^{13}C\ \{^1H\}$ NMR (101 MHz, $CDCl_3$)

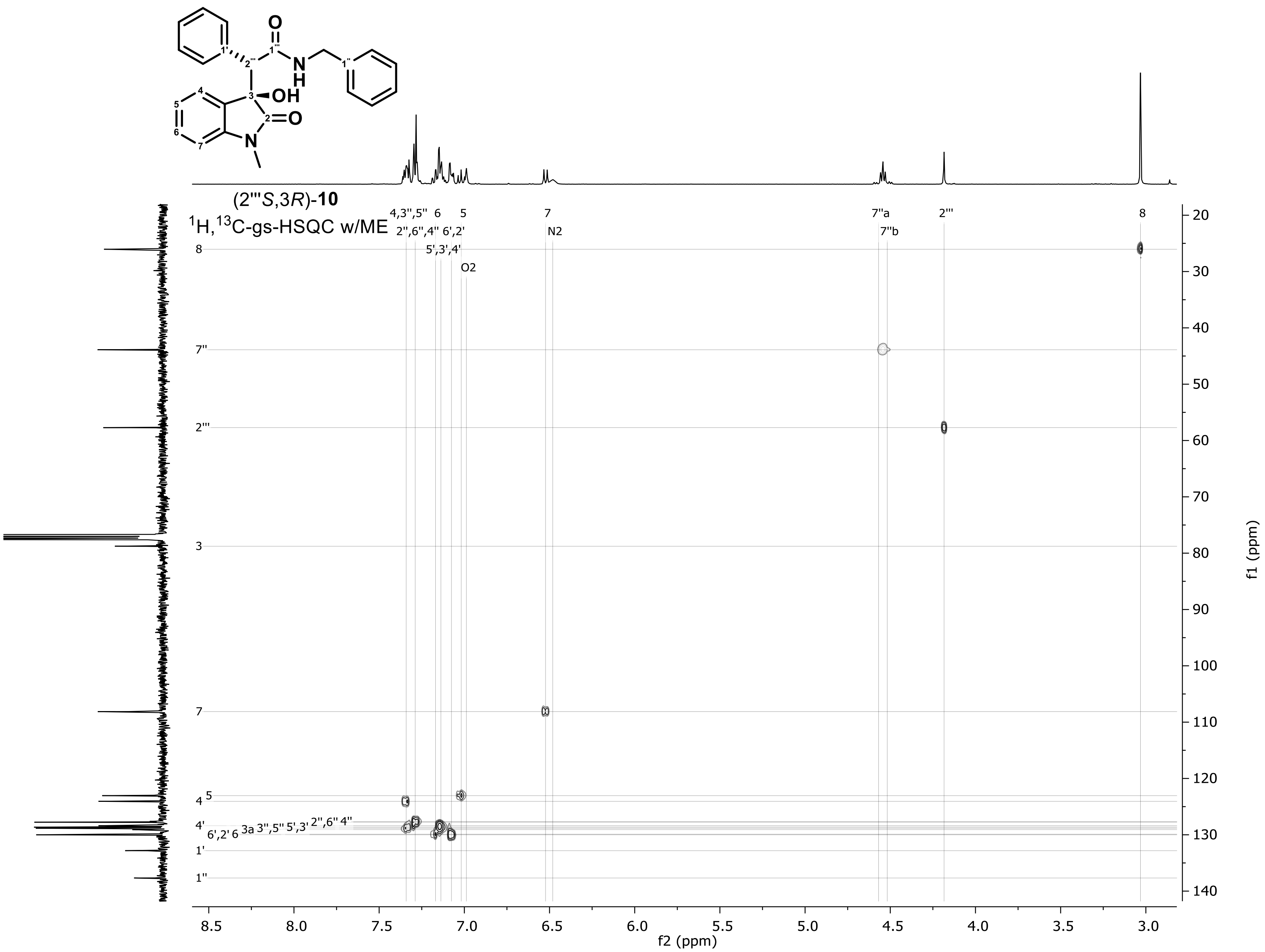
(2'''S,3R)-10
1H,13C-gs-HSQC w/ME
4,3'',5''
2'',6'',4''
6
5'',3',4'
5
6',2'
O2
7
N2
7''a
7''b
2'''
8
8
7''
2'''
3
7
4
5
4'
6',2'
6
3a
3'',5''
5',3'
2'',6''
4''
1'
1''
f1 (ppm)
f2 (ppm)

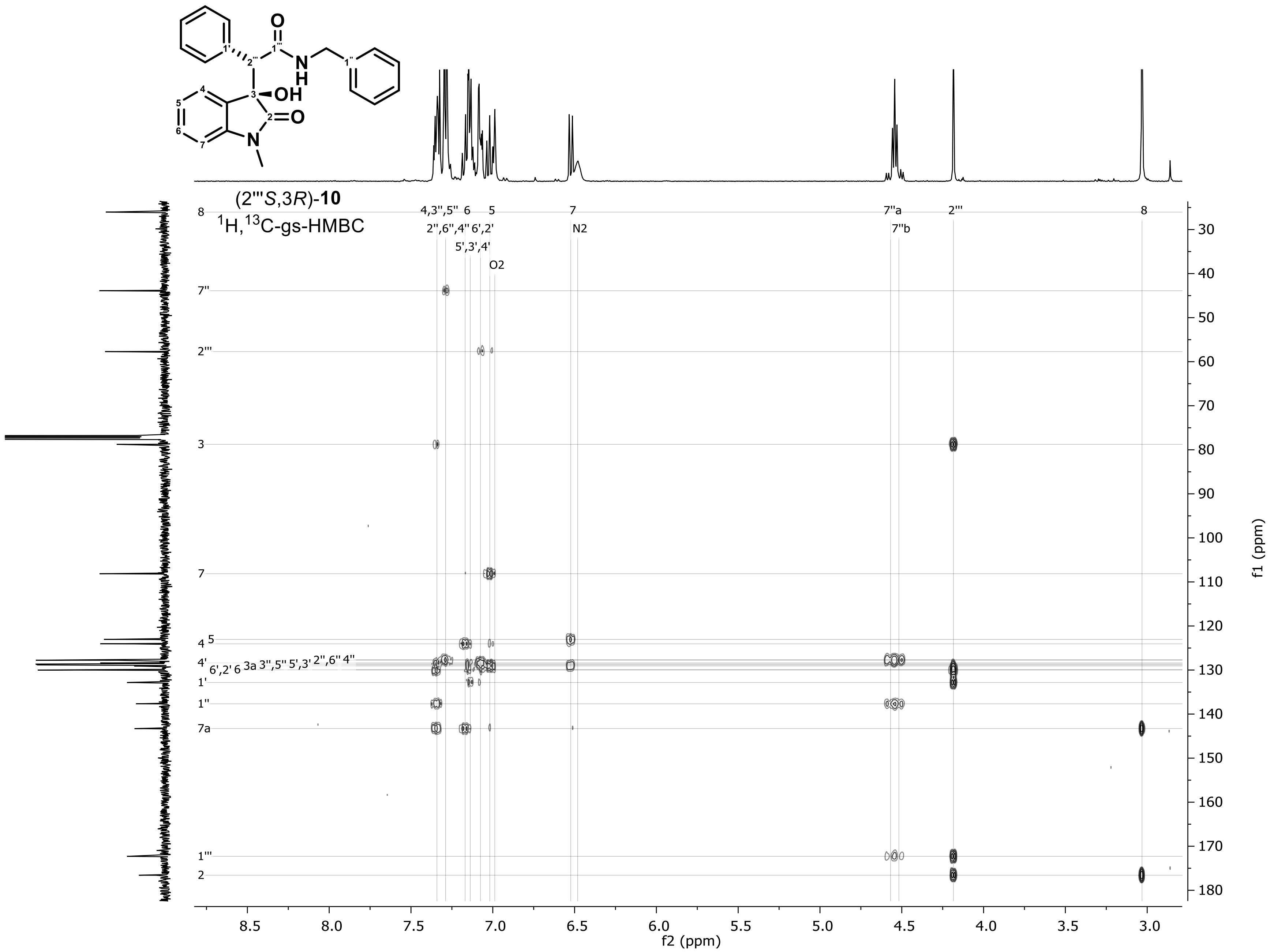

(2'''S,3R)-10
1H,13C-gs-HMBC
f2 (ppm)
f1 (ppm)

e) N-benzyl-2-(1-allyl-3-hydroxy-2-oxoindolin-3-yl)-2-phenylacetamide (11)

To a 25 ml round bottomed flask was added phenylacetic anhydride (95.3 mg, 0.375 mmol), *N*-allylisatin (46.8 mg, 0.250 mmol), and (2*S*,3*R*)-HyperBTM (3.9 mg, 0.012 mmol). The mixture was cooled to 0 °C and CH_2Cl_2 (6.0 ml, 0.04 M) and Hünig's base (54 µl, 0.312 mmol) were added. The mixture was stirred at 0 °C for 3 h. Benzylamine (82 µl, 0.750 mmol) was added and them reaction was left to be stirred overnight at room temperature. 1,3,5-Trimethoxybenzene (0.1 M soln in CH_2Cl_2 500 µl, 0.05 mmol) and the solvent was removed. Purification by column chromatography (Petrol : EtOAc 4:1 → 2:3) gave a fraction of the major diastereomer (52.3 mg), a fraction as a mixture of diastereomers (29.2 mg) and a fraction of the minor diastereomer with some impurities which was recolumned (CH_2Cl_2:EtOAc 9:1 → 7:3) to give a fraction of the minor diastereomer (13.3 mg). Combined yield 94.8 mg, 0.230 mmol, 92%, 80:20 d.r.

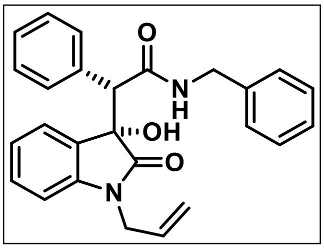

Major (2‴*S*,3*S*)-**11**: **m.p.** 152 – 156 (rac); **R*f*** 0.375 (Petrol : EtOAc 6:4); **Chiral HPLC analysis** Chiralpak AD-H (90:10 hexane:IPA, flow rate 2 ml·min^{-1}, 254 nm, 40 °C) t_R (2‴*S*, 3*S*)-**11**: 26.0 min, t_R (2‴*R*, 3*R*)-**11**: 44.7 min, >99:1; $\alpha_D^{20} = -15.2$ (*c* 2.16, $CHCl_3$); ν_{max} (thin film) 3323 (s, broad, OH), 3086 (w), 3061 (w), 3030 (w), 2920 (w), 1717 (s, C=O), 1705 (s, C=O), 1645 (s), 1612 (s), 1531 (s), 1489 (m), 1468 (s), 1454 (m), 1429 (m), 1358 (s), 1335 (m), 1263 (m), 1227 (m), 1177 (m), 1157 (w), 1113 (m), 1078 (w), 1030 (m), 988 (m), 928 (m), 752 (s); **^{1}H NMR** (400 MHz, $CDCl_3$) δ_H 7.50 (1H, dd, $^3J_{HH}$ = 7.5 Hz, $^4J_{HH}$ = 1.3 Hz, ArC^4H), 7.36 – 7.19 (7H, m, ArC^6H, $PhC^{4'}H$, $PhC^{2'',3'',4'',5'',6''}H$), 7.13 (2H, app t, $^3J_{HH}$ = 7.7 Hz, $PhC^{3',5'}H$), 7.06 (1H, app td, $^3J_{HH}$ = 7.6 Hz, $^4J_{HH}$ = 1.0 Hz, ArC^5H), 6.97 – 6.88 (2H, m, $PhC^{2',6'}H$), 6.60 (1H, d, $^3J_{HH}$ = 7.8 Hz, ArC^7H), 6.72 (1H, s, O*H*), 6.33 (1H, app t, $^3J_{HH}$ = 6.0 Hz, 5.8 Hz, N*H*), 5.19 (1H, dddd, $^3J_{HHtrans}$ = 17.2 Hz, $^3J_{HHcis}$ = 10.3 Hz, $^3J_{HH}$ = 5.7 Hz, 4.6 Hz, C*H*=CH_2), 4.90 (1H, app dq, $^3J_{HHcis}$ = 10.3 Hz, $^3J_{HH}$ = $^4J_{HH}$ = 1.4 Hz, $CH=CH_{cis}H_{trans}$), 4.60 (1H, app dq, $^3J_{HHtrans}$ = 17.2 Hz, $^3J_{HH}$ = $^4J_{HH}$ = 1.6 Hz, $CH=CH_{cis}H_{trans}$), 4.54 (1H, dd, $^2J_{HH}$ = 15.4 Hz, $^3J_{HH}$ = 6.0 Hz, $NHCH_aH_b$-Ph), 4.50 (1H, dd, $^2J_{HH}$ = 15.4 Hz, $^3J_{HH}$ = 5.8 Hz, $NHCH_aH_b$-Ph), 4.29 (1H, s, C*H*-Ph), 4.18 (1H, app ddt, $^2J_{HH}$ = 16.4 Hz, $^3J_{HH}$ = 4.6 Hz, 1.9 Hz, $^4J_{HH}$ = 1.9 Hz, $NCH_aH_bCH=CH_2$), 3.75 (1H, app ddt, $^2J_{HH}$ = 16.4 Hz, $^3J_{HH}$ = 5.7 Hz, 1.6 Hz, $^4J_{HH}$ = 1.6 Hz, $NCH_aH_bCH=CH_2$); **^{13}C {^{1}H} NMR** (101 MHz, $CDCl_3$) δ_C 174.8 (*C*(O)N-allyl), 172.8 (*C*(O)NHBn), 143.1 (ArC^{7a}), 137.6 ($PhC^{1''}CH_2NH$), 132.8 ($PhC^{1'}CH$), 130.7 (*C*H=CH_2), 130.4 ($PhC^{2',6'}H$), 129.8 (ArC^6H), 128.7 ($PhC^{3'',5''}H$), 128.5_4 (ArC^{3a}), 128.4_6 ($PhC^{3',5'}H$), 128.4_1 ($PhC^{4''}H$), 127.6_2 ($PhC^{2'',6''}H$), 127.5_9 ($PhC^{4'}H$), 126.0 (ArC^4H), 123.0 (ArC^5H), 117.2 (CH=*C*H_2), 109.1 (ArC^7H), 79.1 (*C*-OH), 57.1 (*C*H-Ph), 43.7 (NH*C*H_2-Ph), 42.1 (N*C*H_2-CH=CH_2); ***m/z*** (ESI$^+$) 91 ($[C_7H_7]^+$ 14%), 188 ($[M-BnC(O)NHBn]^+$ 13%), 226 ($[BnC(O)NHBn+H]^+$ 100%), 262 ($[M-BnNHCOO]^+$ 95%), 393 ($[M+H]^+$ 13%), 413 ($[M+H]^+$ 91%), 414 ($[M(^{13}C)+H]^+$ 26%), 415 ($[M(^{13}C_2)+H]^+$ 4%), 435 ($[M+Na]^+$ 24%), 451 ($[M+K]^+$ 13%), 646 (15%), 847 ($[2M+Na]^+$ 30%), 879 (12%); **HRMS** (ESI$^+$) *m/z* calcd for $[M+H]^+$ $C_{26}H_{25}N_2O_3$ 413.1870, found 413.1858 (−0.5 ppm).

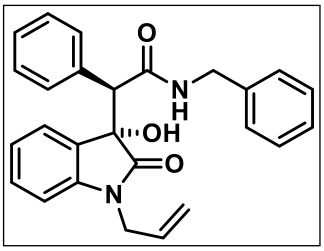

Minor (2‴*R*,3*S*)-**11**: **R*f*** 0.236 (Petrol : EtOAc 6:4); **Chiral HPLC analysis** Chiralpak AD-H (90:10 hexane:IPA, flow rate 2 ml·min^{-1}, 254 nm, 40 °C) t_R (2‴*S*, 3*R*)-**11**: 16.1 min, t_R (2‴*R*, 3*S*)-**11**: 18.7 min, >99:1; $\alpha_D^{20} = +56.0$ (*c* 0.62, $CHCl_3$); ν_{max} (thin film) 3271 (s, broad, OH), 3088 (w), 3063 (w), 3030 (), 2922 (w), 2853 (w), 1697 (s, C=O), 1639 (s), 1612 (s), 1576 (m), 1489 (m), 1468 (m), 1454 (m), 1431 (w), 1362 (m), 1302 (w), 1215 (w), 1177 (m), 1109 (w), 1078 (m), 1030 (w), 984 (m), 928 (m), 795 (w), 750 (s); **^{1}H NMR** (400 MHz, $CDCl_3$) (not all signals resolved in NMR) δ_H 7.38 – 7.25 (6H, m, ArC^4H, 5 × PhC*H*), 7.16 – 7.04 (6H, m, ArC^6H, $PhC^{2',6'}H$, 3 × PhC*H*), 7.00 (1H, app td, $^3J_{HH}$ = 7.6 Hz, $^4J_{HH}$ = 1.0 Hz, ArC^5H), 6.92 (1H, s, O*H*), 6.57 (1H, s(b), N*H*), 6.53 (1H, ddd, $^3J_{HH}$ = 7.8 Hz, $^4J_{HH}$ = 1.0 Hz, $^5J_{HH}$ = 0.6 Hz, ArC^7H), 5.59 (1H, dddd, $^3J_{HHtrans}$ = 17.2 Hz, $^3J_{HHcis}$ = 10.3 Hz, $^3J_{HH}$ = 5.4 Hz, 5.2 Hz, C*H*=CH_2), 5.09 (1H, app dq, $^3J_{HHcis}$ = 10.3 Hz, $^3J_{HH}$ = $^4J_{HH}$ = 1.4 Hz, $CH=CH_{cis}H_{trans}$), 4.96 (1H, app dq, $^3J_{HHtrans}$ = 17.2 Hz, $^3J_{HH}$ = $^4J_{HH}$ = 1.5 Hz, $CH=CH_{cis}H_{trans}$), 4.56 (1H, dd, $^2J_{HH}$ = 15.0 Hz, $^3J_{HH}$ = 5.9 Hz, $NHCH_aH_b$-Ph), 4.51 (1H, dd, $^2J_{HH}$ = 15.0 Hz, $^3J_{HH}$ = 5.7 Hz, $NHCH_aH_b$-Ph), 4.31 (1H, app ddt, $^2J_{HH}$ = 16.3 Hz, $^3J_{HH}$ = 5.2 Hz, 1.7 Hz, $^4J_{HH}$ =

1.7 Hz, NCH$_a$$H_b$CH=CH$_2$), 4.21 (1H, s, C$H$-Ph), 4.03 (1H, app ddt, $^2J_{HH}$ = 16.3 Hz, $^3J_{HH}$ = 5.4 Hz, 1.7 Hz, $^4J_{HH}$ = 1.7 Hz, NCH_aH$_b$CH=CH$_2$); **^{13}C{^{1}H} NMR** (101 MHz, CDCl$_3$) δ_C 176.4 (C(O)N-allyl), 172.3 (C(O)NHBn), 142.7 (ArC^{7a}), 137.7 (Ph$C^{1''}$CH$_2$NH), 132.9 (Ph$C^{1'}$CH), 130.9 (CH=CH$_2$), 130.2 (Ph$C^{2',6'}$H), 129.9 (Ph$C^{3,5}$H), 129.1 (ArC^{3a}), 128.9 (Ph$C^{3,5}$H), 128.8 (Ph$C^{3,5}$H), 128.4 (ArC^6H), 127.8 (Ph$C^{2'',6''}$H), 127.7 (PhC^4H), 124.2 (ArC^4H), 123.1 (ArC^5H), 117.9 (CH=CH$_2$), 109.2 (ArC^7H), 78.7 (C-OH), 57.7 (CH-Ph), 43.9 (NHCH$_2$-Ph), 42.4 (NCH$_2$CH=CH$_2$); ***m/z*** (ESI$^+$) 91 ([C$_7$H$_7$]$^+$ 7%), 188 ([M-BnC(O)NHBn]$^+$ 22%), 226 ([BnC(O)NHBn+H]$^+$ 51%), 262 ([M-BnNHCOO]$^+$ 30%), 413 ([M+H]$^+$ 100%), 414 ([M(^{13}C)+H]$^+$ 28%), 415 ([M(^{13}C$_2$)+H]$^+$ 4%), 435 ([M+Na]$^+$ 11%), 451 ([M+K]$^+$ 7%), 646 (9%), 825 (8%), 847 ([2M+Na]$^+$ 25%), 879 (7%); **HRMS** (ESI$^+$) m/z calcd for [M+H]$^+$ $C_{26}H_{25}N_2O_3$ 413.1870, found 413.1858 (−0.5 ppm).

(±)-anti-**11** + (±)-syn-**11**

Detector A Channel 2 254nm

Peak#	Ret. Time	Area%
1	16.261	6.378
2	18.760	6.487
3	26.315	43.156
4	43.849	43.979
Total		100.000

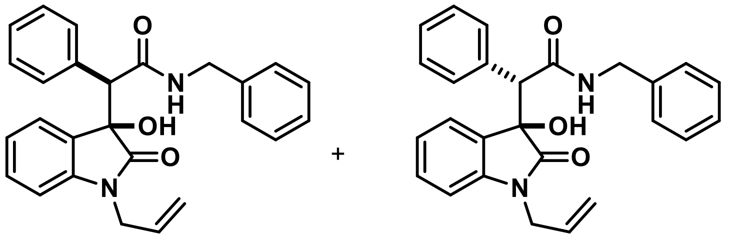

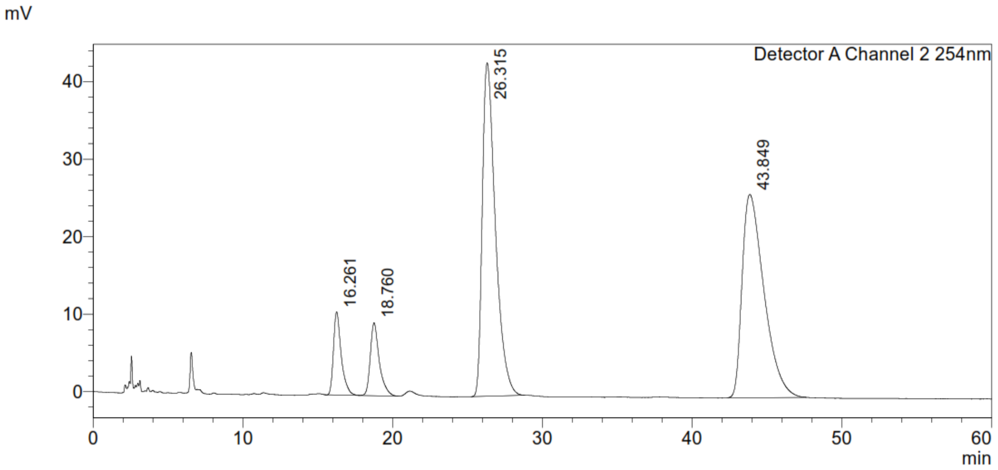

(2'''*S*,3*S*)-**11** + (2'''*R*,3*S*)-**11**

Detector A Channel 2 254nm

Peak#	Ret. Time	Area%
1	16.234	8.303
2	18.775	0.096
3	26.024	90.880
4	44.732	0.721
Total		100.000

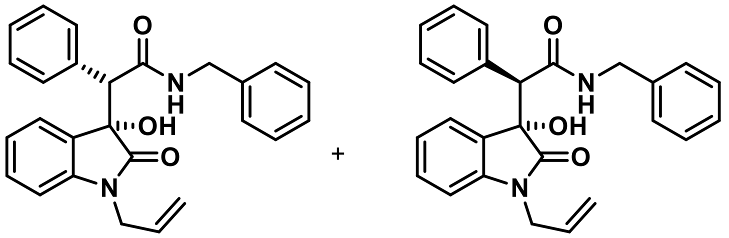

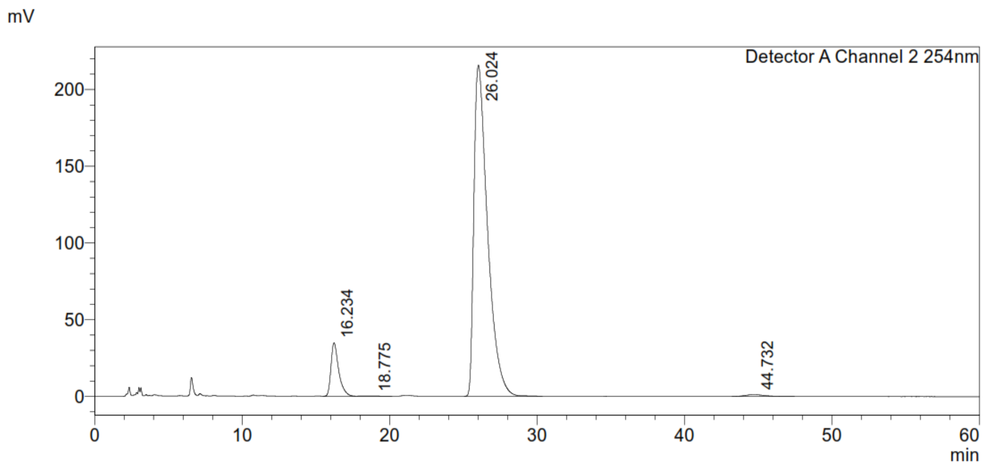
mV
Detector A Channel 2 254nm
200
150
100
50
0
16.234
18.775
26.024
44.732
0
10
20
30
40
50
60
min

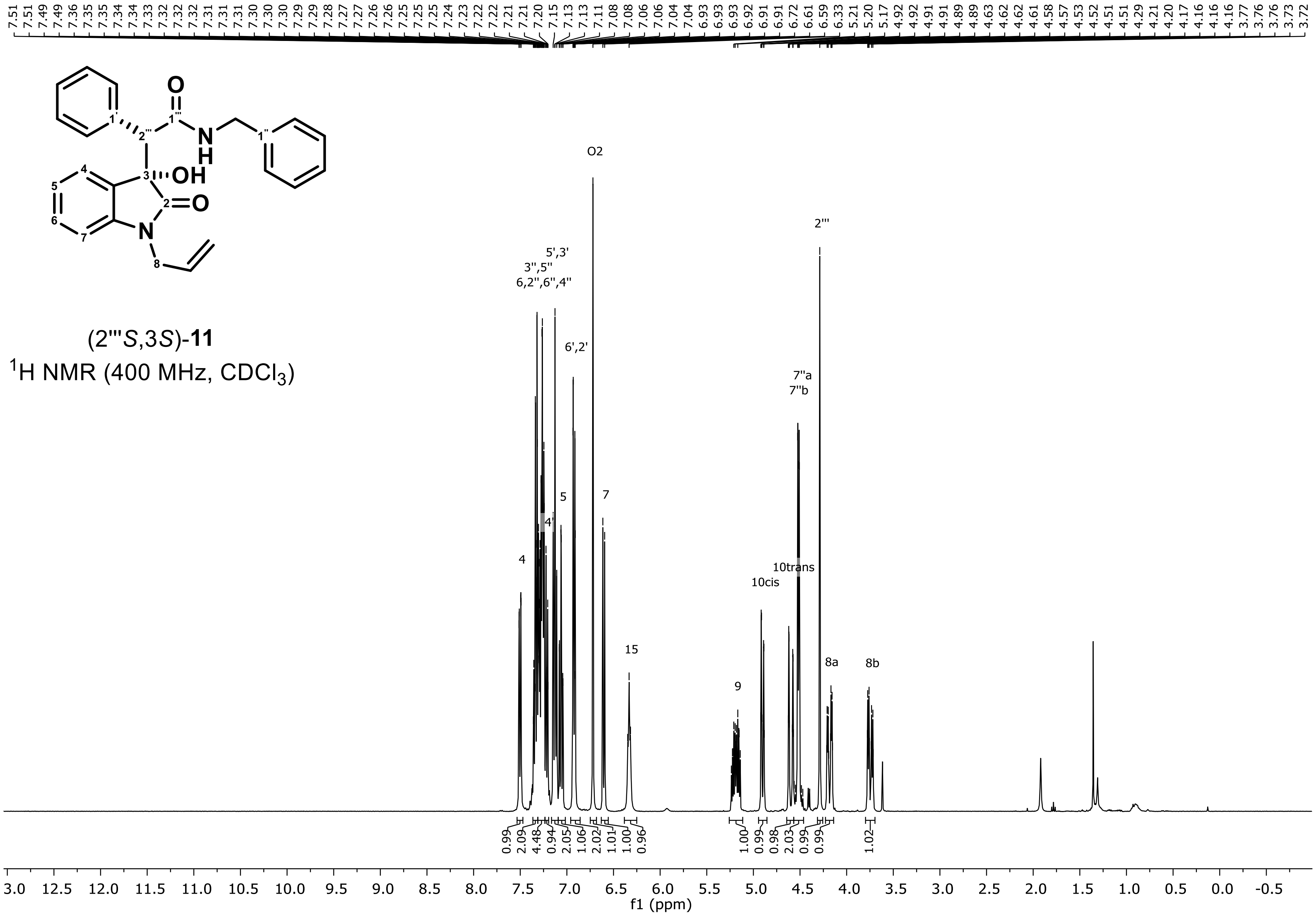

(2'''*S*,3*S*)-**11**

^{1}H NMR (400 MHz, $CDCl_3$)

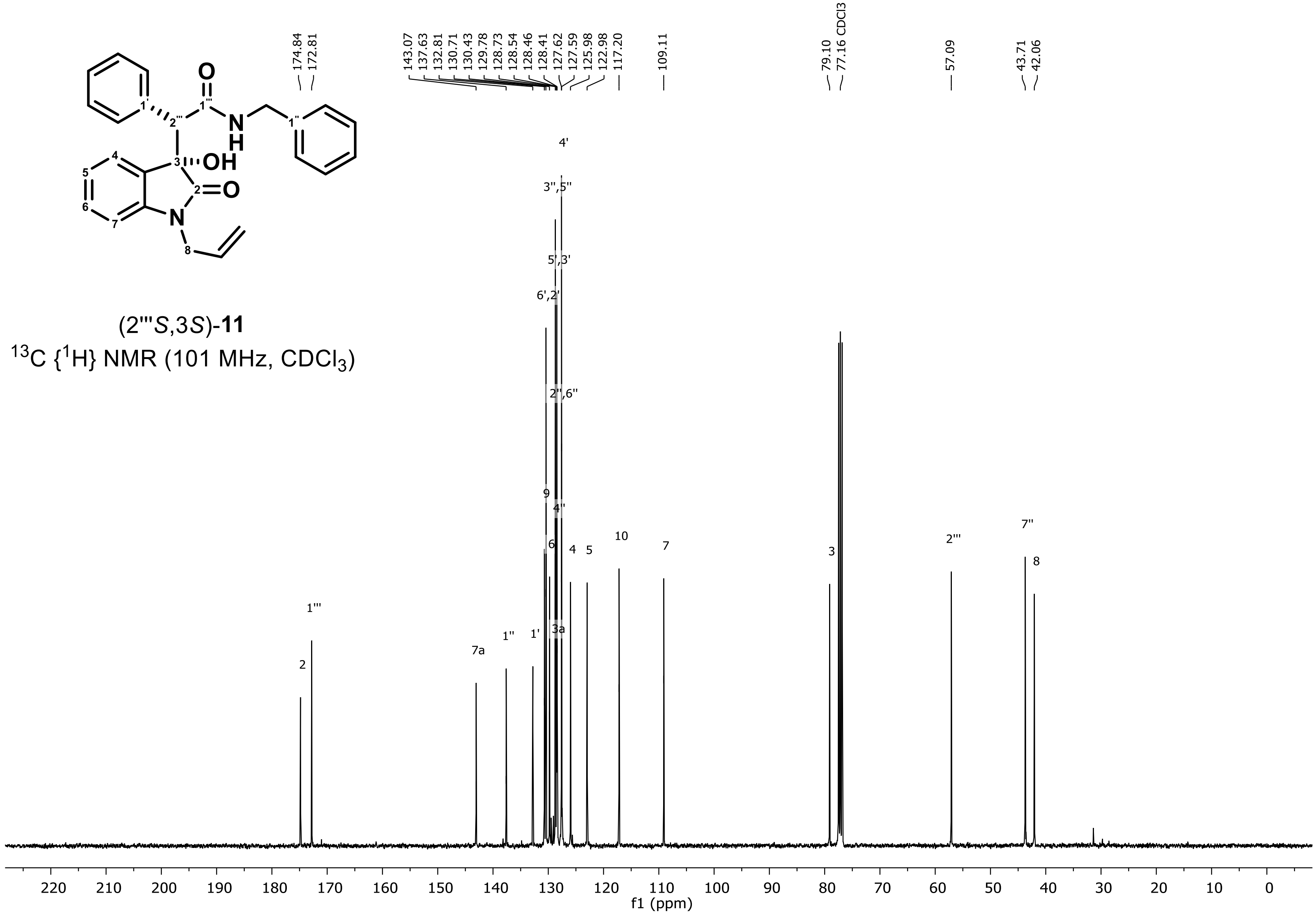

(2'''*S*,3*S*)-**11**

$^{13}C\ \{^{1}H\}$ NMR (101 MHz, $CDCl_3$)

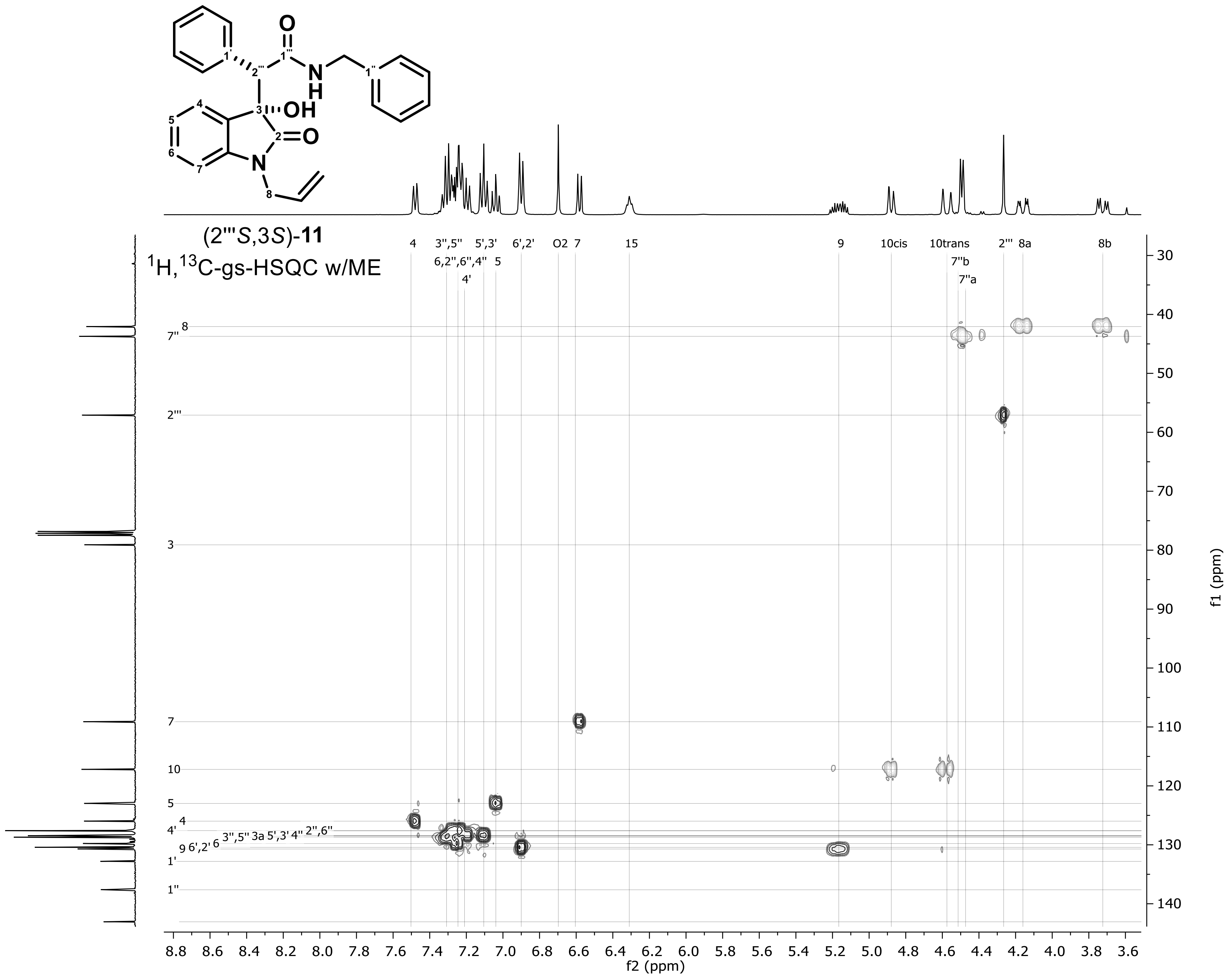

(2‴*S*,3*S*)-**11**

$^{1}H,^{13}C$-gs-HSQC w/ME

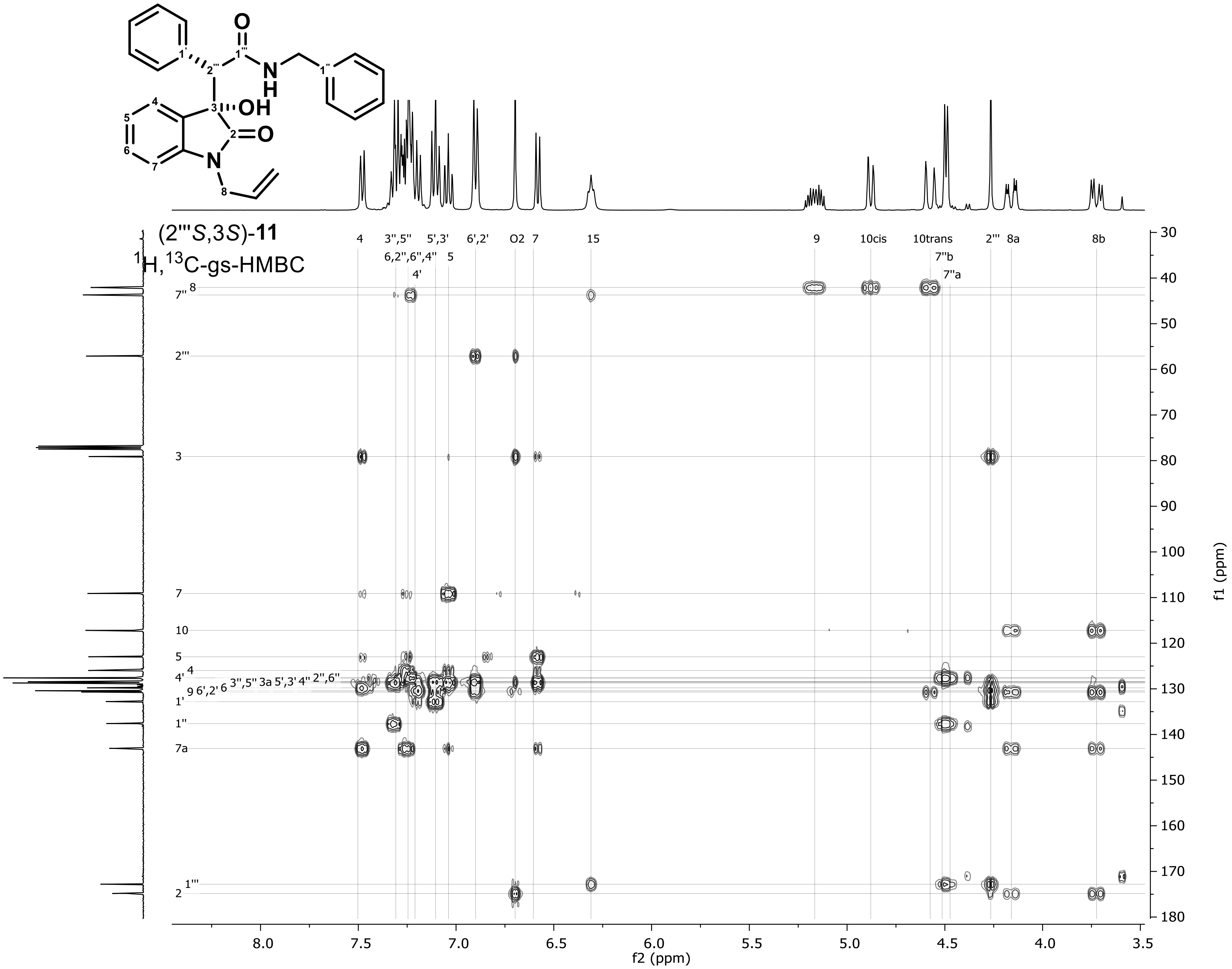

(2'''S,3S)-11
¹H,¹³C-gs-HMBC
f2 (ppm)
f1 (ppm)

(±)-anti-**11** + (±)-syn-**11**

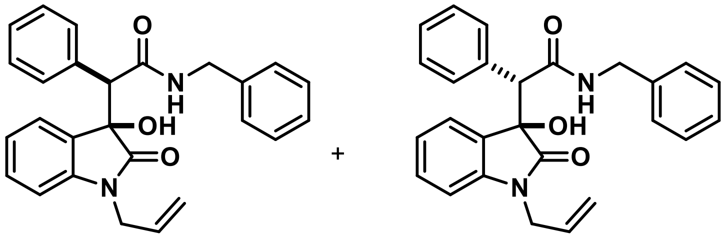

Detector A Channel 2 254nm

Peak#	Ret. Time	Area%
1	16.261	6.378
2	18.760	6.487
3	26.315	43.156
4	43.849	43.979
Total		100.000

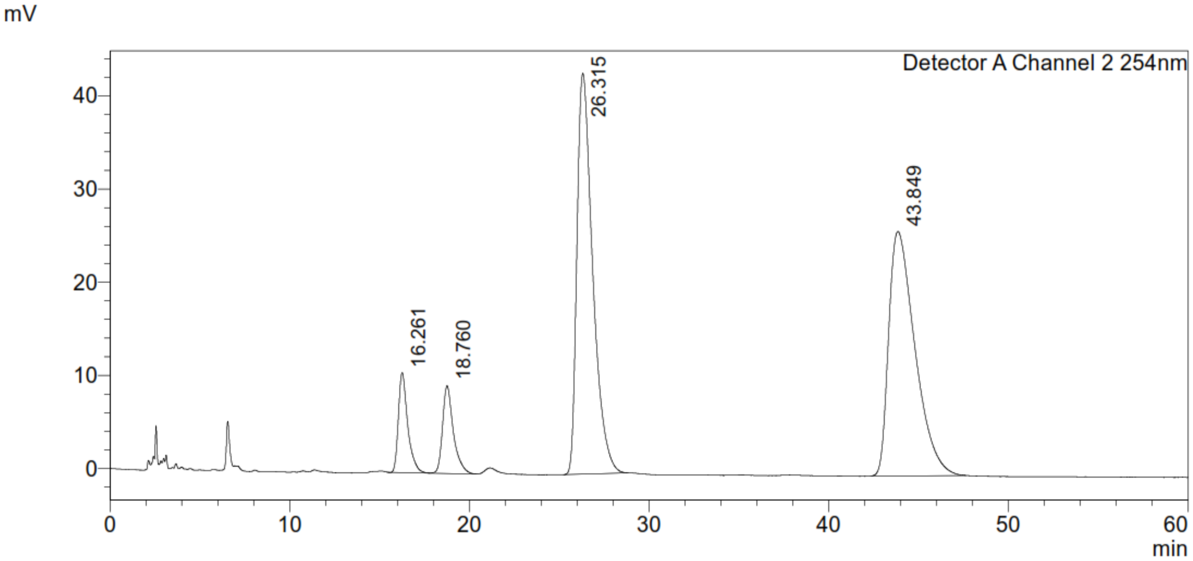

(+)-(2'''*R*,3*S*)-**11**

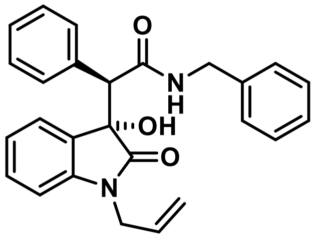

Detector A Channel 2 254nm

Peak#	Ret. Time	Area%
1	16.067	99.687
2	18.743	0.313
Total		100.000

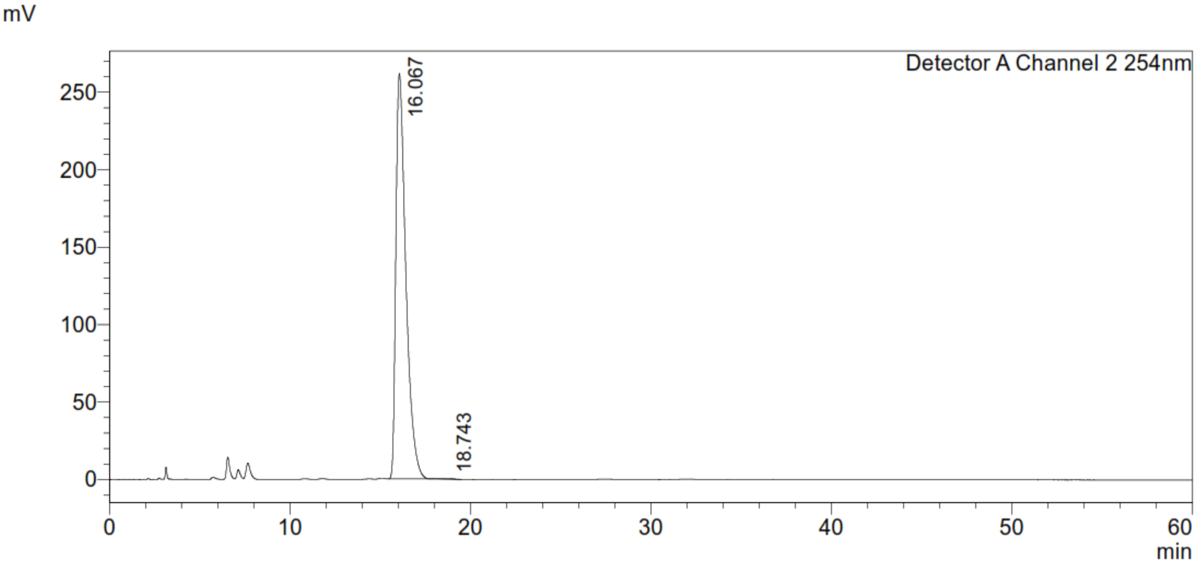

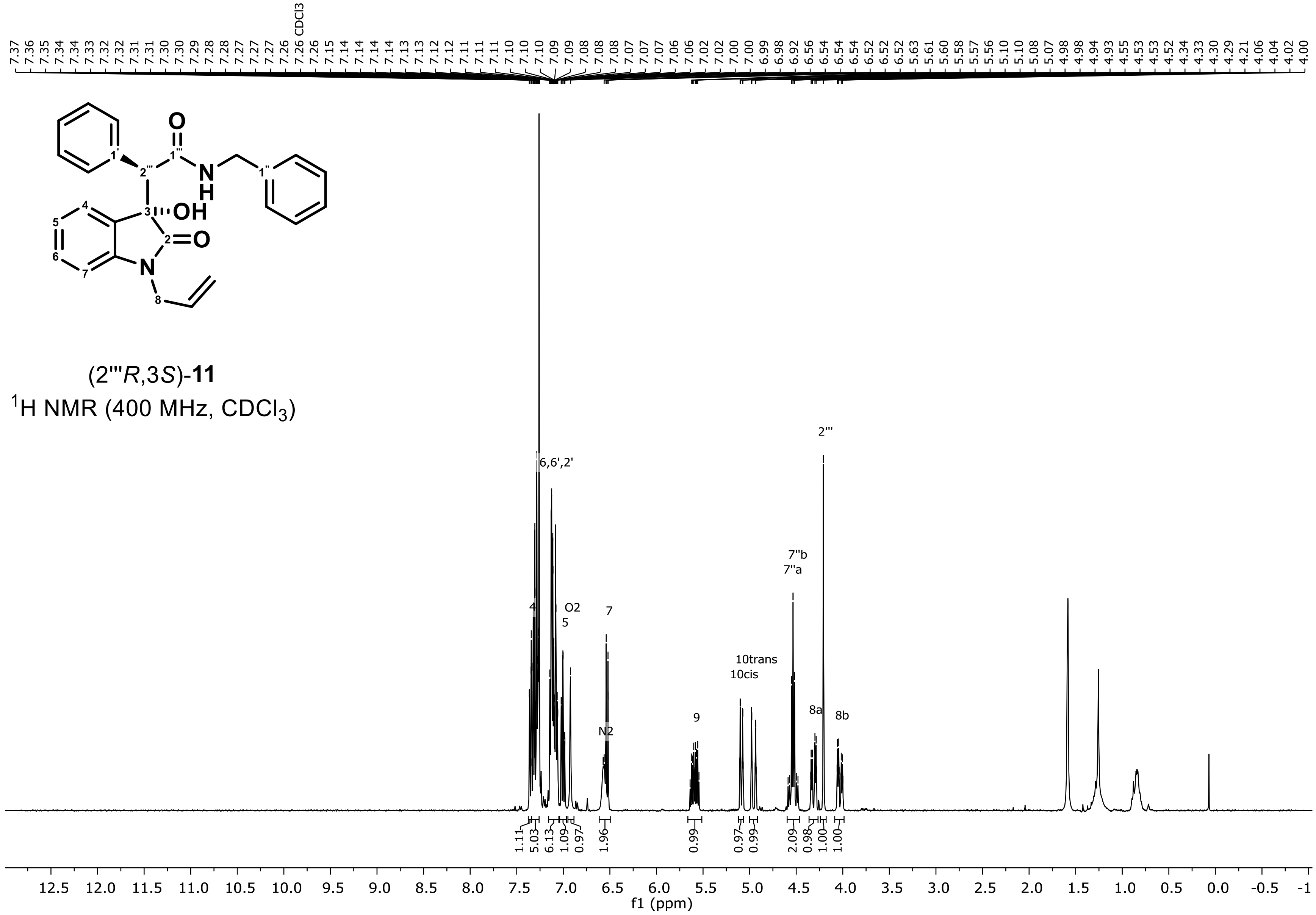

(2'''*R*,3*S*)-**11**

^{1}H NMR (400 MHz, $CDCl_3$)

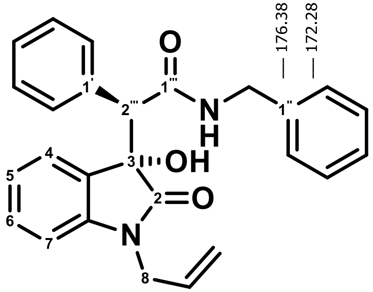

(2'''*R*,3*S*)-**11**

$^{13}C\ \{^{1}H\}$ NMR (101 MHz, $CDCl_3$)

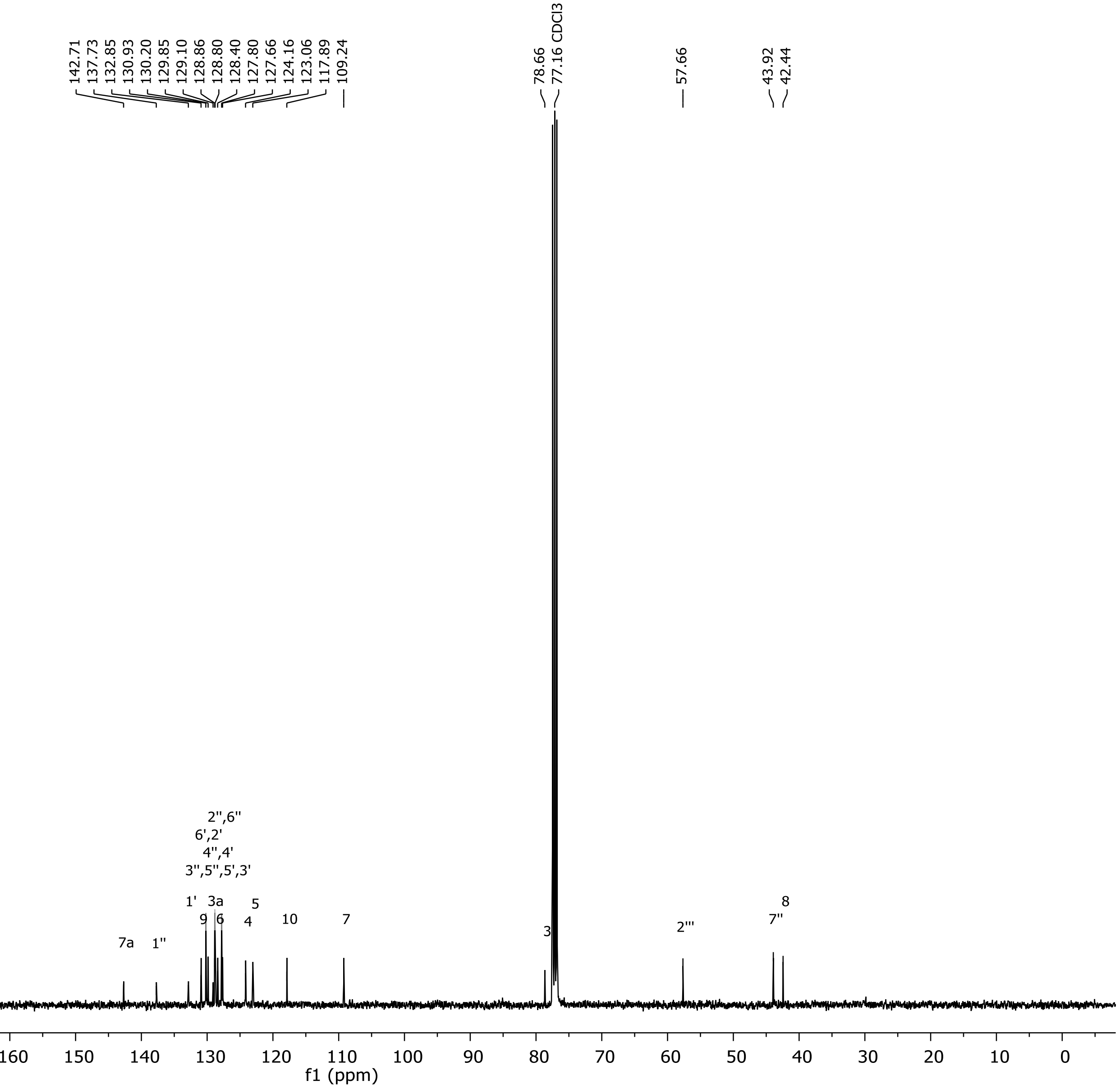

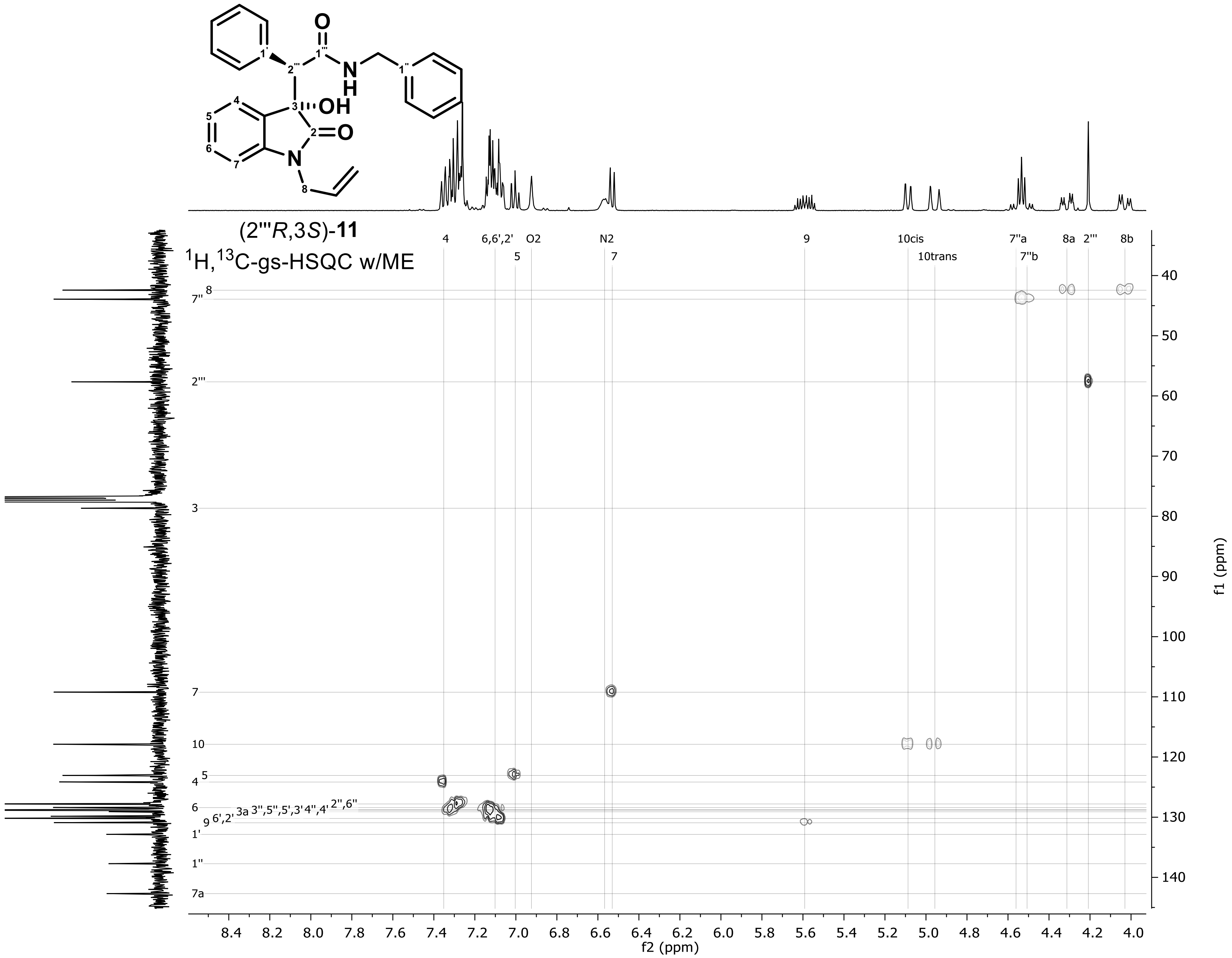

(2'''*R*,3*S*)-**11**

$^{1}H,^{13}C$-gs-HSQC w/ME

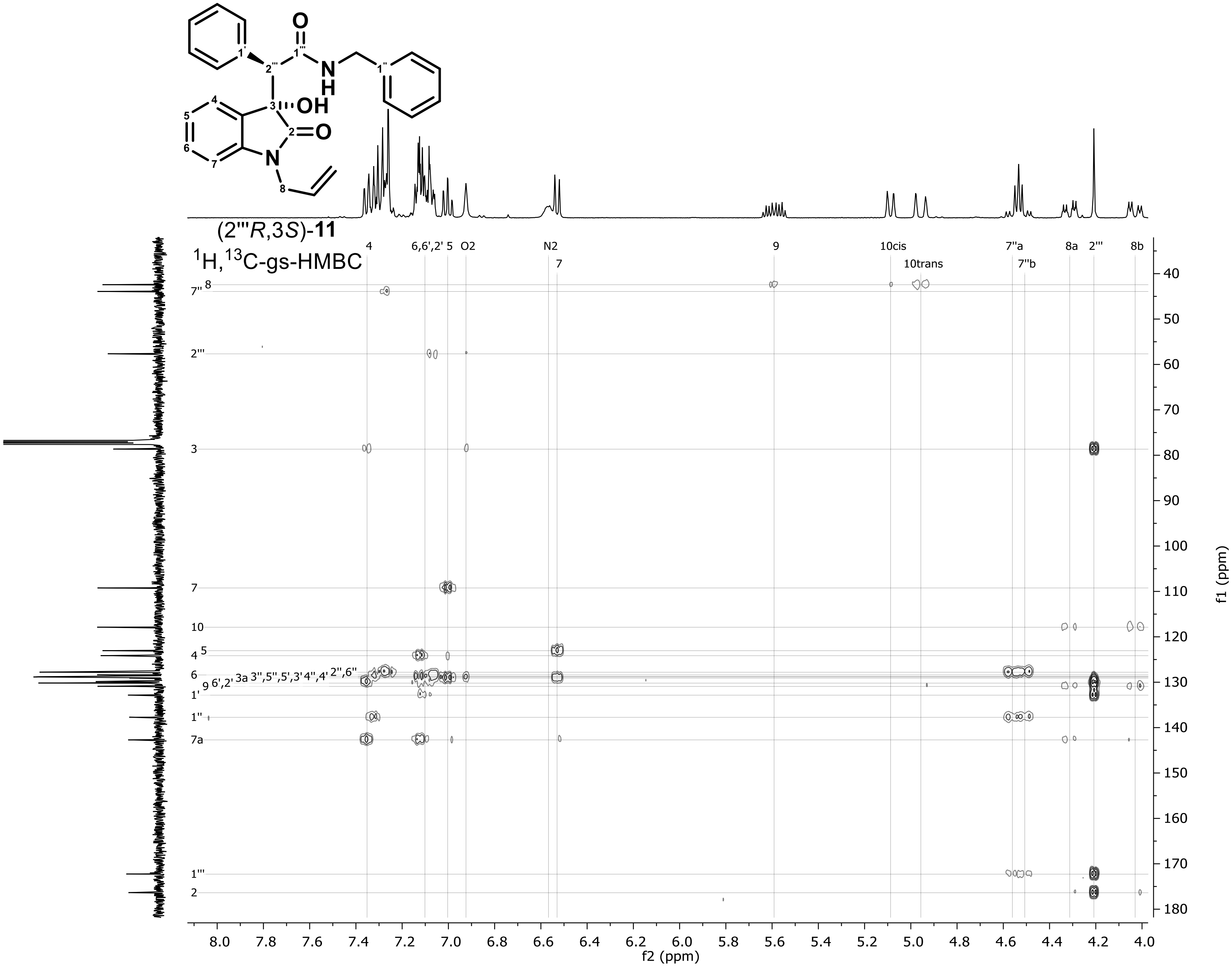
(2'''R,3S)-11
1H,13C-gs-HMBC
4
6,6',2' 5
O2
N2
7
9
10cis
10trans
7''a
7''b
8a
2'''
8b
7''
8
2'''
3
7
10
4
5
6
9
6',2'
3a
3'',5'',5',3'
4'',4'
2'',6''
1'
1''
7a
1'''
2
f1 (ppm)
f2 (ppm)

f) ***N*,*N*'-dibenzyl 2-(*N*-(*tert*-butyloxycarbonyl)anilin-2-yl)-2-hydroxy-3-phenylsuccinamide (12)**

To a 25 ml round bottomed flask was added phenylacetic anhydride (95.3 mg, 0.375 mmol), *N*-Boc isatin (61.8 mg, 0.250 mmol), and (2*S*,3*R*)-HyperBTM (3.9 mg, 0.012 mmol). The mixture was cooled to 0 °C and CH_2Cl_2 (6.0 ml, 0.04 M) and Hünig's base (54 µl, 0.312 mmol) were added. The mixture was stirred at 0 °C for 3 h. Benzylamine (82 µl, 0.750 mmol) was added and the reaction was left to be stirred overnight at room temperature. 1,3,5-trimethoxybenzene (0.1 M soln in CH_2Cl_2 500 0µl, 0.05 mmol) was added and the solvent was removed under reduced pressure. Purification by column chromatography (Hexane:EtOAc 9:1 → 3:2) gave the title compound as yellow tinted solid (91.0 mg, 0.193 mmol, 77%).

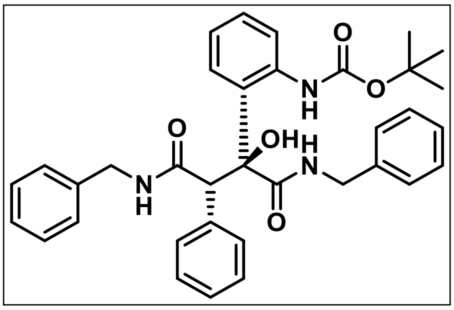

R_f 0.61 (Hexane:EtOAc 1:1); yellow (vanillin, heat); **Chiral HPLC** analysis (Chiralcel® IA 95:5 Hexane : IPA, flow rate 2.0 ml·min^{-1}, 211 nm, 40 °C) t_R (2*S*,3*S*)-**12**: 23.8 min, t_R (2*R*,3*S*)-**12**, 28.1 min, >99:1 e.r.; $\alpha_D^{20} = -127.0$ (*c* 2.27, $CHCl_3$); **ν_{max}** (thin film) 3397 (w), 3316 (m), 3088 (w), 3065 (w), 3030 (w), 2978 (w), 2928 (w), 2249 (w), 1717 (m), 1651 (m), 1639 (m), 1587 (m), 1522 (s), 1497 (m), 1452 (w), 1441 (s), 1391 (w), 1366 (m), 1308 (m), 1231 (s), 1157 (s), 1121 (w), 1045 (m), 1026 (m), 961 (w), 908 (s), 831 (w), 754 (m); **^{1}H NMR** (400 MHz, $CDCl_3$) δ_H 9.15 (1H, s(br), N*H*), 8.01 (1H, s, N*H*), 7.99 (1H, s, N*H*), 7.39 – 7.11 (16H, m, PhC$^{2',3',4',5',6'}$*H*, ArC$^{3'',4''}$*H*, PhC$^{3''',4''',5'''}$*H*, PhC$^{2'''',3'''',4'''',5'''',6''''}$*H*, O*H*), 7.54 (1H, d, $^3J_{HH}$ = 8.0 Hz, ArC$^{6''}$*H*), 7.05 (2H, app d, $^3J_{HH}$ = 7.4 Hz, PhC$^{2''',6'''}$*H*), 6.86 (1H, dd, $^3J_{HH}$ = 8.0 Hz, 7.2 Hz, ArC$^{5''}$*H*), 4.73 (1H, s(br), PhC$^{1'''}$C*H*), 4.50 (1H, dd, $^2J_{HH}$ = 15.2 Hz, $^3J_{HH}$ = 6.1 Hz, PhC$^{1'}$C*H*$_a$H$_b$NH), 4.47 (1H, dd, $^2J_{HH}$ = 15.1 Hz, $^3J_{HH}$ = 6.2 Hz, PhC$^{1''''}$C*H*$_a$H$_b$NH), 4.35 (1H, dd, $^2J_{HH}$ = 15.2 Hz, $^3J_{HH}$ = 5.5 Hz, PhC$^{1'}$CH$_a$*H*$_b$NH), 4.25 (1H, dd, $^2J_{HH}$ = 15.1 Hz, $^3J_{HH}$ = 5.4 Hz, PhC$^{1''''}$CH$_a$*H*$_b$NH), 1.46 (9H, s, OC(C*H*$_3$)$_3$); **^{1}H NMR** (500 MHz, $C_6D_5CD_3$, 373 K) δ_H 9.49 (1H, s, N*H*), 8.31 (1H, d, $^3J_{HH}$ = 7.7 Hz, ArC$^{3''}$*H*), 7.88 (1H, s, N*H*), 7.68 (1H, d, $^3J_{HH}$ = 8.0 Hz, ArC$^{6''}$*H*), 7.12 (2H, app d, $^3J_{HH}$ = 6.84 Hz, PhC$^{2''',6'''}$*H*), 7.07 – 6.86 (15H, m, N*H*, PhC$^{2',3',4',5',6'}$*H*, ArC$^{4''}$*H*, PhC$^{3''',4''',5'''}$*H*, PhC$^{2'''',3'''',4'''',5'''',6''''}$*H*), 6.78 (1H, dd, $^3J_{HH}$ = 8.0 Hz, 7.7 Hz, ArC$^{5''}$*H*), 5.80 (1H, s(br), O*H*), 4.70 (1H, s(br), PhC$^{1'''}$C*H*), 4.27 (1H, dd, $^2J_{HH}$ = 15.3 Hz, $^3J_{HH}$ = 6.9 Hz, PhC$^{1''''}$C*H*$_a$H$_b$NH), 4.17 (1H, dd, $^2J_{HH}$ = 15.4 Hz, $^3J_{HH}$ = 6.2 Hz, PhC$^{1'}$C*H*$_a$H$_b$NH), 4.03 (1H, dd, $^2J_{HH}$ = 15.4 Hz, $^3J_{HH}$ = 6.3 Hz, PhC$^{1'}$CH$_a$*H*$_b$NH), 3.92 (1H, dd, $^2J_{HH}$ = 15.3 Hz, $^3J_{HH}$ = 5.5 Hz, PhC$^{1''''}$CH$_a$*H*$_b$NH), 1.44 (9H, s, OC(C*H*$_3$)$_3$); **^{13}C {^{1}H} NMR** (101 MHz, $CDCl_3$, not all signals resolved) δ_C 175.2 (PhC$^{1'}$CH$_2$NH*C*(O)), 174.4 (Ph*C*$^{1''''}$CH$_2$NH*C*(O)), 152.5 (NH*C*(O)OC(CH$_3$)$_3$), 138.8 (Ar*C*$^{2''}$NHBoc, via HMBC), 137.6 (Ph*C*$^{1''''}$CH$_2$NH), 137.5 (Ph*C*$^{1'}$CH$_2$NH), 133.5 (Ph*C*$^{1'''}$CH), 129.3 (Ph*C*$^{2''',6'''}$H), 128.9 (Ar*C*$^{4''}$H), 128.7 and 128.4 (Ph*C*$^{3',5'}$H, Ph*C*$^{3''',5'''}$H and Ph*C*$^{3'''',5''''}$H), 128.0, 127.5$_4$ and 127.4$_9$ (Ph*C*$^{4'}$H, Ph*C*$^{4'''}$H and Ph*C*$^{4''''}$H), 127.4 (Ph*C*$^{2',6'}$H), 127.3 (Ph*C*$^{2'''',6''''}$H), 126.3 (Ar*C*$^{6''}$H), 122.0 (Ar*C*$^{5''}$H), 85.0 (PhC$^{1''}$*C*H, via HMBC), 79.4 (O*C*(CH$_3$)$_3$), 55.8 (ArC$^{1''}$*C*(OH), via HMBC), 43.6 (PhC$^{1''''}$*C*H$_2$NH), 43.5 (PhC$^{1'}$*C*H$_2$NH), 28.5 (OC(*C*H$_3$)$_3$); **^{13}C {^{1}H} NMR** (126 MHz, deptq, $C_6D_5CD_3$, 368 K, not all signals resolved or obscured by solvent residue signals) δ_C 175.2 (–, PhC$^{1'}$CH$_2$NH*C*(O)), 174.4 (–, Ph*C*$^{1''''}$CH$_2$NH*C*(O)), 152.9 (–, NH*C*(O)OC(CH$_3$)$_3$), 139.5 (–, Ar*C*$^{2''}$NHBoc), 138.8 (–, Ph*C*$^{1''''}$CH$_2$NH), 138.4 (–, Ph*C*$^{1'}$CH$_2$NH), 134.8 (–, Ph*C*$^{1'''}$CH), 129.9 (+, Ph*C*$^{2''',6'''}$H), 129.3 (+), 129.0 (+, Ar*C*$^{4''}$H), 128.7$_5$, 128.7$_7$ and 128.7$_8$ (subtracted from solvent residue signal, various), 128.4 (+, Ph*C*^{3,5}H), 128.1 (+, Ph*C*^{4}H, subtracted from solvent residue signal), 127.7 (+, Ph*C*$^{2',6'}$H), 127.5 (+, Ph*C*$^{2'''',6''''}$H), 127.4 (+, Ph*C*^{4}H), 127.1 (+, Ar*C*$^{6''}$H), 125.5 (–, ArC1''), 122.0 (+, Ar*C*$^{5''}$H), 121.8 (+, Ar*C*$^{3''}$H), 84.6 (PhC$^{1''}$*C*H, via HMBC), 79.4 (–, O*C*(CH$_3$)$_3$), 56.1 (+, ArC$^{1''}$*C*(OH)), 43.7 (–, PhC$^{1'}$*C*H$_2$NH, PhC$^{1''''}$*C*H$_2$NH), 28.8 (+, OC(*C*H$_3$)$_3$); ***m/z*** (ESI$^+$) 141 (4%), 242 (47%), 602 ([M+Na]$^+$ 38%), 618 ([M+K]$^+$ 100%), 619 ([M(^{13}C)+K]$^+$ 39%), 620 ([M(^{13}C$_2$)+K]$^+$ 12%); **HRMS** (ESI$^+$) $C_{25}H_{37}O_5N_3Na$ [M+Na]$^+$ found 602.2622, requires 602.2625 (−0.5 ppm); **HRMS** (ESI$^+$) *m/z* calcd for [M+K]$^+$ $C_{25}H_{37}O_5N_3K$ 618.2365, found 618.2358 (−1.1 ppm).

(±)-anti-**12**

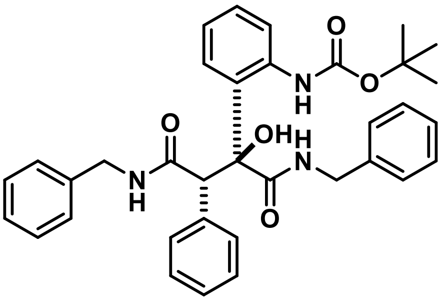

PDA Ch1 211nm

Peak#	Ret. Time	Area%
1	23.777	49.681
2	28.106	50.319
Total		100.000

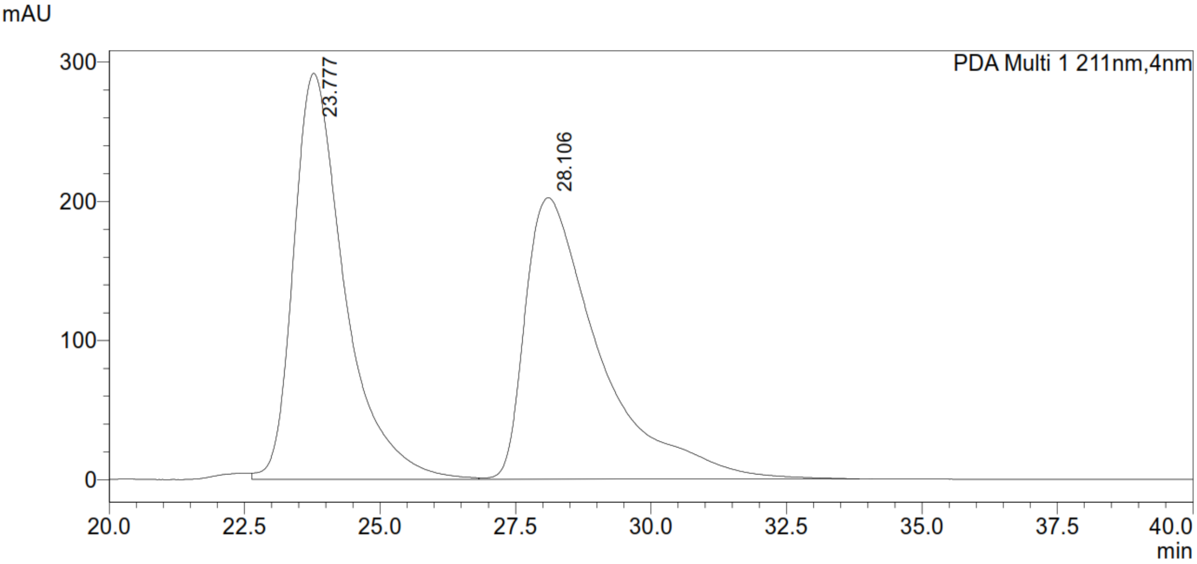

(2*S*,3*S*)-anti-**12**

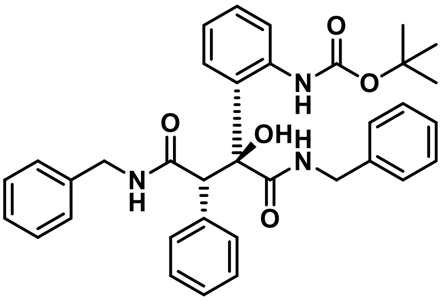

PDA Ch1 211nm

Peak#	Ret. Time	Area%
1	23.660	100.009
2	28.247	-0.009
Total		100.000

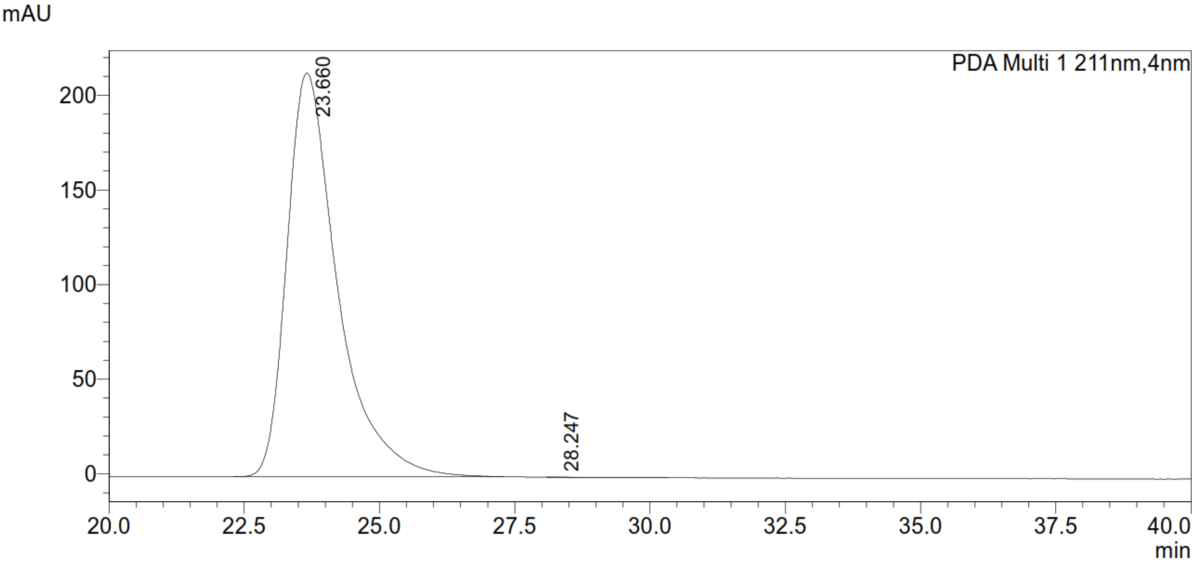

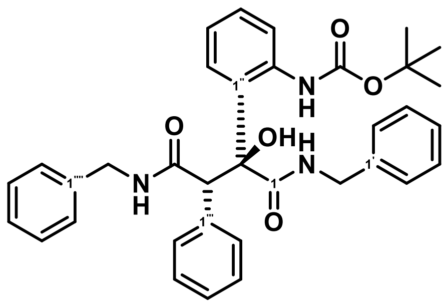

(2*S*,3*S*)-**12**
^{1}H NMR (400 MHz, $CDCl_3$)

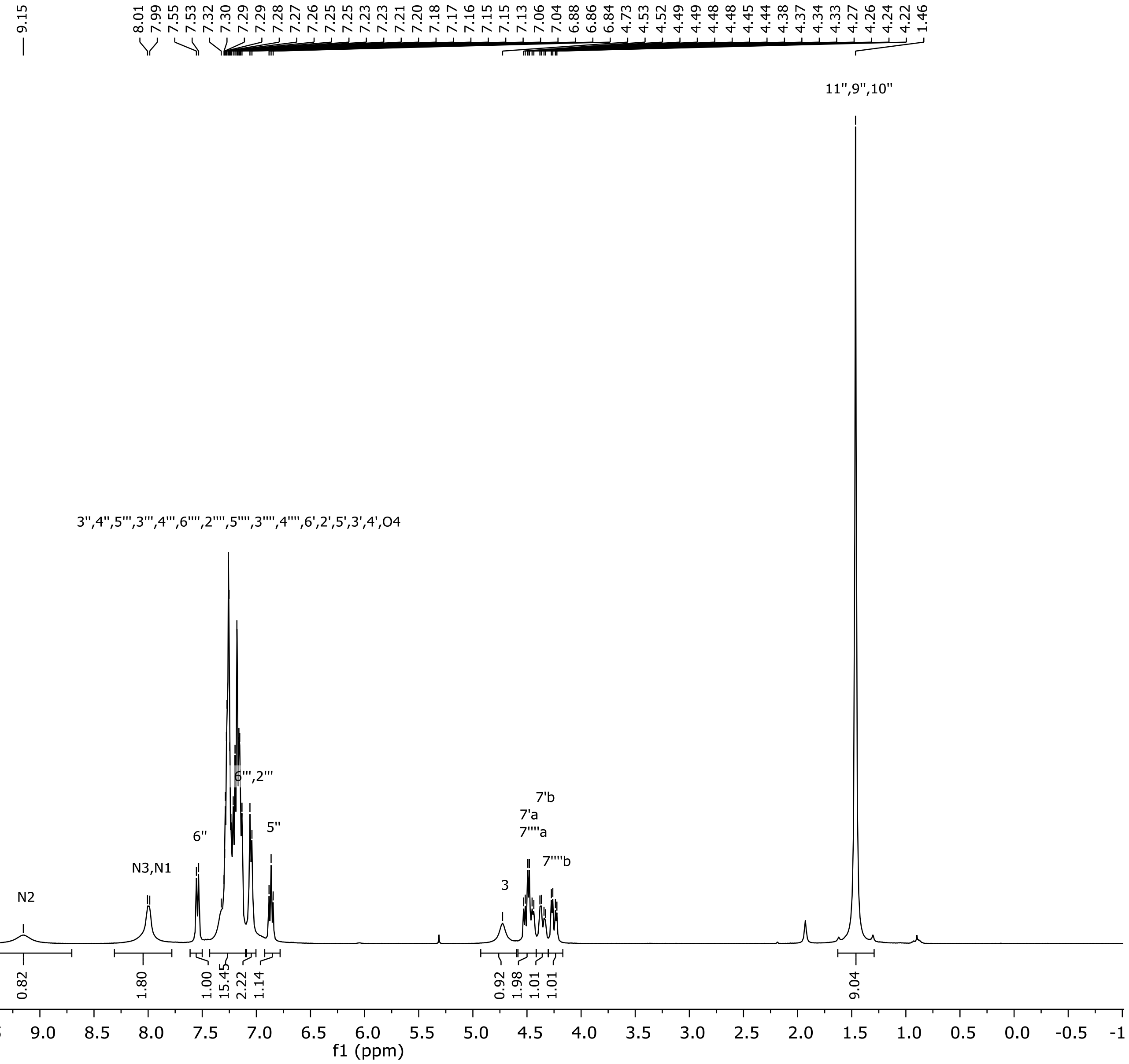

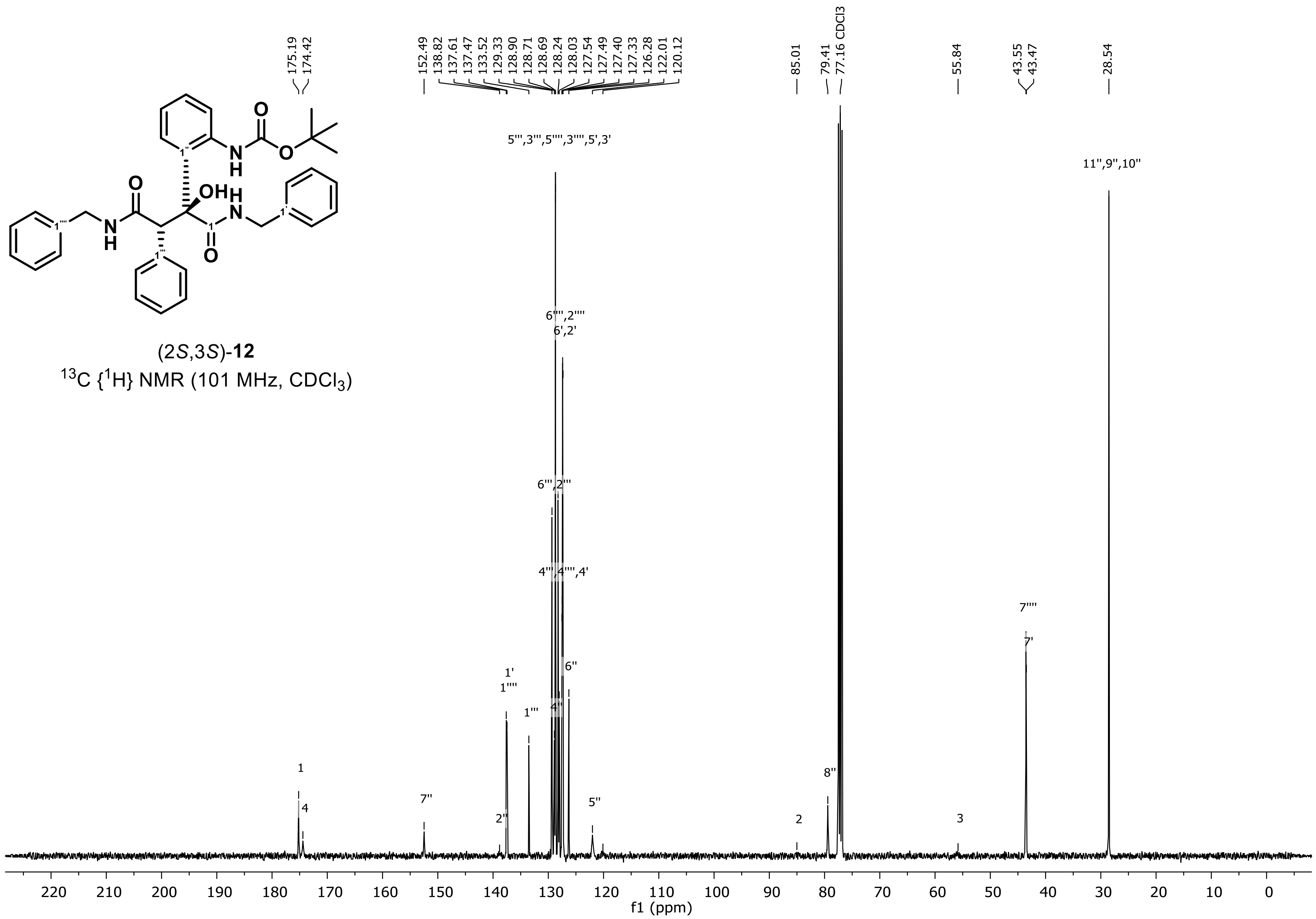

(2*S*,3*S*)-**12**

$^{13}C\{^{1}H\}$ NMR (101 MHz, $CDCl_3$)

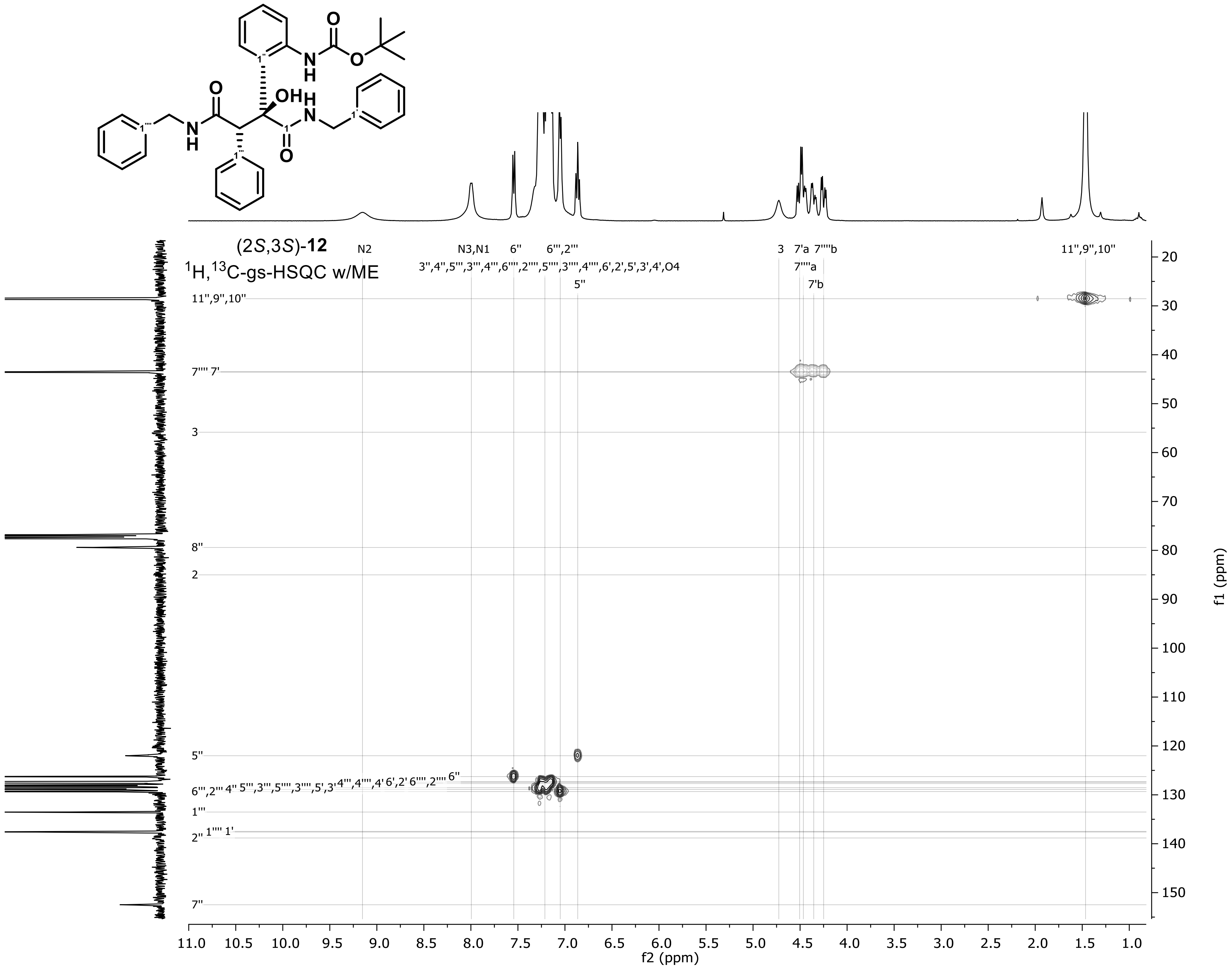

(2S,3S)-12
1H,13C-gs-HSQC w/ME
N2
N3,N1
6''
6''',2'''
3
7'a
7''''b
11'',9'',10''
3'',4'',5'',3''',4''',6'''',2'''',5'''',3'''',4'''',6',2',5',3',4',O4
5''
7''''a
7'b
7'''' 7'
8''
2
1'''
2'' 1'''' 1'
7''
f2 (ppm)
f1 (ppm)

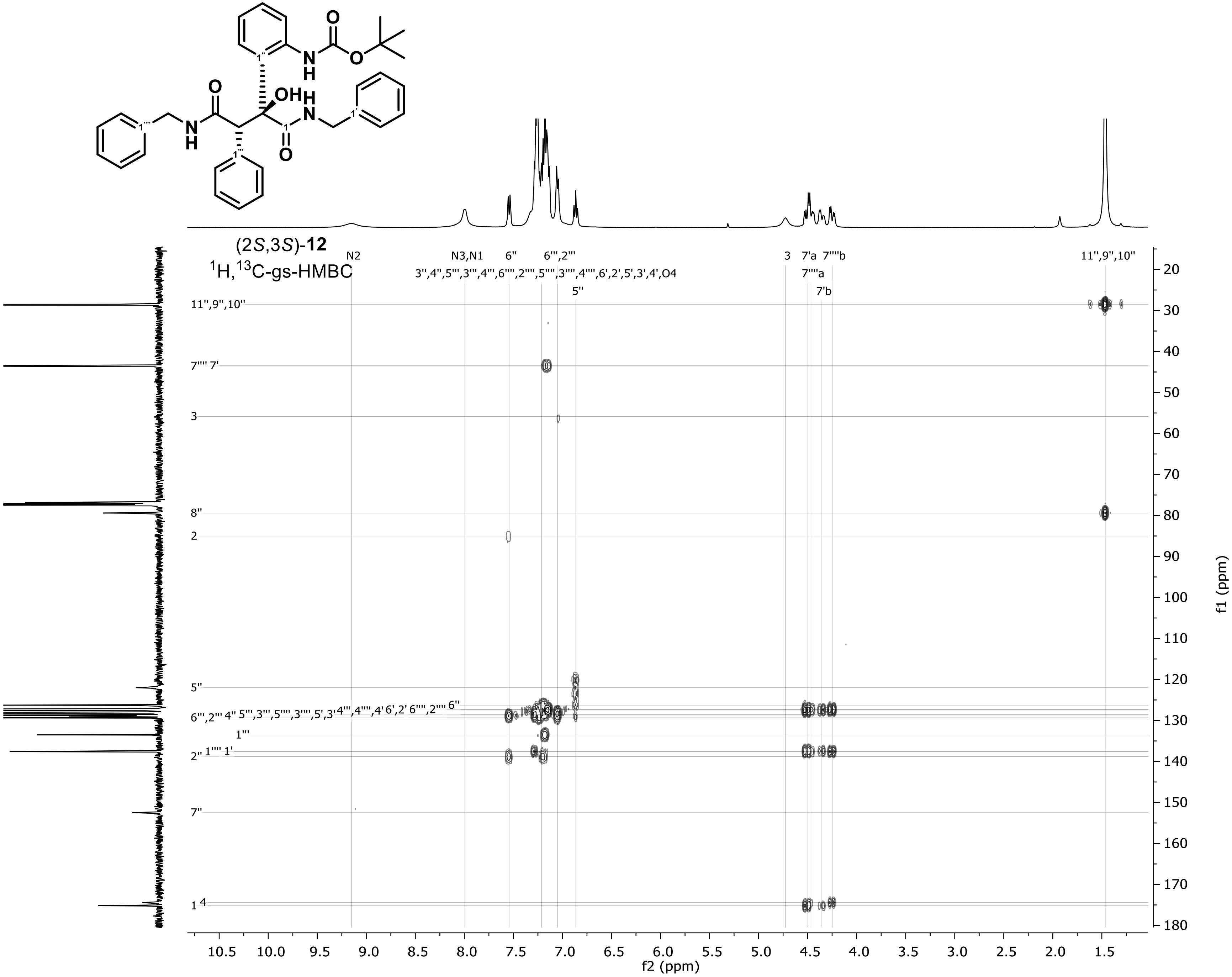

(2S,3S)-12
1H,13C-gs-HMBC
N2
N3,N1
6''
6''',2'''
3'',4'',5''',3''',4''',6'''',2'''',5'''',3'''',4'''',6',2',5',3',4',O4
5''
3
7'a
7''''b
7''''a
7'b
11'',9'',10''
7'''' 7'
8''
2
5''
6''',2''' 4'' 5''',3''',5'''',3'''',5',3' 4''',4'''',4' 6',2' 6'''',2'''' 6''
1'''
2'' 1'''' 1'
7''
1 4
f1 (ppm)
f2 (ppm)

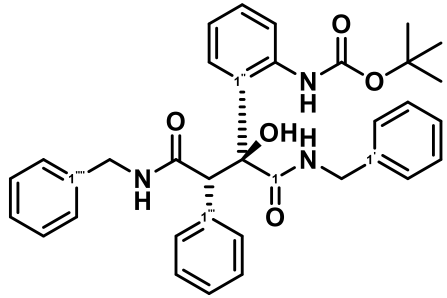

(2*S*,3*S*)-**12**

^{1}H NOESY (295 K)

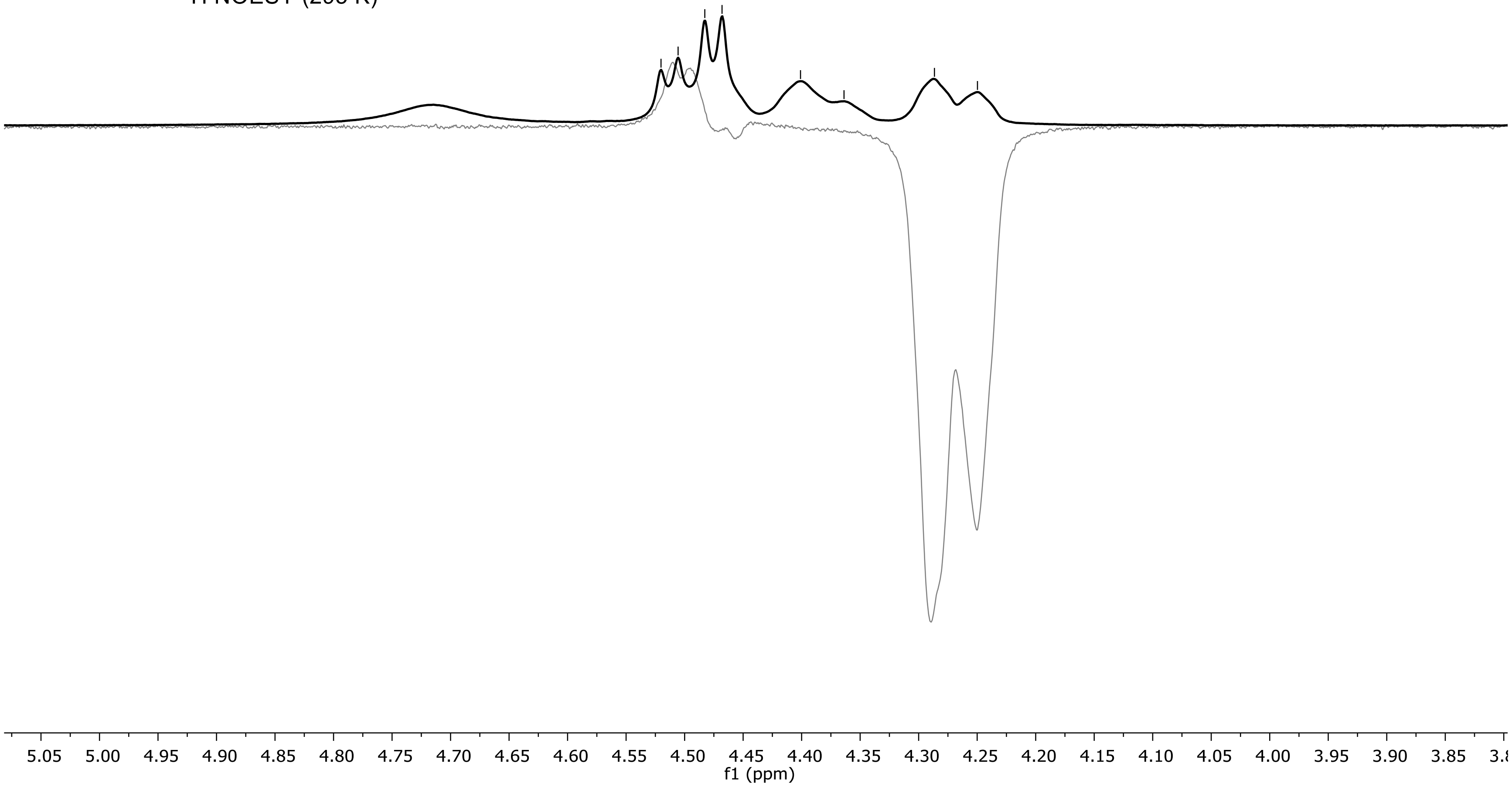

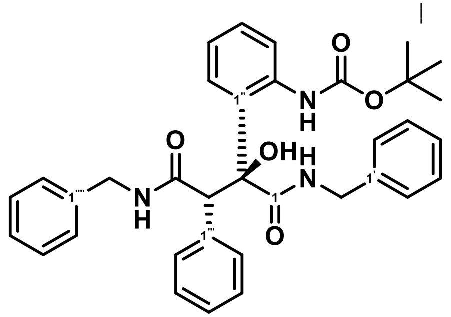

(2*S*,3*S*)-**12**

^{1}H NMR (500 MHz, $C_6D_5CD_3$, 373 K)

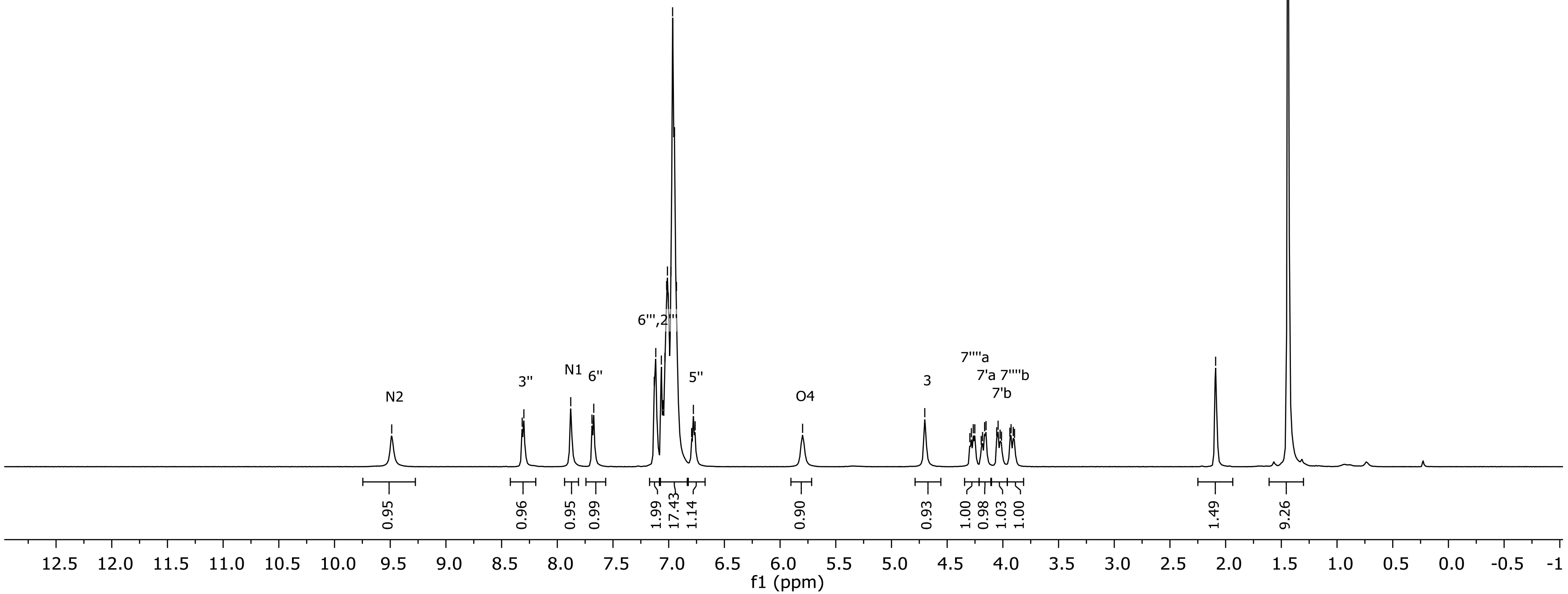

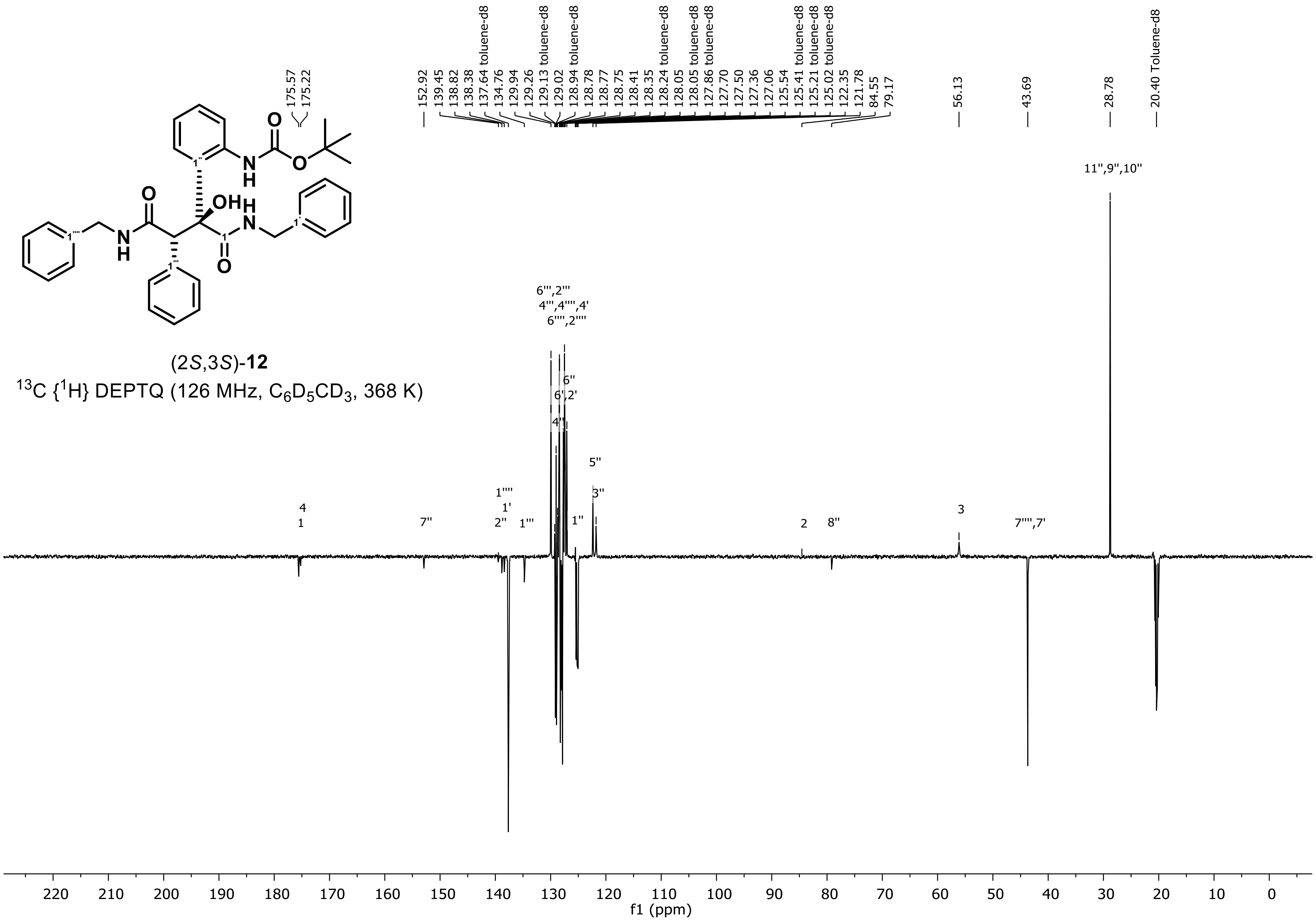

(2*S*,3*S*)-**12**

$^{13}C\ \{^{1}H\}$ DEPTQ (126 MHz, $C_6D_5CD_3$, 368 K)

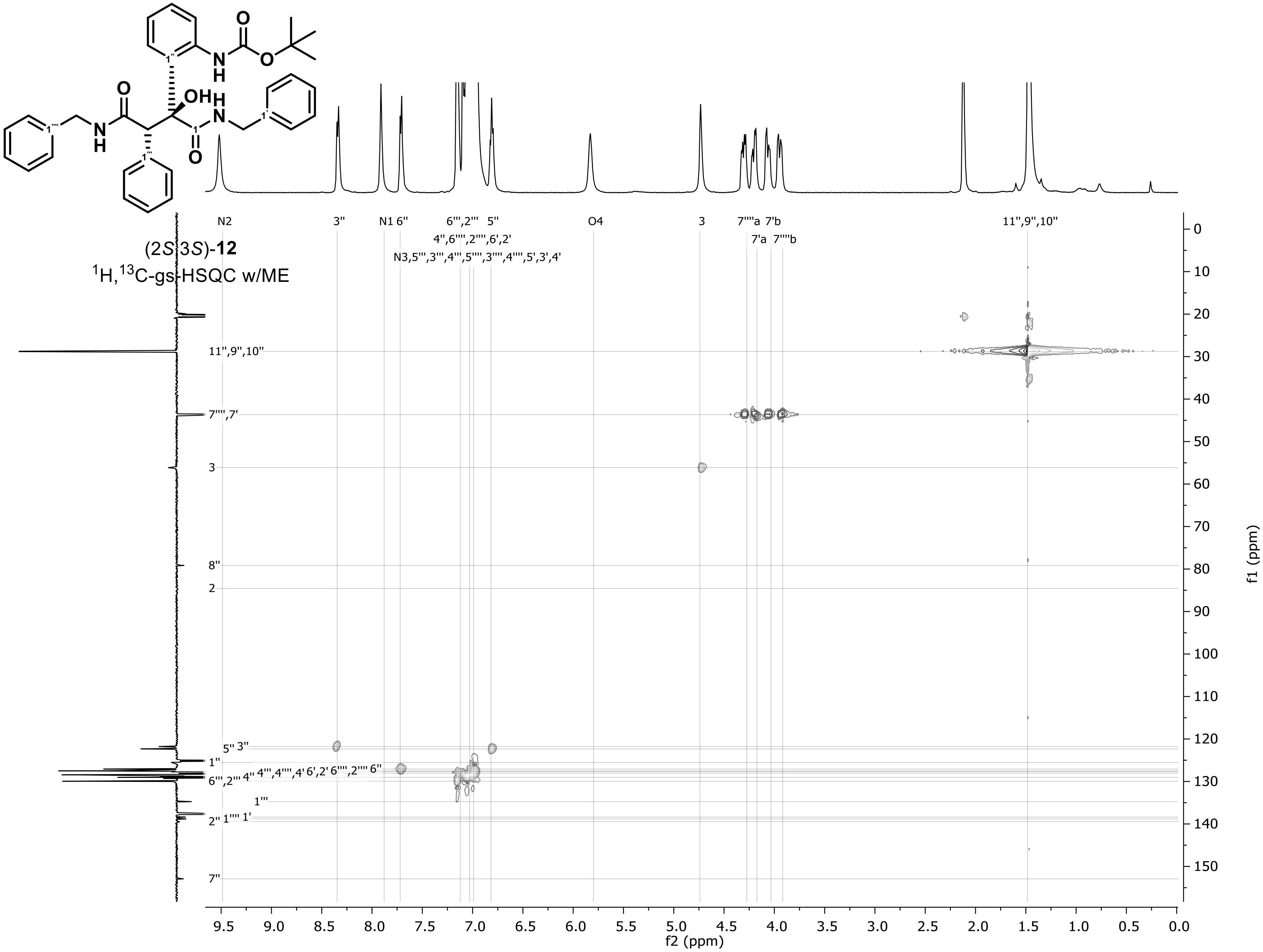
(2S,3S)-12
1H,13C-gs-HSQC w/ME
N2
3''
N1 6''
6''',2'''
5''
4'',6'''',2'''',6',2'
N3,5''',3''',4''',5'''',3'''',4'''',5',3',4'
O4
3
7''''a 7'b
7'a 7''''b
11'',9'',10''
7'''',7'
8''
2
5'' 3''
1''
6''',2''' 4'' 4''',4'''',4' 6',2' 6'''',2'''' 6''
1'''
2'' 1'''' 1'
7''
f2 (ppm)
f1 (ppm)

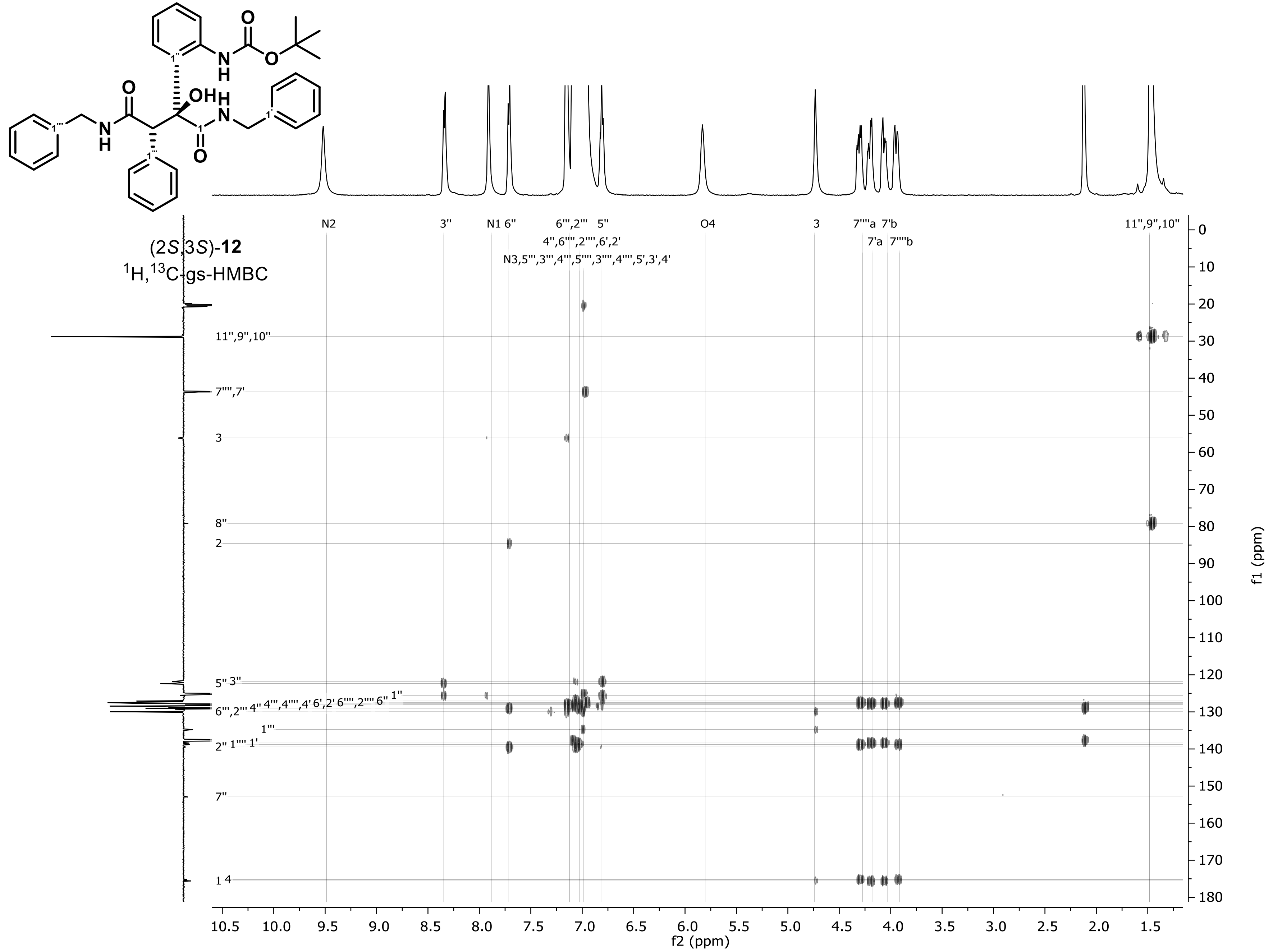

(2S,3S)-12
1H,13C-gs-HMBC
N2
3''
N1 6''
6''',2'''
5''
4'',6'''',2'''',6',2'
N3,5''',3''',4''',5'''',3'''',4'''',5',3',4'
O4
3
7''''a
7'b
7'a
7''''b
11'',9'',10''
7'''',7'
8''
2
5'' 3''
6''',2''' 4'' 4''',4'''',4' 6',2' 6'''',2'''' 6'' 1''
1'''
2'' 1'''' 1'
7''
1 4
f2 (ppm)
f1 (ppm)

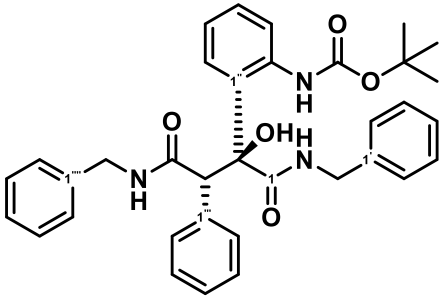

(2*S*,3*S*)-**12**
[1]H NOESY (373 K)

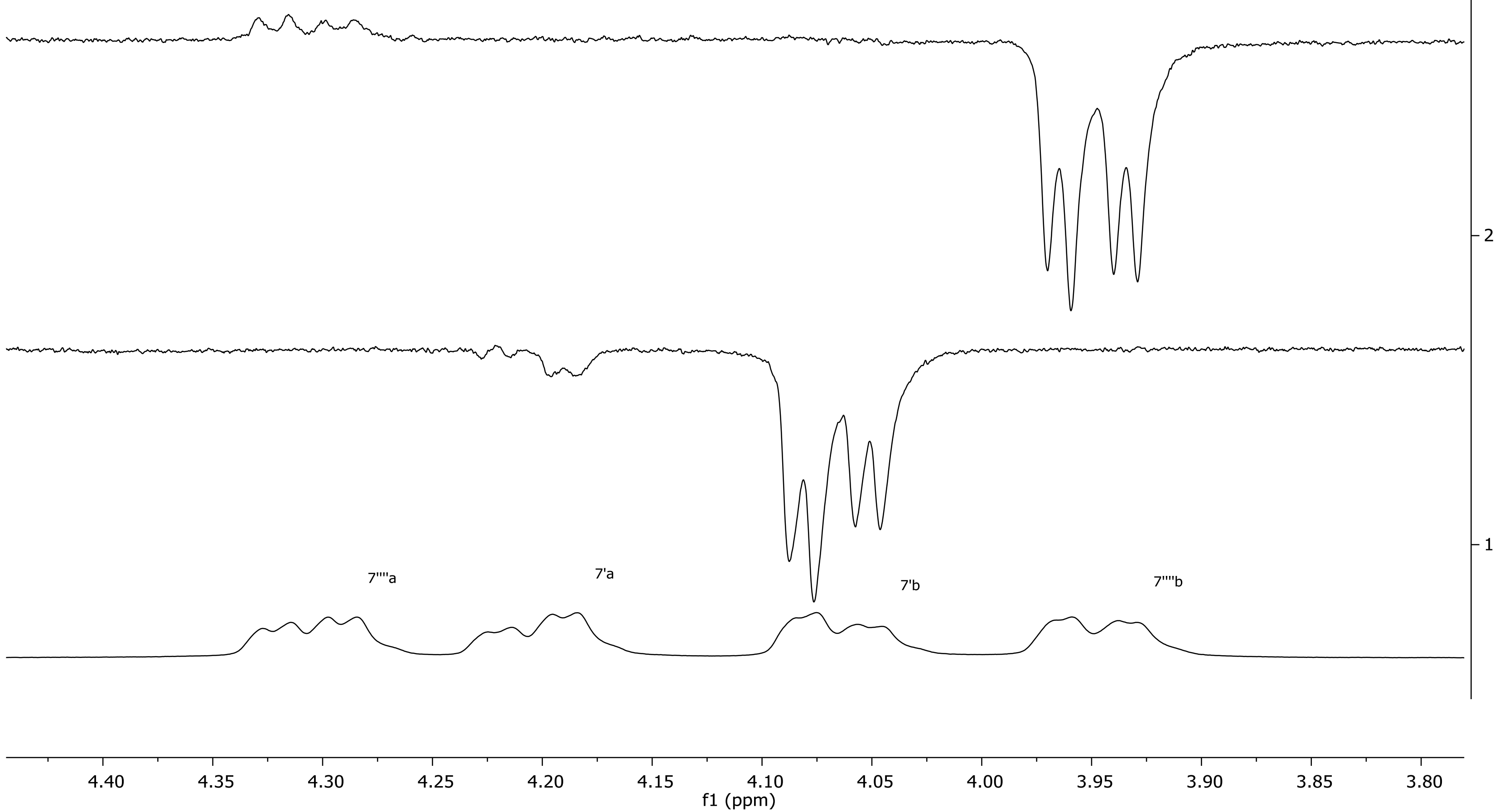

g) N,N′-dibenzyl-2-(3-hydroxy-2-oxo-7-trifluoromethylindolin-3-yl)-2-phenylacetamide (13)

To a 25 ml round bottomed flask was added phenylacetic anhydride (95.3 mg, 0.375 mmol), *N*-benzyl-7-trifluoromethylisatin (80.3 mg, 0.263 mmol), and (2*S*,3*R*)-HyperBTM (3.9 mg, 0.012 mmol). The mixture was cooled to 0 °C and CH_2Cl_2 (6.0 ml, 0.04 M) and Hünig's base (54 µl, 0.312 mmol) were added. The mixture was stirred at 0 °C for 3 h. Benzylamine (82 µl, 0.750 mmol) was added and the reaction was left to be stirred overnight at room temperature. 1,3,5-trimethoxybenzene (0.1 M soln in CH_2Cl_2, 500 µl, 0.05 mmol) was added and the solvent was removed under reduced pressure. Purification by column chromatography (Hexane:Et_2O 3:2 → Hexane:Acetone 9:1 → 3:2, recolumn in CH_2Cl_2:EtOAc 100% → 4:1) gave the title compound in two fractions (major diastereomer as off-white solid (102.9 mg) and minor diastereomer as white/brown semi-solid (30.7 mg); combined (133.6 mg, 0.252 mmol, 96%).

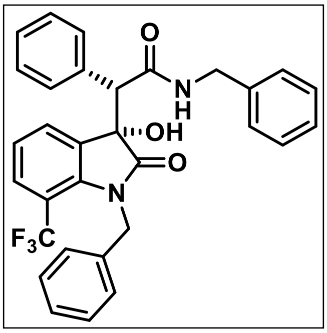

Major (2′′′′*S*,3*S*)-**13**: **m.p.** 144 °C; $\mathbf{R_f}$ 0.85 (Hexane:Acetone 7:3); **Chiral HPLC analysis** Chiralpak AD-H (85:15 hexane:IPA, flow rate 1 ml·min^{-1}, 211 nm, 40 °C) t_R (2′′′′*S*,3*S*)-**13**: 18.3 min, t_R (2′′′′*R*,3*R*)-**13**: 34.6 min, >99:1 e.r.; $\alpha_D^{20} = -0.406$ (*c* 1.54, $CHCl_3$); ν_{max} (thin film) 3343 (m, broad, OH, NH), 3090 (w), 3065 (w), 3032 (w), 2923 (w), 1732 (s, C=O, lactam), 1647 (m, C=O, amide), 1599 (m), 1541 (m), 1533 (m), 1497 (m), 1452 (s), 1439 (m), 1329 (s), 1279 (w), 1227 (w), 1182 (w), 1165 (s), 1125 (s), 1101 (s), 1086 (m), 1030 (w), 961 (w), 845 (w), 810 (w); **^{1}H NMR** (400 MHz, $CDCl_3$) δ_H 7.55 (2H, app d, $^3J_{HH}$ = 7.9 Hz, ArC^{4,6}*H*), 7.37 – 7.26 (4H, m, PhC$^{4''}$*H*, PhC$^{3''',4''',5'''}$*H*), 7.24 – 7.16 (4H, m, PhC$^{3'',5''}$*H*, PhC$^{2''',3'''}$*H*), 7.14 – 7.06 (4H, m, ArC^5*H*, PhC$^{3',4',5'}$*H*), 7.06 (1H, s, O*H*), 6.95 – 6.86 (2H, m, PhC$^{2'',6''}$*H*), 6.64 – 6.54 (2H, m, PhC$^{2',6'}$*H*), 5.95 (1H, t, $^3J_{HH}$ = 5.9 Hz, N*H*), 4.91 (1H, d, $^2J_{HH}$ = 17.1 Hz, NC*H*$_a$H$_b$-Ph), 4.83 (1H, d, $^2J_{HH}$ = 17.1 Hz, NCH$_a$*H*$_b$-Ph), 4.47 (2H, d, $^3J_{HH}$ = 5.9 Hz, NHC*H*$_2$-Ph), 4.16 (1H, s, C*H*-Ph); **^{13}C {^{1}H} NMR** (100 MHz, $CDCl_3$) δ_C 176.6 (*C*(O)NBn), 172.9 (*C*(O)NHBn), 141.5 (Ar*C*^{7a}), 137.4 (Ph*C*$^{1'''}$CH$_2$NH), 135.9 (Ph*C*$^{1'}$CH$_2$N), 132.3 (Ph*C*$^{1''}$CH), 131.9 (Ar*C*^{3a}), 130.4 (Ph*C*$^{2'',6''}$H), 129.6 (Ar*C*^{4}H), 129.1 (Ph*C*H), 128.9 (Ph*C*H), 128.4 (Ph*C*$^{3',5'}$H), 128.0 (q, $^3J_{CF}$ = 6.1 Hz, Ar*C*^{6}H), 127.9 (Ph*C*H), 127.7 (Ph*C*$^{2''',6'''}$H), 126.7 (Ph*C*$^{4'}$H), 125.4 (Ph*C*$^{2',6'}$H), 123.2 (q, $^1J_{CF}$ = 272.5 Hz, ArC^7*C*F$_3$), 122.6 (Ar*C*^{5}H), 113.1 (q, $^2J_{CF}$ = 33.1 Hz, Ar*C*^{7}CF$_3$), 77.0 (*C*-OH), 56.8 (*C*H-Ph), 45.9 (q, $^5J_{CF}$ = 4.9 Hz, N*C*H$_2$-Ph), 43.9 (NH*C*H$_2$-Ph); **^{19}F {^{1}H} NMR** (377 MHz, $CDCl_3$) δ_F −54.99 (3F, s, C*F*$_3$); ***m/z*** (ESI$^+$) 88 (16%), 110 (5%), 380 ([M-BnNHCOO]$^+$ 5%), 531 ([M+H]$^+$ 100%), 532 ([M(^{13}C)+H]$^+$ 35%), 533 ([M(^{13}C$_2$)+H]$^+$ 7%); **HRMS** (ESI$^+$) *m/z* calcd for [M+H]$^+$ $C_{31}H_{26}O_3N_2F_3$ 531.1890, found 531.1879 (−2.1 ppm).

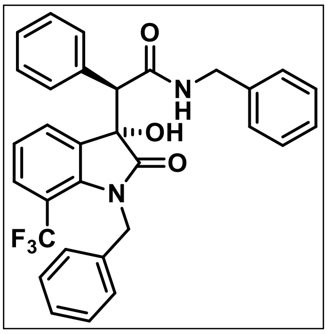

Minor (2′′′′*R*,3*S*)-**13**: $\mathbf{R_f}$ 0.79 (Hexane:Acetone 7:3); **Chiral HPLC analysis** Chiralpak AD-H (85:15 hexane:IPA, flow rate 1 ml·min^{-1}, 211 nm, 40 °C) t_R (2′′′′*R*,3*S*)-**13**: 15.2 min, t_R (2′′′′*S*,3*R*)-**13**: 17.0 min, 99:1 e.r.; $\alpha_D^{20} = -64.7$ (*c* 1.15, $CHCl_3$); ν_{max} (thin film) 3293 (m, broad, OH, NH), 3093(w), 3065 (w), 3032 (w), 2928 (w), 1717 (s, C=O, lactam), 1638 (s, C=O, amide), 1599 (s), 1497 (m), 1452 (s), 1439 (m), 1362 (m), 1329 (s), 1281 (w)m 1242 (w), 1213 (w), 1180 (w), 1163 (s), 1125 (s), 1101 (s), 1080 (m), 1028 (w), 1020 (w), 961 (w), 849 (w), 818 (w), 804 (w); **^{1}H NMR** (400 MHz, $CDCl_3$) δ_H 7.54 (1H, dd, $^3J_{HH}$ = 7.4 Hz, $^4J_{HH}$ = 1.3 Hz, ArC^4*H*), 7.43 (1H, s, O*H*), 7.42 (1H, dd, $^3J_{HH}$ = 8.4 Hz, $^4J_{HH}$ = 1.3 Hz, ArC^6*H*), 7.17 – 7.32 (9H, m, PhC$^{3',4',5'}$*H*, PhC$^{4''}$*H*, PhC$^{3''',4''',5'''}$*H*), 7.16 – 7.11 (2H, m, PhC$^{3'',5''}$*H*), 7.08 (1H, dd, $^3J_{HH}$ = 8.4 Hz, 7.4 Hz, ArC^5*H*), 7.05 – 6.98 (4H, m, PhC$^{2',6'}$*H*, PhC$^{2'',6''}$*H*), 6.35 (1H, t, $^3J_{HH}$ = 5.8 Hz, N*H*), 5.16 (1H, d, $^2J_{HH}$ = 16.9 Hz, NC*H*$_a$H$_b$-Ph), 4.86 (1H, d, $^2J_{HH}$ = 16.9 Hz, NCH$_a$*H*$_b$-Ph), 4.50 (1H, dd, $^2J_{HH}$ = 15.0 Hz, $^3J_{HH}$ = 5.8 Hz, NHC*H*$_a$H$_b$-Ph), 4.45 (1H, dd, $^2J_{HH}$ = 15.0 Hz, $^3J_{HH}$ = 5.8 Hz, NHCH$_a$*H*$_b$-Ph), 4.21 (1H, s, C*H*-Ph); **^{13}C{^{1}H} NMR** (100 MHz, $CDCl_3$) δ_C 178.1 (*C*(O)NBn), 172.1 (*C*(O)NHBn), 141.1 (Ar*C*^{7a}), 137.5 (Ph*C*$^{1'''}$), 135.9 (Ph*C*$^{1'}$), 132.1 (Ar*C*^{3a}), 132.2 (Ph*C*$^{1''}$), 130.0 (Ph*C*$^{2'',6''}$H), 129.1 (Ph*C*$^{3'',5''}$H), 128.8 (Ph*C*H), 128.7 (Ph*C*H), 128.4 (Ph*C*H), 128.1 (Ar*C*^{4}H), 127.9 (q, $^3J_{CF}$ = 6.2 Hz, Ar*C*^{6}H), 127.8 (Ph*C*H), 127.7 (Ph*C*H), 127.0 (Ph*C*$^{4'}$H), 126.1 (Ph*C*$^{2',6'}$H), 123.1 (ArC^7*C*F$_3$), 122.6 (Ar*C*^{5}H), 113.0 (q, $^2J_{CF}$ = 33.0 Hz,

Ar*C*^{7}CF$_3$), 57.2 (*C*H-Ph), 45.7 (q, $^5J_{CF}$ = 4.7 Hz, N*C*H$_2$-Ph), 43.9 (NH*C*H$_2$-Ph); **^{19}F{^{1}H} NMR** (377 MHz, CDCl$_3$) δ_F −55.18 (3F, s, C*F*$_3$); ***m/z*** (ESI$^+$) 91 (Bn$^+$ 72%), 143 (17%), 145 (17%), 170 (23%), 172 (22%), 198 (16%), 200 ([M-BnNHC(O)CH(OH)Ph-Bn+H 16%), 290 ([M-BnNHC(O)CH(OH)Ph+H]$^+$ 16%), 380 ([M-BnNHCOO]$^+$ 57%), 531 ([M+H]$^+$ 100%), 532 ([M(^{13}C)+H]$^+$ 33%), 533 ([M(^{13}C$_2$)+H]$^+$ 6%), 553 ([M+Na]$^+$ 65%), 554 ([M(^{13}C)+Na]$^+$ 35%); **HRMS** (ESI$^+$) *m/z* calcd for [M+H]$^+$ $C_{31}H_{26}O_3N_2F_3$ 531.1890, found 531.1890 (−1.1 ppm).

(±)-anti-**13** + (±)-syn-**13**

Detector A Channel 1 211nm

Peak#	Ret. Time	Area%
1	15.415	2.871
2	16.972	2.836
3	18.434	46.872
4	34.581	47.421
Total		100.000

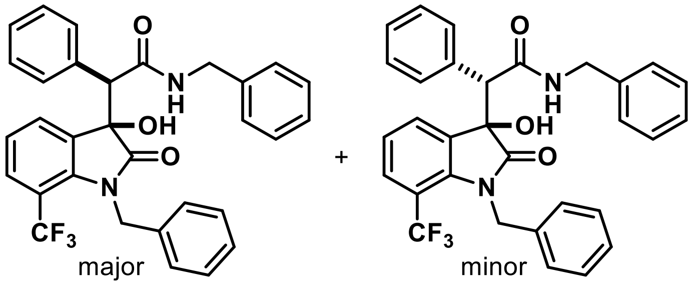

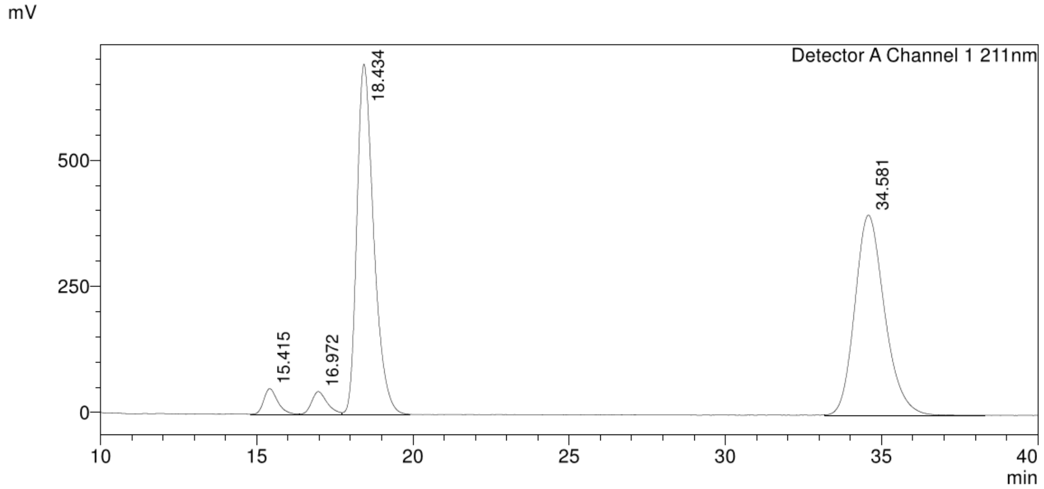

(2''''*S*,3*S*)-**15** + (2''''*R*,3*S*)-**15**

Detector A Channel 1 211nm

Peak#	Ret. Time	Area%
1	15.368	2.094
2	17.175	0.005
3	18.301	97.168
4	34.580	0.733
Total		100.000

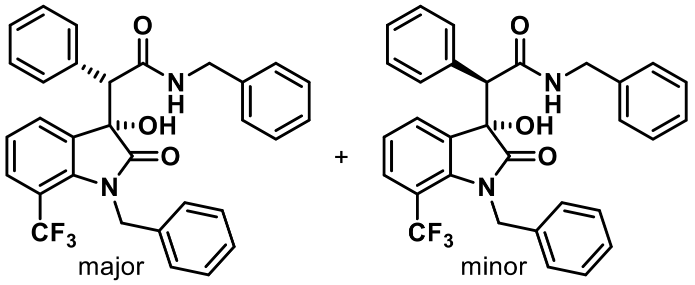

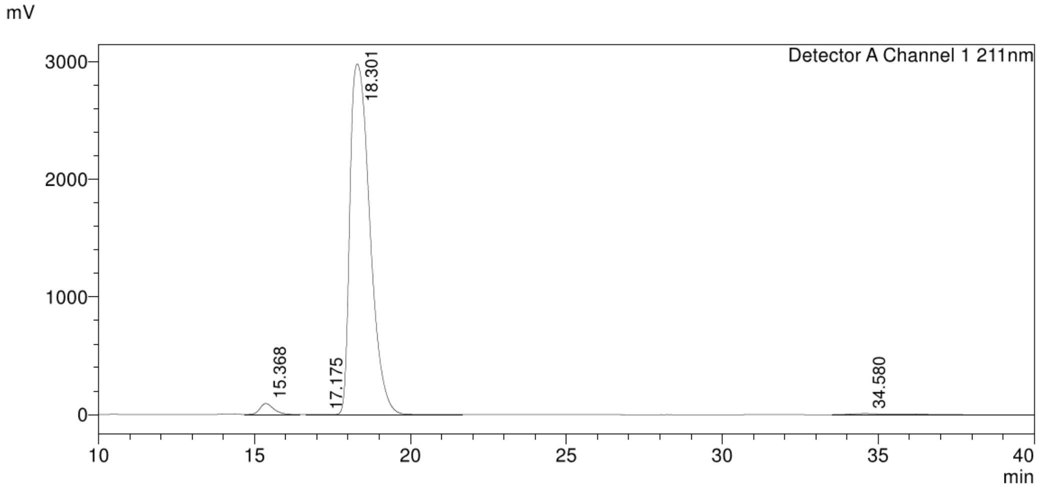

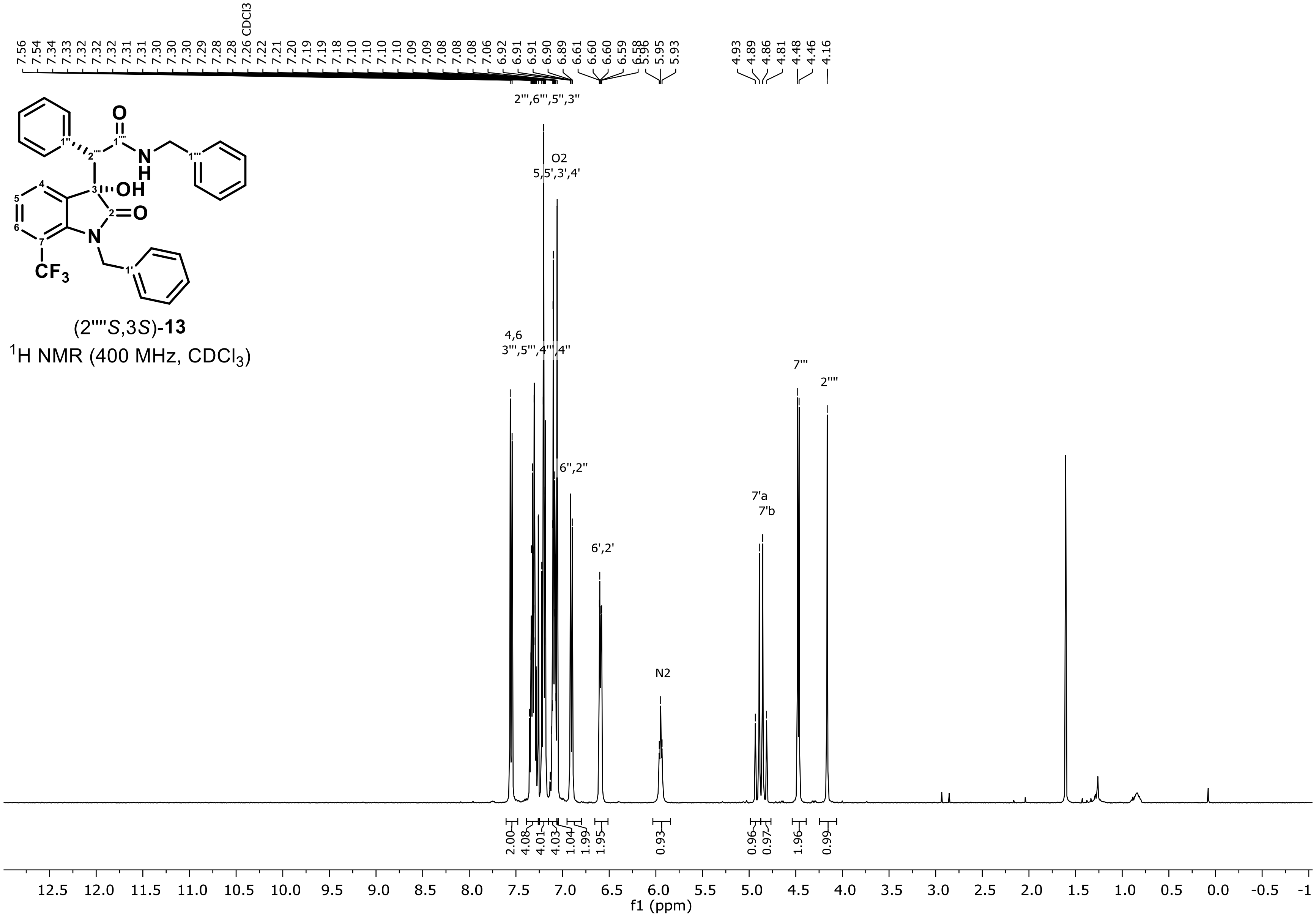
(2''''S,3S)-13
1H NMR (400 MHz, CDCl3)
f1 (ppm)

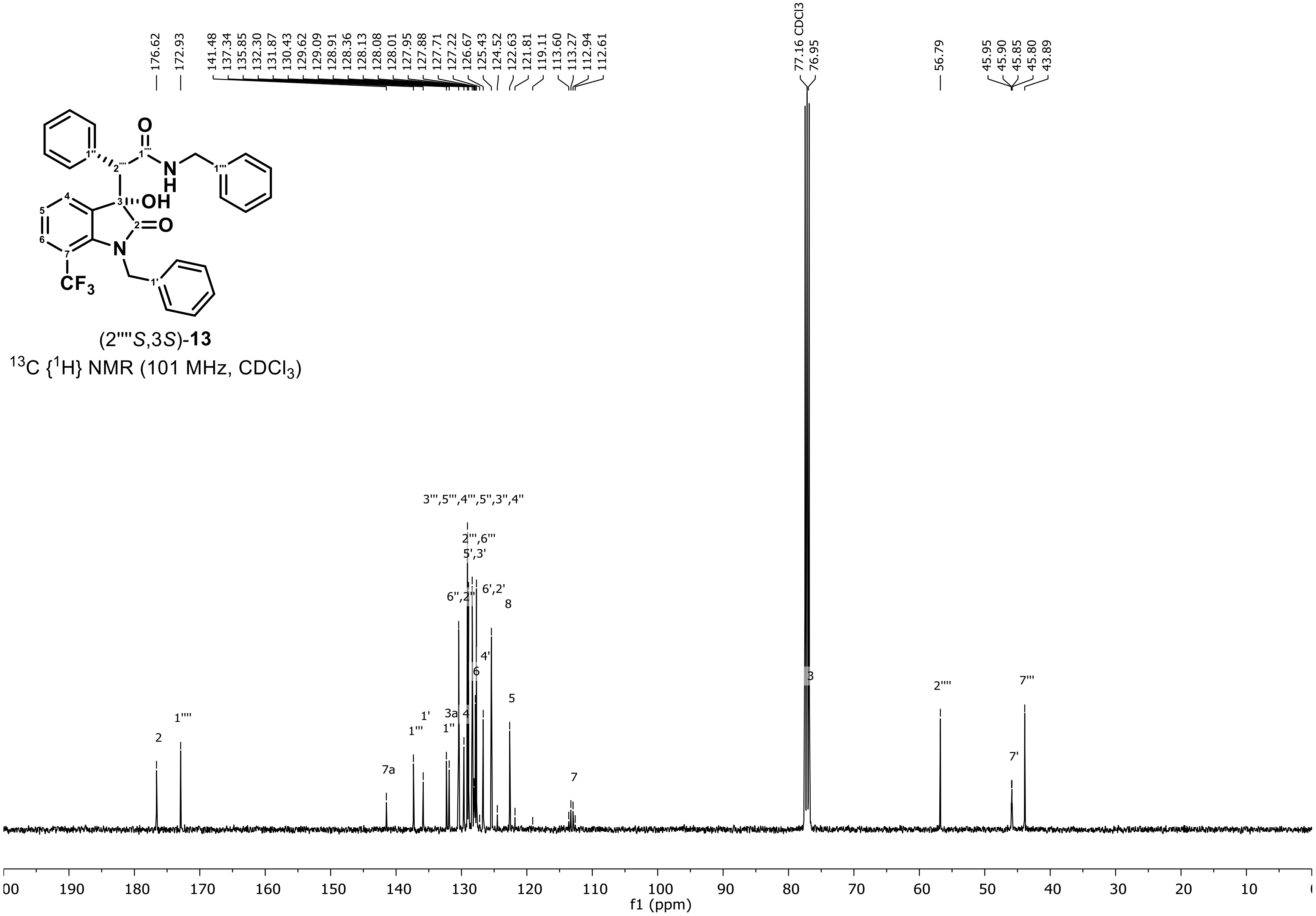

(2''''*S*,3*S*)-**13**

^{13}C {^{1}H} NMR (101 MHz, $CDCl_3$)

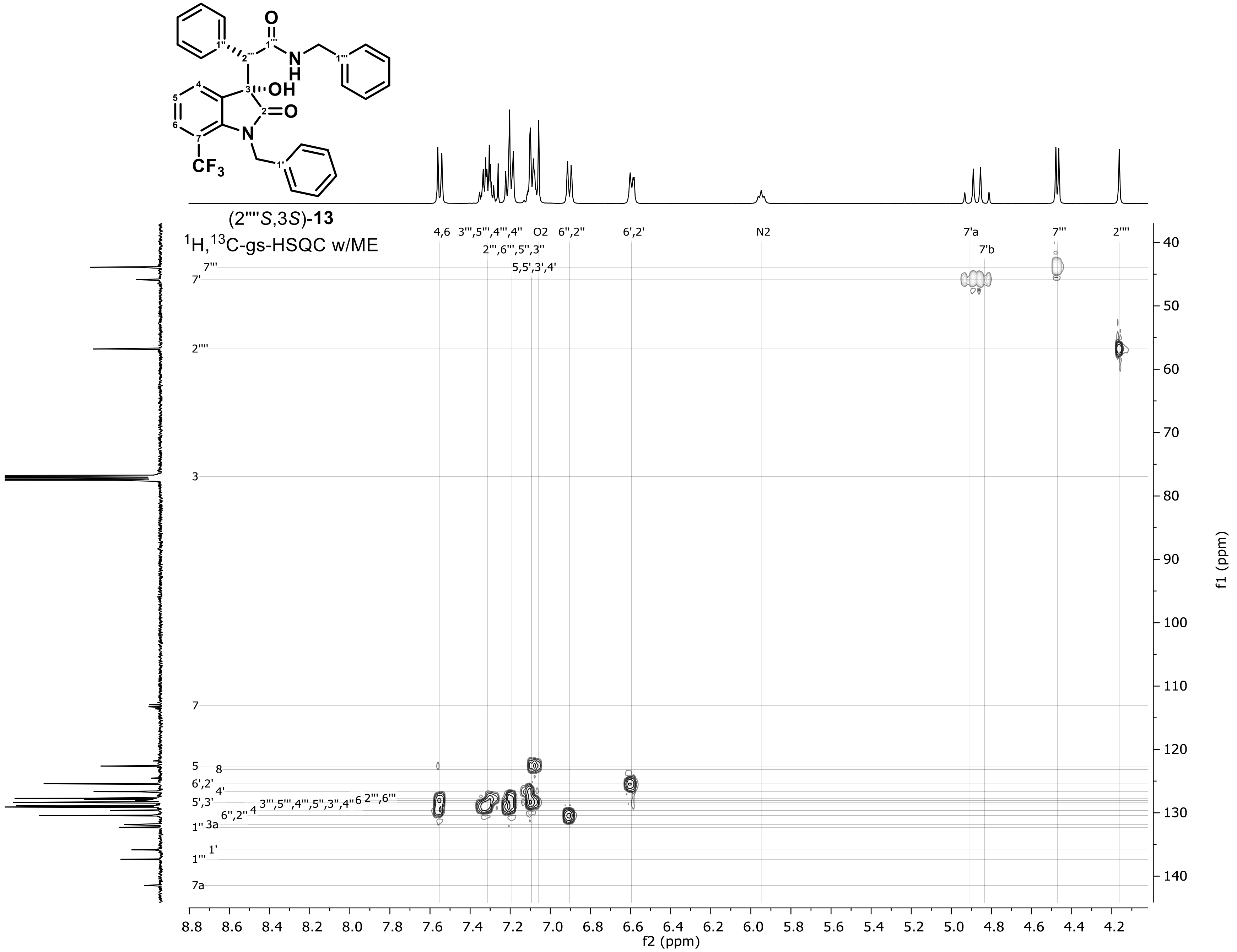
(2''''S,3S)-13
^{1}H,^{13}C-gs-HSQC w/ME
4,6
3''',5''',4''',4''
2''',6''',5'',3''
O2
5,5',3',4'
6'',2''
6',2'
N2
7'a
7'b
7'''
2''''
f2 (ppm)
f1 (ppm)

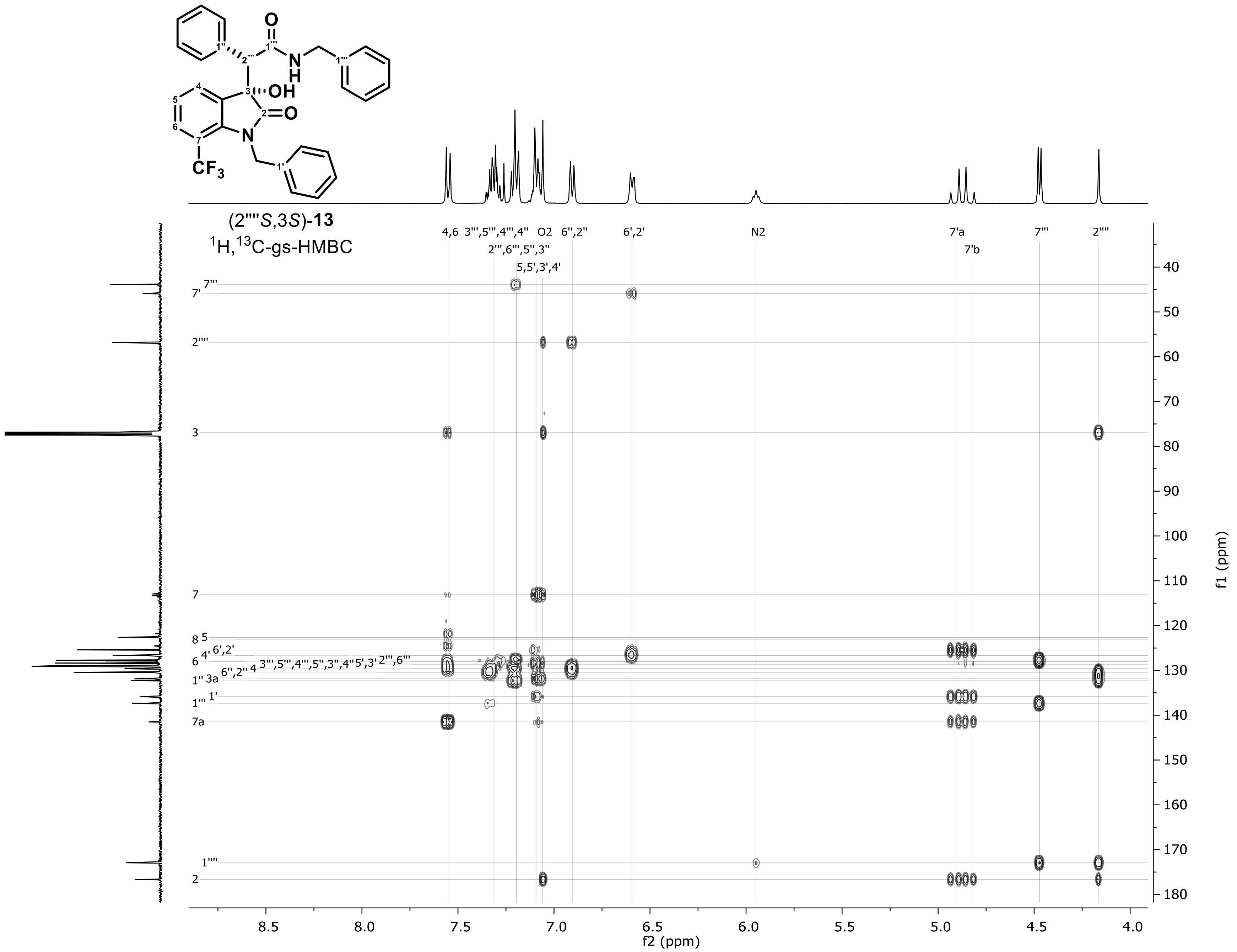

(2''''S,3S)-13
^{1}H,^{13}C-gs-HMBC
4,6
3''',5''',4''',4''
2''',6''',5'',3''
5,5',3',4'
O2
6'',2''
6',2'
N2
7'a
7'b
7'''
2''''
7'''
7'
2''''
3
7
8
5
4'
6',2'
6
1''
3a
6'',2''
4
3''',5''',4''',5'',3'',4''
5',3'
2''',6'''
1'''
1'
7a
1''''
2
f2 (ppm)
f1 (ppm)

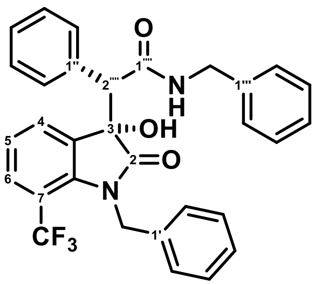

(2''''*S*,3*S*)-**13**

^{19}F {^{1}H} NMR (377 MHz, $CDCl_3$)

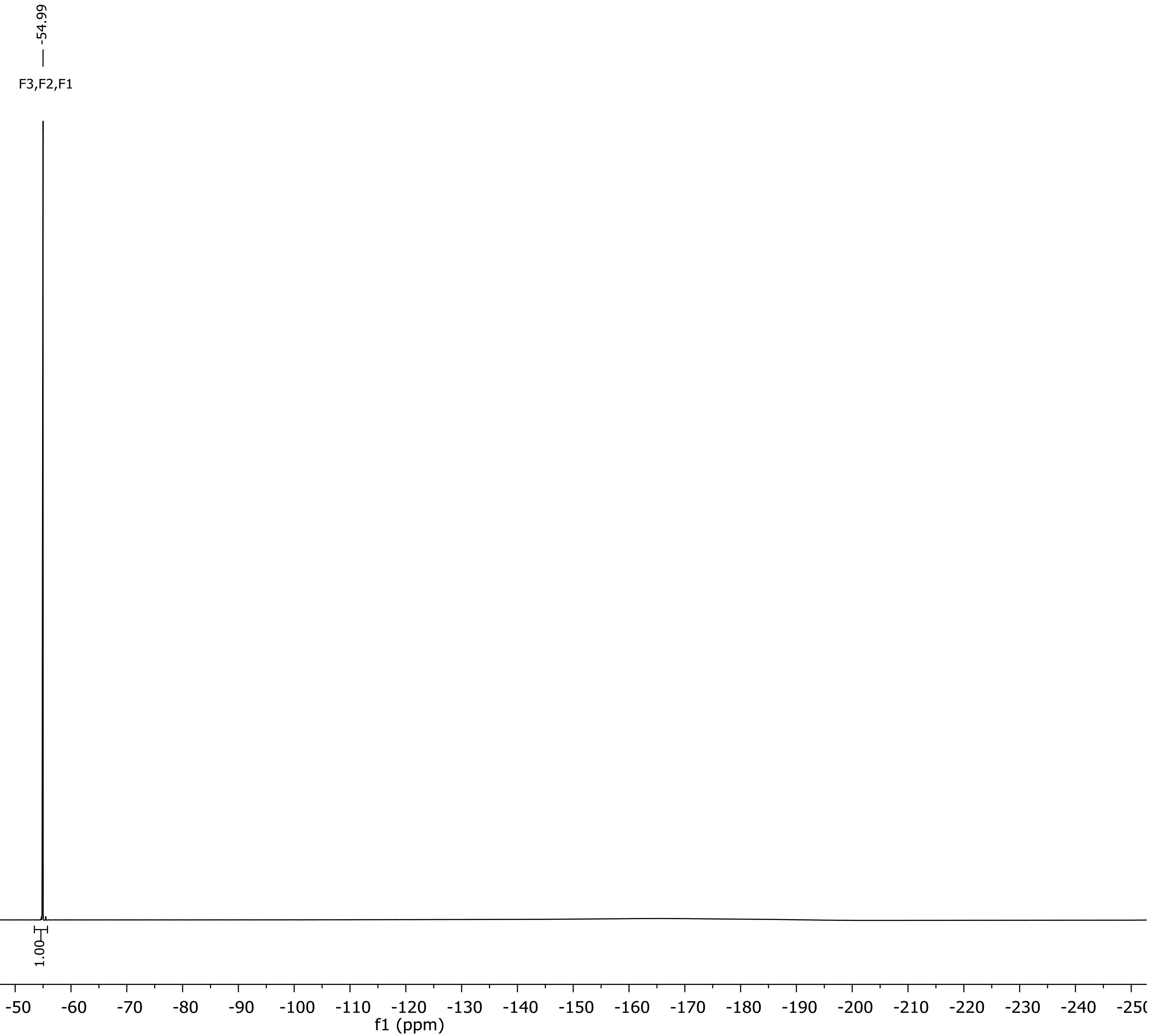

(±)-anti-**13** + (±)-syn-**13**

Detector A Channel 1 211nm

Peak#	Ret. Time	Area%
1	15.415	2.871
2	16.972	2.836
3	18.434	46.872
4	34.581	47.421
Total		100.000

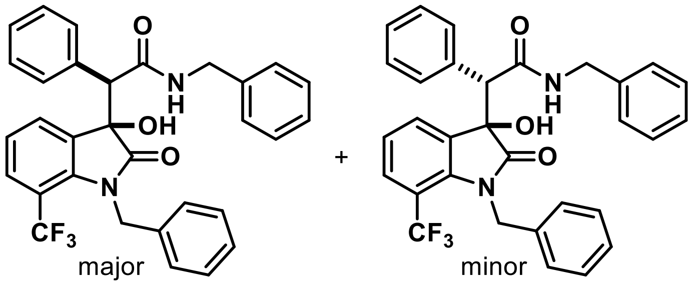

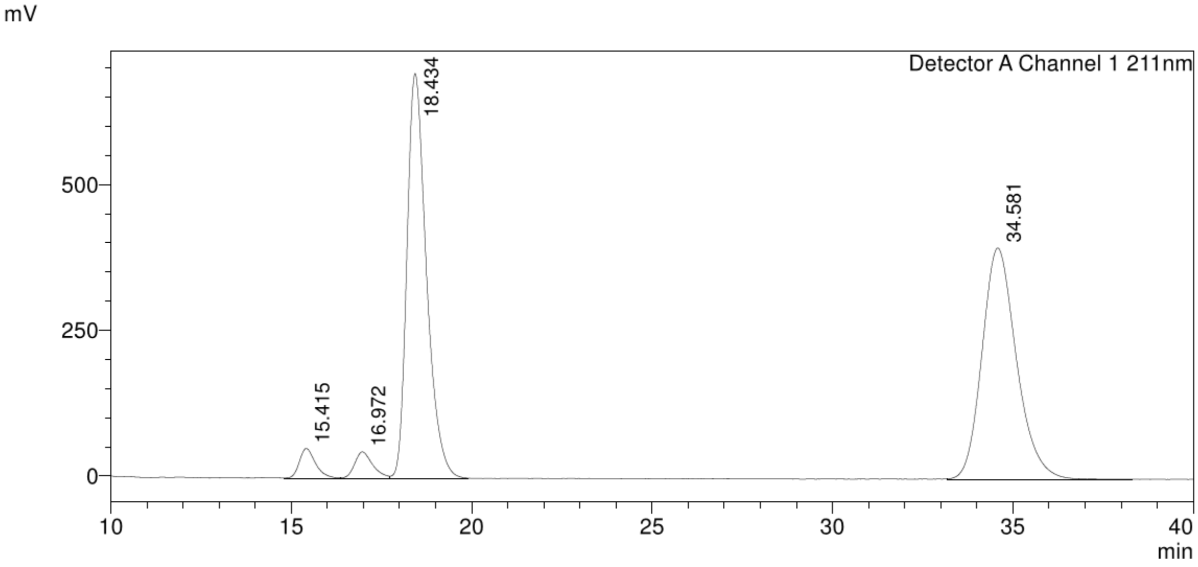

(–)-(2''''*R*,3*S*)-**13**

Detector A Channel 1 211nm

Peak#	Ret. Time	Area%
1	15.192	99.810
2	16.973	0.190
Total		100.000

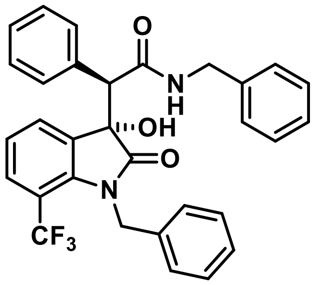

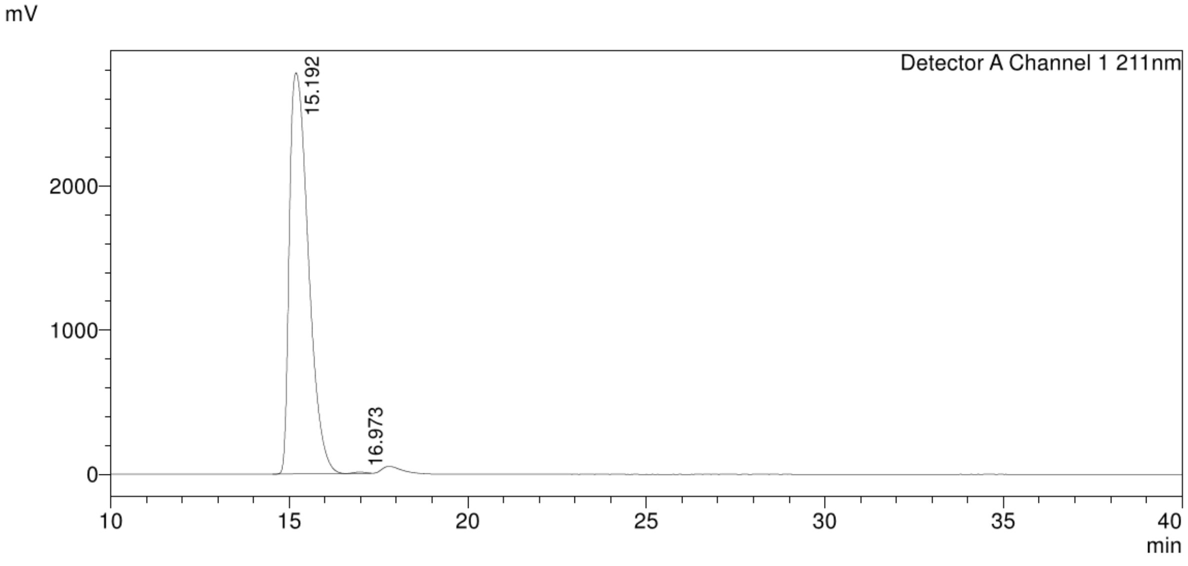

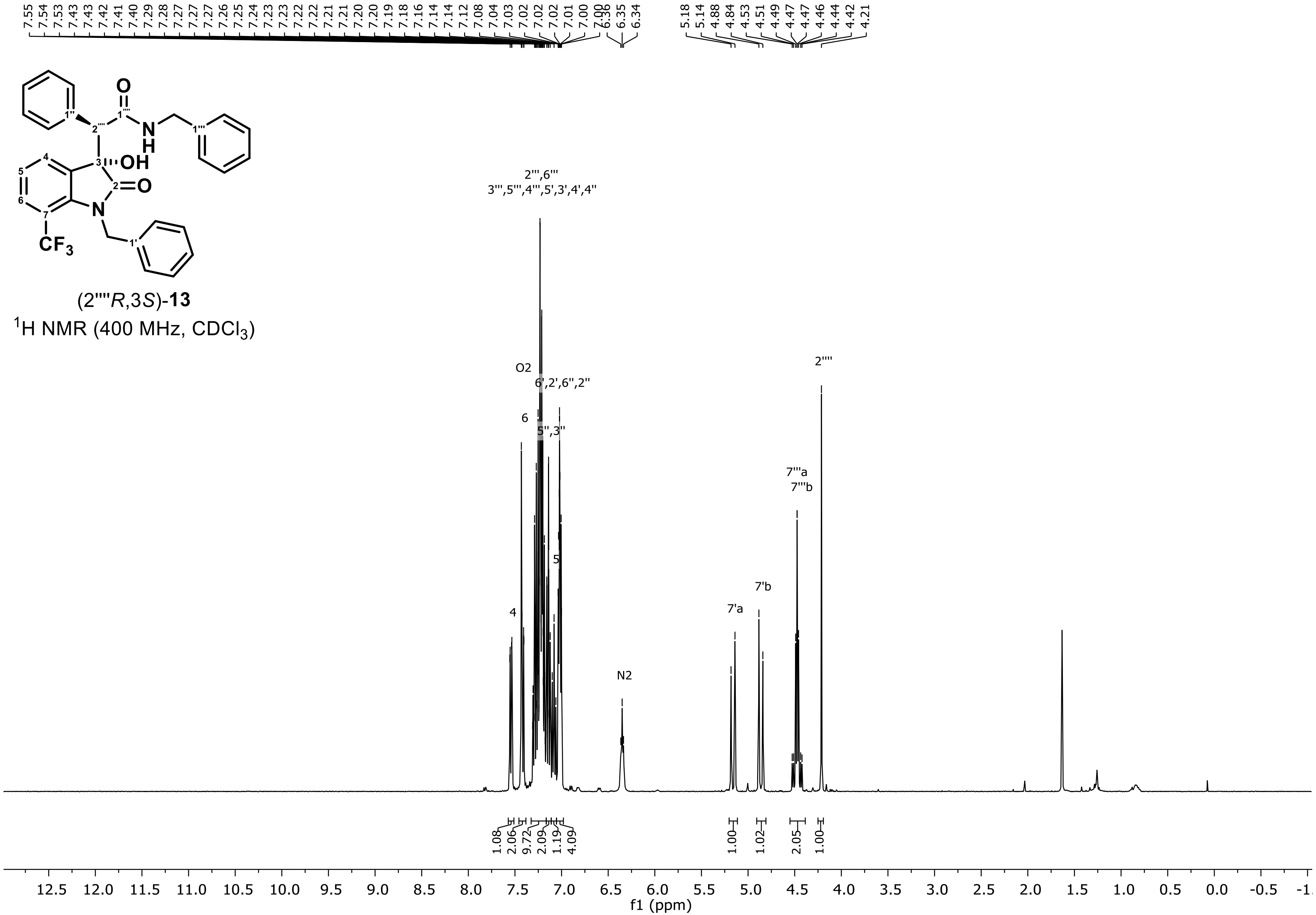
(2''''R,3S)-13
¹H NMR (400 MHz, CDCl₃)
2''',6'''
3''',5''',4''',5',3',4',4''
O2
6',2',6'',2''
6
5'',3''
5
4
N2
7'a
7'b
7'''a
7'''b
2''''
f1 (ppm)

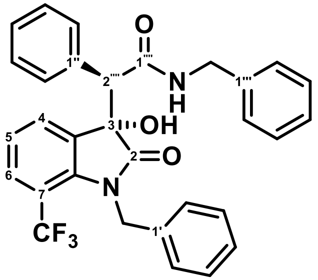

(2''''*R*,3*S*)-**13**

$^{13}C\ \{^{1}H\}$ NMR (100 MHz, $CDCl_3$)

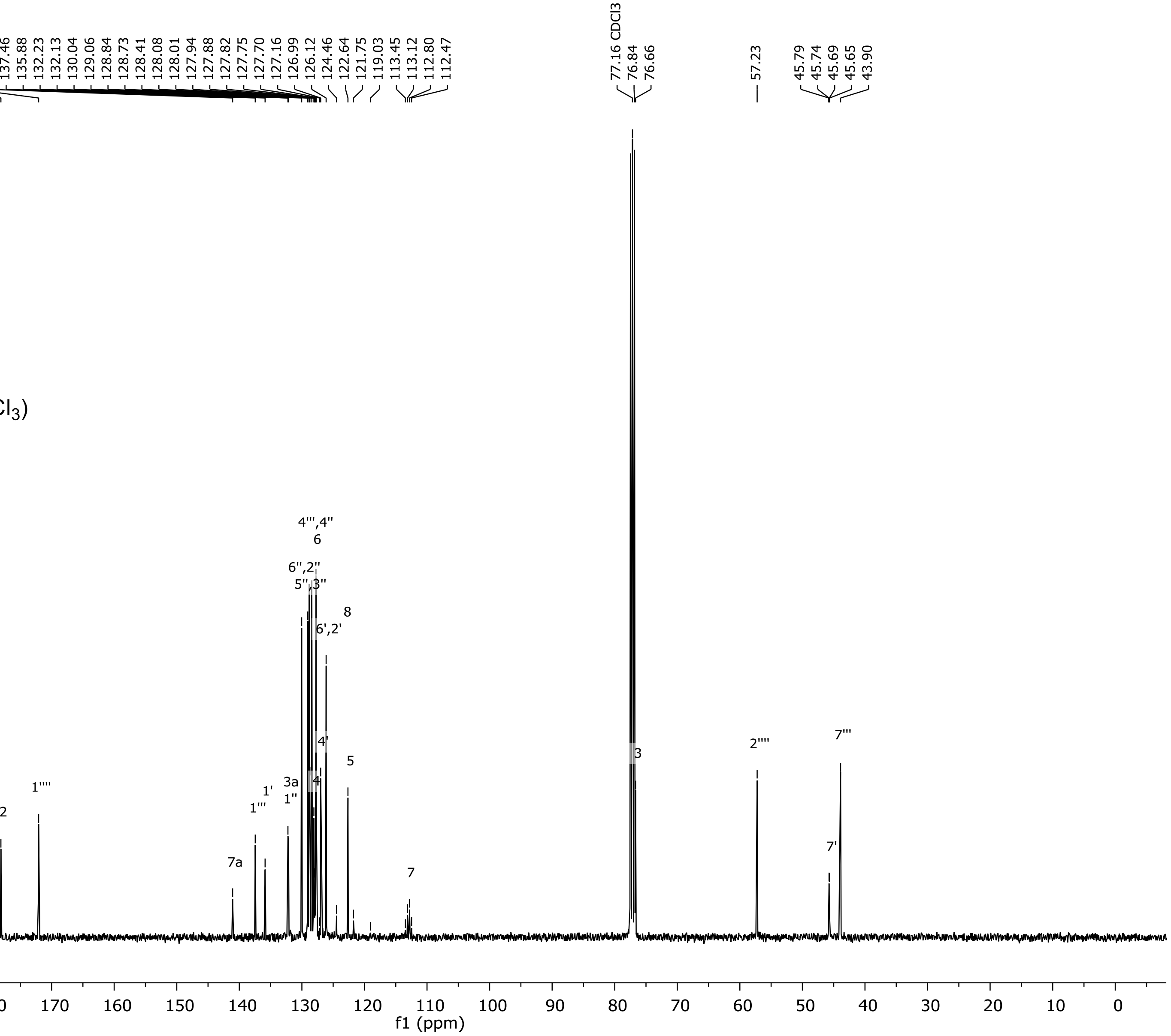

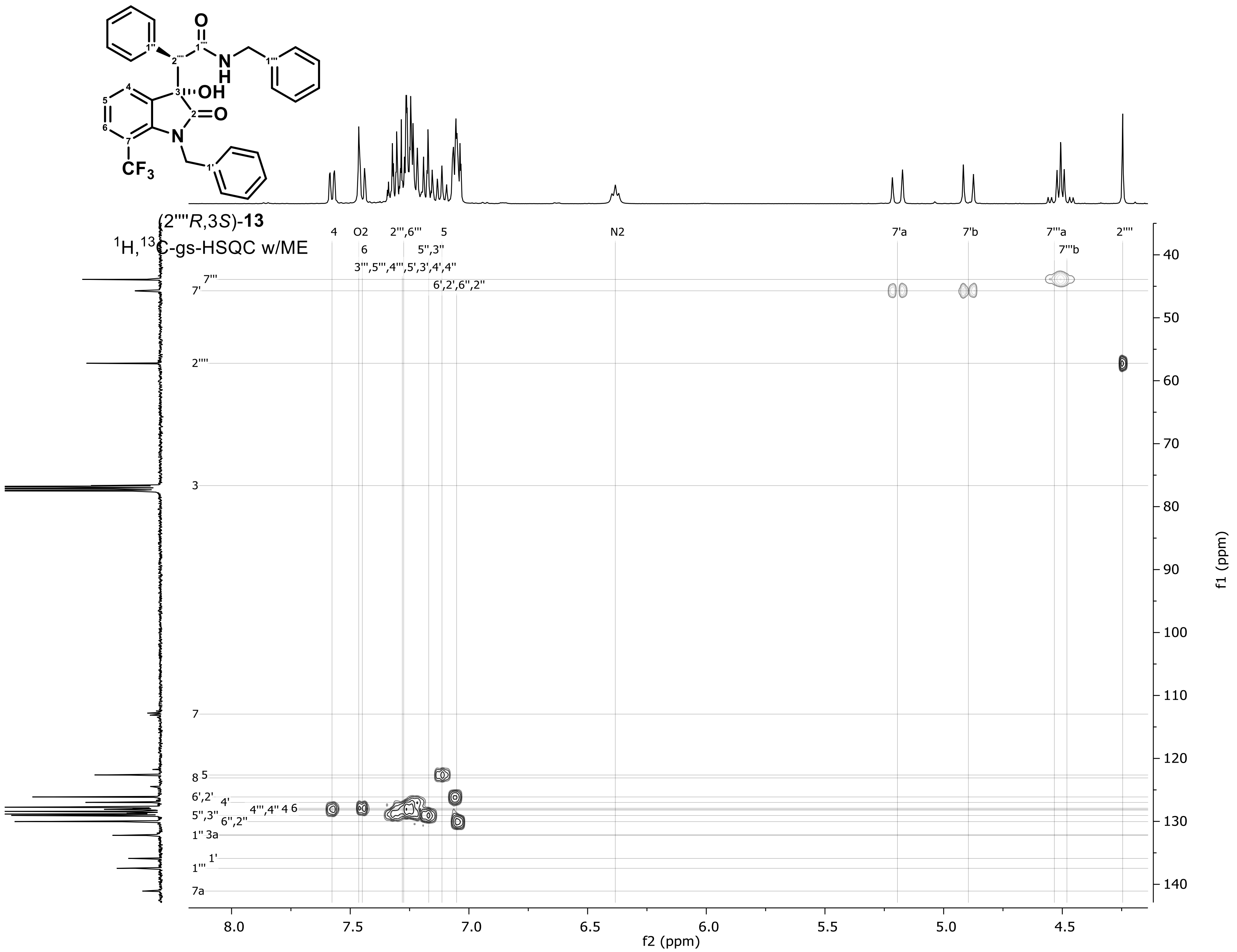
(2''''R,3S)-13
^{1}H,^{13}C-gs-HSQC w/ME
f2 (ppm)
f1 (ppm)

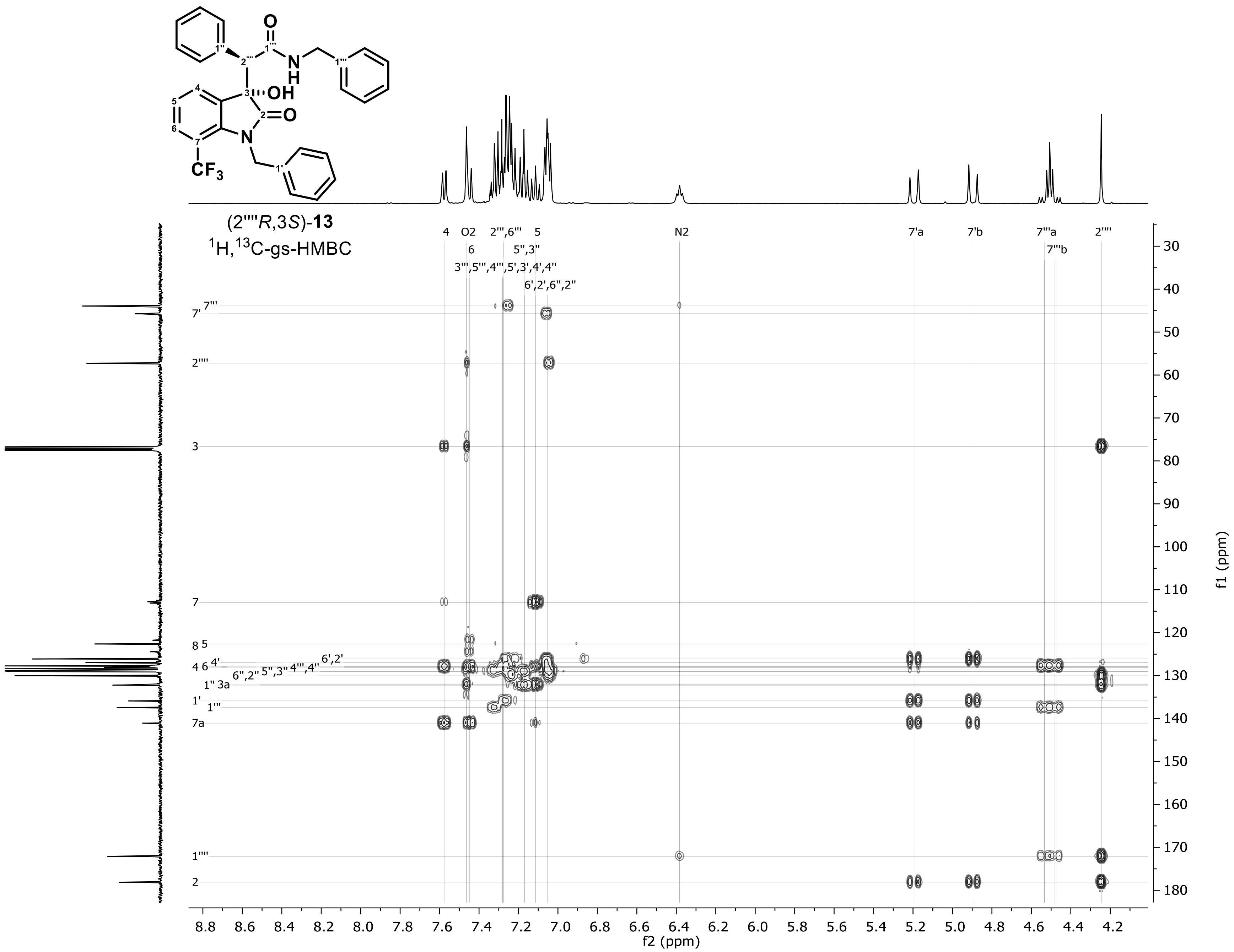

(2''''*R*,3*S*)-**13**
$^{1}H,^{13}C$-gs-HMBC

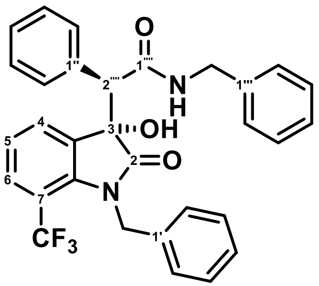

(2''''R,3S)-**13**

$^{19}F\{^{1}H\}$ NMR (377 MHz, $CDCl_3$)

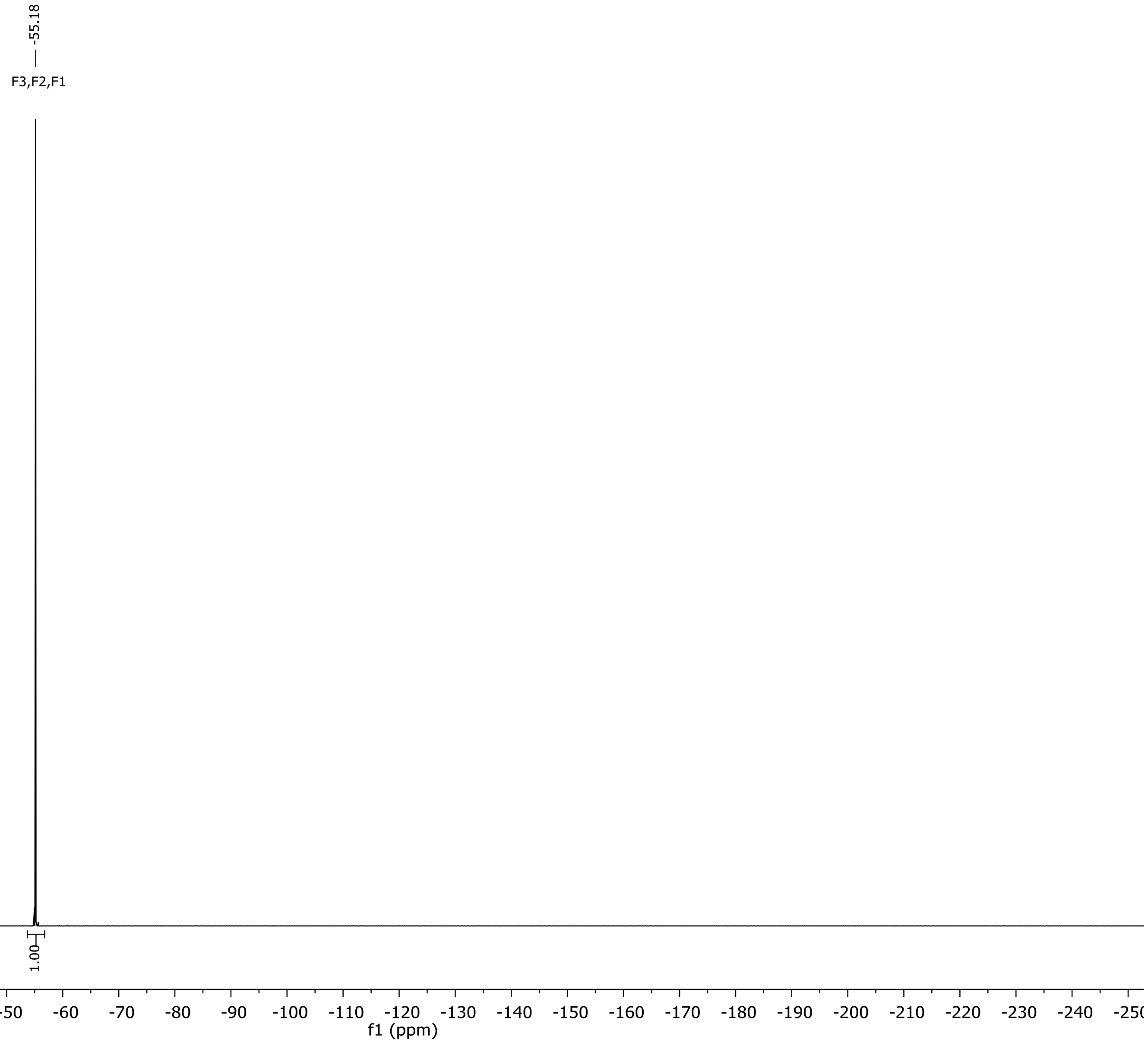

h) N-benzyl-2-(1-benzyl-7-chloro-3-hydroxy-2-oxoindolin-3-yl)-2-phenylacetamide (14)

To a 25 mL round bottomed flask was added phenylacetic anhydride (95.3 mg, 0.375 mmol), 7-chloro-*N*-benzylisatin (68.0 mg, 0.250 mmol), and (2*R*,3*S*)-HyperBTM (3.9 mg, 0.012 mmol). The mixture was cooled to 0 °C and CH_2Cl_2 (6.0 mL, 0.04 M) and Hünig's base (54.0 µL, 0.312 mmol) were added. The mixture was stirred at 0 °C for 3 h. Benzylamine (82.0 µL, 0.750 mmol) was added and the reaction was stirred overnight at room temperature. The solvent was removed under reduced pressure and the reaction purified via column chromatography (Biotage, 10 g SiO_2 column, EtOAc in petroleum ether 0-20% (5CV), 20-25% (3CV), 25-30% (6CV), 35-60% (3CV)) to give major diastereomer as a colourless oil (36 mg, 0.08 mmol, 31%) and minor diastereomer as a colourless oil (18 mg, 0.04 mmol, 16%); combined 54 mg, 0.12 mmol, 47%, 75:25 d.r.

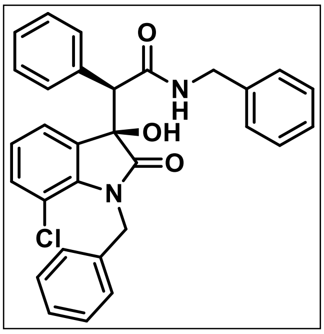

Major (2''''*R*,3*R*)-**14**: **Chiral HPLC analysis** Chiralpak IB (95:5 hexane:IPA, flow rate 2 ml·min^{-1}, 211 nm, 40 °C) t_R (2''''*S*, 3*S*)-**14**: 16.5 min, t_R (2''''*R*, 3*R*)-**14**: 21.5 min, <1:99 er; $\boldsymbol{\alpha_D^{20}}$ = −63.5 (*c* 0.50, $CHCl_3$); $\boldsymbol{\nu_{max}}$ (thin film) 3347 (m(br), OH, NH), 3086 (w), 3063 (w), 3030 (w), 2928 (w), 1721 (s, C=O, lactam), 1647 (m, C=O, amide), 1607 (m), 1584 (w), 1530 (m), 1497 (m), 1468 (m), 1452 (s), 1425 (w), 1354 (m), 1221 (w), 1167 (m), 1134 (m), 1078 (w), 1024 (w), 959 (w), 910 (w), 791 (w); **^{1}H NMR** (400 MHz, $CDCl_3$) δ_H 7.38 (1H, dd, $^3J_{HH}$ = 7.5 Hz, $^4J_{HH}$ = 1.2 Hz, ArC^4*H*), 7.36 – 7.27 (4H, m, PhC$^{4''}$*H*, PhC$^{3''',4''',5'''}$*H*), 7.23 – 7.16 (4H, m, PhC$^{3'',5''}$*H*, PhC$^{2''',6'''}$*H*), 7.15 (1H, dd, $^3J_{HH}$ = 8.2 Hz, $^4J_{HH}$ = 1.2 Hz, ArC^6*H*), 7.15 – 7.05 (3H, m, PhC$^{3',4',5'}$*H*), 7.00 (1H, s, O*H*), 6.95 – 6.90 (2H, m, PhC$^{2'',6''}$*H*), 6.93 (1H, dd, $^3J_{HH}$ = 8.2 Hz, 7.5 Hz, ArC^5*H*), 6.57 – 6.52 (2H, m, PhC$^{2',6'}$*H*), 5.91 (1H, t, $^3J_{HH}$ = 5.9 Hz, N*H*), 5.09 (1H, d, $^2J_{HH}$ = 16.4 Hz, NC$H_a$$H_b$-Ph), 4.98 (1H, d, $^2J_{HH}$ = 16.4 Hz, NC$H_a$$H_b$-Ph), 4.51 (1H, dd, $^2J_{HH}$ = 15.5 Hz, $^3J_{HH}$ = 5.9 Hz, NHC$H_a$$H_b$-Ph), 4.47 (1H, dd, $^2J_{HH}$ = 15.5 Hz, $^3J_{HH}$ = 5.9 Hz, NHC$H_a$$H_b$-Ph), 4.29 (1H, s, C*H*-Ph); **^{13}C{^{1}H} NMR** (100 MHz, $CDCl_3$) δ_C 175.5 (*C*(O)NBn), 173.3 (*C*(O)NHBn), 139.4 (Ar*C*^{7a}), 137.4 (Ph*C*$^{1'''}$$CH_2$NH), 137.1 (Ph*C*$^{1'}$$CH_2$N), 132.5 (Ar*C*^{6}H), 132.4 (Ph*C*$^{1''}$CH), 132.1 (Ar*C*^{3a}), 130.7 (Ph*C*$^{2'',6''}$H), 129.1 (Ph*C*$^{3'',5''}$H), 129.0 (Ph*C*$^{4''}$H), 128.9 (Ph*C*$^{3''',5'''}$H), 128.5 (Ph*C*$^{3',5'}$H), 127.9 (Ph*C*$^{4'''}$H), 127.7 (Ph*C*$^{2''',6'''}$H), 126.8 (Ph*C*$^{4'}$H), 125.9 (Ph*C*$^{2',6'}$H), 124.5 (Ar*C*^{4}H), 124.1 (Ar*C*^{5}H), 115.7 (Ar*C*^{7}Cl), 78.4 (*C*H-OH), 56.9 (*C*H-Ph), 45.0 (N*C*H_2-Ph), 43.9 (NH*C*H_2-Ph); **HRMS** (ESI$^+$) *m/z* calcd for $[M+H]^+$ $C_{30}H_{26}ClN_2O_3$ 497.1626, found 497.1624 (−0.5 ppm).

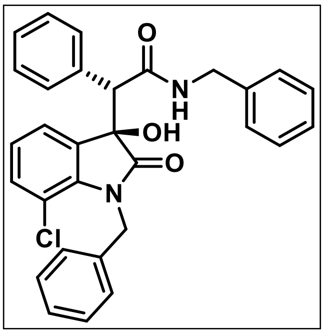

Minor (2''''*S*,3*R*)-**14**: **Chiral HPLC analysis** Chiralpak IB (95:5 hexane:IPA, flow rate 2 mL·min^{-1}, 211 nm, 40 °C) t_R (2''''*R*,3*S*)-**14**: 18.4 min, t_R (2''''*S*,3*R*)-**14**: 25.6 min, <1:99 er; $\boldsymbol{\alpha_D^{20}}$ = −48.2 (*c* 0.5, $CHCl_3$); $\boldsymbol{\nu_{max}}$ (thin film, MeCN) 3244 (broad, OH, NH), 3100 (w), 3063 (w), 3030 (w), 1699 (s, C=O), 1643 (s), 1605 (m), 1591 (m), 1580 (m), 1452 (m), 1423 (m), 1364 (m), 1173 (m), 1136 (m), 1082 (w), 1022 (w), 1007 (w), 961 (w), 849 (w), 799 (w), 789 (w), 772 (w); **^{1}H NMR** (500 MHz, 3:1 $CD_3CN/(CD_3)_2SO$) δ_H 7.38 (1H, app t, $^3J_{HH}$ = 6.1Hz, N*H*), 7.33 – 7.21 (11H, m, PhC*H*), 7.21 – 7.16 (2H, m, PhC*H*), 7.14 (1H, dd, $^3J_{HH}$ = 8.1 Hz, $^4J_{HH}$ = 1.3 Hz, ArC^6*H*), 7.11 – 7.05 (2H, m, PhC$^{2'6'}$*H*), 7.02 (1H, dd, $^3J_{HH}$ = 7.4 Hz, $^4J_{HH}$ = 1.3 Hz, ArC^4*H*), 6.94 (1H, dd, $^3J_{HH}$ = 8.1 Hz, 7.4 Hz, ArC^5*H*), 6.08 (1H, s, O*H*), 5.19 (1H, d, $^2J_{HH}$ = 16.7 Hz, NC$H_a$$H_b$-Ph), 5.12 (1H, d, $^2J_{HH}$ = 16.7 Hz, NC$H_a$$H_b$-Ph), 4.38 (1H, dd, $^2J_{HH}$ = 5.1 Hz, $^3J_{HH}$ = 6.2 Hz, NHC$H_a$$H_b$-Ph), 4.32 (1H, dd, $^2J_{HH}$ = 15.1 Hz, $^3J_{HH}$ = 6.0 Hz, NHC$H_a$$H_b$-Ph), 4.26 (1H, s, C*H*-Ph); **^{13}C {^{1}H} NMR** (100 MHz, 3:1 $CD_3CN/(CD_3)_2SO$) δ_C 179.0 (*C*(O)NBn), 170.5 (*C*(O)NHBn), 140.0_1 and 139.9_6 (Ph*C*$^{1'''}$$CH_2$NH, Ar*C*^{7a}), 138.6 (Ph*C*$^{1'}$$CH_2$N), 135.5 (Ph*C*$^{1''}$CH), 134.6 (Ar*C*^{3a}), 131.7 (Ph*C*$^{2'',6''}$H), 131.1 (Ar*C*^{6}H), 129.0, 128.9 and 128.3 (3 × Ph*C*^{3,5}H), 128.1 (Ph*C*$^{2''',6'''}$H), 128.3, 127.4_9 and 127.4_6 (3 × Ph*C*^{4}H), 126.8 (Ph*C*$^{2',6'}$H), 124.6 (Ar*C*^{4}H), 123.7 (Ar*C*^{5}H), 114.7 (Ar*C*^{7}Cl), 76.1 (*C*-OH), 59.0 (*C*H-Ph), 45.2 (N*C*H_2-Ph), 43.0 (NH*C*H_2-Ph); **HRMS** (ESI$^+$) *m/z* calcd for $[M+H]^+$ $C_{30}H_{26}ClN_2O_3$ 497.1626, found 497.1624 (−0.5 ppm).

(±)-anti-**16**

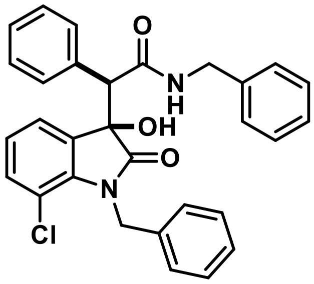

PDA Ch1 211nm

Peak#	Ret. Time	Area%
1	16.499	49.003
2	21.539	50.997
Total		100.000

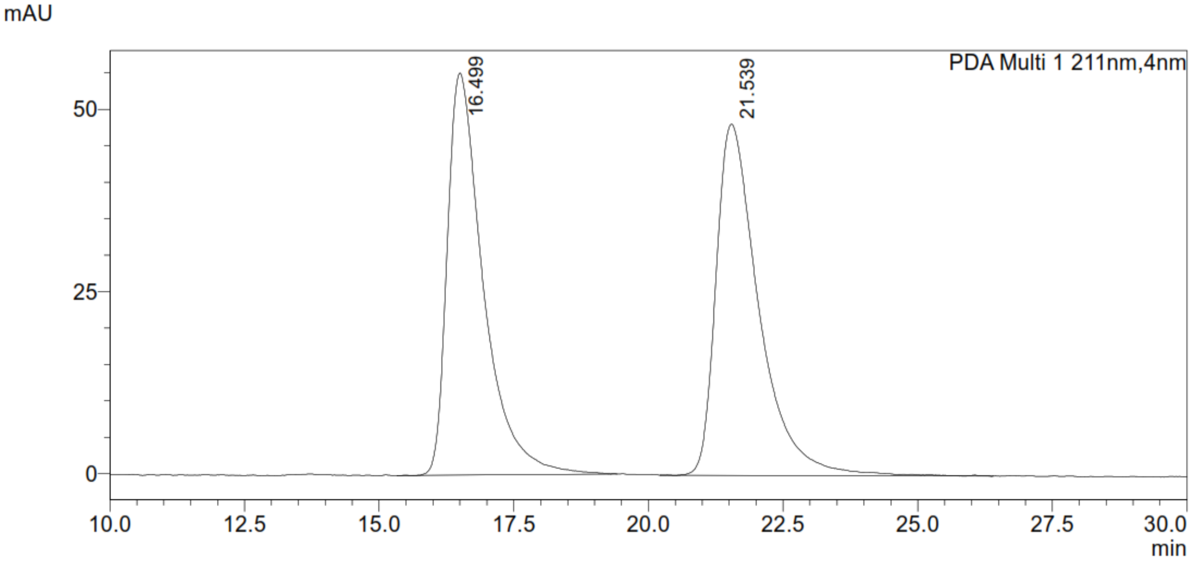

(–)-(2''''*R*,3*R*)-**14**

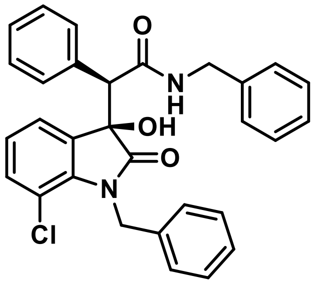

PDA Ch1 211nm

Peak#	Ret. Time	Area%
1	16.694	0.273
2	20.917	99.727
Total		100.000

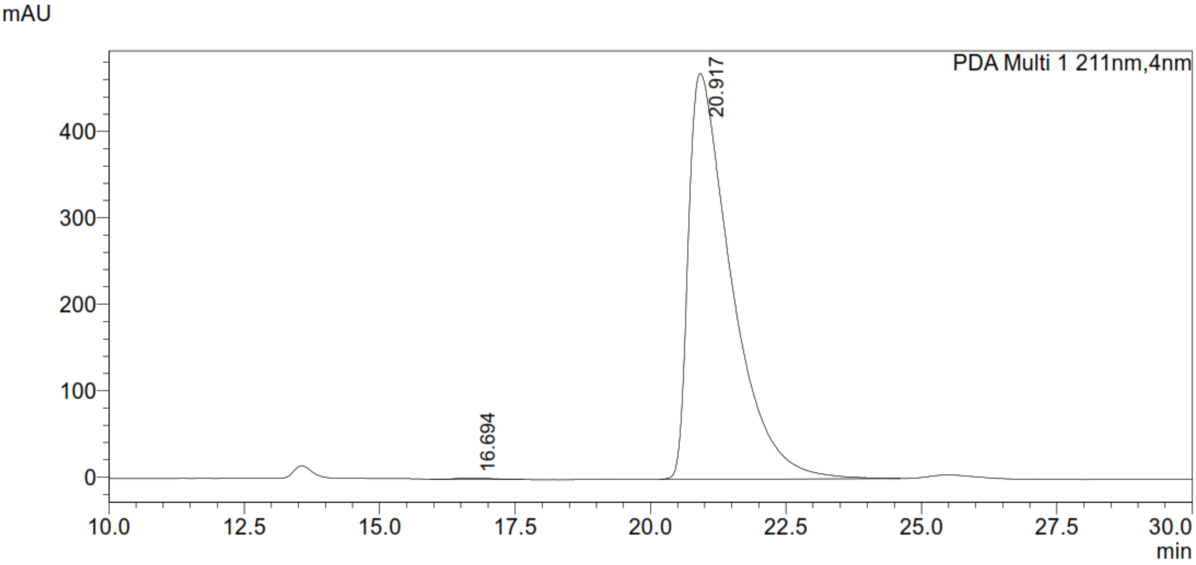

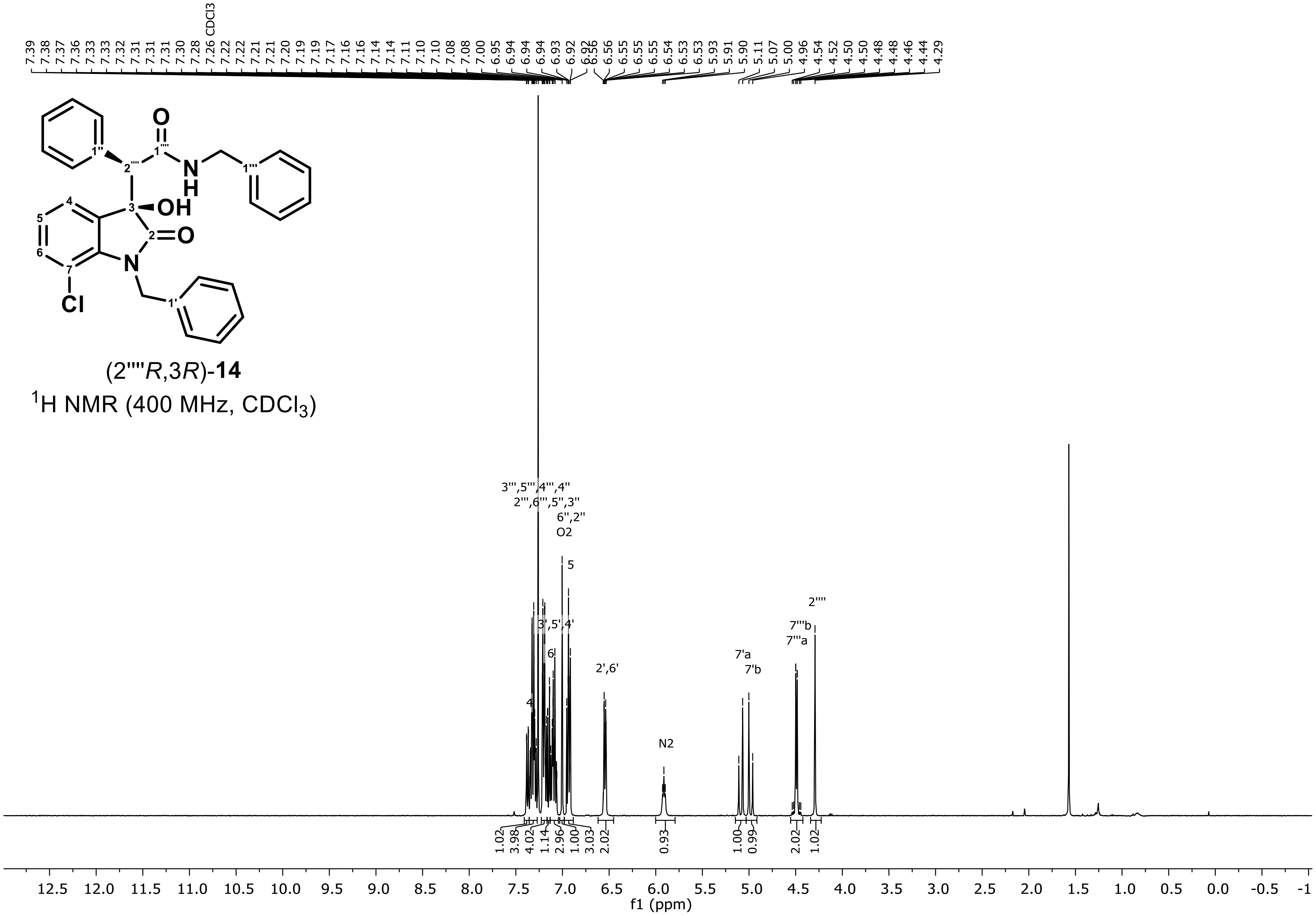

(2''''R,3R)-14
¹H NMR (400 MHz, CDCl3)
f1 (ppm)

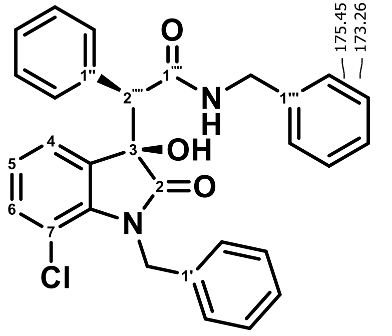

(2''''*R*,3*R*)-**14**

^{13}C NMR (101 MHz, $CDCl_3$)

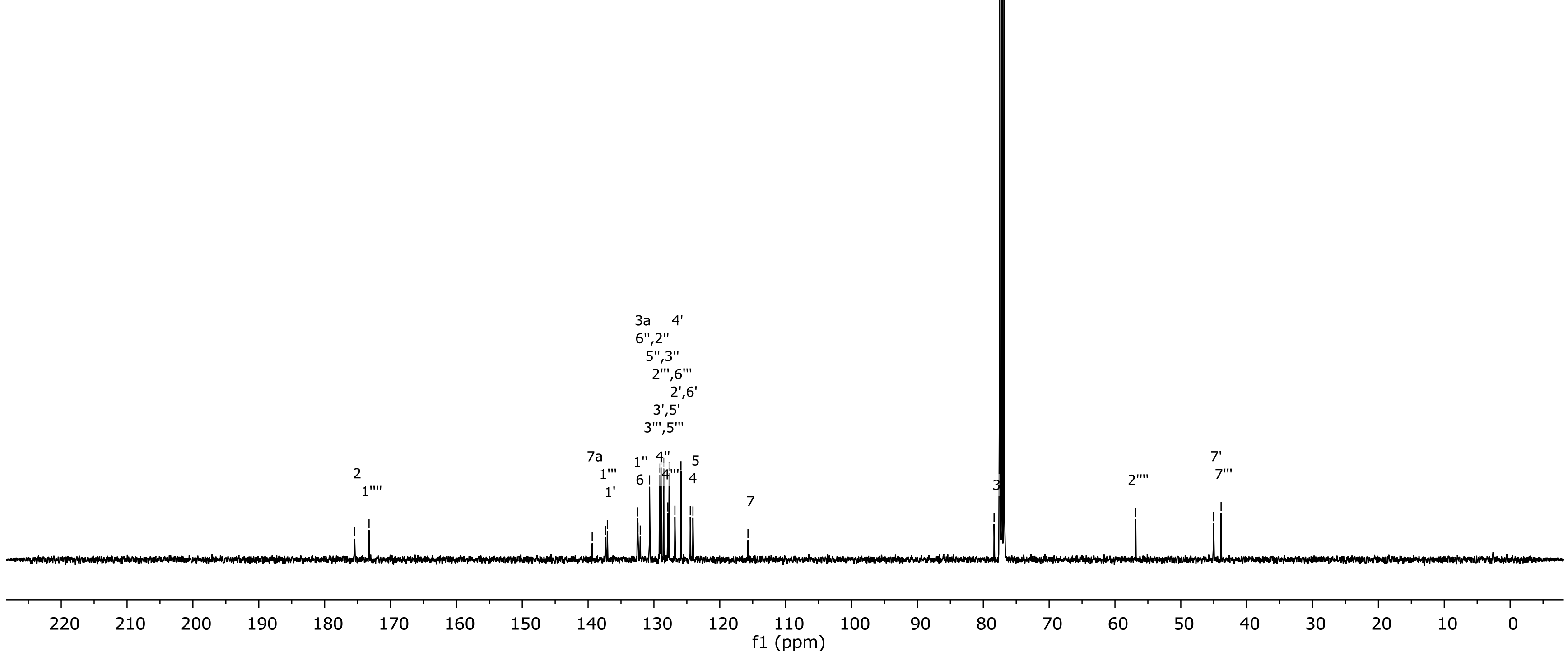

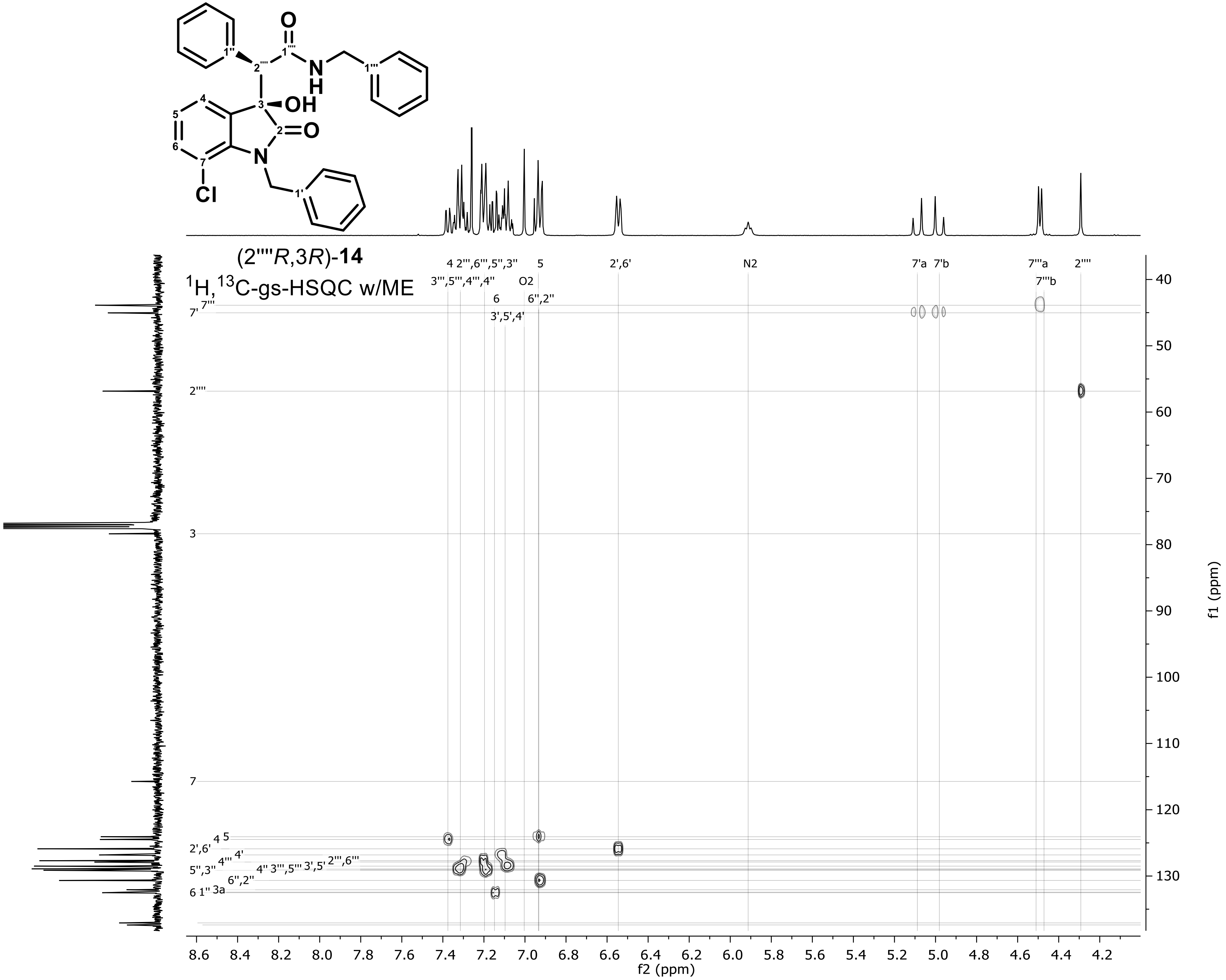

(2''''*R*,3*R*)-**14**

^1H,^{13}C-gs-HSQC w/ME

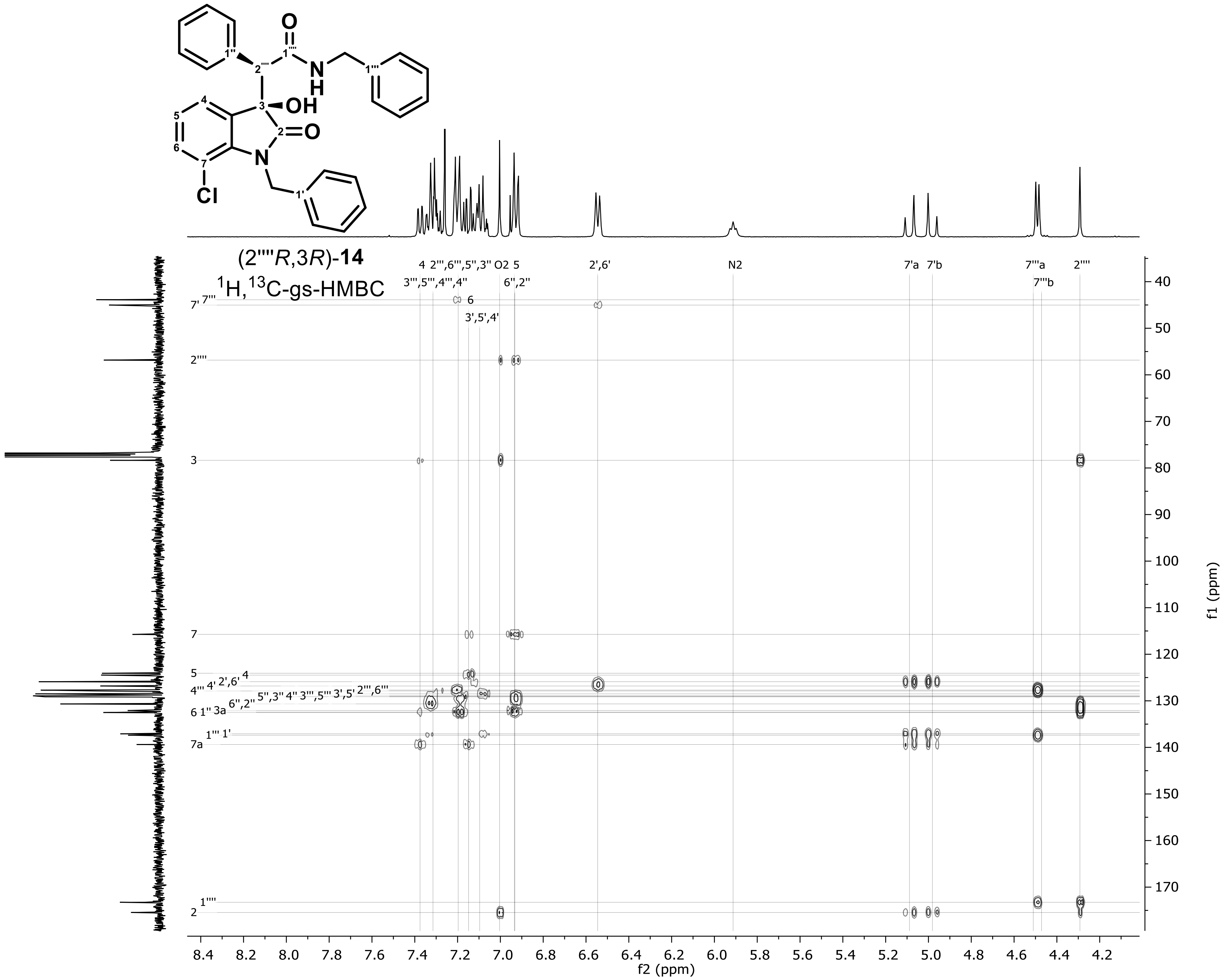

(2''''R,3R)-14
¹H,¹³C-gs-HMBC
f2 (ppm)
f1 (ppm)

(±)-syn-**14**

PDA Ch1 211nm

Peak#	Ret. Time	Area%
1	18.448	48.412
2	25.554	51.588
Total		100.000

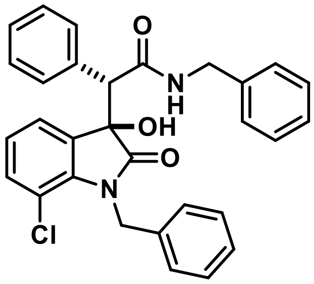

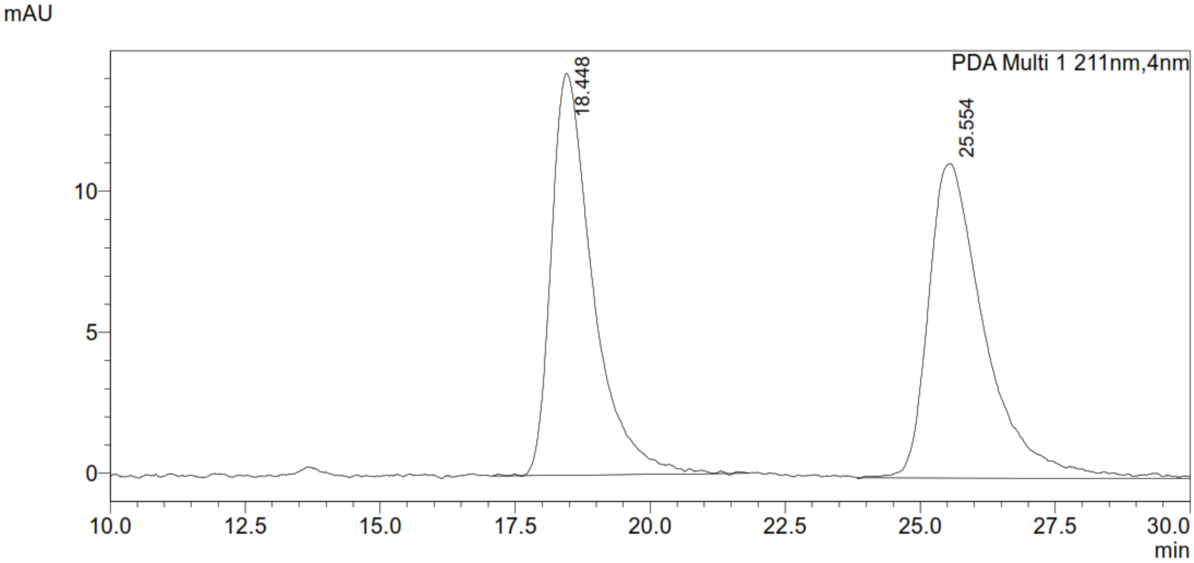

(–)-(2''''*S*,3*R*)-**14**

PDA Ch1 211nm

Peak#	Ret. Time	Area%
1	18.522	0.304
2	24.970	99.696
Total		100.000

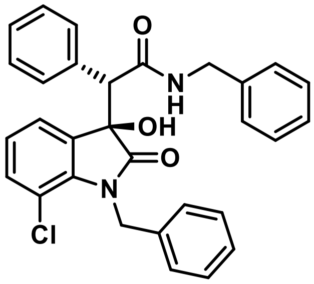

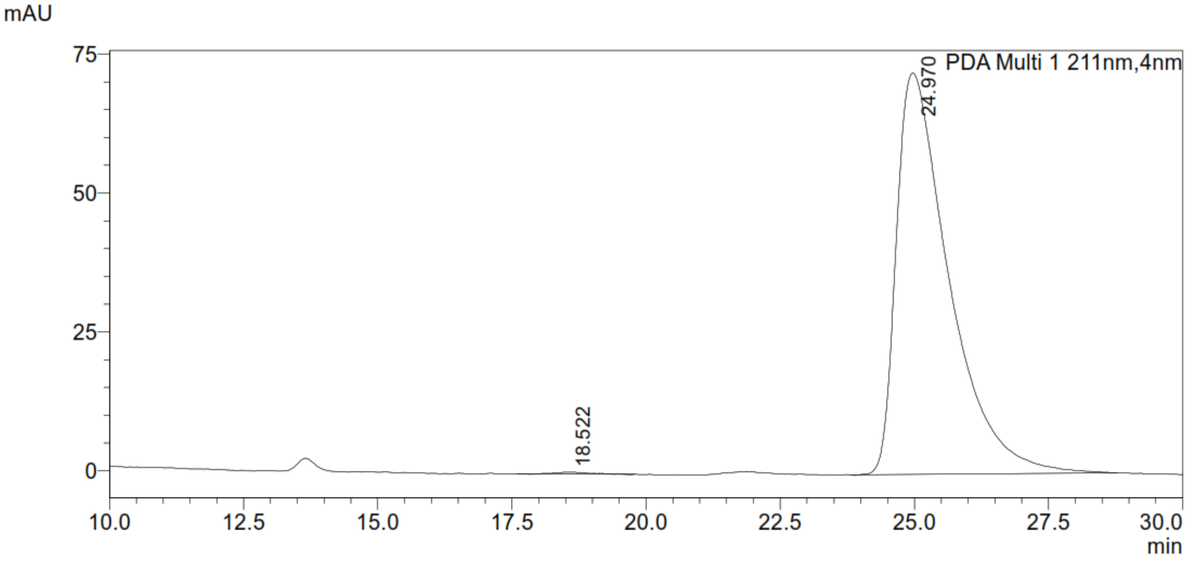

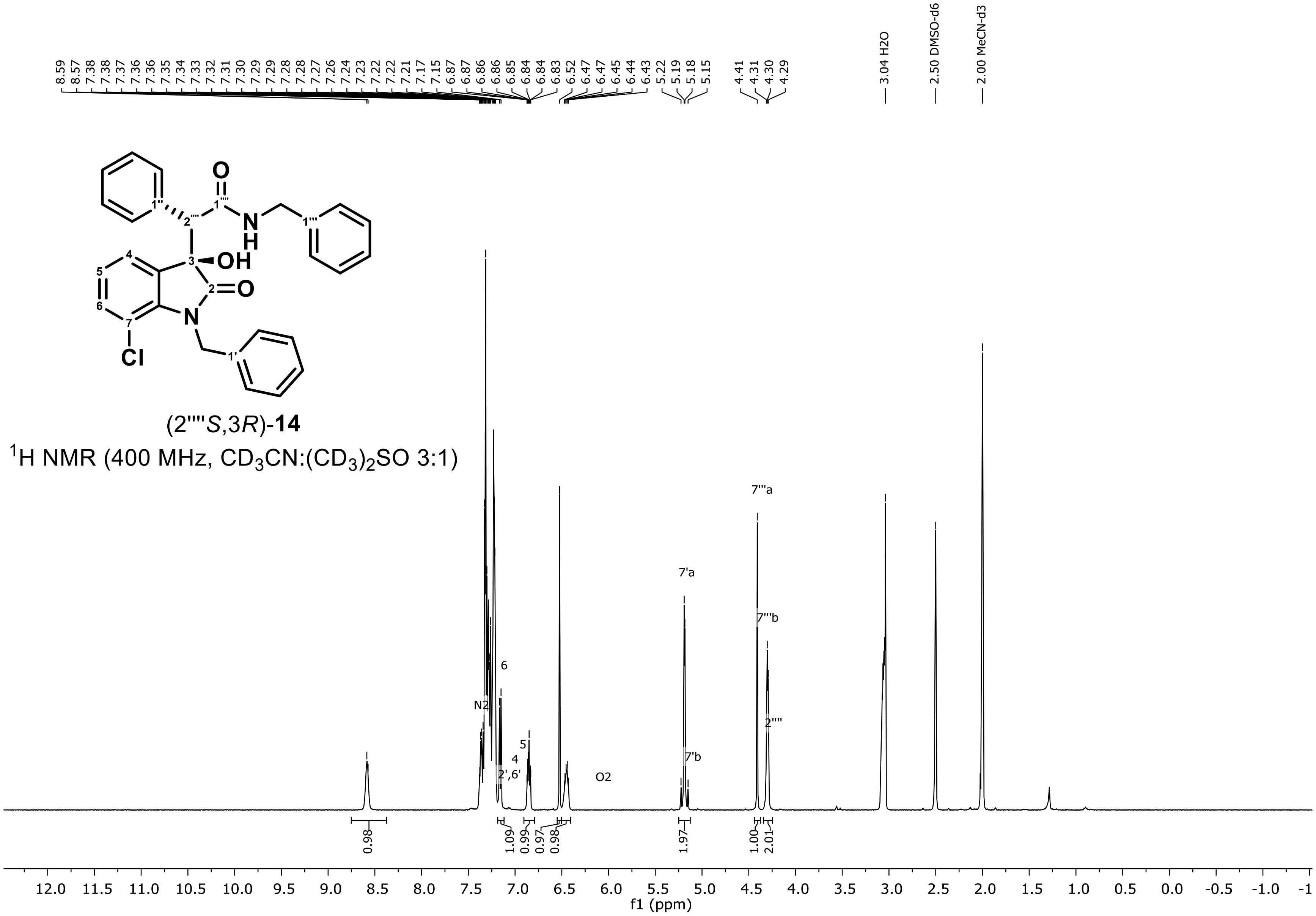
8.59 8.57 7.38 7.38 7.37 7.36 7.36 7.35 7.34 7.33 7.32 7.31 7.30 7.29 7.29 7.28 7.28 7.27 7.26 7.24 7.23 7.22 7.22 7.21 7.17 7.15 6.87 6.87 6.86 6.86 6.85 6.84 6.84 6.83 6.52 6.47 6.47 6.45 6.44 6.43 5.22 5.19 5.18 5.15 4.41 4.31 4.30 4.29
3.04 H2O
2.50 DMSO-d6
2.00 MeCN-d3
(2''''S,3R)-14
^{1}H NMR (400 MHz, CD_3CN:$(CD_3)_2SO$ 3:1)
N2
6
5
4
2',6'
O2
7'a
7'b
7'''a
7'''b
2''''
0.98
1.09
0.99
0.97
0.98
1.97
1.00
2.01
f1 (ppm)

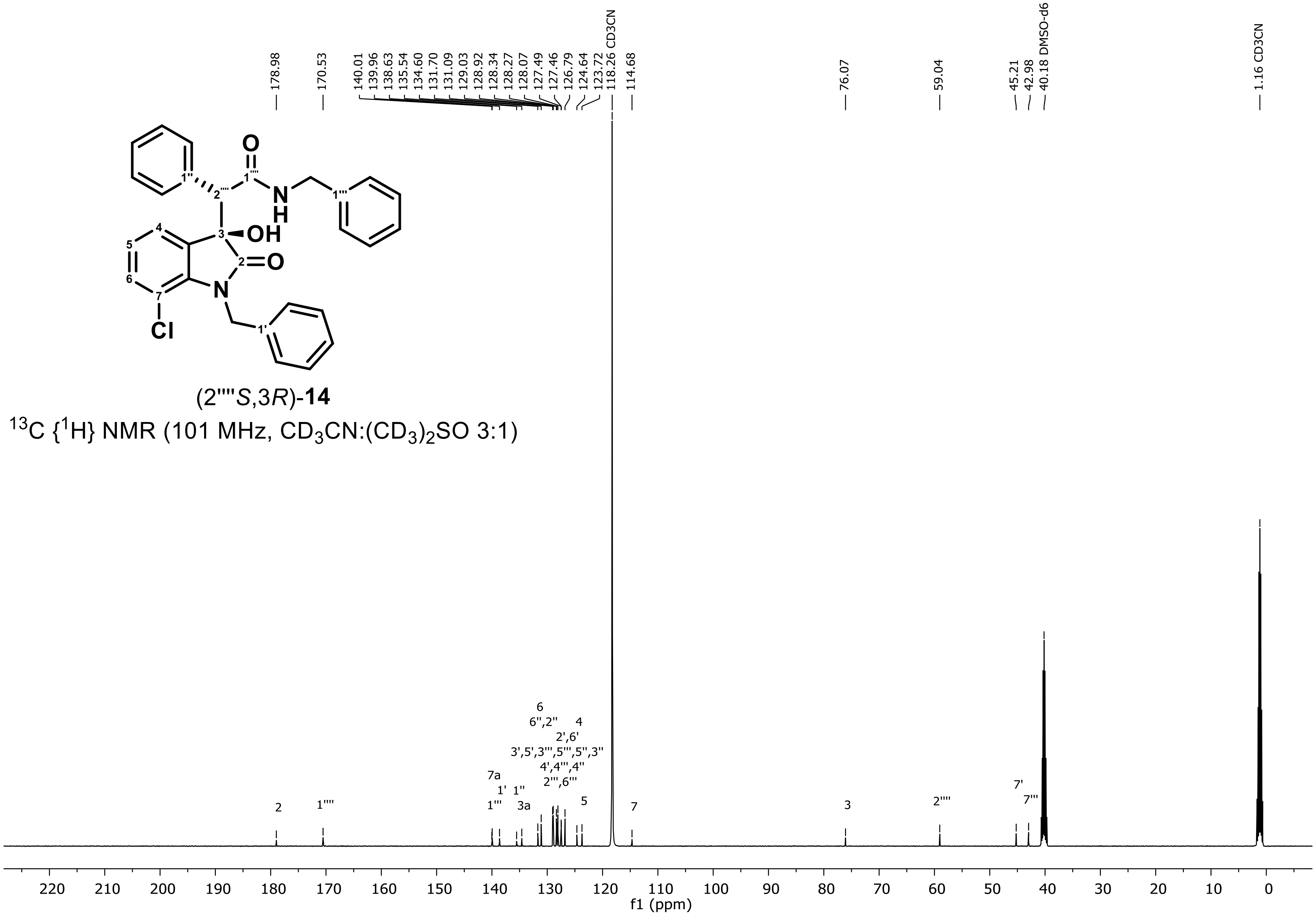

(2''''S,3R)-**14**

$^{13}C\ \{^1H\}$ NMR (101 MHz, CD_3CN:$(CD_3)_2SO$ 3:1)

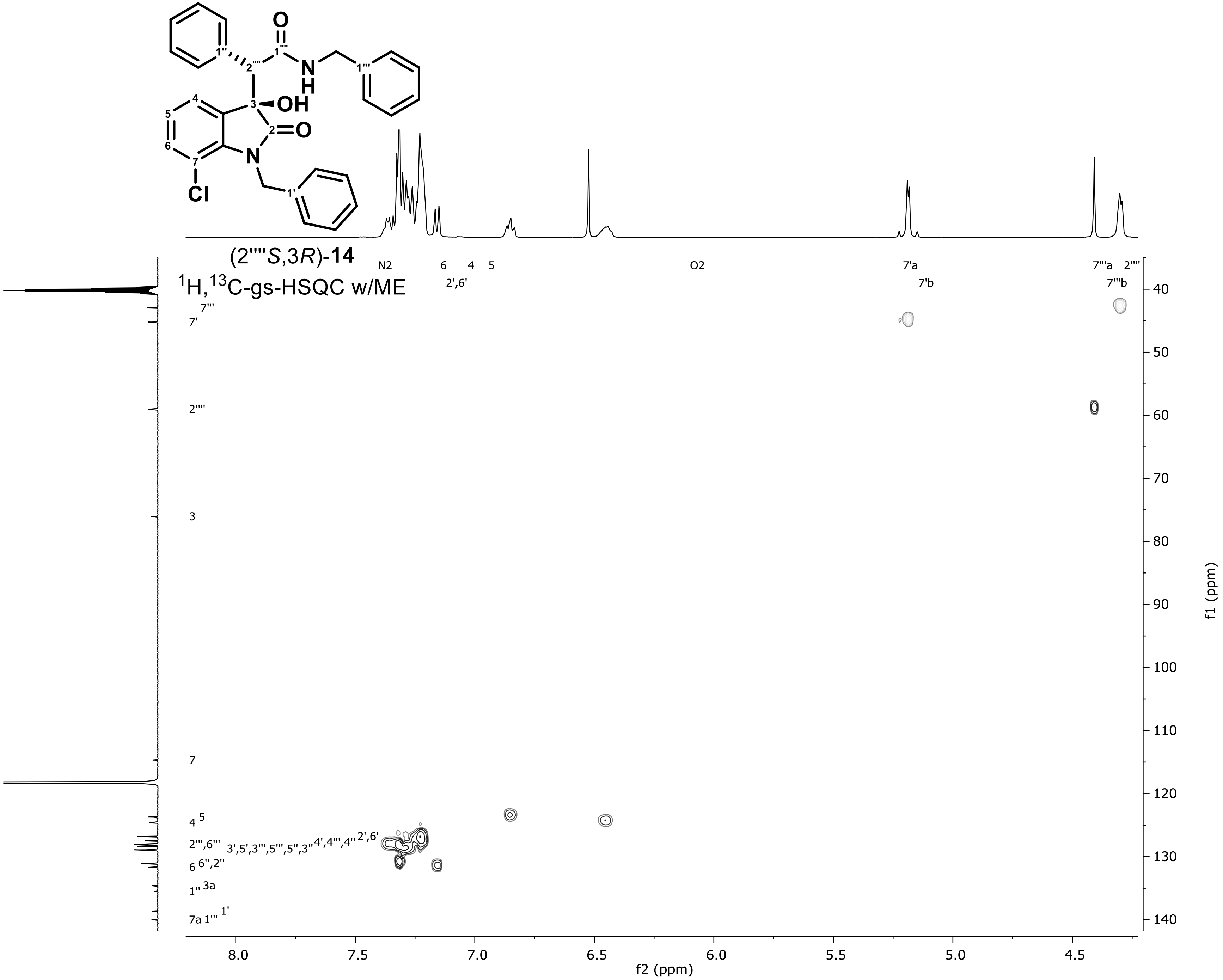
(2''''S,3R)-14
¹H,¹³C-gs-HSQC w/ME
f2 (ppm)
f1 (ppm)

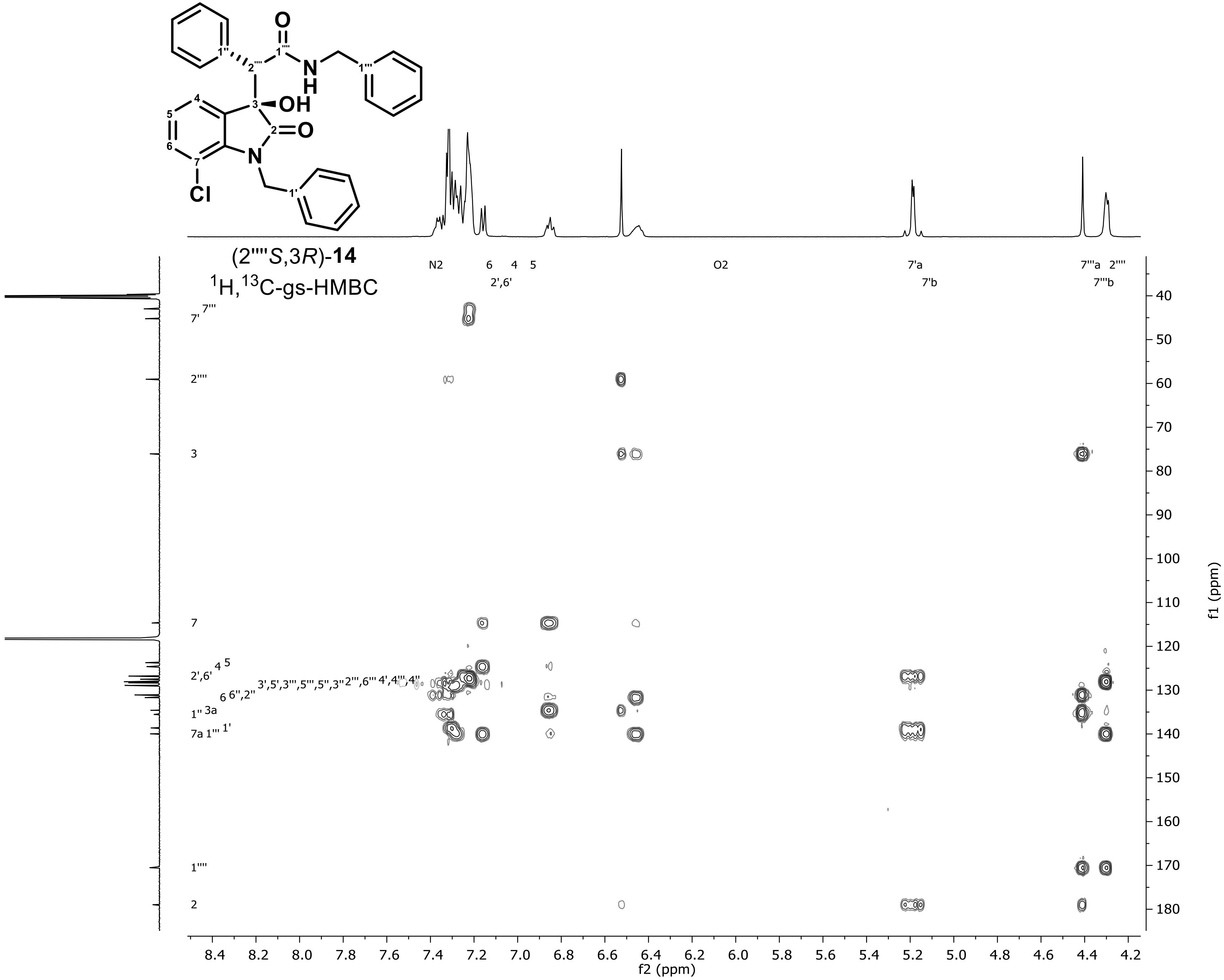
(2''''S,3R)-14
^{1}H,^{13}C-gs-HMBC
N2
6
4
5
2',6'
O2
7'a
7'b
7'''a
2''''
7'''b
7' 7'''
2''''
3
7
2',6' 4 5
6 6'',2''
3',5',3''',5''',5'',3'' 2''',6''' 4',4''',4''
1'' 3a
7a 1''' 1'
1''''
2
f2 (ppm)
f1 (ppm)

i) N,N'-Dibenzyl-2-(6-chloro-3-hydroxy-2-oxoindolin-3-yl)-2-phenylacetamide (15)

To a 25 ml round bottomed flask was added phenylacetic anhydride (95.3 mg, 0.375 mmol), *N*-benzyl 6-chloroisatin (67.9 mg, 0.250 mmol) and (2*R*,3*S*)-HyperBTM (3.9 mg, 0.008 mmol, 5 mol%). The solids were dissolved with dichloromethane (6.0 ml, 0.04 M) at 0 °C and Hünig's base (54 µl, 0.312 mmol) was added at 0 °C. The reaction was stirred at this temperature for three hours. Benzylamine (82µl, 0.750 mmol) was added at 0 °C and the mixture was left to be stirred overnight with the ice bath slowly warming to room temperature. To the mixture was added 1,3,5-trimethoxybenzene (0.1 M soln in CH_2Cl_2 500 µl, 0.05 mmol), and the solvent was removed under reduced pressure. Purification by column chromatography (Pentanes : EtOAc 9:1 → 1:1) gave the title compound as two diastereomers: major (64.0 mg, 0.129 mmol, 52%), minor (14.5 mg, 0.029 mmol, 12%), 73:27 d.r.

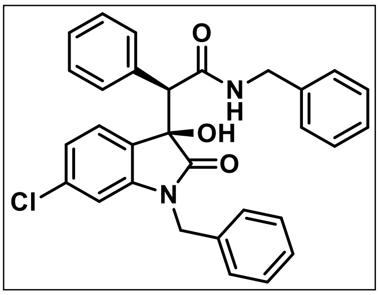

Major (2''''*R*,3*R*)-**15**: **R*f*** (0.47 Hexane : EtOAc 1:1); **Chiral HPLC analysis** Chiralpak IB (95:5 hexane:IPA, flow rate 2 ml·min^{-1}, 211 nm, 40 °C) t_R (2''''*R*,3*R*)-**15**: 18.0 min, t_R (2''''*S*,3*S*)-**15**: 25.6 min, >99:1 er; $\alpha_D^{20} = -14.0$ (*c* 2.03, $CHCl_3$); ***ν*max** (thin film) 3348 (broad, OH, NH), 3063 (w), 3030 (w), 2922 (w), 1717 (s, C=O, lactam), 1651 (m, C=O, amide), 1609 (s), 1539 (m), 1489 (m), 1452 (m), 1441 (m), 1373 (m), 1354 (m), 1219 (w), 1177 (w), 1123 (w), 1076 (m), 1030 (w), 1003 (w), 878 (w), 843 (w), 824 (w), 750 (s); **^{1}H NMR** (400 MHz, $CDCl_3$) δ_H 7.44 (1H, d, $^3J_{HH}$ = 8.0 Hz, ArC^4*H*), 7.38 – 7.26 (4H, m, PhC$^{4''}$*H*, PhC$^{3''',4''',5'''}$*H*), 7.26 – 7.16 (5H, m, PhC$^{4'}$*H*, PhC$^{3'',5''}$*H*, PhC$^{2''',6'''}$*H*), 7.16 – 7.09 (2H, m, NPhC$^{3',5'}$*H*), 7.03 – 6.92 (3H, m, ArC^5*H*, PhC$^{2'',6''}$*H*), 6.74 (1H, s, O*H*), 6.54 (2H, app d, $^3J_{HH}$ = 7.2 Hz, PhC$^{2',6'}$*H*), 6.42 (1H, d, $^4J_{HH}$ = 1.8 Hz, ArC^7*H*), 6.06 (1H, t, $^3J_{HH}$ = 5.9 Hz, N*H*), 4.89 (1H, d, $^2J_{HH}$ = 16.0 Hz, NC*H*$_a$H$_b$-Ph), 4.52 (1H, dd, $^2J_{HH}$ = 15.2 Hz, $^3J_{HH}$ = 5.9 Hz, NHC*H*$_a$H$_b$-Ph), 4.48 (1H, dd, $^2J_{HH}$ = 15.2 Hz, $^3J_{HH}$ = 5.9 Hz, NHCH$_a$*H*$_b$-Ph), 4.36 (1H, s, PhC$^{1''}$C*H*), 4.27 (1H, d, $^2J_{HH}$ = 16.0 Hz, NCH$_a$*H*$_b$-Ph); **^{13}C {^{1}H} NMR** (100 MHz, $CDCl_3$) δ_C 174.9 (*C*(O)NBn), 173.1 (*C*(O)NHBn), 144.5 (Ar*C*^{7a}), 137.5 (Ph*C*$^{1'''}$CH$_2$NH), 135.6 (Ar*C*^{3a}), 134.4 (Ph*C*$^{1'}$CH$_2$N), 132.6 (Ph*C*$^{1''}$CH), 130.6 (Ph*C*$^{2'',6''}$H), 129.1 (Ph*C*^{3,5}H), 128.9 (Ph*C*^{3,5}H), 128.8 (Ph*C*^{3,5}H), 127.8 (Ph*C*^{4}H), 127.6 (Ph*C*$^{2''',6'''}$H), 127.5 (Ph*C*^{4}H), 127.4 (Ar*C*^{6}Cl), 127.0 (Ph*C*^{4}H), 126.6 (N*C*H$_2$-Ph), 123.1 (Ar*C*^{5}H), 110.1 (Ar*C*^{7}H), 78.7 (*C*-OH), 56.9 (PhC$^{1''}$*C*H), 43.8 (N*C*H$_2$-Ph, NH*C*H$_2$-Ph); ***m/z*** (ESI$^+$) 91 ($C_7H_7^+$ 29%), 147 (14%), 151 (12%), 297 (18%), 346 ([M(^{35}Cl)-BnNHCOO]$^+$ 100%), 347 ([M(^{13}C,^{35}Cl)-BnNHCOO]$^+$ 29%), 348 ([M(^{13}C$_2$,^{35}Cl)-BnNHCOO]$^+$, [M(^{37}Cl)-BnNHCOO]$^+$ 31%), 349 ([M(^{13}C,^{35}Cl)-BnNHCOO]$^+$ 7%), 497 ([M(^{35}Cl)+H]$^+$ 38%), 498 ([M(^{13}C,^{35}Cl)+H]$^+$ 12%), 499 ([M(^{13}C$_2$,^{35}Cl)+H]$^+$, [M(^{37}Cl)+H]$^+$ 11%), 500 ([M(^{13}C,^{37}Cl)+H]$^+$ 4%), 519 ([M(^{35}Cl)+Na]$^+$ 24%); **HRMS** (ESI$^+$) *m/z* calcd for [M(^{35}Cl)+H]$^+$ $C_{30}H_{26}ClN_2O_3$ 497.1626, found 497.1621 (−1.1 ppm).

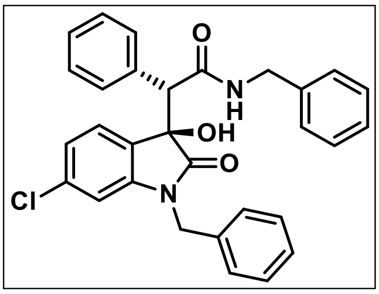

Minor (2''''*S*,3*R*)-**15**: **R*f*** (0.43 Hexane : EtOAc 1:1); **Chiral HPLC analysis** Chiralpak IB (95:5 hexane:IPA, flow rate 2 ml·min^{-1}, 211 nm, 40 °C) t_R (2''''*S*,3*R*)-**15**: 20.3 min, t_R (2''''*R*,3*S*)-**15**: 26.7 min, 99:1 er; $\alpha_D^{20} = -15.4$ (*c* 0.56, $CHCl_3$); ***ν*max** (thin film) 3296 (broad, OH, NH), 3088 (w), 3065 (w), 3030 (w), 2924 (w), 2853 (w), 1707 (s, C=O), 1639 (m), 1611 (s), 1491 (m), 1454 (m), 1439 (m), 1377 (m), 1356 (m), 1298 (w), 1252 (w), 1217 (w), 1179 (m), 1121 (w), 1074 (m), 1030 (w), 988 (w), 880 (w), 845 (w), 818 (w); **^{1}H NMR** (400 MHz, $CDCl_3$) δ_H 7.35 – 7.29 (2H, m, PhC$^{3''',5'''}$*H*), 7.29 – 7.24 (7H, m, PhC$^{3',4',5'}$*H*, PhC$^{2''',4''',6'''}$*H*), 7.23 (1H, d, $^3J_{HH}$ = 8.1 Hz, ArC^4*H*), 7.21 – 7.15 (2H, m, O*H*, PhC$^{4''}$*H*), 7.13 – 7.07 (2H, m, PhC$^{3'',5''}$*H*), 7.09 – 7.03 (4H, m, PhC$^{2',6'}$*H*, PhC$^{2'',6''}$*H*), 6.94 (1H, dd, $^3J_{HH}$ = 8.0 Hz, $^4J_{HH}$ = 1.8 Hz, ArC^5*H*), 6.43 (1H, d, $^4J_{HH}$ = 1.8 Hz, ArC^7*H*), 6.34 (1H, s(br), N*H*), 4.82 (1H, d, $^2J_{HH}$ = 15.8 Hz, NC*H*$_a$H$_b$-Ph), 4.64 (1H, d, $^2J_{HH}$ = 15.8 Hz, NCH$_a$*H*$_b$-Ph), 4.55 (1H, dd, $^2J_{HH}$ = 15.0 Hz, $^3J_{HH}$ = 5.9 Hz, NHC*H*$_a$H$_b$-Ph), 4.48 (1H, dd, $^2J_{HH}$ = 15.0 Hz, $^3J_{HH}$ = 5.7 Hz, NHCH$_a$*H*$_b$-Ph), 4.20 (1H, s, C*H*-Ph); **^{13}C {^{1}H} NMR** (126 MHz, $CDCl_3$) δ_C 176.7 (*C*(O)NBn), 172.2 (*C*(O)NHBn), 144.1 (Ar*C*^{7a}), 137.6 (Ar*C*$^{1'''}$CH$_2$NH), 135.5 (Ar*C*^{3a}), 134.8 (Ph*C*$^{1'}$CH$_2$N), 132.6 (Ph*C*$^{1''}$CH), 130.2 (Ph*C*$^{2'',6''}$H), 129.1, 128.9$_2$ and

128.8$_6$ (Ph$C^{3',5'}$H, Ph$C^{3'',5''}$H and Ph$C^{3''',5'''}$H), 128.6 (Ph$C^{4''}$H), 127.9 and 127.7 (Ph$C^{4'}$H, Ph$C^{4'''}$H), 127.8 (Ph$C^{2''',6'''}$H), 127.6 (ArC^{6}Cl), 27.4 (Ph$C^{2',6'}$H), 125.3 (ArC^{4}H), 123.0 (ArC^{5}H), 110.0 (ArC^{7}H), 78.2 (*C*-OH), 57.0 (*C*H-Ph), 44.1 (N*C*H$_2$-Ph), 43.9 (NH*C*H$_2$-Ph); ***m/z*** (ESI$^+$) 91 ($C_7H_7^+$ 41%), 297 (14%), 346 (100%), 347 (24%), 348 (31%), 349 (8%), 497 ([M(^{35}Cl)+H]$^+$ 63%), 498 ([M(^{13}C,^{35}Cl)+H]$^+$ 16%), 499 ([M(^{13}C$_2$,^{35}Cl)+H]$^+$, [M(^{37}Cl)+H]$^+$ 16%), 500 ([M(^{13}C,^{37}Cl)+H]$^+$ 5%), 519 ([M(^{35}Cl)+Na]$^+$ 58%), 520 ([M(^{13}C,^{35}Cl)+Na]$^+$ 19%), 521 ([M(^{13}C$_2$,^{35}Cl)+Na]$^+$, [M(^{37}Cl)+Na]$^+$ 18%), 522 ([M(^{13}C,^{37}Cl)+Na]$^+$ 6%); **HRMS** (ESI$^+$) *m/z* calcd for [M(^{35}Cl)+Na]$^+$ $C_{30}H_{25}ClN_2O_3Na$ 519.1446, found 519.1441 (−1.0 ppm).

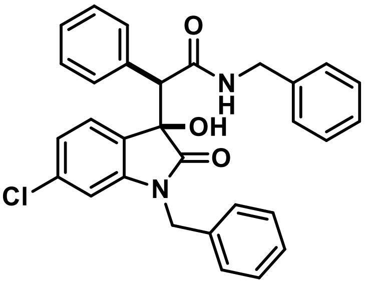

(±)-anti-**15**

PDA Ch1 211nm

Peak#	Ret. Time	Area%
1	18.023	49.994
2	25.602	50.006
Total		100.000

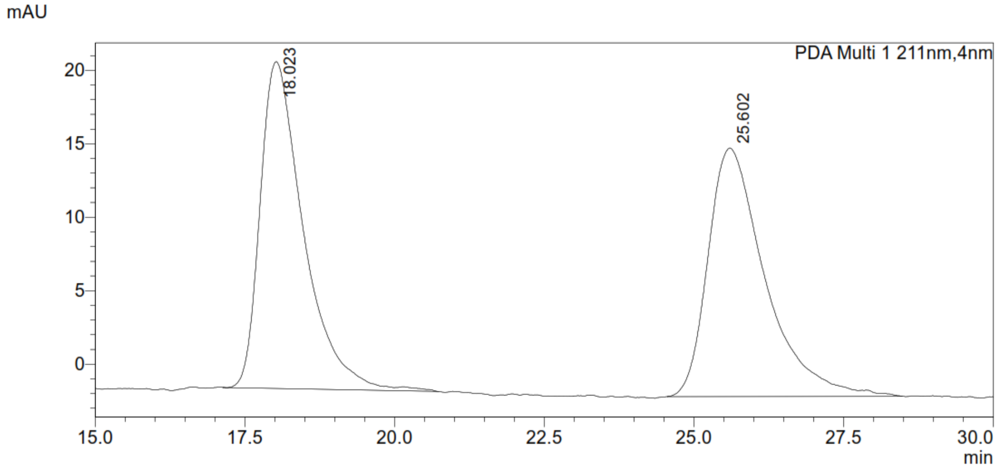

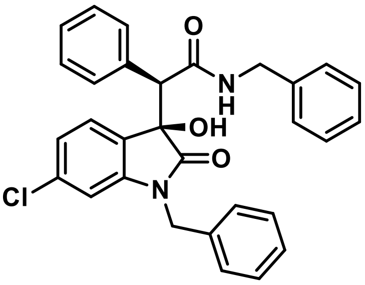

(–)-(2''''*RS*,3*R*)-**15**

PDA Ch1 211nm

Peak#	Ret. Time	Area%
1	18.020	99.414
2	25.550	0.586
Total		100.000

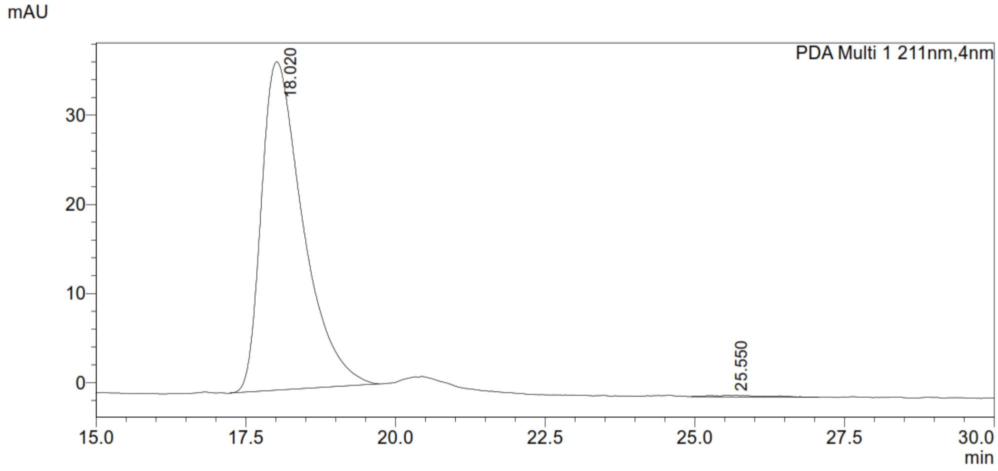

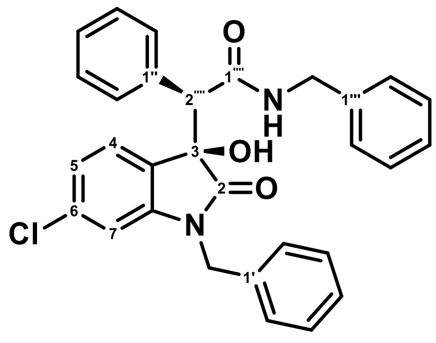

(2''''*R*,3*R*)-**15**

^{1}H NMR (100 MHz, $CDCl_3$)

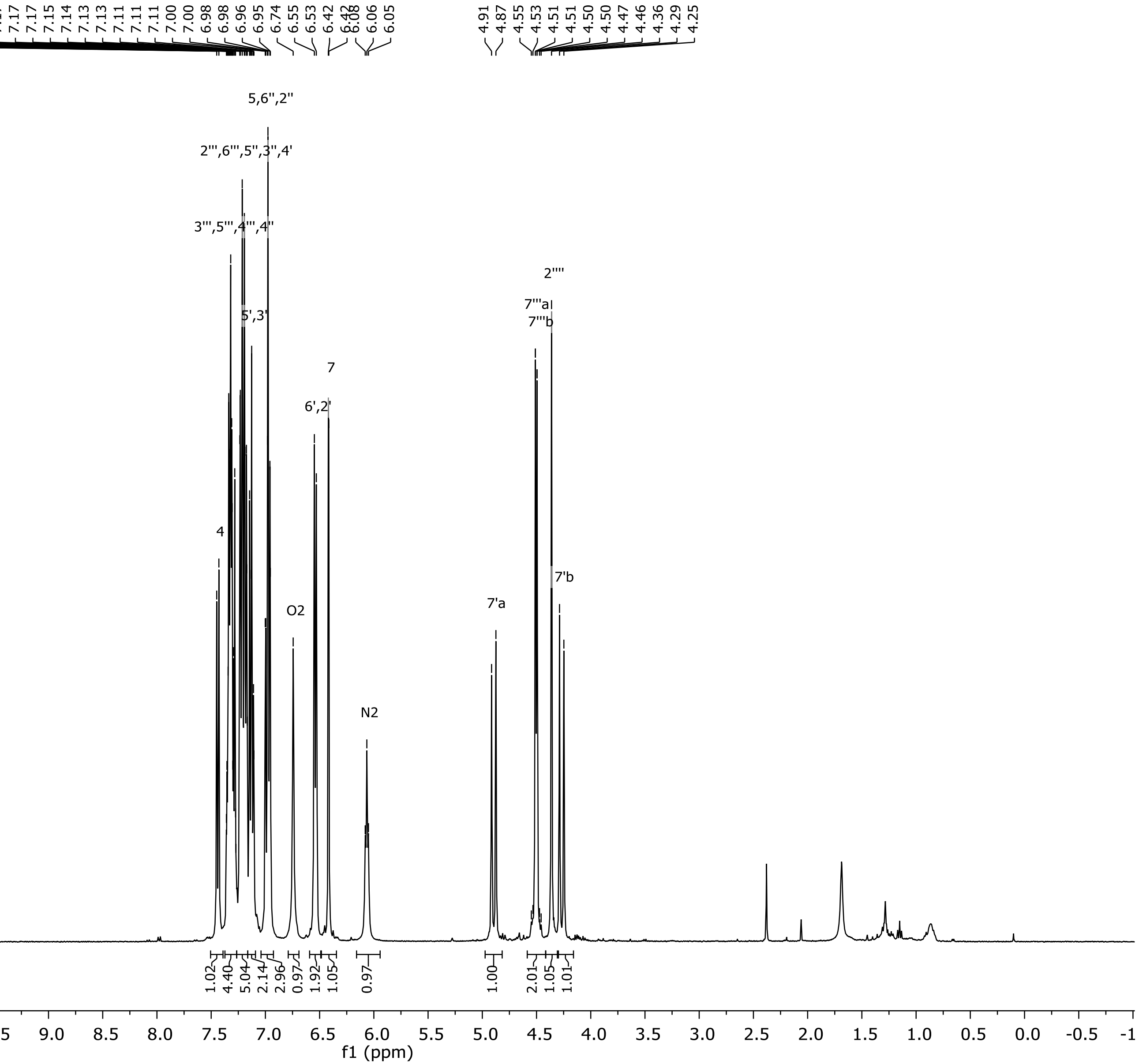

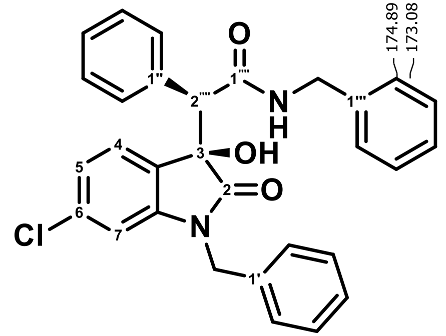

(2''''*R*,3*R*)-**15**

$^{13}C\ \{^{1}H\}$ NMR (100 MHz, $CDCl_3$)

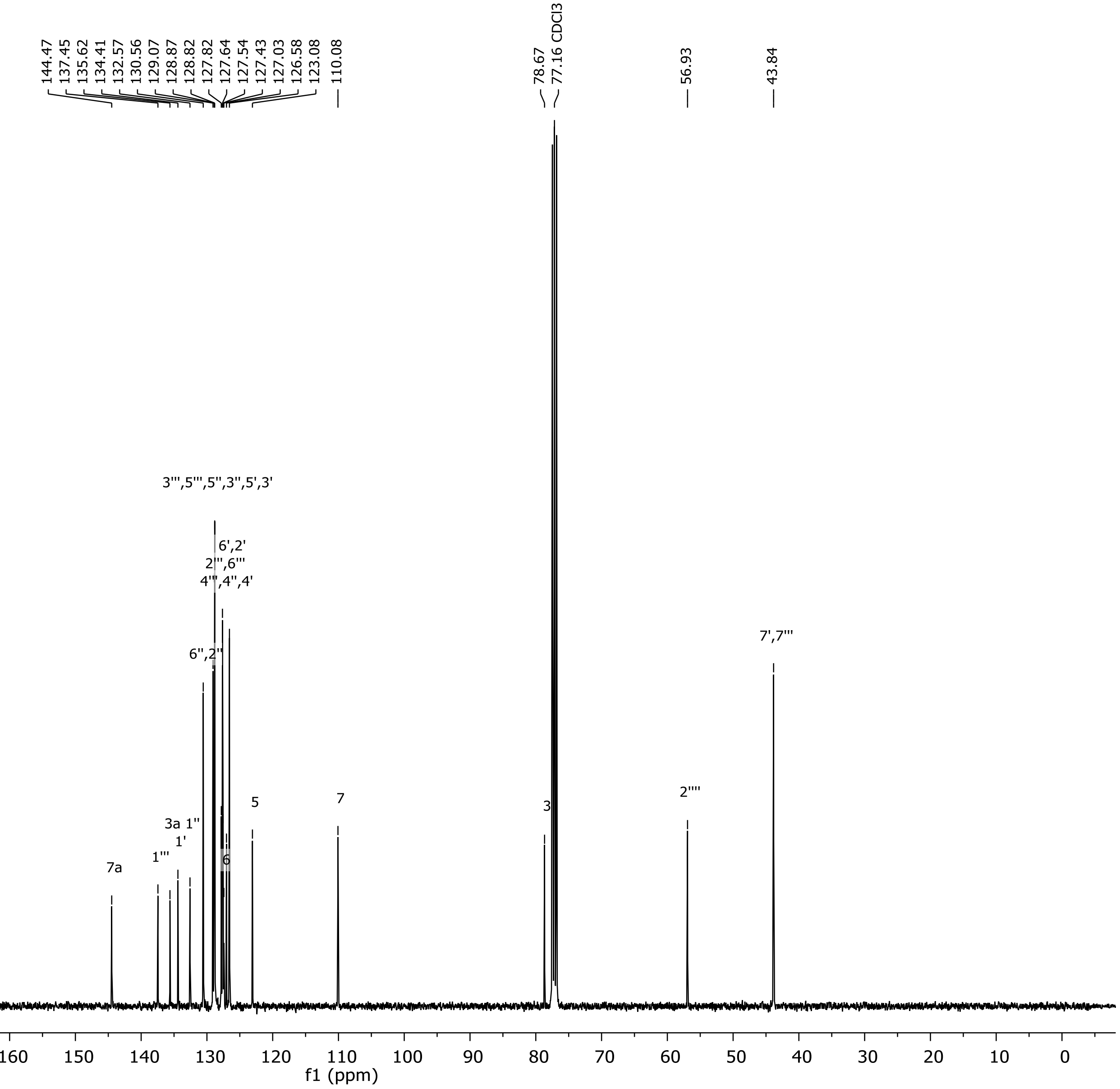

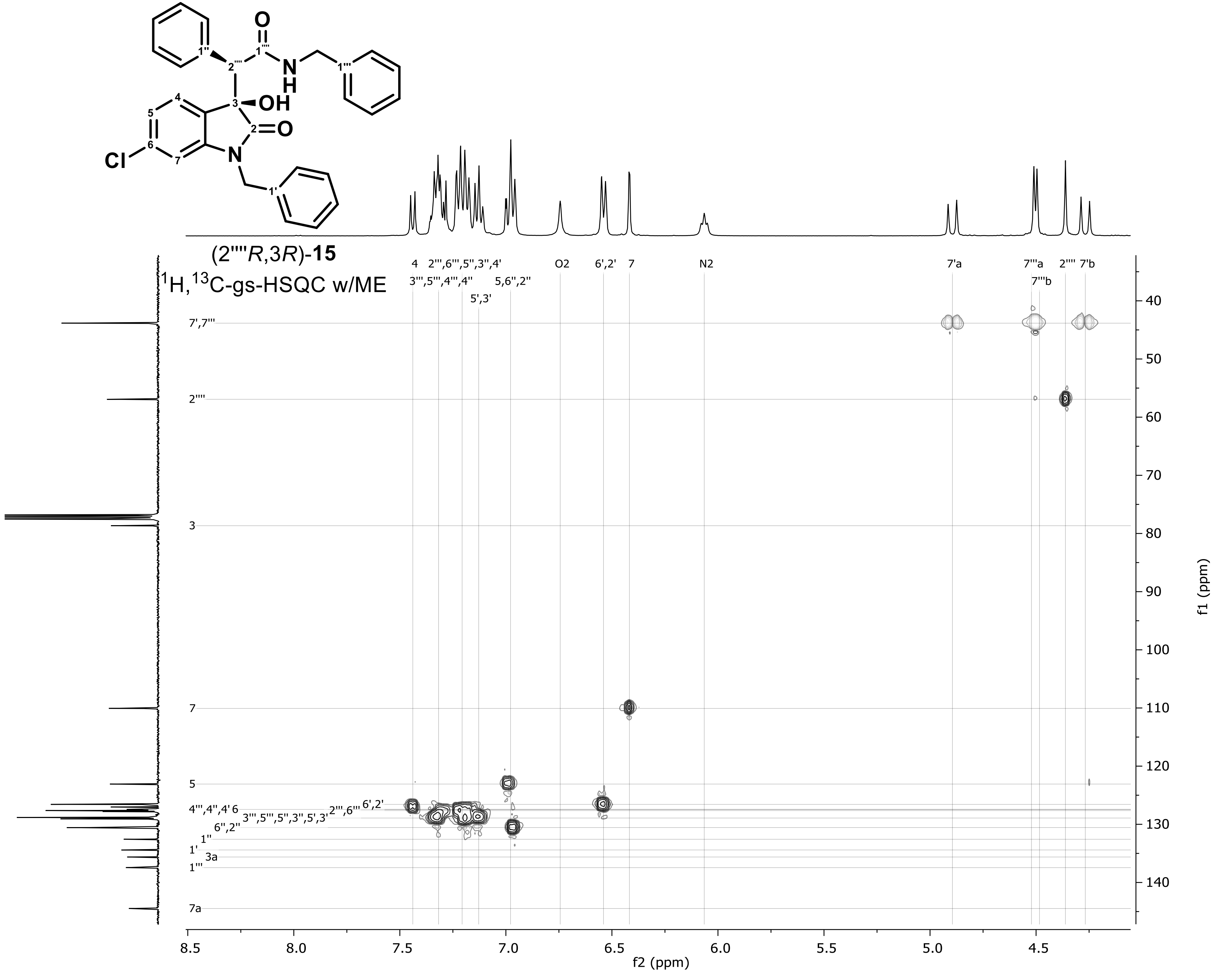

(2''''R,3R)-15
^{1}H,^{13}C-gs-HSQC w/ME
4
3''',5''',4''',4''
2''',6''',5'',3'',4'
5',3'
5,6'',2''
O2
6',2'
7
N2
7'a
7'''a
7'''b
2''''
7'b
7',7'''
2''''
3
7
5
4''',4'',4' 6
6'',2''
3''',5''',5'',3'',5',3'
2''',6'''
6',2'
1''
1'
3a
1'''
7a
f1 (ppm)
f2 (ppm)

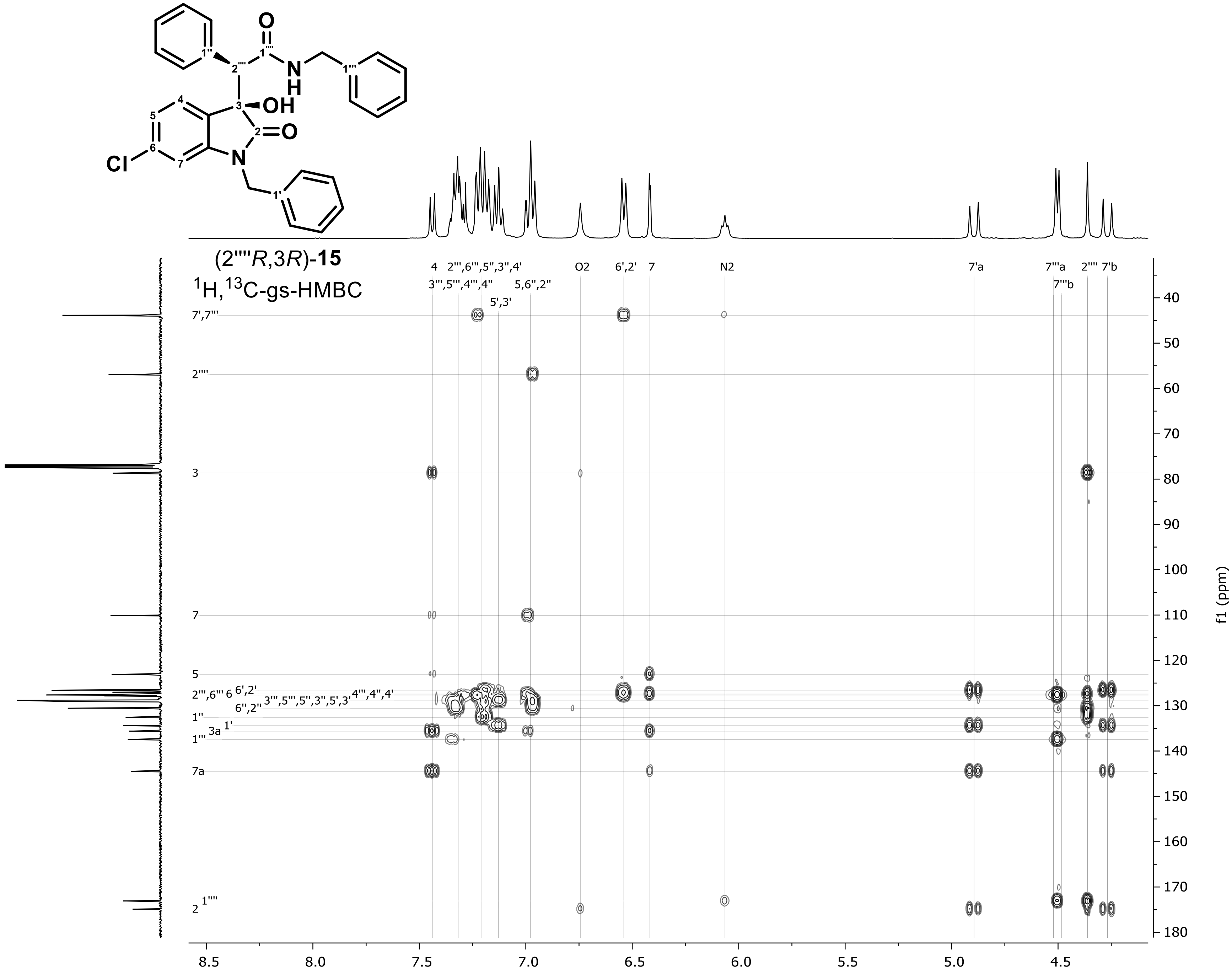

(2''''R,3R)-15
^1H,^{13}C-gs-HMBC
f2 (ppm)
f1 (ppm)

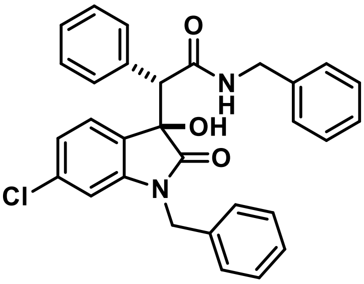

(±)-syn-**15**

PDA Ch1 211nm

Peak#	Ret. Time	Area%
1	20.264	49.001
2	26.679	50.999
Total		100.000

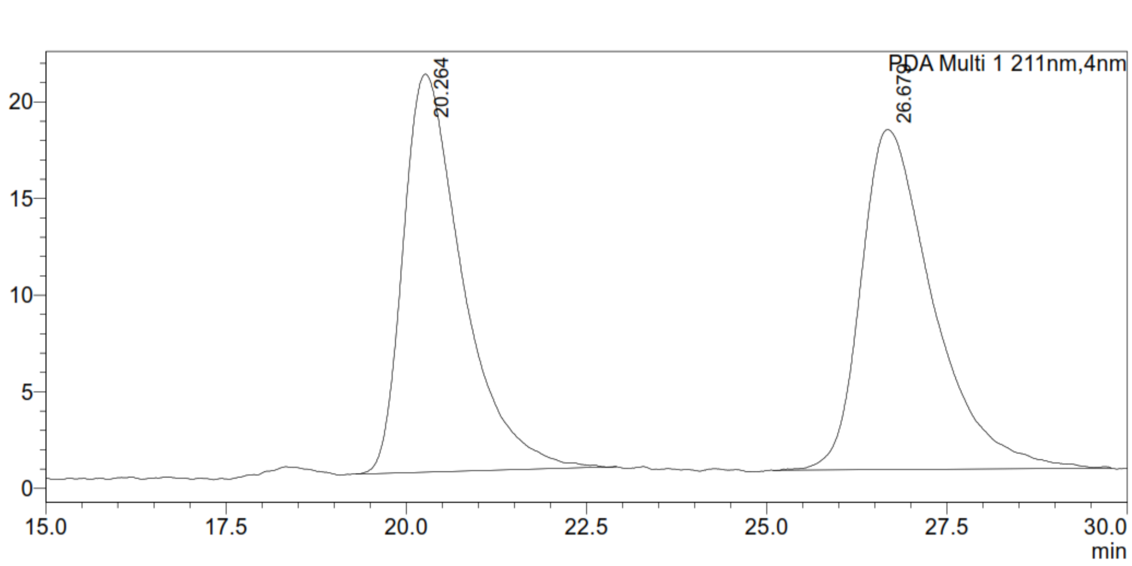

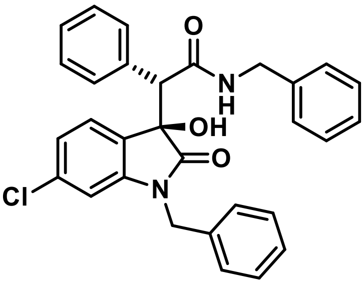

(–)-(2'''S,3R)-**15**

PDA Ch1 211nm

Peak#	Ret. Time	Area%
1	20.198	99.572
2	26.926	0.428
Total		100.000

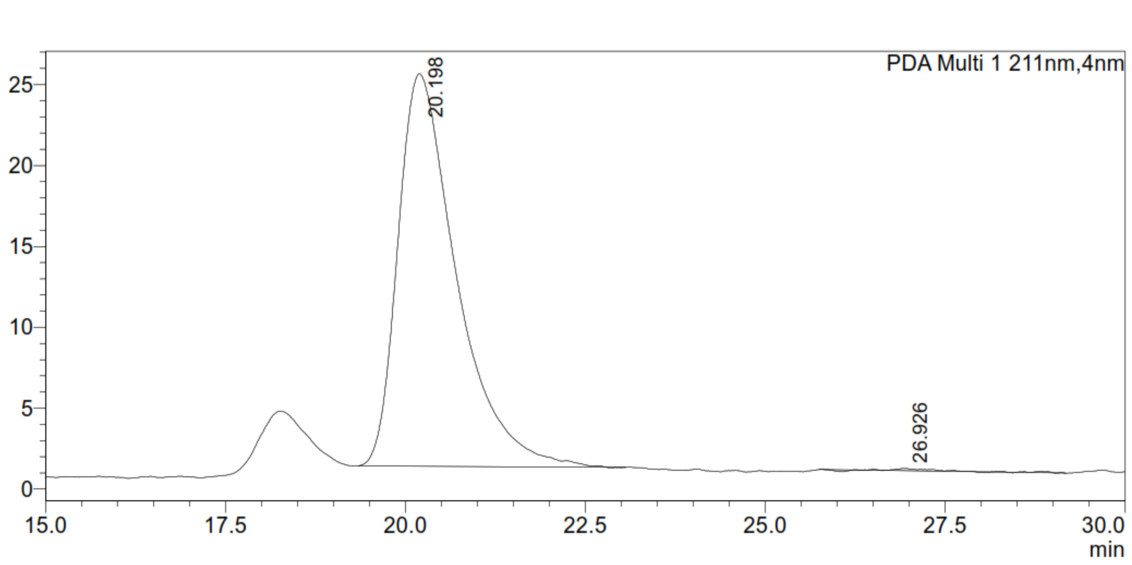

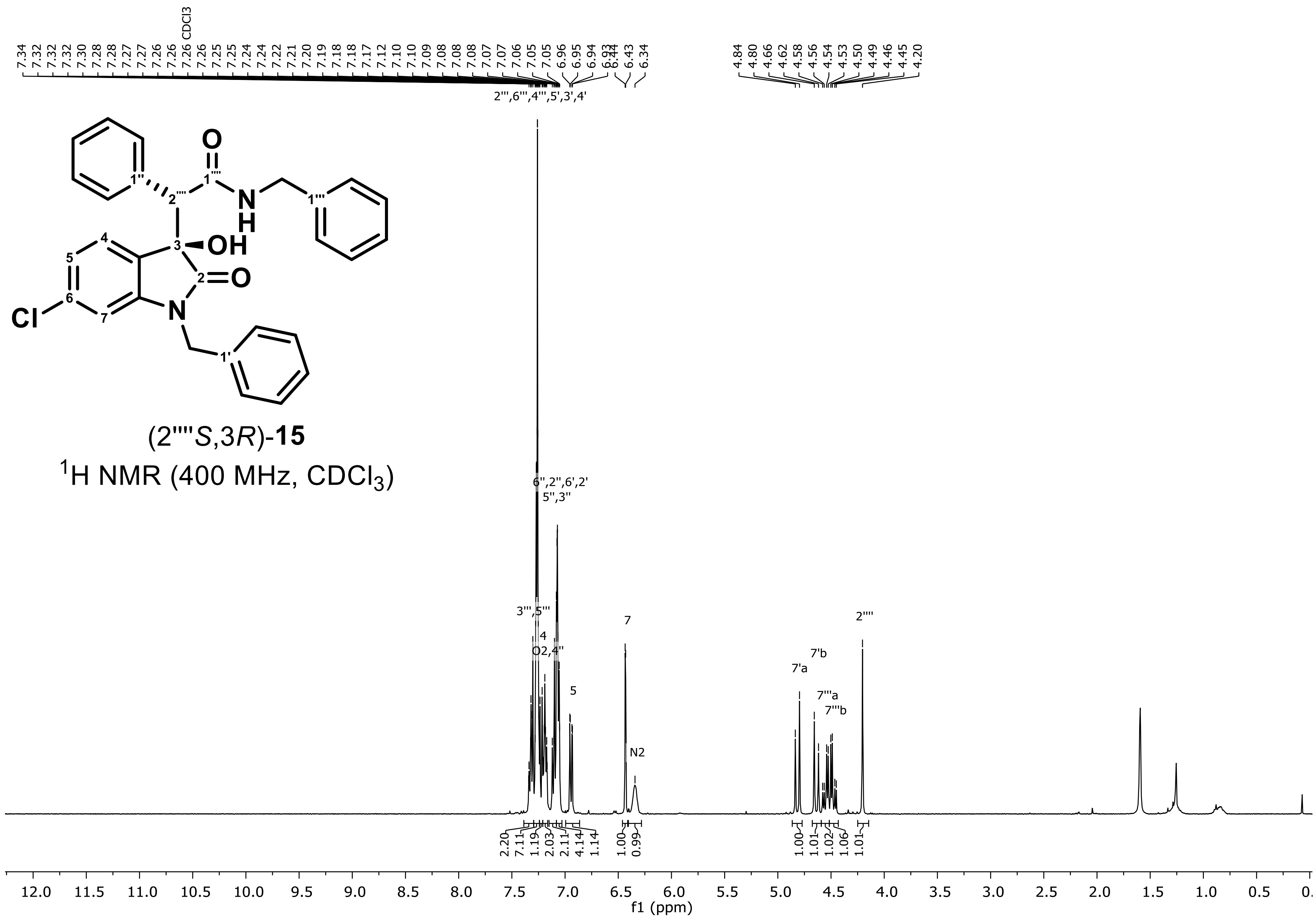

(2''''*S*,3*R*)-**15**

^{1}H NMR (400 MHz, $CDCl_3$)

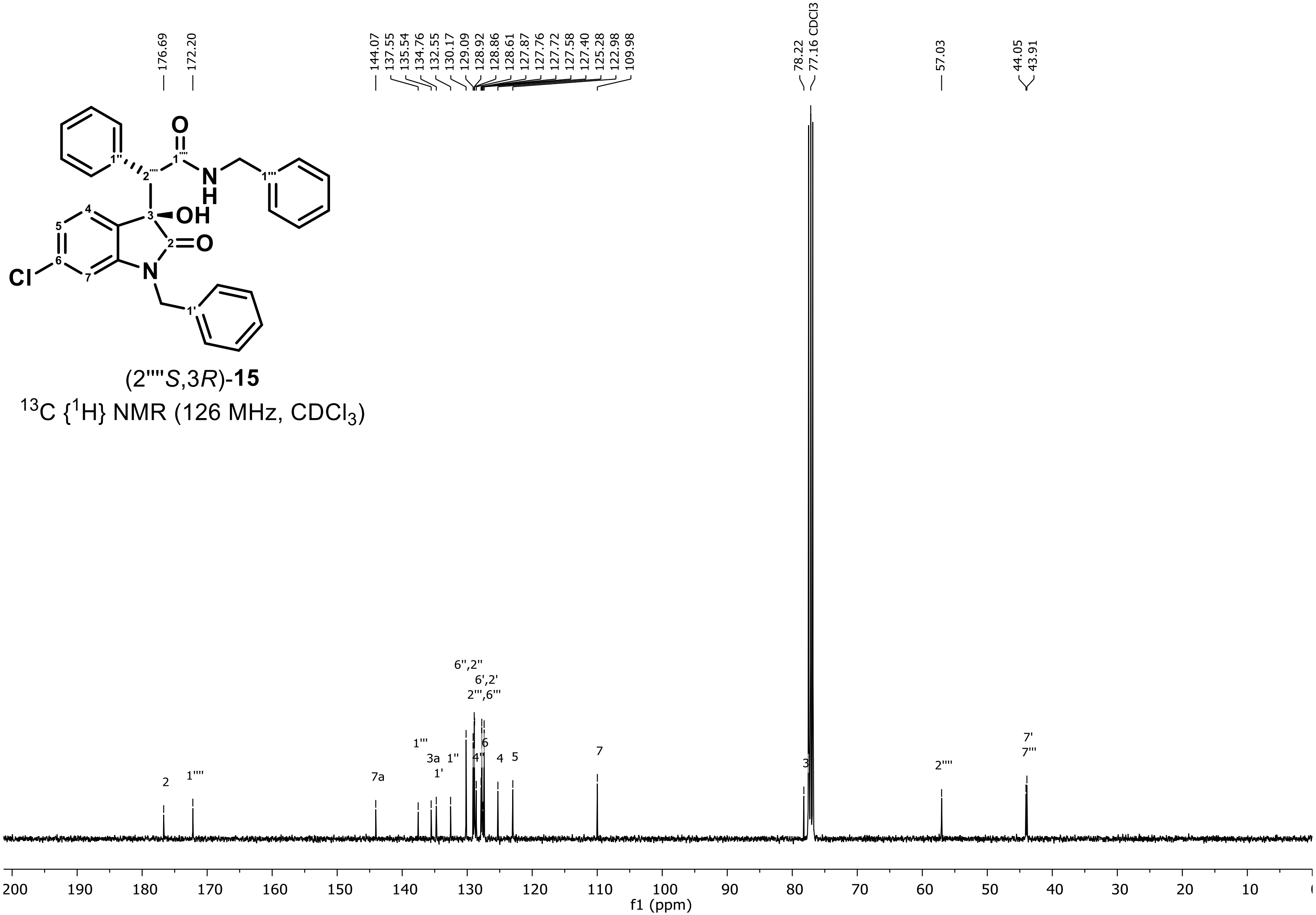

(2''''*S*,3*R*)-**15**

$^{13}C\ \{^1H\}$ NMR (126 MHz, $CDCl_3$)

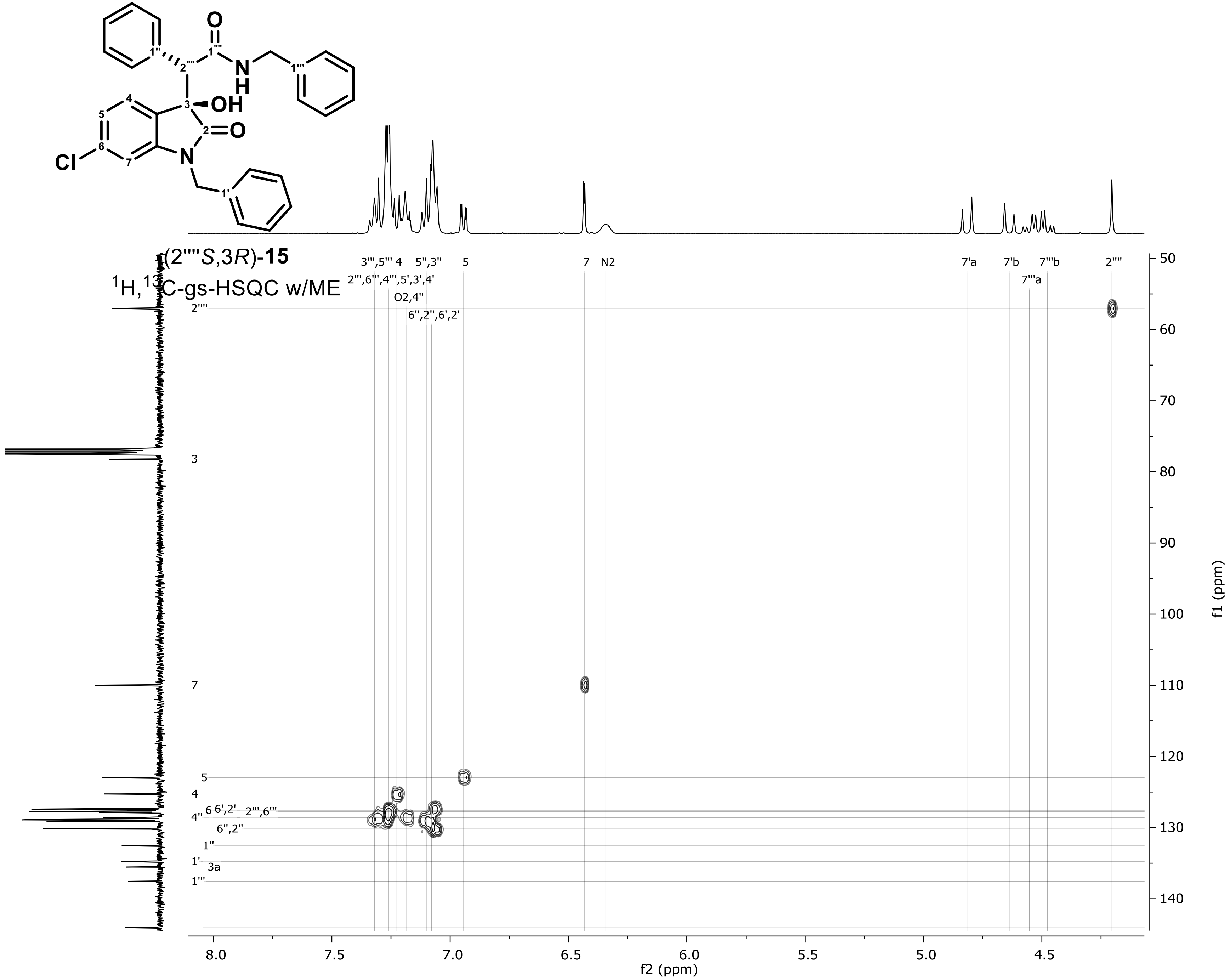
(2''''S,3R)-15
^{1}H,^{13}C-gs-HSQC w/ME
f2 (ppm)
f1 (ppm)

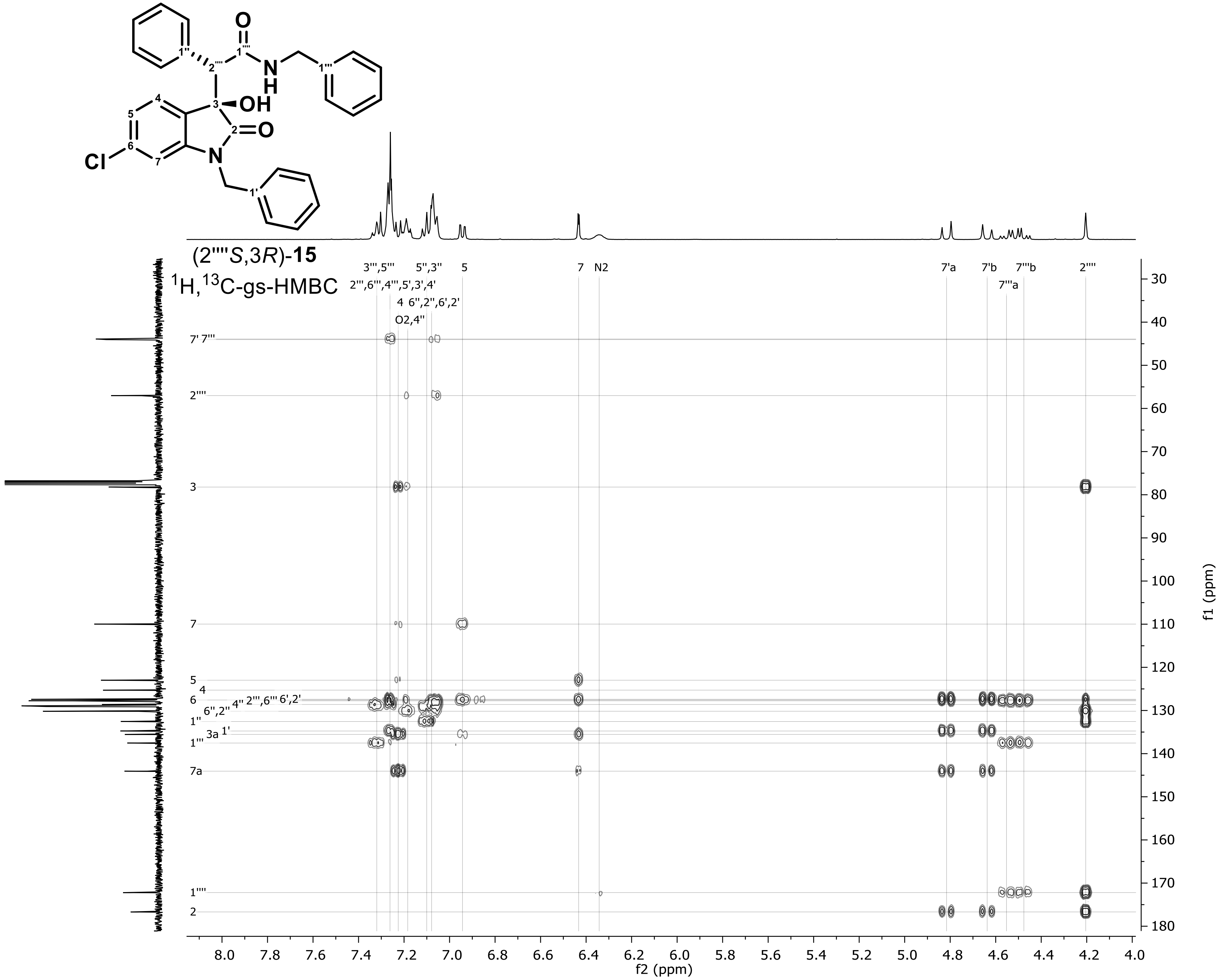
(2''''S,3R)-15
1H,13C-gs-HMBC
f2 (ppm)
f1 (ppm)

j) *N*-benzyl-2-(1-benzyl-5-chloro-3-hydroxy-2-oxoindolin-3-yl)-2-phenylacetamide (16)

To a 25 ml round bottomed flask was added phenylacetic anhydride (95.3 mg, 0.375 mmol), *N*-allylisatin (68.0 mg, 0.250 mmol), and (2*S*,3*R*)-HyperBTM (3.9 mg, 0.012 mmol). The mixture was cooled to 0 °C and CH_2Cl_2 (6.0 ml, 0.04 M) and Hünig's base (54 µl, 0.312 mmol) were added. The mixture was stirred at 0 °C for 3 h. Benzylamine (82 µl, 0.750 mmol) was added and them reaction was left to be stirred overnight at room temperature. 1,3,5-Trimethoxybenzene (0.1 M soln in CH_2Cl_2 500 µl, 0.05 mmol) and the solvent was removed under reduced pressure. Purification by column chromatography (Petrol:EtOAc 9:1 ⟶ 0:10, recolumned Petrol:Et_2O 5:5 ⟶ 4:6) gave the title compound as mixture of diastereomers (74:26 d.r., 118.2 mg, 0.218 mmol, 87%).

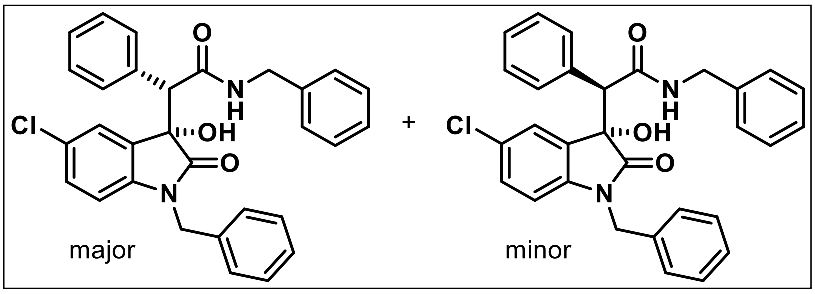

Major (2''''*S*,3*S*)-**16** and **minor** (2''''*R*,3*S*)-**16** analysed as 81:19 mixture of diastereomers. Not all signals are resolved. **m.p.** 82 – 84 °C (ee); ***R_f*** 0.35, 0.30 (Hexane : EtOAc 6:4); $\boldsymbol{\alpha_D^{20}}$ = −95.5 (*c* 1.08, $CHCl_3$); **Chiral HPLC analysis** Chiralpak IB (95:5 hexane:IPA, flow rate 2 ml·min^{-1}, 211 nm, 40 °C) t_R (2''''*S*,3*R*)-**16**: 17.2 min, t_R (2''''*R*,3*S*)-**16**: 43.4 min, <1:99 e.r.; t_R (2''''*R*,3*R*)-**16**: 20.4 min, t_R (2''''*S*,3*S*)-**16**: 26.2 min, <1:99 e.r.; $\boldsymbol{\nu_{max}}$ (thin film): 3339 (m (br), O-H), 3088 (w, C-H), 3063 (w, C-H), 3030 (w, C-H), 2922 (w, C-H), 1713 (s, C=O ketone), 1668 (m), 1645 (s, C=O amide), 1609 (s), 1539 (m), 1526 (m), 1495 (m), 1483 (s), 1454 (s), 1435 (s), 1373 (w), 1344 (s), 1260 (w), 1225 (w), 1171 (s), 1128 (w), 1105 (w), 1078 (m), 1028 (m), 957 (w), 907 (s), 847 (w), 814 (m), 777 (w); **^{1}H NMR** (500 MHz, $CDCl_3$) δ_H 7.62 (0.8H, d, $^4J_{HH}$ = 2.1 Hz, ArC^4*H*), 7.35 – 7.29 (2.6H, m, incl. PhC$^{3''',5'''}$*H* major, ArC^4*H* minor), 7.29 – 7.18 (3.6H, m, incl. Ph$^{2''',6'''}$*H* major), 7.18 – 7.13 (2.6H, m, incl. PhC$^{3'',5''}$*H* major), 7.12 – 7.05 (1.6H, m, PhC$^{3',5'}$*H*), 7.11 (1.6H, dd, $^3J_{HH}$ = 8.4 Hz, $^4J_{HH}$ = 2.1 Hz, ArC^6*H*), 7.03 – 6.99 (0.4H, m), 7.02 (0.2H, dd, $^2J_{HH}$ = 8.3 Hz, $^3J_{HH}$ = 2.2 Hz, ArC^6*H*), 6.95 (1.6H, app d, $^3J_{HH}$ = 7.5 Hz, PhC$^{2'',6''}$*H*), 6.85 (0.8H, s, O*H*), 6.61 (0.2H, s(br), N*H*), 6.48 (1.6H, app d, $^3J_{HH}$ = 7.4 Hz, PhC$^{2',6'}$*H*), 6.34 (0.2H, d, $^3J_{HH}$ = 8.4 Hz, ArC^7*H*), 6.30 (0.8H, d, $^3J_{HH}$ = 8.4 Hz, ArC^7*H*), 6.15 – 6.05 (0.8H, m, N*H*), 4.87 (0.8H, d, $^2J_{HH}$ = 16.0 Hz, NC$H_a$$H_b$-Ph), 4.81 (0.2H, d, $^2J_{HH}$ = 15.8 Hz, NC$H_a$$H_b$-Ph), 4.62 (0.2H, d, $^2J_{HH}$ = 15.98 Hz, NC$H_a$$H_b$-Ph), 4.54 (0.8H, dd, $^2J_{HH}$ = 15.0 Hz, $^3J_{HH}$ = 6.0 Hz, NHC$H_a$$H_b$-Ph), 4.51 – 4.46 (0.4H, m, NHC$H_a$$H_b$-Ph), 4.46 (0.8H, dd, $^2J_{HH}$ = 15.0 Hz, $^3J_{HH}$ = 5.8 Hz, NHC$H_a$$H_b$-Ph), 4.39 (0.8H, s, C*H*-Ph), 4.25 (0.8H, d, $^2J_{HH}$ = 16.0 Hz, NC$H_a$$H_b$-Ph), 4.24 (0.2H, s, C*H*-Ph); **^{13}C {^{1}H} NMR** (126 MHz, $CDCl_3$) δ_C 176.3 (*C*(O)NBn, minor), 174.5 (C(O)NBn, major), 173.0 (C(O)NHBn, major), 172.0 (*C*(O)NHBn, minor), 141.7 (Ar*C*^{7a}, major), 141.2 (Ar*C*^{7a}, minor), 137.5 (Ph*C*$^{1'''}$$CH_2$NH, minor), 137.4 (Ph*C*$^{1'''}$$CH_2$NH, major), 134.8 (Ph*C*$^{1'}$$CH_2$N, minor), 134.5 (Ph*C*$^{1'}$$CH_2$N, major), 132.4$_8$ (Ph*C*$^{1''}$CH, minor), 132.4$_5$ (Ph*C*$^{1''}$CH, major), 130.9 (Ar*C*^{3a}, minor), 130.7 (Ph*C*$^{2'',6''}$H, minor), 130.6 (Ph*C*$^{2'',6''}$H, major), 130.2 (Ar*C*^{3a}, major), 128.9$_9$, 129.0$_2$ (major), 128.9$_3$ (major), 128.8 (major), 128.7 (Ph*C*$^{3',5'}$H, major), 128.6 (Ar*C*), 128.5 (Ar*C*), 128.4 (Ar*C*), 127.7$_8$ (Ar*C*), 127.7$_6$ (Ar*C*), 127.6$_8$ (Ar*C*), 127.6$_4$ (Ar*C*), 127.5$_5$ (Ar*C*), 127.4$_6$ (Ar*C*), 127.3 (Ar*C*), 126.6 (Ph*C*$^{2',6'}$H, major), 126.5 (Ar*C*$_4$, major), 124.8 (Ar*C*^{4}H, minor), 110.6 (Ar*C*^{7}H, major), 110.4 (Ar*C*^{7}H, minor), 79.1 (*C*-OH, major), 78.5 (*C*-OH, minor), 57.3 (*C*H-Ph minor), 56.9 (*C*H-Ph, major), 44.0 (N*C*H_2-Ph or NH*C*H_2-Ph of minor), 43.8 (N*C*H_2-Ph and NHCH_2-Ph of major, N*C*H_2-Ph or NH*C*H_2-Ph of minor); ***m/z*** (ESI$^+$) 179 ([M-Bn-2O-BnCOONHBn+2NH_2]$^+$ 5%), 309 ([M-BnNHCOOH-Cl-O+NH]$^+$ 11%), 346 ([M-BnNHCOO]$^+$ 12%), 497 ([M+H]$^+$ 100%), 498 ([M(^{13}C)+H]$^+$ 33%), 499 ([M($^{13}C_2$)+H]$^+$, [M+3H]$^+$ 33%), 500 ([M+3H]$^+$, 10%), 519 ([M+Na]$^+$, 4%), 993 ([2M+H]$^+$ 15%), 1015 ([2M+Na]$^+$ 3%); **HRMS** (ESI$^+$) *m/z* calcd for [M+H]$^+$ $C_{30}H_{26}O_3N_2Cl$ 497.1626, found 497.1614 (−2.5 ppm).

(±)-anti-**16** + (±)-syn-**16**

PDA Ch1 211nm

Peak#	Ret. Time	Area%
1	16.971	9.356
2	19.818	40.496
3	26.468	40.497
4	43.642	9.652
Total		100.000

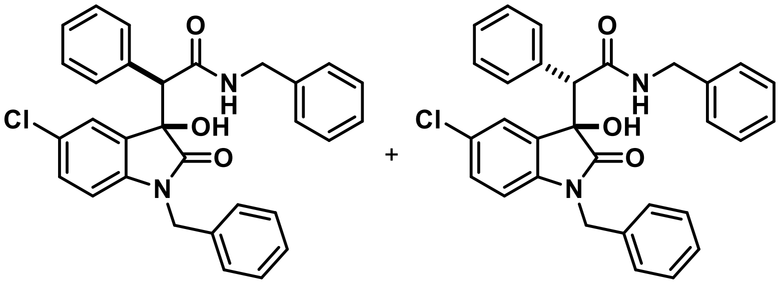

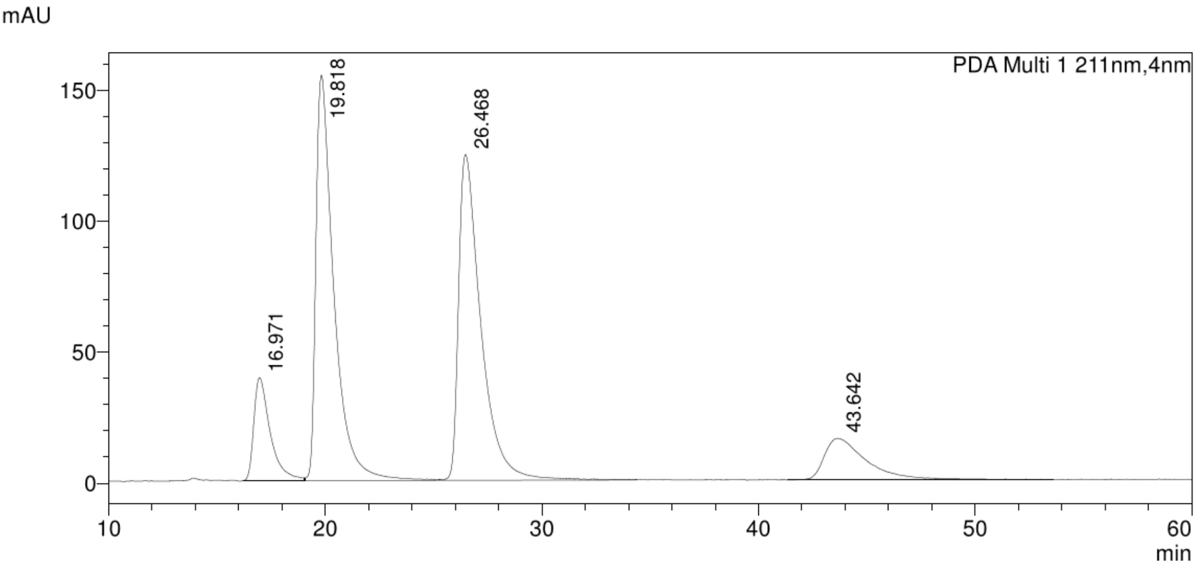

(2''''*S*,3*S*)-**16** + (2''''*R*,3*S*)-**16**

PDA Ch1 211nm

Peak#	Ret. Time	Area%
1	17.216	0.122
2	20.367	0.401
3	26.225	72.998
4	43.396	26.479
Total		100.000

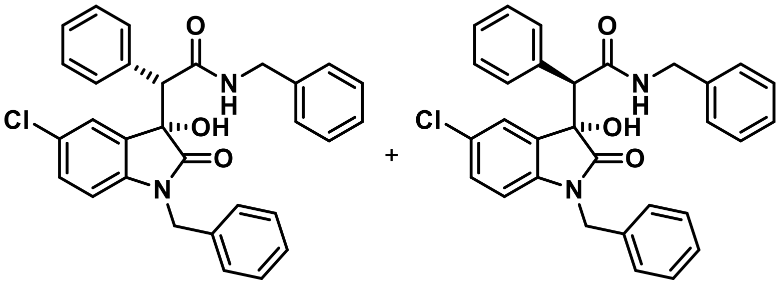

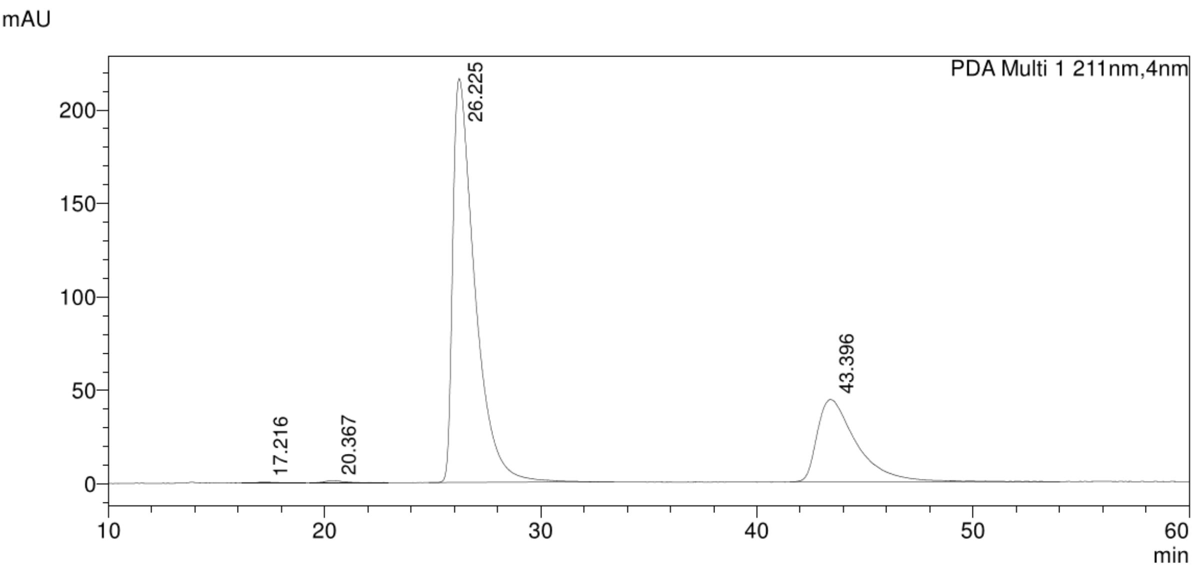

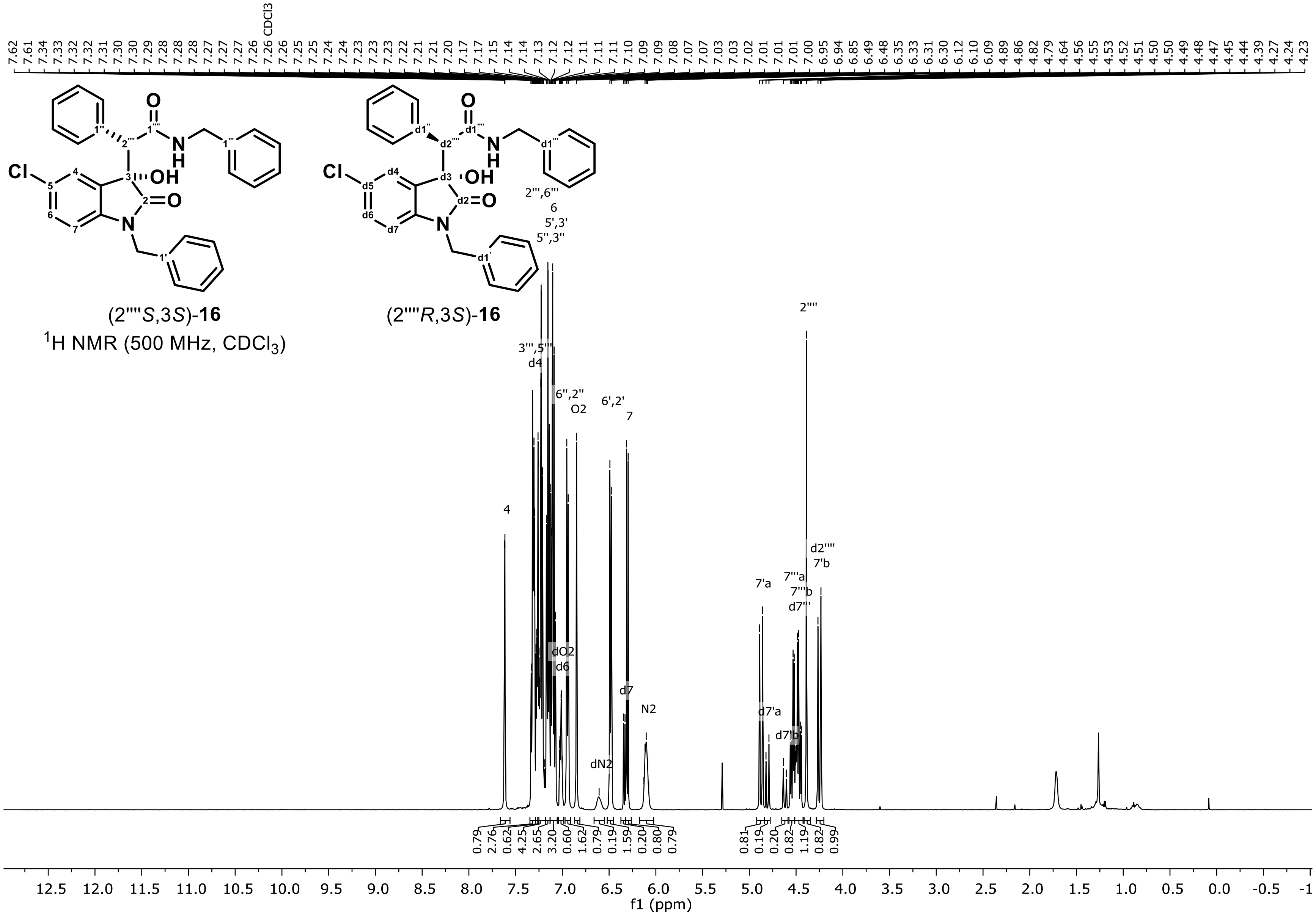

(2''''S,3S)-16
(2''''R,3S)-16
^{1}H NMR (500 MHz, CDCl$_3$)
f1 (ppm)

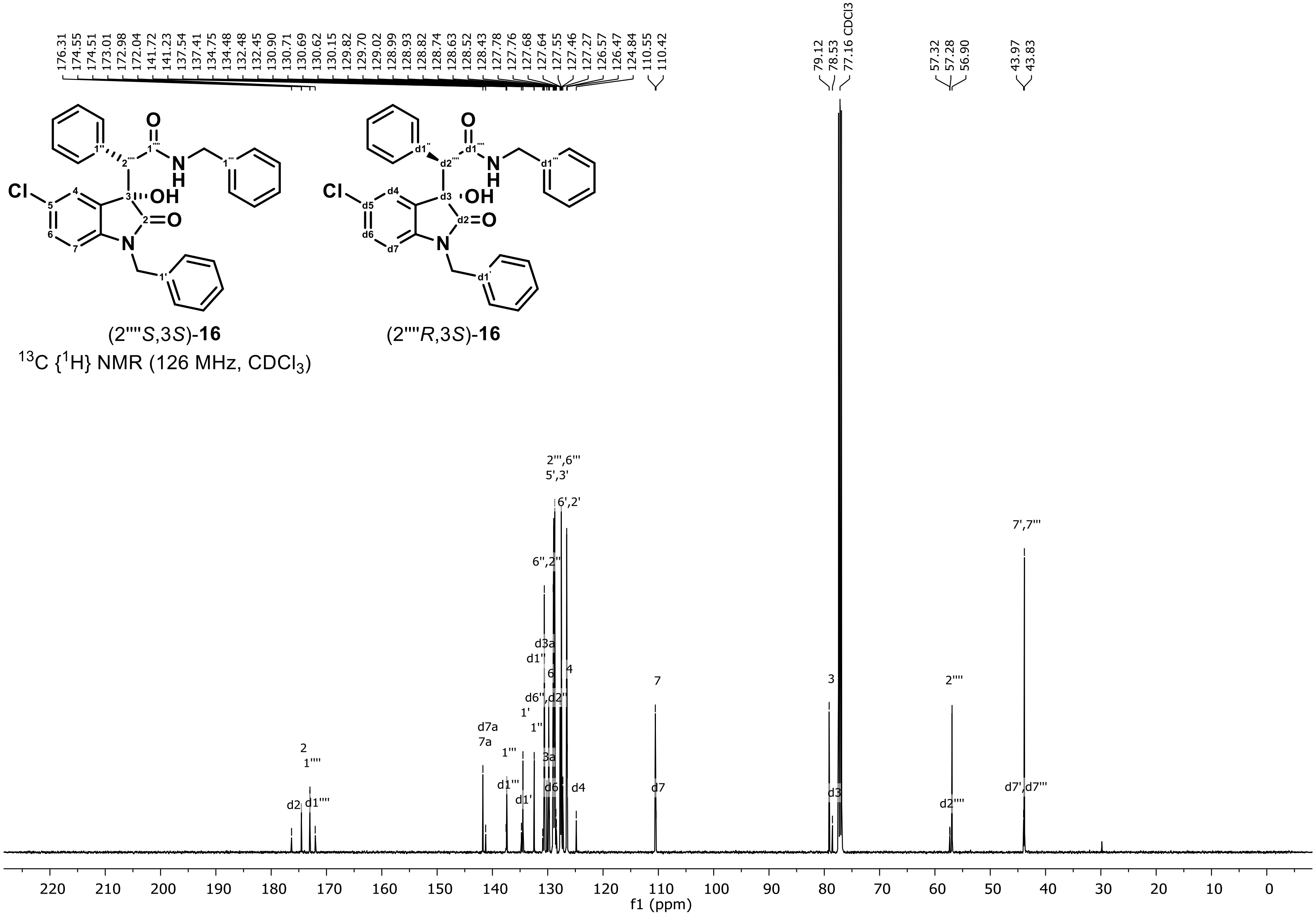

(2''''*S*,3*S*)-**16**

(2''''*R*,3*S*)-**16**

^{13}C {^{1}H} NMR (126 MHz, $CDCl_3$)

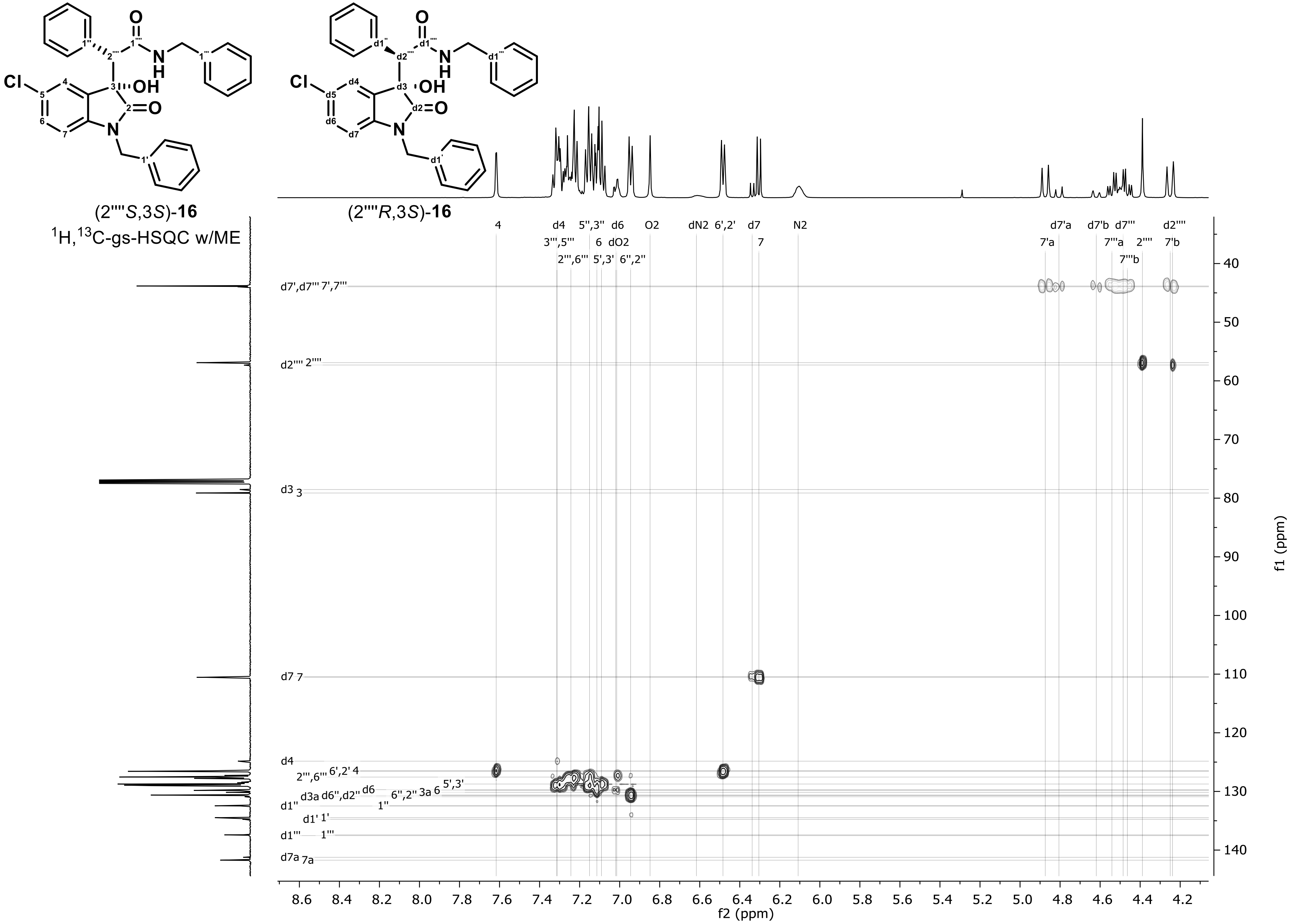

(2''''S,3S)-16
(2''''R,3S)-16
$^1H,^{13}C$-gs-HSQC w/ME
f2 (ppm)
f1 (ppm)

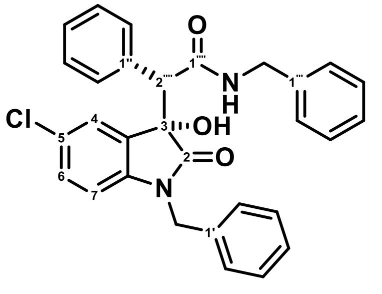

(2''''S,3S)-**16**

$^{1}H,^{13}C$-gs-HMBC

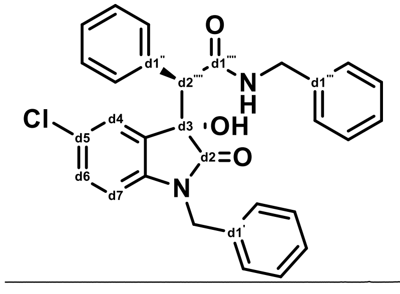

(2''''R,3S)-**16**

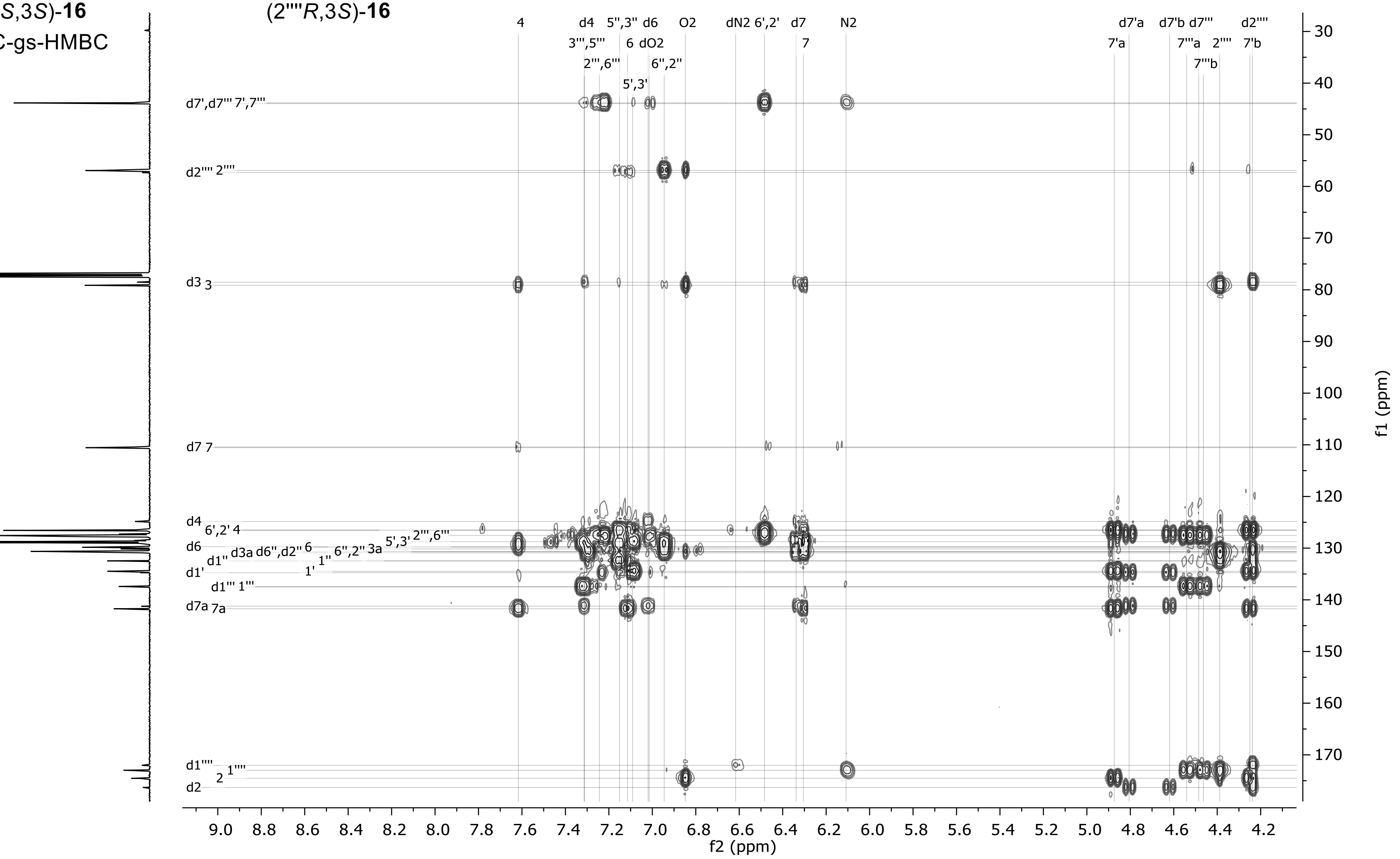

k) ***N*,*N'*-dibenzyl-2-(5-bromo-3-hydroxy-2-oxoindolin-3-yl)-2-phenylacetamide (17)**

To a 25 ml round bottomed flask was added phenylacetic anhydride (95.3 mg, 0.375 mmol), *N*-benzyl-5-bromoisatin (79.0 mg, 0.250 mmol), and (2*S*,2*R*)-HyperBTM (3.9 mg, 0.012 mmol). The mixture was cooled to 0 °C and CH_2Cl_2 (6.0 ml, 0.04 M) and Hünig's base (54 µl, 0.312 mmol) were added. The mixture was stirred at 0 °C for 3 h. Benzylamine (82 µl, 0.750 mmol) was added and the reaction was left to be stirred overnight at room temperature. 1,3,5-trimethoxybenzene (0.1 M soln in CH_2Cl_2 500 µl, 0.05 mmol) was added and the solvent was removed under reduced pressure. Purification by column chromatography (Hexane:Et_2O 6:4 → Hexane:Acetone 9:1 → 3:2) gave the title compound as white solid (72:28. d.r., 108.5 mg, 0.218 mmol, 87%).

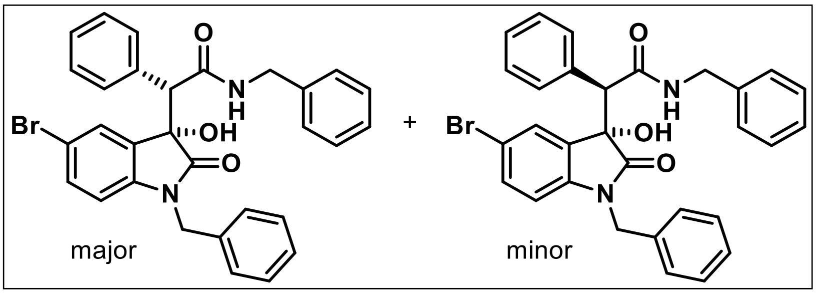

Major (2''''*S*,3*S*)-**17** and **minor** (2''''*R*,3*S*)-**17** analysed as 70:30 mixture of diastereomers. Not all signals are resolved. **m.p.** 178 °C (rac) $\mathbf{R_f}$ 0.14 (Hexane:Acetone 7:3); $\boldsymbol{\alpha_D^{20}}$ = +110.0 (*c* 4.5, $CHCl_3$); **Chiral HPLC** analysis, Chiralpak IB (95:5 hexane:IPA, flow rate 2 ml·min^{-1}, 211 nm, 40 °C) t_R (2''''*S*,3*R*)-**17**: 18.0 min, t_R (2''''*R*,3*S*)-**17**: 48.4 min, <1:99 e.r.; t_R (2''''*R*,3*R*)-**17**: 21.6 min, t_R (2''''*S*,3*S*)-**17**: 26.4 min, <1:99 e.r.; $\boldsymbol{\nu_{max}}$ (thin film) 3374 (w), 3086 (w), 3065 (w), 3032 (w), 1713 (s, C=O, lactam), 1672 (w), 1645 (m, C=O, amide), 1610 (m), 1541 (w), 1495 (w), 1438 (m), 1454 (m), 1434 (w), 1362 (w), 1346 (m), 1267 (w), 1225 (w), 1175 (m), 1128 (w), 1105 (w), 1080 (w), 1065 (w), 1030 (w), 957 (w), 916 (w), 889 (w), 845 (w), 814 (m), 752 (m); **^{1}H NMR** (500 MHz, $CDCl_3$) δ_H 7.77 (0.7H, d, $^4J_{HH}$ = 2.0 Hz, ArC^4*H*), 7.47 (0.3H, d, $^4J_{HH}$ = 2.0 Hz, ArC^4*H*), 7.39 – 7.09 (13H, m, ArC*H*), 7.07 – 7.02 (0.6H, m, PhC$^{2',6'}$*H*), 6.97 (1.4H, app d, $^3J_{HH}$ = 7.3 Hz, PhC$^{2'',6''}$*H*), 6.58 (0.3H, app td, $^3J_{HH}$ = 7.0 Hz, 5.8 Hz, N*H*), 6.52 (1.4H, app d, $^3J_{HH}$ = 7.4 Hz, PhC$^{2',6'}$*H*), 6.32 (0.3H, d, $^3J_{HH}$ = 8.3 Hz, ArC^7*H*), 6.29 (0.7H, d, $^3J_{HH}$ = 8.3 Hz, ArC^7*H*), 6.08 (0.7H, app q (br), $^3J_{HH}$ = 5.8 Hz, N*H*), 4.90 (0.7H, d, $^2J_{HH}$ = 16.0 Hz, NC*H*$_a$H$_b$-Ph), 4.84 (0.3H, d, $^2J_{HH}$ = 15.8 Hz, NC*H*$_a$H$_b$-Ph), 4.65 (0.3H, d, $^2J_{HH}$ = 15.8 Hz, NCH$_a$*H*$_b$-Ph), 4.59 (0.7H, dd, $^2J_{HH}$ = 14.9 Hz, $^3J_{HH}$ = 6.1 Hz, NHC*H*$_a$H$_b$-Ph), 4.55 (0.3H, dd, $^2J_{HH}$ = 15.5 Hz, $^3J_{HH}$ = 5.8 Hz, NHC*H*$_a$H$_b$-Ph), 4.51 (0.3H, dd, $^2J_{HH}$ = 15.5 Hz, $^3J_{HH}$ = 5.8 Hz, NHCH$_a$*H*$_b$-Ph), 4.48 (0.7H, dd, $^2J_{HH}$ = 14.9 Hz, $^3J_{HH}$ = 5.7 Hz, NHCH$_a$*H*$_b$-Ph), 4.40 (0.7H, s, *C*H-Ph), 4.28 (0.7H, d, $^2J_{HH}$ = 16.0 Hz, NCH$_a$*H*$_b$-Ph), 4.25 (0.3H, s, *C*H-Ph); **^{13}C {^{1}H} NMR** (126 MHz, $CDCl_3$) δ_C 176.2 (*C*(O)NBn, minor), 174.3 (*C*(O)NBn, major), 173.1 (*C*(O)NHBn, major), 172.0 (*C*(O)NHBn, minor), 142.2 (Ar*C*^{7a}, major), 141.7 (Ar*C*^{7a}, minor), 137.6 (Ph*C*$^{1'''}$CH$_2$NH, minor), 137.4 (Ph*C*$^{1'''}$CH$_2$NH, major), 134.8 (Ph*C*$^{1'}$CH$_2$N, minor), 134.5 (Ph*C*$^{1'}$CH$_2$N, major), 132.7 (Ar*C*^{6}H, major), 132.6 (Ar*C*^{6}H, minor), 132.5 (Ar*C*^{3a}, minor), 132.4 (Ar*C*^{3a}, major), 131.2 (Ph*C*$^{1''}$CH, major), 131.1 (Ph*C*$^{1''}$CH, minor), 130.6 (Ph*C*$^{2'',6''}$H, major), 130.2 (Ph*C*$^{2'',6''}$H, minor), 129.1$_1$ (Ar*C*^{4}H), 129.0$_5$ (Ar*C*), 128.9$_9$ (Ar*C*), 128.9$_7$ (Ar*C*), 128.8$_4$ (Ar*C*), 128.8$_2$ (Ar*C*), 128.7 (Ar*C*), 128.5 (Ar*C*), 127.7$_9$ (Ar*C*), 127.7$_5$ (Ar*C*), 127.7$_0$ (Ph*C*$^{2''',6'''}$H, minor), 127.6$_3$ (Ar*C*), 127.5$_9$ (Ar*C*^{4}H, minor), 127.5$_3$ (Ph*C*$^{2''',6'''}$H, major), 127.4$_7$ (Ar*C*), 127.3 (Ph*C*$^{2',6'}$H, minor), 126.6 (Ph*C*$^{2',6'}$H, major), 116.0 (Ar*C*^{5}Br, major), 115.7 (Ar*C*^{5}Br, minor), 111.1 (ArC^7*H*, major), 110.9 (Ar*C*^{7}H, minor), 79.1 (*C*-OH, major), 78.5 (*C*-OH, minor), 57.2 (*C*H-Ph, minor), 56.9 (*C*H-Ph, major), 43.9 (N*C*H$_2$-Ph and NHCH$_2$-Ph, minor), 43.8 (N*C*H$_2$-Ph and NHCH$_2$-Ph, major); ***m/z*** (ESI$^+$) 390 ([M(^{79}Br)-BnNHCOOH+H]$^+$ 40%), 391 ([M(^{13}C, ^{79}Br)-BnNHCOOH+H]$^+$ 10%), 392 ([M(^{81}Br)-BnNHCOOH+H]$^+$ 38%), 393 ([M(^{13}C, ^{81}Br)-BnNHCOOH+H]$^+$ 8%), 541 ([M(^{79}Br)+H]$^+$ 100%), 542 ([M(^{13}C, ^{79}Br)+H]$^+$ 33%), 543 ([M(^{13}C$_2$, ^{79}Br)+H]$^+$, [M(^{81}Br)+H]$^+$ 100%), 544 ([M(^{13}C, ^{81}Br)+H]$^+$ 32%), 545 ([M(^{13}C$_2$, ^{81}Br)+H]$^+$ 5%), 563 ([M(^{79}Br)+Na]$^+$ 8%), 564 ([M(^{13}C, ^{79}Br)+Na]$^+$ 3%), 565 ([M(^{13}C$_2$, ^{79}Br)+Na]$^+$, [M(^{81}Br)+Na]$^+$ 8%), 566 ([M(^{13}C, ^{81}Br)+Na]$^+$ 3%); **HRMS** (ESI$^+$) *m/z* cacld for [M+H]$^+$ $C_{30}H_{26}O_3N_2Br$ 541.1121, found 541.1118 (−0.7 ppm).

(±)-anti-**17** + (±)-syn-**17**
PDA Ch1 211nm

Peak#	Ret. Time	Area%
1	17.503	18.093
2	20.914	32.509
3	26.847	31.875
4	48.104	17.523
Total		100.000

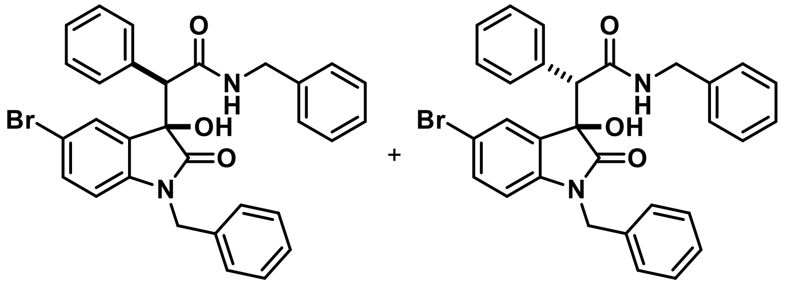

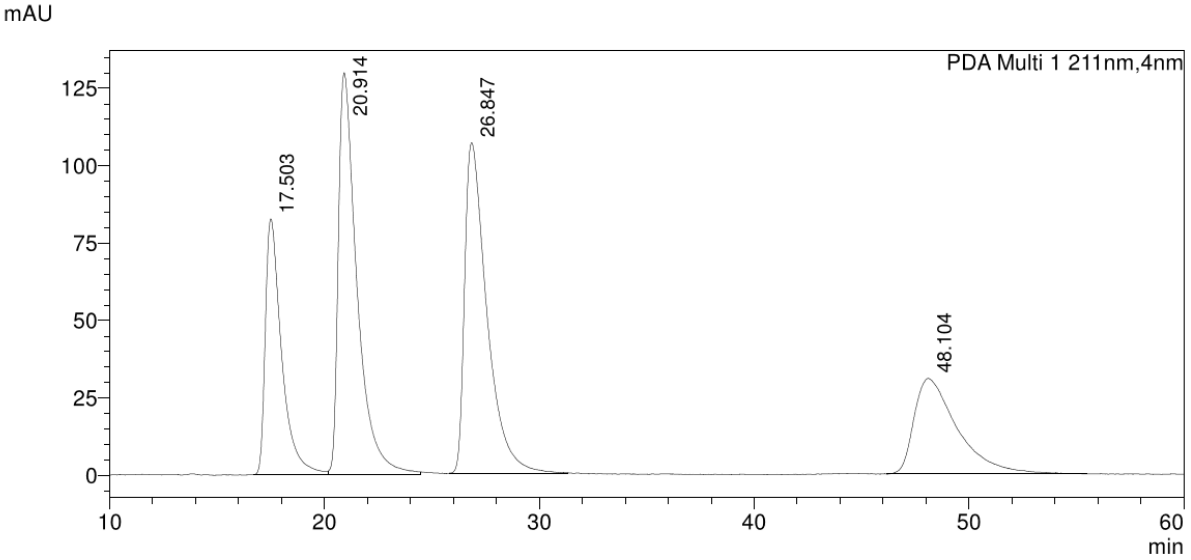

(2''''*S*,3*S*)-**17** + (2''''*R*,3*S*)-**17**
PDA Ch1 211nm

Peak#	Ret. Time	Area%
1	17.992	0.170
2	21.615	0.367
3	26.376	69.972
4	48.398	29.492
Total		100.000

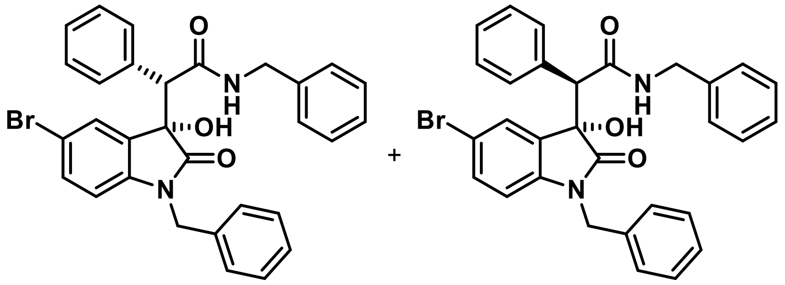

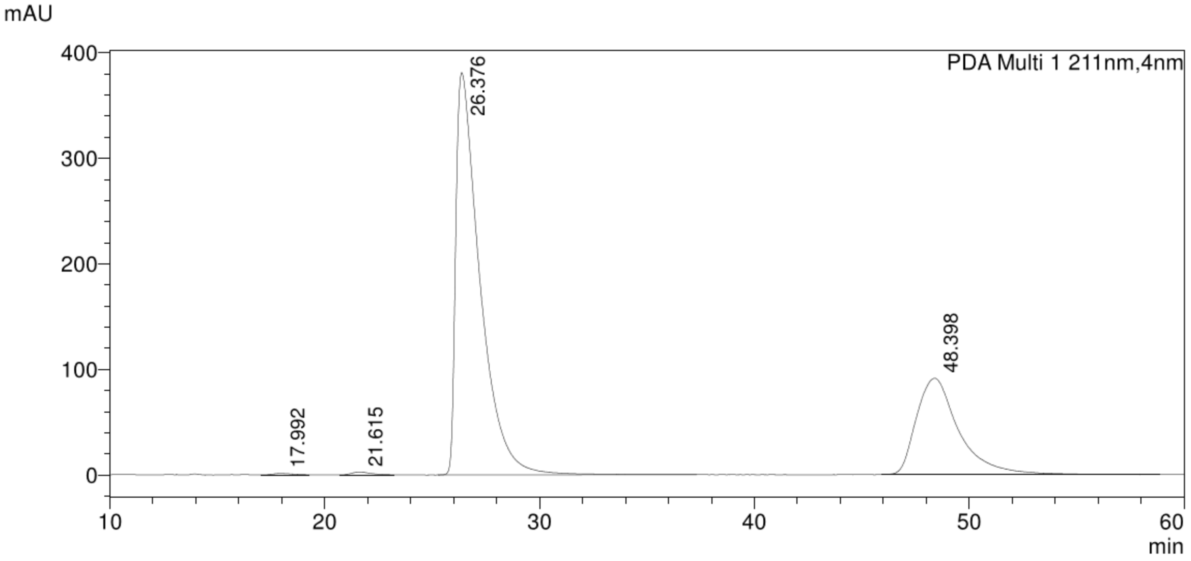

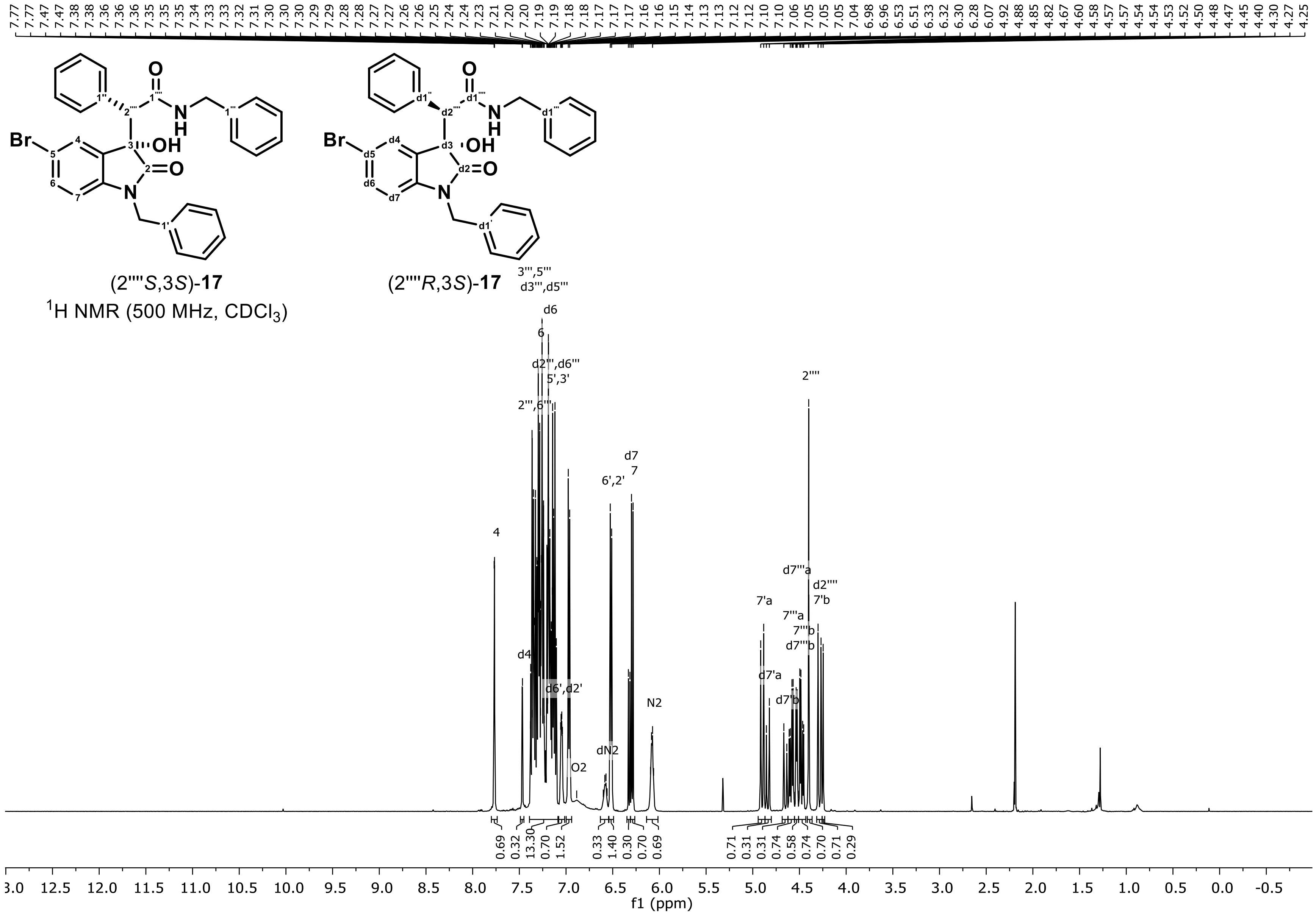
(2''''S,3S)-17
(2''''R,3S)-17
1H NMR (500 MHz, CDCl3)
f1 (ppm)

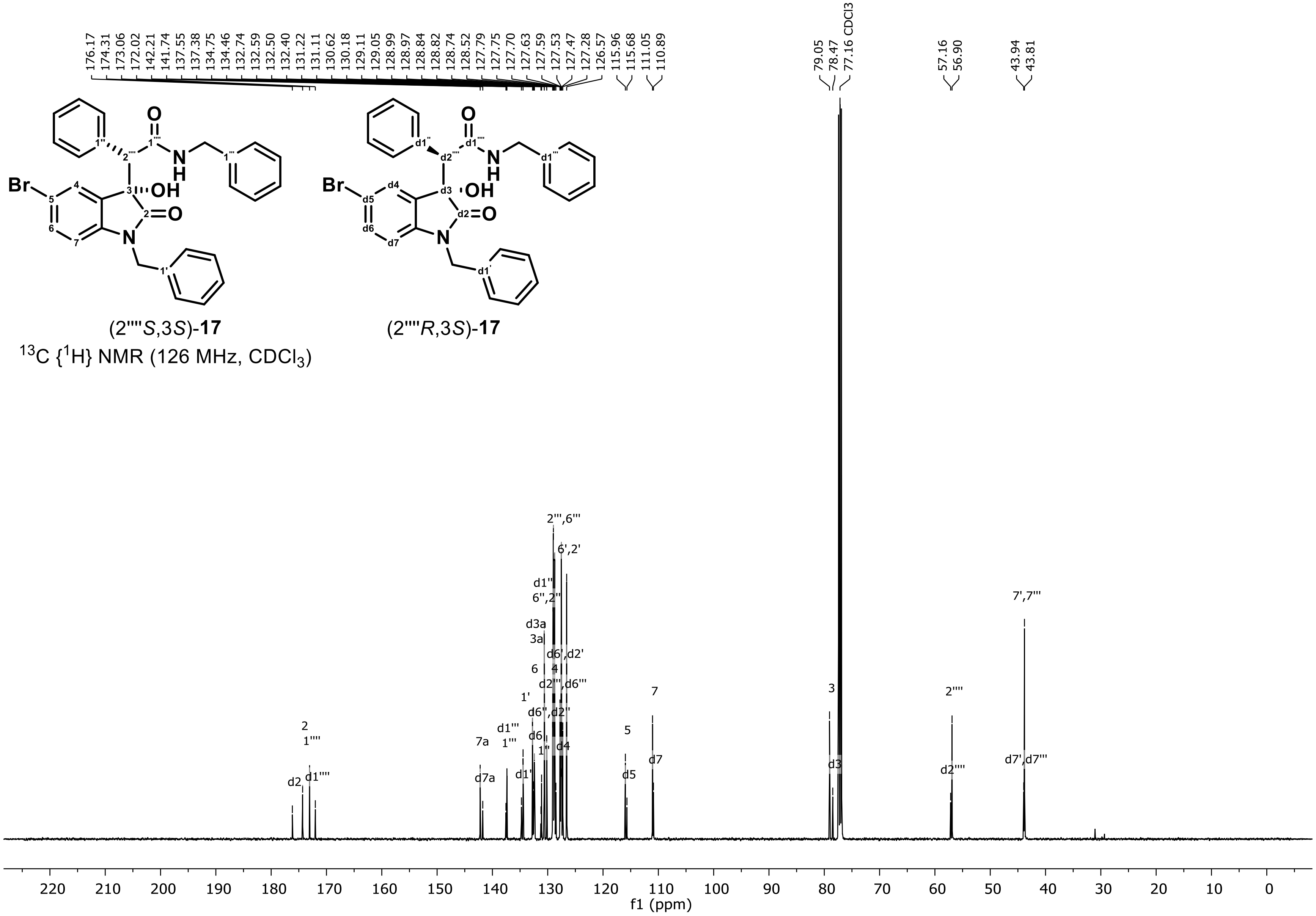

(2''''S,3S)-**17**

(2''''R,3S)-**17**

$^{13}C\ \{^{1}H\}$ NMR (126 MHz, $CDCl_3$)

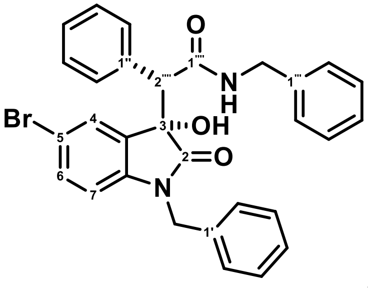

(2''''*S*,3*S*)-**17**

^{1}H,^{13}C-gs-HSQC w/ME

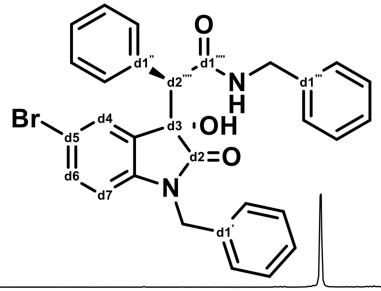

(2''''*R*,3*S*)-**17**

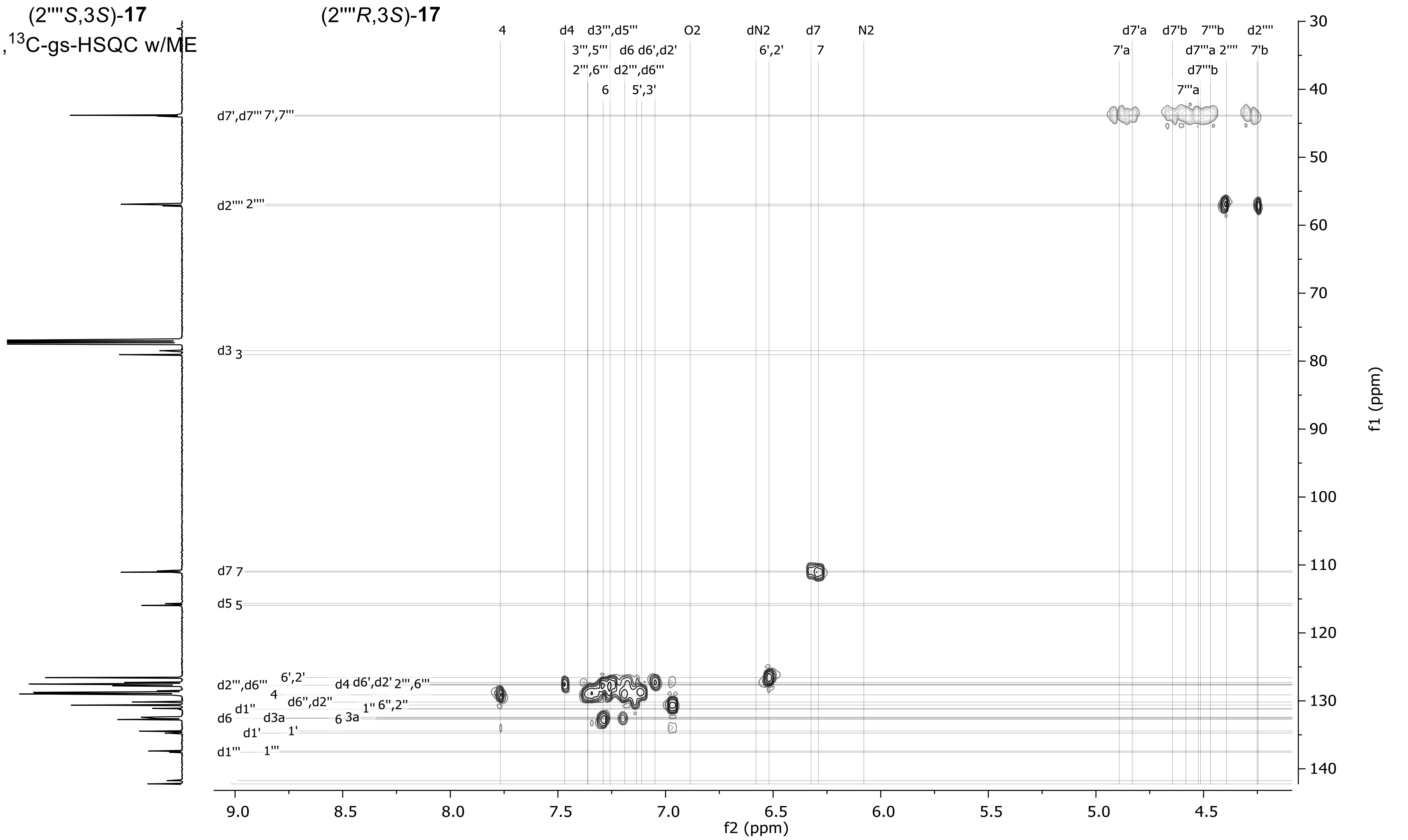

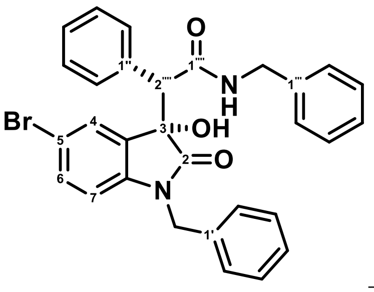

(2''''S,3S)-**17**

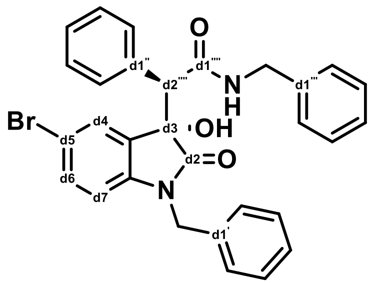

(2''''R,3S)-**17**

$^{1}H,^{13}C$-gs-HMBC

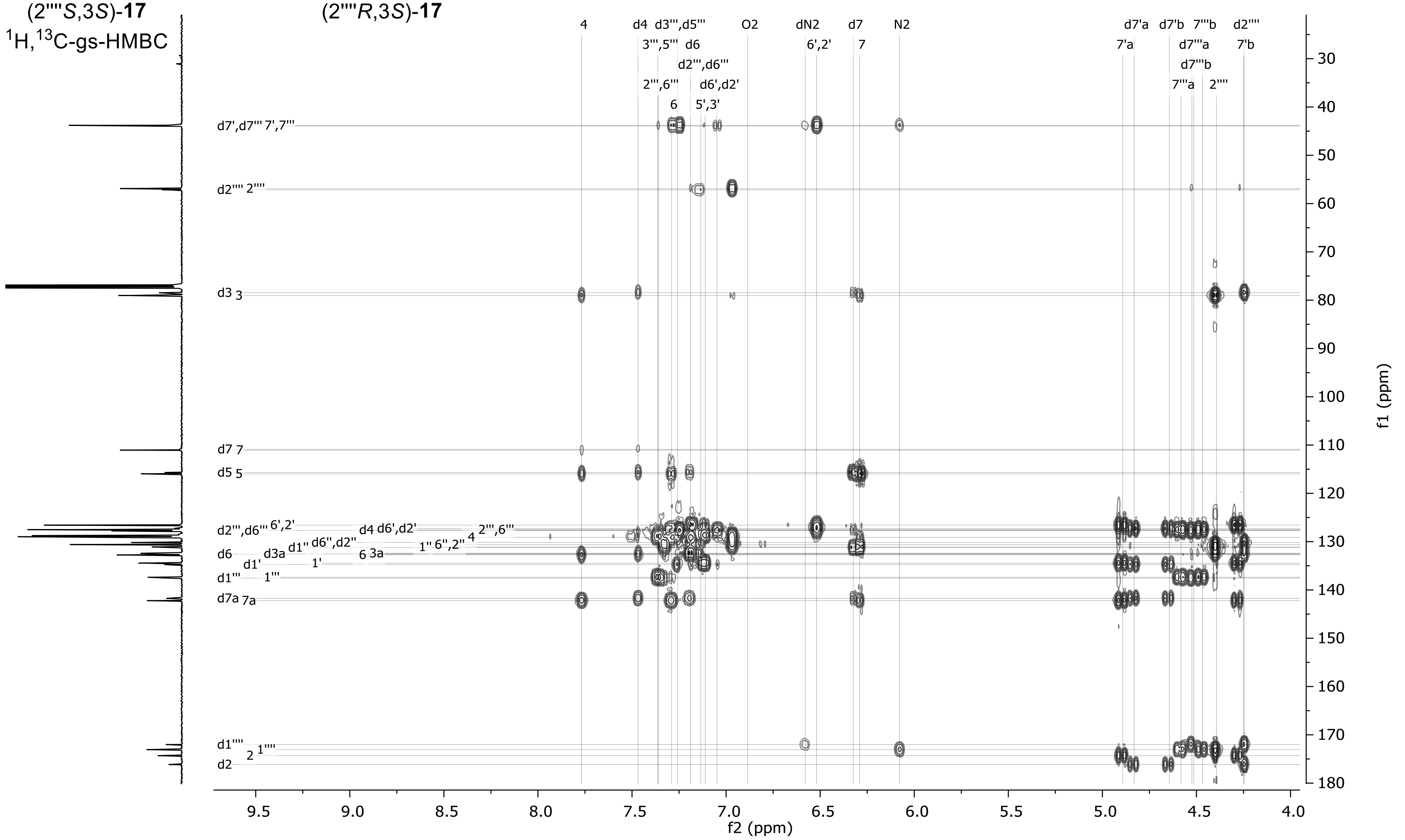

l) ***N,N'*-dibenzyl-2-(3-hydroxy-2-oxo-5-trifluoromethoxyindolin-3-yl)-2-phenylacetamide (18)**

To a 25 ml round bottomed flask was added phenylacetic anhydride (95.3 mg, 0.375 mmol), *N*-benzyl-5-trifluoromethoxyisatin (76.3 mg, 0.238 mmol), and (2*S*,3*R*)-HyperBTM (3.9 mg, 0.012 mmol). The mixture was cooled to 0 °C and CH_2Cl_2 (6.0 ml, 0.04 M) and Hünig's base (54 µl, 0.312 mmol) were added. The mixture was stirred at 0 °C for 3 h. Benzylamine (82 µl, 0.750 mmol) was added and the reaction was left to be stirred overnight at room temperature. 1,3,5-trimethoxybenzene (0.1 M soln in CH_2Cl_2 500 µl, 0.05 mmol) was added and the solvent was removed under reduced pressure. Purification by column chromatography (Hexane:Et_2O 3:2 → Hexane:Acetone 9:1 → 3:2) gave the title compound as white solid in two fractions (78:22 d.r., 122.3 mg, 0.224 mmol, 94%)

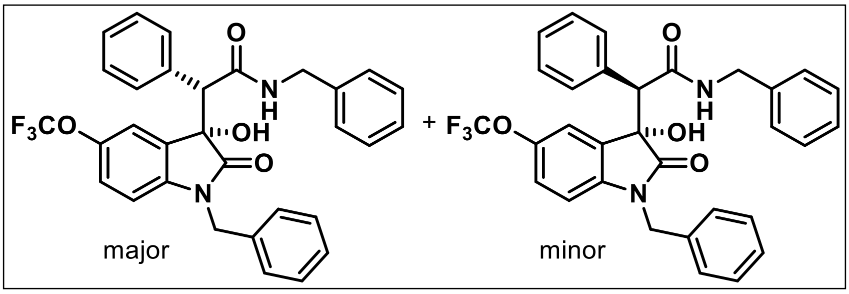

Major (2''''*S*,3*S*)-**18** and **minor** (2''''*R*,3*S*)-**18** analysed as a 79:21 mixture of diastereomers. **R*f*** 0.15 (Hexane:Acetone 7:3); α_D^{20} = 37.1 (*c* 0.39, $CHCl_3$); **Chiral HPLC analysis** (Chiralcel® IB 95:5 Hexane:IPA, flow rate 2.0 ml·min^{-1}, 211 nm, 40 °C) t_R (2''''*S*,3*R*)-**18**: 11.3 min, t_R (2''''*R*,3*S*)-**18**, <1:99 e.r.; t_R (2''''*R*,3*R*)-**18**: 13.8 min, t_R (2''''*S*,3*S*)-**18**: 18.1 min, 5:95 e.r.; ν_{max} (thin film) 3343 (m, broad, OH, NH), 3088 (w, CH), 3065 (w, CH), 3032 (w, CH), 1717 (s, C=O), 1645 (s, C=O), 1620 (m), 1526 (m), 1489 (s), 1454 (s), 1346 (m, OCF_3), 1250 (s, OCF_3), 1217 (s, OCF_3), 1175 (s, OCF_3), 1153 (s, OCF_3), 1126 (w), 1080 (w), 1030 (w), 962 (w), 895 (w), 854 (w), 822 (w); **^{1}H NMR** (400 MHz, CD_3CN) δ_H 7.42 – 7.12 (14.4H, m, PhC*H*), 7.36 (0.8H, s(br), ArC^4*H*), 7.09 (0.8H, dd, $^3J_{HH}$ = 8.5 Hz, $^4J_{HH}$ = 2.5 Hz, ArC^6*H*), 7.06 (0.2H, dd, $^3J_{HH}$ = 8.5 Hz, $^4J_{HH}$ = 2.4 Hz, ArC^6*H*), 6.87 – 6.84 (1.6H, m, PhC^{2,6}*H*), 6.84 (0.2H, s (br), ArC^4*H*), 6.63 (0.2H, d, $^3J_{HH}$ = 8.5 Hz, ArC^7*H*), 6.57 (0.8H, d, $^3J_{HH}$ = 8.5 Hz, ArC^7*H*), 5.96 (0.8H, d(br), $^4J_{HH}$ = 2.0 Hz, O*H*), 5.80 (0.2H, s(br), O*H*), 4.90 (0.8H, d, $^2J_{HH}$ = 16.1 Hz, NC*H*$_a$H$_b$-Ph), 4.88 (0.2H, d, $^2J_{HH}$ = 15.9 Hz, NHC*H*$_a$H$_b$-Ph), 4.70 (0.2H, d, $^2J_{HH}$ = 15.9 Hz, NHCH$_a$*H*$_b$-Ph), 4.51 (0.8H, d, 2J_{HH} = 16.1 Hz, NCH$_a$*H*$_b$-Ph), 4.42 (0.8H, dd, $^2J_{HH}$ = 15.3 Hz, $^3J_{HH}$ = 6.2 Hz, NHC*H*$_a$H$_b$-Ph), 4.37 (0.8H, dd, $^2J_{HH}$ = 15.3 Hz, $^3J_{HH}$ = 5.8 Hz, NHCH$_a$*H*$_b$-Ph), 4.37 – 4.28 (0.2H, m, NHC*H*$_2$-Ph), 4.33 (0.2H, s, C*H*-Ph), 4.11 (0.8H, d, $^4J_{HH}$ = 2.0 Hz, C*H*-Ph); **^{13}C {^{1}H} NMR** (101 MHz, CD_3CN) δ_C 177.6 (*C*(O)NBn, minor), 176.8 (*C*(O)NBn, major), 172.6 (*C*(O)NHBn, major), 171.9 (*C*(O)NHBn, minor), 145.1 (q, $^3J_{CF}$ = 2.5 Hz, Ar*C*^{5}OCF$_3$, major), 145.0 (q, $^3J_{CF}$ = 2.4 Hz, Ar*C*^{5}OCF$_3$, minor), 143.1 (Ar*C*^{7a}, minor), 143.0 (Ar*C*^{7a}, major), 139.9 (Ph*C*$^{1'''}$CH$_2$NH, minor), 139.7 (Ph*C*$^{1'''}$CH$_2$NH, major), 136.5 (Ph*C*$^{1'}$CH$_2$N, minor), 136.1 (Ph*C*$^{1'}$CH$_2$N, major), 134.6 (Ph*C*$^{1''}$CH, minor), 134.4 (Ph*C*$^{1''}$CH, major), 132.4 (Ar*C*^{4a}, minor), 131.3 (Ph*C*H), 131.1 (Ar*C*^{4a}, major), 129.7 (Ph*C*H), 129.6 (Ph*C*H), 129.4 (Ph*C*H), 129.2 (Ph*C*H), 129.1 (Ph*C*H), 129.0 (Ph*C*H), 128.5 (Ph*C*H), 128.4 (Ph*C*H), 128.3 (Ph*C*H), 128.2 (Ph*C*^{2,6}H, major), 128.0 (Ph*C*H), 123.7 (Ar*C*^{6}H, major), 123.4 (Ar*C*^{6}H, minor), 122.8 (Ph*C*^{2,6}H, major), 121.8 (Ar*C*^{4}H, major), 121.6 (q, $^1J_{CF}$ = 255.0 Hz, *C*F$_3$ major), 119.7 (Ar*C*^{4}H, minor), 110.7$_4$ (Ar*C*^{7}, major), 110.7$_0$ (Ar*C*^{7}, minor), 79.2 (*C*-OH, major), 78.1 (*C*-OH, minor), 58.5 (*C*H-Ph, minor), 57.1 (*C*H-Ph, major), 44.3 (N*C*H$_2$-Ph, minor), 44.1 (N*C*H$_2$-Ph, major), 43.7 (NH*C*H$_2$-Ph, major), 43.6 (NH*C*H$_2$-Ph, minor); ***m/z*** (ESI$^+$) 396 ([M-BnNHCOO]$^+$ 24%), 547 ([M+H]$^+$ 100%), 548 ([M(^{13}C)+H]$^+$ 34%), 549 ([M+$^{13}C_2$]$^+$ 6%), 569 ([M+Na]$^+$ 14%), 585 ([M+K]$^+$ 8%), 848 (8%); **HRMS** (ESI$^+$) *m/z* calcd for [M+H]$^+$ $C_{31}H_{26}O_4N_2F_3$ 547.1839, found 547.1823 (−3.0 ppm).

(±)-anti-**18** + (±)-syn-**18**

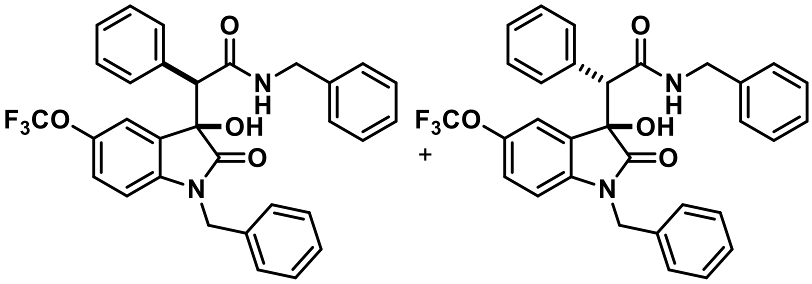

PDA Ch1 211nm

Peak#	Ret. Time	Area%
1	11.276	9.893
2	13.819	40.967
3	18.083	38.060
4	19.941	11.080
Total		100.000

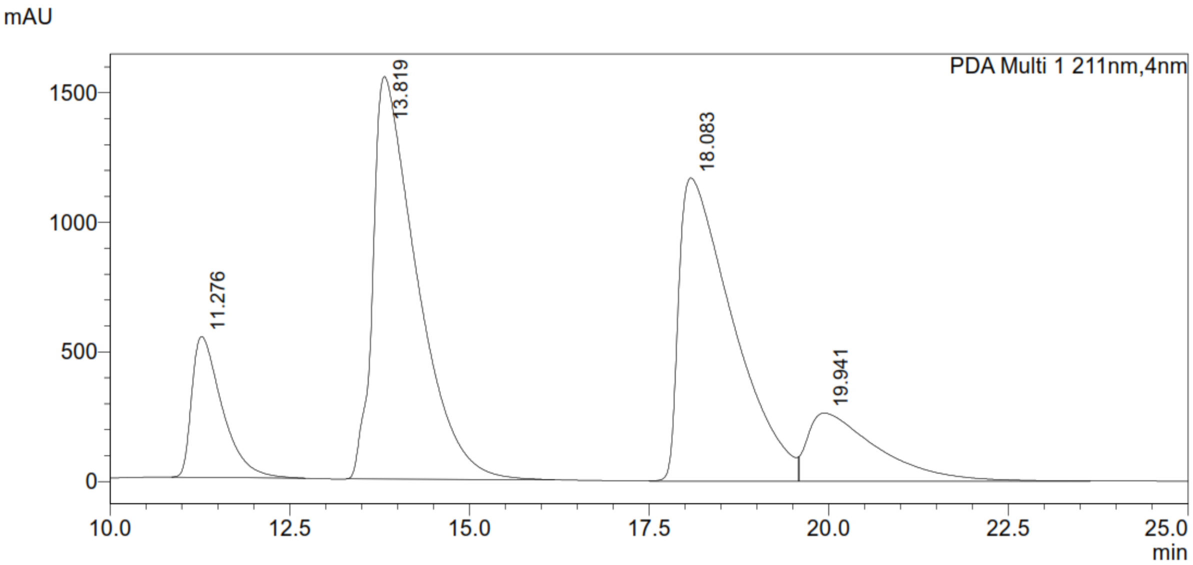

(2''''*S*,3*S*)-**18** + (2''''*R*,3*S*)-**18**

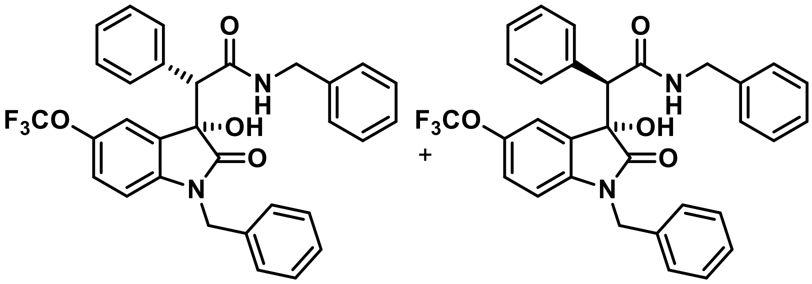

PDA Ch1 211nm

Peak#	Ret. Time	Area%
1	11.595	0.073
2	13.800	3.926
3	18.529	74.221
4	20.427	21.780
Total		100.000

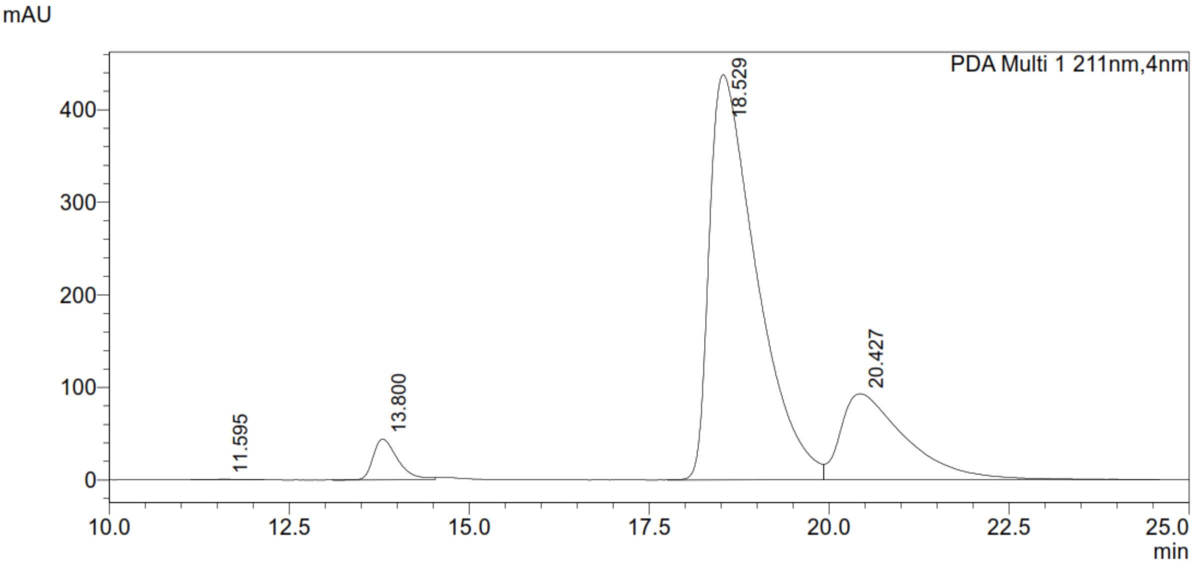

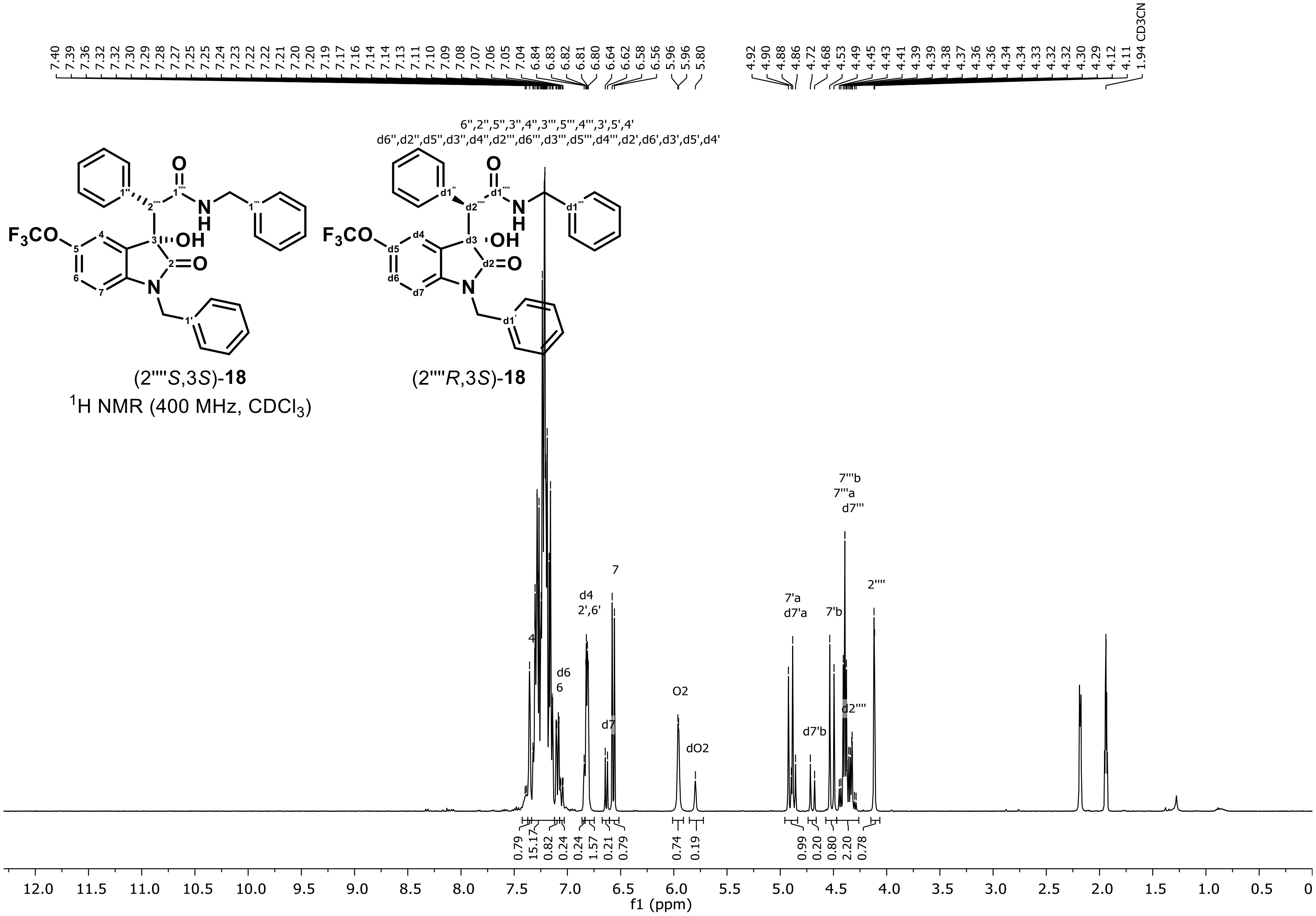

^{1}H NMR (400 MHz, $CDCl_3$)

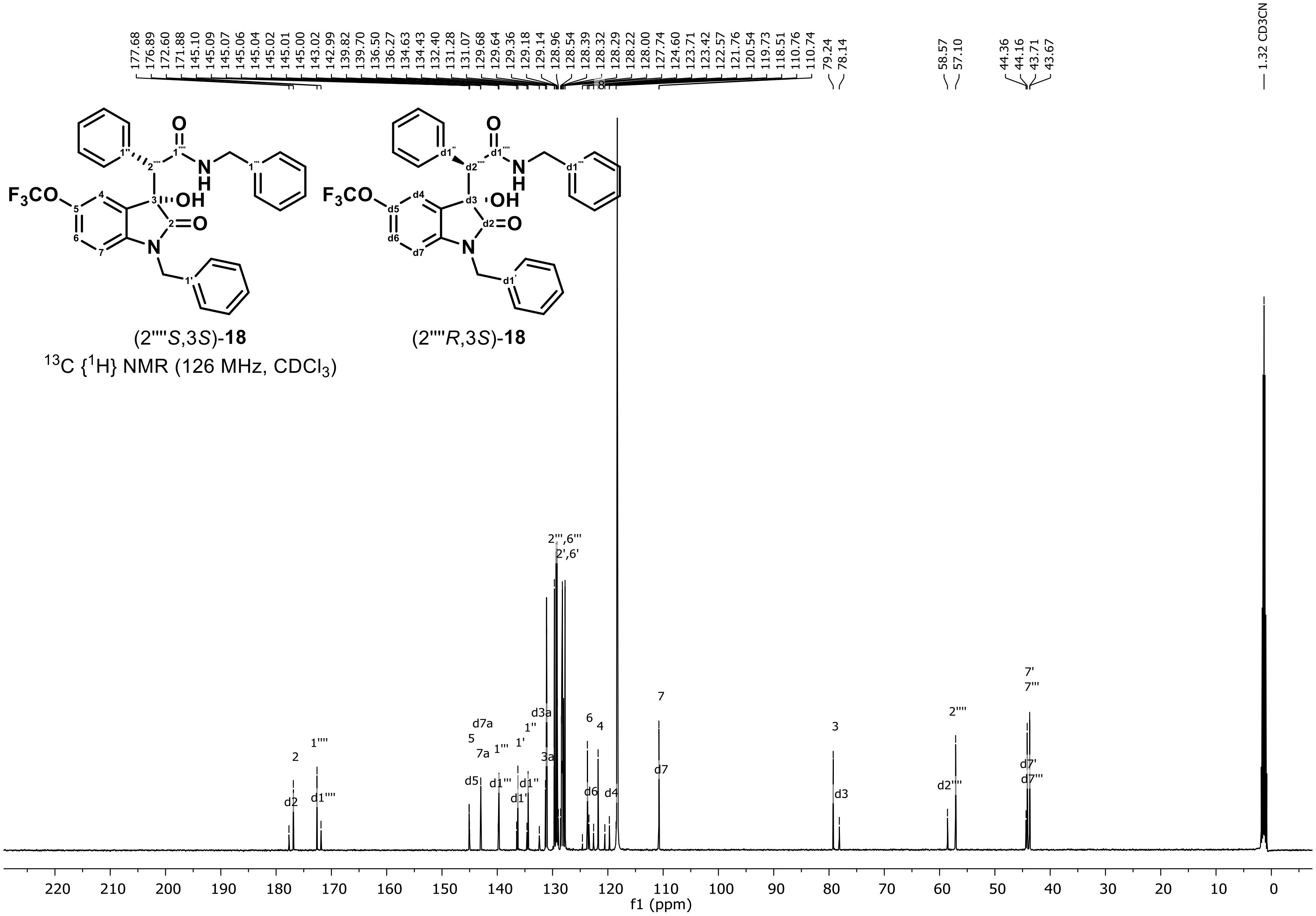
177.68
176.89
172.60
171.88
145.10
145.09
145.07
145.06
145.04
145.02
145.01
145.00
143.02
142.99
139.82
139.70
136.50
136.27
134.63
134.43
132.40
131.28
131.07
129.68
129.64
129.36
129.18
129.14
128.96
128.54
128.50
128.39
128.32
128.29
128.22
128.00
127.74
124.60
123.71
123.42
122.57
121.76
120.54
119.73
118.51
110.76
110.74
79.24
78.14
58.57
57.10
44.36
44.16
43.71
43.67
1.32 CD3CN
F3CO
OH
O
N
H
(2''''S,3S)-18
(2''''R,3S)-18
13C {1H} NMR (126 MHz, CDCl3)
2
d2
1''''
d1''''
5
d5
d7a
7a
1'''
d1'''
1''
1'
d1''
d1'
d3a
3a
2''',6'''
2',6'
6
4
d6
d4
7
d7
3
d3
2''''
d2''''
7'
7'''
d7'
d7'''
f1 (ppm)
220
210
200
190
180
170
160
150
140
130
120
110
100
90
80
70
60
50
40
30
20
10
0

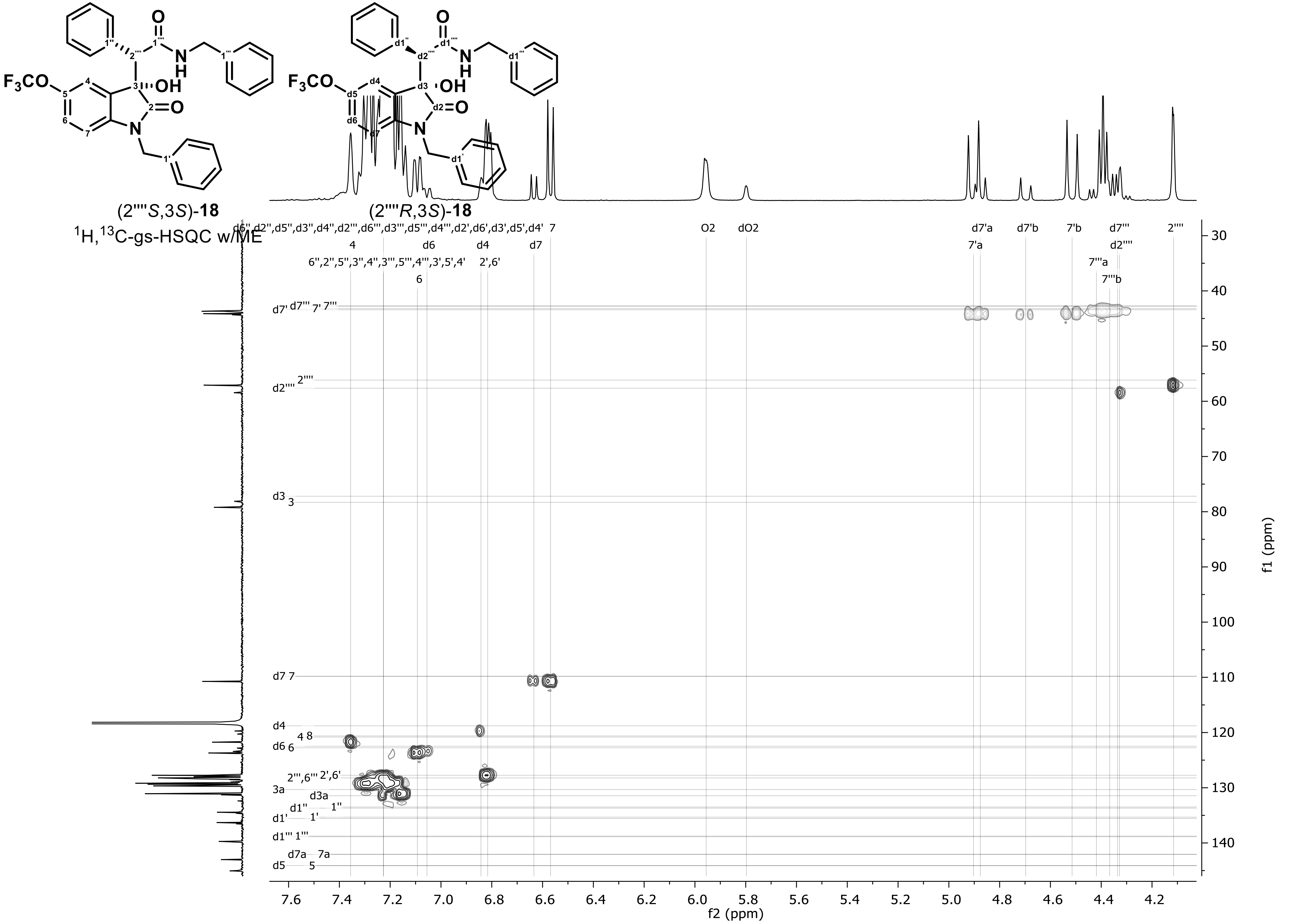

(2''''S,3S)-18 (2''''R,3S)-18

$^{1}H,^{13}C$-gs-HSQC w/ME

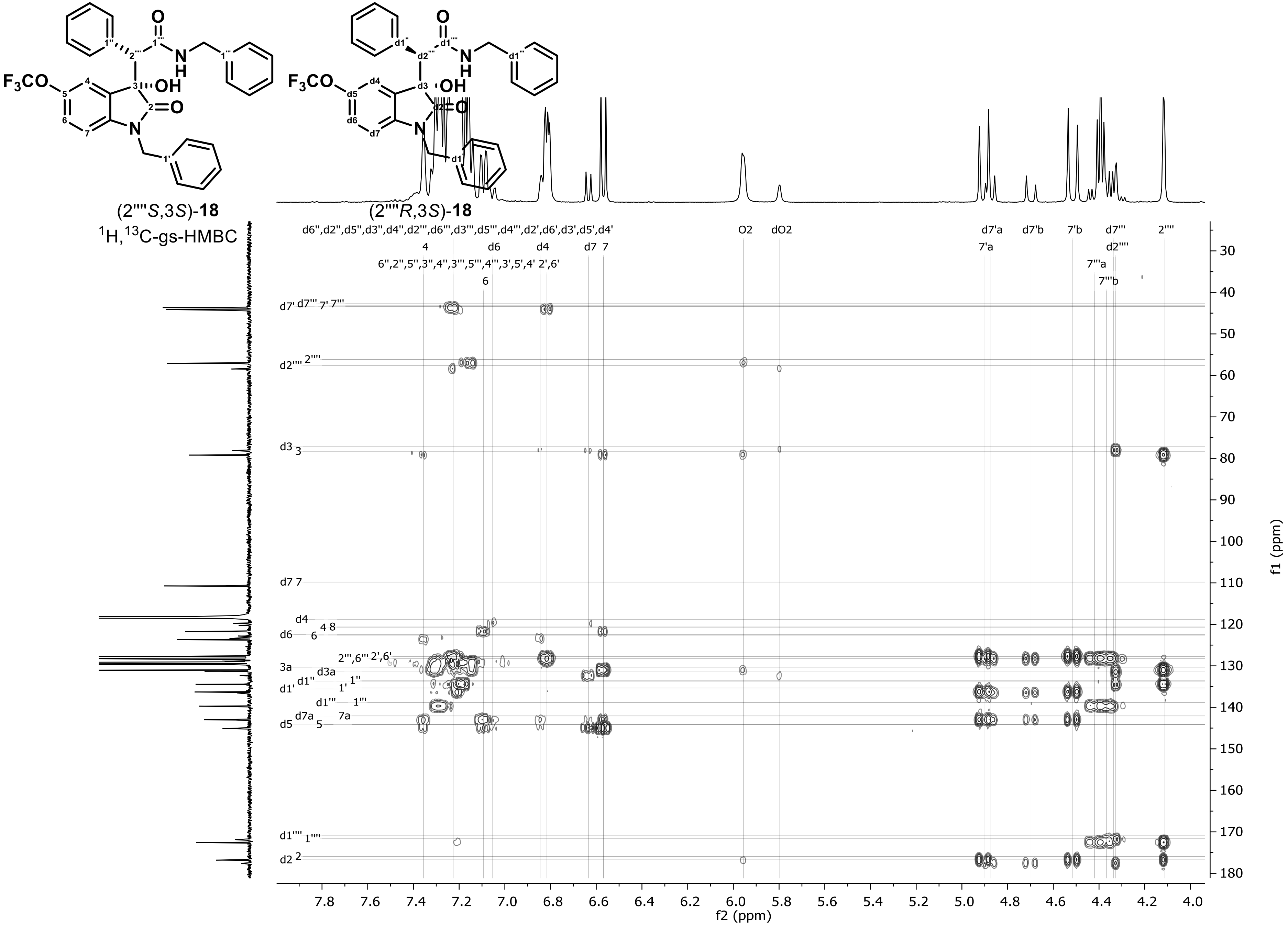

(2''''*S*,3*S*)-**18**

(2''''*R*,3*S*)-**18**

$^{1}H,^{13}C$-gs-HMBC

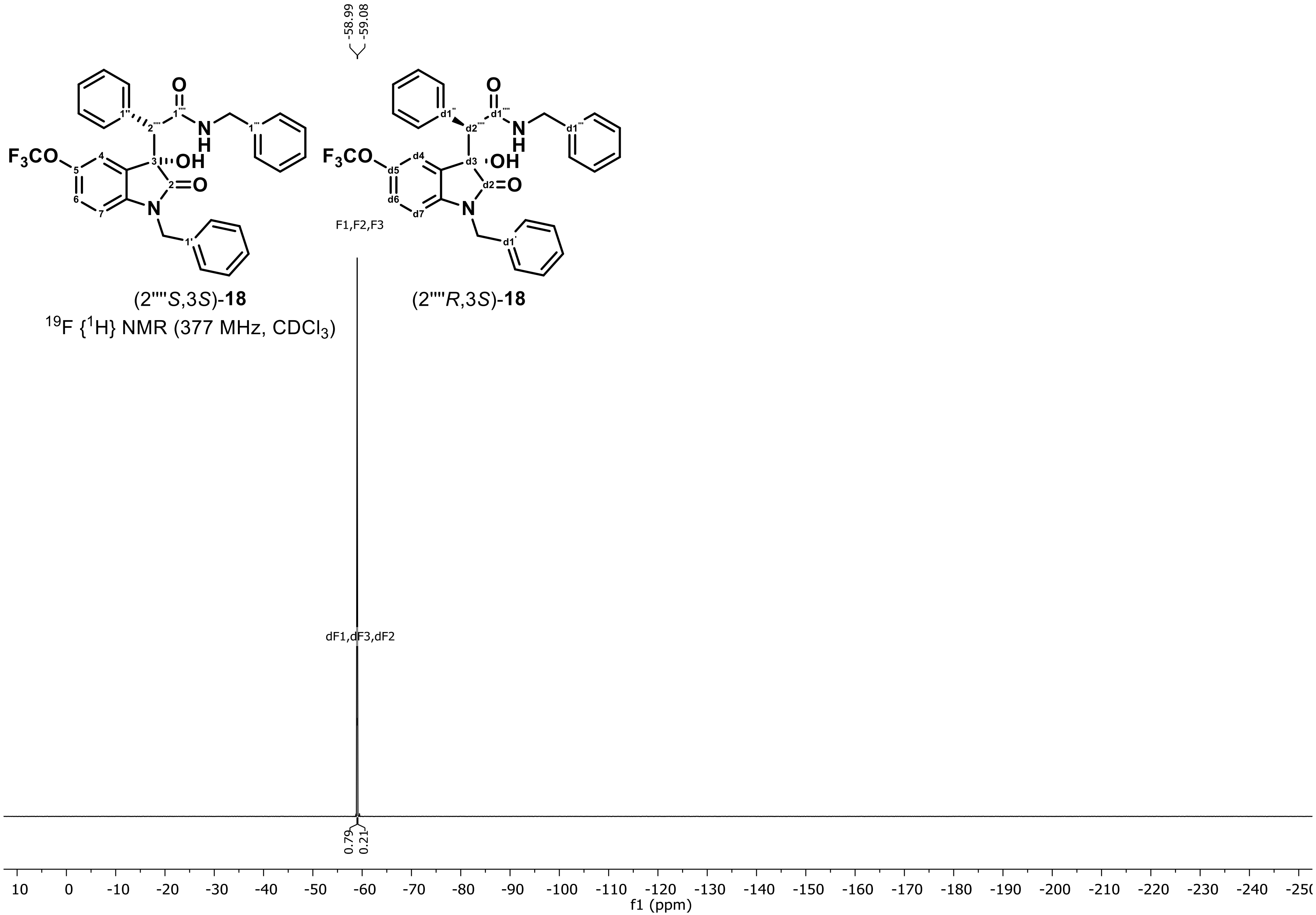

(2''''*S*,3*S*)-**18**

(2''''*R*,3*S*)-**18**

$^{19}F\{^{1}H\}$ NMR (377 MHz, $CDCl_3$)

m) N,N'-Dibenzyl-2-(3-hydroxy-5-methoxy-2-oxoindolin-3-yl)-2-phenylacetamide (19)

To a 25 ml round bottomed flask was added phenylacetic anhydride (95.3 mg, 0.375 mmol), *N*-benzyl-5-methoxyisatin (66.9 mg, 0.250 mmol), and 2*S*,3*R*-HyperBTM (3.9 mg, 0.012 mmol). The mixture was cooled to 0 °C and CH_2Cl_2 (6.0 ml, 0.04 M) and Hünig's base (54 µl, 0.312 mmol) were added. The mixture was stirred at 0 °C for 3 h. Benzylamine (82 µl, 0.750 mmol) was added and them reaction was left to be stirred overnight at room temperature. 1,3,5-Trimethoxybenzene (0.1 M soln in CH_2Cl_2 500 µl, 0.05 mmol) and the solvent was removed. Purification via column chromatography (Petrol:EtOAc 4:1 → 2:3) gave three fractions: The major diastereoisomer (90.1 mg, 0.176 mmol, 71%) as light brown semi-solid, a mixture of diastereomers (7.6 mg, 0.015 mmol, 6%) as brown solid and the minor diastereomer (21.1 mg, 0.041 mmol, 17%) as brown solid; combined yield of 93% (118.8 mg, 0.233 mmol).

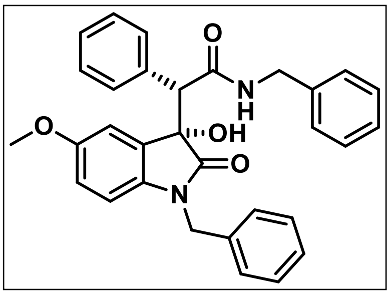

Major (2''''*S*,3*S*)-**19**: **m.p.** 168 °C; **R*f*** 0.51 (4:6 Hexane:EtOAc); α_D^{20} = +84.9 (*c* 1.62 in $CHCl_3$); **Chiral HPLC** analysis, Chiralpak IB (95:5 hexane:IPA, flow rate 2 ml·min^{-1}, 211 nm, 40 °C) t_R (2''''*R*,3*S*)-**19**: 27.0 min, t_R (2''''*S*,3*S*)-**19**: 33.3 min, <1:99 e.r.; ***ν*max** (thin film) 3339 (m (br), O-H), 3086 (w, C-H), 3061 (w, C-H), 3032 (w, C-H), 3005 (w, C-H), 2928 (w, C-H), 2033 (w, C-H), 1717 (s, C=O amide), 1670 (m, C=O isatin), 1651 (m, C=O isatin), 1603 (w), 1539 (w), 1526 (w), 1493 (s, C=C), 1472 (m), 1454 (w), 1437 (m), 1348 (w), 1296 (w), 1277 (w), 1246 (w), 1227 (w), 1198 (w), 1182 (w), 1157 (w), 1078 (w), 1030 (w), 1018 (w), 910 (w), 814 (w); **^{1}H NMR** (400 MHz, $CDCl_3$) δ_H 7.34 – 7.23 (4H, m, PhC$^{4''}$*H*, PhC$^{3''',4''',5'''}$*H*), 7.23 – 7.19 (2H, m, PhC$^{2''',6'''}$*H*), 7.19 – 7.12 (4H, m, ArC^4*H*, PhC$^{4'}$*H*, PhC$^{3'',5''}$*H*), 7.12 – 7.06 (2H, m, PhC$^{3',5'}$*H*), 6.96 (2H, app d, $^3J_{HH}$ = 7.3 Hz, PhC$^{2'',6''}$*H*), 6.86 (1H, s(br), O*H*), 6.67 (1H, dd, $^3J_{HH}$ = 8.5 Hz, $^4J_{HH}$ = 2.6 Hz, ArC^6*H*), 6.55 (2H, app d, $^3J_{HH}$ = 7.0 Hz, PhC$^{2',6'}$*H*), 6.31 (1H, d, $^3J_{HH}$ = 8.5 Hz, ArC^7*H*), 5.93 (1H, t(br), $^3J_{HH}$ = 5.9 Hz, N*H*), 4.89 (1H, d, $^2J_{HH}$ = 15.9 Hz, NC*H*$_a$H$_b$-Ph), 4.53 (1H, dd, $^2J_{HH}$ = 14.9 Hz, $^3J_{HH}$ = 5.9 Hz, NHC*H*$_a$H$_b$-Ph), 4.48 (1H, dd, $^2J_{HH}$ = 14.9 Hz, $^3J_{HH}$ = 5.9 Hz, NHCH$_a$*H*$_b$-Ph), 4.33 (1H, s, C*H*-Ph), 4.30 (1H, d, $^2J_{HH}$ = 15.9 Hz, NCH$_a$*H*$_b$-Ph), 3.68 (3H, s, OC*H*$_3$); **^{13}C {^{1}H} NMR** (101 MHz, $CDCl_3$) δ_C 174.6 (*C*(O)NBn), 173.4 (*C*(O)NHBn), 156.3 (Ar*C*^{5}OCH$_3$), 137.5 (Ph*C*$^{1'''}$), 136.6 (Ar*C*^{7a}), 135.2 (Ph*C*$^{1'}$), 132.9 (Ph*C*$^{1''}$), 130.8 (Ph*C*$^{2'',6''}$H), 130.1 (Ar*C*^{3a}), 129.0 (Ph*C*$^{3'',5''}$H), 128.9 (Ph*C*$^{3''',5'''}$H), 128.7$_2$ (Ph*C*$^{4''}$H), 128.6$_7$ (Ph*C*$^{3',5'}$H), 127.8 (Ph*C*$^{4'''}$H), 127.6 (Ph*C*$^{2''',6'''}$H), 127.3 (Ph*C*$^{4'}$H), 126.7 (Ph*C*$^{2',6'}$H), 115.0 (Ar*C*^{6}H), 112.5 (Ar*C*^{4}H), 110.1 (Ar*C*^{7}H), 57.0 (PhC$^{1''}$*C*H), 55.9 (O*C*H$_3$), 43.9 (PhC$^{1'}$*C*H$_2$N), 43.8 (PhC$^{1'''}$*C*H$_2$NH); ***m/z*** (ESI$^+$) 309 ([M-BnNHCOOH-OMe-O+NH]$^+$ 37%), 342 ([M-BnNHCOO]$^+$ 30%), 493 ([M+H]$^+$ 100%), 494 ([M(^{13}C)+H]$^+$ 34%), 495 ([M(^{13}C$_2$)+H]$^+$ 6%), 515 ([M+Na]$^+$ 8%), 801 ([2M-BnNHCOOH-OMe-O+NH]$^+$ 3%), 985 ([2M+H]$^+$ 3%); **HRMS** (ESI$^+$) *m/z* calcd for [M+H]$^+$ $C_{31}H_{29}O_4N_2$ 493.2122, found 493.2104 (−3.7 ppm).

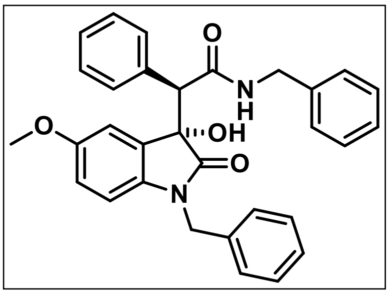

Minor (2''''*R*,3*S*)-**19**: **m.p.** 199 – 200 °C; **R*f*** 0.39 (4:6 Hexane:EtOAc); **Chiral HPLC** analysis, Chiralpak IB (92:8 hexane:IPA, flow rate 2 ml·min^{-1}, 211 nm, 40 °C) t_R (2''''*S*,3*R*)-**19**: 14.6 min, t_R (2''''*R*,3*S*)-**19**: 31.1 min, <1:99 e.r.; α_D^{20} = +93.9 (*c* 0.75 in $CHCl_3$); ***ν*max** (thin film): 3279 (m (br), O-H), 3088 (w, C-H), 3063 (w, C-H), 3030 (w, C-H), 2926 (w, C-H), 1695 (s, C=O amide), 1638 (s, C=O isatin), 1605 (m), 1578 (m), 1495 (s, C=C), 1468 (w), 1454 (m), 1437 (m), 1373 (w), 1350 (m), 1296 (w), 1277 (m), 1254 (w), 1200 (m), 1180 (m), 1163 (m), 1080 (m), 1047 (m), 1028 (w), 1018 (m), 910 (m), 814 (m), 760 (m); **^{1}H NMR** (400 MHz, $CDCl_3$) δ_H 7.35 – 7.24 (5H, m, PhC$^{2''',3''',5''',6'''}$*H* and PhC$^{4'}$*H* or PhC$^{4'''}$*H*), 7.24 – 7.20 (3H, m, PhC$^{3,'5'}$*H* and PhC$^{4'}$*H* or PhC$^{4'''}$*H*), 7.20 – 7.14 (1H, m, PhC$^{4''}$*H*), 7.14 – 7.07 (4H, m, PhC$^{2'',6''}$*H*, PhC$^{3'',5''}$*H*), 7.03 – 6.96 (2H, m, PhC$^{2',6'}$*H*), 6.98 (1H, d, $^4J_{HH}$ = 2.6 Hz, ArC^4*H*), 6.88 (1H, t(br), N*H*), 6.77 (1H, s(br), O*H*), 6.58 (1H, dd, $^3J_{HH}$ = 8.5 Hz, $^4J_{HH}$ = 2.6 Hz, AcC^6*H*), 6.31 (1H, d, $^3J_{HH}$ = 8.5 Hz, ArC^7*H*), 4.82 (1H, d, $^2J_{HH}$ = 15.8 Hz, NC*H*$_a$H$_b$-Ph), 4.60 (1H, d, $^2J_{HH}$ = 15.8 Hz, NCH$_a$*H*$_b$-Ph), 4.56 (1H, dd, $^{2J}{}_{HH}$ = 15.0 Hz, $^3J_{HH}$ = 5.7 Hz, NHC*H*$_a$H$_b$-Ph), 4.51 (1H, dd, $^2J_{HH}$ =15.0 Hz, $^3J_{HH}$ = 5.7 Hz, NHCH$_a$*H*$_b$-Ph), 4.27 (1H, s, C*H*-Ph), 3.70 (3H, s, OC*H*$_3$); **^{13}C {^{1}H} NMR** (101 MHz,

CDCl$_3$) δ_C 176.6 (*C*(O)NBn), 172.1 (*C*(O)NHBn), 156.3 (ArC^5OCH$_3$), 137.8 (Ph$C^{1'''}$CH$_2$NH), 136.0 (ArC^{7a}), 135.2 (Ph$C^{1'}$CH$_2$N), 132.9 (Ph$C^{1''}$CH), 130.4 (ArC^{3a}), 130.1 (Ph$C^{2'',6''}$H), 128.8$_6$, 128.8$_1$ and 128.7$_4$ (Ph$C^{3',5'}$H, Ph$C^{3'',5''}$H and Ph$C^{3''',5'''}$H), 128.3 (Ph$C^{4''}$H), 127.8 (Ph$C^{2''',6'''}$H), 127.6 (Ph$C^{4'}$H and Ph$C^{4'''}$H, not resolved), 127.3 (Ph$C^{2',6'}$H), 114.6 (ArC^6H), 111.3 (ArC^4H), 110.0 (ArC^7H), 78.9 (*C*-OH), 58.0 (*C*H-Ph), 55.9 (O*C*H$_3$), 44.0 (N*C*H$_2$-Ph), 43.9 (NH*C*H$_2$-Ph); ***m/z*** (ESI$^+$) 309 ([M-BnNHCOOH-OMe-O+NH]$^+$ 65%), 342 ([M-BnNHCOO]$^+$ 10%), 493 ([M+H]$^+$ 100%), 494 ([M(^{13}C)+H]$^+$ 34%), 495 ([M(^{13}C$_2$)+H]$^+$ 6%), 801 ([2M-BnNHCOOH-OMe-O+NH]$^+$ 11%), 985 ([2M+H]$^+$ 38%); **HRMS** (ESI$^+$) *m/z* calcd for [M+H]$^+$ $C_{31}H_{29}O_4N_2$ 493.2122, found 493.2103 (−3.9 ppm).

(±)-anti-**19**

PDA Ch1 211nm

Peak#	Ret. Time	Area%
1	26.422	50.005
2	34.968	49.995
Total		100.000

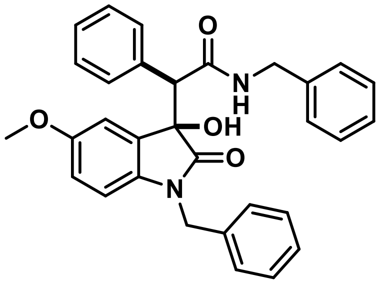

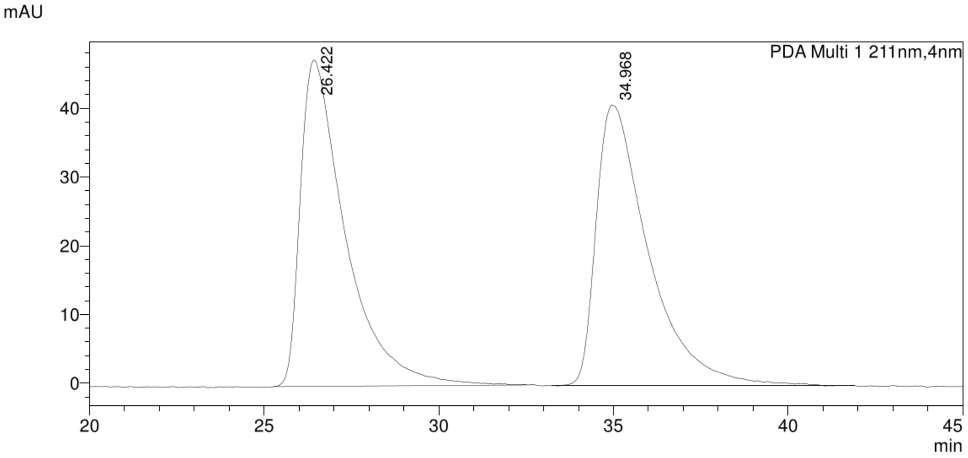

(+)-(2''''*S*,3*S*)-**19**

PDA Ch1 211nm

Peak#	Ret. Time	Area%
1	26.989	0.242
2	33.284	99.758
Total		100.000

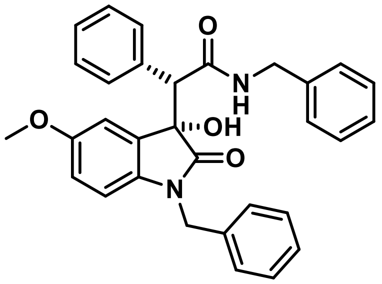

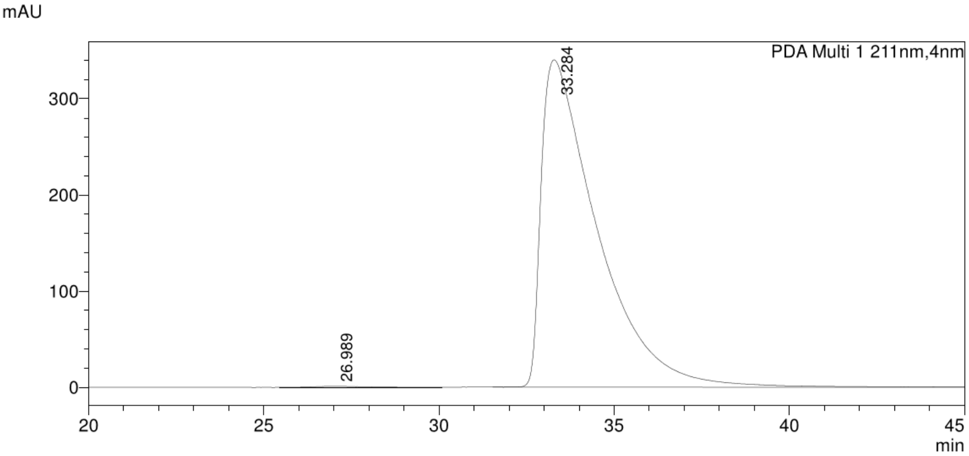

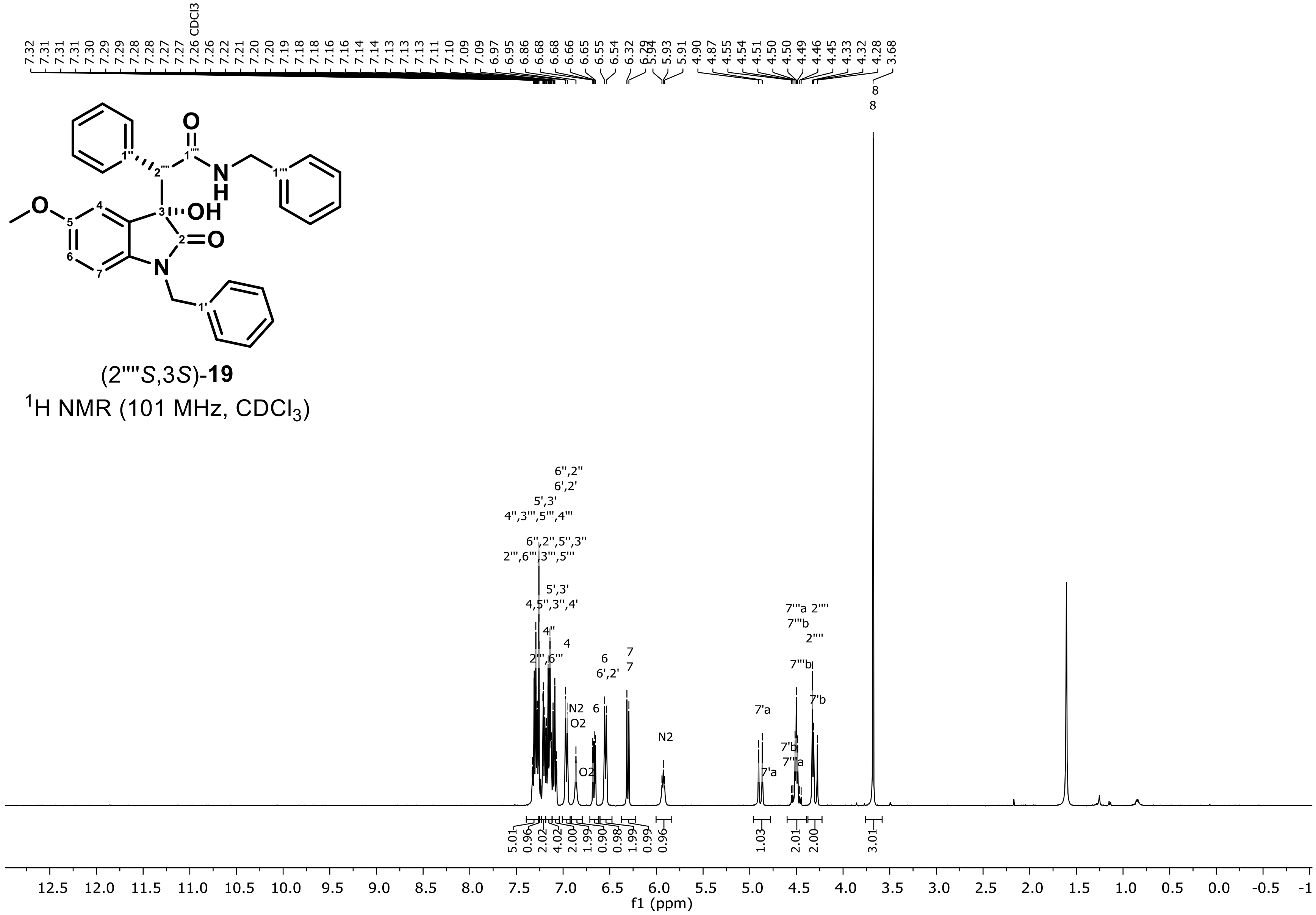

(2''''*S*,3*S*)-**19**

^{1}H NMR (101 MHz, $CDCl_3$)

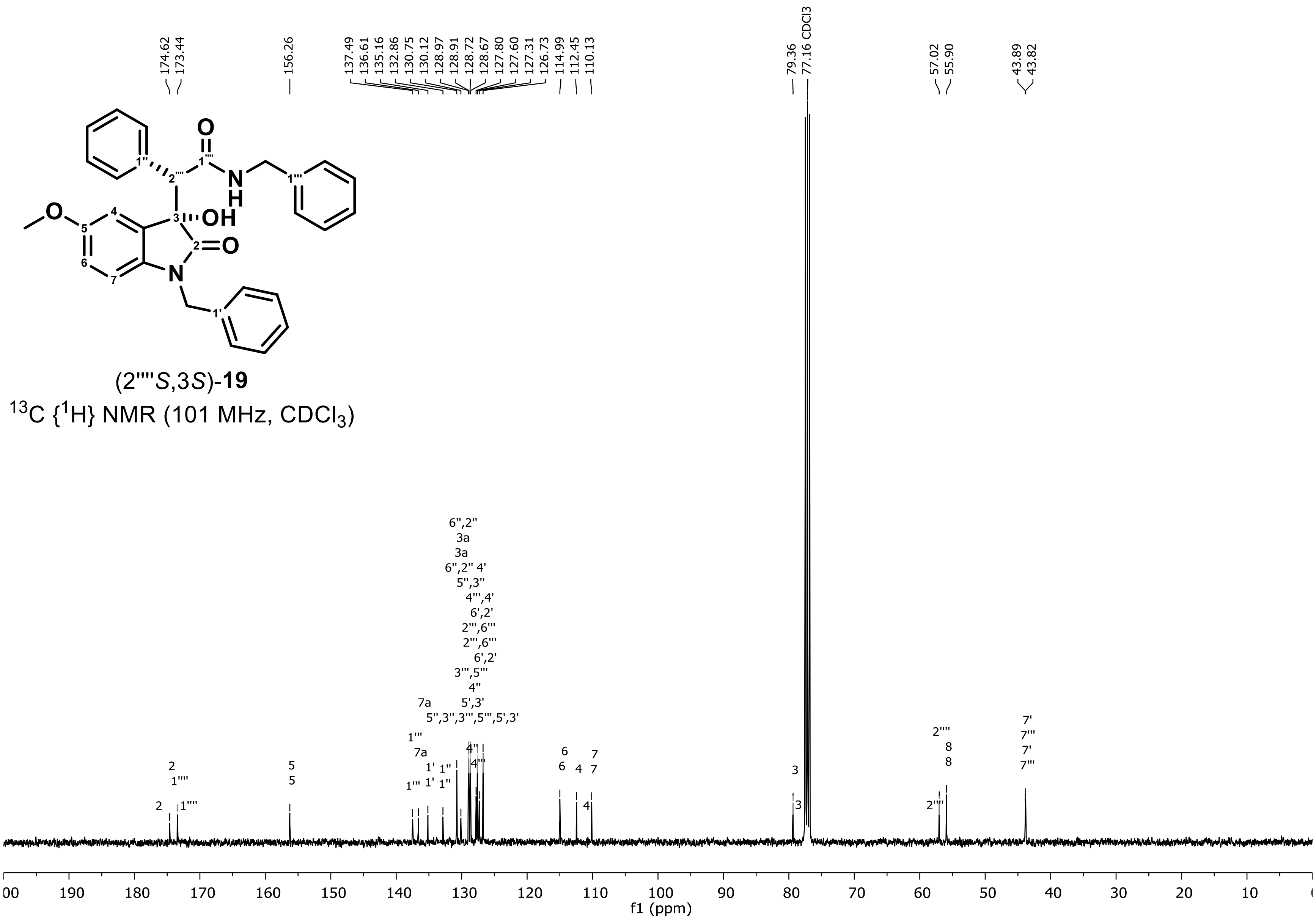

(2''''*S*,3*S*)-**19**

$^{13}C\ \{^{1}H\}$ NMR (101 MHz, $CDCl_3$)

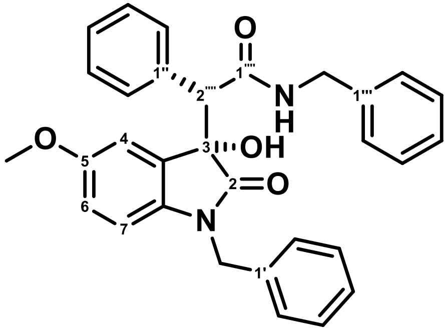

(2''''*S*,3*S*)-**19**

^{1}H,^{13}C-gs-HSQC w/ME

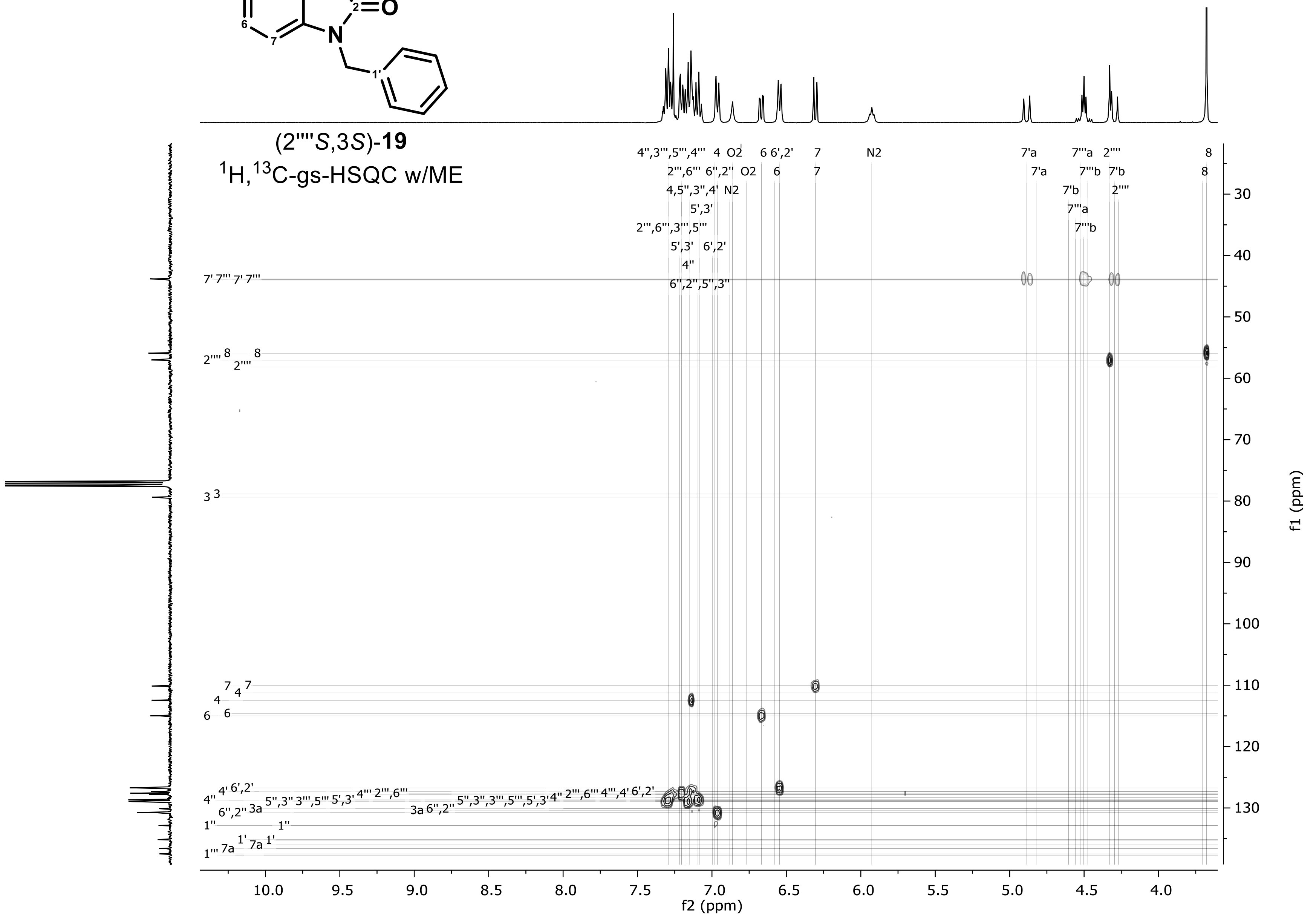

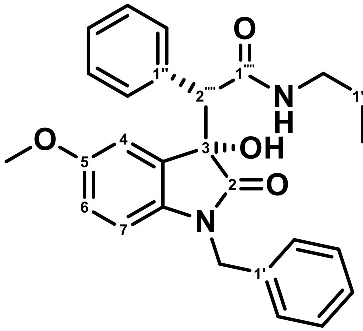

(2''''*S*,3*S*)-**19**

^{1}H,^{13}C-gs-HMBC

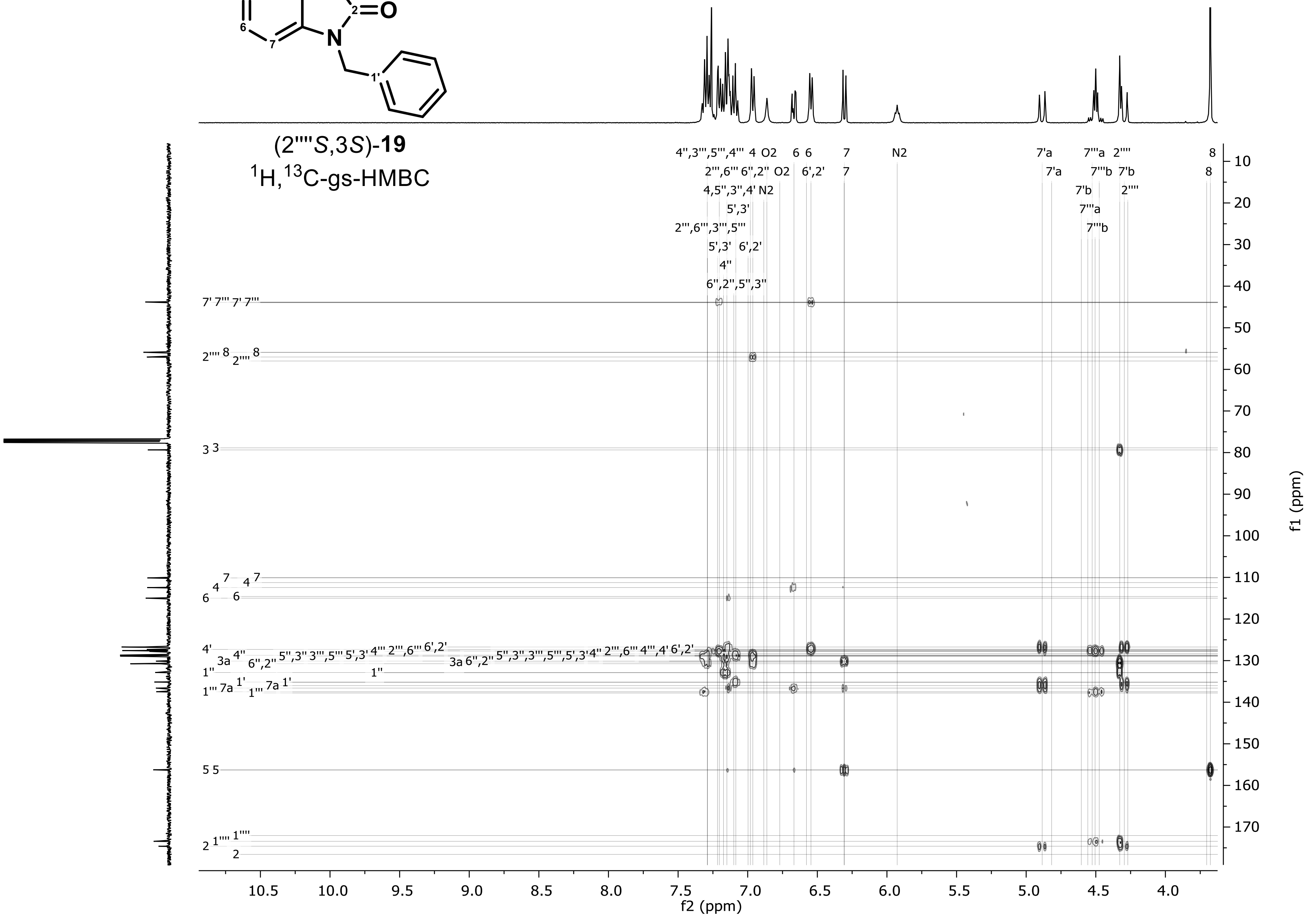

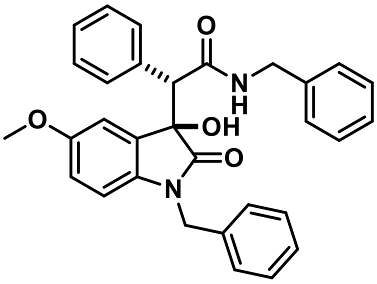

(±)-syn-**19**

PDA Ch1 211nm

Peak#	Ret. Time	Area%
1	14.503	50.072
2	30.647	49.928
Total		100.000

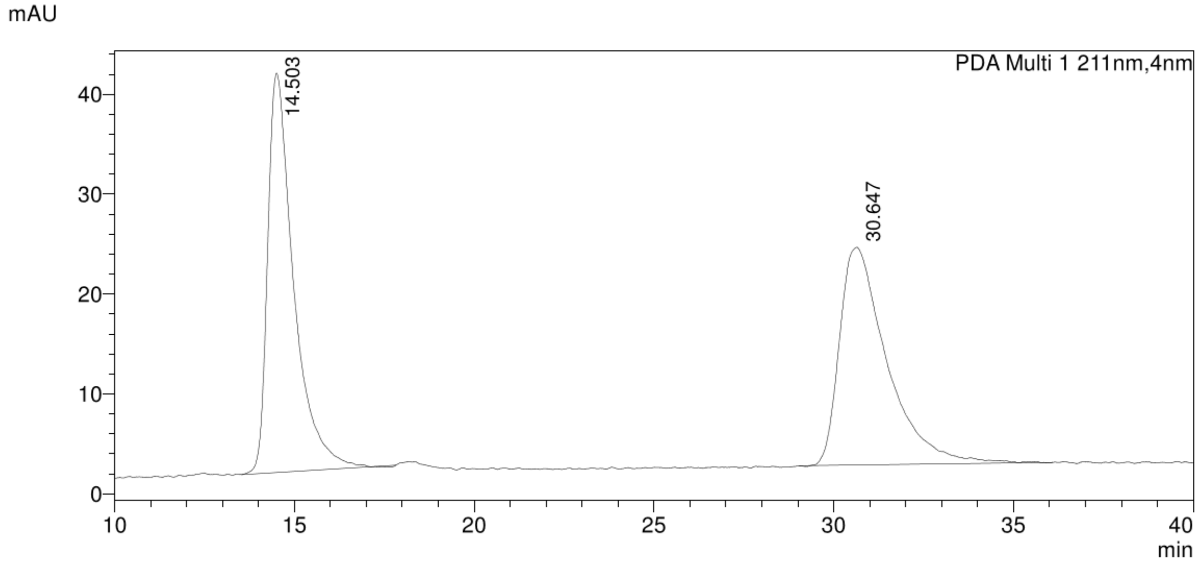

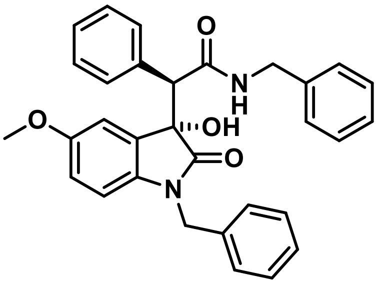

(+)-(2''''*R*,3*S*)-**19**

PDA Ch1 211nm

Peak#	Ret. Time	Area%
1	14.616	0.595
2	31.149	99.405
Total		100.000

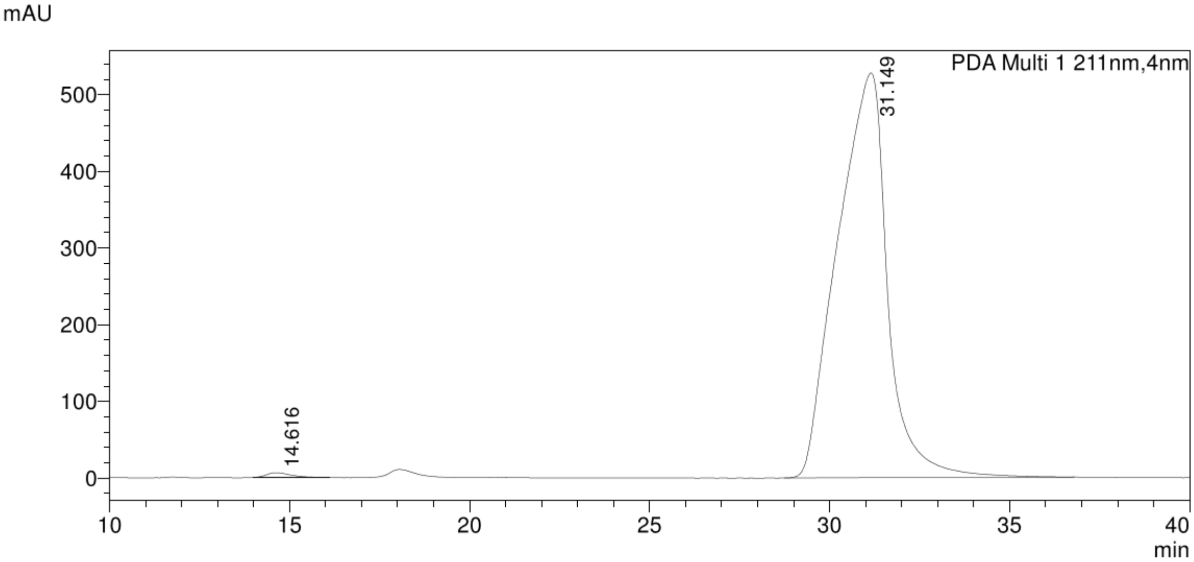

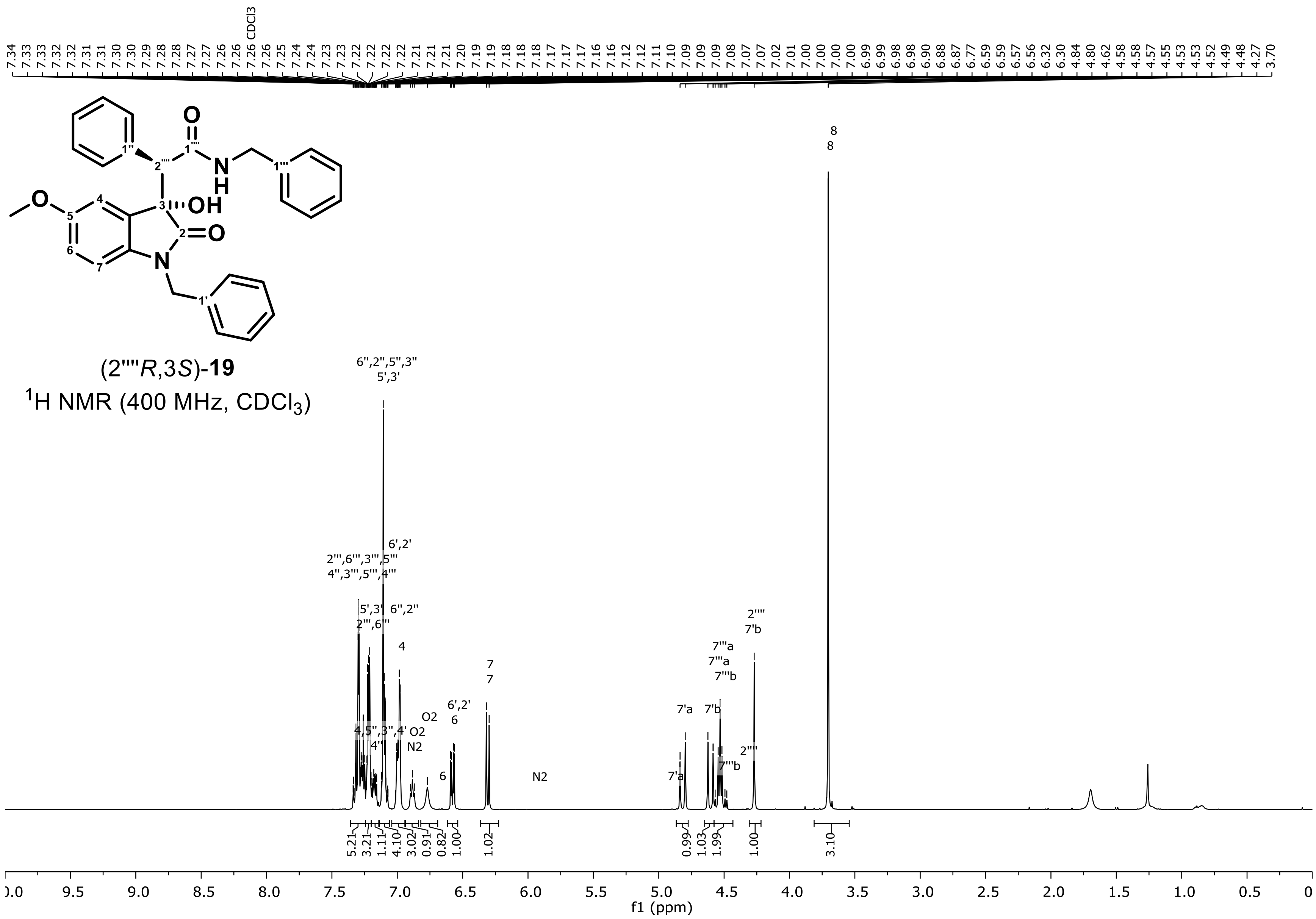

(2''''R,3S)-**19**

^{1}H NMR (400 MHz, $CDCl_3$)

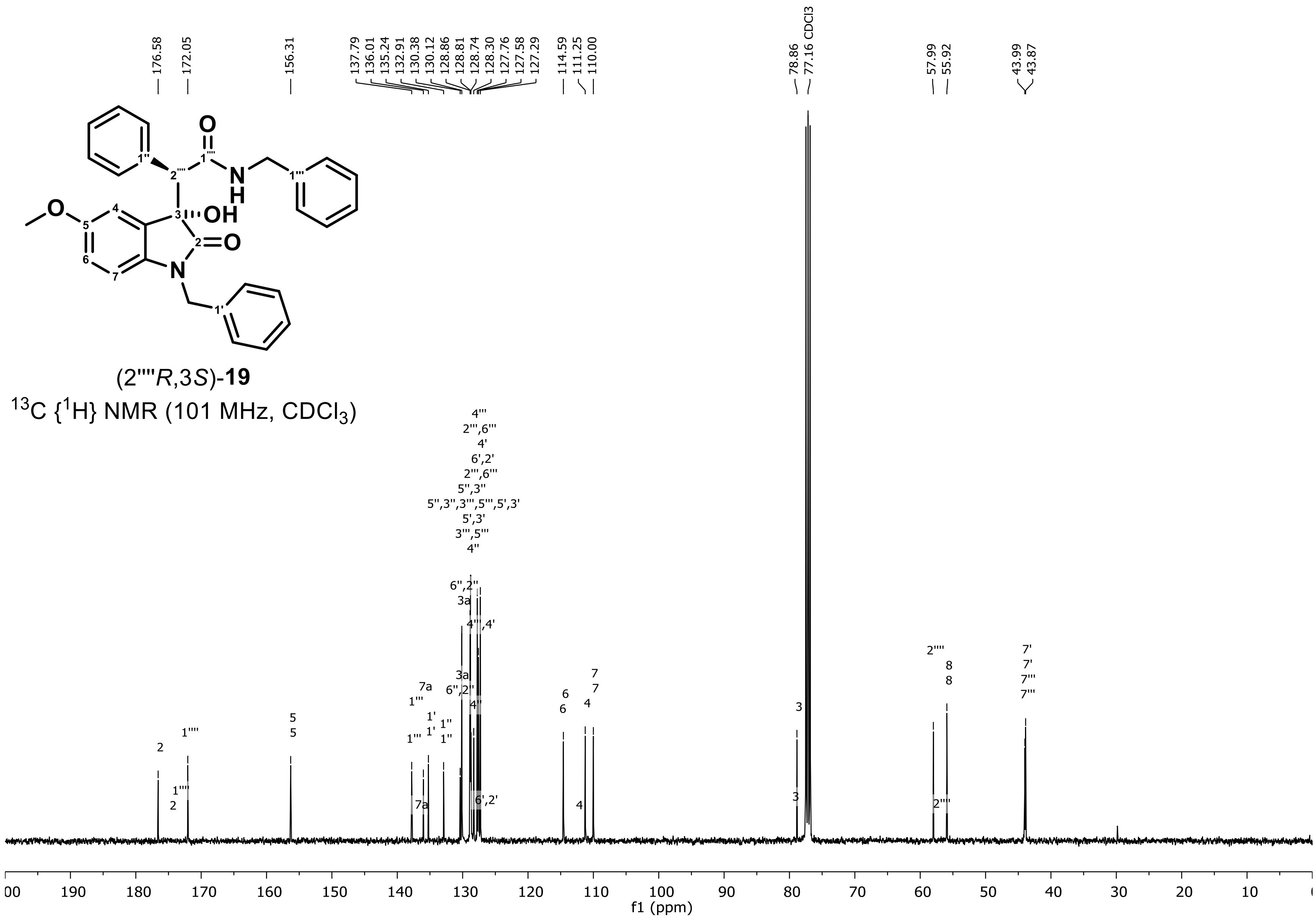

(2''''*R*,3*S*)-**19**

$^{13}C\ \{^1H\}$ NMR (101 MHz, $CDCl_3$)

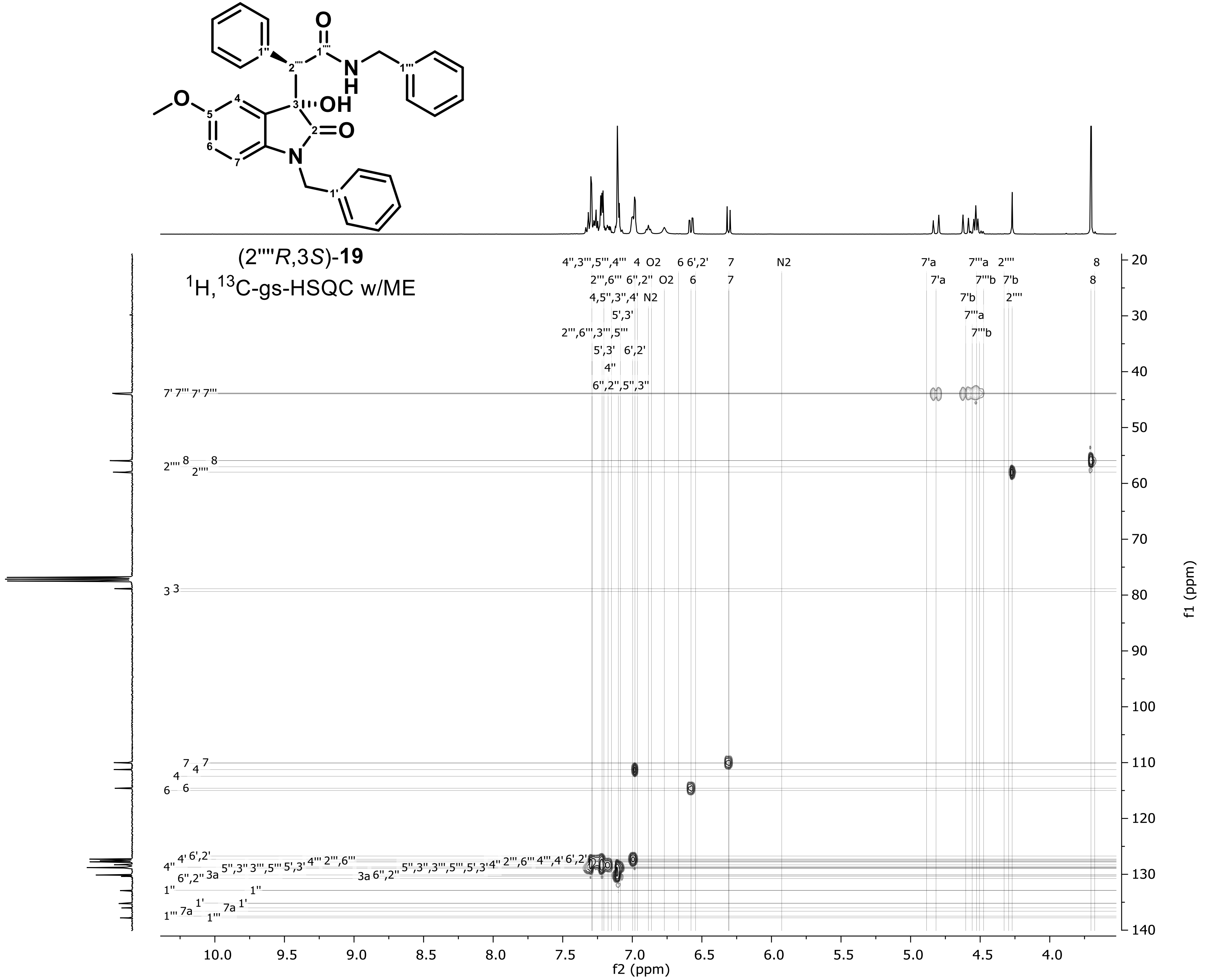

(2''''R,3S)-19
1H,13C-gs-HSQC w/ME
f2 (ppm)
f1 (ppm)

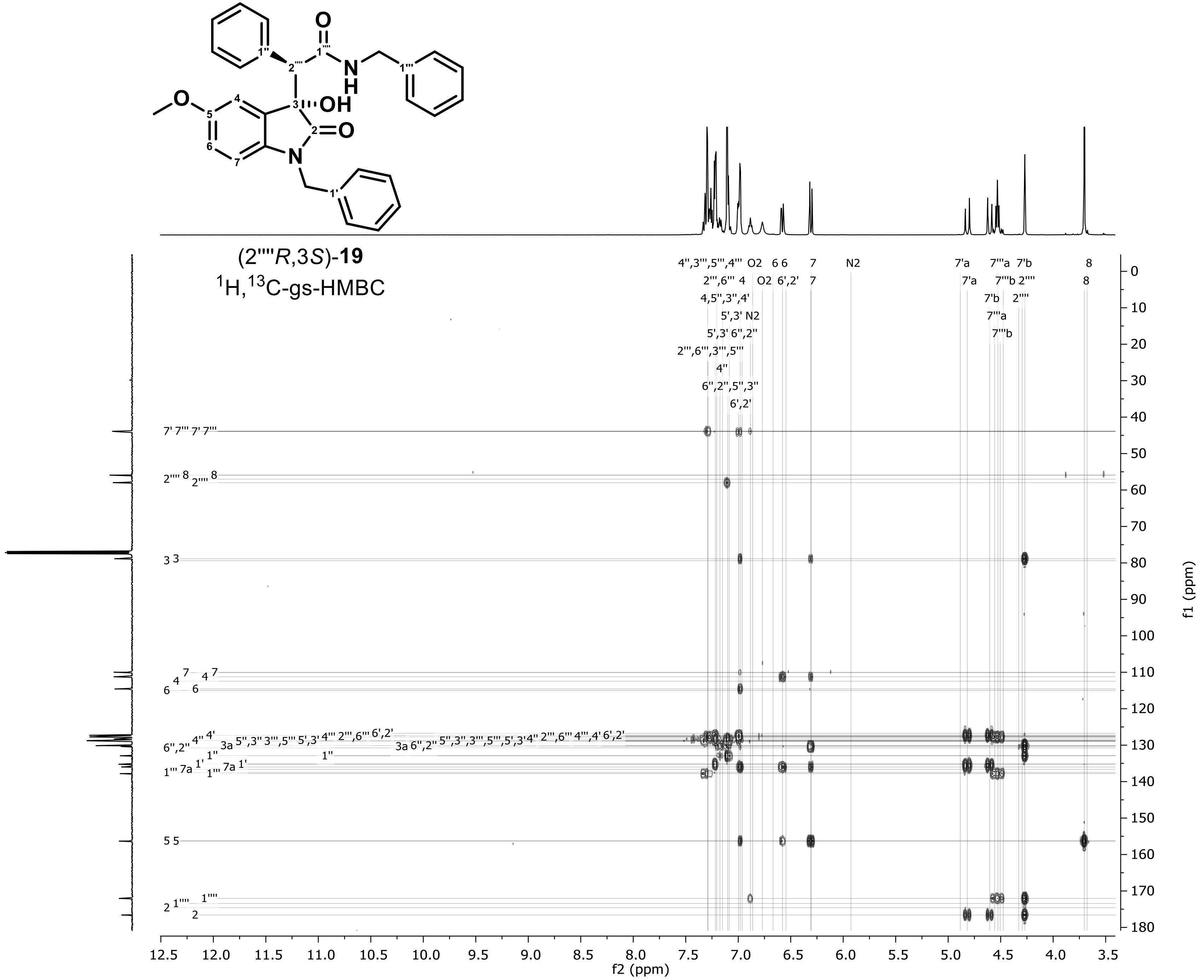

(2''''R,3S)-**19**

$^{1}H,^{13}C$-gs-HMBC

n) N,N'-Dibenzyl-2-(3-hydroxy-2-oxoindolin-3-yl)-2-*p*-anisylacetamide (20)

To a 25 ml round bottomed flask was added 2-(*p*-anisyl)acetic anhydride (118 mg, 0.375 mmol), *N*-benzylisatin (59.3 mg, 0.250 mmol) and (2*R*,3*S*)-HyperBTM (3.9 mg, 0.012 mmol). The mixture was cooled to 0 °C and CH_2Cl_2 (6.0 ml, 0.04 M) and Hünig's base (54.4 µL, 0.313 mmol) were added. The mixture was stirred at 0 °C for 3 h. Benzylamine (82.0 µL, 0.750 mmol) was added at 0 °C and the reaction was left to be stirred overnight at room temperature. 1,3,5-trimethoxybenzene (0.1 M soln in CH_2Cl_2, 500 µl, 0.05 mmol) was added and the solvent was removed under reduced pressure. Purification by column chromatography (20% – 30% EtOAc in Pentane) gave the title compound as mixture of two diastereomers as white solid (95.0 mg, 0.193 mmol, 77%, 92:8 dr).

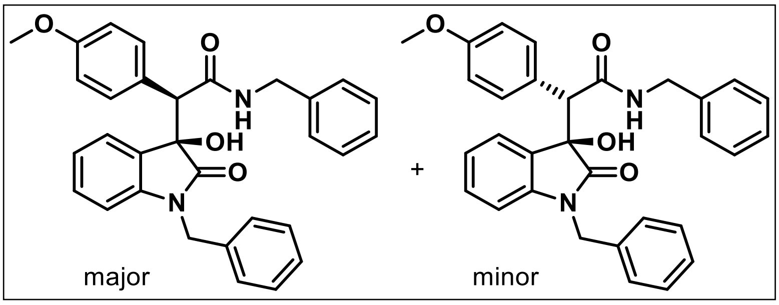

Major (2''''*R*,3*R*)-**20** and **minor** (2''''*S*,3*R*)-**20** (NMR analysed as 94:6 mixture of diastereomers, a sample of a single major diastereomer was obtained for the HPLC): **R*f*** 0.36 (1:1 Hexane:EtOAc), **Chiral HPLC analysis** Chiralpak IB (95:5 hexane:IPA, flow rate 2 mL·min^{-1}, 211 nm, 40 °C), t_R (2''''*R*,3*R*)-**20**: 29.0 min, t_R (2''''*S*,3*S*)-**20**: 45.5 min, >99:1 er; $\boldsymbol{\alpha}_D^{20}$ = −78.3 (*c* 3.71, $CHCl_3$); $\boldsymbol{\nu}_{max}$ (thin film) 3337 (O–H), 3057 (N–H), 1717 (C=O, lactam), 1611 (C=O amide), 1263 (OMe), 1179 (C–O alcohol); **^{1}H NMR** (400 MHz, $CDCl_3$) δ_H 7.52 (1H, app d, $^3J_{HH}$ = 7.3 Hz, ArC^4*H*), 7.34 – 7.25 (3H, m, PhC$^{3''',4''',5'''}$*H*), 7.24 – 7.19 (2H, m, PhC$^{2''',6'''}$*H*), 7.18 – 7.11 (2H, m, ArC^6*H*, PhC$^{4'}$*H*), 7.11 – 7.05 (2H, m, PhC$^{3',5'}$*H*), 7.01 (1H, ddd, $^3J_{HH}$ = 7.8 Hz, 7.4 Hz, $^4J_{HH}$ = 1.0 Hz, ArC^5*H*), 6.84 (1H, s (b), O*H*), 6.79 (2H, app d, $^3J_{HH}$ = 8.5 Hz, AnC$^{2'',6''}$*H*), 6.63 (2H, app d, $^3J_{HH}$ = 8.8 Hz, AnC$^{3'',5''}$*H*), 6.50 (2H, app d, $^3J_{HH}$ = 7.0 Hz, PhC$^{2',6'}$*H*), 6.41 (1H, d, $^3J_{HH}$ = 7.8 Hz, ArC^7*H*), 6.13 (1H, s (b), N*H*), 4.97 (1H, d, $^2J_{HH}$ = 16.0 Hz, NC$H_a$$H_b$-Ph), 4.52 (1H, dd, $^2J_{HH}$ = 15.0 Hz, $^3J_{HH}$ = 6.0 Hz, NHC$H_a$$H_b$-Ph), 4.45 (1H, dd, $^2J_{HH}$ = 15.0 Hz, $^3J_{HH}$ = 5.9 Hz, NHC$H_a$$H_b$-Ph), 4.35 (1H, s, C*H*-An), 4.23 (1H, d, $^2J_{HH}$ = 16.0 Hz, NC$H_a$$H_b$-Ph), 3.73 (3H, s, OC$H_3$); **^{13}C {^{1}H} NMR** (100 MHz, $CDCl_3$) δ_C 174.9 (*C*(O)NBn), 173.6 (*C*(O)NHBn), 159.8 (An$C^{4''}$OCH_3), 143.3 (ArC^{7a}), 137.6 (Ph$C^{1'''}$$CH_2$NH), 135.0 (Ph$C^{1'}$$CH_2$N), 131.8 (An$C^{2'',6''}$H), 129.9 (Ar$C^6$H), 129.1 (Ar$C^{3a}$), 128.8 (Ph$C^{3''',5'''}$H), 128.5 (Ph$C^{3',5'}$H), 127.7 (Ph$C^{4'''}$H), 127.6 (Ph$C^{2''',6'''}$H), 127.3 (Ph$C^{4'}$H), 126.7 (Ph$C^{2',6'}$H), 125.7 (Ar$C^4$H), 124.6 (An$C^{1''}$CH), 123.1 (Ar$C^5$H), 114.3 (An$C^{3'',5''}$H), 109.5 (Ar$C^7$H), 79.1 (*C*-OH), 56.2 (*C*H-An), 55.2 (O*C*H_3), 43.7 (NH*C*H_2-Ph, N*C*H_2-Ph); **HRMS** (ESI$^+$) *m/z* calcd for $[M]^+$ $C_{31}H_{29}N_2O_4$ 493.2122, found 493.2117 (−1.0 ppm).

(±)-anti-**20**

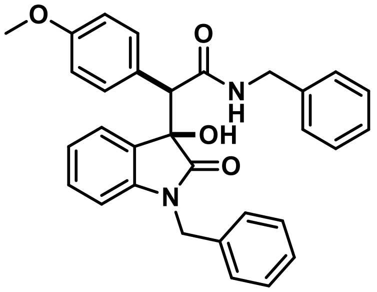

PDA Ch1 211nm

Peak#	Ret. Time	Area%
1	30.709	49.549
2	44.492	50.451
Total		100.000

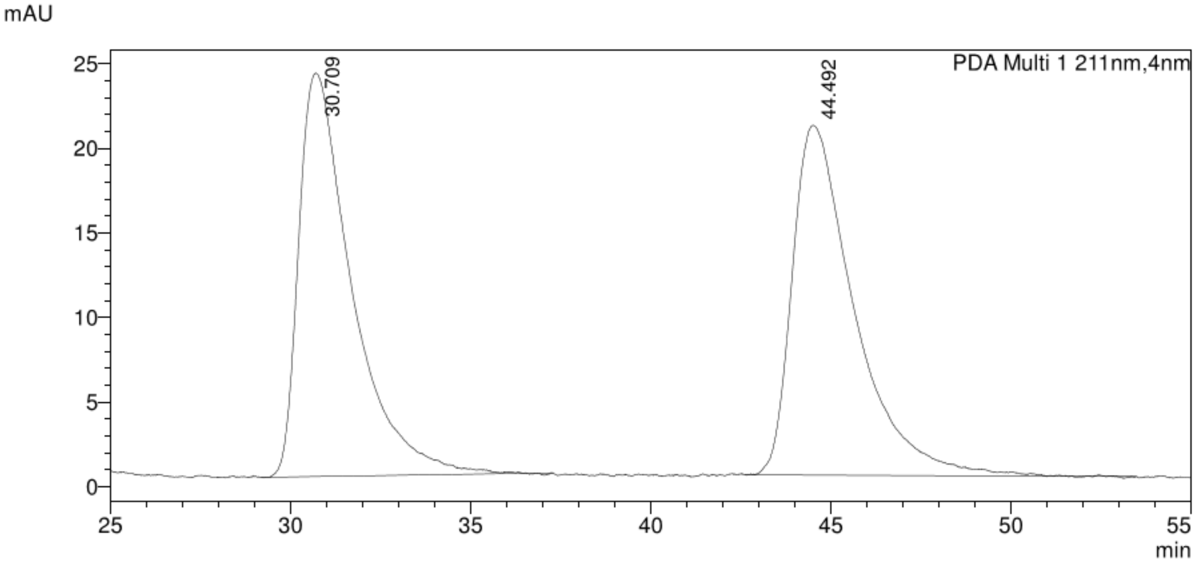

(–)-(2''''*R*,3*R*)-**20**

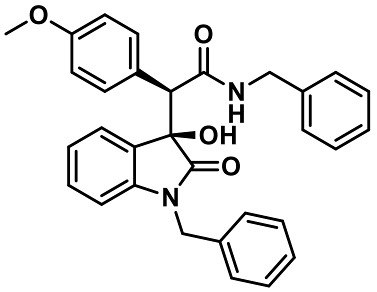

PDA Ch1 211nm

Peak#	Ret. Time	Area%
1	29.016	99.531
2	45.461	0.469
Total		100.000

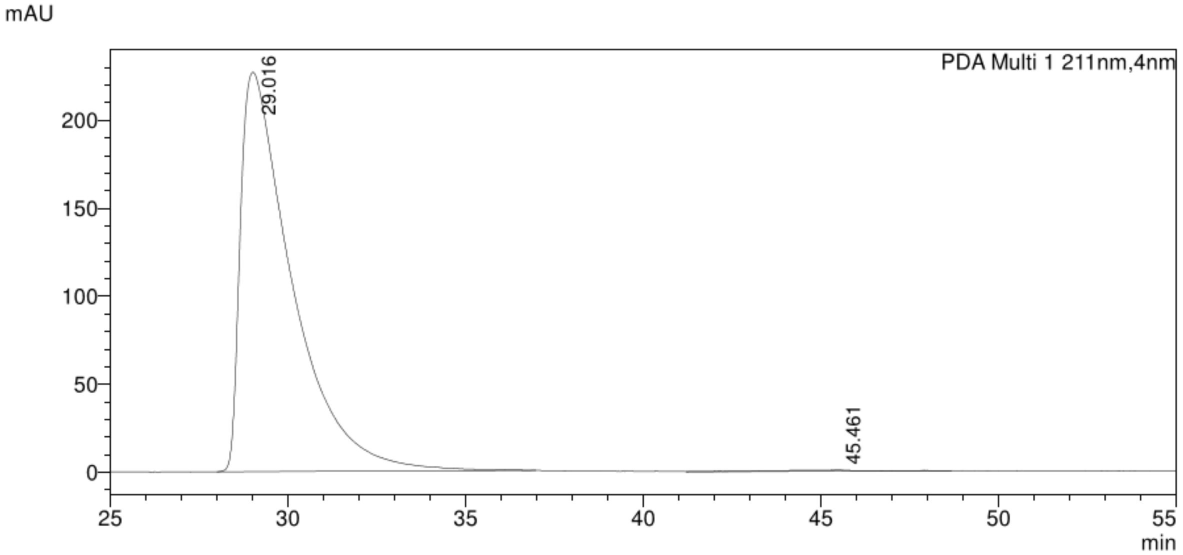

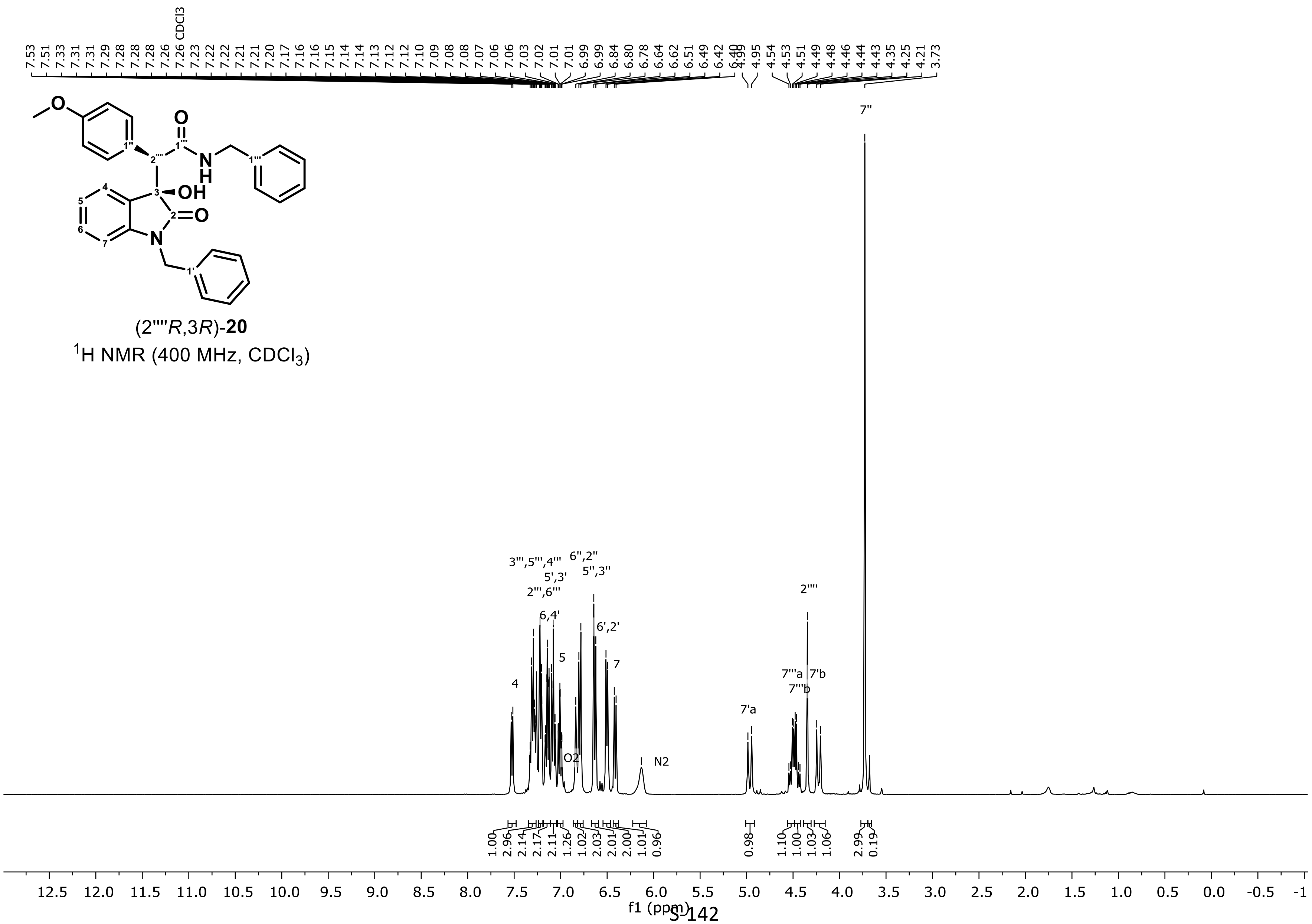
7.53 7.51 7.33 7.31 7.31 7.29 7.28 7.28 7.26 7.26 CDCl3 7.23 7.22 7.22 7.21 7.21 7.20 7.17 7.16 7.16 7.15 7.14 7.14 7.13 7.12 7.12 7.10 7.09 7.08 7.08 7.07 7.06 7.06 7.03 7.02 7.01 7.01 6.99 6.99 6.84 6.80 6.78 6.64 6.62 6.51 6.49 6.42 6.40 4.99 4.95 4.54 4.53 4.51 4.49 4.48 4.46 4.44 4.43 4.35 4.25 4.21 3.73
(2''''R,3R)-20
^{1}H NMR (400 MHz, CDCl$_3$)
7''
3''',5''',4'''
6'',2''
5',3'
5'',3''
2''',6'''
6,4'
6',2'
5
7
4
O2
N2
7'a
7'''a
7'b
7'''b
2''''
1.00 2.96 2.14 2.17 2.11 1.26 1.02 2.03 2.01 2.00 1.01 0.96 0.98 1.10 1.00 1.03 1.06 2.99 0.19
f1 (ppm)

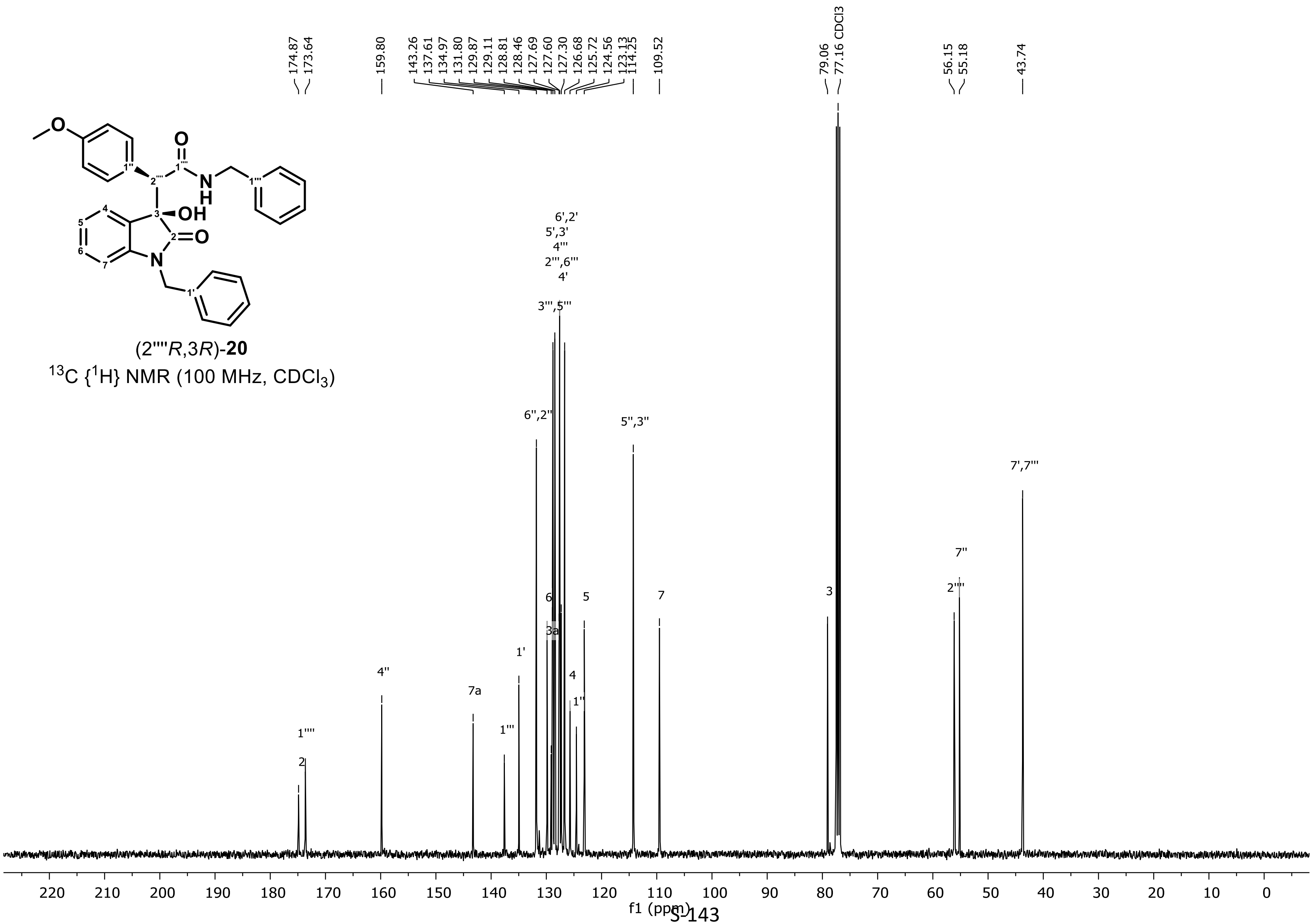
174.87
173.64
159.80
143.26
137.61
134.97
131.80
129.87
129.11
128.81
128.46
127.69
127.60
127.30
126.68
125.72
124.56
123.13
114.25
109.52
79.06
77.16 CDCl3
56.15
55.18
43.74
(2''''R,3R)-20
$^{13}C\{^{1}H\}$ NMR (100 MHz, $CDCl_3$)
f1 (ppm)

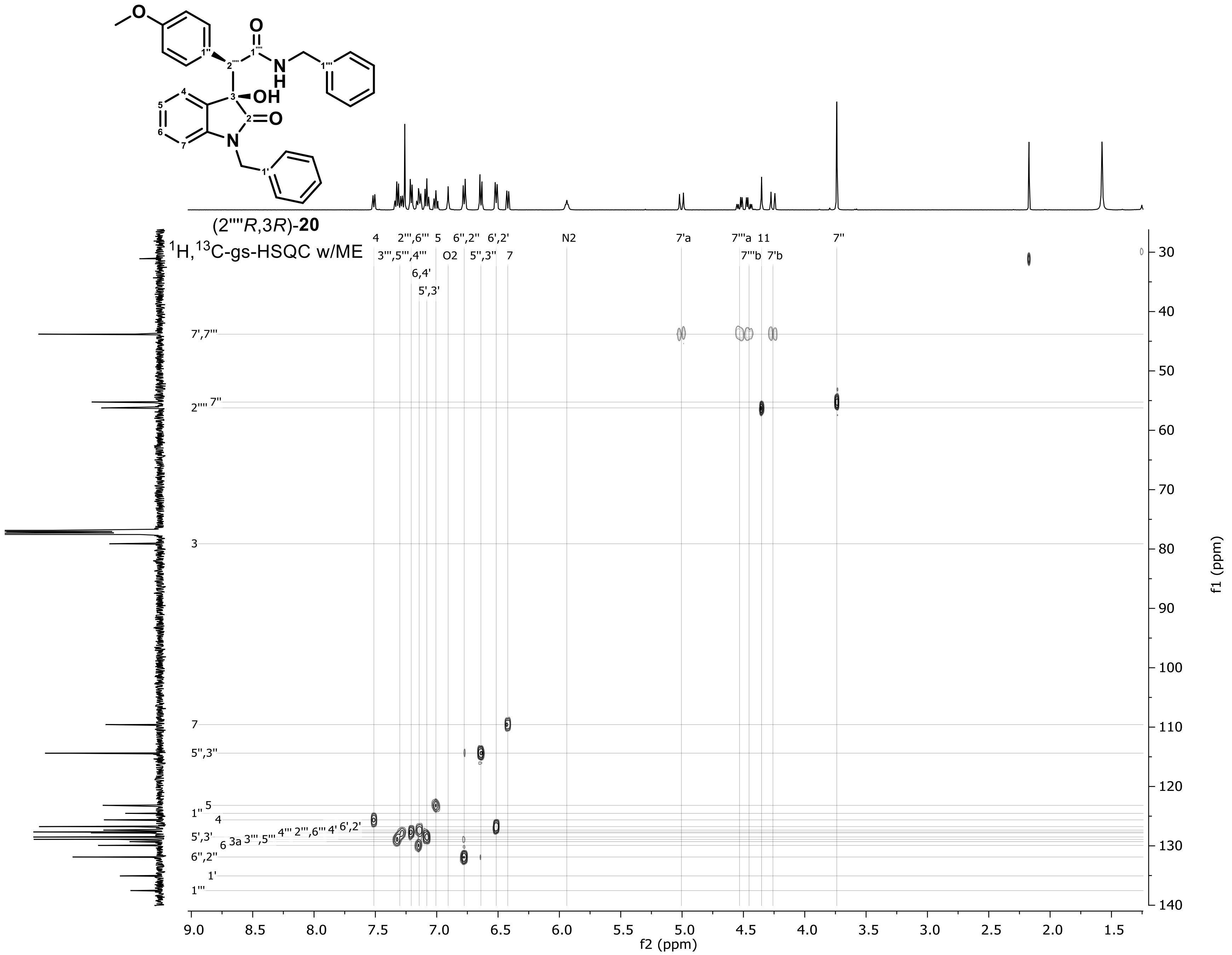
(2''''R,3R)-20
1H,13C-gs-HSQC w/ME
f2 (ppm)
f1 (ppm)

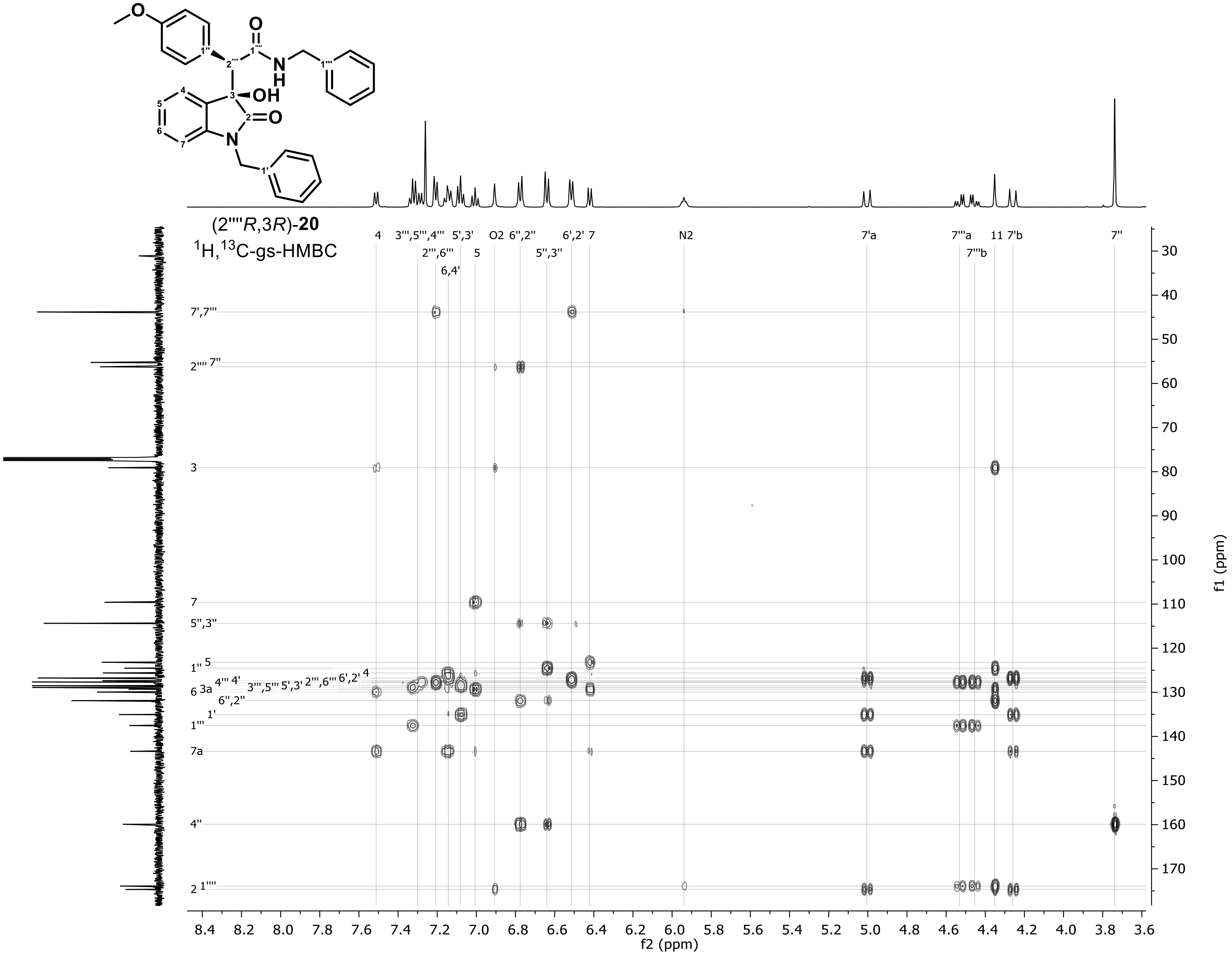

(2''''R,3R)-20
^{1}H,^{13}C-gs-HMBC
4 3''',5''',4''' 5',3' O2 6'',2'' 6',2' 7 N2 7'a 7'''a 11 7'b 7''
2''',6''' 5 5'',3'' 7'''b
6,4'
7',7'''
2'''' 7''
3
7
5'',3''
1'' 5
6 3a 4''' 4' 3''',5''' 5',3' 2''',6''' 6',2' 4
6'',2''
1'
1'''
7a
4''
2 1''''
f2 (ppm)
f1 (ppm)

o) N,N′-Dibenzyl-2-(3-hydroxy-2-oxoindolin-3-yl)-2-*p*-tolylacetamide (21)

To a 25 ml round bottomed flask was added 2-(*p*-tolyl)acetic anhydride (106 mg, 0.375 mmol), *N*-benzylisatin (59.3 mg, 0.250 mmol) and (2*R*,3*S*)-HyperBTM (3.9 mg, 0.012 mmol). The mixture was cooled to 0 °C and CH_2Cl_2 (6.0 ml, 0.04 M) and Hünig's base (54.4 µL, 0.313 mmol) were added. The mixture was stirred at 0 °C for 3 h. Benzylamine (82.0 µL, 0.750 mmol) was added at 0 °C and the reaction was left to be stirred overnight at room temperature. 1,3,5-trimethoxybenzene (0.1 M soln in CH_2Cl_2, 500 µl, 0.05 mmol) was added and the solvent was removed under reduced pressure. Purification by column chromatography (15% – 25% EtOAc in Pentane) gave the title compound in two fractions (major diastereomer as white solid (68.1 mg, 57%) and minor diastereomer as white solid (10.5 mg, 9%); combined (78.6 mg, 0.165 mmol, 66%, 88:12 dr).

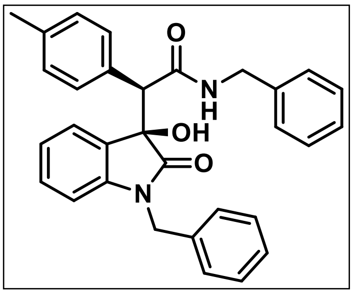

Major (2′′′′*R*,3*R*)-**21**: **Chiral HPLC analysis** Chiralpak IB (95:5 hexane:IPA, flow rate 2 mL·min^{-1}, 211 nm, 40 °C) t_R (2′′′′*R*,3*R*)-**21**: 19.6 min, t_R (2′′′′*S*,3*S*)-**21**: 30.4 min, >99:1; α_D^{20} = −46.0 (*c* 0.62, $CHCl_3$); ν_{max} (thin film) 3347 (O–H), 3059 (N–H), 3030 (N–H), 2920, 1717 (C=O lactam), 1612 (C=O amide), 1468 (CH3), 1175 (C–O alcohol); **^{1}H NMR** (400 MHz, $CDCl_3$) δ_H 7.52 (1H, dd, $^3J_{HH}$ = 7.5 Hz, $^4J_{HH}$ = 1.3 Hz, ArC^4*H*), 7.35 – 7.25 (3H, m, PhC$^{3''',4''',5'''}$*H*), 7.23 – 7.19 (2H, m, PhC$^{2''',6'''}$*H*), 7.17 – 7.12 (1H, m, PhC$^{4'}$*H*), 7.14 (1H, app td, $^3J_{HH}$ = 7.8 Hz, $^4J_{HH}$ = 1.3 Hz, ArC^6*H*), 7.10 – 7.05 (2H, m, PhC$^{3',5'}$*H*), 7.00 (1H, app td, $^3J_{HH}$ = 7.6 Hz, $^4J_{HH}$ 1.0 Hz, ArC^5*H*), 6.95 – 6.91 (2H, m, TolC$^{3'',5''}$*H*), 6.88 (1H, s, O*H*), 6.79 – 6.75 (2H, m, TolC$^{2'',6''}$*H*), 6.58 – 6.53 (2H, m, PhC$^{2',6'}$*H*), 6.41 (1H, app dt, $^3J_{HH}$ = 7.8 Hz, $^4J_{HH}$ = $^5J_{HH}$ = 0.7 Hz, ArC^7*H*), 5.99 (1H, app t, $^3J_{HH}$ = 5.9 Hz, N*H*), 4.96 (1H, d, $^2J_{HH}$ = 16.0 Hz, NC*H*$_a$H$_b$-Ph), 4.52 (1H, dd, $^2J_{HH}$ = 14.9 Hz, $^3J_{HH}$ = 6.0 Hz, NHC*H*$_a$H$_b$-Ph), 4.46 (1H, dd, $^2J_{HH}$ = 14.9 Hz, $^3J_{HH}$ = 5.9 Hz, NHCH$_a$*H*$_b$-Ph), 4.36 (1H, s, C*H*-Tol), 4.26 (1H, d, $^2J_{HH}$ = 16.0 Hz, NCH$_a$*H*$_b$-Ph), 2.30 (3H, s, TolC$^{4''}$C*H*$_3$); **^{13}C {^{1}H} NMR** (101 MHz, $CDCl_3$) δ_C 174.8 (*C*(O)NBn), 173.7 (*C*(O)NHBn), 143.3 (Ar*C*^{7a}), 138.5 (Tol*C*$^{4''}$CH$_3$), 137.6 (Ph*C*$^{1'''}$CH$_2$NH), 135.1 (Ph*C*1′CH$_2$N), 130.5 (Tol*C*$^{2'',6''}$H), 129.9 (Ar*C*^{6}H), 129.7$_2$ (Tol*C*$^{1''}$CH), 129.6$_6$ (Tol*C*$^{5''3''}$H), 129.2 (Ar*C*^{3a}), 128.9 (Ph*C*$^{3''',5'''}$H), 128.5 (Ph*C*$^{3',5'}$H), 127.8 (Ph*C*$^{4'''}$H), 127.6 (Ph*C*$^{2''',6'''}$H), 127.3 (Ph*C*$^{4'}$H), 126.8 (Ph*C*$^{2',6'}$H), 125.8 (Ar*C*^{4}H), 123.1 (Ar*C*^{5}H), 109.5 (Ar*C*^{7}H), 79.1 (*C*-OH), 56.7 (*C*H-Tol), 43.8$_5$ and 43.7$_9$ (NH*C*H$_2$-Ph, N*C*H$_2$-Ph), 21.4 (*C*H$_3$); **HRMS** (ESI$^+$) *m/z* calcd for [M]$^+$ $C_{31}H_{29}N_2O_3$ 477.2173, found 477.2159 (-3.0 ppm).

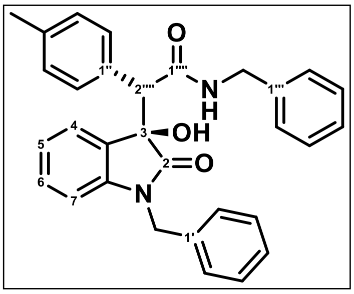

Minor (2′′′′*S*,3*R*)-**21**: **Chiral HPLC analysis** Chiralpak IB (95:5 hexane:IPA, flow rate 2 mL·min^{-1}, 211 nm, 40 °C) t_R (2′′′′*S*, 3*R*)-**21**: 22.4 min, t_R (2′′′′*R*, 3*S*)-**21**: 30.3 min, 98:2; α_D^{20} = −26.4 (*c* 1.02, $CHCl_3$); ν_{max} (thin film) 3289 (O–H), 3059 (N–H), 3030 (N–H), 2922 (C–H), 1697 (C=O lactam), 1612 (C=O amide), 1467 (CH3), 1178 (C–O alcohol); **^{1}H NMR** (400 MHz, $CDCl_3$) δ_H 7.37 (1H, dd, $^3J_{HH}$ = 7.3 Hz, $^4J_{HH}$ = 1.3 Hz, ArC^4*H*), 7.35 – 7.27 (5H, m, PhC$^{2''',3''',4''',5''',6'''}$*H*), 7.25 – 7.20 (3H, m, PhC$^{3',4',5'}$*H*), 7.10 (1H, s(br), O*H*), 7.06 (1H, app dt, $^3J_{HH}$ = 7.6 Hz, $^4J_{HH}$ = 1.3 Hz, ArC^6*H*), 7.07 – 7.02 (2H, m, PhC$^{2',6'}$*H*), 6.98 (1H, ddd, $^3J_{HH}$ = 7.6 Hz, 7.3 Hz, $^4J_{HH}$ 1.1 Hz, ArC^5*H*), 6.98 – 6.93 (2H, m, TolC$^{2'',6''}$*H*), 6.90 – 6.83 (2H, m, TolC$^{3'',5''}$*H*), 6.60 (1H, app t, $^3J_{HH}$ = 5.9 Hz, N*H*), 6.42 (1H, app dt, $^3J_{HH}$ = 7.7, $^4J_{HH}$ = $^5J_{HH}$ = 0.8 Hz, ArC^7*H*), 4.87 (1H, d, $^2J_{HH}$ 15.8 Hz, NC*H*$_a$H$_b$-Ph), 4.64 (1H, d, $^2J_{HH}$ = 15.8 Hz, NCH$_a$*H*$_b$-Ph), 4.56 (1H, dd, $^2J_{HH}$ = 15.0 Hz, $^3J_{HH}$ 5.9 Hz, NHC*H*$_a$H$_b$-Ph), 4.50 (1H, dd, $^2J_{HH}$ = 15.0 Hz, $^3J_{HH}$ = 5.7 Hz, NHCH$_a$*H*$_b$-Ph), 4.22 (1H, s, C*H*-Tol), 2.22 (3H, s, TolC$^{4''}$C*H*$_3$); **^{13}C {^{1}H} NMR** (101 MHz, $CDCl_3$) δ_C 176.8 (*C*(O)NBn), 172.6 (*C*(O)NHBn), 142.8 (Ar*C*^{7a}), 138.1 (Tol*C*$^{4''}$CH$_3$), 137.8 (Ph*C*$^{1'''}$CH$_2$NH), 135.3 (Ph*C*$^{1'}$CH$_2$N), 130.1 (Tol*C*$^{2'',6''}$H), 129.8 (Ar*C*^{6}H), 129.7 (Tol*C*$^{1''}$CH), 129.6 (Tol*C*$^{3'',5''}$H), 129.3 (Ar*C*^{3a}), 128.8 (Ph*C*$^{3''',5'''}$H), 128.7 (Ph*C*$^{3',5'}$H), 127.8 (Ph*C*$^{2''',6'''}$H), 127.6$_0$ and 127.5$_8$ (Ph*C*$^{4'}$H and Ph*C*$^{4'''}$H), 127.4 (Ph*C*$^{2',6'}$H), 124.2 (Ar*C*^{4}H), 123.1 (Ar*C*^{5}H), 109.4 (Ar*C*^{7}H), 78.6 (*C*-OH), 57.0 (*C*H-Tol), 43.9$_1$ and 43.8$_7$ (N*C*H$_2$-Ph and NH*C*H$_2$-Ph), 21.2 (TolC$^{4''}$*C*H$_3$); **HRMS** (ESI$^+$) *m/z* calcd for [M]$^+$ $C_{31}H_{29}N_2O_3$ 477.2173, found 477.2162 (−2.3 ppm).

(±)-anti-**21**

PDA Ch1 211nm

Peak#	Ret. Time	Area%
1	19.630	49.423
2	30.418	50.577
Total		100.000

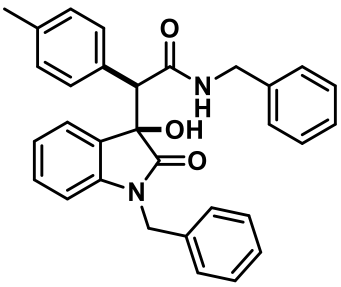

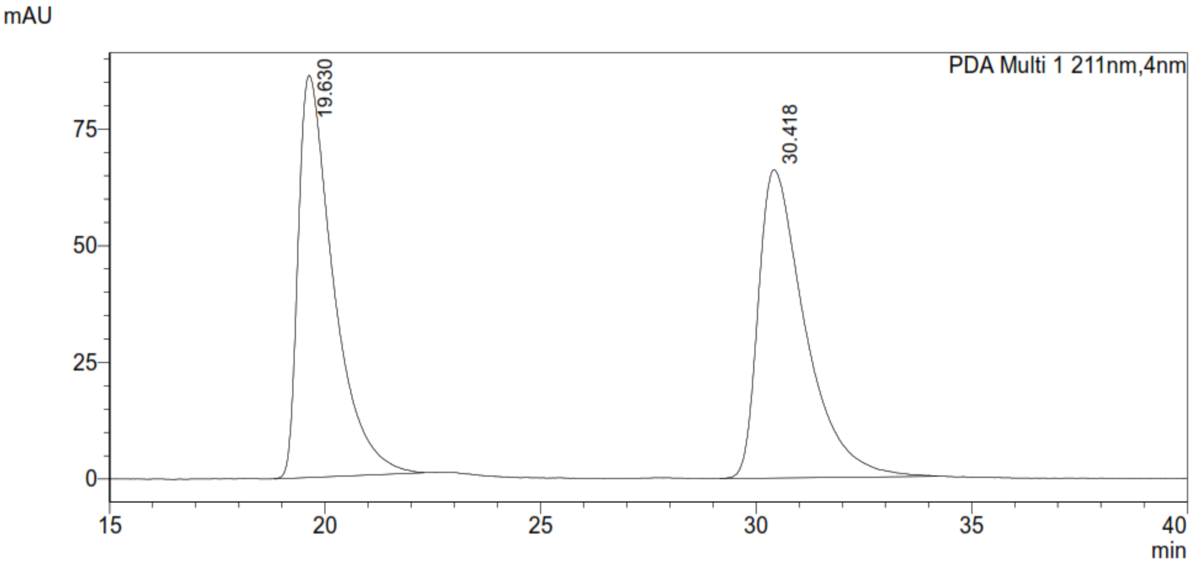

(–)-(2''''*R*,3*R*)-**21**

PDA Ch1 211nm

Peak#	Ret. Time	Area%
1	19.298	99.804
2	31.169	0.196
Total		100.000

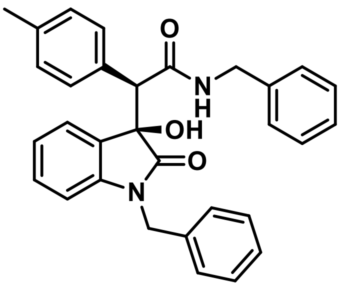

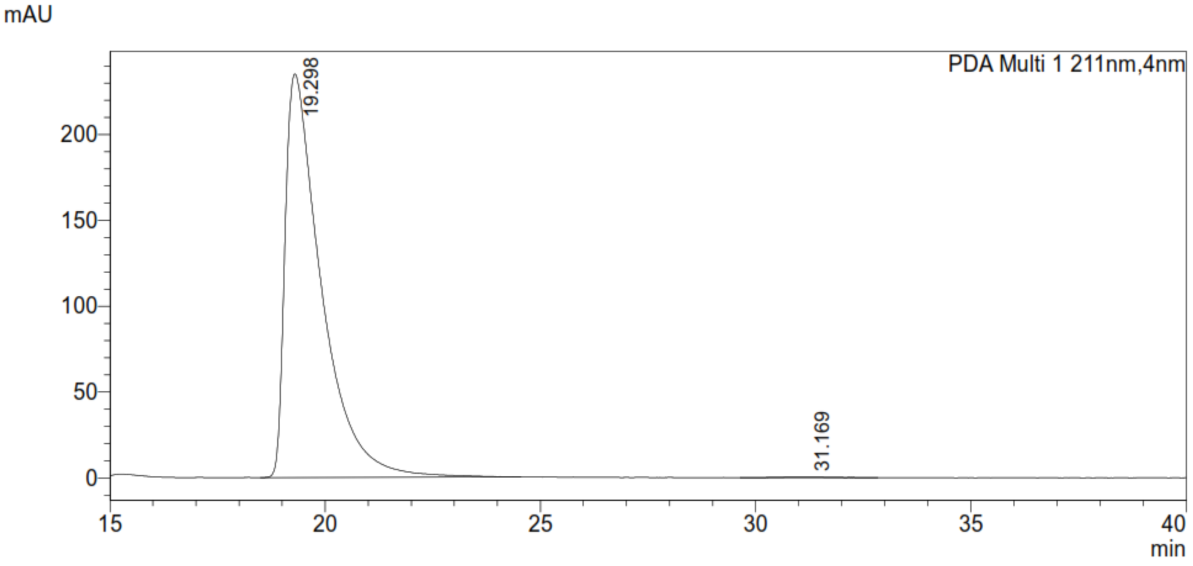

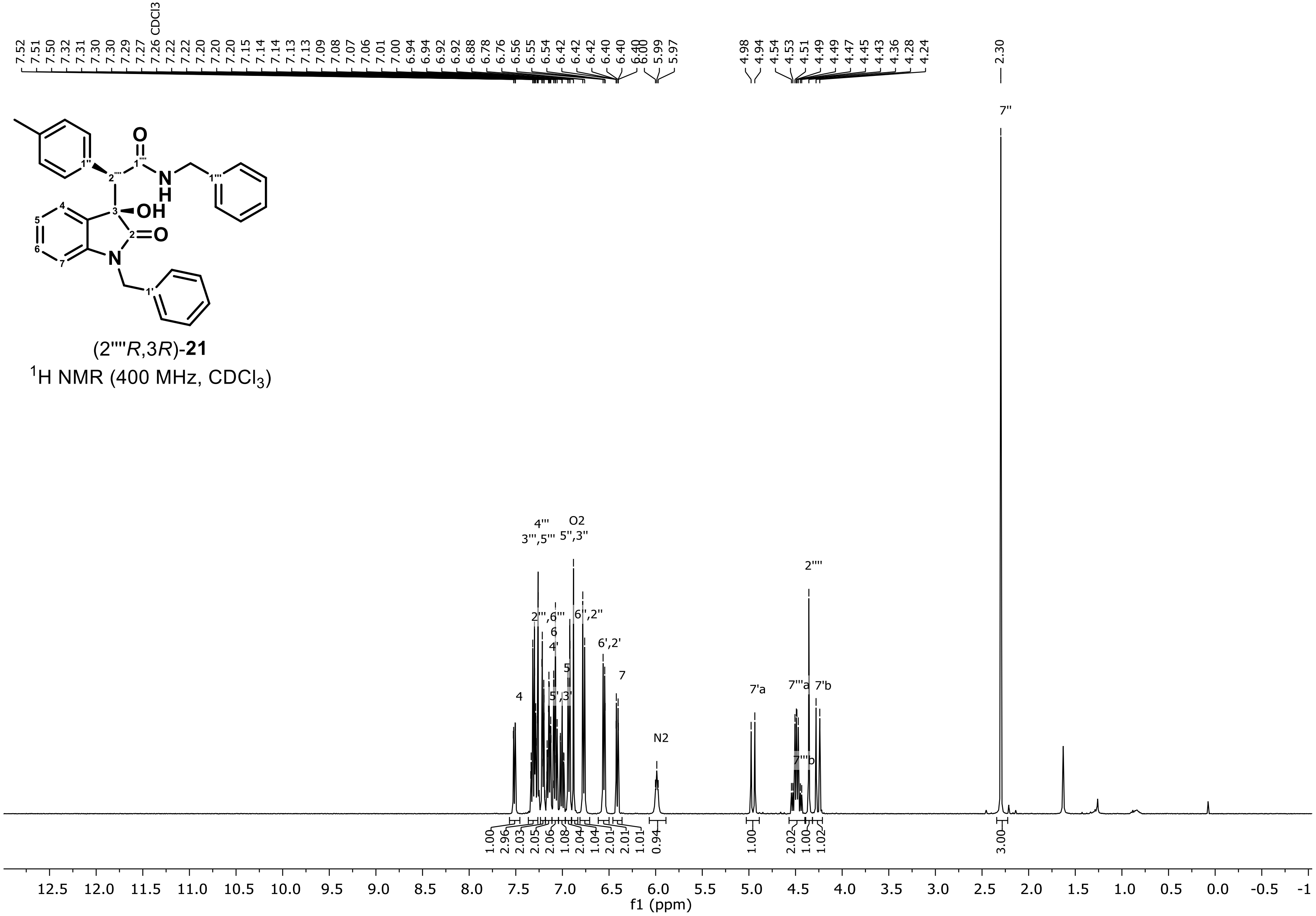
(2''''R,3R)-21
1H NMR (400 MHz, CDCl3)
f1 (ppm)
CDCl3
4
2''',6'''
6
4'
5
5',3'
6'',2''
6',2'
7
N2
7'a
7'''a
7'b
7'''b
2''''
7''
4'''
3''',5'''
O2
5'',3''

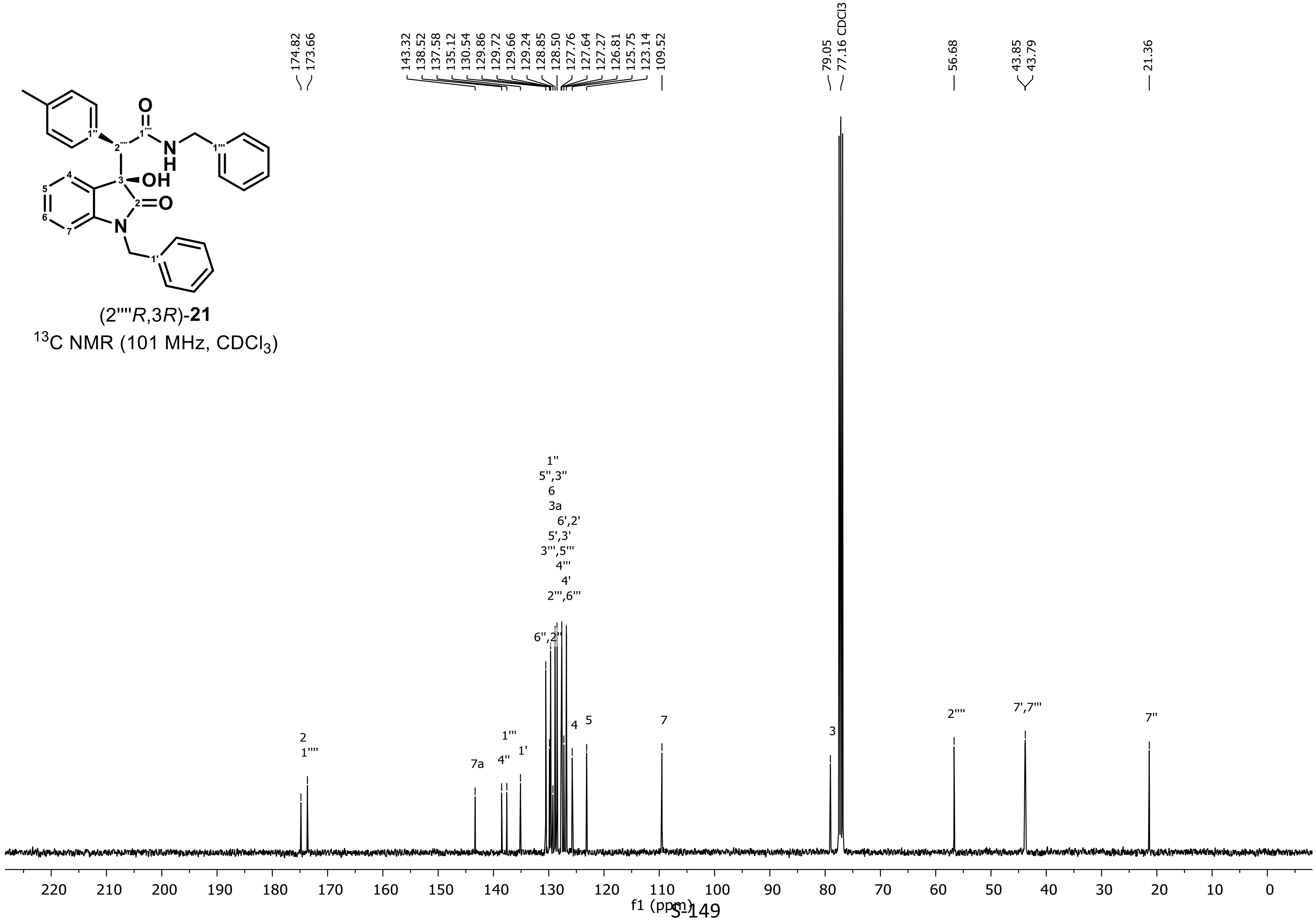

(2''''*R*,3*R*)-**21**

^{13}C NMR (101 MHz, $CDCl_3$)

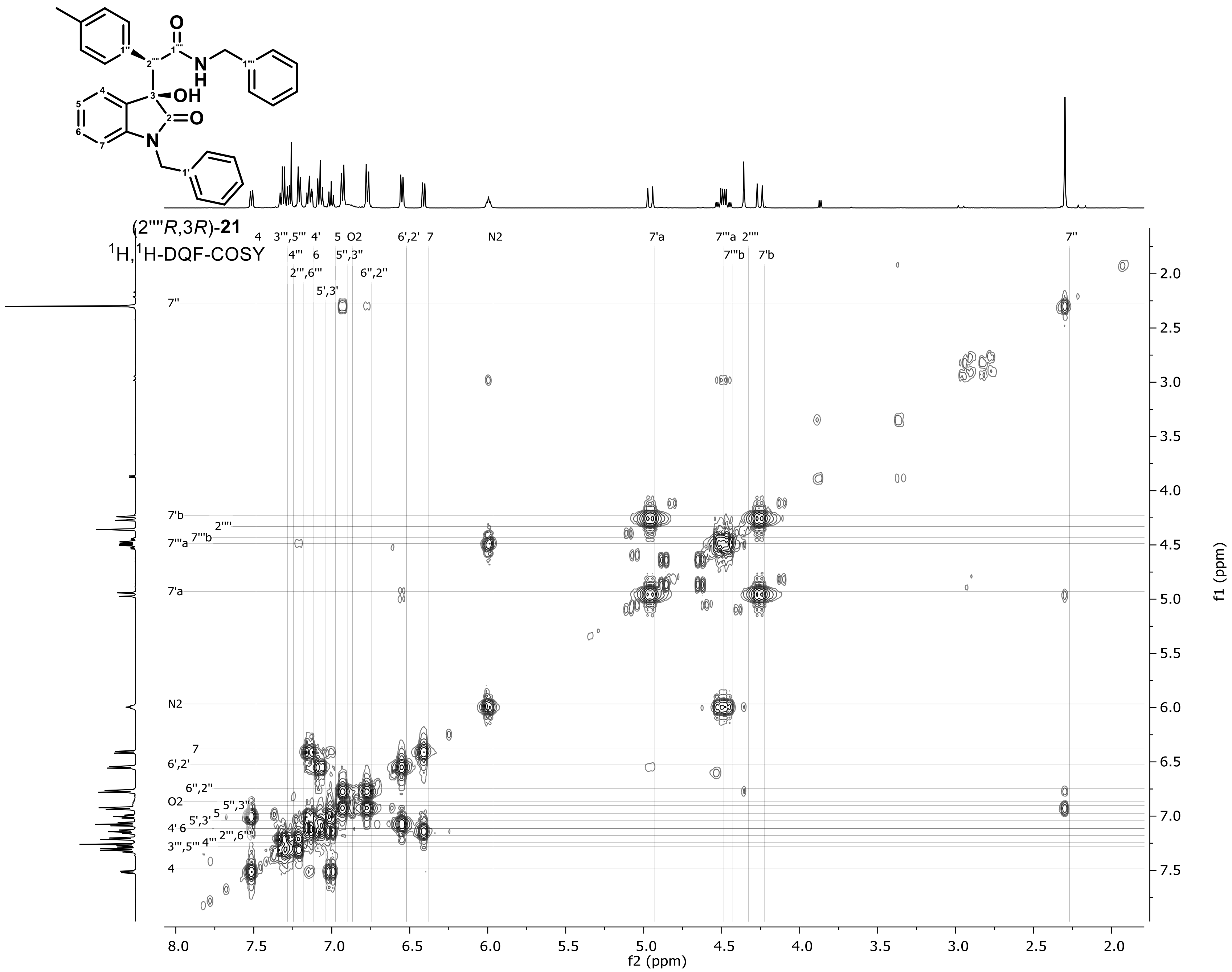

(2''''R,3R)-21
^{1}H,^{1}H-DQF-COSY
f2 (ppm)
f1 (ppm)

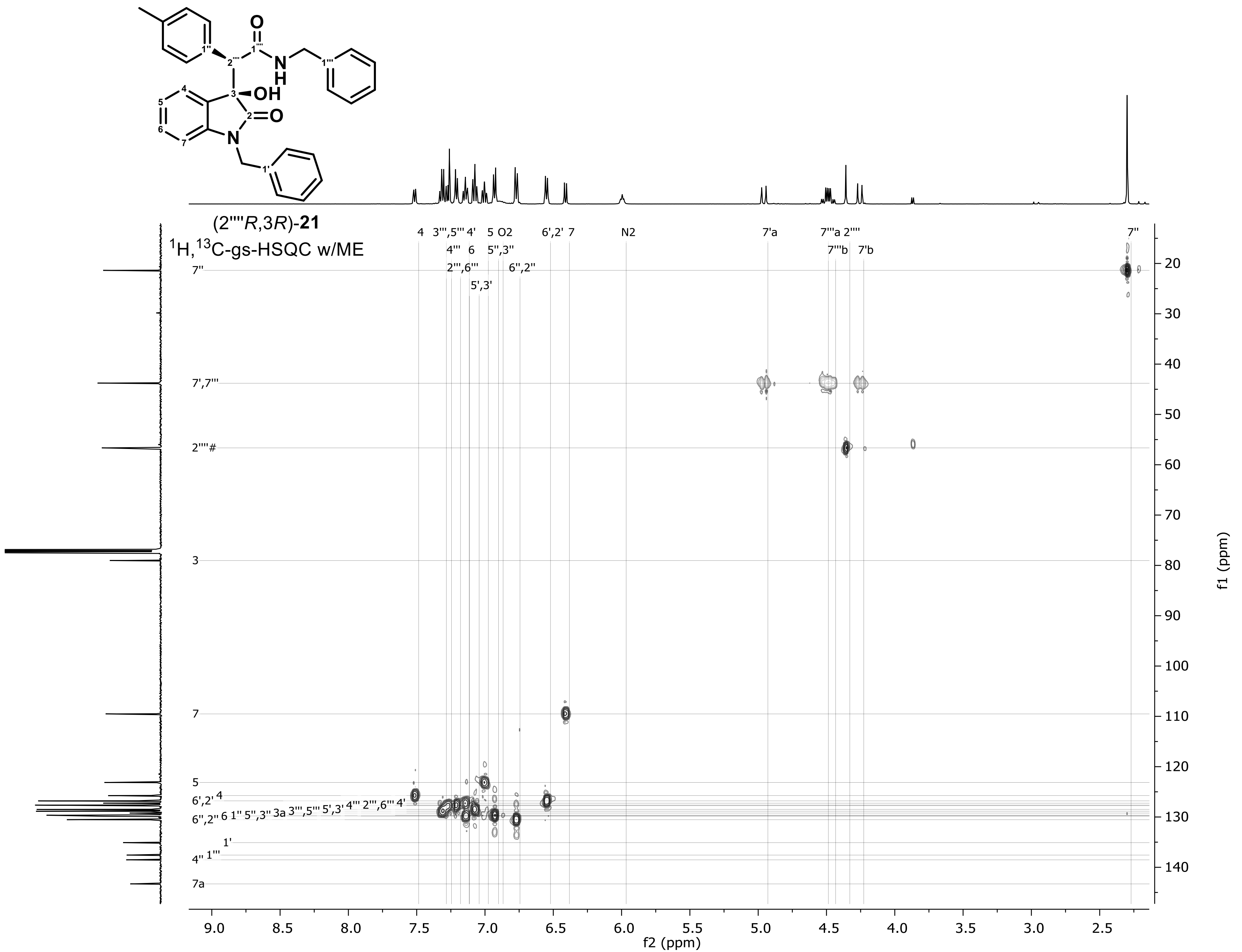

(2''''R,3R)-21
^{1}H,^{13}C-gs-HSQC w/ME
f2 (ppm)
f1 (ppm)

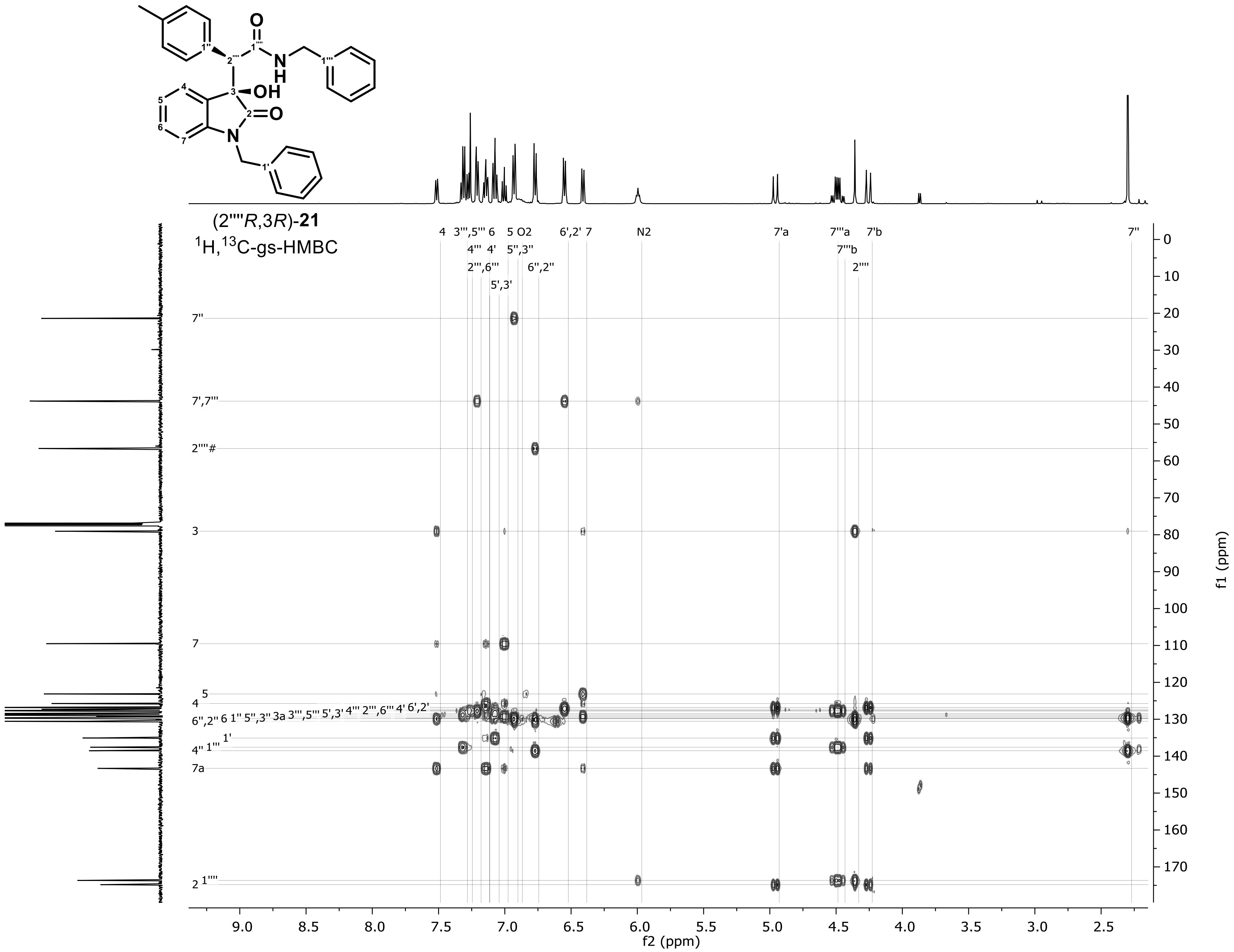
(2''''R,3R)-21
$^1H,^{13}C$-gs-HMBC
f2 (ppm)
f1 (ppm)

(±)-syn-**21**

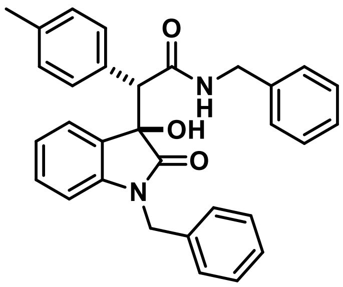

PDA Ch1 211nm

Peak#	Ret. Time	Area%
1	22.410	49.861
2	30.305	50.139
Total		100.000

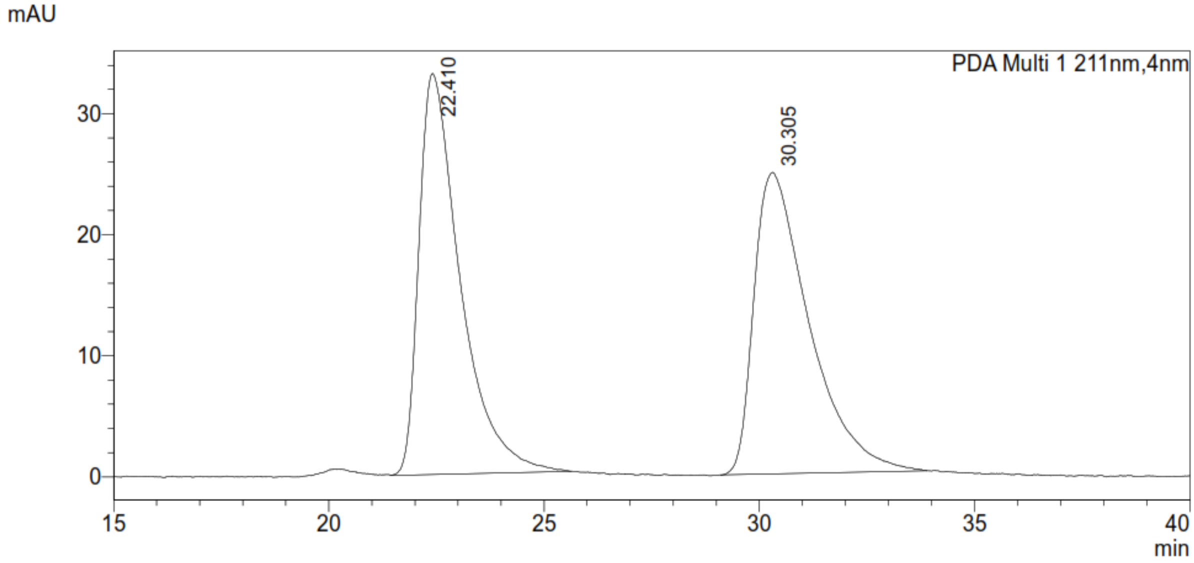

(–)-(2''''*S*,3*R*)-**21**

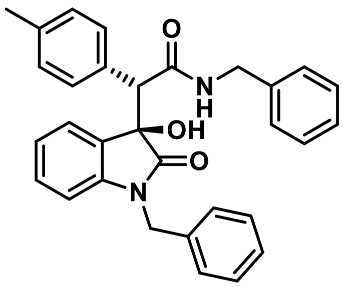

PDA Ch1 211nm

Peak#	Ret. Time	Area%
1	21.843	99.033
2	30.980	0.967
Total		100.000

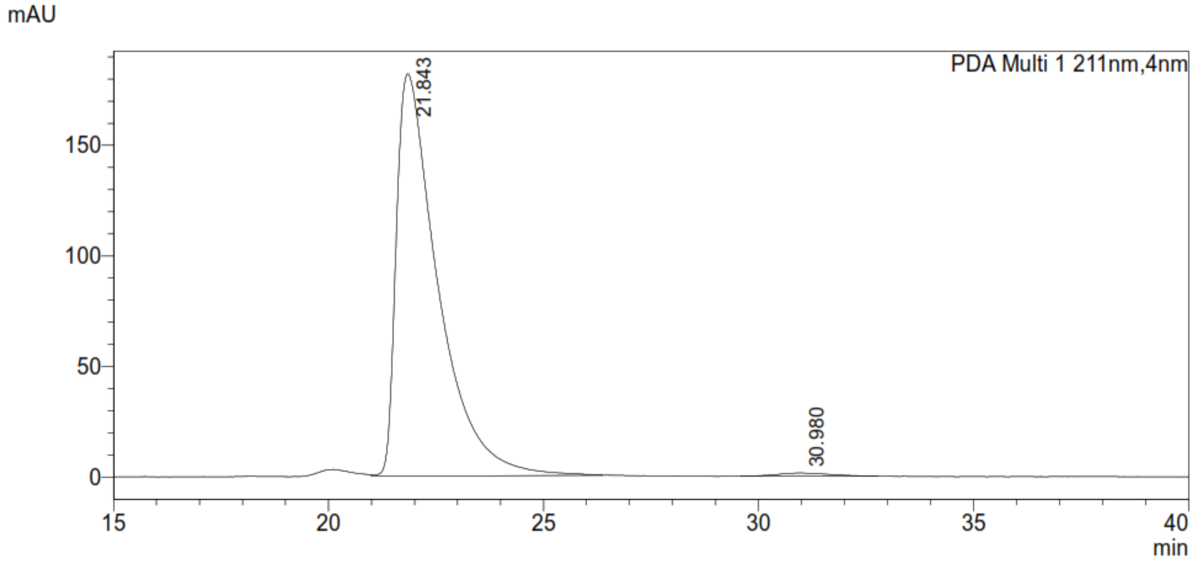

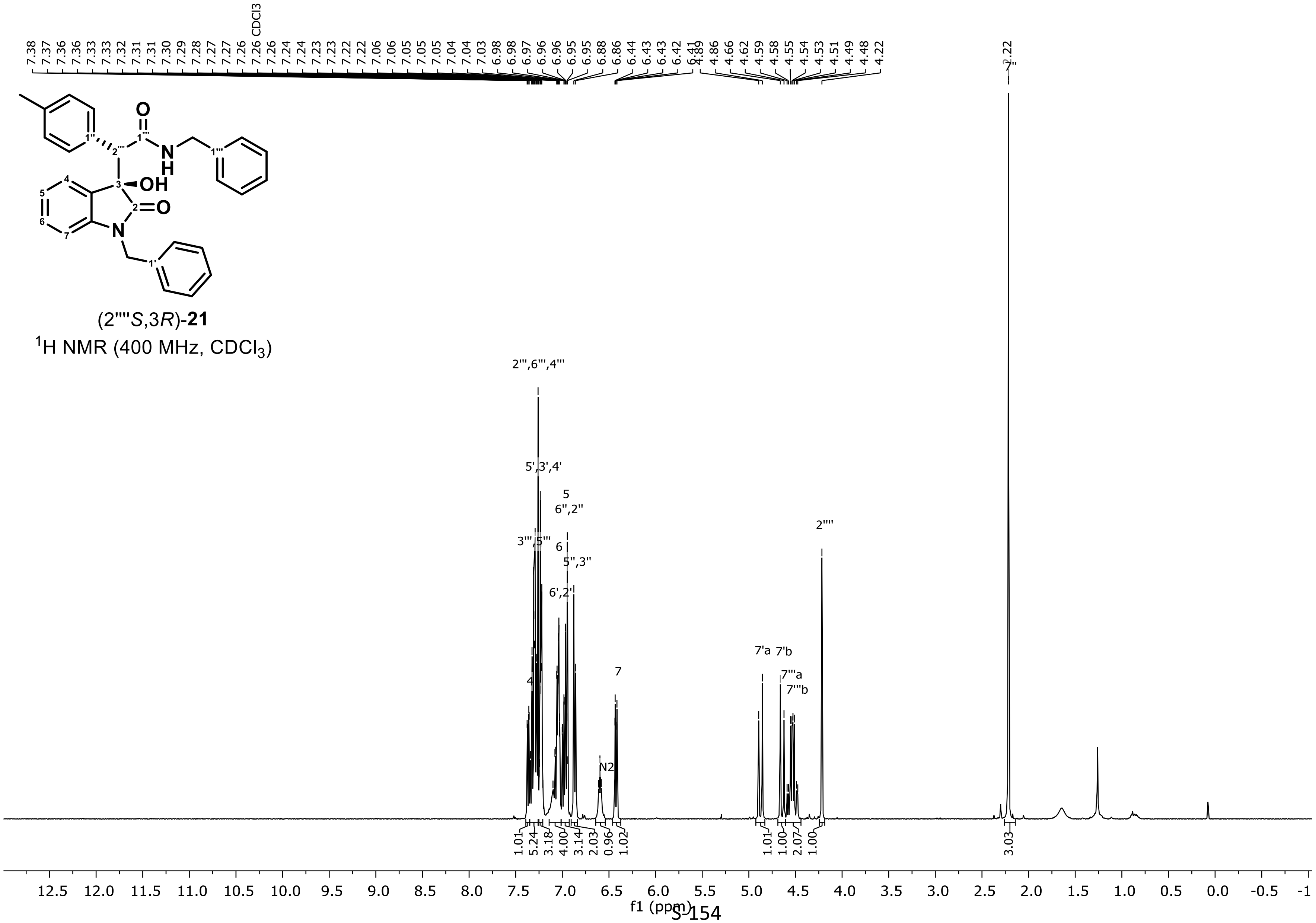

(2''''*S*,3*R*)-**21**

^{1}H NMR (400 MHz, $CDCl_3$)

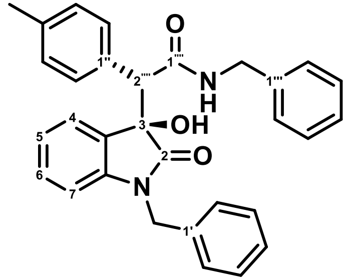

(2''''S,3R)-**21**

^{13}C NMR (101 MHz, $CDCl_3$)

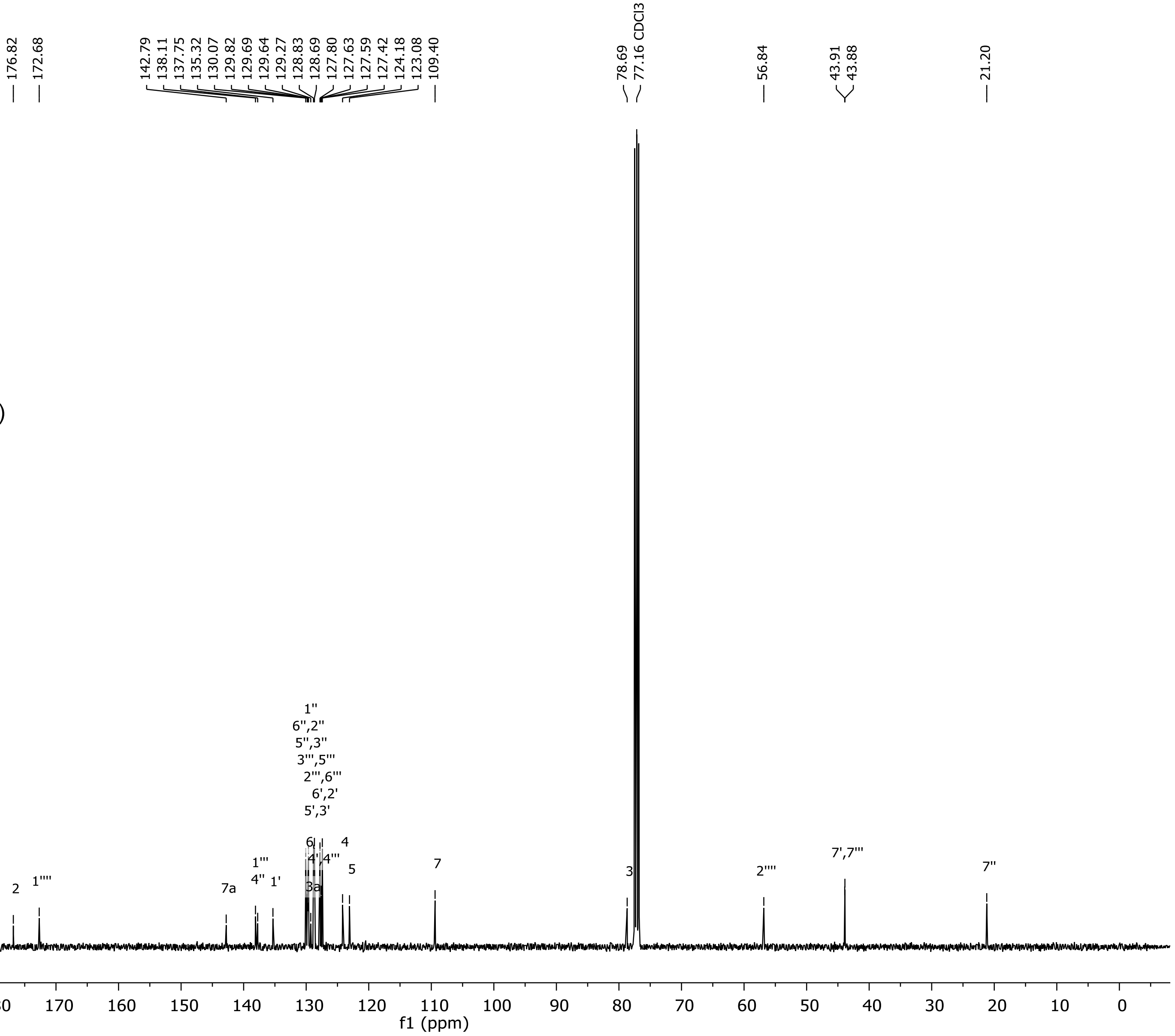

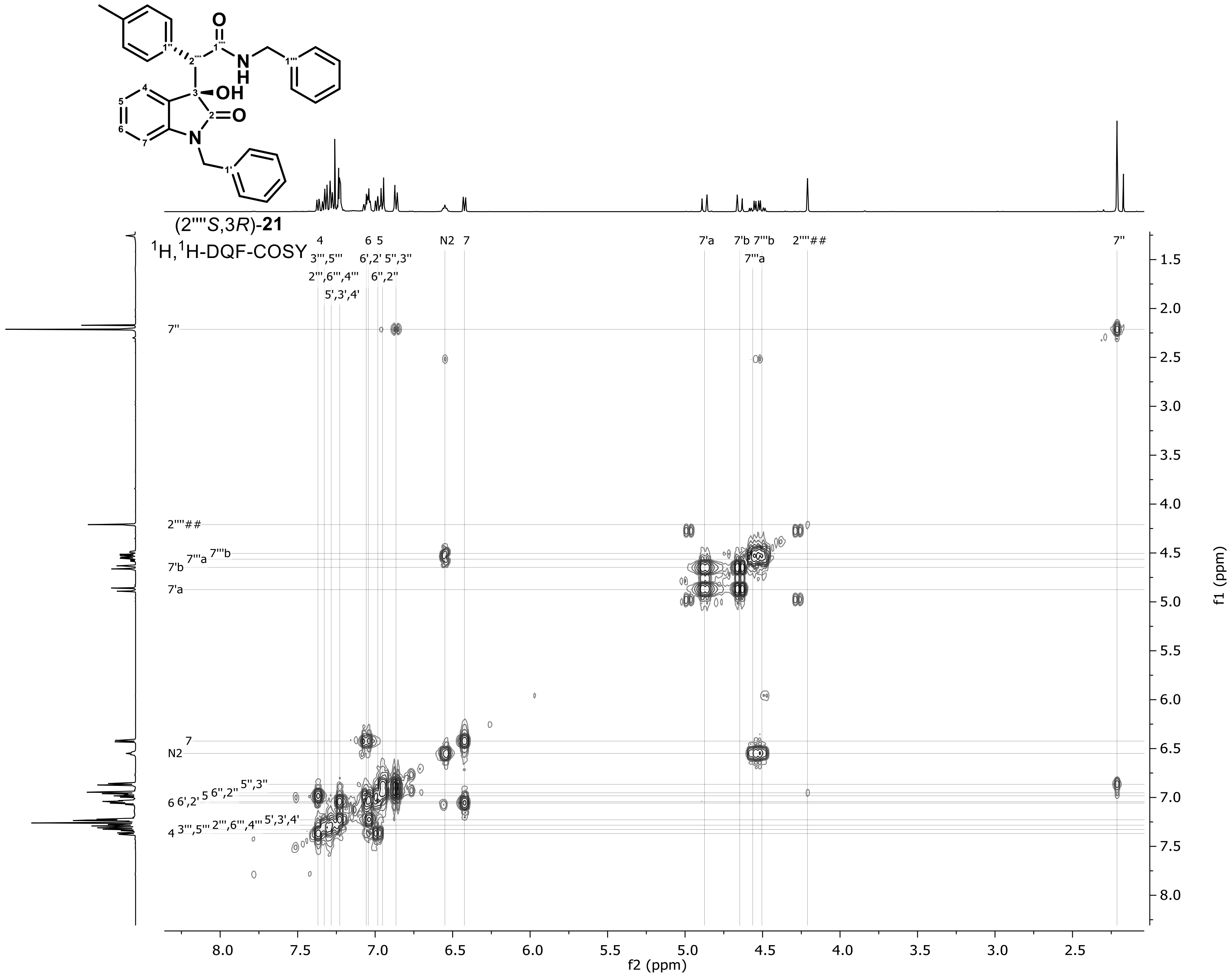

(2''''S,3R)-21
^{1}H,^{1}H-DQF-COSY
f2 (ppm)
f1 (ppm)

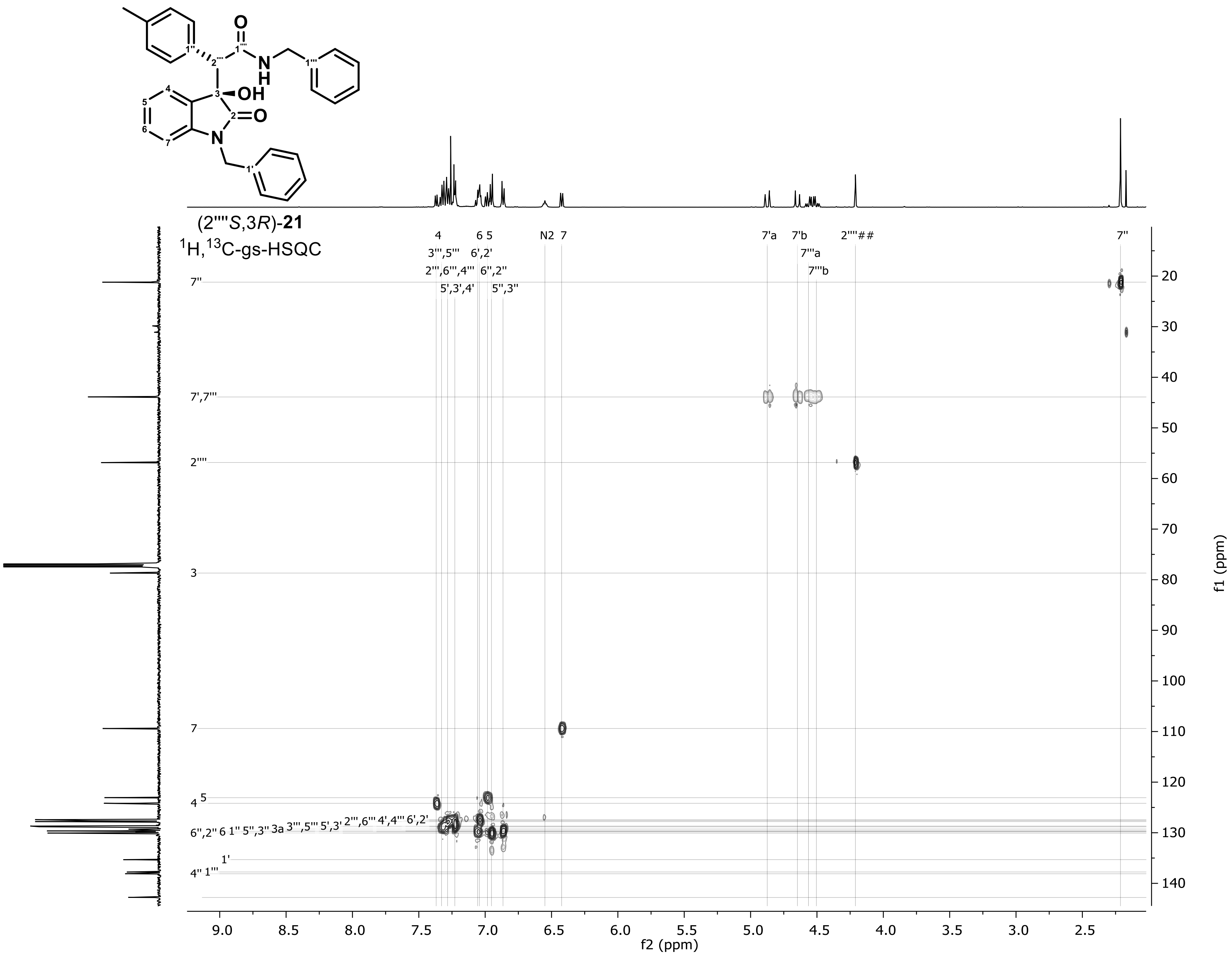
(2''''S,3R)-21
$^{1}H,^{13}C$-gs-HSQC
f2 (ppm)
f1 (ppm)

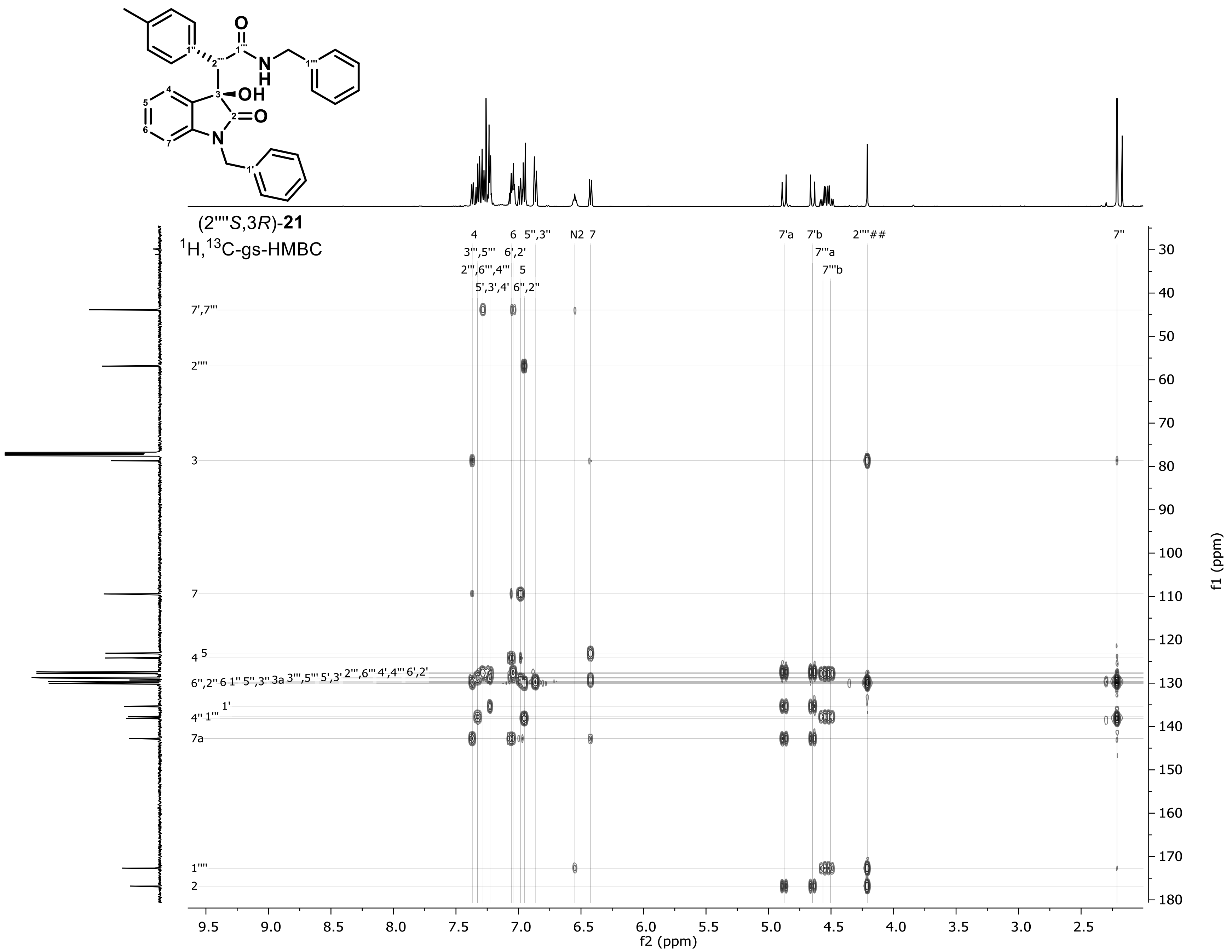
(2''''S,3R)-21
1H,13C-gs-HMBC
f2 (ppm)
f1 (ppm)

p) N,N'-Dibenzyl-2-(3-hydroxy-2-oxoindolin-3-yl)-2-*p*-chlorophenylacetamide (22)

To a 25 ml round bottomed flask was added 2-(*4*-chlorophenyl)acetic anhydride (121 mg, 0.375 mmol), *N*-benzylisatin (59.3 mg, 0.250 mmol) and (2*R*,3*S*)-HyperBTM (3.9 mg, 0.012 mmol). The mixture was cooled to 0 °C and CH_2Cl_2 (6.0 ml, 0.04 M) and Hünig's base (54.4 µL, 0.313 mmol) were added. The mixture was stirred at 0 °C for 3 h. Benzylamine (82.0 µL, 0.750 mmol) was added at 0 °C and the reaction was left to be stirred overnight at room temperature. 1,3,5-trimethoxybenzene (0.1 M soln in CH_2Cl_2, 500 µl, 0.05 mmol) was added and the solvent was removed under reduced pressure. Purification by column chromatography (20% – 30% EtOAc in Pentane) gave the title compound in two fractions (major diastereomer (57.5 mg, 46%) and minor diastereomer as white solids (7.6 mg, 6%); combined (65.1 mg, 0.13 mmol, 52%, 69:31 dr).

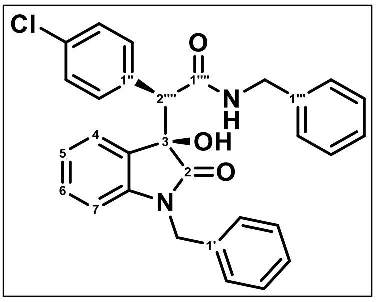

Major (2''''*R*,3*R*)-**22**: **Chiral HPLC analysis** Chiralpak IB (95:5 hexane:IPA, flow rate 2 ml·min^{-1}, 211 nm, 40 °C) t_R (2''''*R*,3*R*)-**22**: 18.5 min, t_R (2''''*S*,3*S*)-**22**: 26.1 min, 99:1; $\alpha_D^{20} = -32.4$ (*c* 1.26, $CHCl_3$); ν_{max} (thin film) 3333 (b, NH, OH), 3063 (w, CH), 3032 (w, CH), 2926 (w, CH), 1707 (s, C=O lactam),1672 (s), 1657 (s), 1612 (s, C=O amide), 1543 (m), 1537 (m), 1528 (m), 1491 (s), 1468 (s), 1454 (m), 1371 (m), 1354 (m), 1223 (w), 1175 (w), 119 (w), 1092 (w),1080 (w), 1030 (w), 1016 (w), 833 (w), 795 (w), 752 (s); **^{1}H NMR** (500 MHz, $CDCl_3$) δ_H 7.53 (1H, ddd, $^3J_{HH}$ = 7.5 Hz, $^4J_{HH}$ = 1.3 Hz, $^5J_{HH}$ = 0.4 Hz, ArC^4*H*), 7.33 – 7.24 (3H, m, PhC$^{3''',4''',5'''}$*H*), 7.24 – 7.20 (2H, m, PhC$^{2''',6'''}$*H*), 7.20 – 7.14 (4H, m, ArC^6*H*, PhC$^{3',4',5'}$*H*), 7.11 – 7.06 (2H, m, Ar(Cl)C$^{3'',5''}$*H*), 7.03 (1H, app td, $^3J_{HH}$ = 7.6 Hz, $^4J_{HH}$ = 1.0 Hz, ArC^5*H*), 6.88 – 6.83 (2H, m, Ar(Cl)C$^{2'',6''}$*H*), 6.48 (1H, s, O*H*), 6.53 – 6.59 (2H, m, PhC$^{2',6'}$*H*), 6.46 (1H, ddd, $^3J_{HH}$ = 7.9 Hz, $^4J_{HH}$ = 1.0 Hz, $^5J_{HH}$ = 0.4 Hz, ArC^7*H*), 6.12 (1H, dd, $^3J_{HH}$ = 6.0 Hz, 5.6 Hz, N*H*), 4.93 (1H, d, $^2J_{HH}$ = 15.9 Hz, NCH_aH$_b$-Ph), 4.51 (1H, dd, $^2J_{HH}$ = 14.8 Hz, $^3J_{HH}$ = 6.0 Hz, NHCH_aH$_b$-Ph), 4.48 (1H, dd, $^2J_{HH}$ = 14.8 Hz, $^3J_{HH}$ = 5.6, NHCH$_a$$H_b$-Ph), 4.34 (1H, s, C*H*-Ar(Cl)), 4.25 (1H, d, $^2J_{HH}$ = 15.9 Hz, NCH$_a$$H_b$-Ph); **^{13}C {^{1}H} NMR** (126 MHz, $CDCl_3$) δ_C 175.0 (*C*(O)NBn), 172.3 (*C*(O)NHBn), 143.2 (Ar*C*^{7a}), 137.5 (Ph*C*$^{1'''}$$CH_2$NH), 134.8$_4$ and 134.8$_1$ (Ph*C*$^{1'}$$CH_2$N and Ar(Cl)*C*$^{1''}$CH), 132.0 (Ar(Cl)*C*$^{2'',6''}$H), 131.4 (Ar*C*$^{4''}$Cl), 130.2 (Ar*C*^{6}H), 129.0 (Ph*C*$^{3''',5'''}$H), 128.9 (Ar(Cl)*C*$^{3'',5''}$H), 128.7 (Ph*C*$^{3',5'}$H), 128.3 (Ar*C*^{3a}), 127.9 (Ph*C*$^{4'''}$H), 127.8 (Ph*C*$^{2''',6'''}$H), 127.6 (Ph*C*$^{4'}$H), 126.7 (Ph*C*$^{2',6'}$H), 126.1 (Ar*C*^{4}H), 123.4 (Ar*C*^{5}H), 109.7 (Ar*C*^{7}H), 78.9 (*C*-OH), 56.4 (C*H*-Ar(Cl)), 44.0 (NH*C*H_2-Ph), 43.9 (N*C*H_2-Ph); **HRMS** (ESI$^+$) *m/z* calcd for [M(^{35}Cl)+H]$^+$ $C_{30}H_{26}ClN_2O_3$ 497.1626, found 497.1614 (−2.4 ppm).

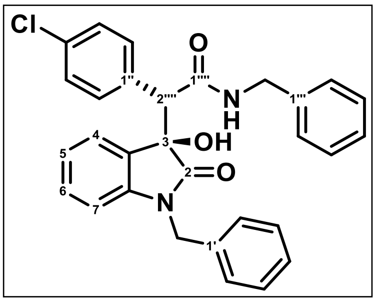

Minor (2''''*S*,3*R*)-**22**: **Chiral HPLC analysis** Chiralpak IB (95:5 hexane:IPA, flow rate 2 ml·min^{-1}, 211 nm, 40 °C) t_R (2''''*S*,3*R*)-**22**: 21.0 min, t_R (2''''*R*,3*S*)-**22**: 28.8 min, >99:1; $\alpha_D^{20} = -43.4$ (*c* 0.29, $CHCl_3$); ν_{max} (thin film) 3300 (b, NH, OH), 3086 (w, CH), 3063 (w, CH), 3030 (w, CH), 2924 (CH), 2851 (w, CH), 1701 (s, C=O lactam), 1645 (m), 1614 (s, C=O amide), 1491 (s), 1468 (m), 1454 (w), 1373 (m), 1177 (m), 1092 (m), 1080 (m), 1030 (w), 1016 (n), 827 (w), 752 (s); **^{1}H NMR** (500 MHz, $CDCl_3$) δ_H 7.37 – 7.22 (9H, m, ArC^4*H*, PhC$^{3',4',5'}$*H*, PhC$^{2''',3''',4''',5''',6'''}$*H*), 7.12 (1H, app td, $^3J_{HH}$ = 7.8 Hz, $^4J_{HH}$ = 1.3 Hz, ArC^6*H*), 7.05 – 6.95 (7H, m, ArC^5*H*, Ph*C*$^{2',6'}$*H*, Ar(Cl)C$^{2'',3'',5'',6''}$*H*), 6.93 (1H, app t, $^3J_{HH}$ = 5.8 Hz, N*H*), 6.48 (1H, ddd, $^3J_{HH}$ 7.9 Hz, $^4J_{HH}$ = 1.0 Hz, $^5J_{HH}$ = 0.4 Hz, ArC(4)*H*), 6.41 (1H, s, O*H*), 4.89 (1H, d, $^2J_{HH}$ = 15.8 Hz, NCH_aH$_b$-Ph), 4.58 (1H, d, $^2J_{HH}$ = 15.8 Hz, NCH_aH$_b$-Ph), 4.56 (1H, dd, $^2J_{HH}$ = 15.0 Hz, $^3J_{HH}$ = 5.9 Hz, NHCH_aH$_b$-Ph), 4.51 (1H, dd, $^2J_{HH}$ = 15.0 Hz, $^3J_{HH}$ 5.7 Hz, NHCH$_a$$H_b$-Ph), 4.23 (1H, s, C*H*-Ar(Cl)); **^{13}C {^{1}H} NMR** (127 MHz, $CDCl_3$) δ_C 176.5 (*C*(O)NBn), 171.5 (*C*(O)NHBn), 142.6 (Ar*C*^{7a}), 137.7 (Ph*C*$^{1'''}$$CH_2$NH), 129.0 (Ar*C*^{3a} and Ar(Cl)*C*$^{3'',5''}$H), 135.0 (Ph*C*$^{1'}$$CH_2$N), 134.4 (Ar*C*$^{4''}$Cl), 131.5 (Ar(Cl)*C*$^{1''}$CH), 131.4 (Ar(Cl)*C*$^{2'',6''}$H), 130.2 (Ar*C*^{6}H), 128.9 and 128.8 (Ph*C*$^{3',5'}$H, Ph*C*$^{3''',5'''}$H), 127.9 (Ph*C*$^{2''',6'''}$H), 127.8 and 127.7 (Ph*C*$^{4'}$H and Ph*C*$^{4'''}$H), 127.3 (Ph*C*$^{2',6'}$H), 124.2 (Ar*C*^{4}H), 123.4 (Ar*C*^{5}H), 109.6 (Ar*C*^{7}H), 78.4 (*C*-OH), 57.5 (*C*H-Ar(Cl)), 43.9$_9$ and 43.9$_7$ (N*C*H_2-Ph and NH*C*H_2-Ph); **HRMS** (ESI$^+$) *m/z* calcd for [M(^{35}Cl)+H]$^+$ $C_{30}H_{26}ClN_2O_3$ 497.1626, found 497.1616 (−2.1 ppm).

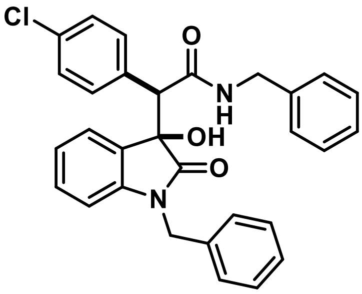

(±)-anti-**22**

PDA Ch1 211nm

Peak#	Ret. Time	Area%
1	18.966	50.063
2	25.718	49.937
Total		100.000

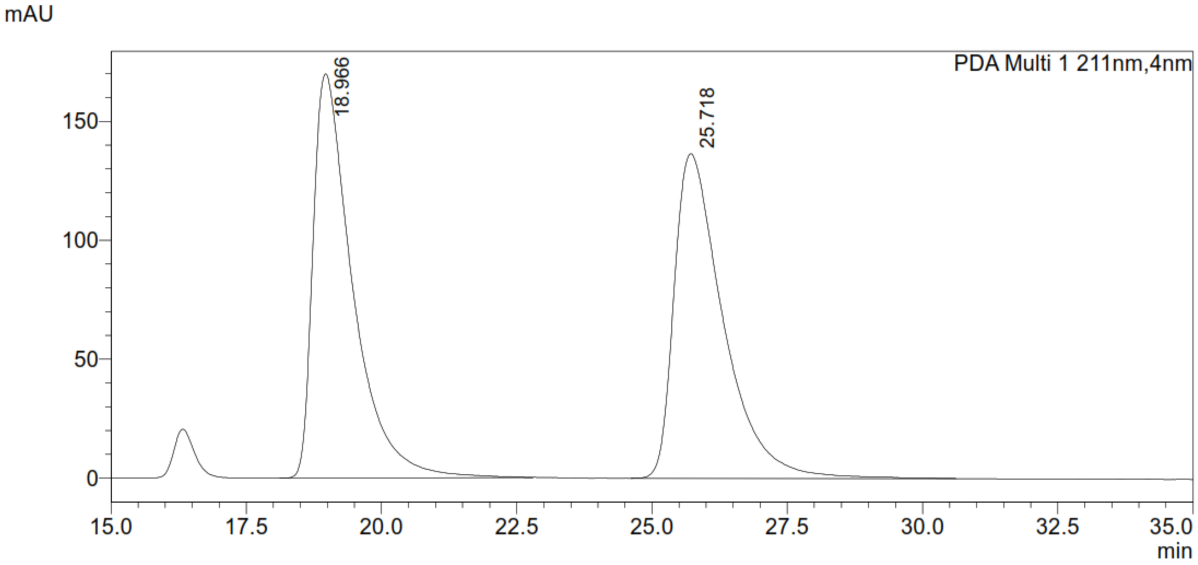

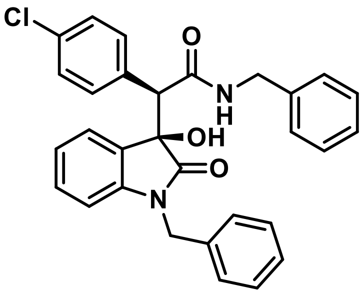

(–)-(2''''*R*,3*R*)-**22**

PDA Ch1 211nm

Peak#	Ret. Time	Area%
1	18.468	99.186
2	26.105	0.814
Total		100.000

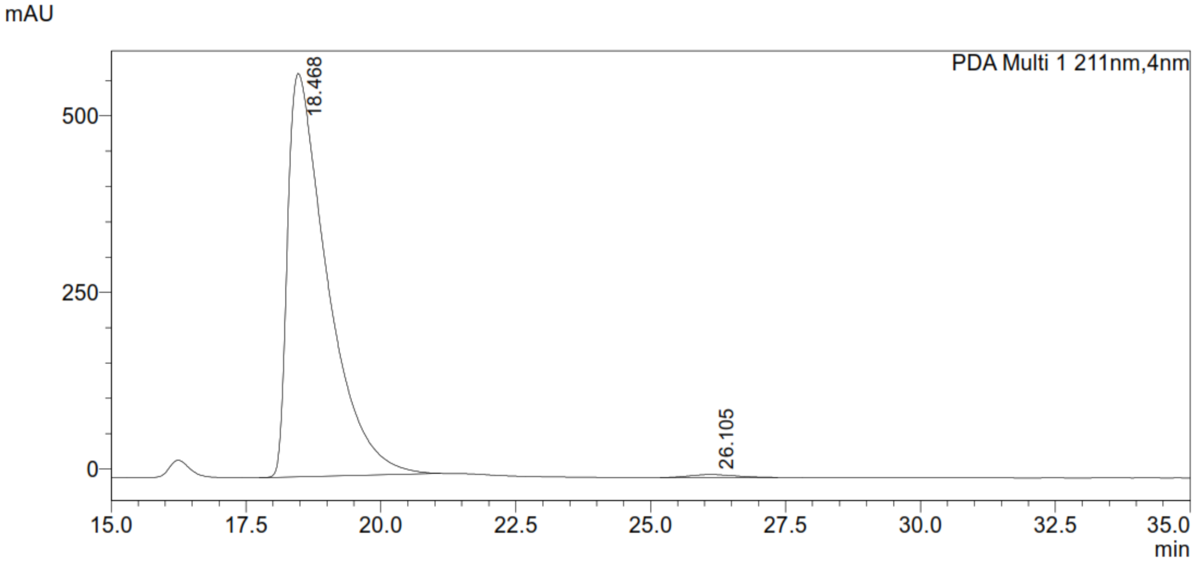

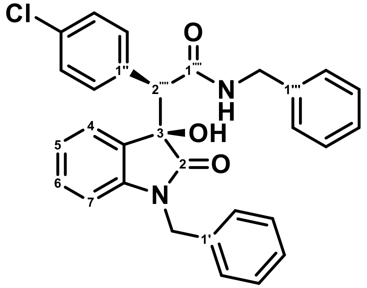

(2''''*R*,3*R*)-**22**
^{1}H NMR (500 MHz, $CDCl_3$)

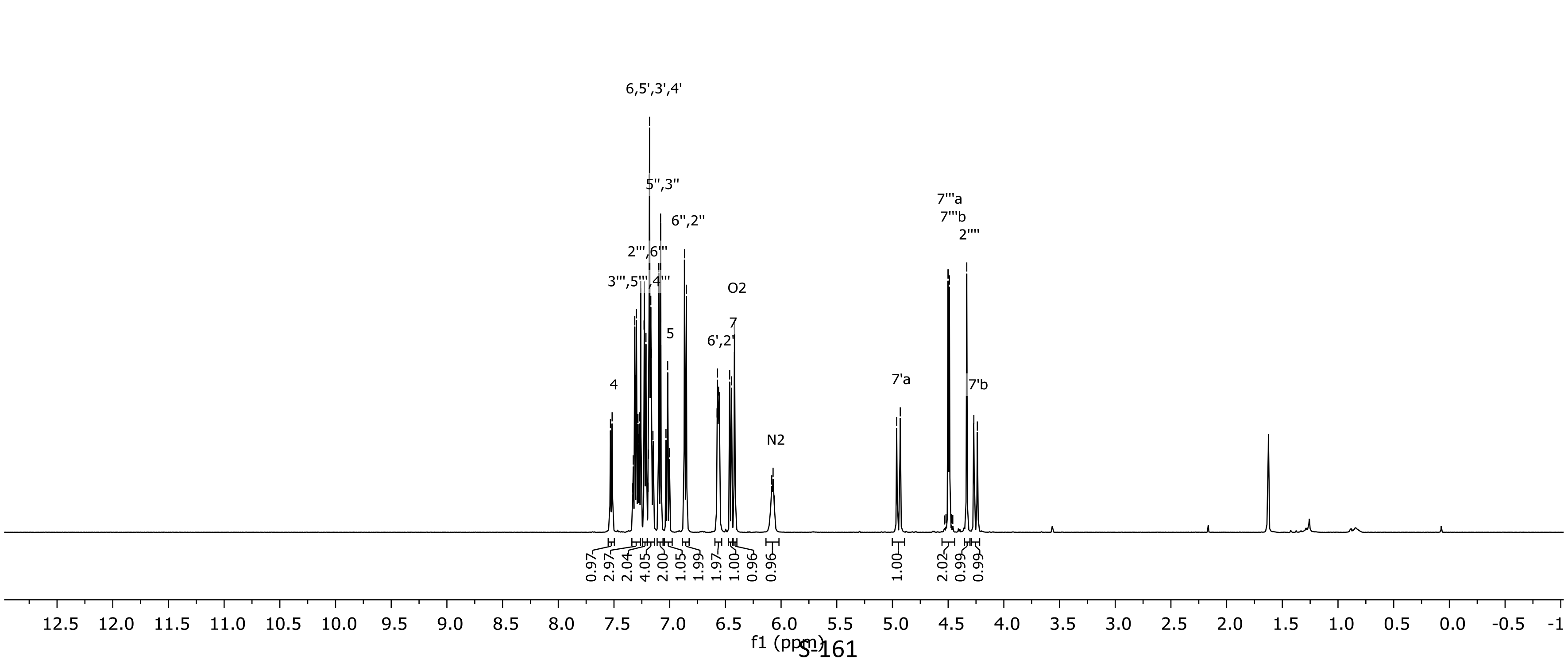

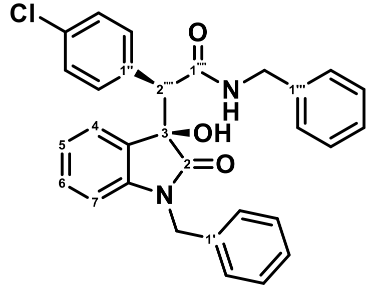

(2''''R,3R)-**22**

$^{13}C\ \{^{1}H\}$ NMR (126 MHz, $CDCl_3$)

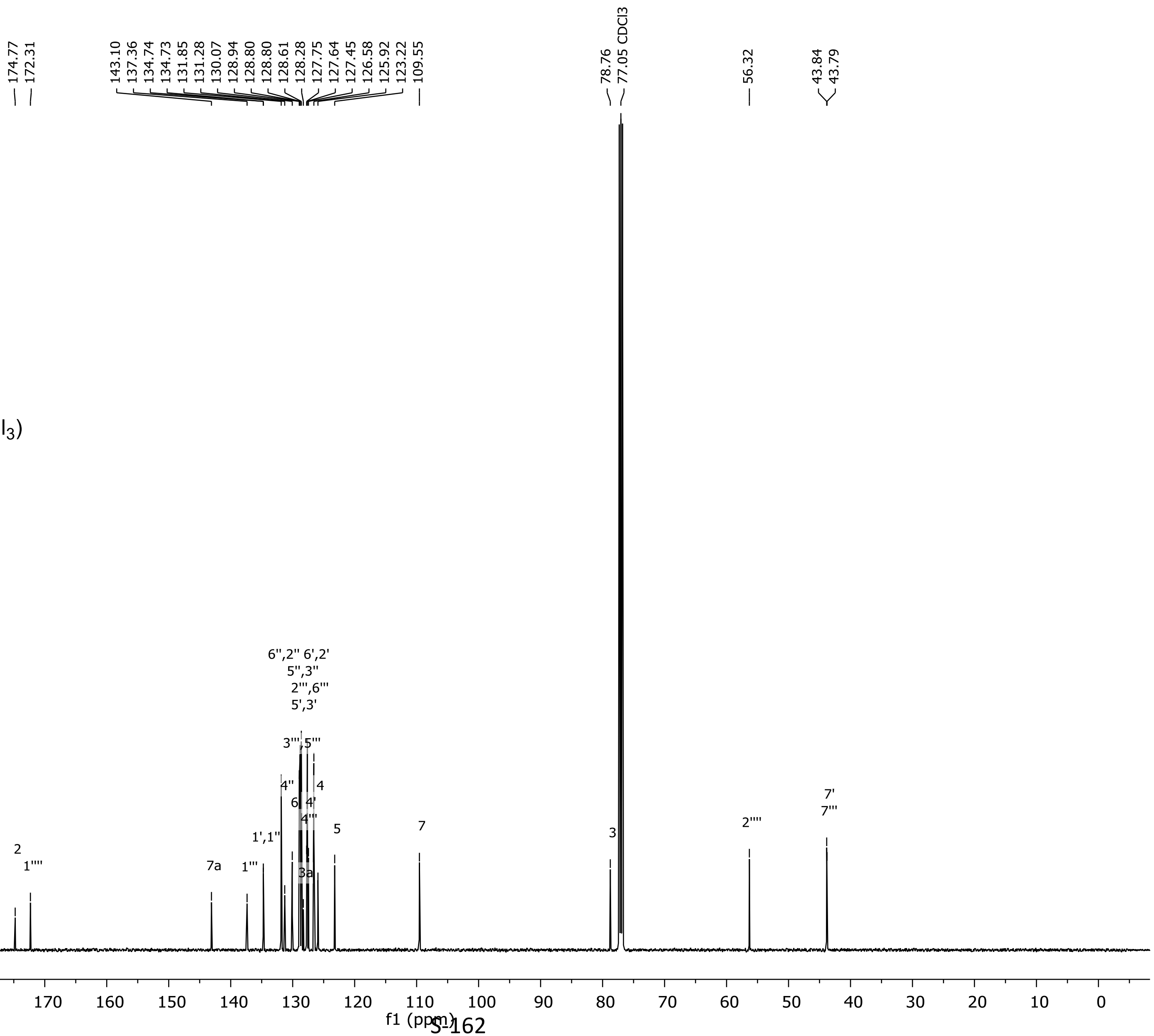

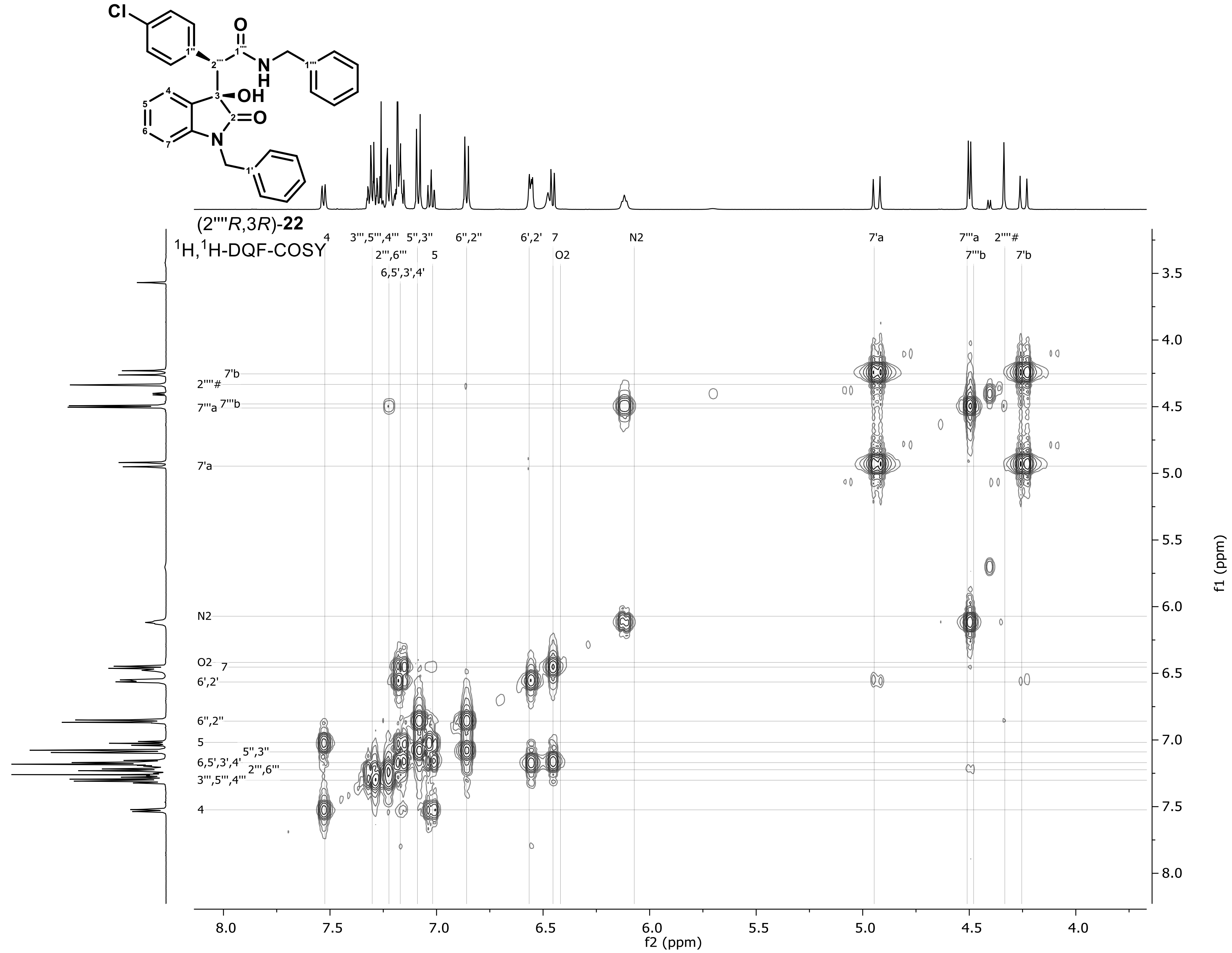
(2''''R,3R)-22
^{1}H,^{1}H-DQF-COSY
f2 (ppm)
f1 (ppm)

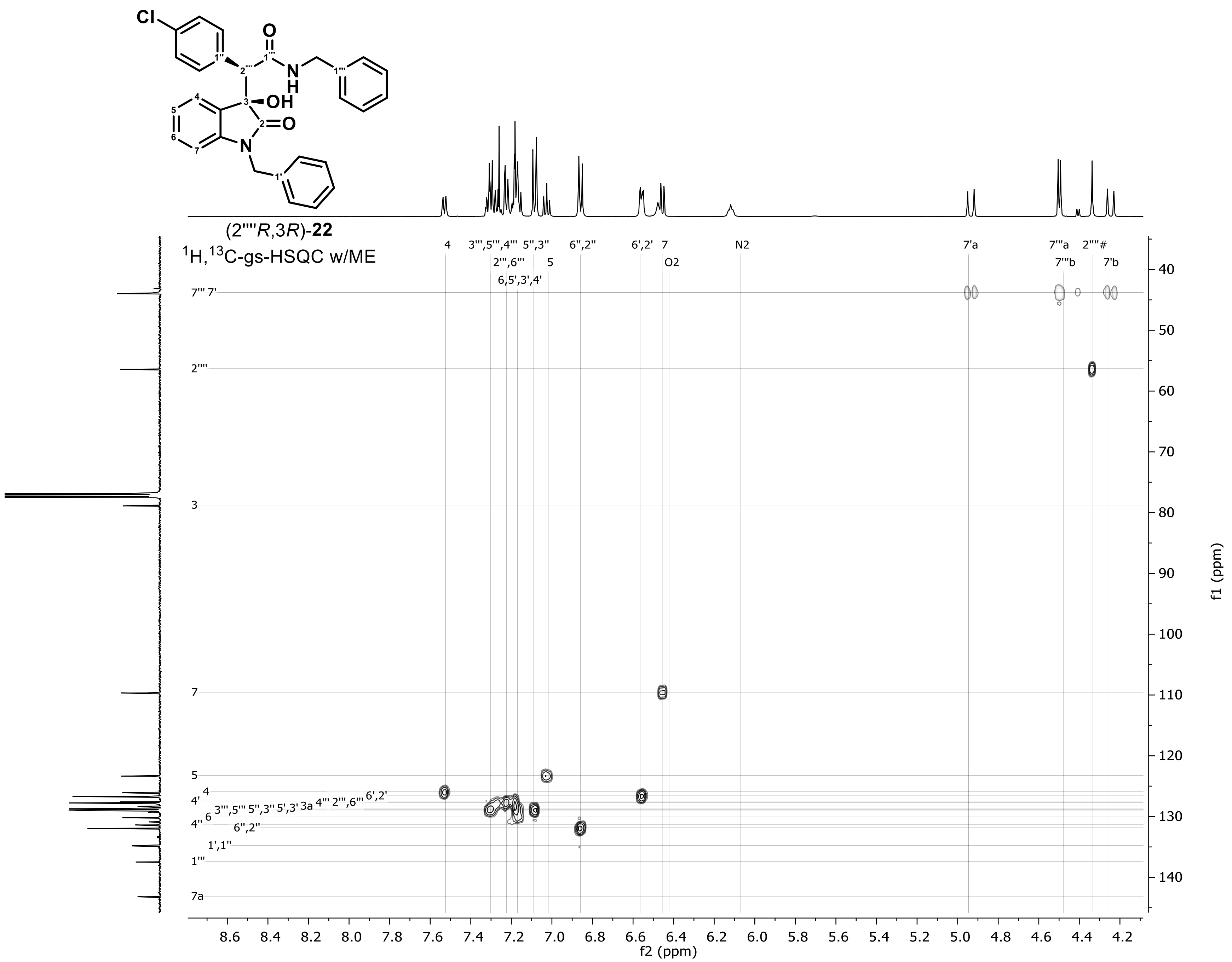

Cl
O
1''''
2''''
1''
NH
OH
3
2
O
N
1'
1'''
4
5
6
7
(2''''R,3R)-22
^{1}H,^{13}C-gs-HSQC w/ME
f2 (ppm)
f1 (ppm)

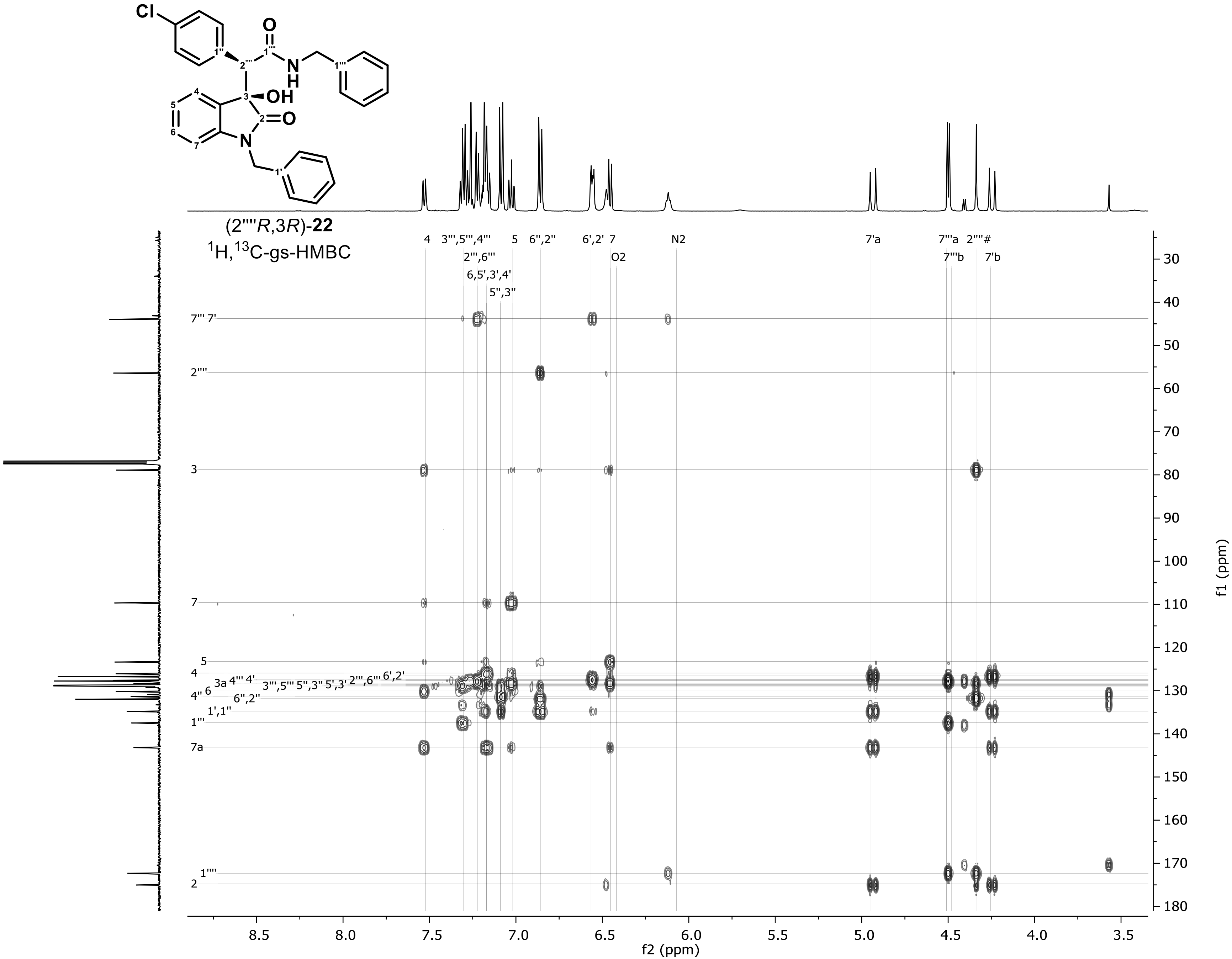

(2''''R,3R)-**22**

$^{1}H,^{13}C$-gs-HMBC

(±)-syn-**22**

PDA Ch1 211nm

Peak#	Ret. Time	Area%
1	21.273	50.247
2	28.067	49.753
Total		100.000

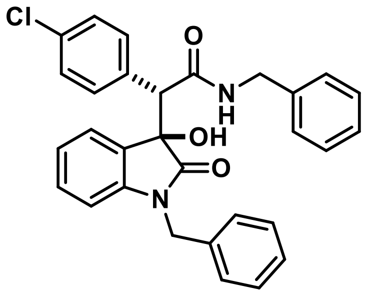

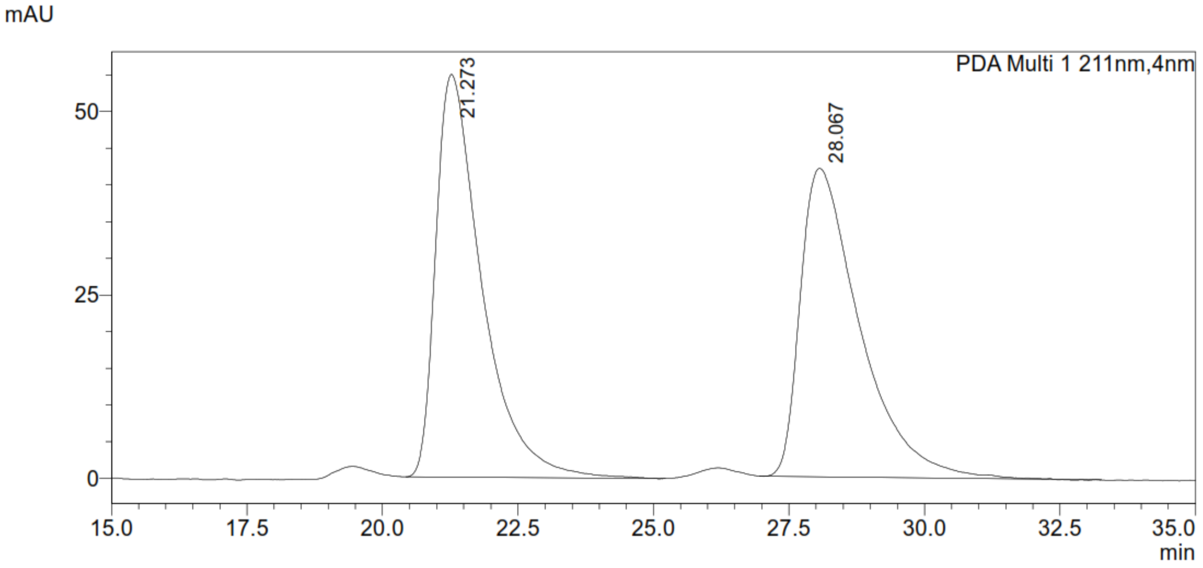

(–)-(2''''*S*,3*R*)-**22**

PDA Ch1 211nm

Peak#	Ret. Time	Area%
1	20.981	99.467
2	28.835	0.533
Total		100.000

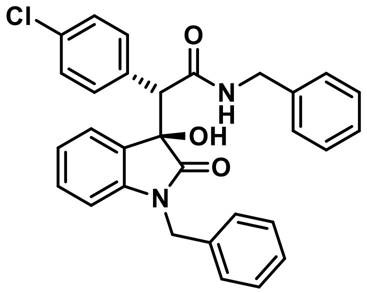

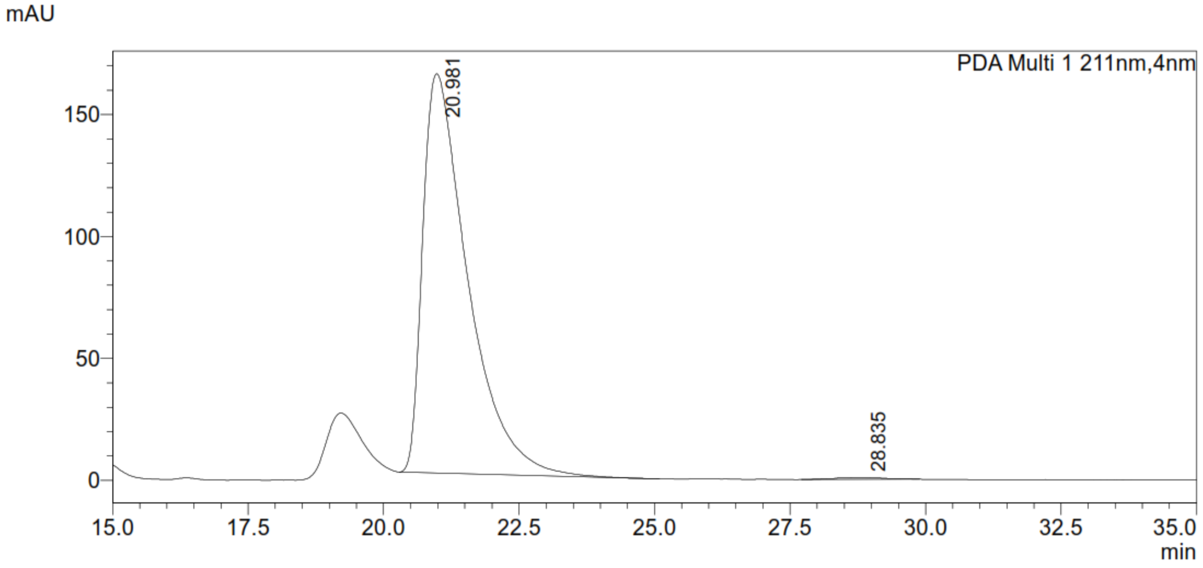

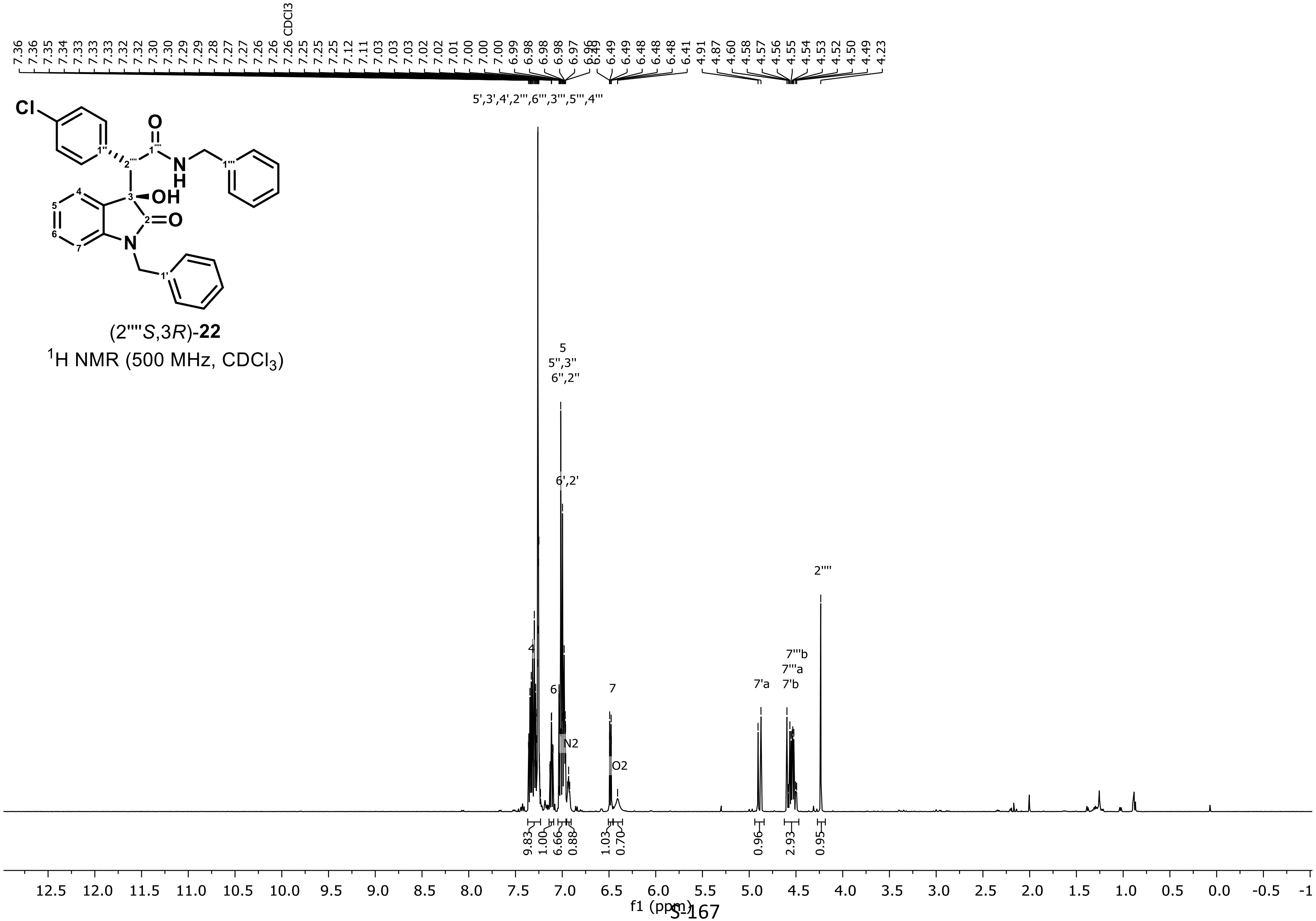

(2''''*S*,3*R*)-**22**

^{1}H NMR (500 MHz, $CDCl_3$)

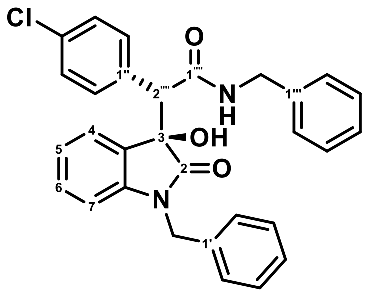

(2''''*S*,3*R*)-**22**

$^{13}C\ \{^{1}H\}$ NMR (127 MHz, $CDCl_3$)

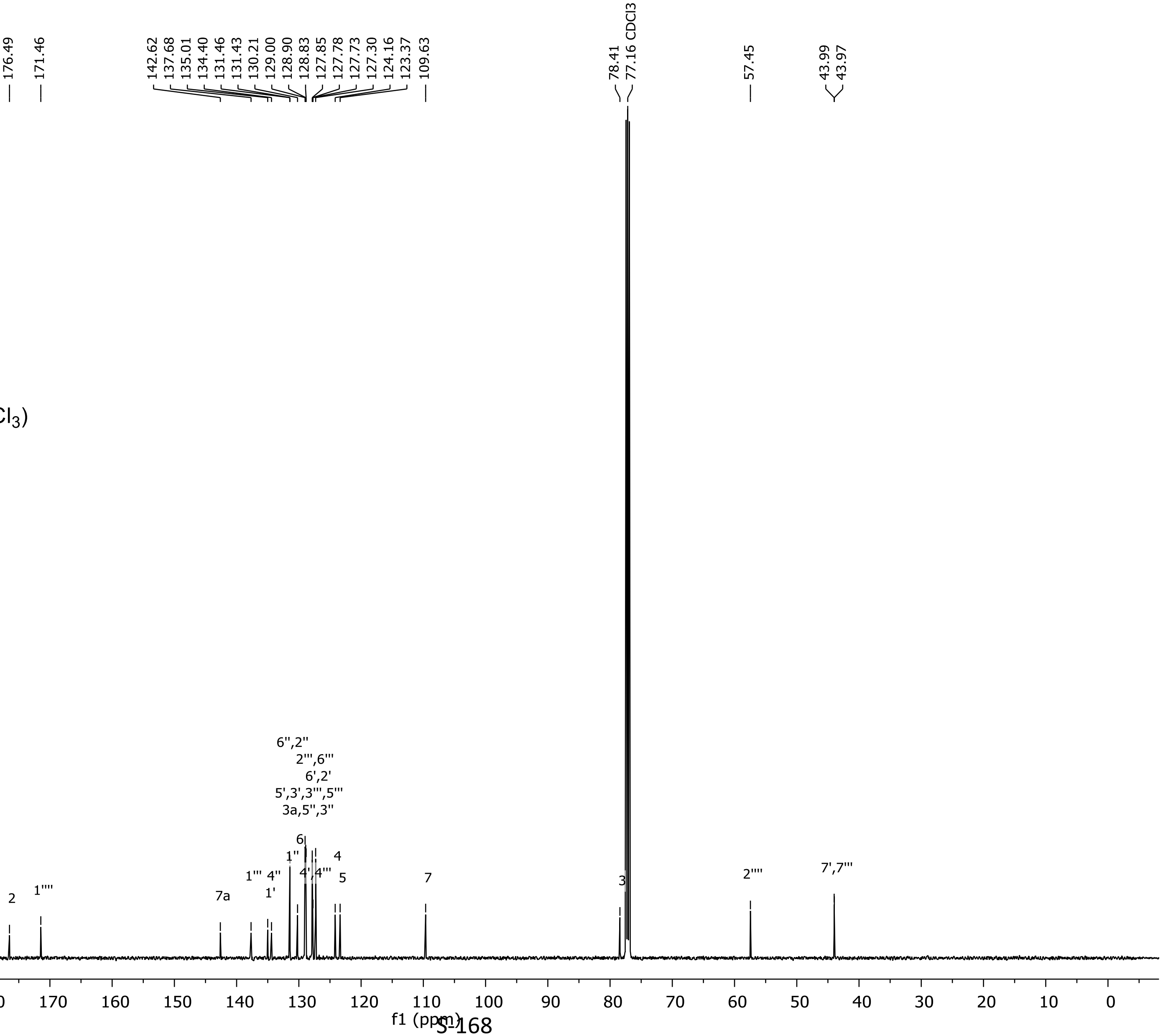

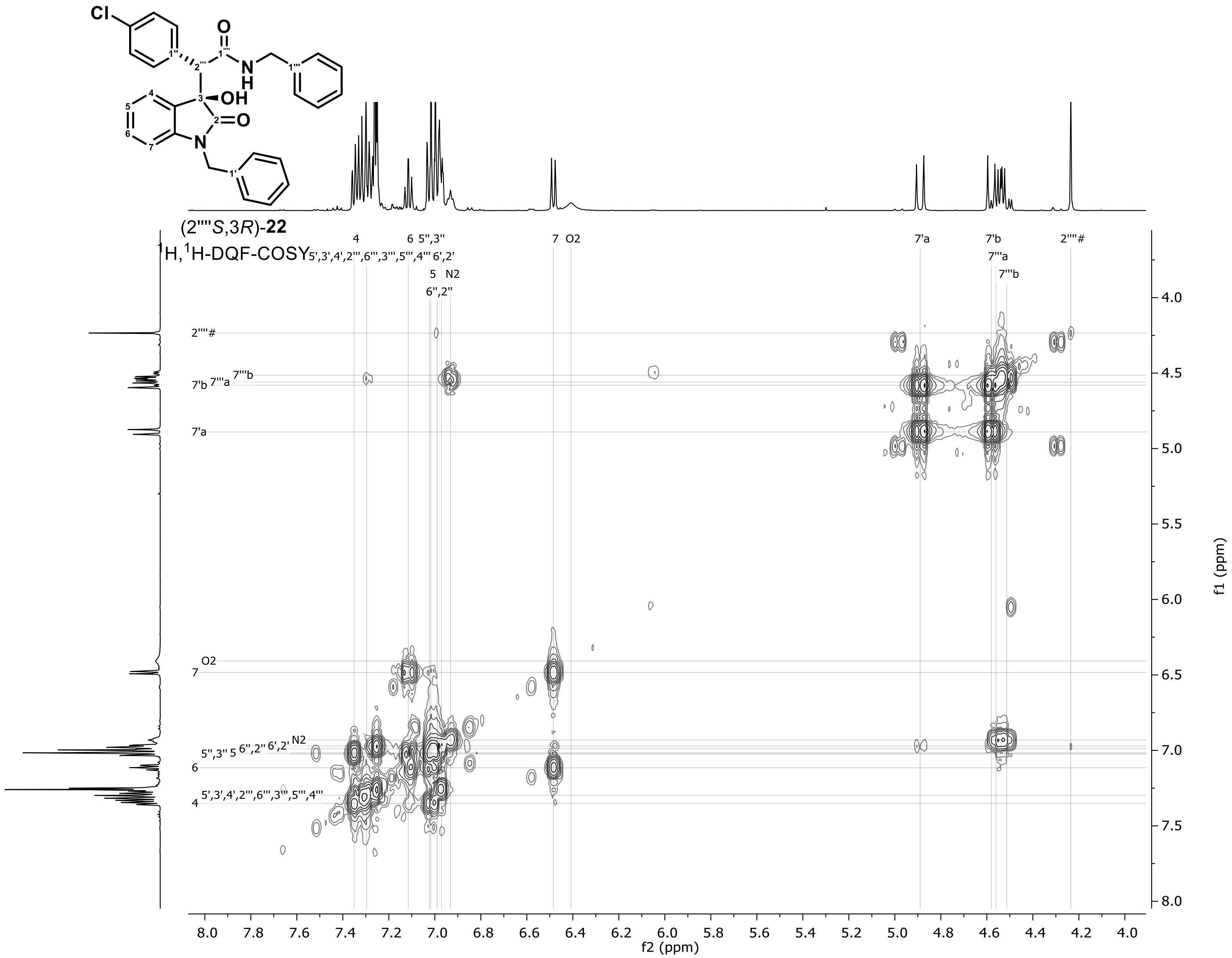
(2''''S,3R)-22
1H,1H-DQF-COSY
f2 (ppm)
f1 (ppm)

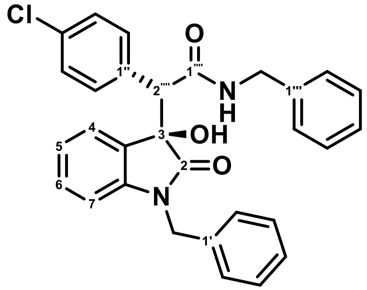

(2''''*S*,3*R*)-**22**

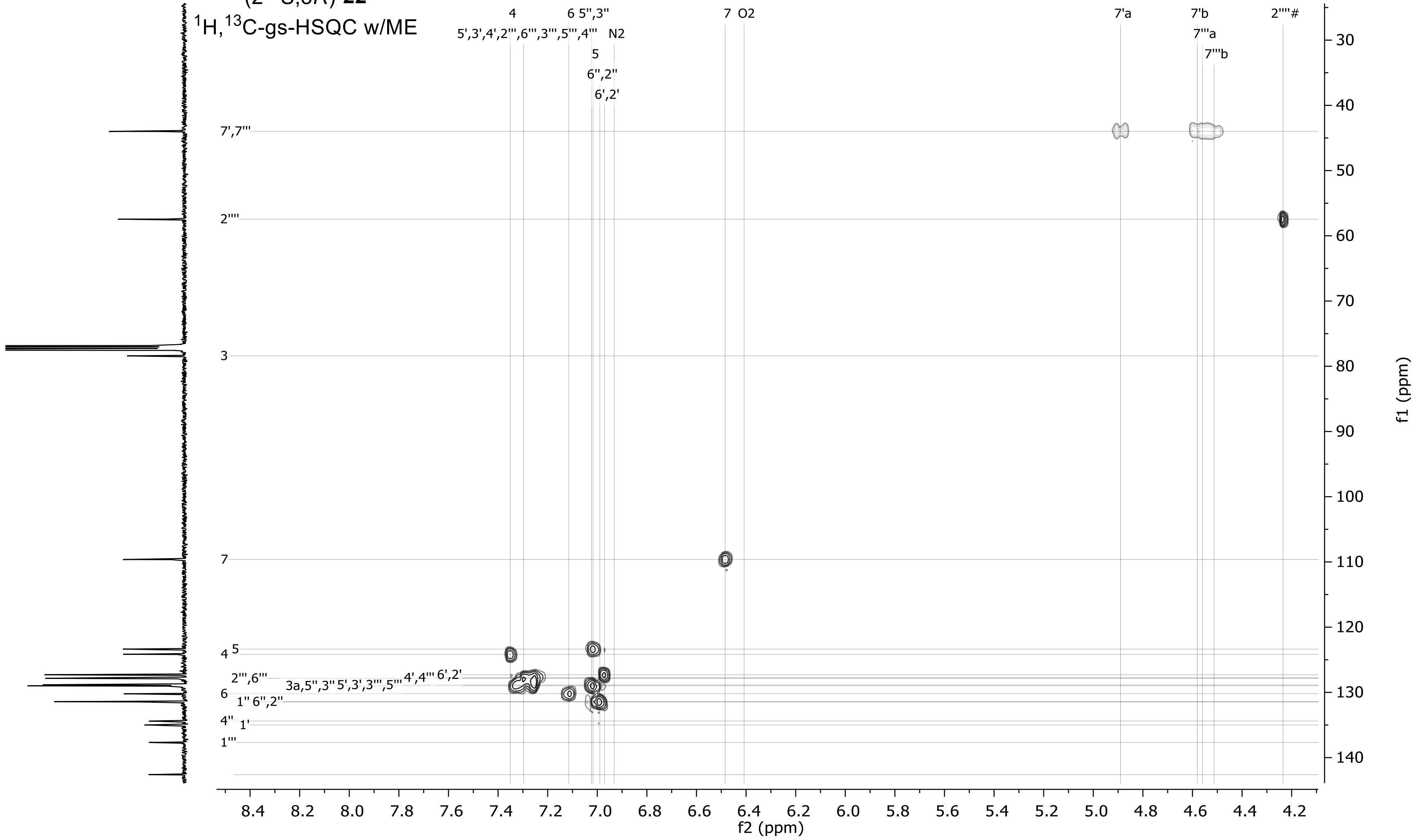

1H,13C-gs-HSQC w/ME
4
6 5'',3''
5',3',4',2''',6''',3''',5''',4'''
N2
5
6'',2''
6',2'
7 O2
7'a
7'b
7'''a
7'''b
2''''#
7',7'''
2''''
3
7
4 5
2''',6'''
3a,5'',3'' 5',3',3''',5''' 4',4''' 6',2'
6
1'' 6'',2''
4''
1'
1'''
f2 (ppm)
f1 (ppm)

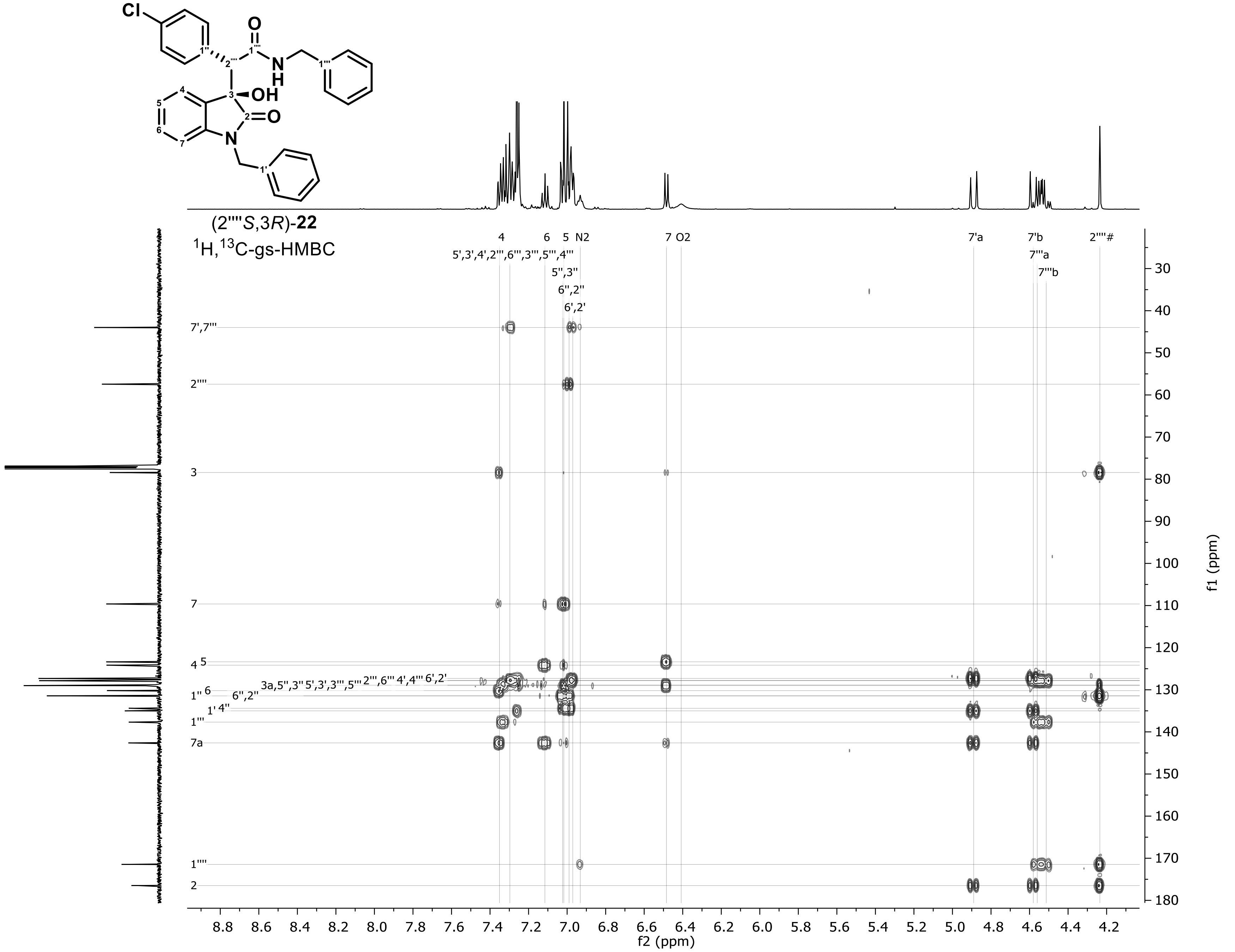

(2''''S,3R)-22
^{1}H,^{13}C-gs-HMBC
f2 (ppm)
f1 (ppm)

q) N,N'-Dibenzyl-2-(3-hydroxy-2-oxoindolin-3-yl)-2-*p*-bromophenylacetamide (23)

To a 25 ml round bottomed flask was added 2-(4-bromophenyl)acetic anhydride (155 mg, 0.375 mmol), *N*-benzylisatin (59.3 mg, 0.250 mmol) and (2*R*,3*S*)-HyperBTM (3.9 mg, 0.012 mmol). The mixture was cooled to 0 °C and CH_2Cl_2 (6.0 ml, 0.04 M) and Hünig's base (54.4 µL, 0.313 mmol) were added. The mixture was stirred at 0 °C for 3 h. Benzylamine (82.0 µL, 0.750 mmol) was added at 0 °C and the reaction was left to be stirred overnight at room temperature. 1,3,5-trimethoxybenzene (0.1 M soln in CH_2Cl_2, 500 µl, 0.05 mmol) was added and the solvent was removed under reduced pressure. Purification by column chromatography (15% – 35% EtOAc in Pentane) gave the title compound in two fractions (major diastereomer (45 mg, 33%) and minor diastereomer as white solids (23.8 mg, 18%); combined (68.9 mg, 0.127 mmol, 51%, 80:20 dr).

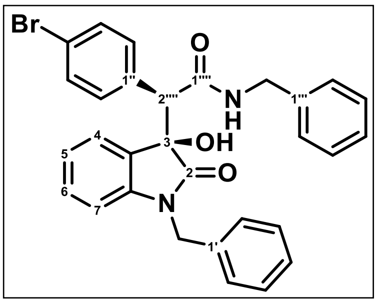

Major (2''''*R*,3*R*)-**23**: **Chiral HPLC analysis** Chiralpak IB (95:5 hexane:IPA, flow rate 2 ml·min^{-1}, 211 nm, 40 °C) t_R (2''''*R*,3*R*)-**23**: 20.4 min, t_R (2''''*S*,3*S*)-**23**: 28.0 min, >99:1 er; α_D^{20} = −26.0 (*c* 0.79, $CHCl_3$); ν_{max} (thin film) 3335 (O–H), 3061 (N–H), 3030 (N–H), 1707 (C=O lactam), 1612 (C=O amide), 1487 (CH2), 1175 (C–O alcohol); **^{1}H NMR** (500 MHz, $CDCl_3$) δ_H 7.52 (1H, ddd, $^3J_{HH}$ 7.5 Hz, $^4J_{HH}$ = 1.4 Hz, $^5J_{HH}$ = 0.5 Hz, ArC^4*H*), 7.34 – 7.18 (10H, m, PhC$^{2',4',6'}$*H*, Ar(Br)C$^{3'',5''}$*H*, PhC$^{2''',3''',4''',5''',6'''}$*H*), 7.16 (1H, app td, $^3J_{HH}$ = 7.8 Hz, $^4J_{HH}$ = 1.4 Hz, ArC^6*H*), 7.02 (1H, app td, $^3J_{HH}$ = 7.8 Hz, 7.6 Hz, $^4J_{HH}$ = 1.0 Hz, ArC^5*H*), 6.83 – 6.77 (2H, m, Ar(Br)C$^{2'',6''}$*H*), 6.60 – 6.54 (2H, m, PhC$^{2',6'}$*H*), 6.45 (1H, ddd, $^3J_{HH}$ = 7.8 Hz, $^4J_{HH}$ = 1.0 Hz, $^5J_{HH}$ = 0.5 Hz, ArC^7*H*), 6.42 (1H, s, O*H*), 6.12 (1H, app t, $^3J_{HH}$ = 5.7 Hz, N*H*), 4.95 (1H, d, $^2J_{HH}$ = 15.9 Hz, NCH_aH$_b$-Ph), 4.51 (1H, dd, $^2J_{HH}$ = 14.9 Hz, $^3J_{HH}$ = 5.7 Hz, NHCH_aH$_b$-Ph), 4.47 (1H, dd, $^2J_{HH}$ = 14.9 Hz, $^3J_{HH}$ = 5.9 Hz, NHCH$_a$$H_b$-Ph), 4.32 (1H, s, C*H*-Ar(Br)), 4.25 (1H, d, $^2J_{HH}$ = 15.9 Hz, NCH$_a$$H_b$-Ph); **^{13}C {^{1}H} NMR** (127 MHz, $CDCl_3$) δ_C 174.9 (*C*(O)NBn), 172.3 (*C*(O)NHBn), 143.2 (Ar*C*^{7a}), 137.5 (Ph*C*$^{1'''}$), 134.9 (Ph*C*$^{1'}$), 132.3 (Ar(Br)*C*$^{2'',6''}$H), 132.0 (Ar(Br)*C*$^{3'',5''}$H), 131.9 (Ar(Br)*C*$^{1''}$), 128.9 and 128.8 (Ph*C*$^{3',5'}$H), 130.2 (Ph*C*$^{3''',5'''}$H), 128.4 (Ar*C*^{3a}), 127.9 and 127.6 (Ph*C*$^{4'}$H and Ph*C*$^{4'''}$H), 127.8 (Ph*C*$^{2''',6'''}$H), 126.7 (Ph*C*$^{2',6'}$H), 126.1 (Ar*C*^{4}H), 123.3 (Ar*C*^{5}H), 123.1 (Ar*C*$^{4''}$Br), 109.7 (Ar*C*^{7}H), 78.8 (*C*-OH), 56.5 (*C*H-Ar(Br)), 44.0 and 43.9 (N*C*H$_2$-Ph and NH*C*H$_2$-Ph); **HRMS** (ESI$^+$) *m/z* calcd for $[M(^{79}Br)+H]^+$ $C_{30}H_{26}BrN_2O_3$ 541.1121, found 541.1121 (−0.0 ppm).

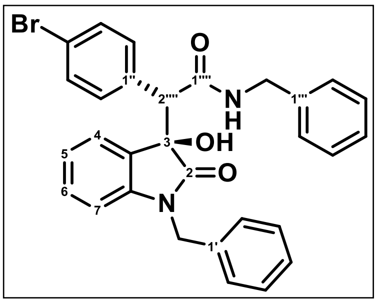

Minor (2''''*S*,3*R*)-**23**: **Chiral HPLC analysis** Chiralpak IB (95:5 hexane:IPA, flow rate 2 mL·min^{-1}, 211 nm, 40 °C) t_R (2''''*S*,3*R*)-**23**: 23.0 min, t_R (2''''*R*,3*S*)-**23**: 31.2 min, 99:1 er; α_D^{20} = −78.3 (*c* 0.23, $CHCl_3$); ν_{max} (thin film) 3292 (m, broad, OH, NH), 3088 (w), 3063 (w), 2955 (w), 2924 (w), 2855 (w), 1701 (s, C=O, lactam), 1641 (s, C=O, amide), 1614 (s), 1489 (s), 1468 (m), 1454 (m), 1435 (w), 1373 (m), 1300 (w), 1177 (m), 1074 (m), 1030 (w), 1013 (m), 935 (w), 824 (w), 787 (w), 752 (m); **^{1}H NMR** (500 MHz, $CDCl_3$) δ_H 7.38 – 7.24 (9H, m, ArC^4*H*, PhC$^{3',4',5'}$*H*, PhC$^{2''',3''',4''',5''',6'''}$*H*), 7.12 (1H, app td, $^3J_{HH}$ = 7.8 Hz, $^4J_{HH}$ = 1.3 Hz, ArC^6*H*), 7.20 – 7.15 (2H, m, Ar(Br)C$^{3'',5''}$*H*), 7.02 (1H, app td, $^3J_{HH}$ = 7.6 Hz, $^4J_{HH}$ = 1.0 Hz, ArC^5*H*), 7.00 – 6.96 (2H, m, PhC$^{2',6'}$*H*), 6.95 – 6.90 (2H, m, Ar(Br)C$^{2'',6''}$*H*), 6.82 (1H, dd, $^3J_{HH}$ = 5.8 Hz, 5.4 Hz, N*H*), 6.49 (1H, d, $^3J_{HH}$ = 7.8 Hz, ArC^7*H*), 6.38 (1H, s, O*H*), 4.90 (1H, d, $^2J_{HH}$ = 15.8 Hz, NCH_aH$_b$-Ph), 4.58 (1H, d, $^2J_{HH}$ 15.8 Hz, NCH$_a$$H_b$-Ph), 4.55 (1H, dd, $^2J_{HH}$ = 15.3 Hz, $^3J_{HH}$ 5.4 Hz, NHCH_aH$_b$-Ph), 4.51 (1H, dd, $^2J_{HH}$ = 15.3 Hz, $^3J_{HH}$ = 5.8 Hz, NHCH$_a$$H_b$-Ph), 4.20 (1H, s, C*H*-Ar); **^{13}C {^{1}H} NMR** (126 MHz, $CDCl_3$) δ_C 176.4 (*C*(O)NBn), 171.4 (*C*(O)NHBn), 142.7 (Ar*C*^{7a}), 137.7 (Ph*C*$^{1'''}$CH$_2$NH), 135.1 (Ph*C*$^{1'}$CH$_2$N), 131.9$_9$ (Ar(Br)*C*$^{1''}$CH), 131.9$_8$ (Ar(Br)*C*$^{3'',5''}$H), 131.8 (CH-Ar(Br)*C*$^{2'',6''}$H), 130.2 (Ar*C*^{6}H), 129.0 (Ar*C*^{3a}), 128.8$_7$ and 128.9$_1$ (Ph*C*$^{3',5'}$H and Ph*C*$^{3''',5'''}$H), 127.9 (Ph*C*$^{2''',6'''}$H), 127.7$_6$ and 127.8$_0$ (Ph*C*$^{4'}$H and Ph*C*$^{4'''}$H), 127.3 (Ph*C*$^{2',6'}$H), 124.2 (Ar*C*^{4}H), 123.4 (Ar*C*^{5}H), 122.7 (Ar*C*$^{4''}$Br), 109.7 (Ar*C*^{7}H), 78.4 (*C*-OH), 57.5 (*C*H-Ar(Br)), 44.0 (N*C*H$_2$-Ph and NH*C*H$_2$-Ph); **HRMS** (ESI$^+$) *m/z* calcd for $[M+H]^+$ $C_{30}H_{26}BrN_2O_3$ 541.1121, found 541.1120 (−0.2 ppm).

(±)-anti-**23**

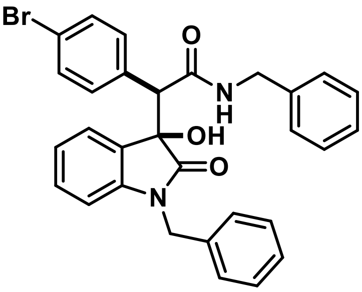

PDA Ch1 211nm

Peak#	Ret. Time	Area%
1	20.681	50.401
2	27.581	49.599
Total		100.000

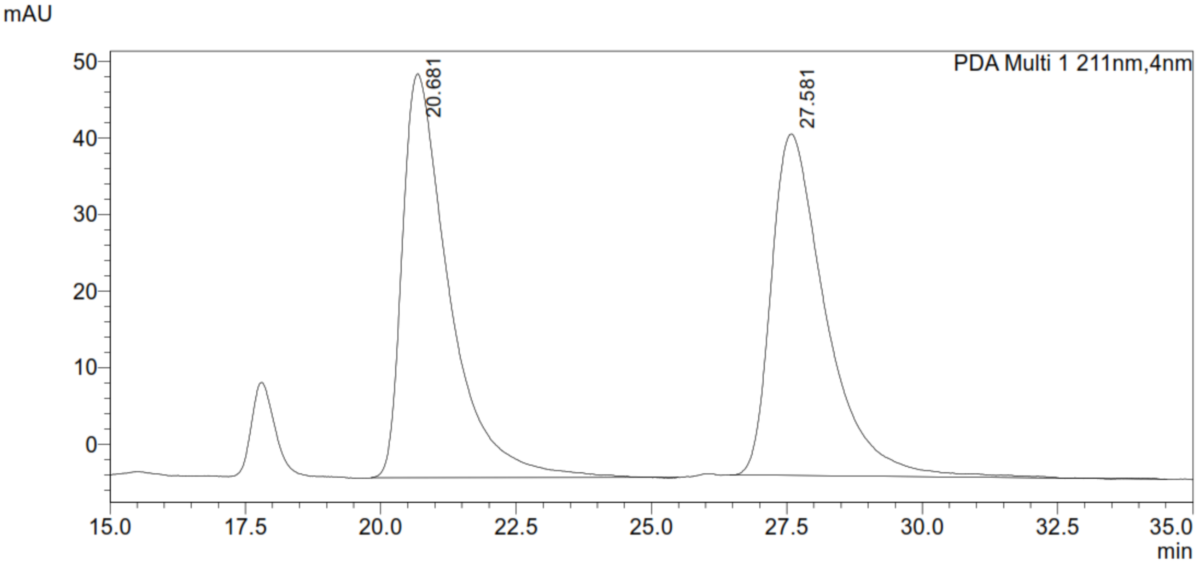

(–)-(2''''*R*,3*R*)-**23**

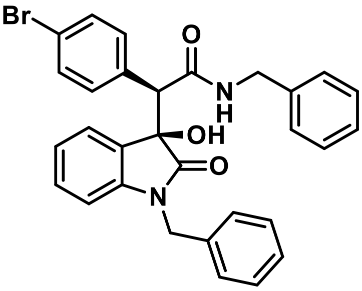

PDA Ch1 211nm

Peak#	Ret. Time	Area%
1	20.421	99.378
2	28.001	0.622
Total		100.000

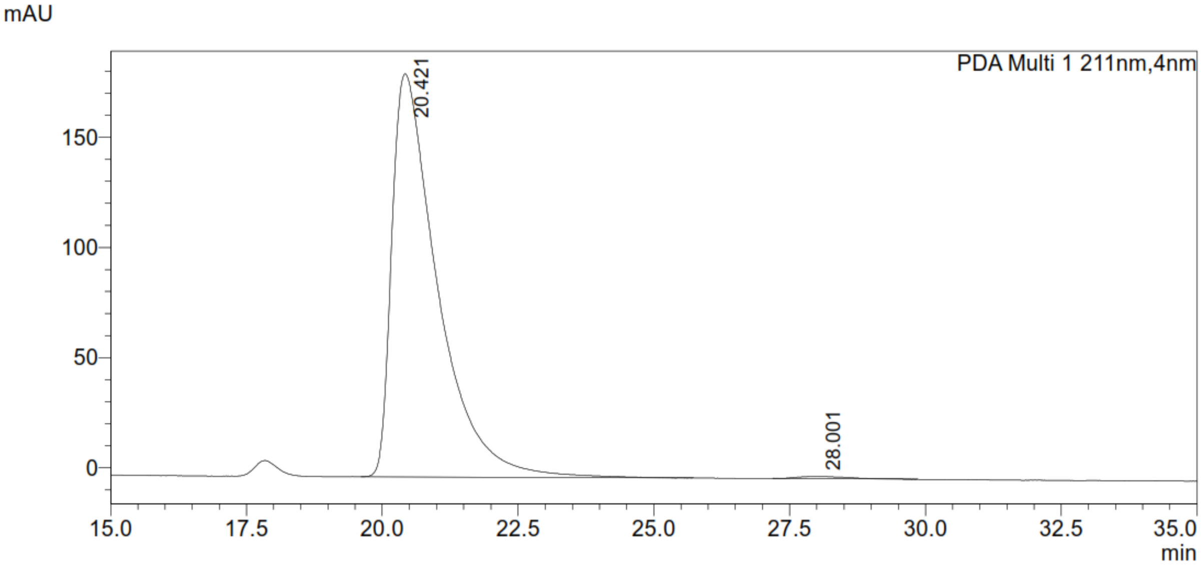

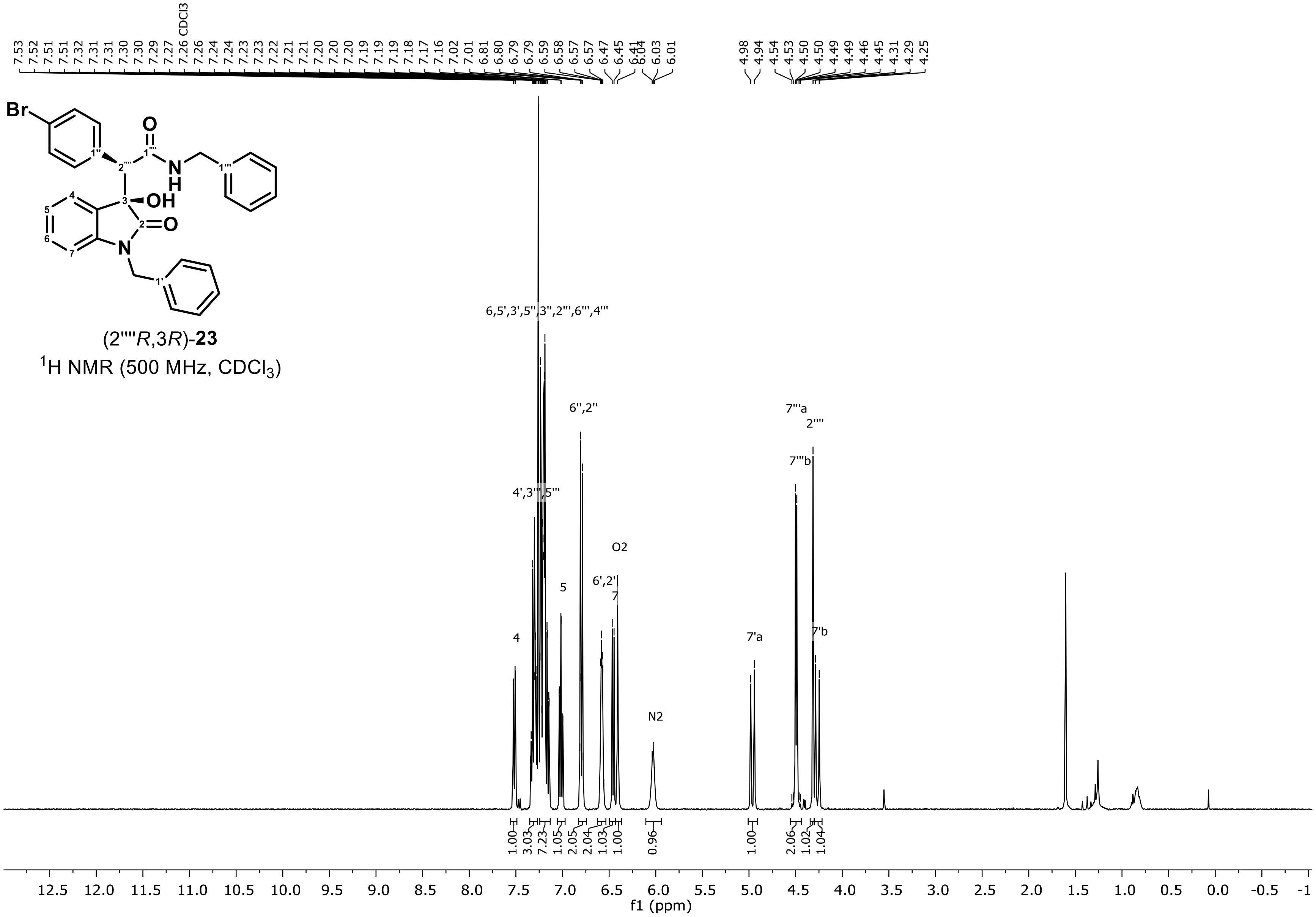

(2''''R,3R)-23
^{1}H NMR (500 MHz, CDCl$_3$)
f1 (ppm)

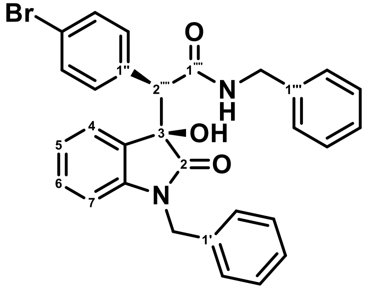

(2''''*R*,3*R*)-**23**

^{13}C {^{1}H} NMR (127 MHz, $CDCl_3$)

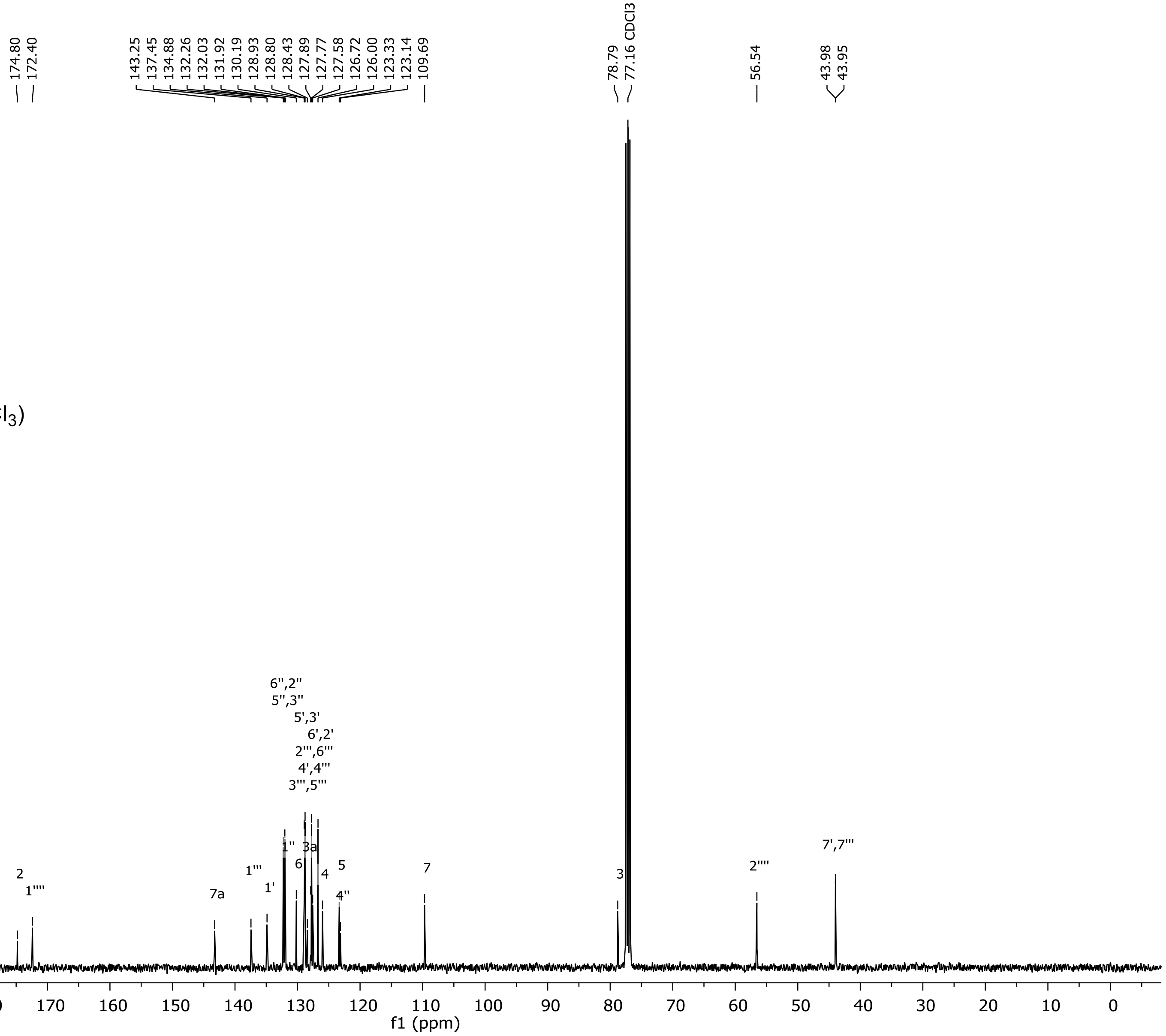

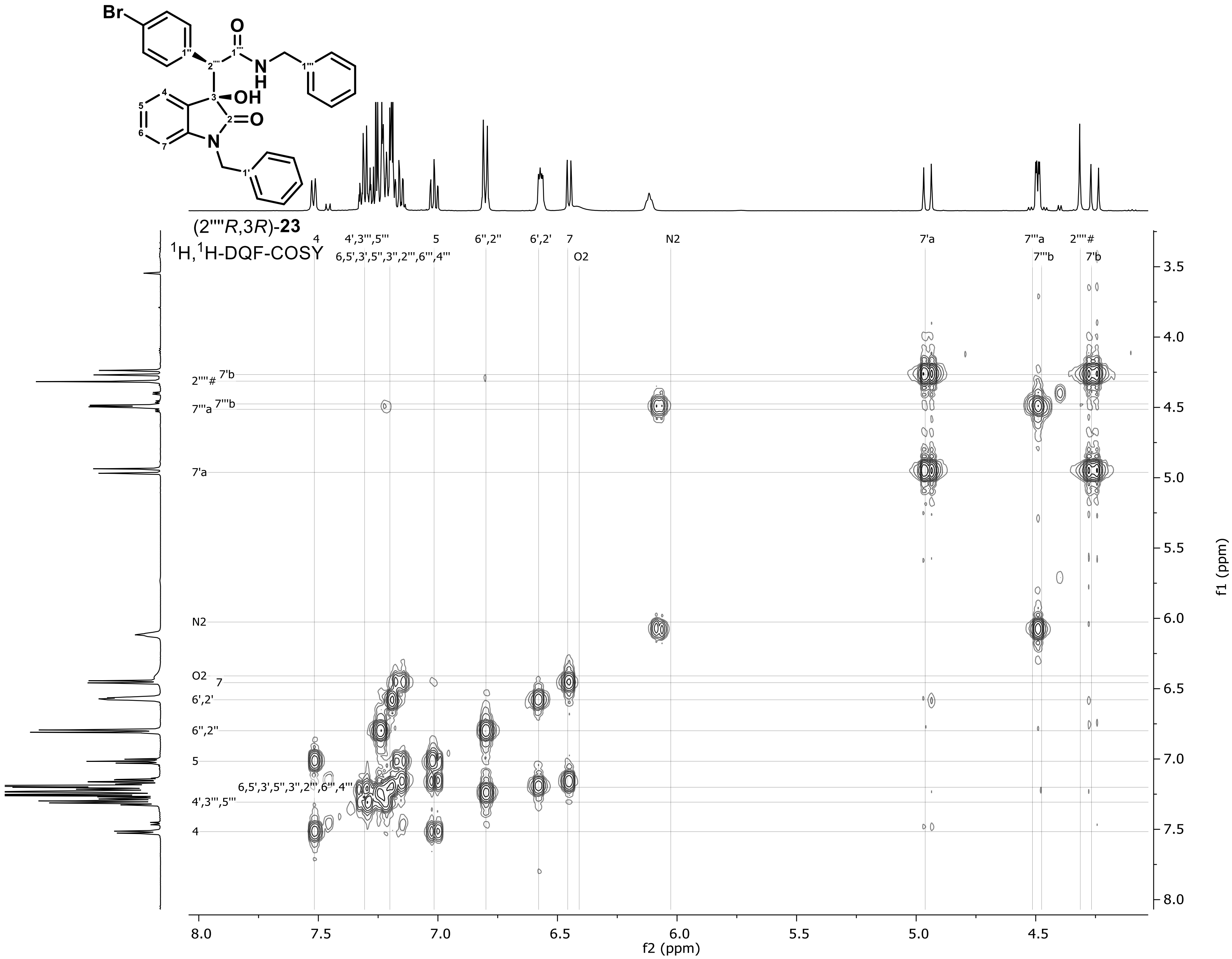

(2''''R,3R)-23
1H,1H-DQF-COSY
f2 (ppm)
f1 (ppm)

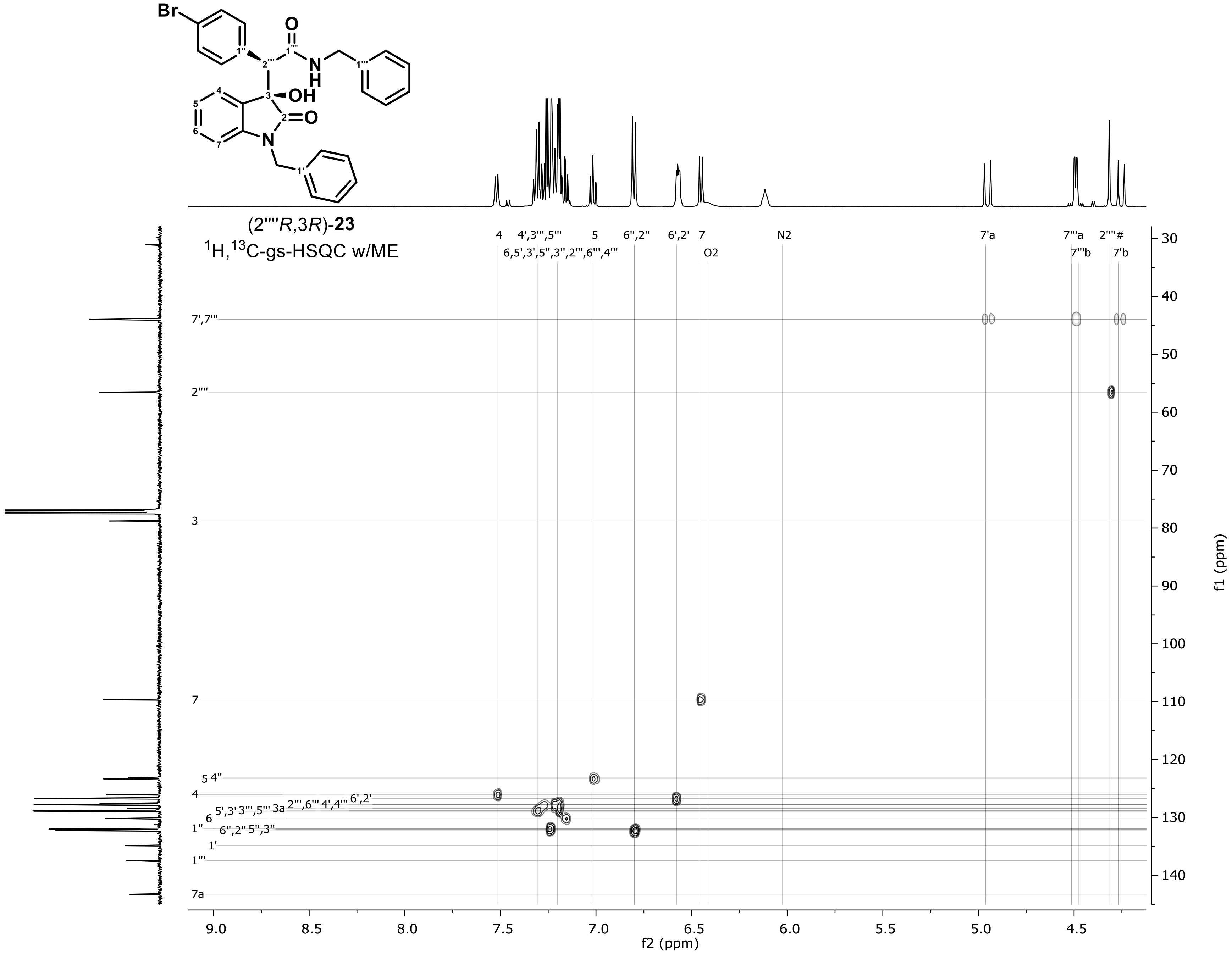
Br
O
N
H
OH
O
N
(2''''R,3R)-23
1H,13C-gs-HSQC w/ME
4
4',3''',5'''
6,5',3',5'',3'',2''',6''',4'''
5
6'',2''
6',2'
7
O2
N2
7'a
7'''a
7'''b
2''''#
7'b
7',7'''
2''''
3
7
5 4''
4
6 5',3' 3''',5''' 3a 2''',6''' 4',4''' 6',2'
1'' 6'',2'' 5'',3''
1'
1'''
7a
f2 (ppm)
f1 (ppm)

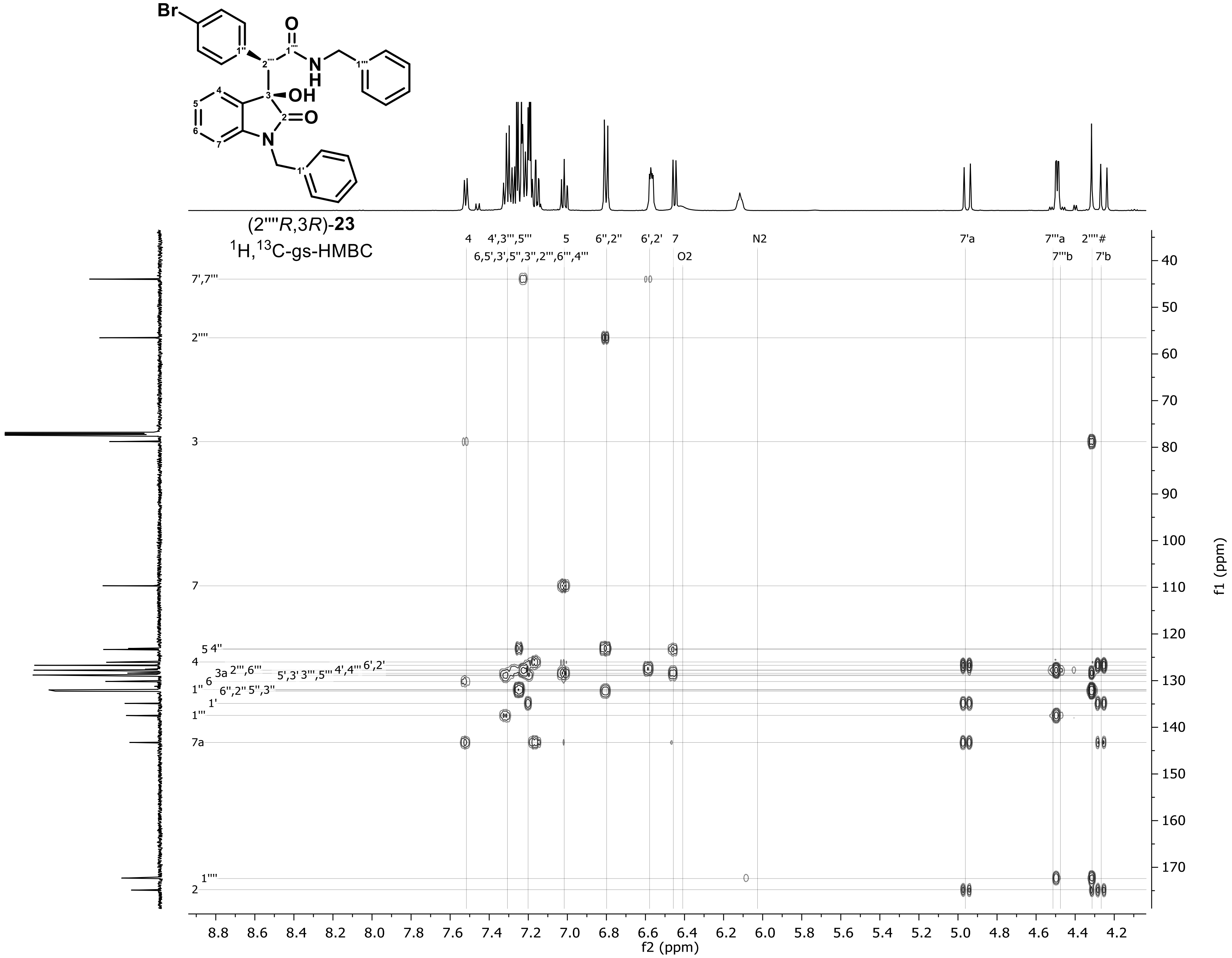
Br
O
N
H
OH
O
N
(2''''R,3R)-23
1H,13C-gs-HMBC
4
4',3''',5'''
6,5',3',5'',3'',2''',6''',4'''
5
6'',2''
6',2'
7
O2
N2
7'a
7'''a
7'''b
2''''#
7'b
7',7'''
2''''
3
7
5 4''
4
3a 2''',6'''
6
5',3' 3''',5''' 4',4''' 6',2'
1'' 6'',2'' 5'',3''
1'
1'''
7a
1''''
2
f2 (ppm)
f1 (ppm)

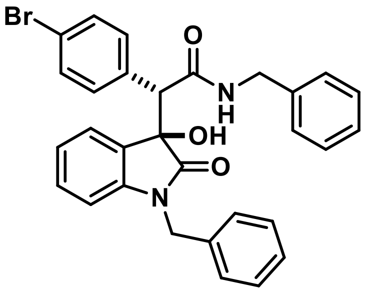

(±)-syn-**23**

PDA Ch1 211nm

Peak#	Ret. Time	Area%
1	23.336	49.389
2	30.466	50.611
Total		100.000

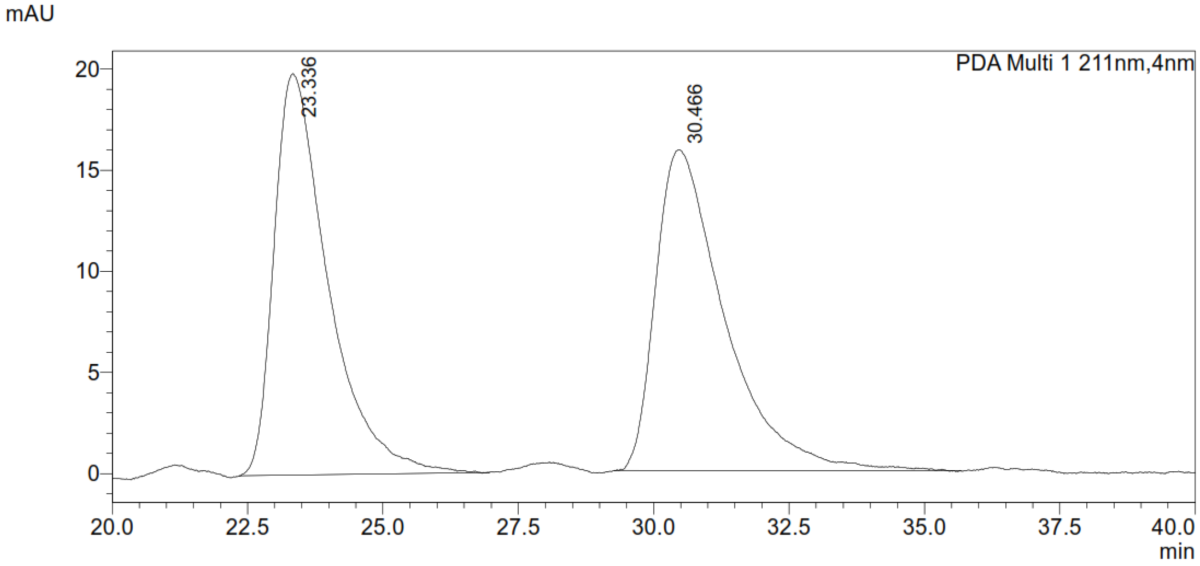

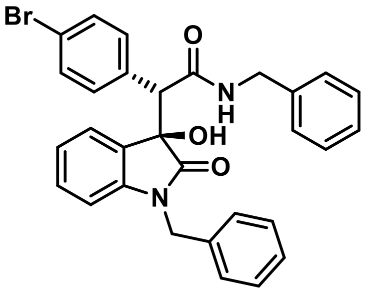

(–)-(2''''*S*,3*R*)-**23**

PDA Ch1 211nm

Peak#	Ret. Time	Area%
1	22.955	99.005
2	31.166	0.995
Total		100.000

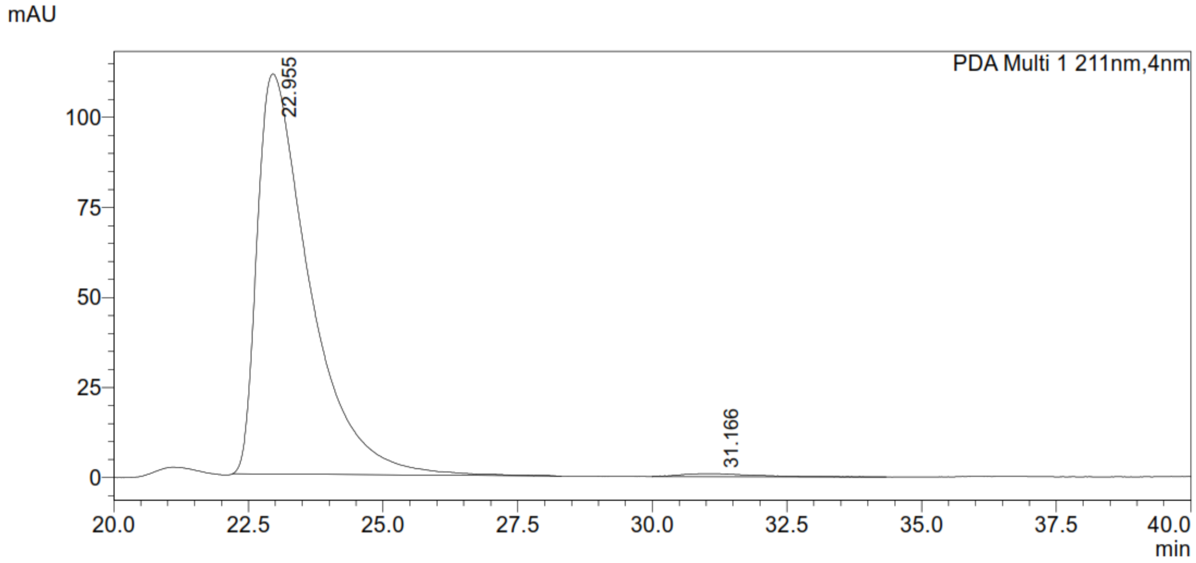

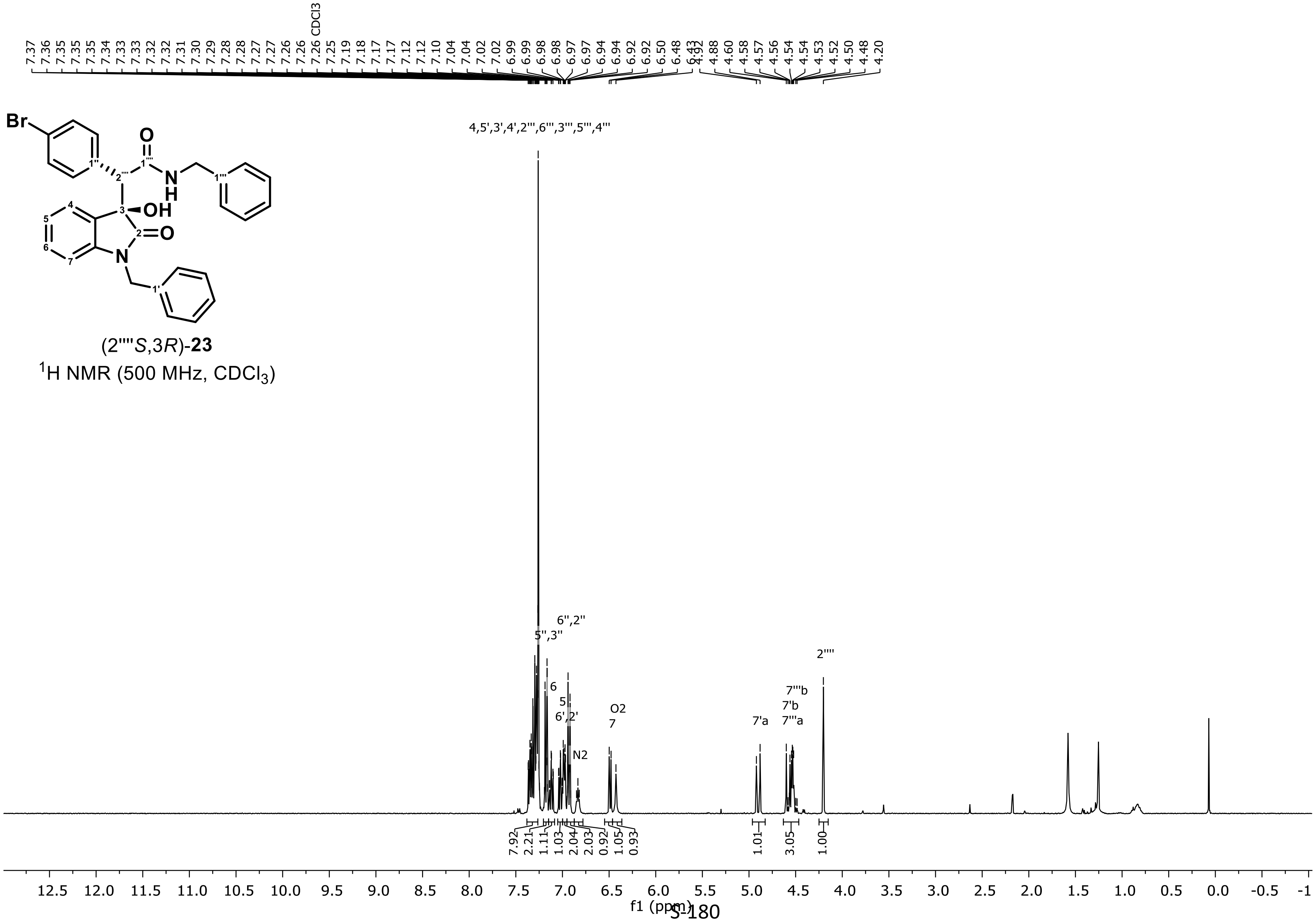

(2''''*S*,3*R*)-**23**

^{1}H NMR (500 MHz, $CDCl_3$)

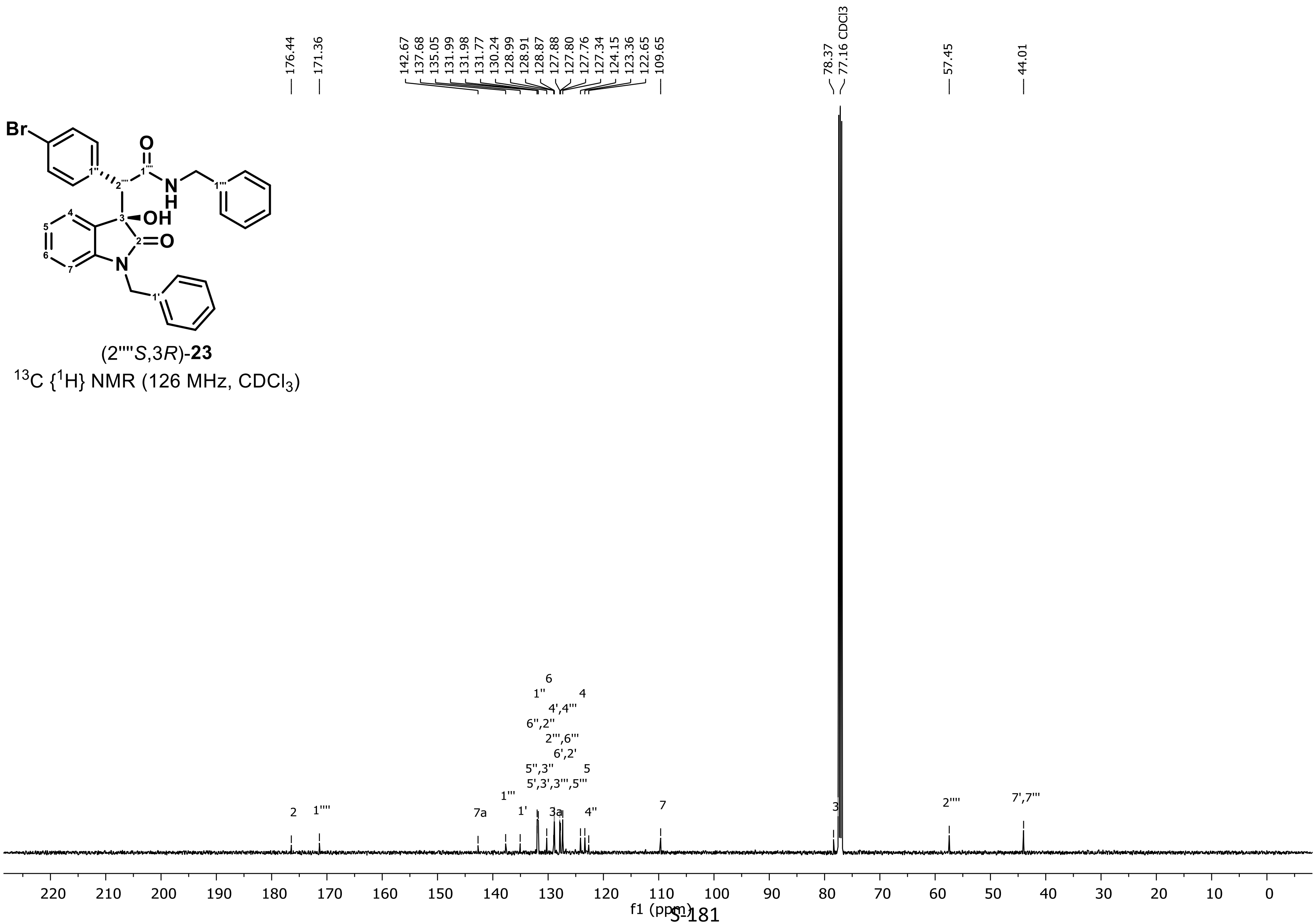

(2''''S,3R)-**23**

$^{13}C\{^{1}H\}$ NMR (126 MHz, $CDCl_3$)

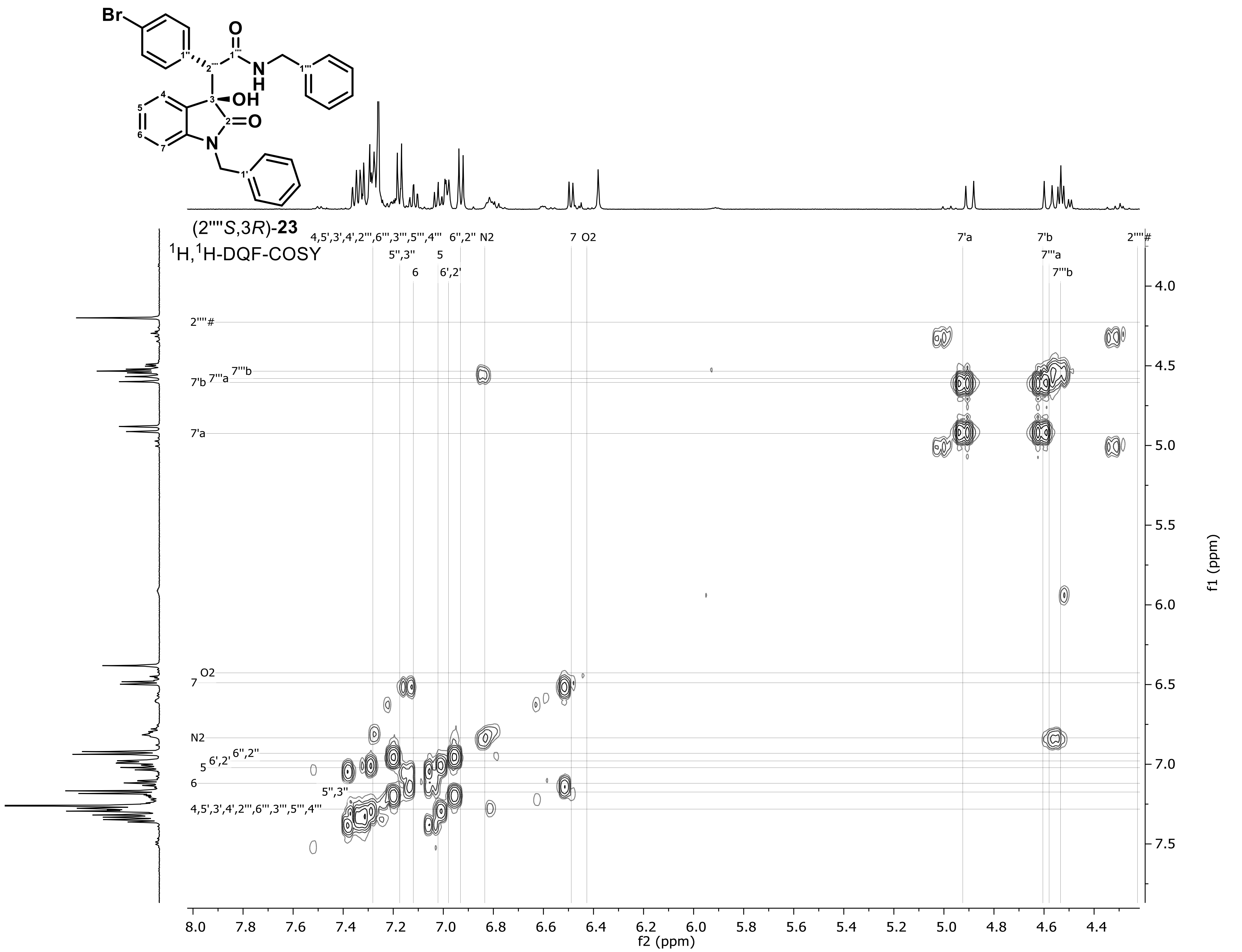
Br
O
N
H
OH
O
N
(2''''S,3R)-23
1H,1H-DQF-COSY
4,5',3',4',2''',6''',3''',5''',4'''
5'',3''
6
5
6',2'
6'',2''
N2
7
O2
7'a
7'b
7'''a
7'''b
2''''#
f1 (ppm)
f2 (ppm)

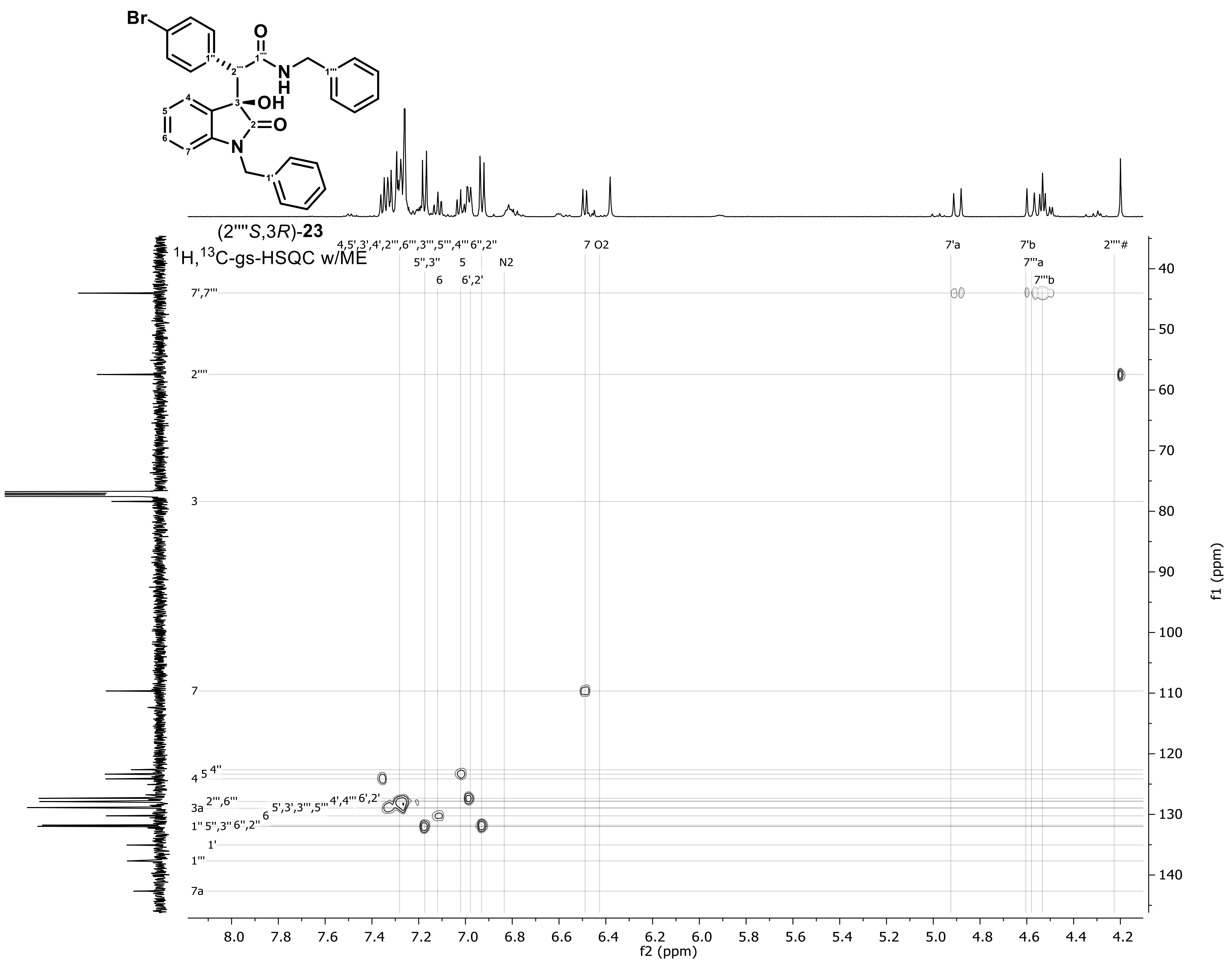

(2''''S,3R)-23
^{1}H,^{13}C-gs-HSQC w/ME
f2 (ppm)
f1 (ppm)

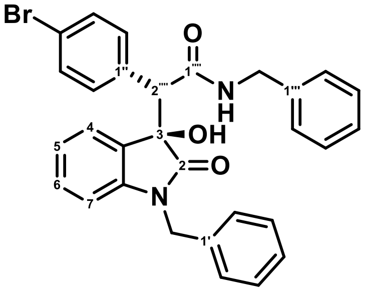

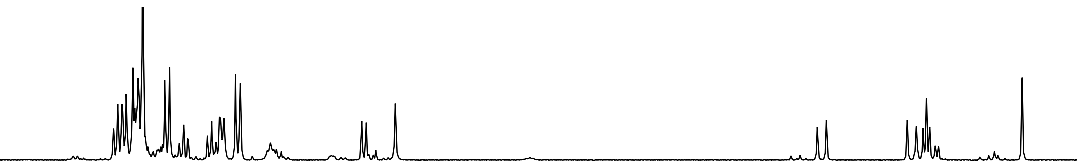

(2''''*S*,3*R*)-**23**

$^{1}H,^{13}C$-gs-HMBC

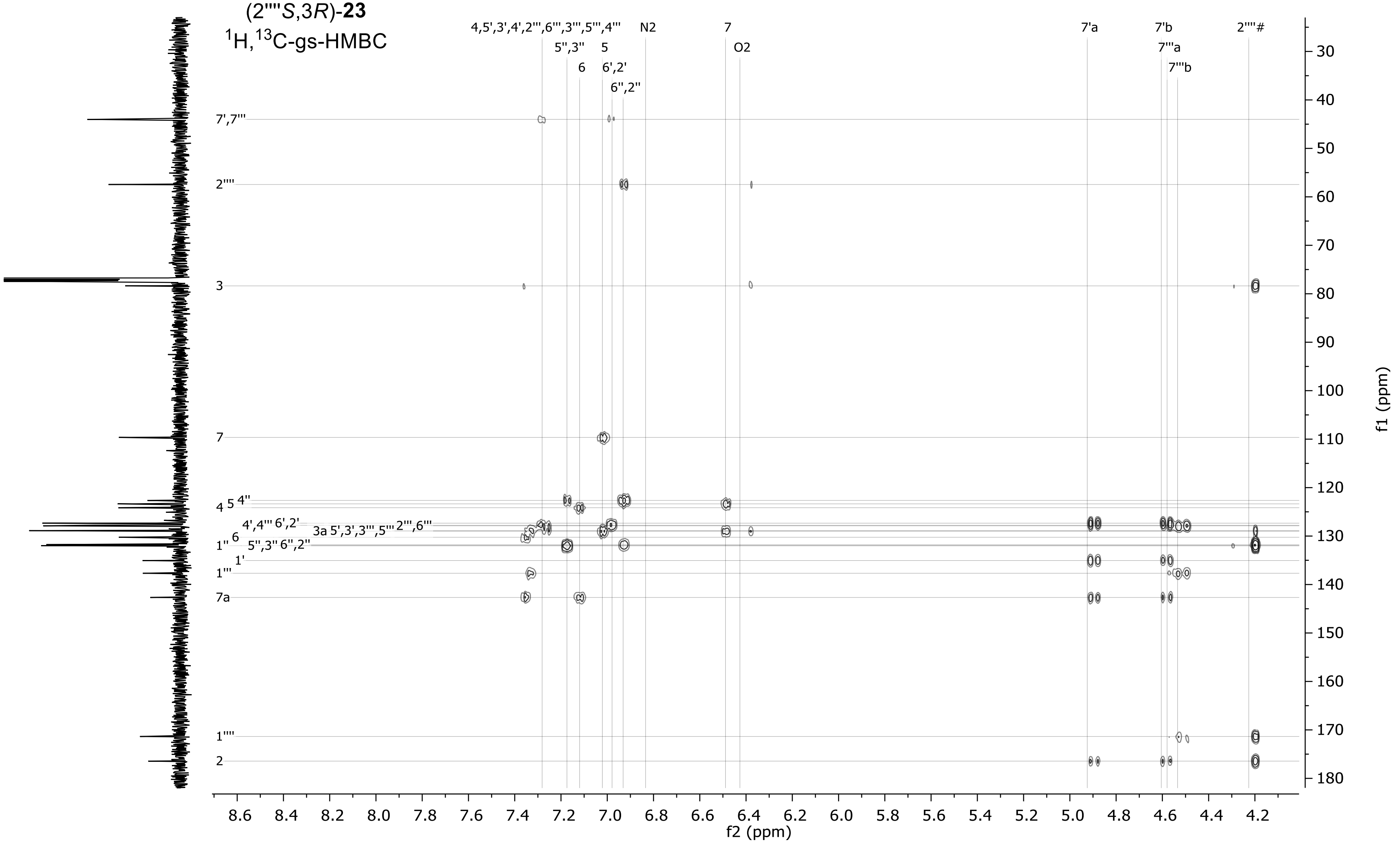

r) *N,N'*-Dibenzyl-2-(3-hydroxy-2-oxoindolin-3-yl)-2-*m*-bromophenylacetamide (24)

To a 25 ml round bottomed flask was added 2-(3-bromophenyl)acetic anhydride (155 mg, 0.375 mmol), *N*-benzylisatin (59.3 mg, 0.250 mmol) and (2*R*,3*S*)-HyperBTM (3.9 mg, 0.012 mmol). The mixture was cooled to 0 °C and CH_2Cl_2 (6.0 ml, 0.04 M) and Hünig's base (54.4 µL, 0.313 mmol) were added. The mixture was stirred at 0 °C for 3 h. Benzylamine (82.0 µL, 0.750 mmol) was added at 0 °C and the reaction was left to be stirred overnight at room temperature. 1,3,5-trimethoxybenzene (0.1 M soln in CH_2Cl_2, 500 µl, 0.05 mmol) was added and the solvent was removed under reduced pressure. Purification by column chromatography (15% – 35% EtOAc in Pentane) gave the title compound in two fractions (major diastereomer (35 mg, 26%) and minor diastereomer as white solids (9 mg, 7%); combined (44 mg, 0.081 mmol, 33%, 87:13 dr).

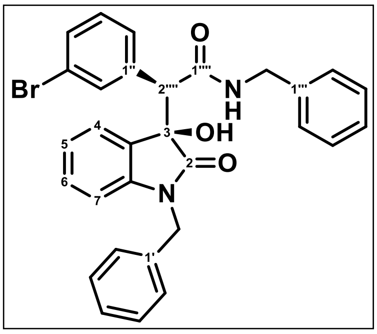

Major (2''''*R*,3*R*)-**24**: **Chiral HPLC analysis** Chiralpak IB (95:5 hexane:IPA, flow rate 2 ml·min^{-1}, 211 nm, 40 °C) t_R (2''''*R*,3*R*)-**24**: 17.0 min, t_R (2''''*S*,3*S*)-**24**: 21.7 min, >99:1 er; $\alpha_D^{20} = -8.9$ (*c* 0.95, $CHCl_3$); ν_{max} (thin film) 3341 (m, broad, OH, NH), 3084 (w), 3063 (w), 3030 (w), 2924 (w), 1717 (s, C=O, lactam), 1653 (s, C=O, amide), 1614 (s), 1558 (m), 1541 (m), 1489 (m), 1470 (s), 1454 (m, 1431 (w), 1368 (m), 1352 (m), 1223 (w), 1177 (w), 1119 (w), 1098 (w), 1078 (w), 1030 (w), 997 (w), 752 (s); **^{1}H NMR** (500 MHz, $CDCl_3$) δ_H 7.45 (1H, d, $^3J_{HH}$ = 7.5 Hz, ArC^4*H*), 7.38 (1H, ddd, $^3J_{HH}$ = 8.0 Hz, $^4J_{HH}$ = 1.9 Hz, 1.1 Hz, Ar(Br)C$^{4''}$*H*), 7.32 (2H, app t, $^3J_{HH}$ = 7.4 Hz, PhC$^{3''',5'''}$*H*), 7.29 – 7.25 (1H, m, PhC$^{4'''}$*H*), 7.22 (2H, app d, $^3J_{HH}$ = 7.4 Hz, PhC$^{2''',6'''}$*H*), 7.20 – 7.13 (4H, m, ArC^6*H*, PhC$^{3',4',5'}$*H*), 7.08 (1H, app t, $^4J_{HH}$ = 1.9 Hz, Ar(Br)C$^{2''}$*H*), 7.01 (1H, dd, $^3J_{HH}$ = 7.7 Hz, 7.5 Hz, ArC^5*H*), 6.97 (1H, dd, $^3J_{HH}$ = 8.0 Hz, 7.7 Hz, Ar(Br)C$^{5''}$*H*), 6.89 (1H, d, $^3J_{HH}$ = 7.7 Hz, Ar(Br)C$^{6''}$*H*), 6.67 (2H, app d, $^3J_{HH}$ = 7.1 Hz, PhC$^{2',6'}$*H*), 6.49 (1H, d, $^3J_{HH}$ = 7.9 Hz, ArC^7*H*), 6.41 (1H, d, $^4J_{HH}$ = 1.9 Hz, O*H*), 6.04 (1H, app dt, *J* = 7.0 Hz, $^3J_{HH}$ = 5.8 Hz, N*H*), 4.89 (1H, d, $^2J_{HH}$ = 15.8 Hz, NC*H*$_a$H$_b$-Ph), 4.52 (1H, dd, $^2J_{HH}$ = 14.9 Hz, $^3J_{HH}$ = 5.8 Hz, NHC*H*$_a$H$_b$-Ph), 4.49 (1H, dd, $^2J_{HH}$ = 14.9 Hz, $^3J_{HH}$ = 5.8 Hz, NHCH$_a$*H*$_b$-Ph), 4.34 (1H, d, $^2J_{HH}$ = 15.8 Hz, NCH$_a$*H*$_b$-Ph), 4.24 (1H, s, C*H*-Ar(Br)); **^{13}C {^{1}H} NMR** (127 MHz, $CDCl_3$) δ_C 175.0 (*C*(O)NBn), 172.1 (*C*(O)NHBn), 143.1 (Ar*C*^{7a}), 137.4 (Ph*C*$^{1'''}$CH$_2$NH), 135.2 (Ar(Br)*C*$^{1''}$CH), 135.0 (Ph*C*$^{1'}$CH$_2$N), 133.5 (Ar(Br)*C*$^{2''}$H), 131.8 (Ar(Br)*C*$^{4''}$H), 130.2 (Ar*C*^{6}H, Ar(Br)*C*$^{5''}$H), 128.9 and 128.8 (Ph*C*$^{3',5'}$H and Ph*C*$^{3''',5'''}$H), 128.2 (Ar*C*^{3a}), 127.9 (Ph*C*$^{4'''}$H), 127.7 (Ph*C*$^{2''',6'''}$H), 127.5 (Ph*C*$^{4'}$H), 126.8 (Ph*C*$^{2',6'}$H), 126.0 (Ar*C*^{4}H), 123.3 (Ar*C*^{5}H), 122.7 (Ar*C*$^{3''}$Br), 109.6 (Ar*C*^{7}H), 78.8 (*C*-OH), 56.6 (*C*H-Ar(Br)), 43.9$_4$ and 43.9$_1$ (N*C*H$_2$-Ph and NH*C*H$_2$-Ph); **HRMS** (ESI$^+$) *m/z* calcd for [M(^{79}Br)+Na]$^+$ $C_{30}H_{25}BrNaN_2O_3$ 563.0941, found 563.0927 (−2.5 ppm).

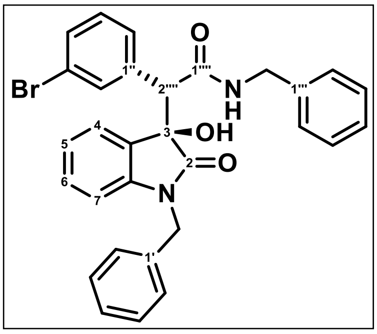

Minor (2''''*S*,3*R*)-**24**: **Chiral HPLC analysis** Chiralpak IB (95:5 hexane:IPA, flow rate 2 ml·min^{-1}, 211 nm, 40 °C) t_R (2''''*S*,3*R*)-**24**: 19.6 min, t_R (2''''*R*,3*S*)-**24**: 29.0 min, >99:1 er; $\alpha_D^{20} = -12.7$ (*c* 0.25, $CHCl_3$); ν_{max} (thin film) 3292 (m, broad, NH, OH), 3088 (w), 3063 (w), 3030 (w), 2955 (w), 2922 (w), 2853 (w), 1701 (s, C=O, lactam), 1639 (s, C=O, amide), 1612 (s), 1566 (m), 1489 (m), 1468 (s), 1454 (m), 1427 (w), 1373 (m), 1356 (m), 1298 (w), 1263 (w), 117 (w), 1179 (m), 1078 (m), 1030 (w), 997 (w), 935 (w), 797 (w), 752 (s); **^{1}H NMR** (500 MHz, $CDCl_3$) δ_H 7.21 – 7.36 (12H, m, O*H*, ArC^4*H*, PhC$^{3',4',5'}$*H*, Ar(Br)C$^{2'',4''}$*H*, PhC$^{2''',3''',4''',5''',6'''}$*H*), 7.11 (1H, app t, $^3J_{HH}$ = 7.9 Hz, ArC^6*H*), 7.08 – 7.04 (2H, m, PhC$^{2',6'}$*H*), 7.04 – 6.99 (2H, m, ArC^5*H*, Ar(Br)C$^{6''}$*H*), 6.97 – 6.91 (2H, m, Ar(Br)C$^{5''}$*H*), 6.88 (1H, t(br), $^3J_{HH}$ = 5.4 Hz, N*H*), 6.49 (1H, d, $^3J_{HH}$ = 7.8 Hz, ArC^7*H*), 4.83 (1H, d, $^2J_{HH}$ = 15.7 Hz, NC*H*$_a$H$_b$-Ph), 4.67 (1H, d, $^2J_{HH}$ = 15.7 Hz, NCH$_a$*H*$_b$-Ph), 4.57 (1H, dd, $^2J_{HH}$ = 15.0 Hz, $^3J_{HH}$ = 5.4 Hz, NHC*H*$_a$H$_b$-Ph), 4.52 (1H, dd, $^2J_{HH}$ = 15.0 Hz, $^3J_{HH}$ = 5.4 Hz, NHCH$_a$*H*$_b$-Ph), 4.19 (1H, s, C*H*-Ar); **^{13}C {^{1}H} NMR** (101 MHz, $CDCl_3$) δ_C 176.4 (*C*(O)NBn), 171.1 (*C*(O)NHBn), 142.6 (Ar*C*^{7a}), 137.7 (Ph*C*$^{1''}$CH''NH'), 135.4 (Ar(Br)*C*$^{1''}$CH), 135.2 (Ph*C*$^{1'}$CH$_2$N), 133.2 (Ar(Br)*C*$^{2''}$H), 131.5 (Ar(Br)*C*$^{4''}$H), 130.2 (Ar*C*^{6}H, Ar(Br)*C*$^{5''}$H), 128.9 (Ph*C*$^{3',5'}$H, Ph*C*$^{3''',5'''}$H), 128.8 (Ar*C*^{3a}), 128.6 (Ar(Br)*C*$^{6''}$H), 127.9 (Ph*C*$^{2''',6'''}$H),

127.8 (Ph$C^{4'}$H, Ph$C^{4''''}$H), 127.3 (Ph$C^{2',6'}$H), 124.2 (ArC^{4}H), 123.4 (ArC^{5}H), 122.8 (Ar$C^{3''}$Br), 109.7 (ArC^{7}H), 78.4 (*C*-OH), 57.8 (*C*H-Ar(Br)), 44.0 (N*C*H_2-Ph and NH*C*H_2-Ph); **HRMS** (ESI$^+$) *m/z* calcd for $[M+H]^+$ $C_{30}H_{26}BrN_2O_3$ 541.1121, found 541.1120 (−0.2 ppm).

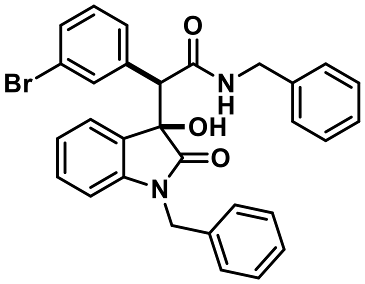

(±)-anti-**24**

PDA Ch1 211nm

Peak#	Ret. Time	Area%
1	17.079	49.605
2	21.483	50.395
Total		100.000

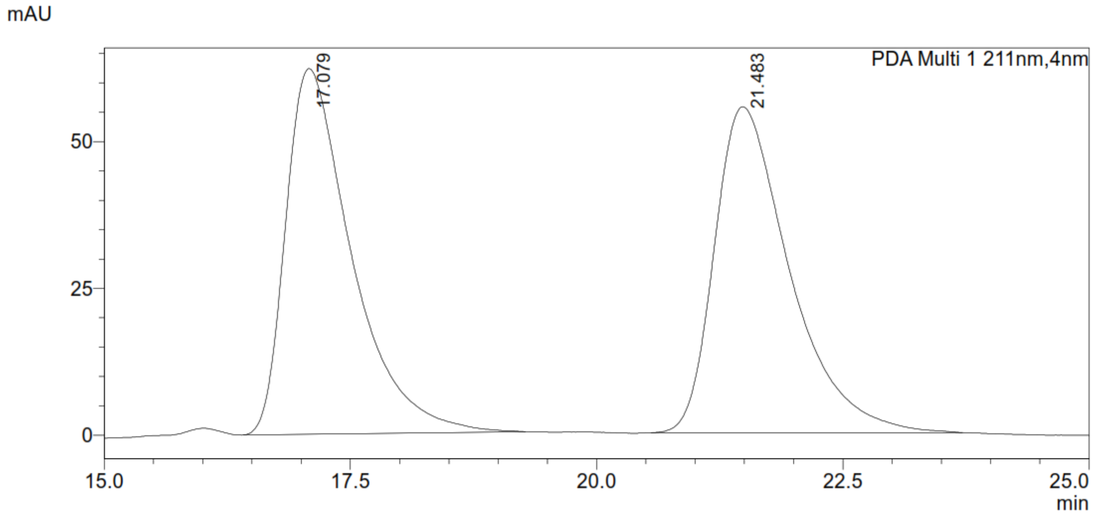

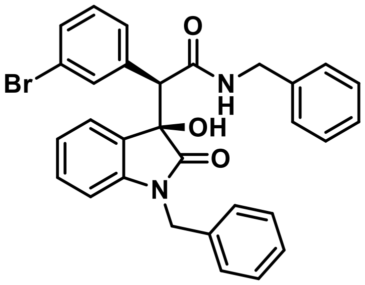

(–)-(2''''*R*,3*R*)-**24**

PDA Ch1 211nm

Peak#	Ret. Time	Area%
1	16.954	99.525
2	21.707	0.475
Total		100.000

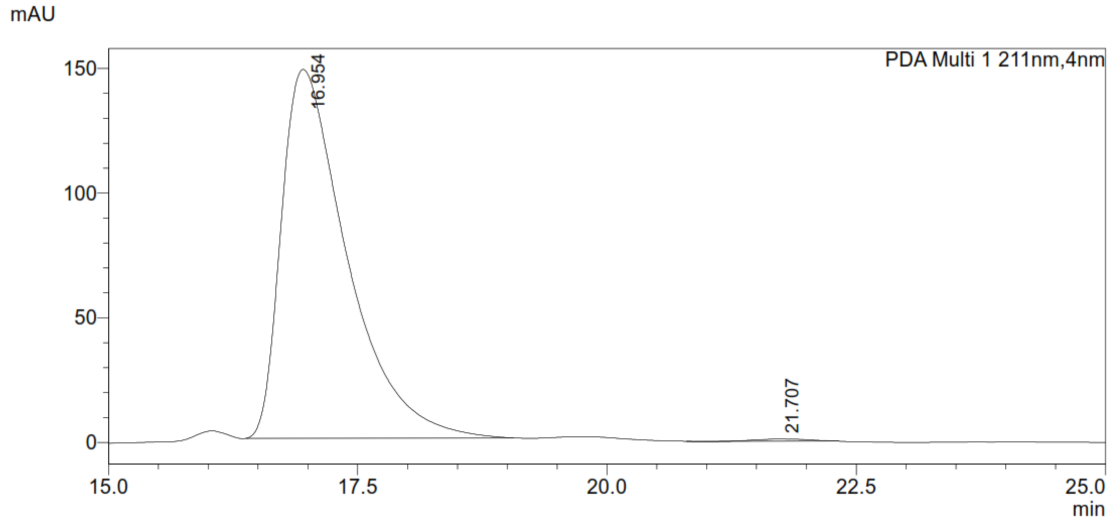

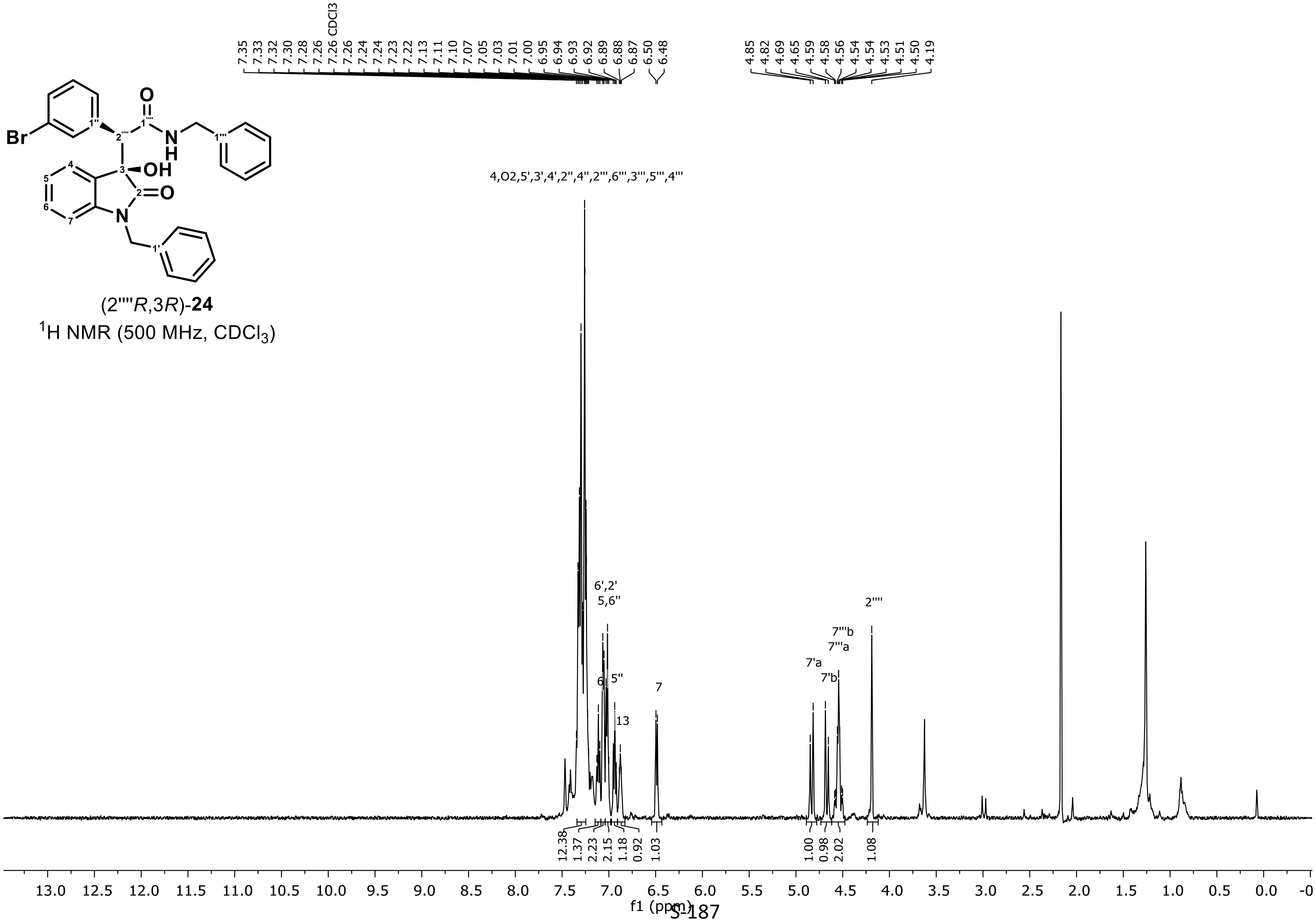

(2''''*R*,3*R*)-**24**

^{1}H NMR (500 MHz, $CDCl_3$)

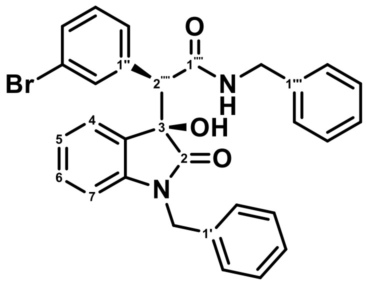

(2''''*R*,3*R*)-**24**

$^{13}C\ \{^{1}H\}$ NMR (127 MHz, $CDCl_3$)

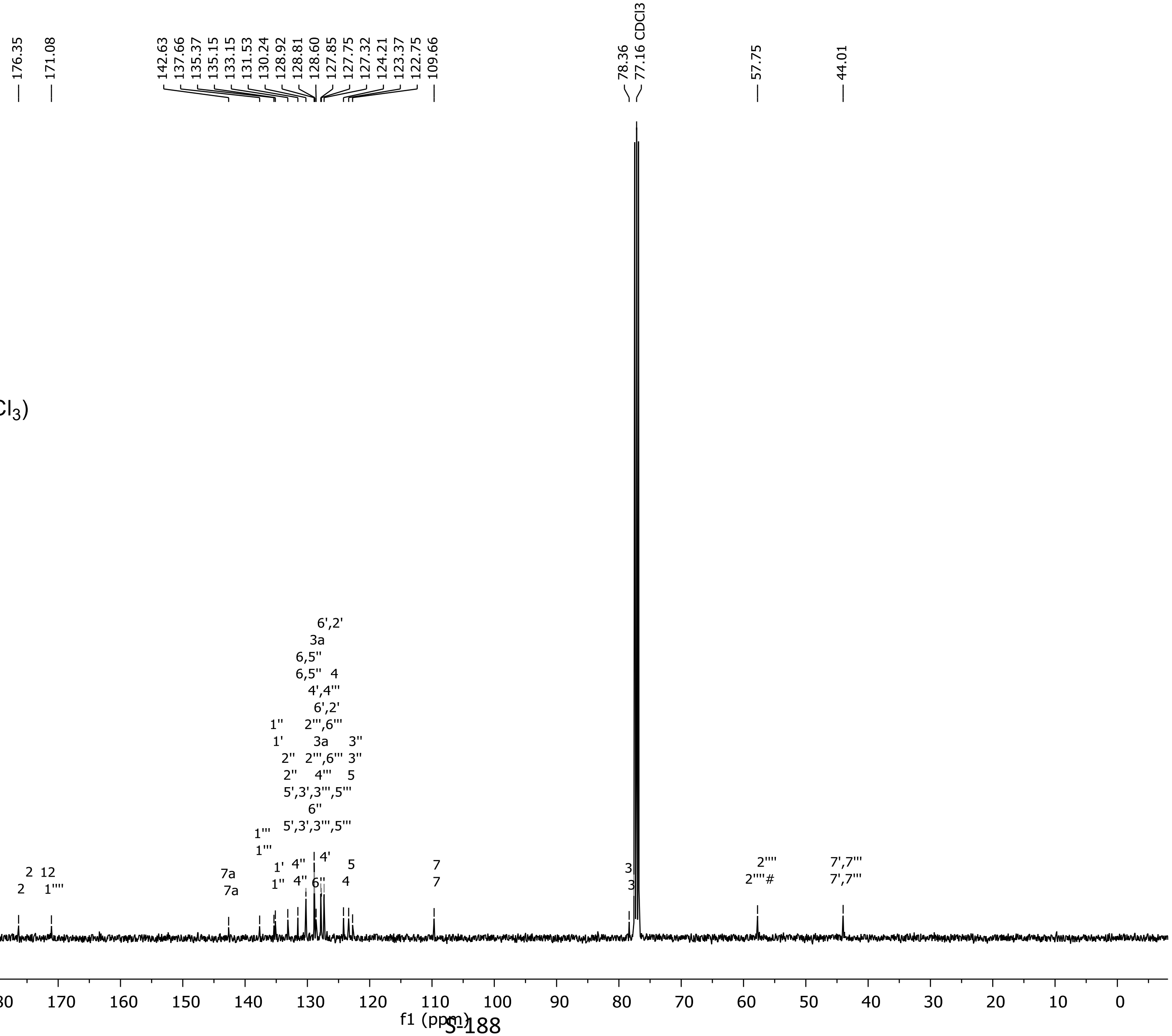

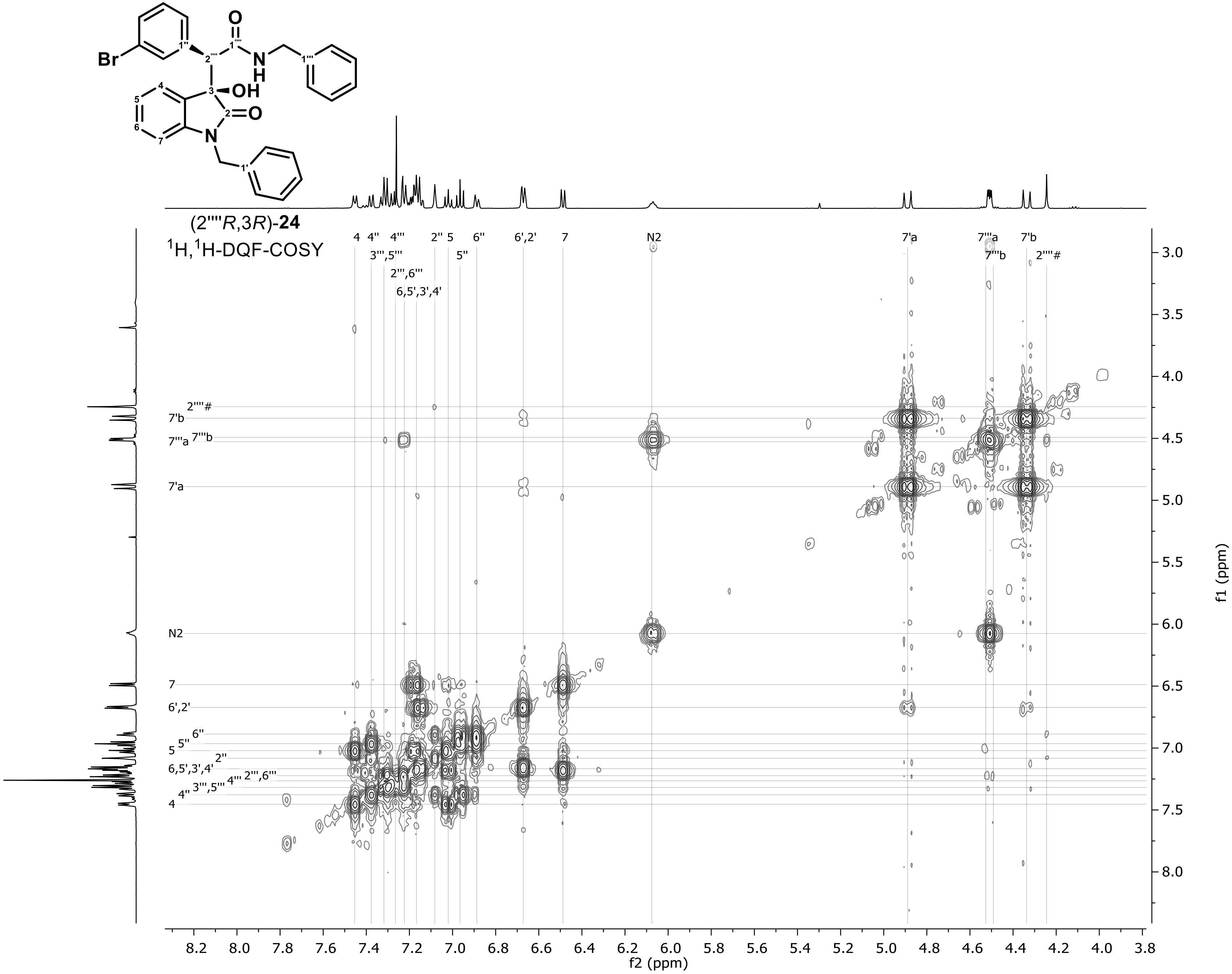

(2''''R,3R)-**24**

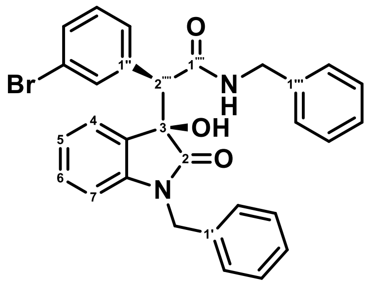

(2''''R,3R)-**24**

$^{1}H,^{13}C$-gs-HSQC w/ME

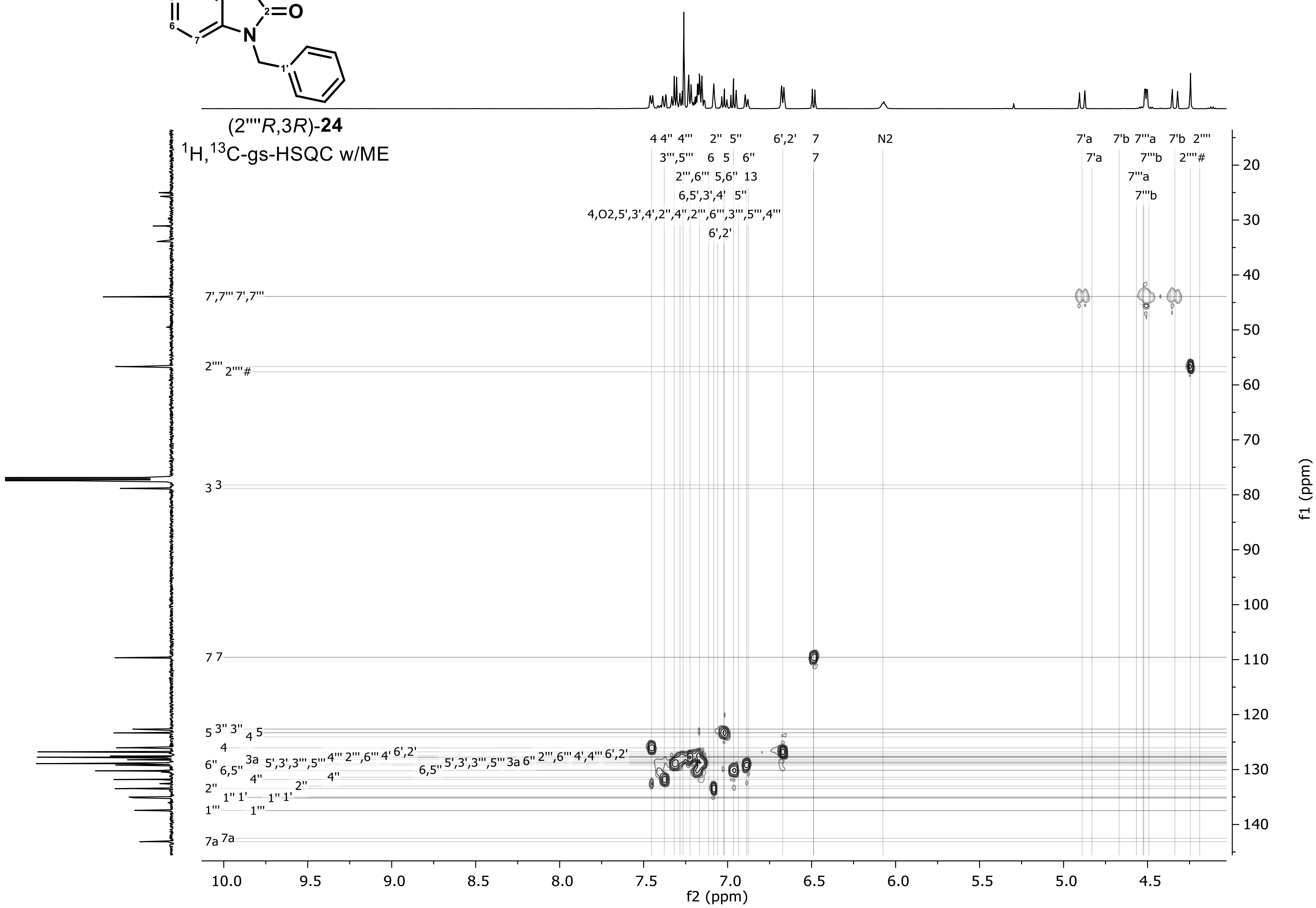

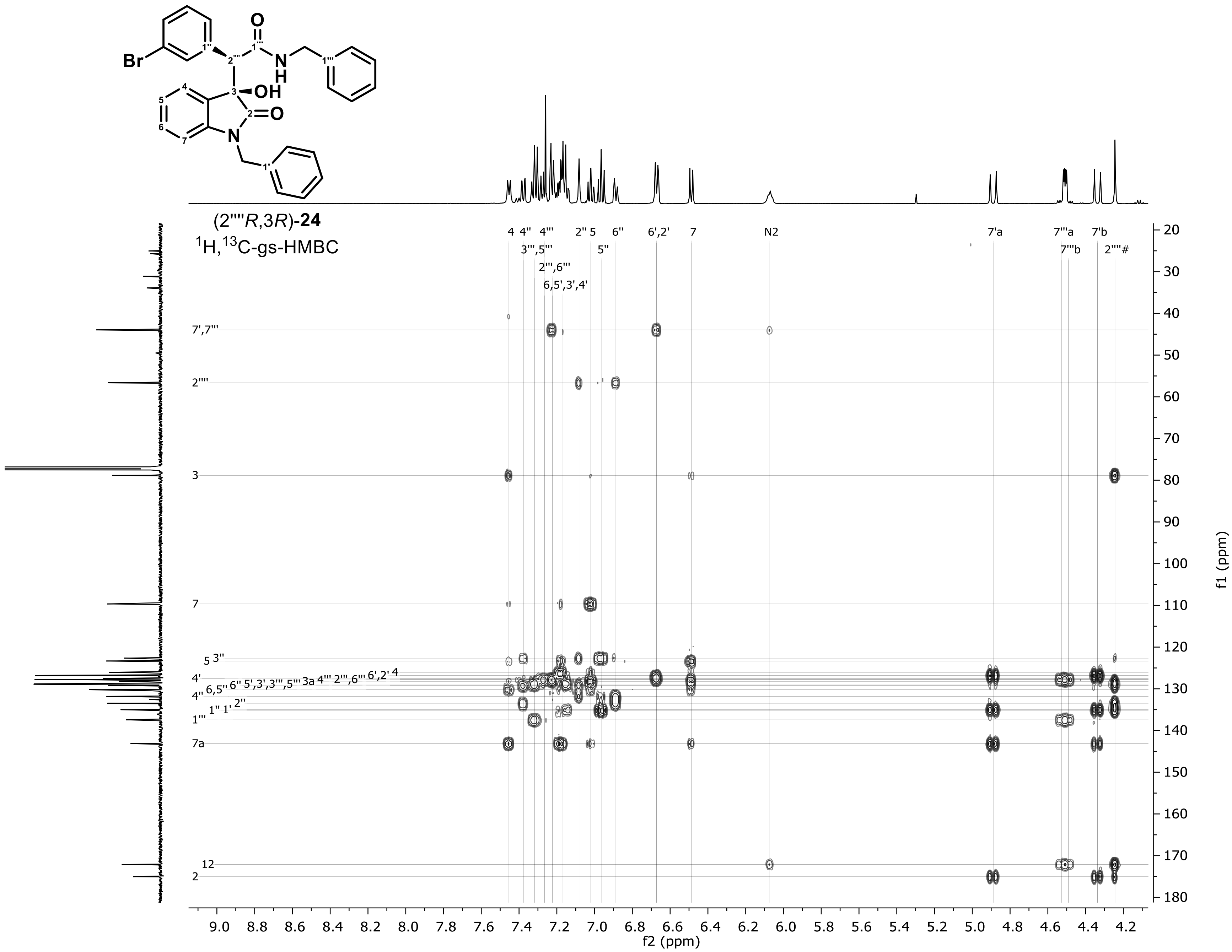

(2''''R,3R)-24
1H,13C-gs-HMBC
f2 (ppm)
f1 (ppm)

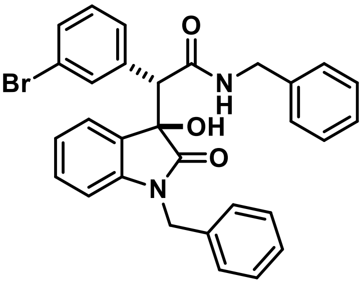

(±)-syn-**24**

PDA Ch1 211nm

Peak#	Ret. Time	Area%
1	19.506	49.872
2	28.240	50.128
Total		100.000

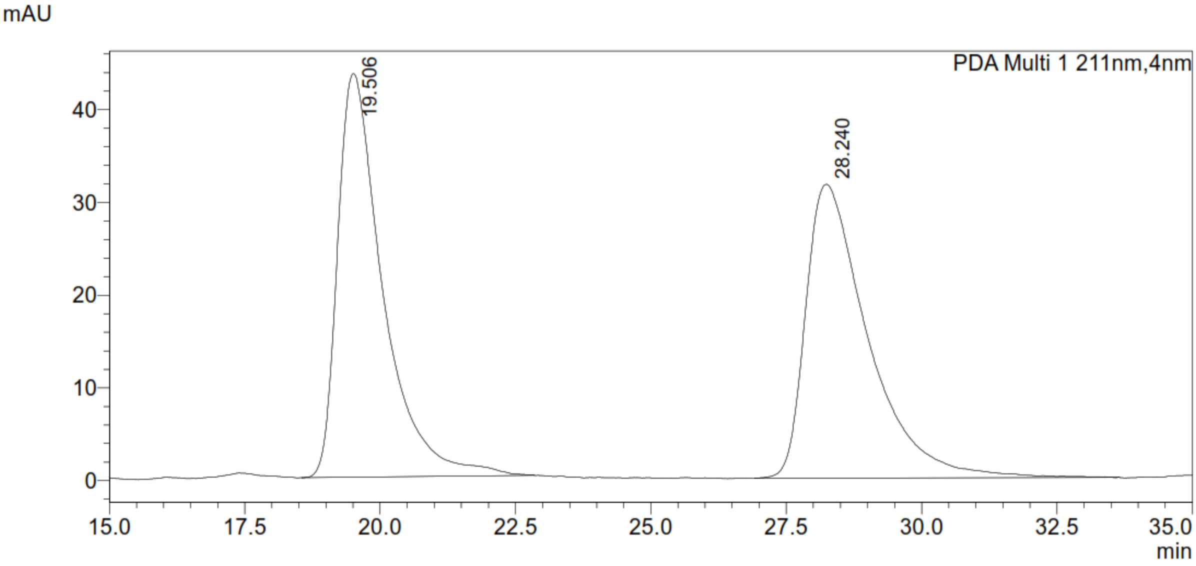

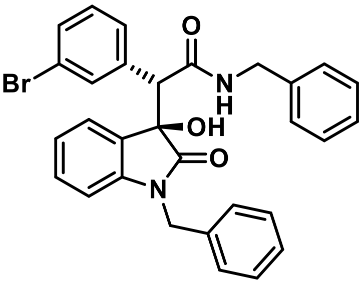

(–)-(2''''*S*,3*R*)-**24**

PDA Ch1 211nm

Peak#	Ret. Time	Area%
1	19.649	99.015
2	28.989	0.985
Total		100.000

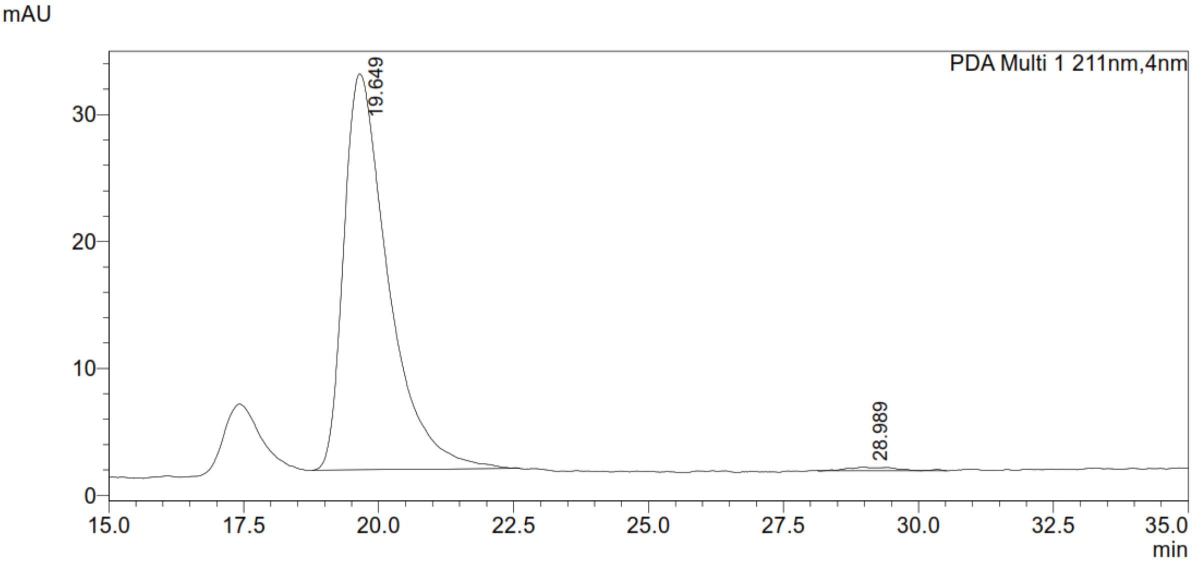

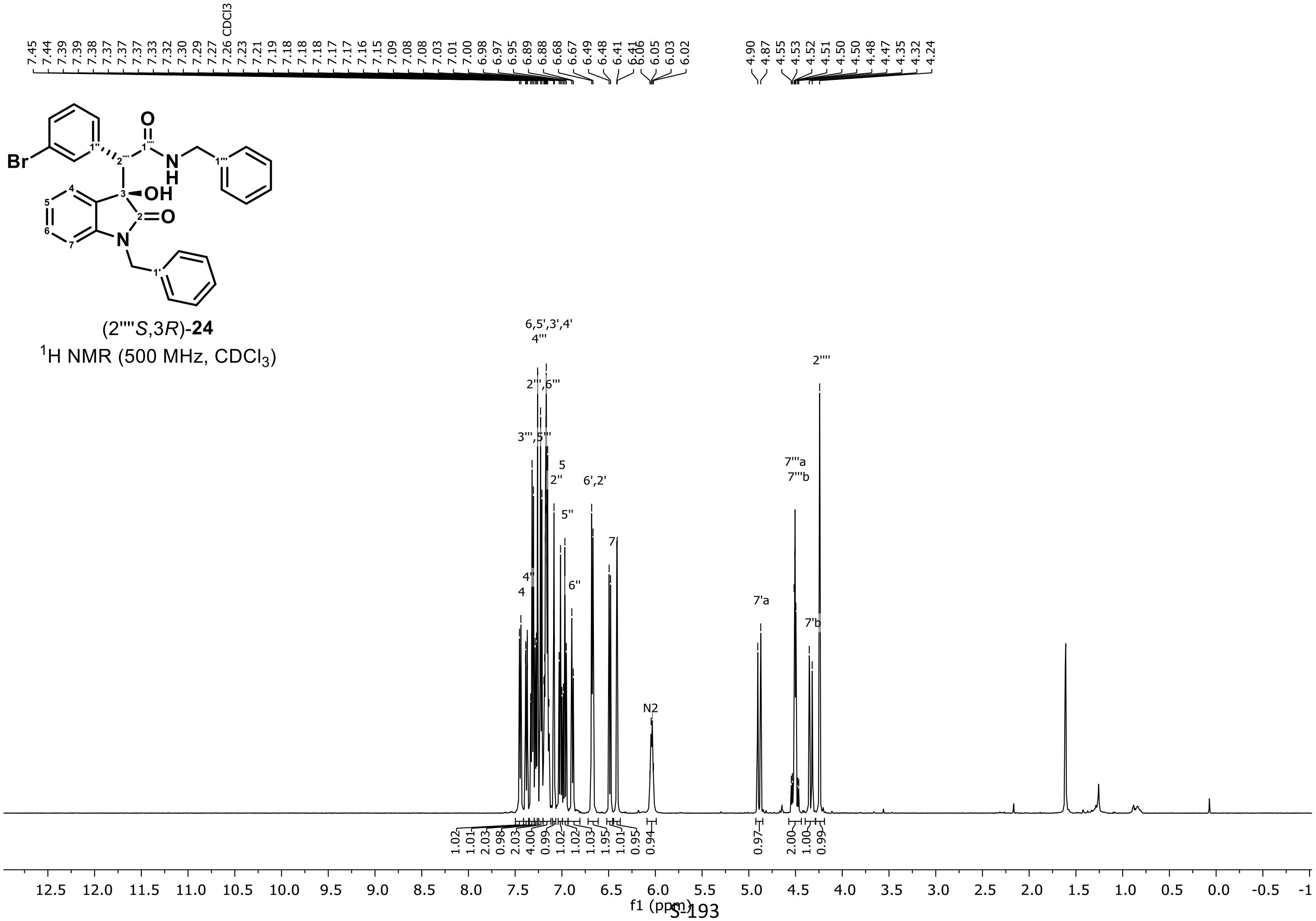

(2''''*S*,3*R*)-**24**

^{1}H NMR (500 MHz, $CDCl_3$)

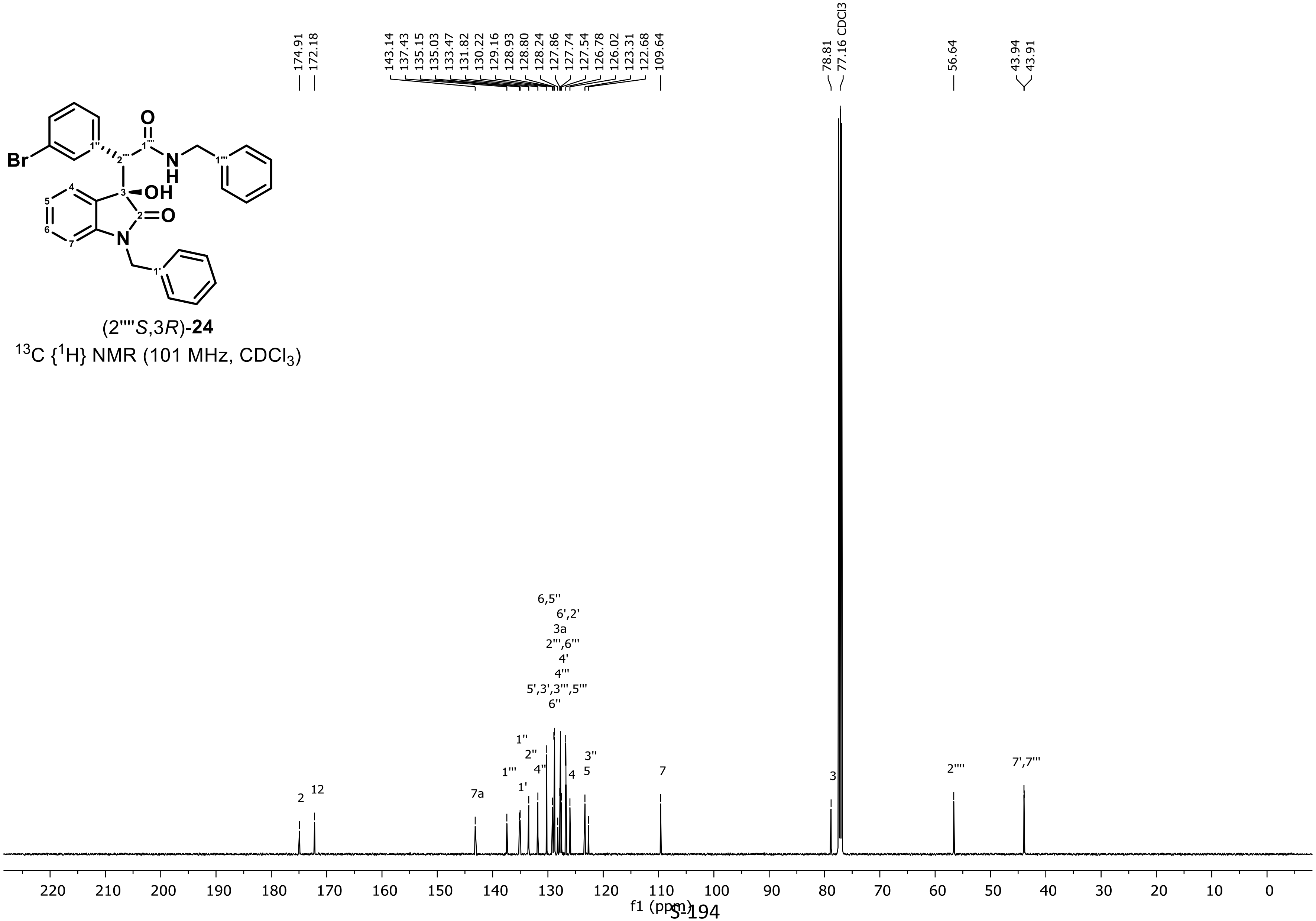

(2''''S,3R)-**24**

$^{13}C\{^{1}H\}$ NMR (101 MHz, $CDCl_3$)

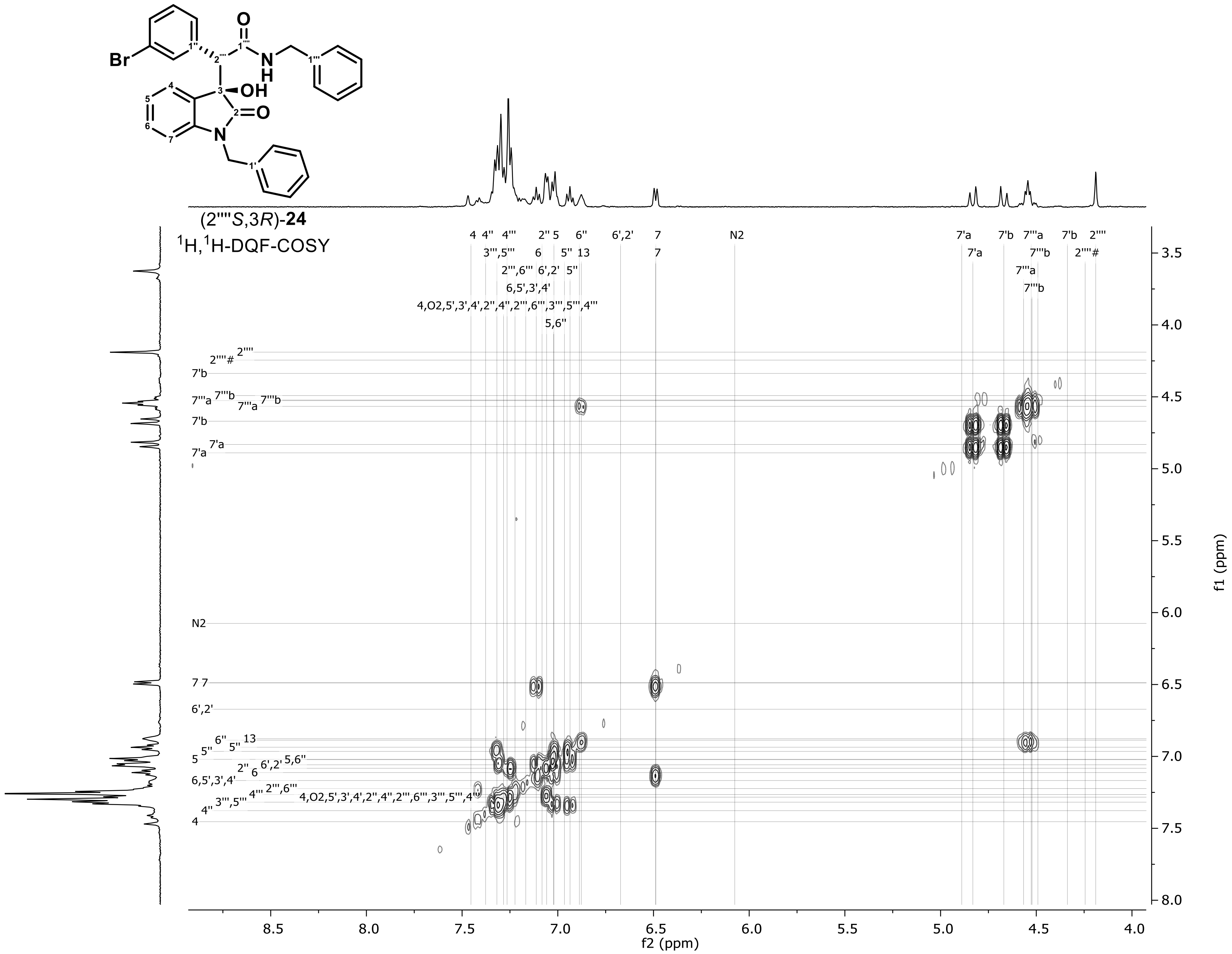

(2''''*S*,3*R*)-**24**

$^{1}H,^{1}H$-DQF-COSY

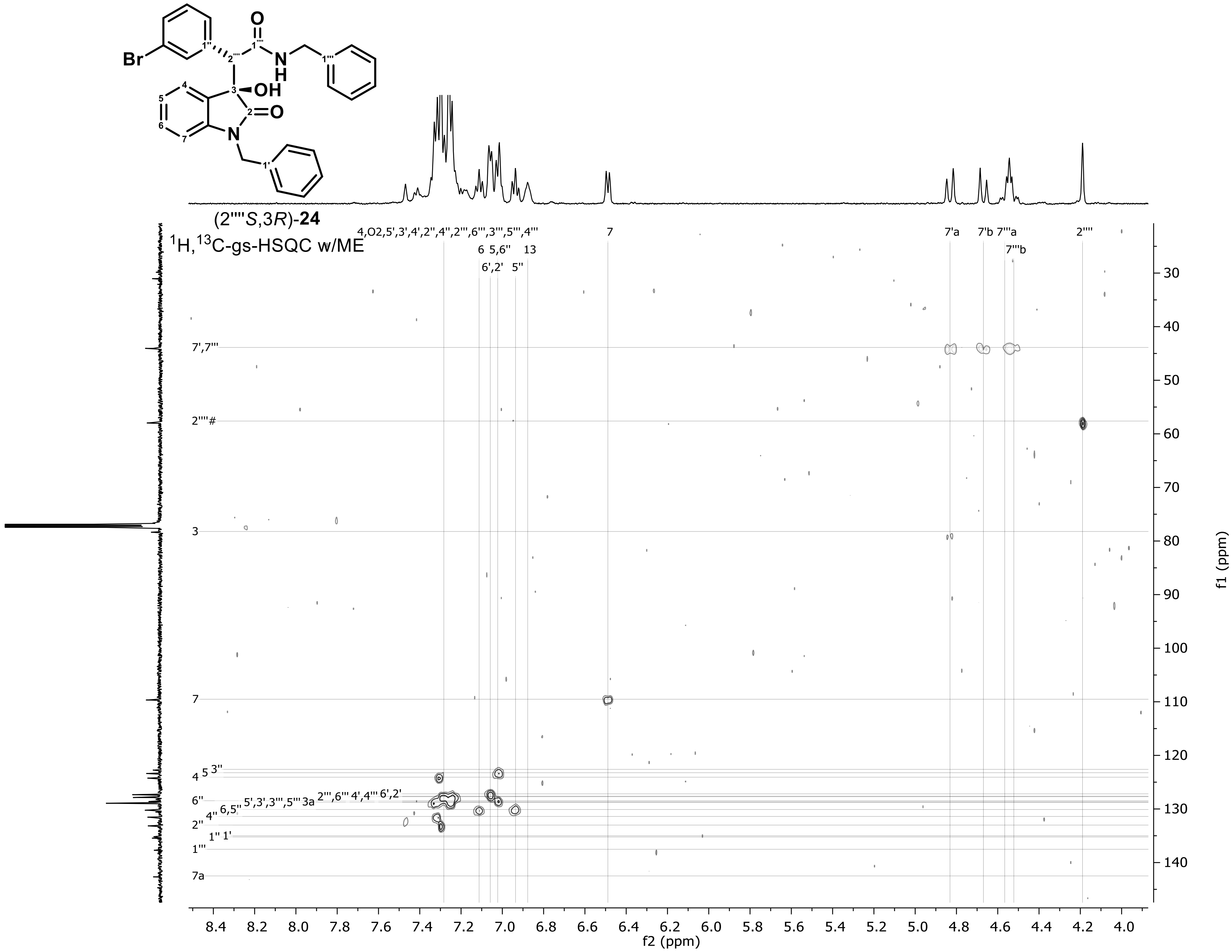
(2''''S,3R)-24
^{1}H,^{13}C-gs-HSQC w/ME
4,O2,5',3',4',2'',4'',2''',6''',3''',5''',4'''
6 5,6''
6',2' 5''
13
7
7'a 7'b 7'''a
7'''b
2''''
7',7'''
2''''#
3
7
4 5 3''
6''
2''
1'''
7a
f2 (ppm)
f1 (ppm)

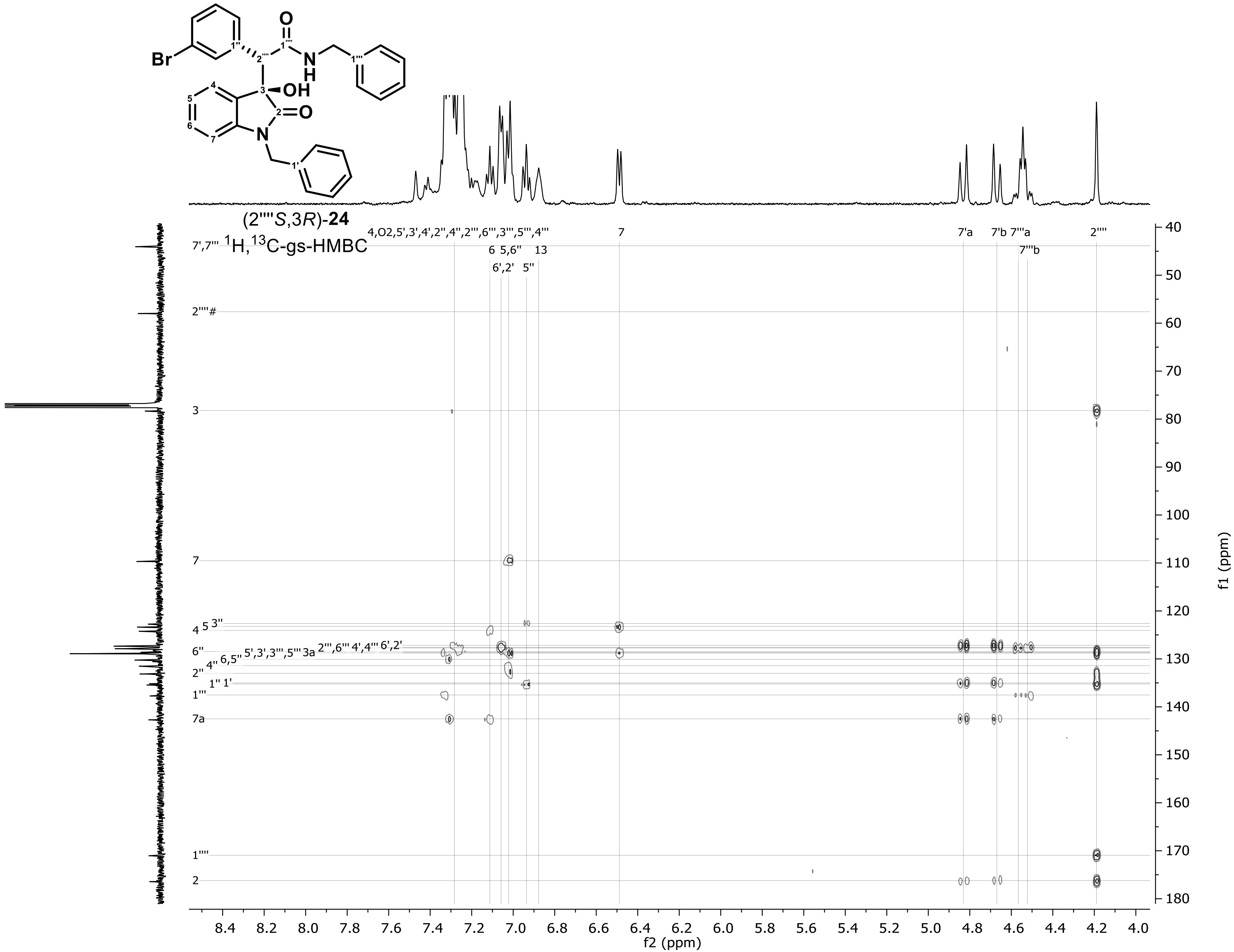

(2''''S,3R)-24
^{1}H,^{13}C-gs-HMBC
f2 (ppm)
f1 (ppm)

s) N,N′-dibenzyl-2-(3-hydroxy-2-oxindolin-3-yl)-2-naphth-1-ylacetamide (25)

To a 25 ml round bottomed flask was added phenylacetic anhydride (95.3 mg, 0.375 mmol), *N*-benzyl isatin (59.3 mg, 0.250 mmol), and (2*S*,3*R*)-HyperBTM (3.9 mg, 0.012 mmol). The mixture was cooled to 0 °C and CH_2Cl_2 (6.0 ml, 0.04 M) and Hünig's base (54 µl, 0.312 mmol) were added. The mixture was stirred at 0 °C for 3 h. Benzylamine (82 µl, 0.750 mmol) was added and the reaction was left to be stirred overnight at room temperature. 1,3,5-trimethoxybenzene (0.1 M soln in CH_2Cl_2 500 µl, 0.05 mmol) was added and the solvent was removed under reduced pressure (88:12 d.r.). Purification by column chromatography (Hexane:EtOAc 9:1 → 1:1, recolumn in same solvent system 1:0 → 23:2) gave the title compound as mixture of diastereomers as colourless semi-solid (87:13 d.r., 63.7 mg, 0.124 mmol, 50%).

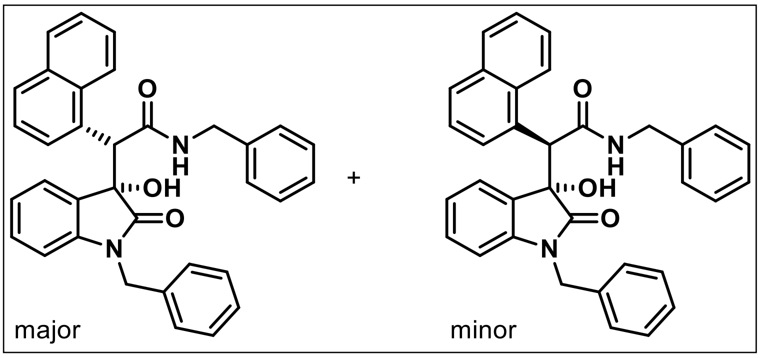

Major (2′′′′*S*,3*S*)-**25** and **minor** (2′′′′*R*,3*S*)-**25** analysed as 87:13 mixture of diastereomers: **m.p.** 145 – 147 °C (rac); $\mathbf{R_f}$ 0.50 (Hexane:EtOAc 1:1), yellow/orange (vanillin, heat); **Chiral HPLC** analysis (Chiralcel® IB 85:15 Hexane : IPA, flow rate 1.0 ml·min^{-1}, 211 nm, 40 °C) t_R (2′′′′*R*,3*R*)-**25**: 13.9 min, t_R (2′′′′*S*,3*S*)-**25**, 15.2 min, 1:99 e.r.; t_R (2′′′′*S*,3*R*)-**25**: 16.8 min, t_R (2′′′′*R*,3*S*)-**25**: 23.0 min, ≥3:97 e.r.; α_D^{20} = +80.7 (*c* 3.21, $CHCl_3$): ν_{max} (film) 3410 (m, broad, OH, NH), 3337 (m, broad, OH, NH), 3057 (w, CH), 3030 (w, CH), 3007 (w, CH), 2922 (w, CH); 1717 (s, C=O), 1645 (m, C=O), 1612 (s), 1520 (m), 1487 (m), 1468 (m), 1454 (m), 1431 (w), 1395 (w), 1354 (m), 1298 (w), 1256 (w), 1215 (w), 1175 (m), 1113 (w), 1096 (w), 1078 (w), 1028 (w), 1011 (w), 989 (w), 972 (w), 949 (w), 787 (s); **^{1}H NMR** (400 MHz, $CDCl_3$) δ_H 8.20 (0.87H, s(br), Ar*CH*), 8.11 (0.13H, d, $^3J_{HH}$ = 8.6 Hz, ArC*H*), 7.86 – 7.80 (1H, ArC*H* (major+minor), 7.77 (0.87H, d, $^3J_{HH}$ = 8.3 Hz, ArC*H*), 7.76 – 7.71 (0.13H, m, ArC*H*), 7.66 (0.13H, d, $^3J_{HH}$ = 8.2 Hz, ArC*H*), 7.56 – 7.40 (3.13H, ArC^4*H* (major), 2ArC*H* (major), 4ArC*H* (minor)), 7.34 – 7.17 (5H, m, ArC^4*H* (minor), PhC$^{3''',5'''}$*H* (major+minor), 3ArC*H* (major), 2ArC*H* (minor)), 7.17 – 7.04 (5H, m, ArC^6*H* (major), 4ArCH (major), 5ArCH (minor)), 7.03 – 6.83 (4.87H, m, ArC^5*H* (major), ArC^6*H* (minor), PhC$^{3',5'}$*H* (major+minor), 2ArC*H* (major), ArC*H* (minor)), 6.73 (0.13H, app t, $^3J_{HH}$ = 7.6 Hz, ArC^5*H*), 6.46 (0.13H, d, $^3J_{HH}$ = 7.9 Hz, PhC$^{2',6'}$*H*), 6.40 (0.87H, d, $^3J_{HH}$ = 7.8 Hz, ArC^7*H*), 6.30 (1.74H, app d, $^3J_{HH}$ = 7.6 Hz, PhC$^{2',6'}$*H*), 6.23 (0.13H, s(br), N*H*), 5.98 (0.87H, s(br), N*H*), 5.33 (0.87H, s, NpC$^{1''}$C*H*), 5.20 (0.13H, s, NpC$^{1''}$C*H*), 4.92 (0.13H, d, $^2J_{HH}$ = 15.6 Hz, PhC$^{1'}$C*H*$_a$H$_b$N), 4.79 (0.87H, d, $^2J_{HH}$ = 15.8 Hz, PhC$^{1'}$C*H*$_a$H$_b$N), 4.70 (0.13H, d, $^2J_{HH}$ = 15.6 Hz, PhC$^{1'}$CH$_a$*H*$_b$N), 4.45 (2H, PhC1′′′C*H*$_2$NH, both diastereomers), 4.12 (0.87H, d, $^2J_{HH}$ = 15.8 Hz, PhC$^{1'}$CH$_a$*H*$_b$N); **^{13}C {^{1}H} NMR** (101 MHz, $CDCl_3$) δ 176.7 (*C*(O)NBn, minor), 174.9 (*C*(O)NBn, major), 173.8 (*C*(O)NHBn, major), 172.8 (*C*(O)NHBn, minor), 143.3 (Ar*C*^{7a}, major), 142.7 (Ar*C*^{7a}, minor), 137.5 (Ph*C*$^{1'''}$, major+minor), 135.5 (Ph*C*$^{1'}$, minor), 135.0 (Ph*C*$^{1'}$, major), 134.0 (Np*C*, major), 133.9 (Np*C*, minor), 132.3 (Np*C*^{8a}, major), 132.2 (Np*C*^{8a}, minor), 129.8 (Ar*C*), 129.7 (Ar*C*), 129.4 (Ar*C*), 129.3 (Ar*C*), 129.2 (Ar*C*), 129.0 (Ar*C*), 128.9 (Ar*C*), 128.8$_1$ (Ar*C*^{3a}, minor), 128.7$_7$ (Ph*C*$^{3''',5'''}$H, major), 128.7 (Ar*C*, minor), 128.5 (Ph*C*$^{3',5'}$H, major), 127.6$_9$ (Ar*C*, minor), 127.6$_7$ (Ar*C*, major), 127.6$_2$ (Ar*C*, minor), 127.5$_6$ (Ph*C*$^{2''',6'''}$H, major), 127.2 (Ar*C*), 127.1 (Ar*C*), 126.8 (Ar*C*, minor), 126.6 (Ph*C*$^{2',6'}$H, major), 126.3 (Ar*C*, major), 126.1 (Ar*C*, minor), 125.9 (Ar*C*, major), 125.1 (Ar*C*, minor), 124.9 (Ar*C*, major), 124.3 (Ar*C*^{4}H, minor), 123.4 (Ar*C*, major), 123.1 (Ar*C*^{5}H, major), 122.9 (Ar*C*, minor), 122.7 (Ar*C*^{5}H, minor), 109.4 (Ar*C*^{7}H, major), 109.1 (Ar*C*^{7}H, minor), 79.1 (*C*-OH, major), 78.7 (*C*-OH, minor), 50.4 (br, NpC$^{1''}$*C*H, major+minor), 44.0 (PhC$^{1'}$*C*H$_2$N), 43.8$_1$ (PhC$^{1'''}$*C*H$_2$NH, minor), 43.7$_5$ (PhC$^{1'}$*C*H$_2$N, PhC$^{1'''}$*C*H$_2$NH, major); ***m/z*** (ESI$^+$) 91 ([C_7H_7]$^+$ 25%), 196 (10%), 246 (45%), 266 (64%), 334 (22%), 354

(36%), 362 ([M-BnNHCOO]$^+$ 39%), 513 ([M+H]$^+$ 100%), 514 ([M(^{13}C)+H]$^+$ 39%), 515 ([M($^{13}C_2$)+H]$^+$ 7%), 540 (7%), 796 (9%); **HRMS** (ESI$^+$) *m/z* calcd for [M+H]$^+$ $C_{34}H_{29}O_3N_2$ 513.2173, found 513.2158 (−2.9 ppm).

(±)-anti-**25** + (±)-syn-**25**

PDA Ch1 211nm

Peak#	Ret. Time	Area%
1	13.945	42.282
2	15.229	6.638
3	16.786	45.428
4	23.040	5.652
Total		100.000

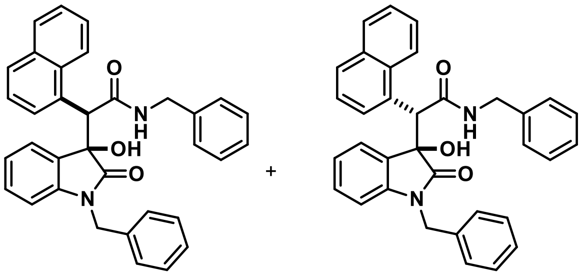

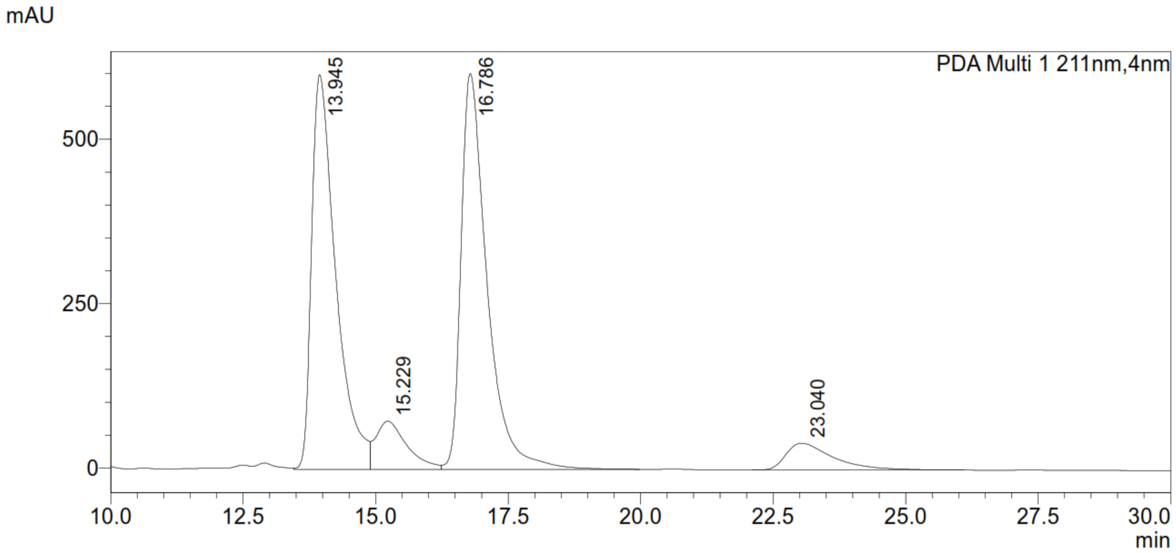

(2''''*S*,3*S*)-**25** + (2''''*R*,3*S*)-**25**

PDA Ch1 211nm

Peak#	Ret. Time	Area%
1	12.888	1.187
2	14.841	0.361
3	16.705	86.368
4	22.880	12.084
Total		100.000

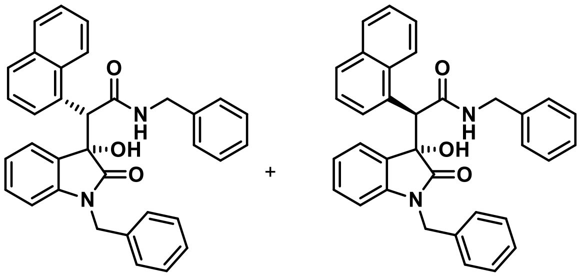

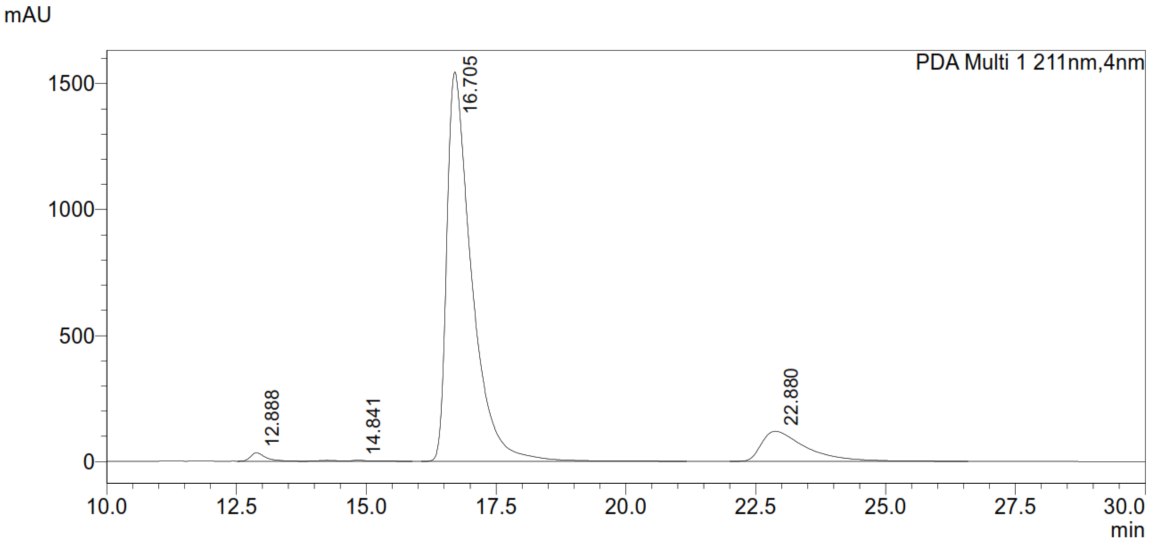

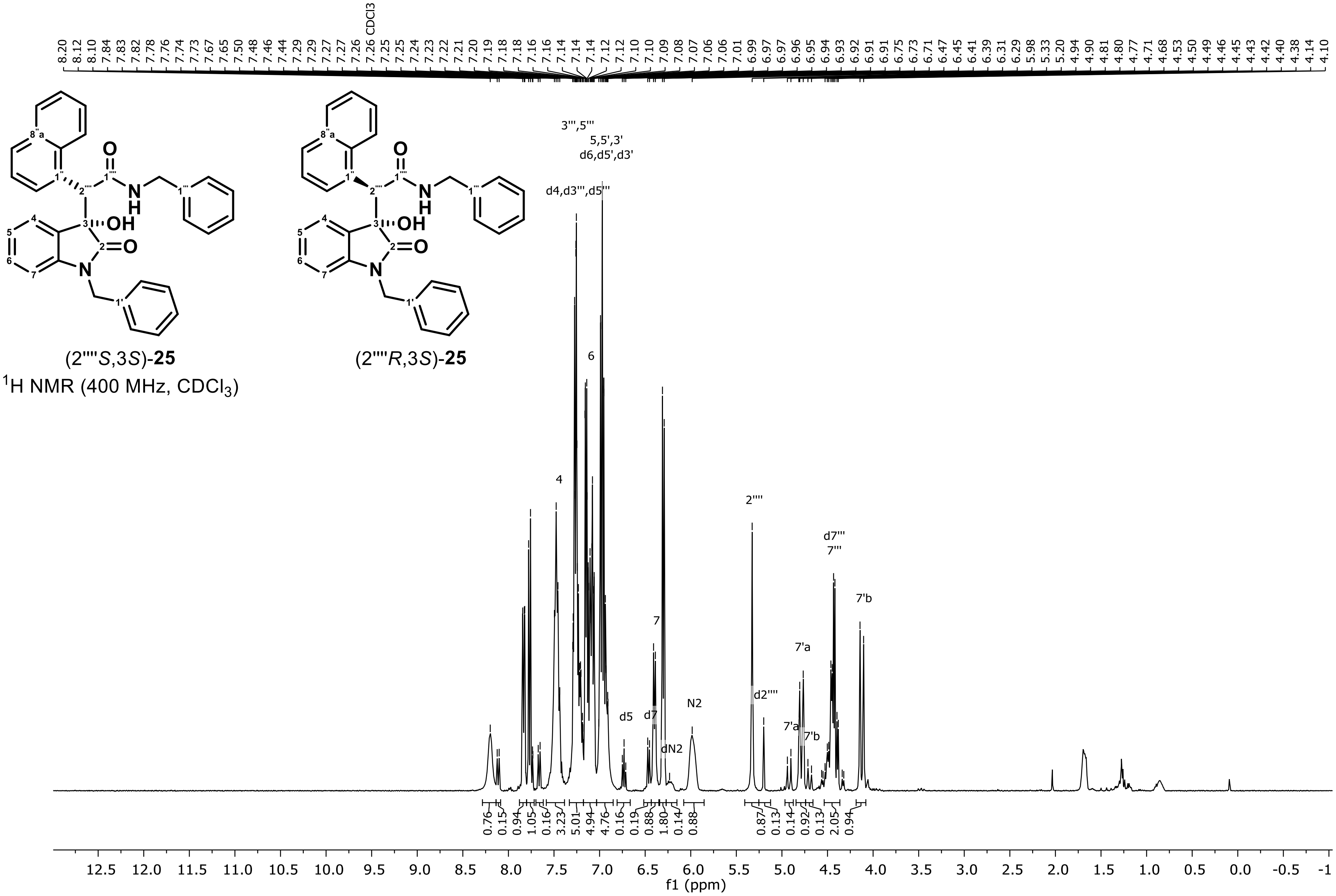

(2''''*S*,3*S*)-**25** (2''''*R*,3*S*)-**25**

^{1}H NMR (400 MHz, $CDCl_3$)

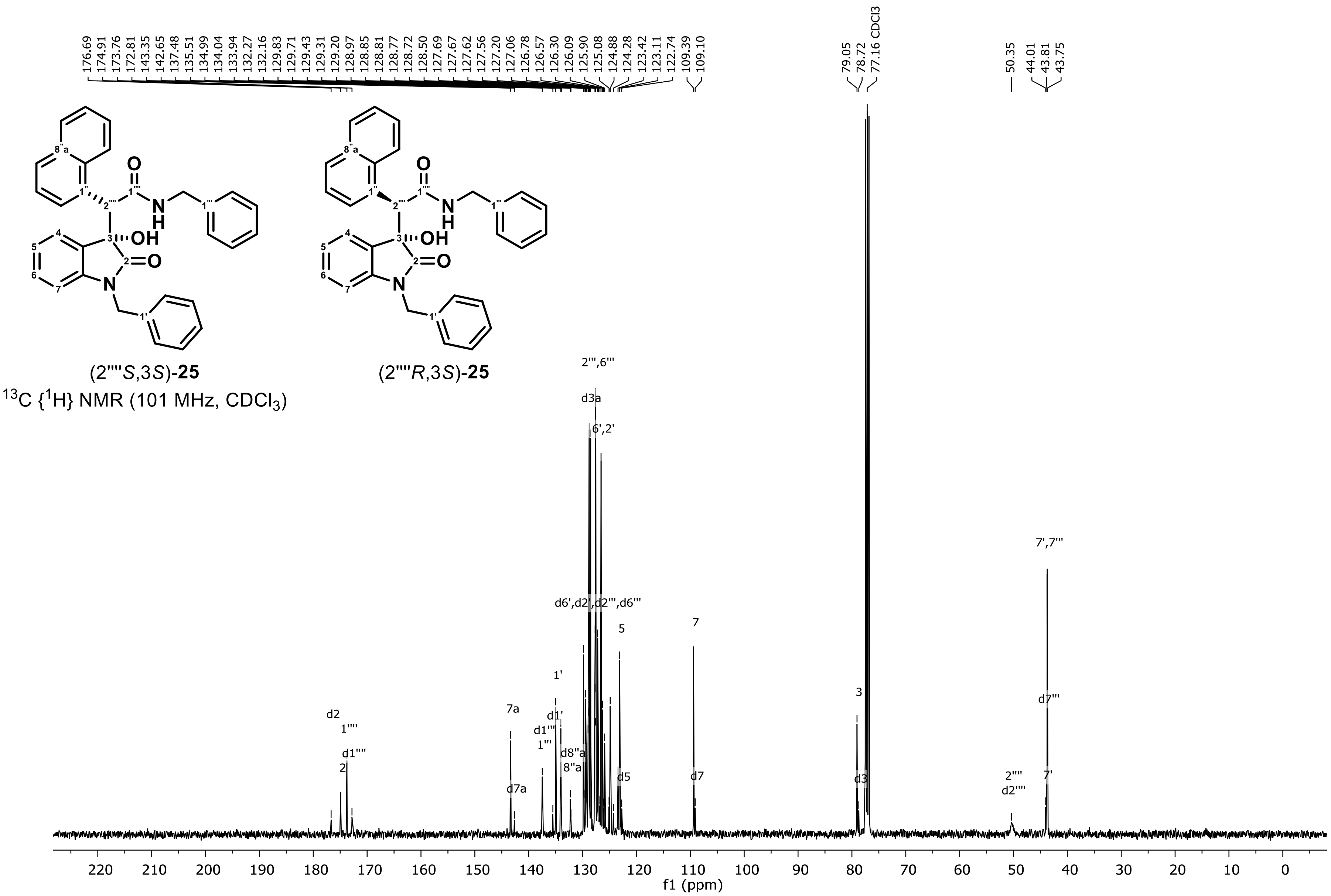
176.69
174.91
173.76
172.81
143.35
142.65
137.48
135.51
134.99
134.04
133.94
132.27
132.16
129.83
129.71
129.43
129.31
129.20
128.97
128.85
128.81
128.77
128.72
128.50
127.69
127.67
127.62
127.56
127.20
127.06
126.78
126.57
126.30
126.09
125.90
125.08
124.88
124.28
123.42
123.11
122.74
109.39
109.10
79.05
78.72
77.16 CDCl3
50.35
44.01
43.81
43.75
(2''''S,3S)-25
(2''''R,3S)-25
^{13}C {^{1}H} NMR (101 MHz, CDCl$_3$)
2''',6'''
d3a
6',2'
d6',d2',d2''',d6'''
5
7
1'
d1'
d1''''
1'''
d8''a
8''a
d5
d7
7a
d7a
d2
1''''
d1''''
2
3
d3
2''''
d2''''
7',7'''
d7'''
7'
f1 (ppm)

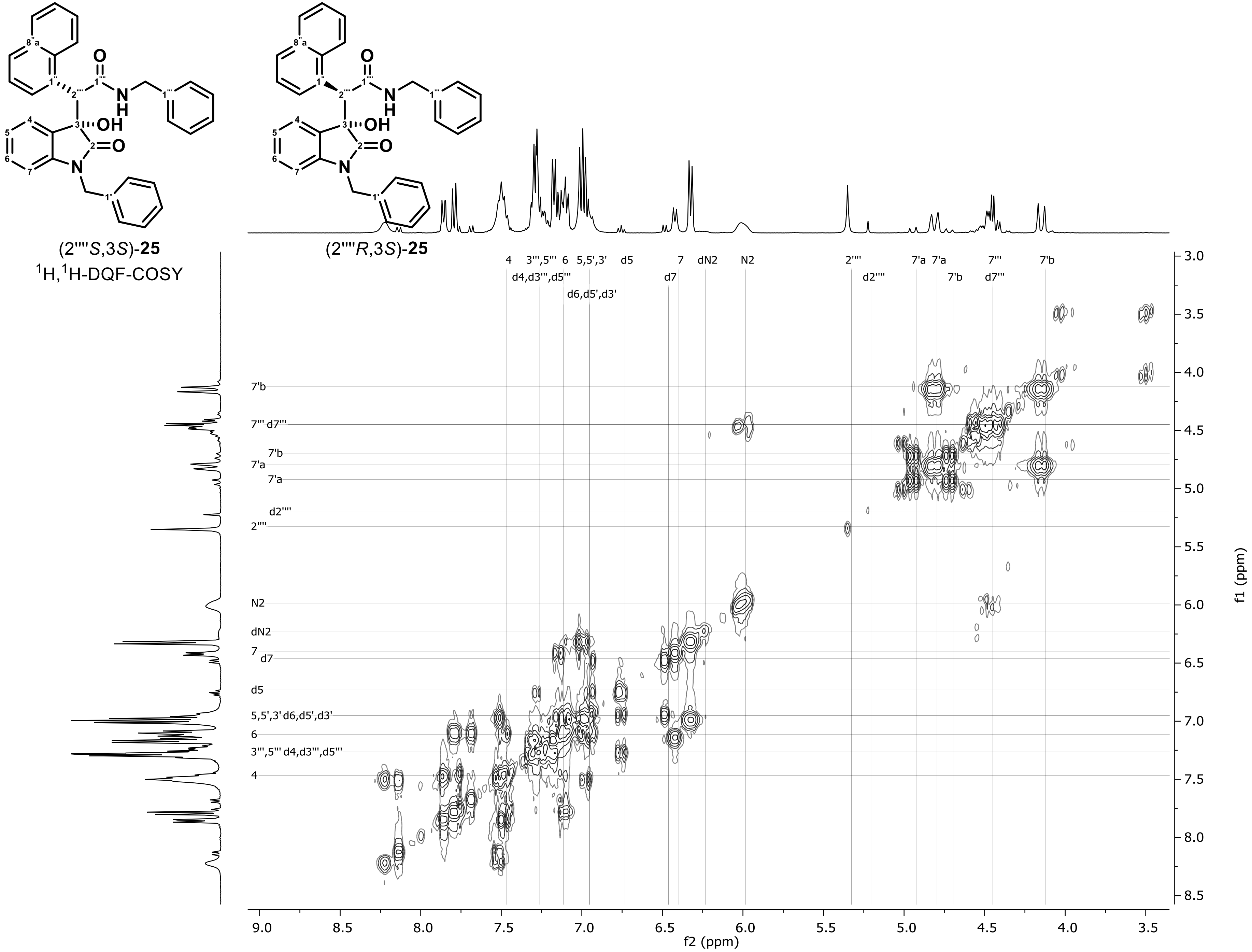

(2''''*S*,3*S*)-**25**

^{1}H,^{1}H-DQF-COSY

(2''''*R*,3*S*)-**25**

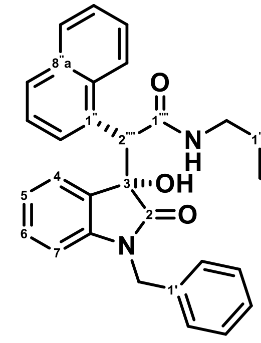

(2''''S,3S)-**25**

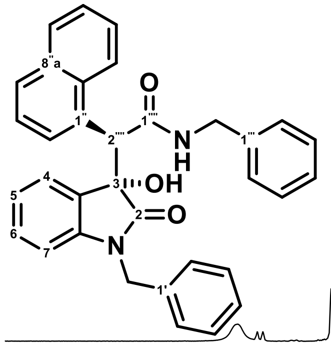

(2''''R,3S)-**25**

^{1}H,^{13}C-gs-HSQC w/ME

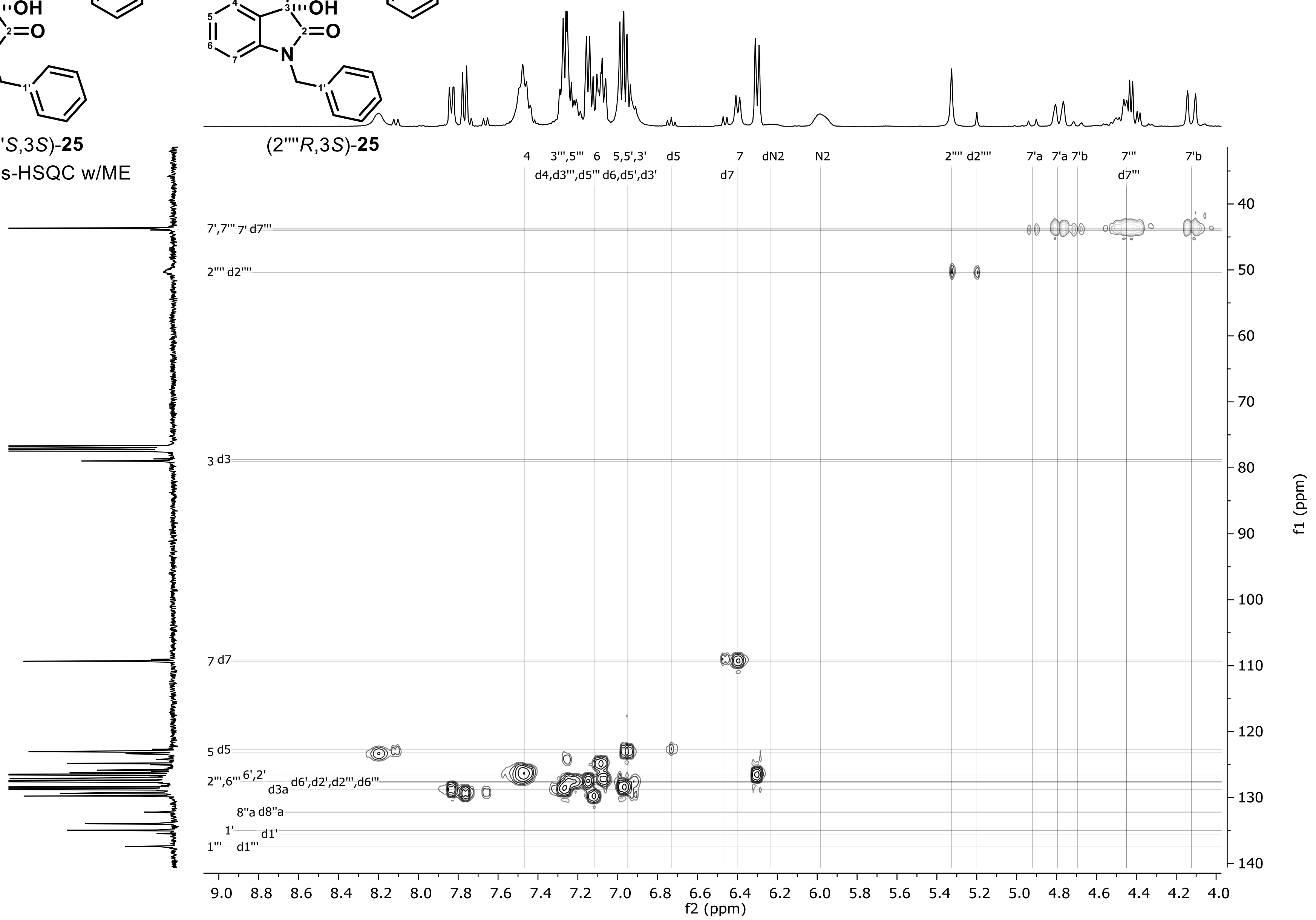

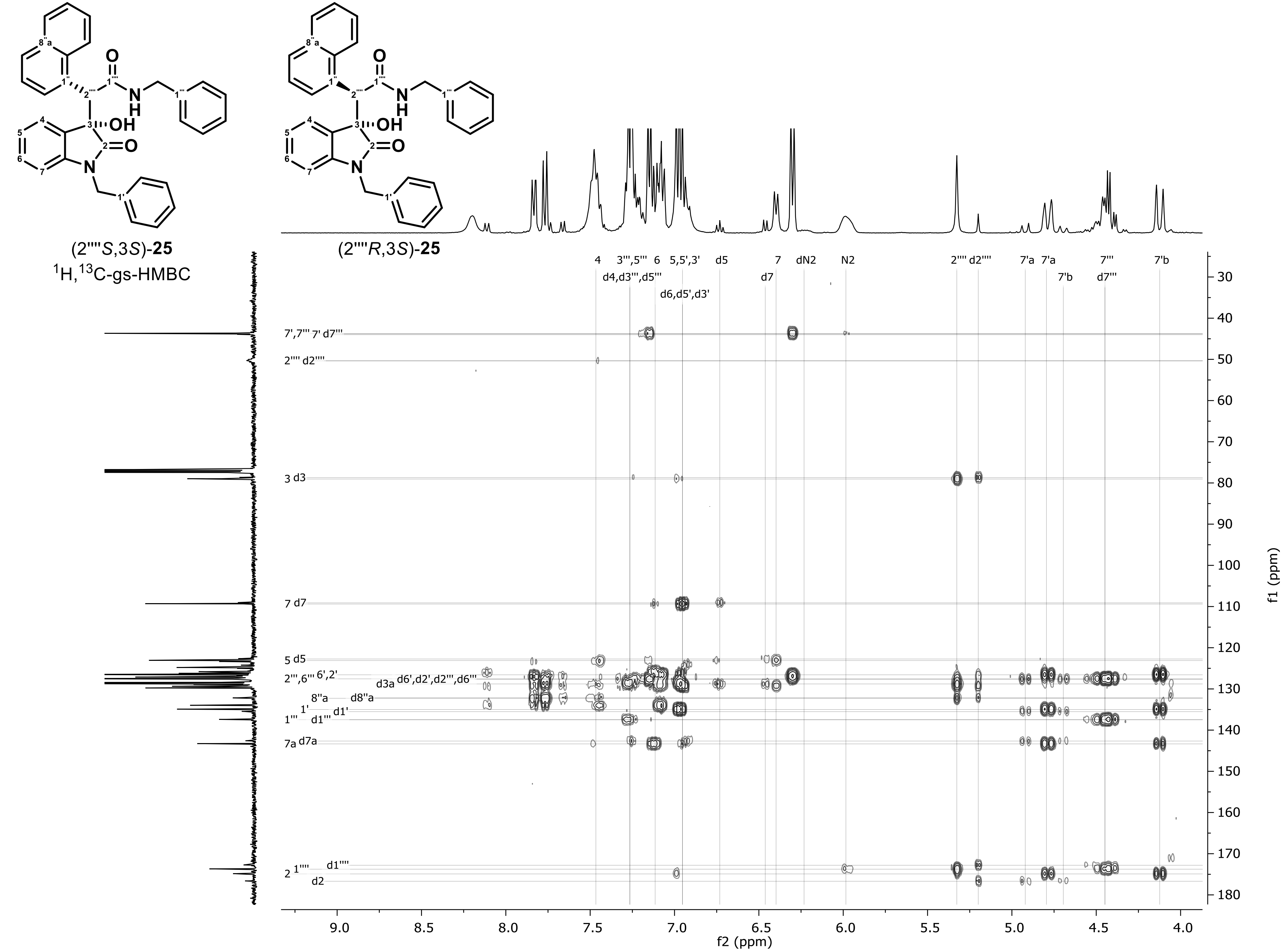

(2''''S,3S)-25

$^{1}H,^{13}C$-gs-HMBC

(2''''R,3S)-25

t) ***N*-Benzyl 2-(1-benzyl-3-hydroxyoxindol-3-yl)-2-thiophen-3-ylacetamide (26)**

To a 25 ml round bottomed flask was added thiophen3-ylacetic anhydride (99.9 mg, 0.400 mmol), *N*-benzyl isatin (59.3 mg, 0.250 mmol), and (2*S*,3*R*)-HyperBTM (3.9 mg, 0.012 mmol). The mixture was cooled to 0 °C and CH_2Cl_2 (6.0 ml, 0.04 M) and Hünig's base (54 µl, 0.312 mmol) were added. The mixture was stirred at 0 °C for 3 h. Benzylamine (82 µl, 0.750 mmol) was added and the reaction was left to be stirred overnight at room temperature. 1,3,5-trimethoxybenzene (0.1 M soln in CH_2Cl_2 500 µl, 0.05 mmol) was added and the solvent was removed under reduced pressure. Purification by column chromatography (Hexane:EtOAc 9:1 → 1:1) gave the title compound as yellow oil (single diastereomer, 108.3 mg, 0.231 mmol, 93%).

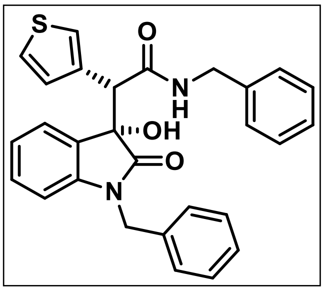

(2′′′′*S*,3*S*)-**26**: **R*f*** 0.41 (Hexane:EtOAc 1:1), purple (vanillin, heat); **Chiral HPLC** analysis (Chiralcel® IB 95:5 Hexane : IPA, flow rate 2.0 ml·min^{-1}, 211 nm, 40 °C) t_R (2′′′′*R*,3*R*)-**26**: 22.3 min, t_R (2′′′′*S*,3*S*)-**26**, 31.1 <1:99 e.r.; $\boldsymbol{\alpha_D^{20}} = -18.5$ (*c* 2.1, $CHCl_3$); $\boldsymbol{\nu_{max}}$ (film) 3339 (m, C-H), 3088 (s, C-H), 3061 (s, C-H), 3030 (s, C-H), 2920 (s, C-H), 2247 (s), 1713 (s, C=O, isatin), 1668 (w), 1647 (s, C=O, amide), 1612 (s), 1539 (w), 1524 (m), 1495 (m), 1489 (m), 1468 (s), 1454 (m), 1433 (w), 1532 (s), 1300 (w), 1236 (w), 1219 (w), 1175 (m), 1157 (w), 1117 (w), 1098 (w), 1080 (w), 1030 (w), 1005 (w), 974 (w), 908 (s, C(sp^2)-H), 841 (m), 752(s); **^{1}H NMR** (400 MHz, $CDCl_{3c}$) δ_H 7.43 (1H, dd, $^3J_{HH}$ = 7.5 Hz, $^4J_{HH}$ = 1.2 Hz, ArC^4*H*), 7.35 – 7.25 (3H, m, PhC$^{3''',4''',5'''}$*H*), 7.25 – 7.20 (2H, m, PhC$^{2''',6'''}$*H*), 7.20 – 7.12 (4H, m, ArC^6*H*, PhC$^{3',4',5'}$*H*), 7.10 (1H, dd, $^3J_{HH}$ = 5.0 Hz, $^4J_{HH}$ = 3.0 Hz, ArC$^{4''}$*H*), 6.98 (1H, app td, $^3J_{HH}$ = 7.6 Hz, $^4J_{HH}$ = 1.0 Hz, ArC^5*H*), 6.90 (1H, dd, $^4J_{HH}$ = 3.0 Hz, 1.3 Hz, ArC$^{2''}$*H*), 6.86 (1H, s (br), O*H*), 6.71 – 6.64 (2H, PhC$^{2',6'}$*H*), 6.47 (1H, d, $^3J_{HH}$ = 7.8 Hz, ArC^7*H*), 6.42 (1H, app, dt, $^3J_{HH}$ = 5.0 Hz, $^4J_{HH}$ = 1.1 Hz, ArC$^{5''}$*H*), 6.27 – 6.17 (1H, m, N*H*), 4.89 (1H, d, $^2J_{HH}$ = 15.8 Hz, NC*H*$_a$H$_b$-Ph), 4.55 (1H, dd, $^2J_{HH}$ = 15.0 Hz, $^3J_{HH}$ = 6.1 Hz, NHC*H*$_a$H$_b$-Ph), 4.54 (1H, s, ArC$^{3''}$C*H*), 4.44 (1H, dd, $^2J_{HH}$ = 15.0 Hz, $^3J_{HH}$ = 5.7 Hz, NHCH$_a$*H*$_b$-Ph), 4.32 (1H, d, $^2J_{HH}$ = 15.8 Hz, NCH$_a$*H*$_b$-Ph); **^{13}C {^{1}H} NMR** (101 MHz, $CDCl_3$) δ_C 174.9 (*C*(O)NBn), 172.8 (*C*(O)NHBn), 143.3 (Ar*C*^{7a}), 137.5 (Ph*C*$^{1'''}$), 135.1 (Ph*C*$^{1'}$), 132.9 (Ar*C*$^{3''}$), 130.0 (Ar*C*^{6}H), 129.1 (Ar*C*^{3a}), 128.8 and 128.7 (Ph*C*$^{3',5'}$H and Ph*C*$^{3''',5'''}$H), 128.4 (Ar*C*$^{5''}$H), 127.6 (Ph*C*$^{2''',6'''}$H), 127.8 and 127.4 (Ph*C*$^{4'}$H and Ph*C*$^{4'''}$H), 126.8 (Ph*C*$^{2',6'}$H), 126.6 (Ar*C*$^{2''}$H), 126.5 (Ar*C*$^{4''}$H), 125.4 (Ar*C*^{4}H), 123.2 (Ar*C*^{5}H), 109.6 (Ar*C*^{7}H), 78.5 (*C*-OH), 51.9 (ArC$^{3''}$*C*H), 43.8$_4$ and 43.7$_9$ (N*C*H$_2$Ph and NH*C*H$_2$Ph); ***m/z*** (ESI$^+$) 163 (12%), 196 (4%), 260 (4%), 296 (4%), 318 ([M-BnNHCOO]$^+$ 40%), 469 ([M+H]$^+$ 6%), 491 ([M+Na]$^+$ 100%), 492 ([M(^{13}C)+Na]$^+$ 31%), 493 ([M($^{13}C_2$)+Na]$^+$ 4%), 507 ([M+K]$^+$ 11%), 722 (5%), 959 ([2M+Na]$^+$ 61%); **HRMS** (ESI$^+$) *m/z* calcd for [M+Na]$^+$ $C_{28}H_{24}O_3N_2NaS$ 491.1389, found 491.1389 (−2.2 ppm).

(±)-anti-**26**

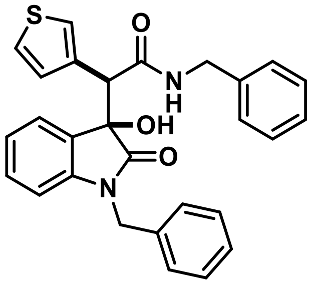

PDA Ch1 211nm

Peak#	Ret. Time	Area%
1	22.280	49.288
2	31.426	50.712
Total		100.000

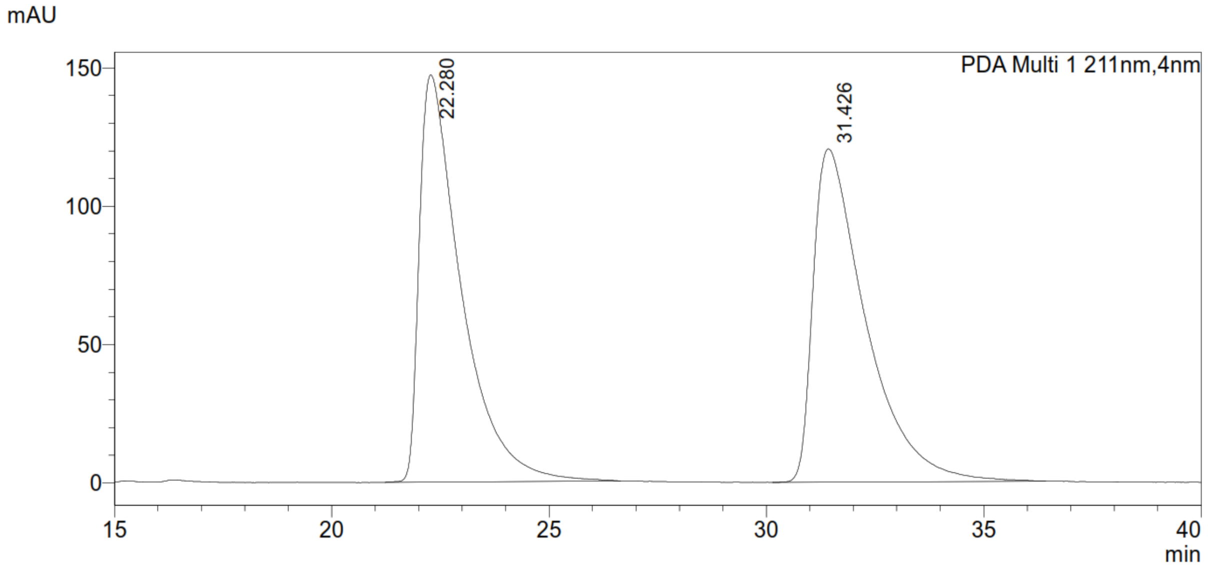

(–)-(2'''*S*,3*R*)-**26**

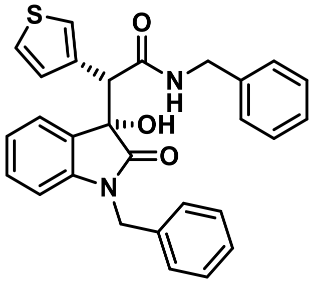

PDA Ch1 211nm

Peak#	Ret. Time	Area%
1	22.425	-0.008
2	31.103	100.008
Total		100.000

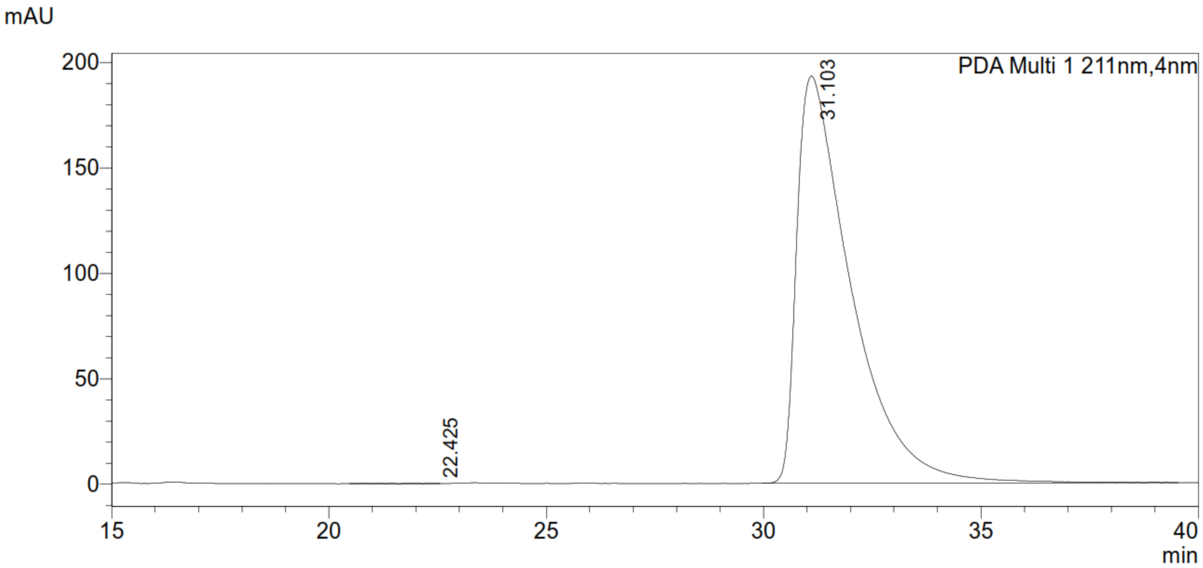

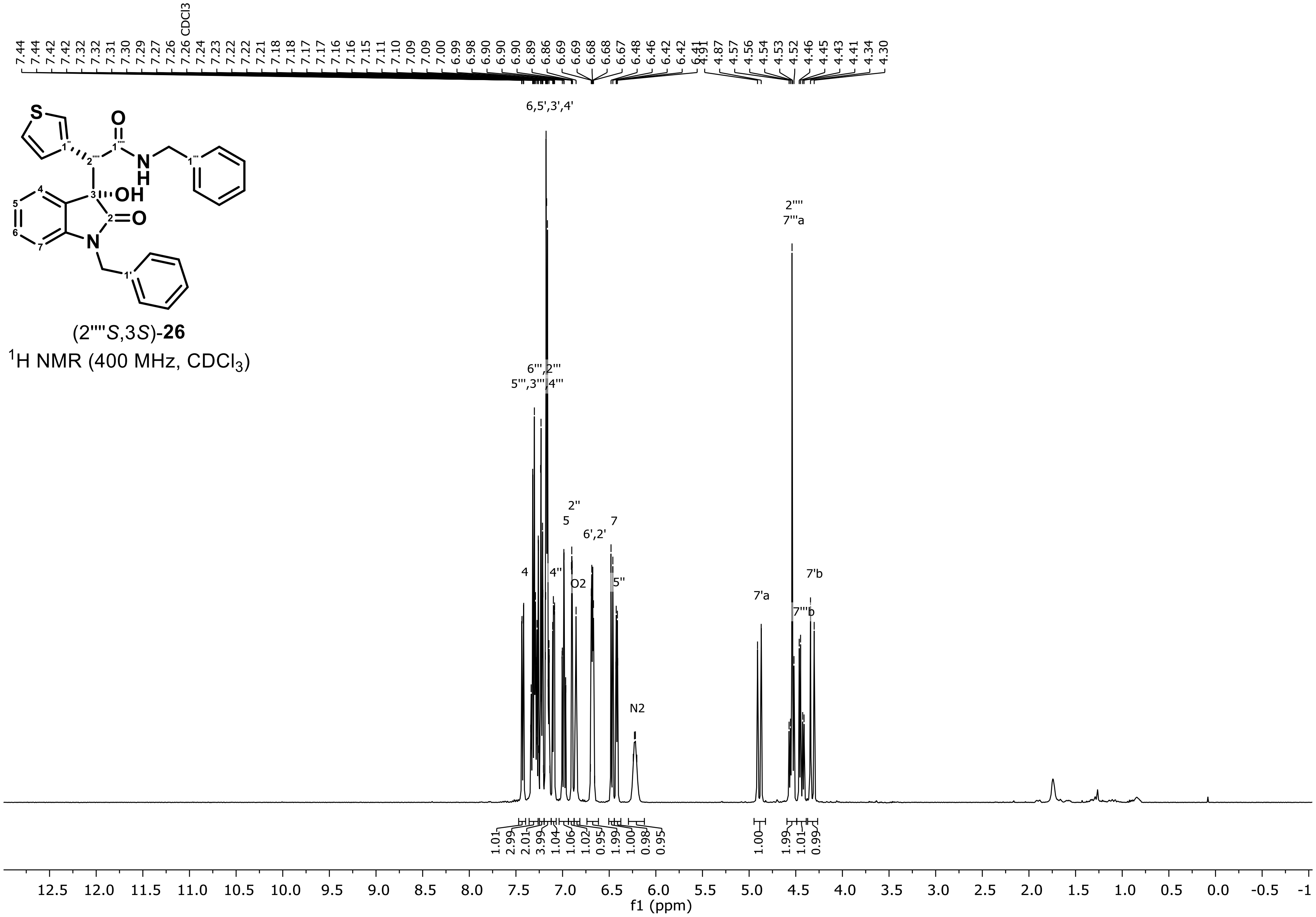

(2''''*S*,3*S*)-**26**

^1H NMR (400 MHz, $CDCl_3$)

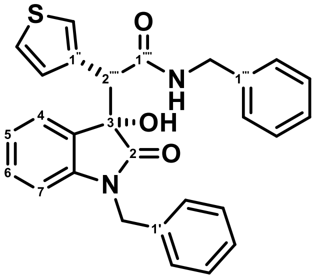

(2''''*S*,3*S*)-**26**

$^{13}C\{^1H\}$ NMR (101 MHz, $CDCl_3$)

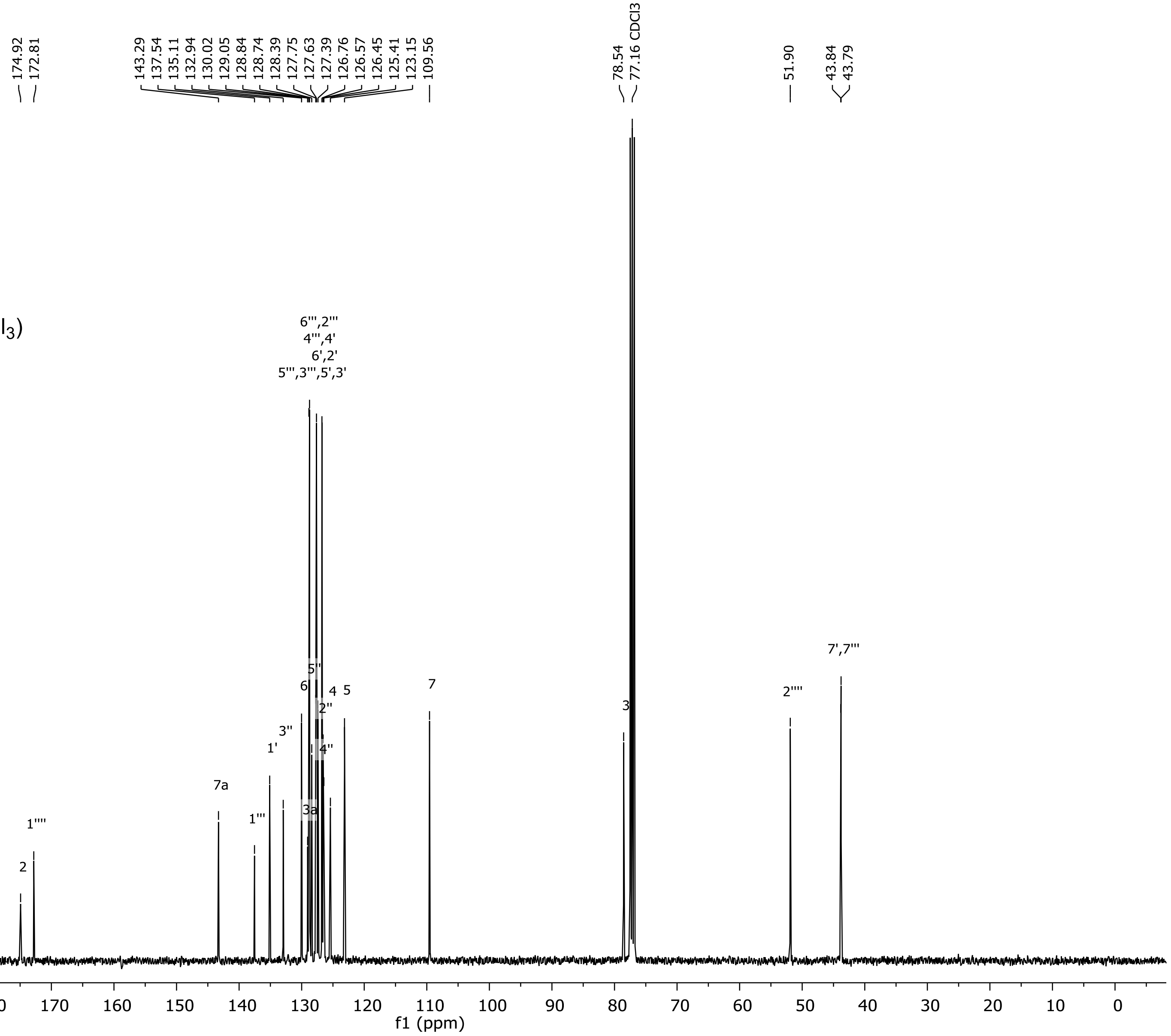

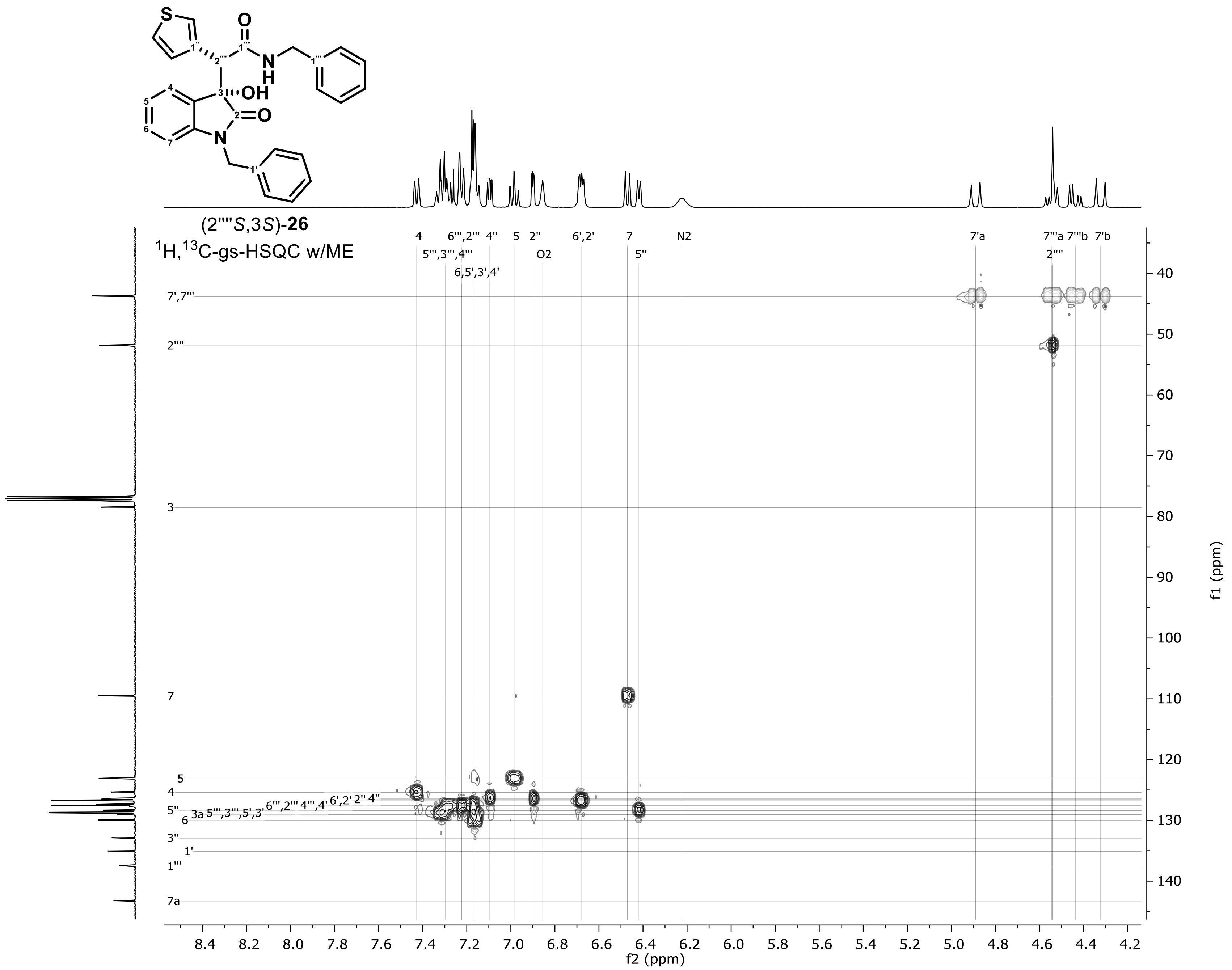

(2''''*S*,3*S*)-**26**

$^{1}H,^{13}C$-gs-HSQC w/ME

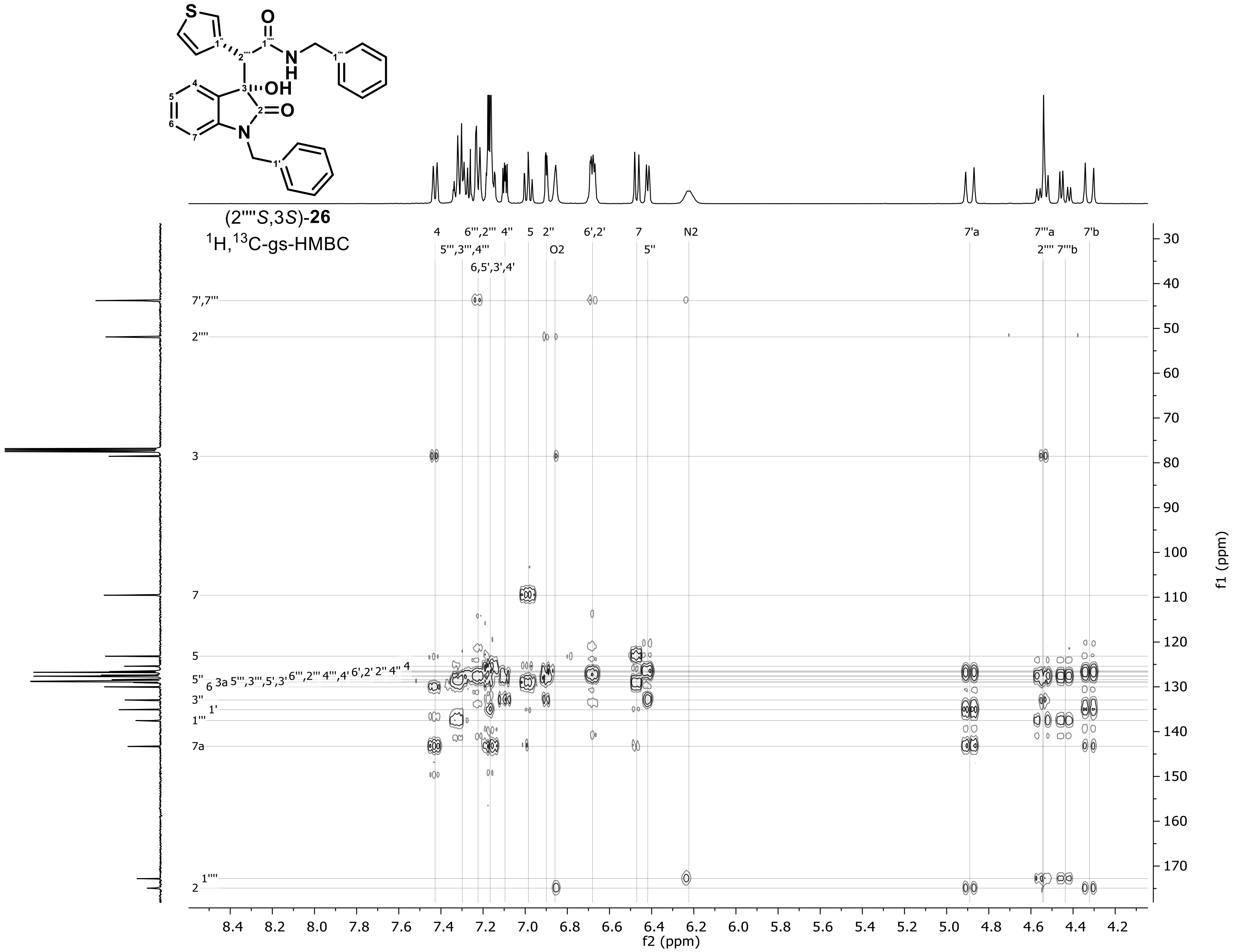

(2''''*S*,3*S*)-**26**
^{1}H,^{13}C-gs-HMBC

u) ***N*-Benzyl 2-(1-benzyl-3-hydroxyoxindol-3-yl)-5-methylhex-3-enamide (27)**

To a 25 ml round bottomed flask was added (*E*)-5-methylhexen-3-enoic anhydride (89.4 mg, 0.400 mmol), *N*-benzyl isatin (59.3 mg, 0.250 mmol), and (2*S*,3*R*)-HyperBTM (3.9 mg, 0.012 mmol). The mixture was cooled to 0 °C and CH_2Cl_2 (6.0 ml, 0.04 M) and Hünig's base (54 µl, 0.312 mmol) were added. The mixture was stirred at 0 °C for 3 h. Benzylamine (82 µl, 0.750 mmol) was added and the reaction was left to be stirred overnight at room temperature. 1,3,5-trimethoxybenzene (0.1 M soln in CH_2Cl_2 500 µl, 0.05 mmol) was added and the solvent was removed under reduced pressure. Purification by column chromatography (Hexane:EtOAc 9:1 → 3:2) gave the title compound as pale yellow oil (96:4 d.r., 108.2 mg, 0.238 mmol, 95%).

10 mmol scale reaction: To a 25 ml round bottomed flask was added (*E*)-5-methylhexen-3-enoic anhydride (3575.5 mg, 15.000 mmol), *N*-benzyl isatin (2373.0 mg, 10.000 mmol), and (2*S*,3*R*)-HyperBTM (154.3 mg, 0.500 mmol). The mixture was cooled to 0 °C and CH_2Cl_2 (240 ml, 0.04 M) and Hünig's base (2.2 ml, 12.632 mmol) were added. The mixture was stirred at 0 °C for 3 h. Benzylamine (3.3 ml, 30.216 mmol) was added, and the reaction was left to be stirred overnight at room temperature. The solvent was removed under reduced pressure. Purification by column chromatography (Hexane:EtOAc 4:1 → 1:4) gave the title compound as pale yellow oil (96:4 d.r., 4126.5 mg, 9.078 mmol, 91%).

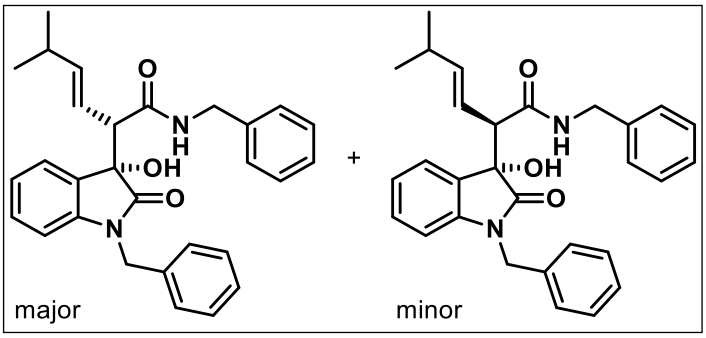

Major (2'''*S*,3*S*)-**27**; **minor** (2'''*R*,3*S*)-**27** not resolved; analysed as 96:4 mixture of diastereomers: **m.p.** 79 °C (rac); $\mathbf{R_f}$ 0.55 (Hexane:EtOAc 1:1), green → purple (vanillin, heat); **Chiral HPLC** analysis (Chiralcel® IB 95:5 Hexane : IPA, flow rate 2.0 ml·min^{-1}, 211 nm, 40 °C) t_R (2'''*R*,3*R*)-**27**: 13.2 min, t_R (2'''*S*,3*S*)-**27**, 16.9 min, <1:99 e.r.; α_D^{20} = −35.5 (*c* 5.2, $CHCl_3$); ν_{max} **(film)** 3339 (m, C-H), 3088 (w, C-H), 3063 (w, C-H), 3032 (w, C-H), 2959 (m, C-H), 2928 (w, C-H), 2868 (w, C-H), 2247 (w), 1717 (s, C=O, isatin), 1645 (m, C=O, amide), 1614 (s), 1539 (m), 1526 (m), 1497 (m), 1489 (m), 1468 (s), 1454 (m), 1435 (w), 1362 (s), 1350 (s), 1298 (w), 1242 (w), 1209 (w), 1175 (s), 1109 (m), 1078 (m), 1030 (m), 1009 (w), 976 (m), 907 (s, C(sp^2)-H); ^{1}**H NMR** (400 MHz, $CDCl_3$) δ_H 7.43 (1H, dd, $^3J_{HH}$ = 7.5 Hz, $^4J_{HH}$ = 1.2 Hz, ArC^4*H*), 7.38 – 7.23 (10H, m, PhC$^{2',3',4',5',6'}$*H*, PhC$^{2'',3'',4'',5'',6''}$*H*), 7.20 (1H, ddd, $^3J_{HH}$ = 7.8 Hz, 7.7 Hz, $^4J_{HH}$ = 1.2 Hz, ArC^6*H*), 6.99 (1H, ddd, $^3J_{HH}$ = 7.8 Hz, 7.5 Hz, $^4J_{HH}$ = 1.0 Hz, ArC^5*H*), 6.67 (1H, d, $^3J_{HH}$ = 7.8 Hz, ArC^7*H*), 6.55 (1H, s (br), N*H*), 5.72 (1H, dd, $^3J_{HHtrans}$ = 15.5 Hz, $^3J_{HH}$ = 6.7 Hz, CH=C*H*^{i}Pr), 4.99 (1H, dd, $^3J_{HHtrans}$ = 15.5 Hz, $^3J_{HH}$ =9.7 Hz, C*H*=CH^{i}Pr), 4.86 (1H, d, $^2J_{HH}$ = 15.7 Hz, NC*H*$_a$H$_b$Ph), 4.73 (1H, d, $^2J_{HH}$ = 15.7 Hz, NCH$_a$*H*$_b$Ph), 4.54 (1H, dd, $^2J_{HH}$ = 15.3 Hz, $^3J_{HH}$ = 5.8 Hz, NHC*H*$_a$H$_b$Ph), 4.50 (1H, dd, $^2J_{HH}$ = 15.3 Hz, $^3J_{HH}$ = 5.8 Hz, NHCH$_a$*H*$_b$Ph), 3.65 (1H, d, $^3J_{HH}$ = 9.7 Hz, C*H*-CH=CH^{i}Pr), 2.18 (1H, heptdd, $^3J_{HH}$ = 6.8 Hz, 6.7 Hz, $^4J_{HH}$ = 1.4 Hz, C*H*(CH_3)$_2$), 0.87 (3H, d, $^3J_{HH}$ = 6.8 Hz, CH(CH_3)$_a$(C*H*$_3$)$_b$), 0.85 (3H, d, $^3J_{HH}$ = 6.8 Hz, CH(C*H*$_3$)$_a$(CH_3)$_b$); ^{13}**C {^{1}H} NMR** (101 MHz, $CDCl_3$) δ_C 175.6 (*C*(O)NBn), 172.7 (*C*(O)NHBn), 147.5 (CH=*C*H^{i}Pr), 142.8 (Ar*C*^{7a}), 137.6 (Ph*C*$^{1''}$), 135.4 (Ph*C*$^{1'}$), 129.7 (Ar*C*^{6}H), 128.7 (Ar*C*^{3a}, Ph*C*$^{3',5'}$H and Ph*C*$^{3'',5''}$H), 127.6$_1$ and 127.5$_8$ (Ph*C*$^{4'}$H and Ph*C*$^{4''}$H), 127.5$_6$ (Ph*C*$^{2'',6''}$H), 127.2 (Ph*C*$^{2',6'}$H), 125.4 (Ar*C*^{4}H), 123.0 (Ar*C*^{5}H), 118.6 (*C*H=CH^{i}Pr), 109.3 (Ar*C*^{7}H), 77.9 (*C*-OH), 54.0 (*C*HCH=CH^{i}Pr), 43.8 (N*C*H$_2$Ph), 43.6 (NH*C*H$_2$Ph), 31.1 (*C*H(CH_3)$_2$), 22.1 (CH(CH_3)$_a$(*C*H$_3$)$_b$), 21.9 (CH(*C*H$_3$)$_a$(CH_3)$_b$); ***m/z*** (ESI$^+$) 163 ([$C_9H_{11}N_2O$]$^+$ 3%), 309 ([M-$C_{10}H_{10}O$]$^+$ 100%), 310 ([M(^{13}C)-$C_{10}H_{10}O$]$^+$ 21%), 311 ([M($^{13}C_2$)-$C_{10}H_{10}O$]$^+$ 5%), 477 ([M+Na]$^+$ 21%), 931 ([2M+Na]$^+$ 32%); ***m/z*** (ESI$^-$) 216 (21%), 302 ([M-BnNHCOOH-H]$^-$ 5%), 435 ([M-H_2O]$^-$ 100%), 436 ([M(^{13}C)-H_2O]$^-$ 34%), 437 ([M($^{13}C_2$)-H_2O]$^-$ 5%), 907 ([2M-H]$^-$ 3%); **HRMS** (ESI$^+$) *m/z* calcd for [M+Na]$^+$ $C_{29}H_{30}O_3N_2Na$ 477.2149, found 477.2137 (−2.4 ppm).

(±)-anti-**27**

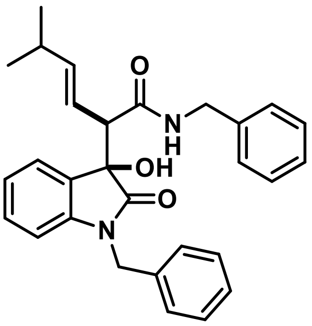

PDA Ch1 211nm

Peak#	Ret. Time	Area%
1	13.214	48.958
2	17.444	51.042
Total		100.000

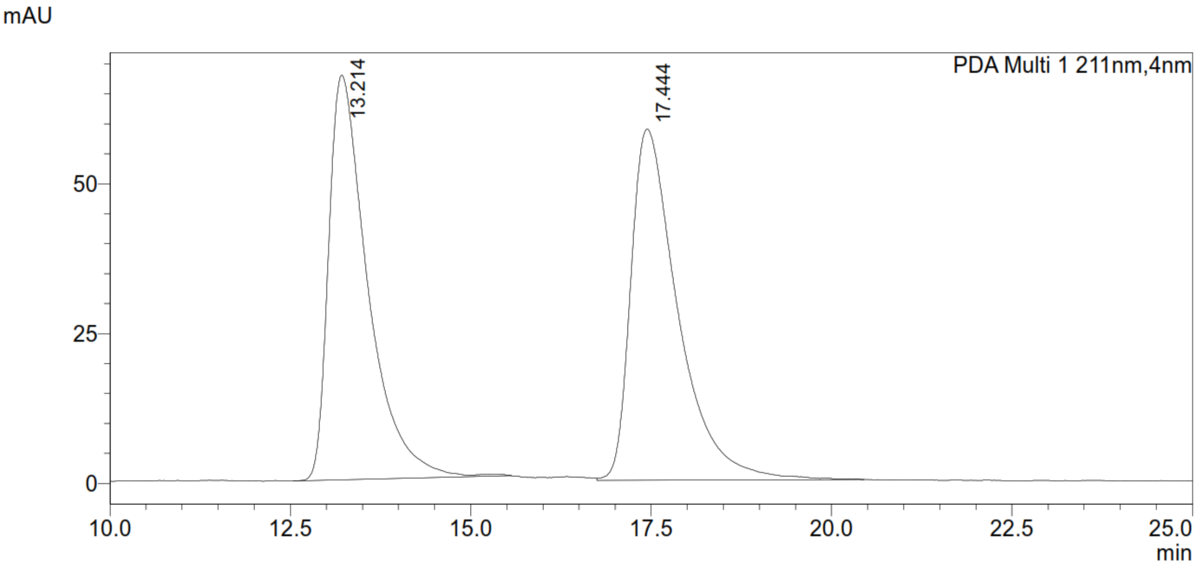

(–)-(2'''*S*,3*S*)-**27**

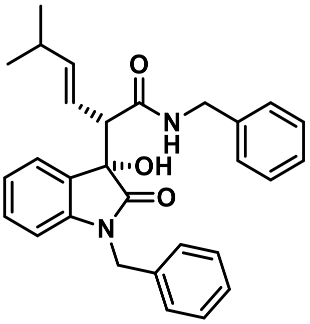

PDA Ch1 211nm

Peak#	Ret. Time	Area%
1	13.187	0.333
2	16.852	99.667
Total		100.000

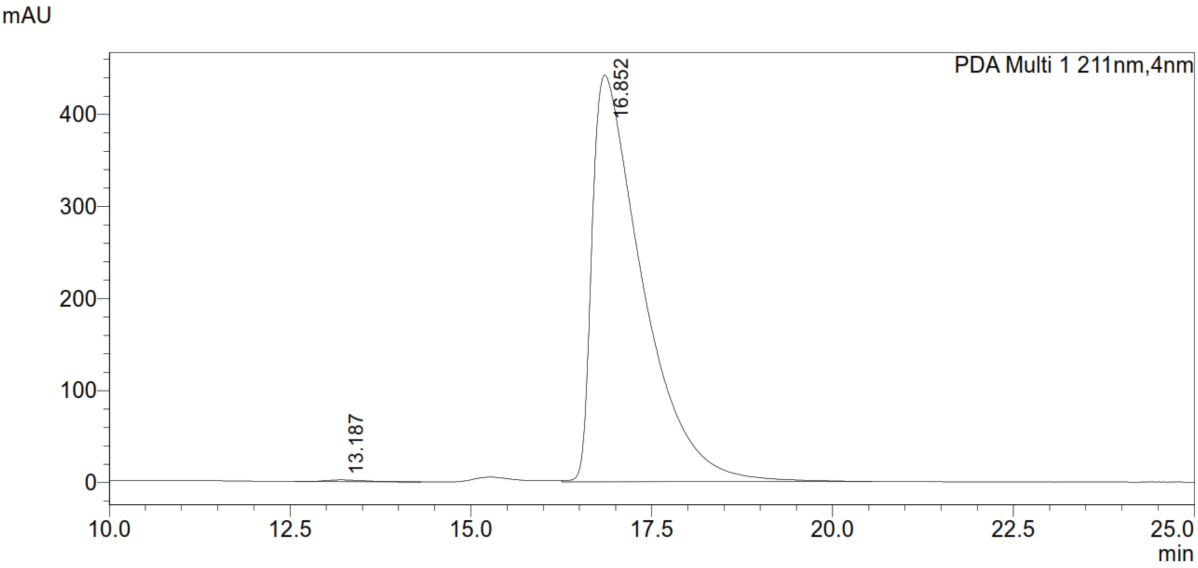

(–)-(2'''*S*,3*S*)-**27** (10 mmol scale)

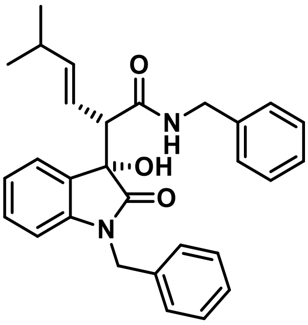

PDA Ch1 211nm

Peak#	Ret. Time	Area%
1	13.483	0.320
2	17.416	99.680
Total		100.000

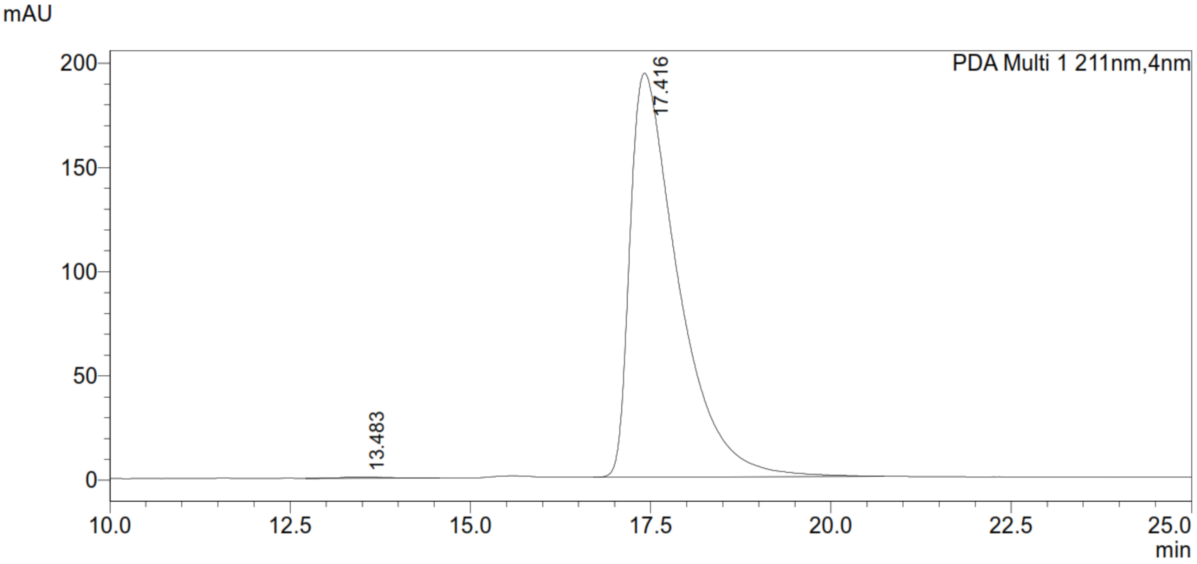

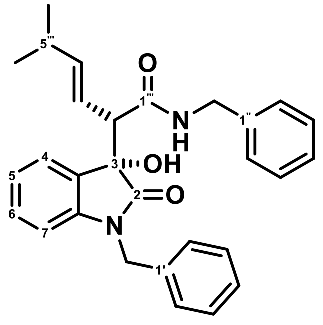

(2'''*S*,3*S*)-**27**

^{1}H NMR (400 MHz, $CDCl_3$)

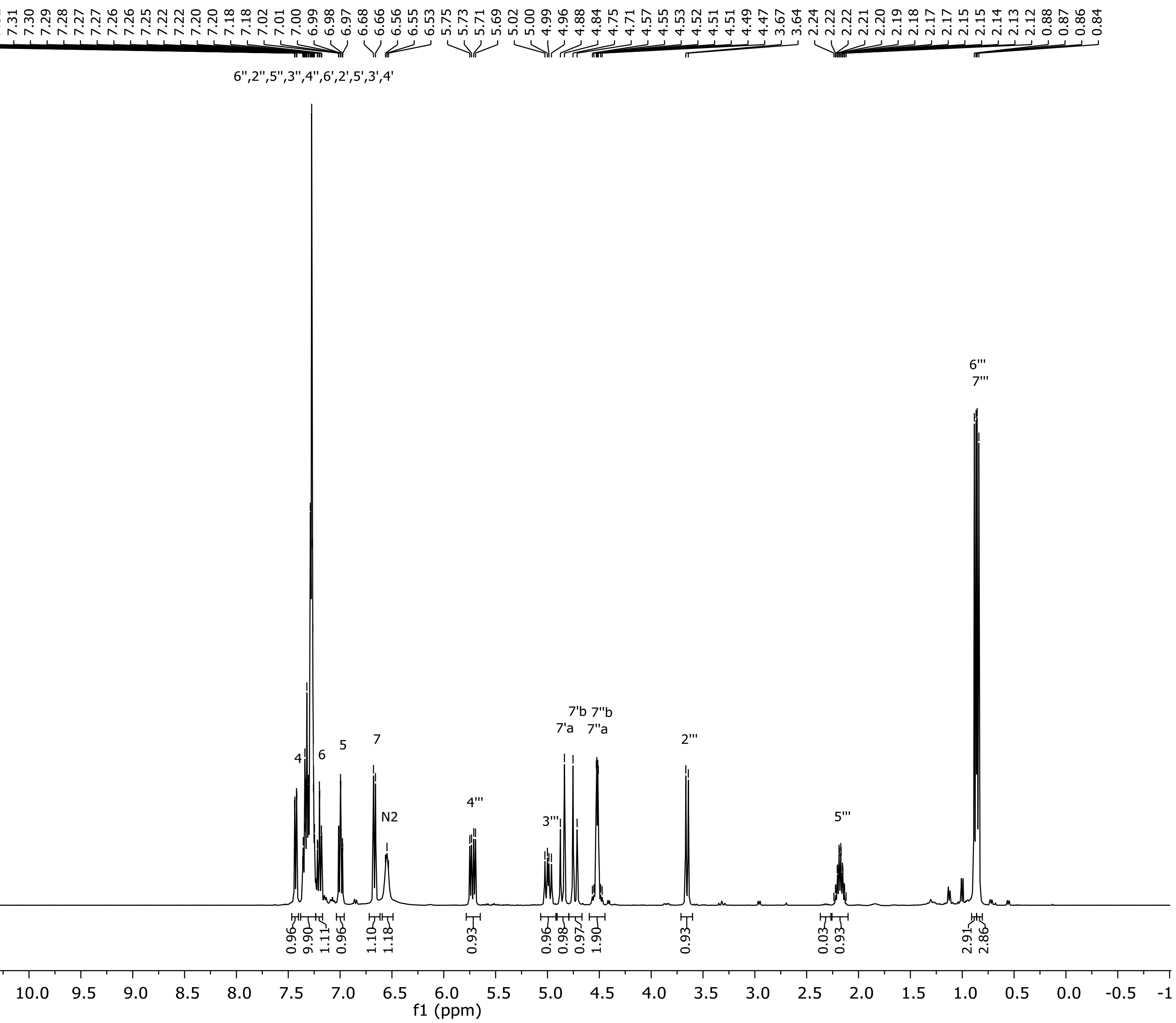

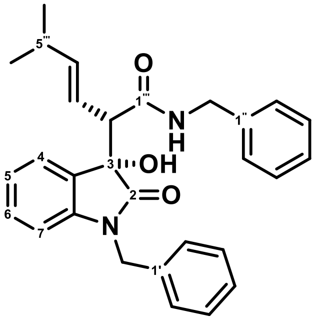

(2'''*S*,3*S*)-**27**

^{13}C {^{1}H} NMR (101 MHz, $CDCl_3$)

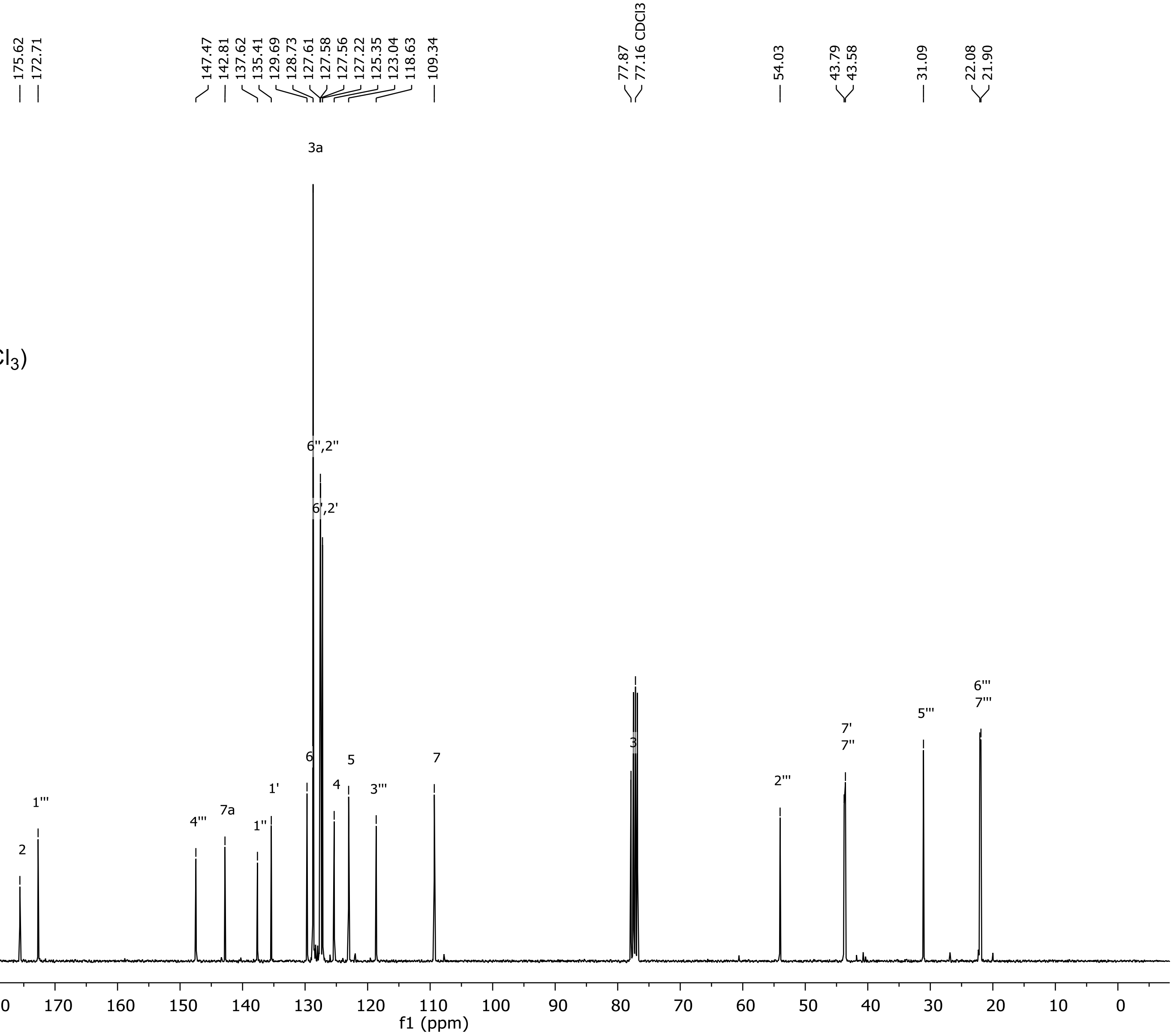

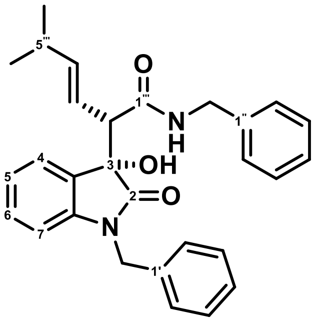

(2'''*S*,3*S*)-**27**

$^{1}H,^{13}C$-gs-HSQC w/ME

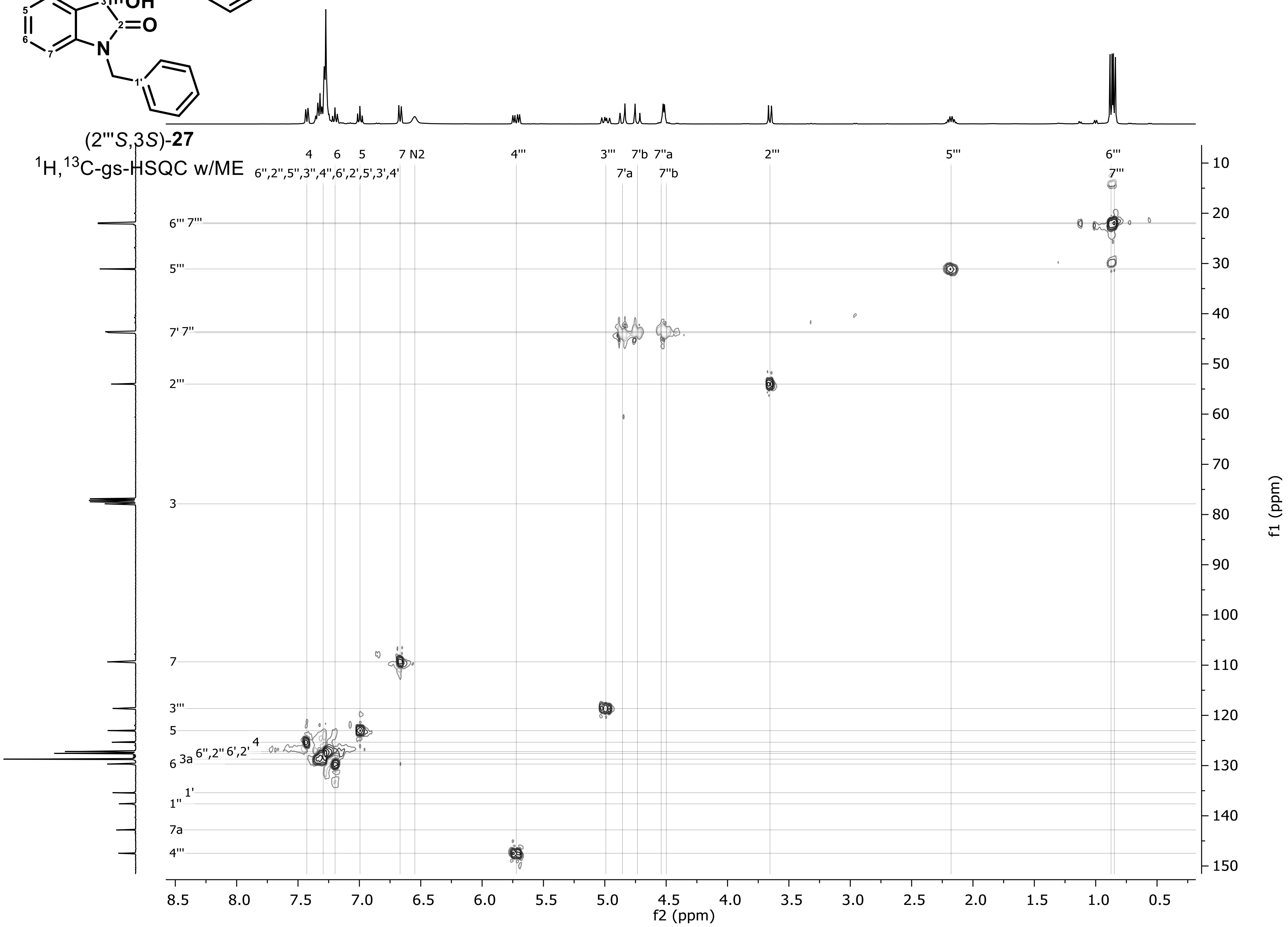

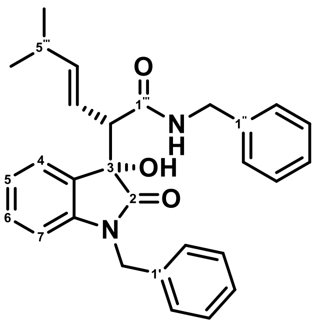

(2'''*S*,3*S*)-**27**

$^{1}H,^{13}C$-gs-HMBC

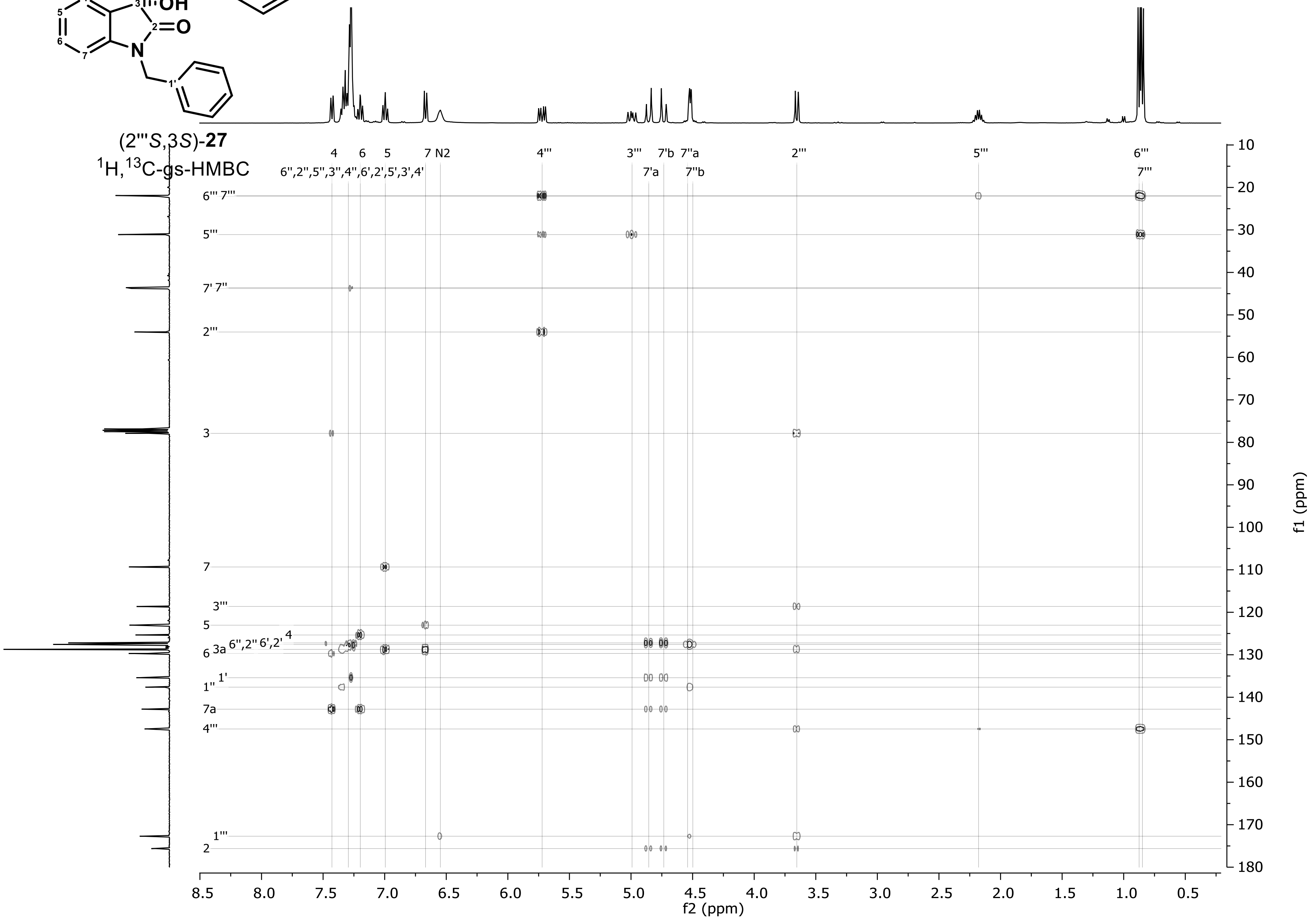

v) 1-Benzyl-7-chloro-3-hydroxy-3-(2-morpholino-2-oxo-1-phenylethyl)indolin-2-one (28)

To a 25 ml round bottomed flask was added phenylacetic anhydride (95.3 mg, 0.375 mmol), *N*-benzyl-7-chloroisatin (68.0 mg, 0.250 mmol), and (2*S*,3*R*)-HyperBTM (3.9 mg, 0.012 mmol). The mixture was cooled to 0 °C and CH_2Cl_2 (6.0 ml, 0.04 M) and Hünig's base (54 µl, 0.312 mmol) were added. The mixture was stirred at 0 °C for 3 h. Morpholine (65 µl, 0.750 mmol) was added and the reaction was left to be stirred overnight at room temperature. 1,3,5-trimethoxybenzene (0.1 M soln in CH_2Cl_2 500 µl, 0.05 mmol) was added and the solvent was removed under reduced pressure. Purification by column chromatography (Hexane : EtOAc 6:4 → CH_2Cl_2:EtOAc 1:1) gave the title compound as mixture of diastereomers and colourless semi-solid (87:13 d.r., 116.1 mg, 0.248 mmol 99%)

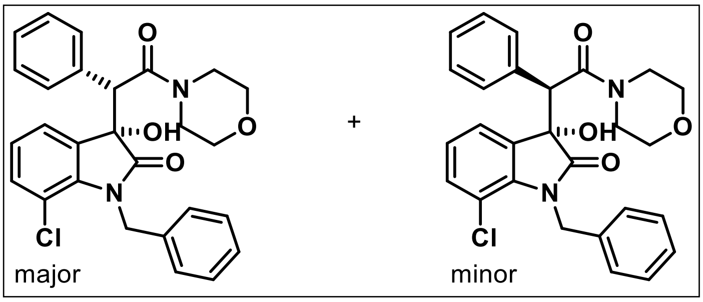

Major (2''''*S*,3*S*)-**28** and **minor** (2''''*R*,3*S*)-**28** analysed as 86:14 mixture of diastereomers: **m.p.** 176 – 178 °C (rac), 152 – 153 °C (ee); **R*f*** 0.34 (Hexane:EtOAc 4:6), **R*f*** 0.41 (CH_2Cl_2:EtOAc 7:3); $\alpha_D^{20} = -135.1$ (*c* 2.05, $CHCl_3$); **Chiral HPLC analysis** (Chiralcel® AD-H 85:15 Hexane:IPA, flow rate 2.0 ml·min^{-1}, 211 nm, 40 °C) t_R (2''''*R*,3*S*)-**28**: 10.5 min, t_R (2''''*S*,3*R*)-**28**: 34.9 min, >99:1 e.r.; t_R (2''''*S*,3*S*)-**28**: 14.5 min, t_R (2''''*R*,3*R*)-**28**: 19.1 min, >99:1 e.r.;); ***ν*max (film)**: 3397 (s, br), 3063 (w), 3030 (w), 2965 (w), 2922 (w), 2857 (w), 1721 (s, C=O, amide), 1624 (s, C=O, lactam), 1607 (s), 1584 (w), 1497 (w), 1450 (s), 1356 (m), 1302 (w), 1267 (w), 1227 (m), 1167 (m), 1134 (s), 1113 (s), 1069 (m), 1022 (m), 970 (w), 908 (s), 862 (w), 843 (w), 818 (w), 787 (w); **^{1}H NMR** (400 MHz, $CDCl_3$) δ_H 7.42 – 7.09 (9.28H, m, PhC*H*), 7.07 (1H, dd, $^3J_{HH}$ = 8.2 Hz, $^4J_{HH}$ = 1.2 Hz, ArC^6*H*), 7.06 – 7.01 (1.72H, m, PhC$^{2',6'}$H), 6.82 (0.14H, dd, $^3J_{HH}$ = 8.2 Hz, 7.4 Hz, ArC^5*H*), 6.81 (0.86H, dd, $^3J_{HH}$ = 8.2 Hz, 7.5 Hz, ArC^5*H*), 6.55 (0.14H, dd, $^3J_{HH}$ = 7.4 Hz, $^4J_{HH}$ = 1.2 Hz, ArC^4*H*), 6.40 (0.86H, s (br), O*H*), 5.30 (0.86H, d, $^2J_{HH}$ = 16.2 Hz, NC*H*$_a$H$_b$-Ph), 5.28 (0.14H, d, $^2J_{HH}$ = 16.3 Hz, NC*H*$_a$H$_b$-Ph), 5.22 (0.14H, d, $^2J_{HH}$ = 16.4 Hz, NCH$_a$*H*$_b$-Ph), 5.21 (0.86H, d, $^2J_{HH}$ = 16.2 Hz, NCH$_a$*H*$_b$-Ph), 4.64 (0.14H, s(br), O*H*), 4.43 (0.14H, s, C*H*-Ph), 4.19 (0.86H, s, C*H*-Ph), 3.77 – 3.61 (2.72H, m, NC*H*$_c$*H*$_d$C*H*$_c$H$_d$O, major, NC*H*$_c$H$_d$CH$_c$H$_d$O, minor), 3.61 – 3.50 (1H, m, NCH$_c$H$_d$CH$_c$*H*$_d$O, major, NCH$_c$H$_d$C*H*$_c$H$_d$O, minor), 3.40 (0.86H, ddd, $^2J_{HH}$ = 11.4 Hz, $^3J_{HH}$ = 6.2 Hz, 3.0 Hz, NCH$_a$H$_b$C*H*$_a$H$_b$O), 3.50 – 3.35 (0.42H, m, NCH$_a$H$_b$C*H*$_a$H$_b$O, NCH$_c$*H*$_d$CH$_c$*H*$_d$O), 3.31 (0.86H, ddd, $^2J_{HH}$ = 13.4 Hz, $^3J_{HH}$ = 6.9 Hz, 3.0 Hz, NC*H*$_a$H$_b$CH$_a$H$_b$O), 3.25 (0.14H, ddd, $^2J_{HH}$ = 10.2 Hz, $^3J_{HH}$ = 7.5 Hz, 3.1 Hz, NCH$_a$H$_b$C*H*$_a$H$_b$O), 3.15 (0.86H, $^2J_{HH}$ = 13.4 Hz, $^3J_{HH}$ = 6.3 Hz, 3.0 Hz, NCH$_a$*H*$_b$CH$_a$H$_b$O), 3.08 (0.14H, ddd, $^2J_{HH}$ = 13.5 Hz, $^3J_{HH}$ = 5.5 Hz, 2.9 Hz, NCH$_a$*H*$_b$CH$_a$H$_b$O), 3.02 (0.86H, ddd, $^2J_{HH}$ = 11.4 Hz, $^3J_{HH}$ = 6.9 Hz, 3.0 Hz, NCH$_a$H$_b$CH$_a$*H*$_b$O), 2.96 (0.14H, ddd, $^2J_{HH}$ = 11.0 Hz, $^3J_{HH}$ = 7.5 Hz, 3.0 Hz, NCH$_a$H$_b$CH$_a$*H*$_b$O); **^{13}C {^{1}H} NMR** (101 MHz, $CDCl_3$) δ_C 177.0 (*C*(O)NBn, both), 170.8 (*C*(O)N(CH_2CH_2)$_2$O, major), 169.3 (*C*(O)N(CH_2CH_2)$_2$O, minor), 139.7 (Ar*C*^{7a}, minor), 138.7 (Ar*C*^{7a}, major), 137.6 (Ph*C*$^{1'}$CH$_2$N, minor), 137.3 (Ph*C*$^{1'}$CH$_2$N, major), 133.0 (Ar*C*^{3a}, minor), 132.9 (Ph*C*$^{1''}$CH, minor), 132.7 (Ph*C*$^{1''}$CH, major), 132.3 (Ar*C*^{6}H, major), 131.4 (Ar*C*^{3a}, major), 130.2 (Ph*C*$^{2'',6''}$H, minor), 130.0 (Ph*C*$^{2'',6''}$H, major), 128.8 (Ph*C*H, major), 128.7 (Ph*C*H, minor), 128.6 (Ph*C*H, major), 128.5$_0$ (Ph*C*H, minor), 128.4$_8$ (Ph*C*H, minor), 128.4 (Ph*C*H, major), 127.2 (Ph*C*H, major), 127.0 (Ph*C*H, minor), 126.6 (Ph*C*$^{2',6'}$H, minor), 126.5 (Ph*C*$^{2',6'}$H), 125.9 (Ar*C*^{4}H, major), 123.6 (Ar*C*^{5}H, major), 123.4 (Ar*C*^{5}H, minor), 123.3 (Ar*C*^{4}H, minor), 115.5 (Ar*C*^{7}Cl, minor), 115.3 (Ar*C*^{7}Cl, major), 78.1 (*C*-OH, major), 76.4 (*C*-OH, minor), 66.7 (NCH$_c$H$_d$*C*H$_c$H$_d$O, both), 66.1 (NCH$_a$H$_b$*C*H$_a$H$_b$O, both), 55.6 (*C*H-Ph, minor), 53.0 (*C*H-Ph, major), 46.6 (N*C*H$_a$H$_b$CH$_a$H$_b$O, major), 46.4 (N*C*H$_a$H$_b$CH$_a$H$_b$O, minor), 45.2 (N*C*H$_2$-Ph, minor), 45.0 (N*C*H$_2$-Ph, major), 42.6 (N*C*H$_c$H$_d$CH$_c$H$_d$O, major), 42.2 (N*C*H$_c$H$_d$CH$_c$H$_d$O, minor); ***m/z*** (ESI$^+$) 477 ([M(^{35}Cl)+H]$^+$ 100%), 478 ([M(^{13}C,^{35}Cl)+H]$^+$ 29%), 479 ([M(^{13}C$_2$)+H]$^+$, [M(^{37}Cl)+H]$^+$ 34%), 480 ([M(^{13}C,^{37}Cl)+H]$^+$, 10%); **HRMS** (ESI$^+$) *m/z* calcd for [M(^{35}Cl)+H]$^+$ $C_{27}H_{26}O_4N_2Cl$ 477.1576, found 477.1559 (−3.5 ppm).

(±)-anti-**28** + (±)-syn-**28**

PDA Ch1 211nm

Peak#	Ret. Time	Area%
1	10.585	8.588
2	14.674	41.495
3	19.128	41.368
4	35.018	8.549
Total		100.000

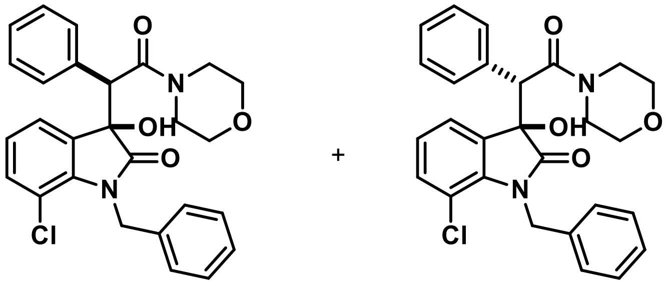

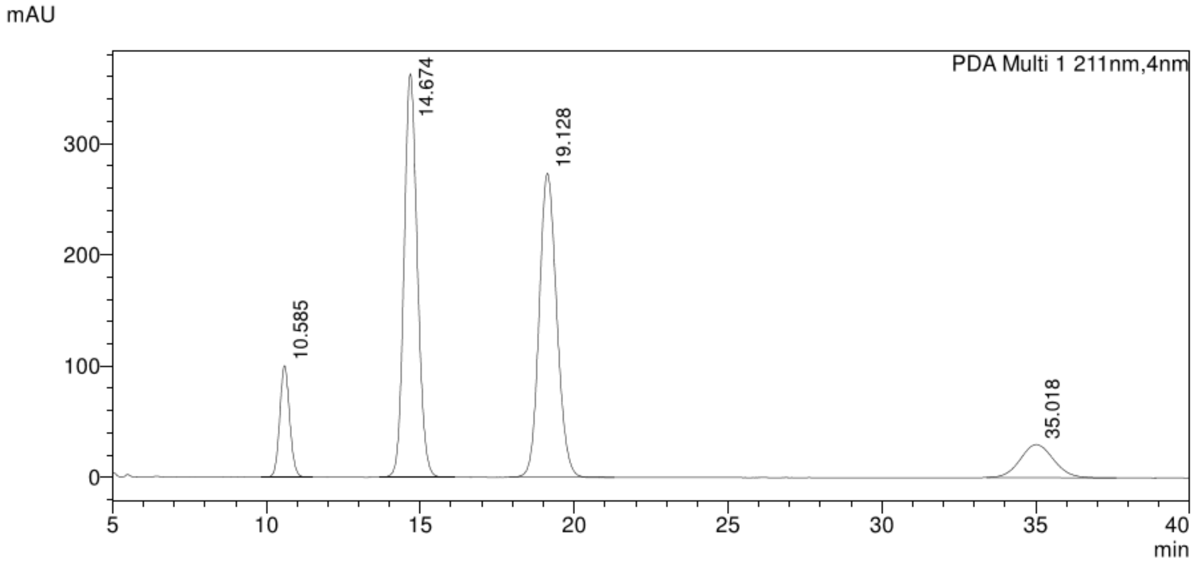

(2'''S,3S)-**28** + (2'''R,3S)-**28**

PDA Ch1 211nm

Peak#	Ret. Time	Area%
1	10.548	17.009
2	14.497	82.560
3	19.054	0.384
4	34.890	0.047
Total		100.000

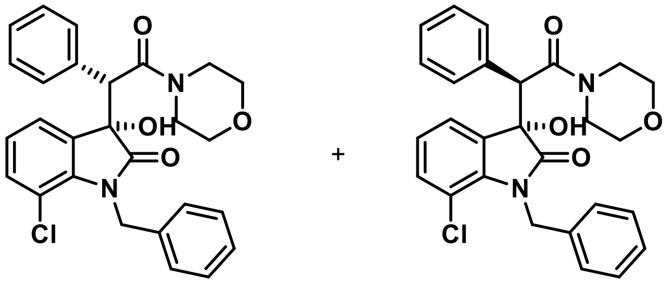

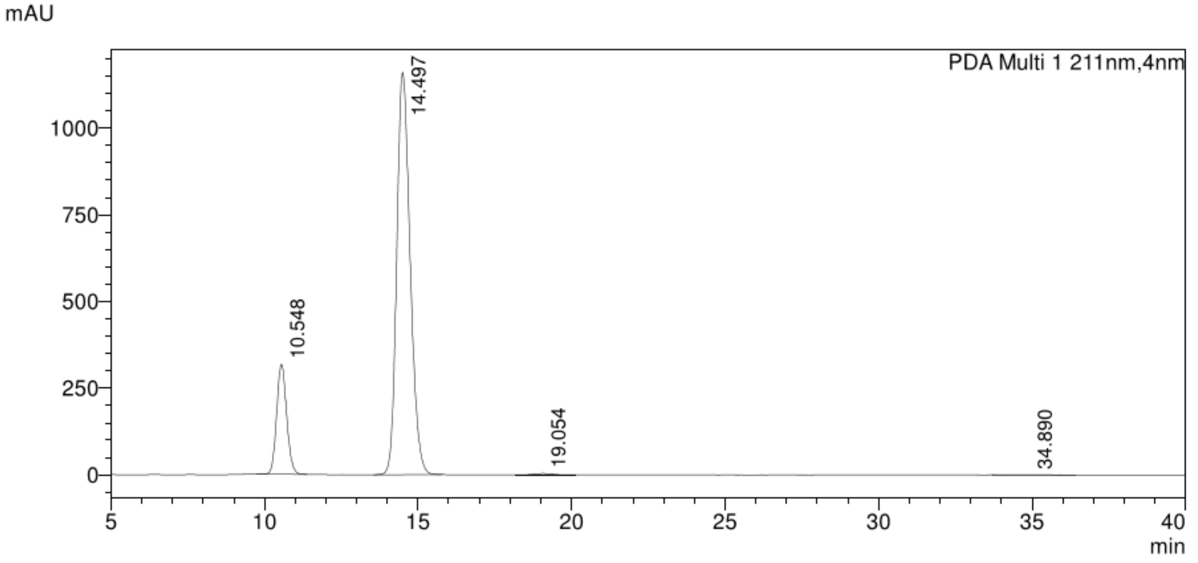

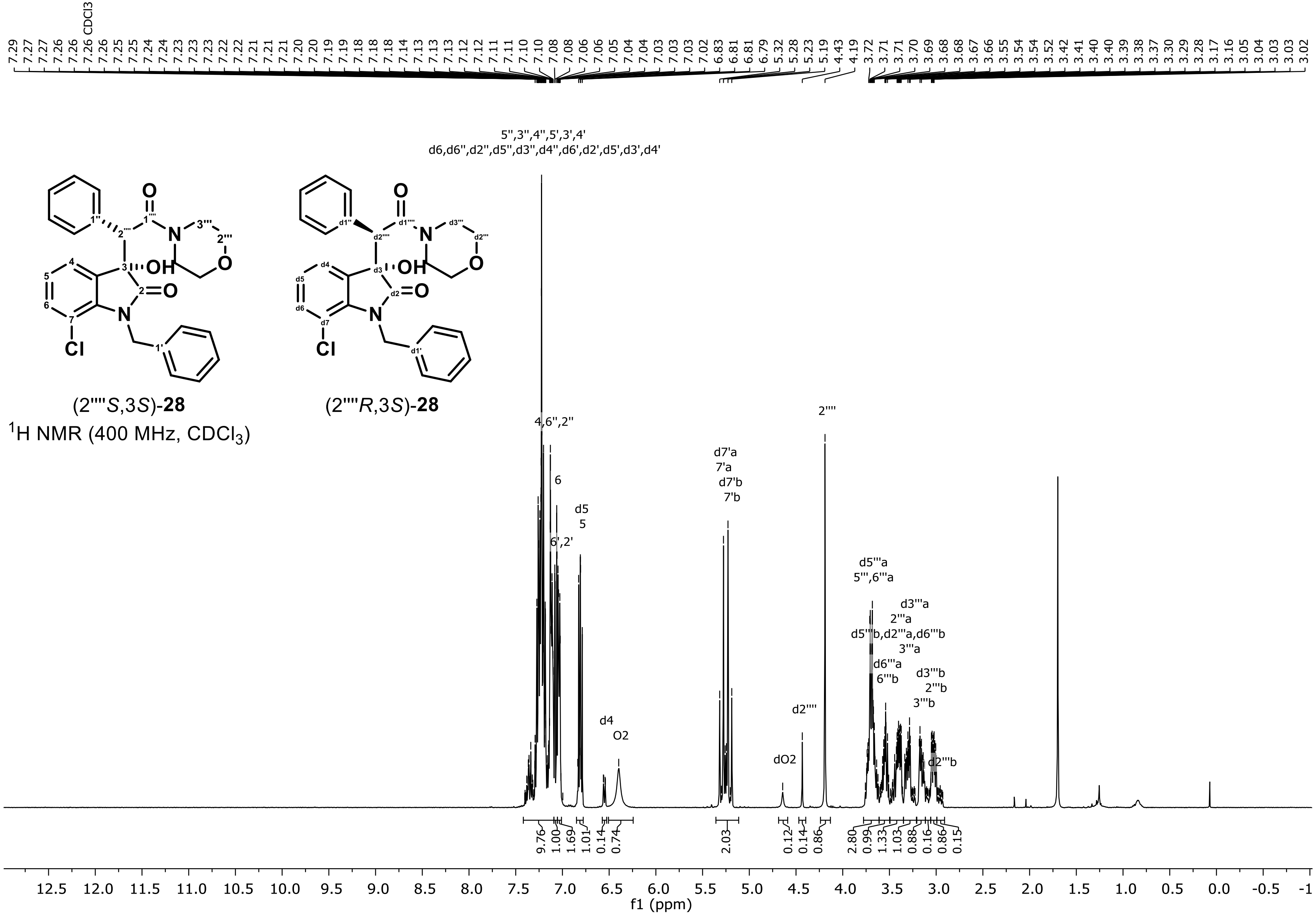

(2''''S,3S)-**28** (2''''R,3S)-**28**

^{1}H NMR (400 MHz, $CDCl_3$)

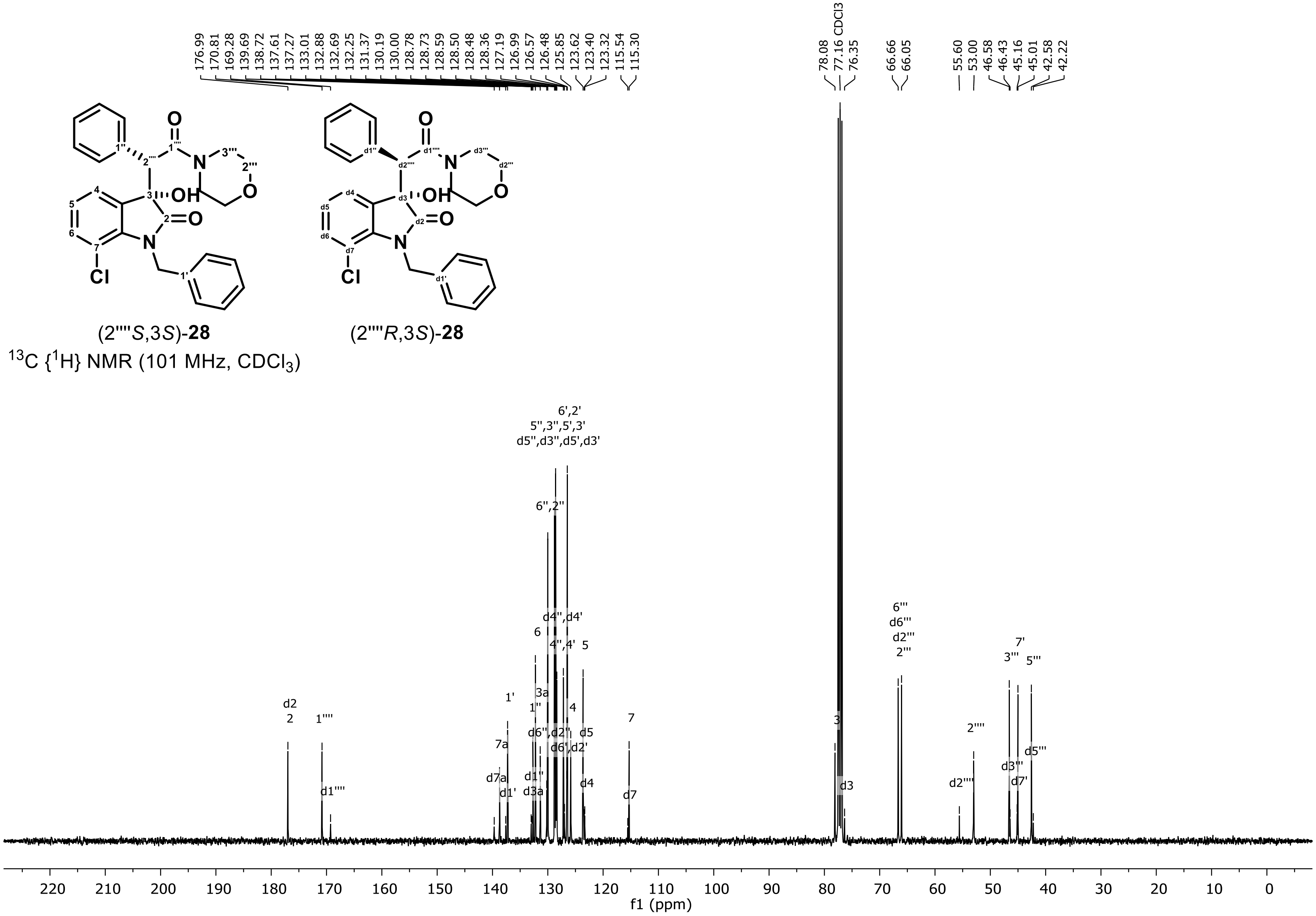

(2''''S,3S)-**28** (2''''R,3S)-**28**

$^{13}C\ \{^{1}H\}$ NMR (101 MHz, $CDCl_3$)

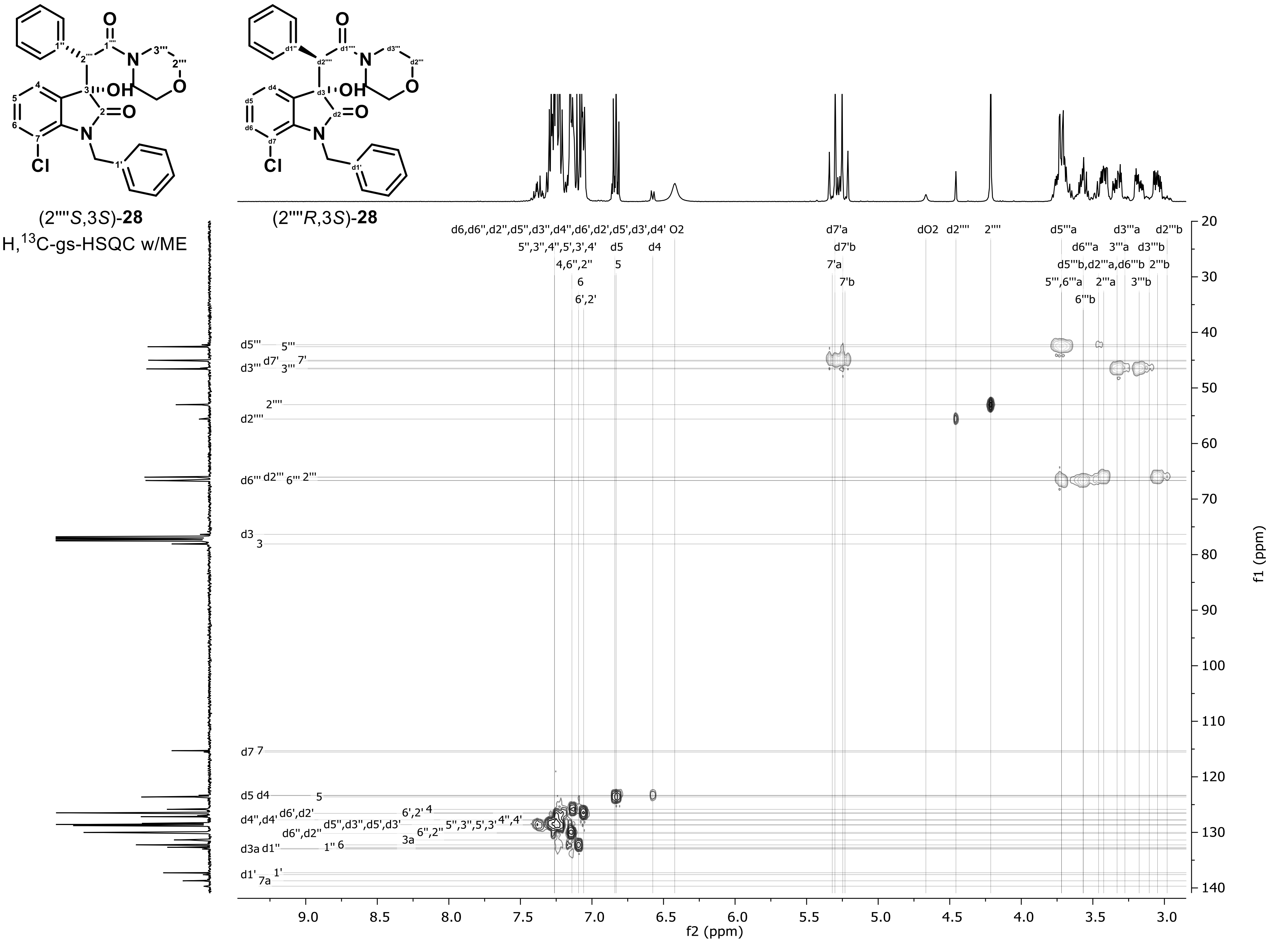

(2''''*S*,3*S*)-**28** (2''''*R*,3*S*)-**28**

$^{1}H,^{13}C$-gs-HSQC w/ME

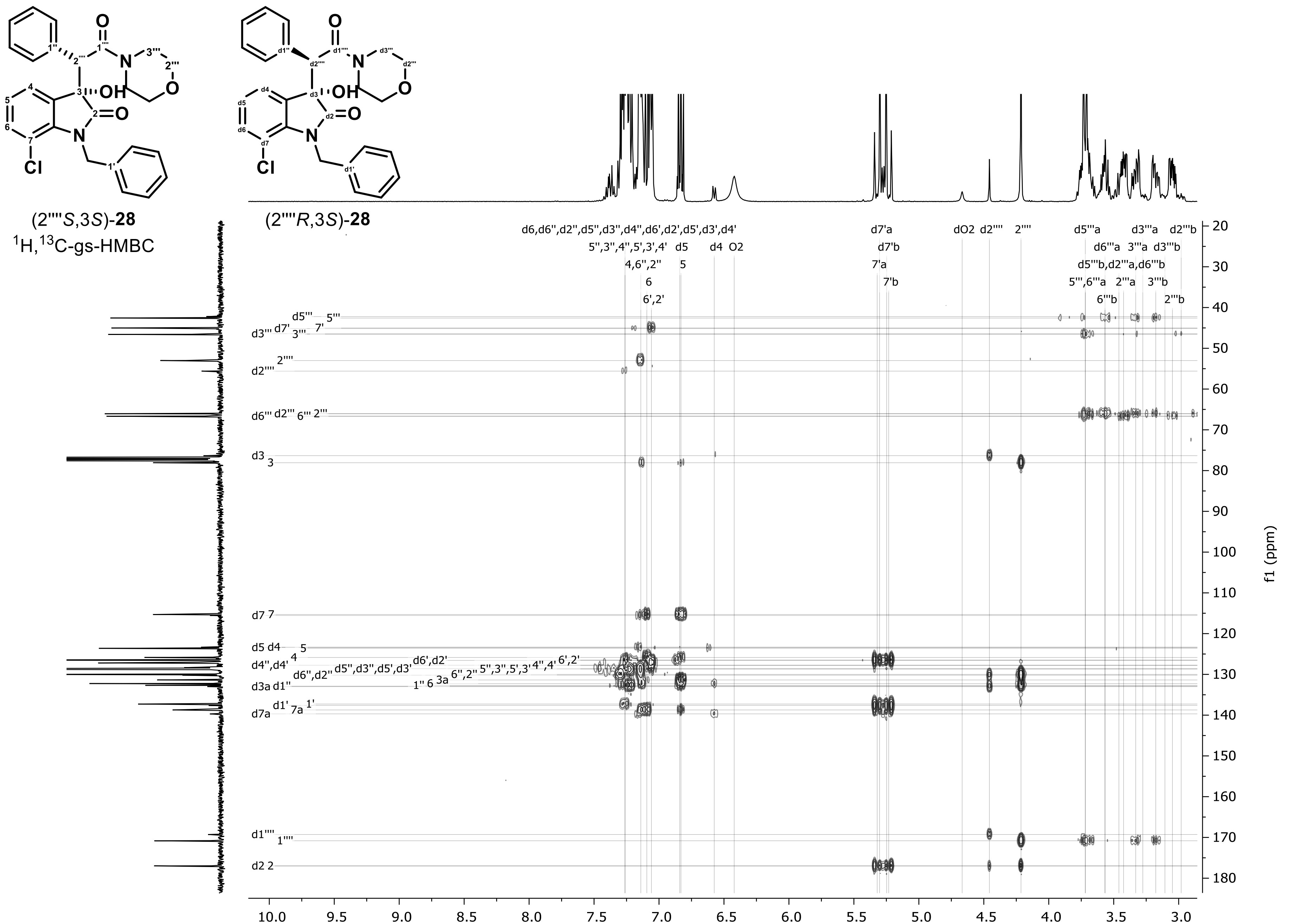

(2''''S,3S)-28
^{1}H,^{13}C-gs-HMBC
(2''''R,3S)-28
f2 (ppm)
f1 (ppm)

w) 1-Benzyl-6-chloro-3-hydroxy-3-(2-morpholino-2-oxo-1-phenylethyl)indolin-2-one (29)

kk2/50: To a 25 ml round bottomed flask was added phenylacetic anhydride (95.3 mg, 0.375 mmol), *N*-benzyl-6-chloroisatin (68.0 mg, 0.250 mmol), and (2*S*,3*R*)-HyperBTM (3.9 mg, 0.012 mmol). The mixture was cooled to 0 °C and CH_2Cl_2 (6.0 ml, 0.04 M) and Hünig's base (54 µl, 0.312 mmol) were added. The mixture was stirred at 0 °C for 3 h. Morpholine (65 µl, 0.750 mmol) was added and the reaction was left to be stirred overnight at room temperature. 1,3,5-trimethoxybenzene (0.1 M soln in CH_2Cl_2 500 µl, 0.05 mmol) was added and the solvent was removed under reduced pressure. Purification by column chromatography (Hexane : EtOAc 6:4) gave the title compound as yellow amorphous solid (94.5 mg, 0.202 mmol, 81%, 87:13 d.r.)

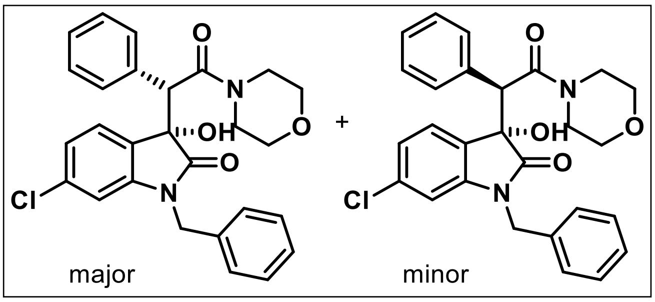

Major (2′′′′*S*,3*S*)-**29** and **minor** (2′′′′*R*,3*S*)-**29** analysed as an 87:13 mixture of diastereomers. **m.p.** 210 – 212 °C (rac); 169 – 176 °C (ee); **R*f*** 0.29 (Hexane:EtOAc 4:6); α_D^{20} = −68.7 (*c* 2.37 $CHCl_3$); **Chiral HPLC analysis** (Chiralcel® AD-H 85:15 Hexane:IPA, flow rate 2.0 ml·min^{-1}, 211 nm, 40 °C) t_R (2′′′′*R*,3*S*)-**29**: 10.1 min, t_R (2′′′′*S*,3*S*)-**29**: 11.1 min, >99:1 e.r.; t_R (2′′′′*S*,3*R*)-**29**: 26.2 min, t_R (2′′′′*R*,3*R*)-**29**: 23.0 min, >99:1 e.r.; ν_{max} (thin film) 3393 (m (br), O-H), 3063 (w, C-H), 3030 (w, C-H), 2965 (w, C-H), 2920 (w, C-H), 2859 (w, C-H), 1721 (s, C=O keonte), 1624 (s), 1607 (s, C=O amide), 1489 (m), 1454 (m), 1437 (s), 1375 (m), 1356 (m), 1339 (m), 1302 (m), 1267 (m), 1252 (m), 1227 (m), 1175 (m), 1113 (s), 1074 (s), 1031 (m), 1003 (m), 970 (w), 937 (w), 907 (s), 878 (m), 843 (m), 820 (m), 791 (w); **^{1}H NMR** (400 MHz, $CDCl_3$) δ_H 7.40 (0.87H, d, $^3J_{HH}$ = 8.1 Hz, ArC^4*H*), 7.38 – 7.19 (4.5H, m, PhC$^{3',4',5'}$*H* (major), PhC$^{4''}$*H* (major), PhC$^{2',3',4',5',6'}$*H* (minor), PhC$^{2'',3'',4'',5'',6''}$*H* (minor)), 7.16 (app t, $^3J_{HH}$ = 7.4 Hz, PhC$^{3'',5''}$*H*), 7.07 (app d, $^3J_{HH}$ = 7.2 Hz, PhC$^{2'',6''}$*H*), 6.98 – 6.91 (1.74H, m, PhC$^{2',6'}$*H*), 6.88 (0.87H, dd, $^3J_{HH}$ = 8.1 Hz, $^4J_{HH}$ = 1.9 Hz, ArC^5*H*), 6.80 (0.13H, dd, $^3J_{HH}$ = 8.0 Hz, $^4J_{HH}$ = 1.8 Hz, ArC^5*H*), 6.59 (0.13H, d, $^4J_{HH}$ = 1.8 Hz, ArC^7*H*), 6.44 (0.87H, d, $^4J_{HH}$ = 1.9 Hz, ArC^7*H*), 5.90 (0.87H, s(br), O*H*), 4.96 (0.13H, NC*H*$_a$H$_b$-Ph), 4.87 (0.87H, d, $^2J_{HH}$ = 15.9 Hz, NC*H*$_a$H$_b$-Ph), 4.63 (0.13H, d, $^2J_{HH}$ = 15.9 Hz, NCH$_a$*H*$_b$-Ph), 4.59 (0.87H, d, $^2J_{HH}$ = 15.9 Hz, NCH$_a$*H*$_b$-Ph), 4.48 (0.13H, s, C*H*-Ph), 4.38 (0.13H, s(br), O*H*), 4.34 (0.87H, s, C*H*-Ph), 3.76 (0.87H, ddd, $^2J_{HH}$ = 12.5 Hz, $^3J_{HH}$ = 5.7 Hz, 2.7 Hz, NC*H*$_c$H$_d$), 3.70 (1H, ddd, $^2J_{HH}$ = 11.0 Hz, $^3J_{HH}$ = 5.8 Hz, 2.6 Hz, OC*H*$_c$H$_d$, minor signal not resolved for NC*H*$_c$H$_d$), 3.62 (1H, ddd, $^2J_{HH}$ = 12.7 Hz, $^3J_{HH}$ = 7.0 Hz, 2.6 Hz, NCH$_c$H$_d$, minor signal not resolved for OCH$_c$*H*$_d$), 3.53 (0.87H, ddd, $^2J_{HH}$ = 10.3 Hz, $^3J_{HH}$ = 6.9 Hz, 2.8 Hz, OC*H*$_c$H$_d$), 3.44 (0.87H, ddd, $^2J_{HH}$ = 11.5 Hz, $^3J_{HH}$ = 6.1 Hz, 3.0 Hz, OC*H*$_c$H$_d$), 3.50 – 3.36 (0.39H, m (signals not resolved), NCH$_c$*H*$_d$, OCH$_c$*H*$_d$, OC*H*$_a$H$_b$), 3.33 (0.87H, ddd, $^2J_{HH}$ = 13.5 Hz, $^3J_{HH}$ = 7.2 Hz, 3.1 Hz, NC*H*$_a$H$_b$), 3.27 (0.13H, $^2J_{HH}$ = 10.6 Hz, $^3J_{HH}$ = 7.6 Hz, 3.0 Hz, NC*H*$_a$H$_b$), 3.16 (0.87H, ddd, $^2J_{HH}$ = 13.5 Hz, $^3J_{HH}$ = 6.1 Hz. 3.0 Hz, NCH$_a$*H*$_b$), 3.09 (0.13H, ddd, $^2J_{HH}$ = 13.6 Hz, $^3J_{HH}$ = 5.5 Hz, 2.9 Hz, NCH$_a$*H*$_b$), 3.01 (0.87H, ddd, $^2J_{HH}$ = 11.4 Hz, $^3J_{HH}$ = 7.1 Hz, 3.0 Hz, OCH$_a$*H*$_b$), 2.95 (0.13H, ddd, $^2J_{HH}$ = 11.0 Hz, $^3J_{HH}$ = 7.6 Hz, 2.9 Hz, OCH$_a$*H*$_b$); **^{13}C {^{1}H} NMR** (100 MHz, $CDCl_3$) δ_C 176.6 (*C*(O)NBn, minor), 176.0 (*C*(O)NBn, major), 170.7 (*C*(O)N$(CH_2)_2O(CH_2)_2$, major), 169.2 (*C*(O)N$(CH_2)_2O(CH_2)_2$, minor), 145.0 (Ar*C*^{7a}, minor), 144.0 (Ar*C*^{7a}, major), 135.3$_5$ (Ar*C*^{3a}, minor), 135.3$_0$ (Ar*C*^{3a}, major), 135.1 (PhC$^{1'}$CH$_2$N, minor), 134.7 (Ph*C*$^{1'}$CH$_2$N, major), 133.2 (Ph*C*$^{1''}$CH, minor), 132.7 (Ph*C*$^{1''}$CH, major), 130.2 (Ph*C*$^{2'',6''}$H, minor), 129.8 (Ph*C*$^{2'',6''}$H, major), 128.8$_4$ and 128.8$_1$ (Ph*C*$^{3',5'}$H and Ph*C*$^{3'5'}$H, major), 128.7 (Ar*C*), 128.5$_0$ (Ar*C*), 128.4$_5$ (Ar*C*), 128.2 (Ar*C*^{6}Cl, minor), 127.8 (Ar*C*), 127.7 (Ar*C*), 127.2 (Ph*C*$^{2',6'}$H, minor), 127.1 (Ar*C*^{6}Cl, major), 127.0 (Ph*C*$^{2',6'}$H, major), 122.7 (Ar*C*^{5}H, major), 122.2 (Ar*C*^{5}H, minor), 110.0 (Ar*C*^{7}H, minor), 109.7 (Ar*C*^{7}H, major), 78.7 (*C*-OH, major), 76.5 (*C*-OH, minor), 66.6 (O*C*H$_c$H$_d$, major and minor), 66.0 (O*C*H$_a$H$_b$, major and minor), 55.6 (*C*H-Ph, minor), 54.0 (*C*H-Ph, major), 46.5 (N*C*H$_a$H$_b$, major), 46.4 (N*C*H$_a$H$_b$, minor), 44.1 (N*C*H$_2$-Ph, minor), 43.9 (N*C*H$_2$-Ph, major), 42.5 (N*C*H$_c$H$_d$, major), 42.2 (N*C*H$_c$H$_d$, minor); ***m/z*** (ESI$^+$) 151 (5%), 206 (5%), 459 ([M(^{35}Cl)-OH]$^+$ 4%), 477 ([M(^{35}Cl)+H]$^+$ 100%), 478 ([M(^{13}C,^{35}Cl)+H]$^+$ 29%), 479 ([M(^{13}C$_2$,^{35}Cl)+H]$^+$ [M(^{37}Cl)+H]$^+$ 33%), 480

([M(^{13}C,^{37}Cl)+H]$^+$ 9%), 499 ([M(^{35}Cl)+Na]$^+$ 4%), 975 ([2M(^{35}Cl)+Na]$^+$ 5%); **HRMS** (ESI$^+$) *m/z* calcd for [M(^{35}Cl)+H]$^+$ $C_{27}H_{26}O_4N_2Cl$, found 477.1561 (−3.1 ppm).

(±)-anti-**29** + (±)-syn-**29**

PDA Ch1 211nm

Peak#	Ret. Time	Area%
1	10.054	24.684
2	11.116	25.322
3	23.053	25.472
4	26.051	24.522
Total		100.000

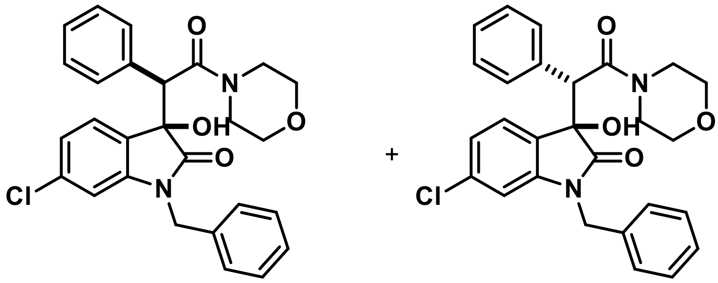

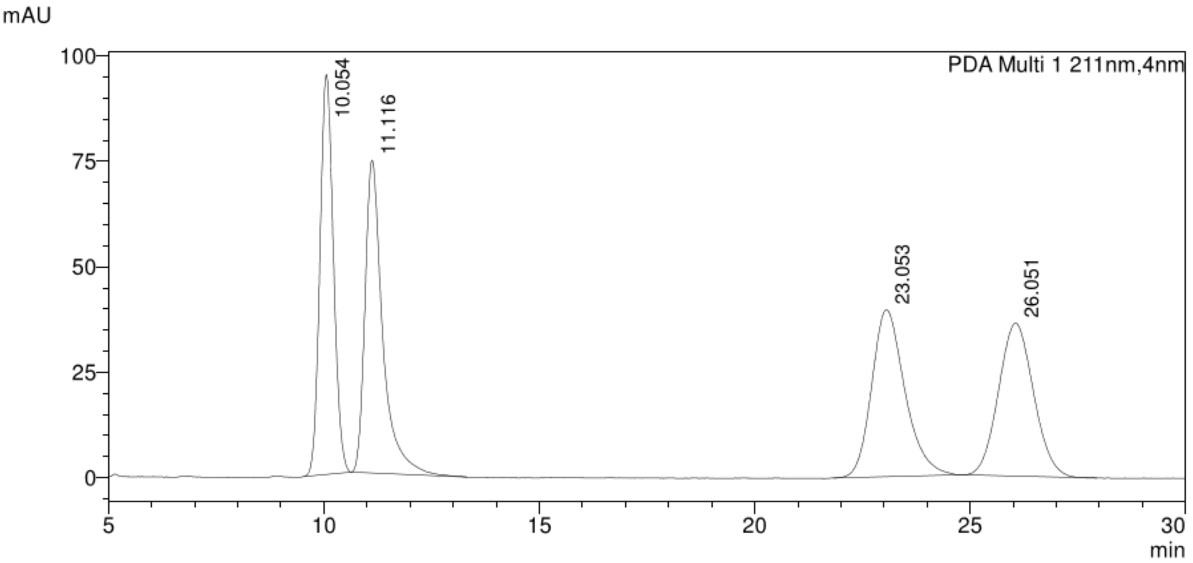

(2''''*S*,3*S*)-**29** + (2''''*R*,3*S*)-**29**

PDA Ch1 211nm

Peak#	Ret. Time	Area%
1	10.108	14.376
2	11.050	85.355
3	23.011	0.184
4	26.154	0.085
Total		100.000

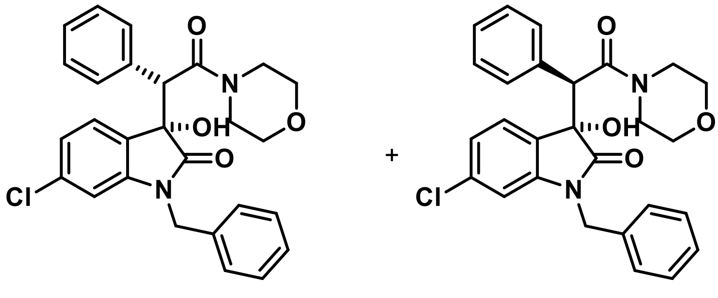

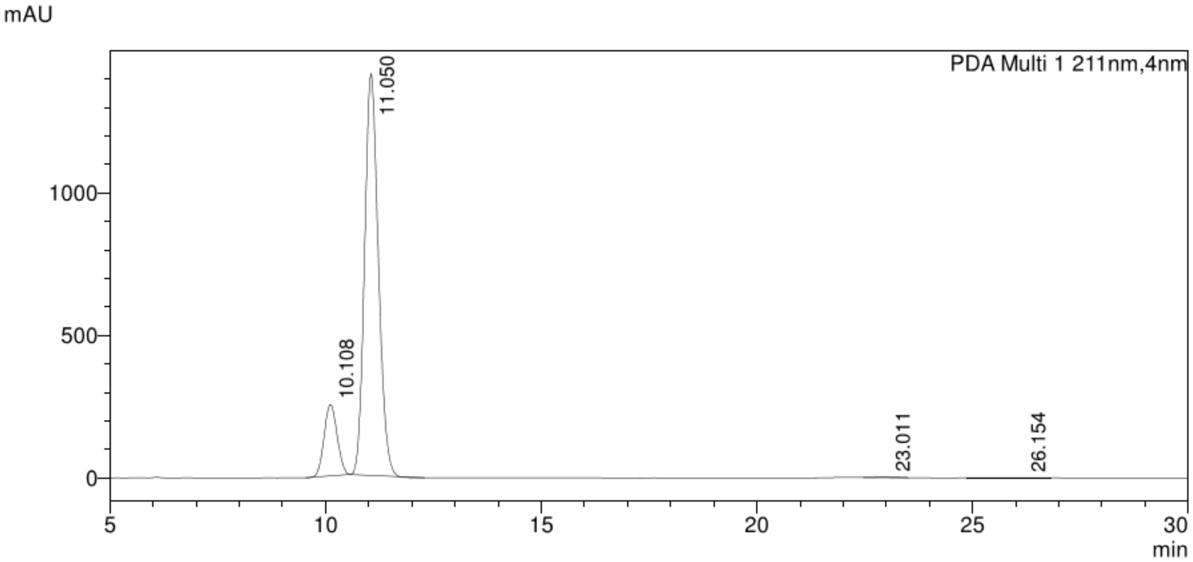

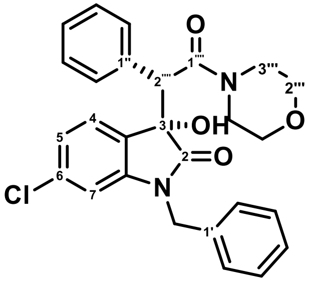

(2''''*S*,3*S*)-**29**

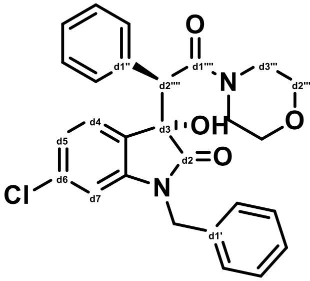

(2''''*R*,3*S*)-**29**

^{1}H NMR (400 MHz, $CDCl_3$)

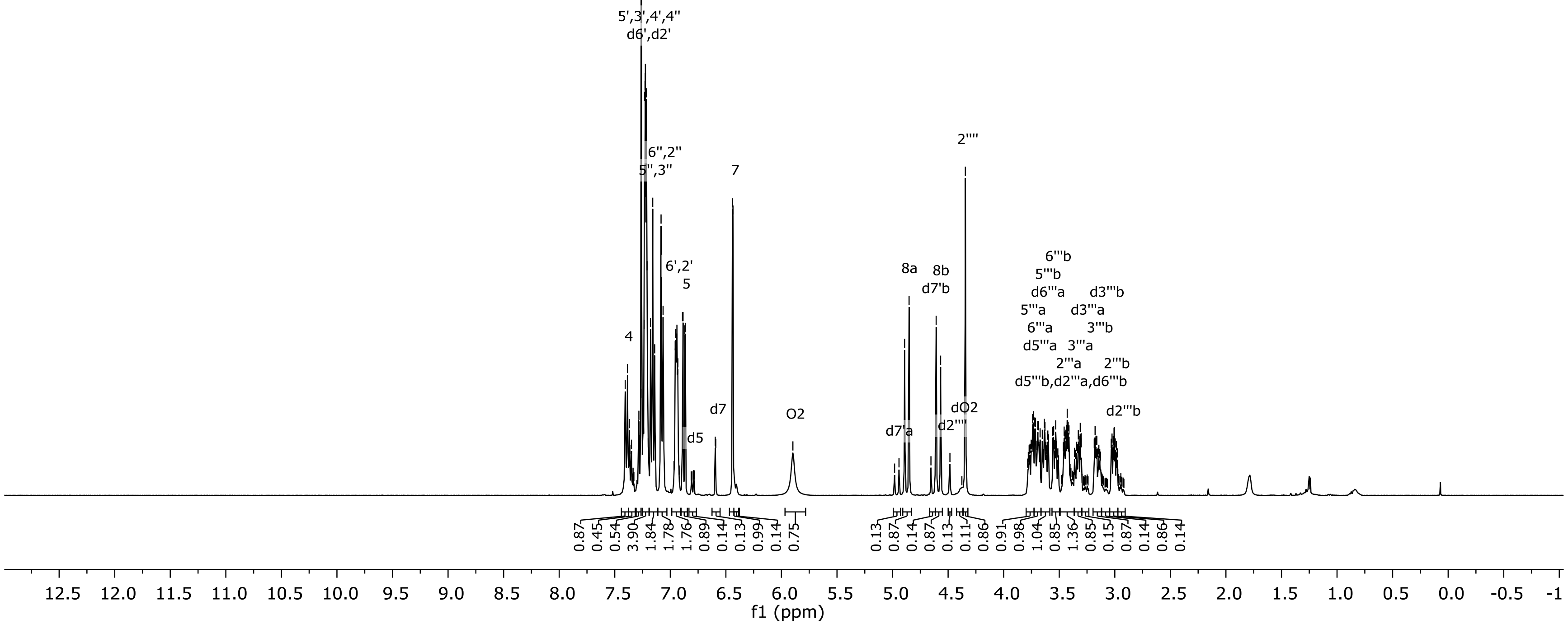

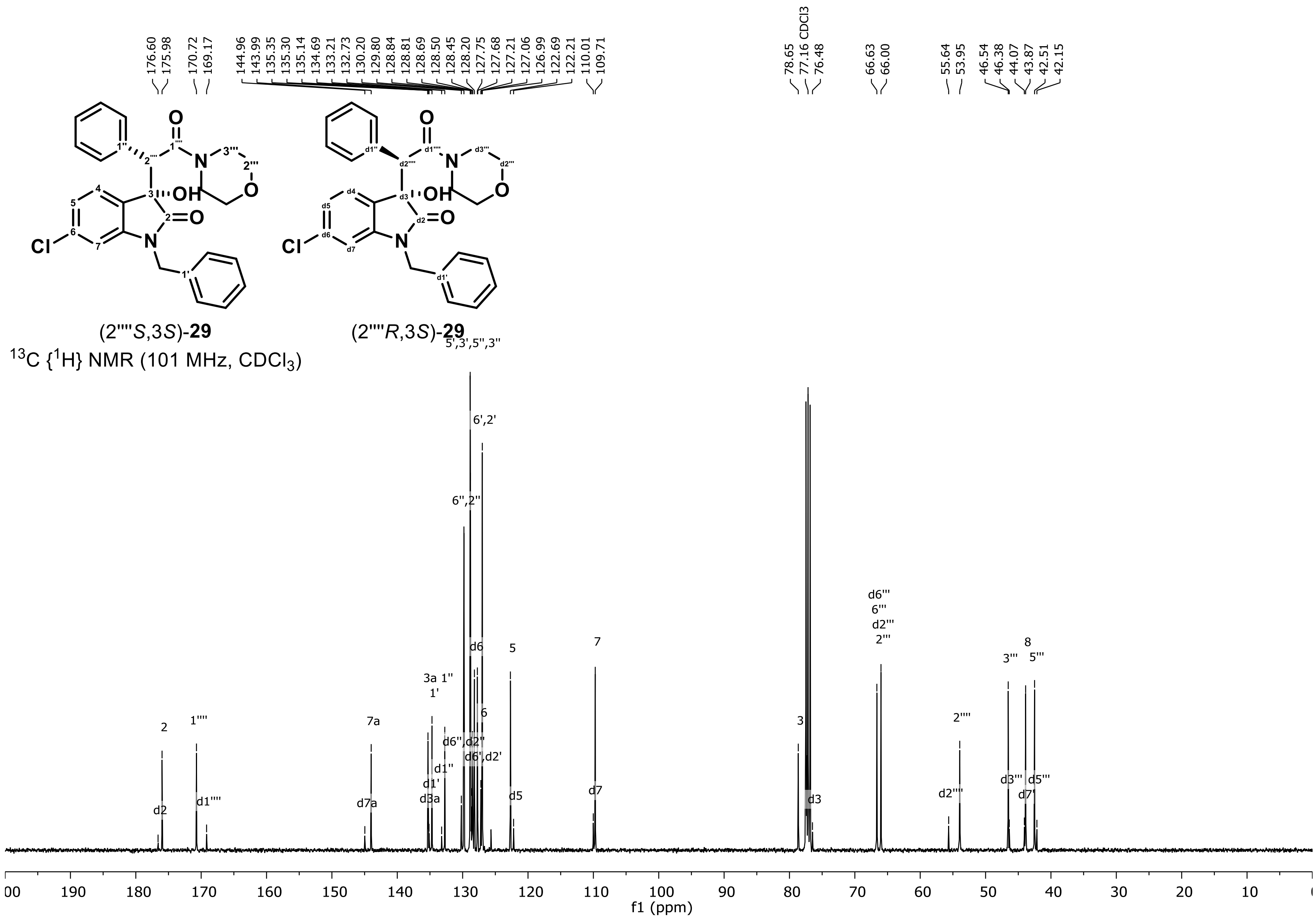
(2''''S,3S)-29
(2''''R,3S)-29
^{13}C {^{1}H} NMR (101 MHz, $CDCl_3$)
176.60
175.98
170.72
169.17
144.96
143.99
135.35
135.30
135.14
134.69
133.21
132.73
130.20
129.80
128.84
128.81
128.69
128.50
128.45
128.20
127.75
127.68
127.21
127.06
126.99
122.69
122.21
110.01
109.71
78.65
77.16 CDCl3
76.48
66.63
66.00
55.64
53.95
46.54
46.38
44.07
43.87
42.51
42.15
f1 (ppm)

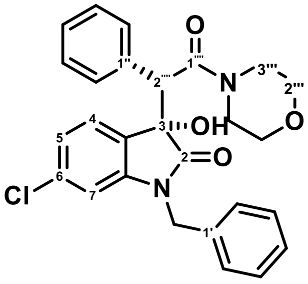

(2''''*S*,3*S*)-**29**
^{1}H,^{13}C-gs-HSQC w/ME

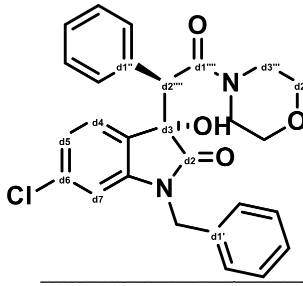

(2''''*R*,3*S*)-**29**

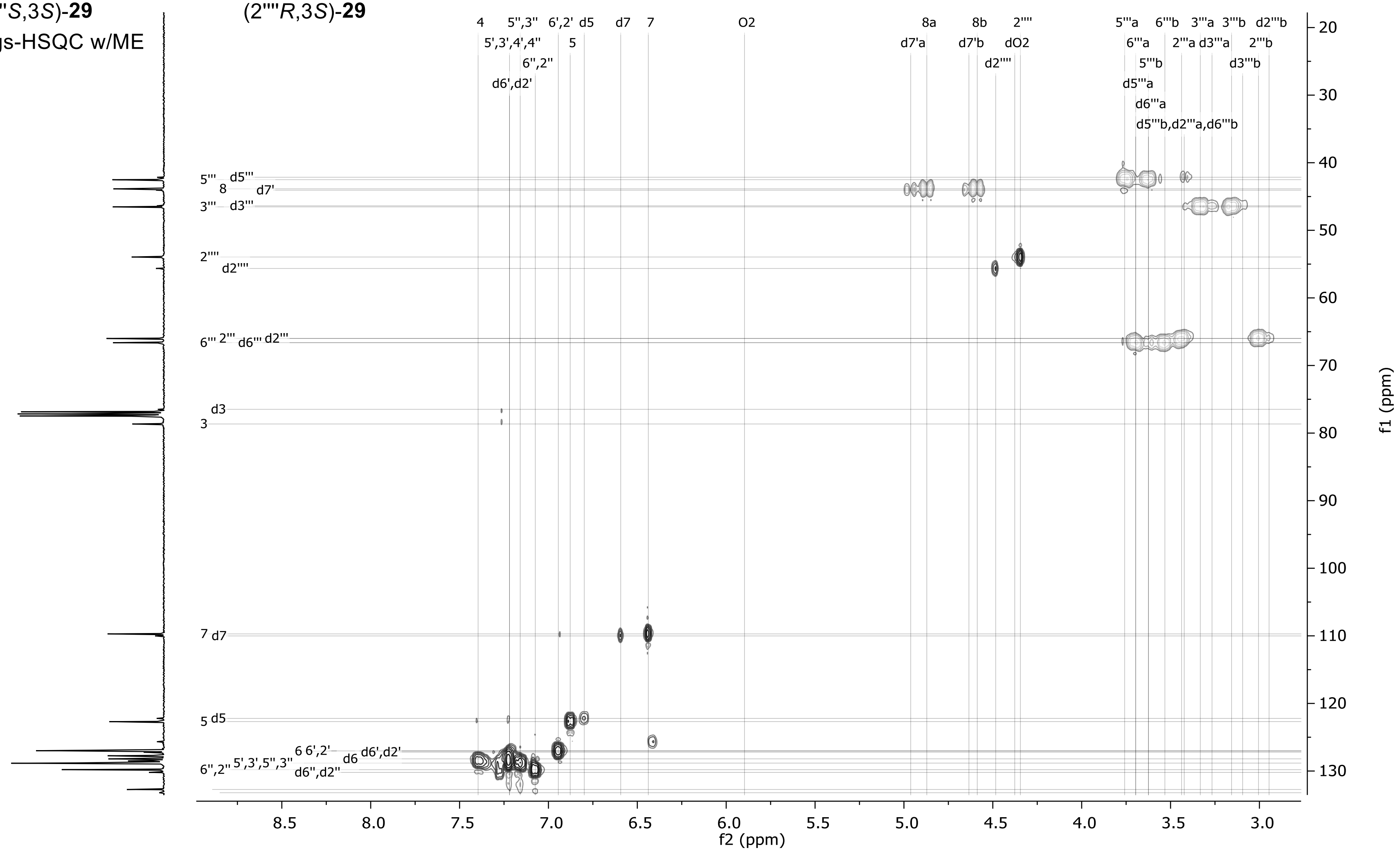

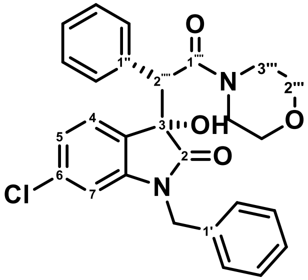

(2''''*S*,3*S*)-**29**

^{1}H,^{13}C-gs-HMBC

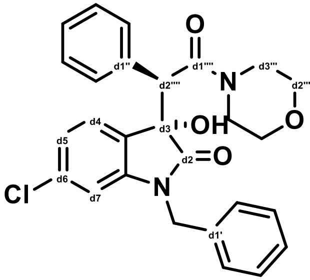

(2''''*R*,3*S*)-**29**

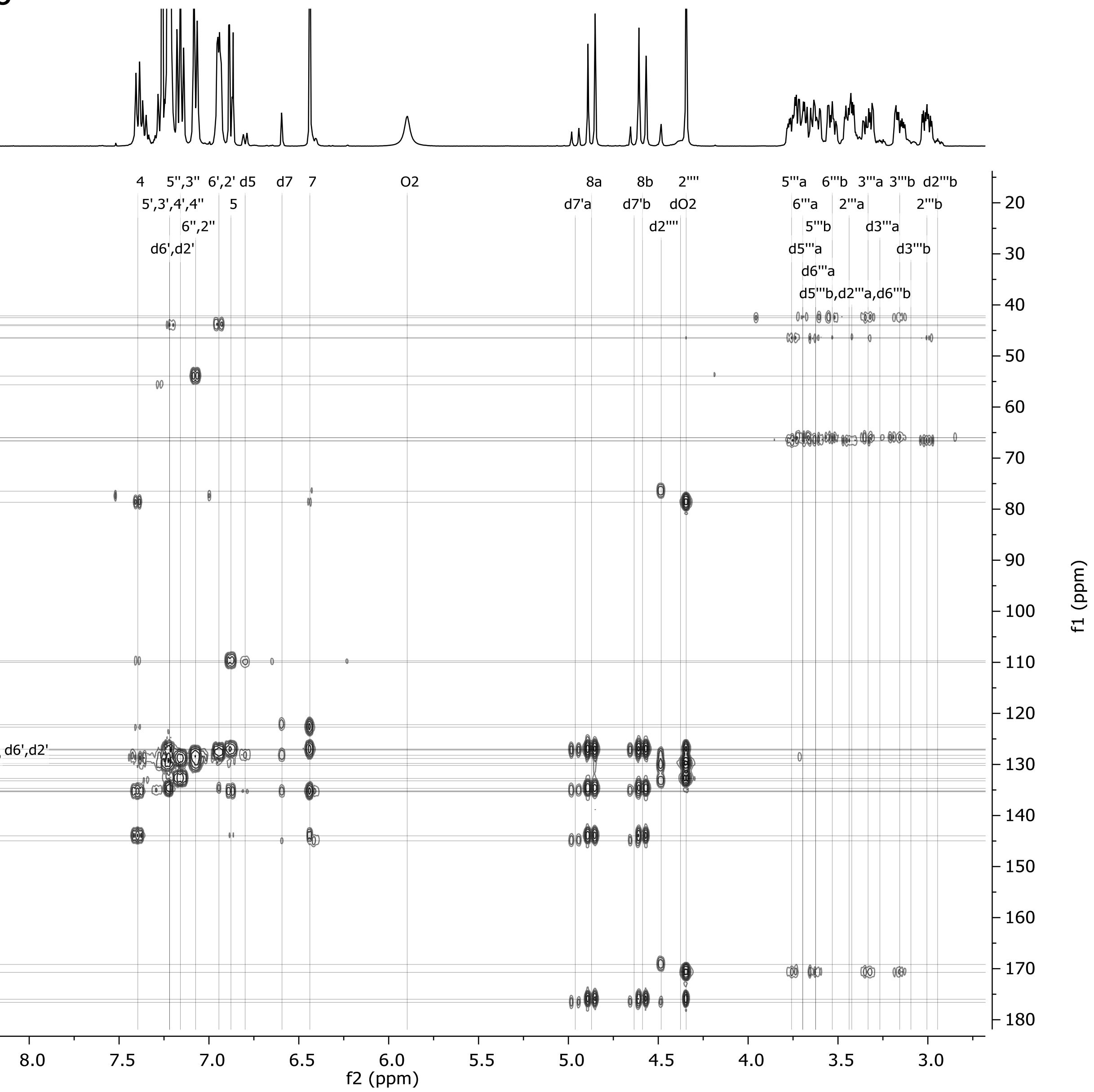

x) 1-Benzyl-5-chloro-3-hydroxy-3-(2-morpholino-2-oxo-1-phenylethyl)indolin-2-one (30)

To a 25 ml round bottomed flask was added phenylacetic anhydride (95.3 mg, 0.375 mmol), *N*-benzyl-5-chloroisatin (68.0 mg, 0.250 mmol), and (2*S*,3*R*)-HyperBTM (3.9 mg, 0.012 mmol). The mixture was cooled to 0 °C and CH_2Cl_2 (6.0 ml, 0.04 M) and Hünig's base (54 µl, 0.312 mmol) were added. The mixture was stirred at 0 °C for 3 h. Morpholine (65 µl, 0.750 mmol) was added and the reaction was left to be stirred overnight at room temperature. 1,3,5-trimethoxybenzene (0.1 M soln in CH_2Cl_2 500 µl, 0.05 mmol) was added and the solvent was removed under reduced pressure. Purification by column chromatography (Hexane : EtOAc 7:3 → 3:7) gave the title compound in 2 fractions: a 1:1 mixture of diastereomers (33.9 mg, 0.07 mmol) as dark yellow solid (m.p. 190 °C, dec) and a 92:8 mixture of diastereomers (18.3 mg, 0.04 mg) as light brown semi-solid to give a combined yield of 45% (52.2 mg, 0.112 mmol, reduced yield due to solubility issue).

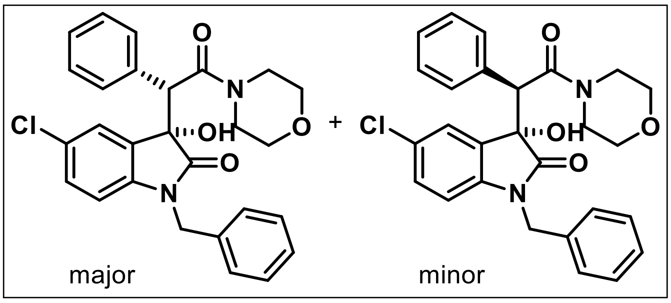

Major (2′′′′*S*,3*S*)-**30** + **minor** (2′′′′*R*,3*S*)-**30** analysed as 92:8 mixture of diastereomers. **R*f*** 0.37 (Hexane:EtOAc 2:8), **R*f*** 0.41 (CH_2Cl_2:EtOAc 7:3); **m.p.** 190 °C dec; $\boldsymbol{\alpha_D^{20}}$ = +9.1 (*c* 0.40, $CHCl_3$) (50:50 d.r.); **Chiral HPLC analysis** (Chiralcel® AD-H 85:15 Hexane:IPA, flow rate 2.0 ml·min^{-1}, 211 nm, 40 °C) t_R (2′′′′*S*,3*S*)-**30**: 10.6 min, t_R (2′′′′*R*,3*R*)-**30**: 36.9 min, >99:1 e.r.; t_R (2′′′′*R*,3*S*)-**30**: 22.2 min, t_R (2′′′′*S*,3*R*)-**30**: 25.2 min, 76:24 e.r. (due to solubility issues on column and HPLC); not all signals for minor diastereomer are resolved; $\boldsymbol{\nu_{max}}$ (thin film) 3327 (m, broad, OH, NH), 3061 (w), 3030 (w), 2961 (w), 2922 (w), 2857 (w), 1722 (s, C=O, lactam), 1626 (s, C=O, amide), 1609 (m), 1584 (w), 1497 (w), 1479 (m), 1454 (m), 1435 (s), 1375 (w), 1342 (m), 1302 (w), 1257 (m), 1227 (m), 1173 (m), 1115 (m), 1080 (m), 1030 (m), 972 (w), 943 (w), 908 (s), 860 (w), 829 (w), 812 (w); **^{1}H NMR** (400 MHz, $CDCl_3$) δ_H 7.57 (1H, d, $^4J_{HH}$ = 2.2 Hz, ArC^4*H*), 7.24 – 7.13 (6H, m, PhC$^{3',4',5'}$*H*, PhC$^{3'',4'',5''}$*H*), 7.10 – 7.05 (2H, m, PhC$^{2'',6''}$*H*), 7.03 (dd, $^3J_{HH}$ = 8.3 Hz, $^4J_{HH}$ = 2.2 Hz, ArC^6*H*), 6.95 – 6.87 (2H, m, PhC$^{2',6'}$*H*), 6.33 (1H, d, $^3J_{HH}$ = 8.3 Hz, ArC^7*H*), 5.79 (1H, s(br), O*H*), 4.88 (1H, d, $^2J_{HH}$ = 15.9 Hz, NC*H*$_a$H$_b$-Ph), 4.62 (1H, d, $^2J_{HH}$ = 15.9 Hz, NCH$_a$*H*$_b$-Ph), 4.41 (1H, s, *C*H-Ph), 3.80 (1H, ddd, $^2J_{HH}$ = 12.8 Hz, $^3J_{HH}$ = 5.7 Hz, 2.8 Hz, NC*H*$_c$H$_d$), 3.73 (1H, ddd, $^2J_{HH}$ = 11.1 Hz, $^3J_{HH}$ = 5.7 Hz, 2.6 Hz, OC*H*$_c$H$_d$), 3.64 (1H, ddd, $^2J_{HH}$ = 12.8 Hz, $^3J_{HH}$ = 7.1 Hz, 2.6 Hz, NCH$_c$*H*$_d$), 3.57 (1H, ddd, $^2J_{HH}$ = 11.1 Hz, $^3J_{HH}$ = 7.1 Hz, 2.8 Hz, OCH$_c$*H*$_d$), 3.45 (1H, ddd, $^2J_{HH}$ = 11.5 Hz, $^3J_{HH}$ = 6.0 Hz, 3.0 Hz, OC*H*$_a$H$_b$), 3.33 (1H, ddd, $^2J_{HH}$ = 13.5 Hz, $^3J_{HH}$ = 7.2 Hz, 3.0 Hz, NC*H*$_a$H$_b$), 3.15 (1H, ddd, $^2J_{HH}$ = 13.5 Hz, $^3J_{HH}$ = 6.0 Hz, 3.0 Hz, NCH$_a$*H*$_b$), 3.01 (1H, ddd, $^2J_{HH}$ = 11.5 Hz, $^3J_{HH}$ = 7.2 Hz, 3.1 Hz, OCH$_a$*H*$_b$); **^{13}C {^{1}H} NMR** (101 MHz, $CDCl_3$) δ_C 175.5 (*C*(O)NBn), 170.7 (*C*(O)N(CH$_2$)$_2$O(CH$_2$)$_2$), 141.3 (Ar*C*^{7a}), 134.8 (Ph*C*$^{1'}$CH$_2$), 132.7 (Ph*C*$^{1''}$CH), 130.4 (Ar*C*^{3a}), 129.8 (Ph*C*$^{2'',6''}$H), 129.5 (Ar*C*^{6}H), 128.9 and 128.8 (Ph*C*$^{3',5'}$H, Ph*C*$^{3'',5''}$H), 128.3$_2$ (Ar*C*^{5}Cl), 128.2$_6$ (Ph*C*$^{4''}$H), 128.0 (Ar*C*^{4}H), 127.7 (Ph*C*$^{4'}$H), 127.0 (Ph*C*$^{2',6'}$H), 110.2 (Ar*C*^{7}H), 79.2 (*C*-OH), 66.7 (O*C*H$_c$H$_d$), 66.1 (O*C*H$_a$H$_b$), 54.5 (Ph-*C*H), 46.7 (N*C*H$_a$H$_b$), 44.0 (Ph-*C*H$_2$), 42.6 (N*C*H$_c$H$_d$); ***m/z*** (ESI$^+$) 225 (42%), 399 (4%), 449 (13%), 477 ([M(^{35}Cl)+H]$^+$ 100%), 478 ([M(^{13}C,^{35}Cl)+H]$^+$ 29%), 479 ([M(^{13}C$_2$,^{35}Cl)+H]$^+$, [M(^{37}Cl)+H]$^+$ 32%), 480 ([M(^{13}C,^{37}Cl)+H]$^+$ 10%), 499 ([M(^{35}Cl)+Na]$^+$ 4%), 975 ([2M(^{35}Cl)+Na]$^+$ 8%); **HRMS** (ESI$^+$) *m/z* calcd for [M(^{35}Cl)+H]$^+$ $C_{27}H_{26}O_4N_2Cl$ 477.1576, found 477.1562 (−2.9 ppm).

(±)-anti-**30** + (±)-syn-**30**

PDA Ch1 211nm

Peak#	Ret. Time	Area%
1	10.659	46.196
2	22.207	3.804
3	25.292	3.768
4	37.159	46.232
Total		100.000

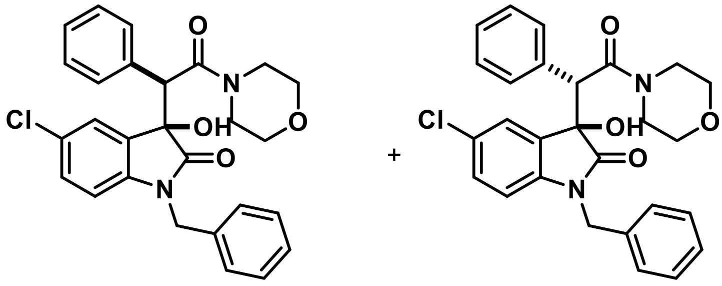

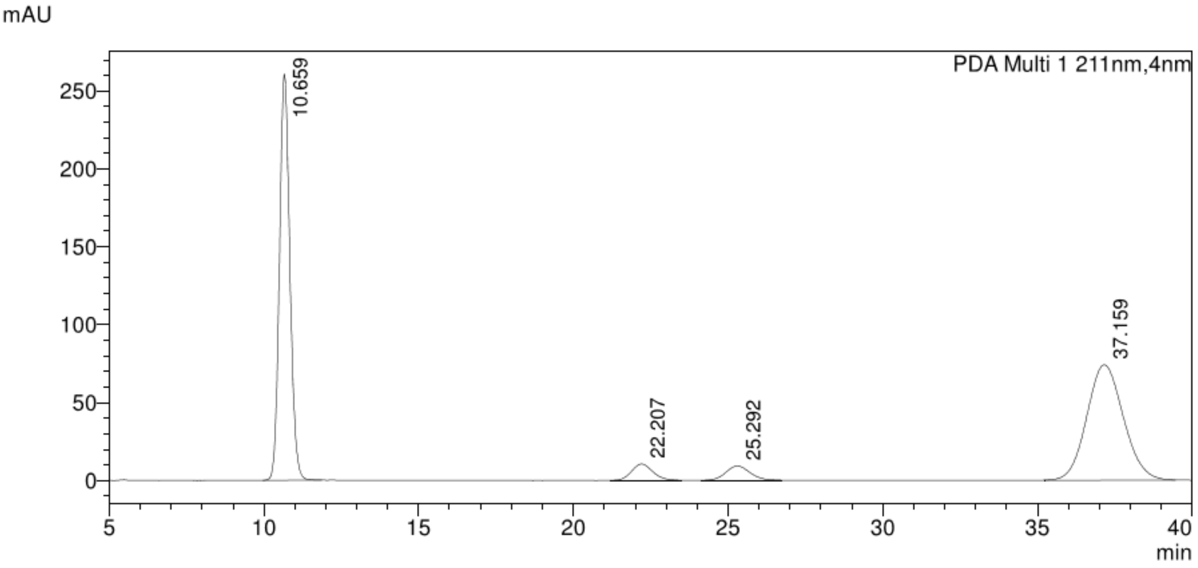

(2''''*S*,3*S*)-**30** + (2''''*R*,3*S*)-**30**

PDA Ch1 211nm

Peak#	Ret. Time	Area%
1	10.640	81.353
2	22.182	14.002
3	25.241	4.309
4	36.930	0.337
Total		100.000

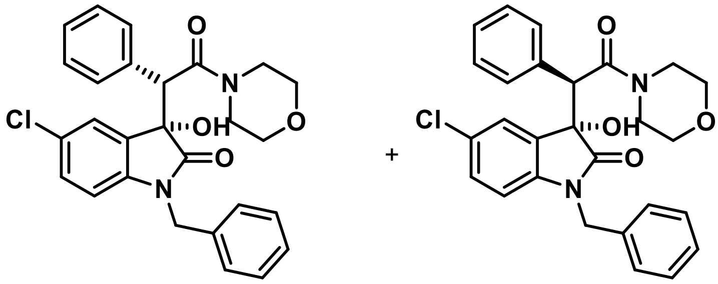

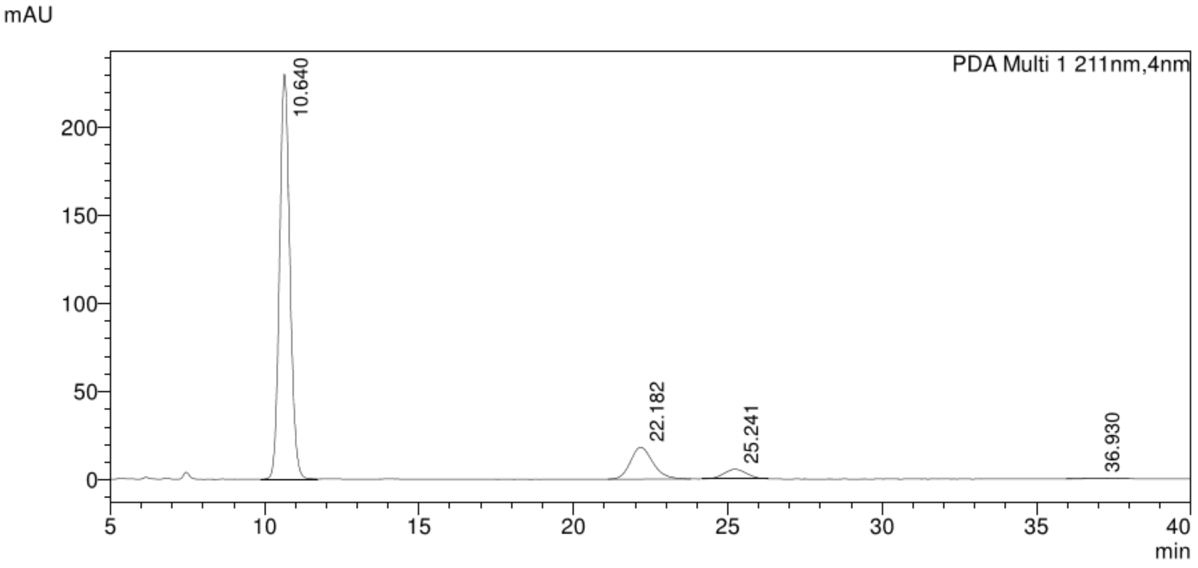

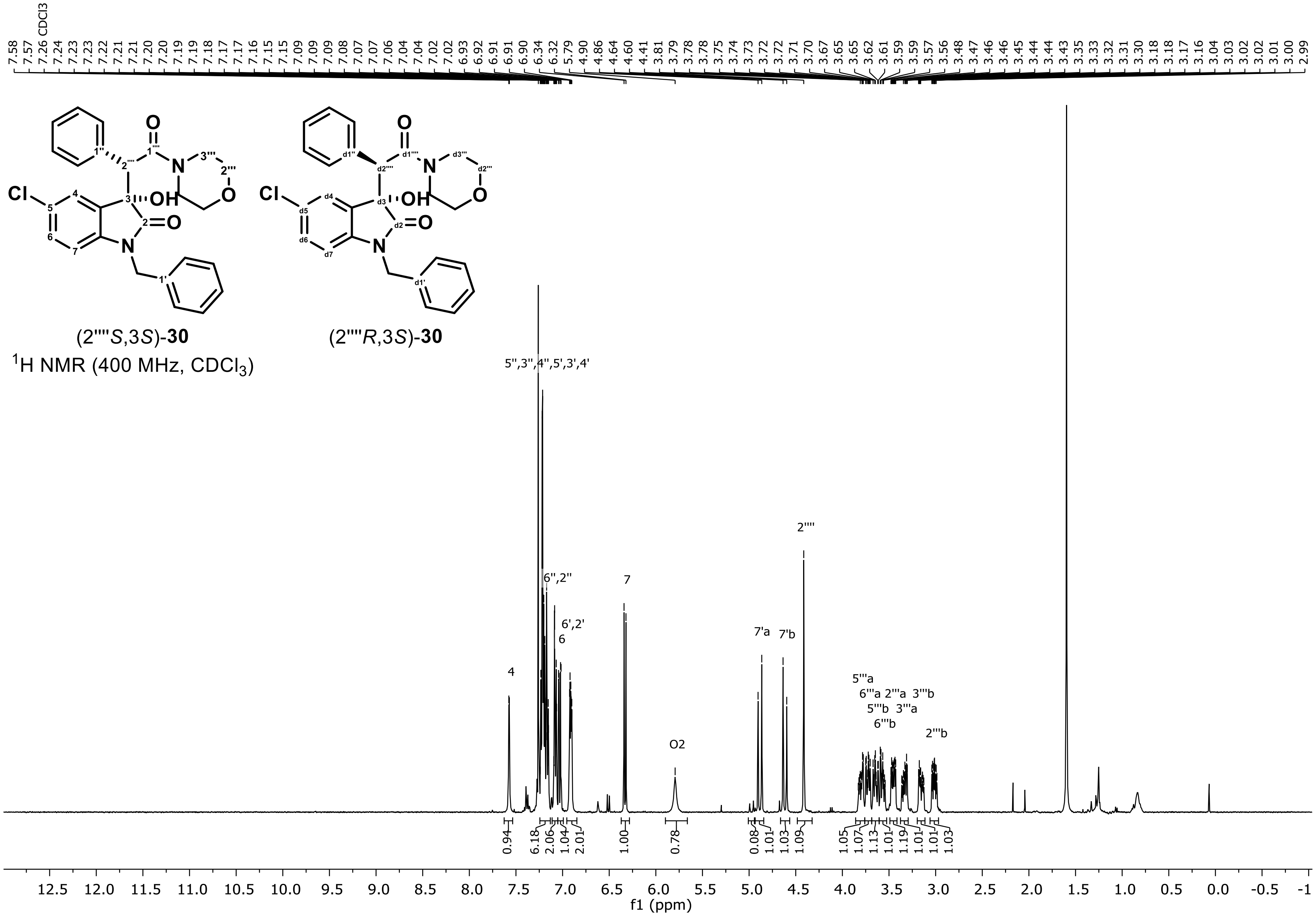
7.58 7.57 7.26 CDCl3 7.24 7.23 7.23 7.22 7.21 7.21 7.20 7.19 7.19 7.18 7.17 7.17 7.16 7.15 7.15 7.09 7.09 7.09 7.08 7.07 7.07 7.06 7.04 7.04 7.02 7.02 6.93 6.92 6.91 6.90 6.34 6.32 5.79 4.90 4.86 4.64 4.60 4.41 3.81 3.79 3.78 3.78 3.75 3.74 3.73 3.72 3.72 3.71 3.70 3.67 3.65 3.65 3.62 3.61 3.59 3.59 3.57 3.56 3.48 3.47 3.46 3.46 3.45 3.44 3.44 3.43 3.35 3.33 3.32 3.31 3.30 3.18 3.18 3.17 3.16 3.04 3.03 3.02 3.02 3.01 3.00 2.99
(2''''S,3S)-30
(2''''R,3S)-30
^{1}H NMR (400 MHz, $CDCl_3$)
5'',3'',4'',5',3',4'
6'',2''
6',2'
6
4
7
O2
7'a
7'b
2''''
5'''a
6'''a 2'''a 3'''b
5'''b 3'''a
6'''b
2'''b
0.94 6.18 2.06 1.04 2.01 1.00 0.78 0.08 1.01 1.03 1.09 1.05 1.07 1.13 1.01 1.19 1.01 1.03
12.5 12.0 11.5 11.0 10.5 10.0 9.5 9.0 8.5 8.0 7.5 7.0 6.5 6.0 5.5 5.0 4.5 4.0 3.5 3.0 2.5 2.0 1.5 1.0 0.5 0.0 -0.5 -1
f1 (ppm)

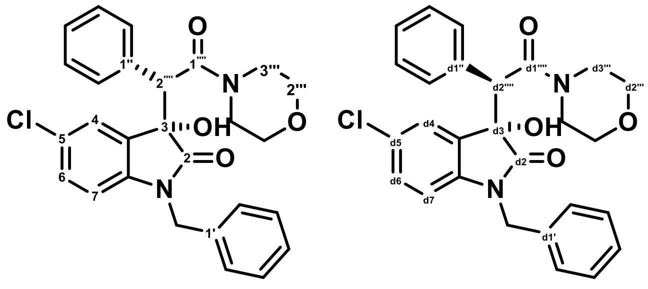

(2''''S,3S)-**30** (2''''R,3S)-**30**

$^{13}C\{^{1}H\}$ NMR (101 MHz, $CDCl_3$)

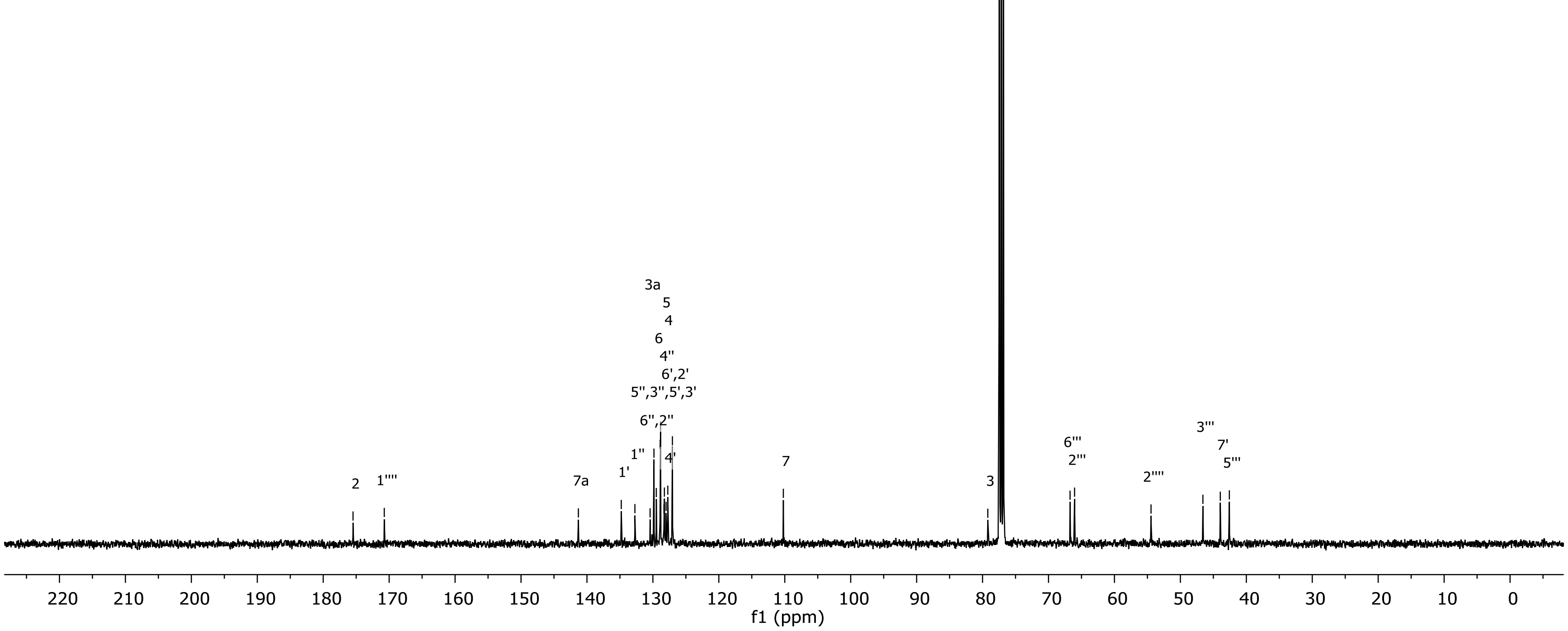

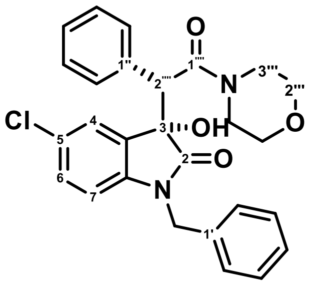

(2''''S,3S)-**30**

$^1H,^{13}C$-gs-HSQC w/ME

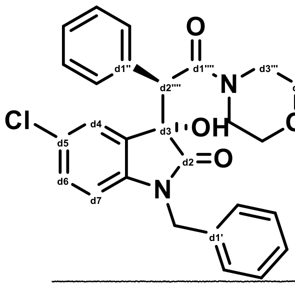

(2''''R,3S)-**30**

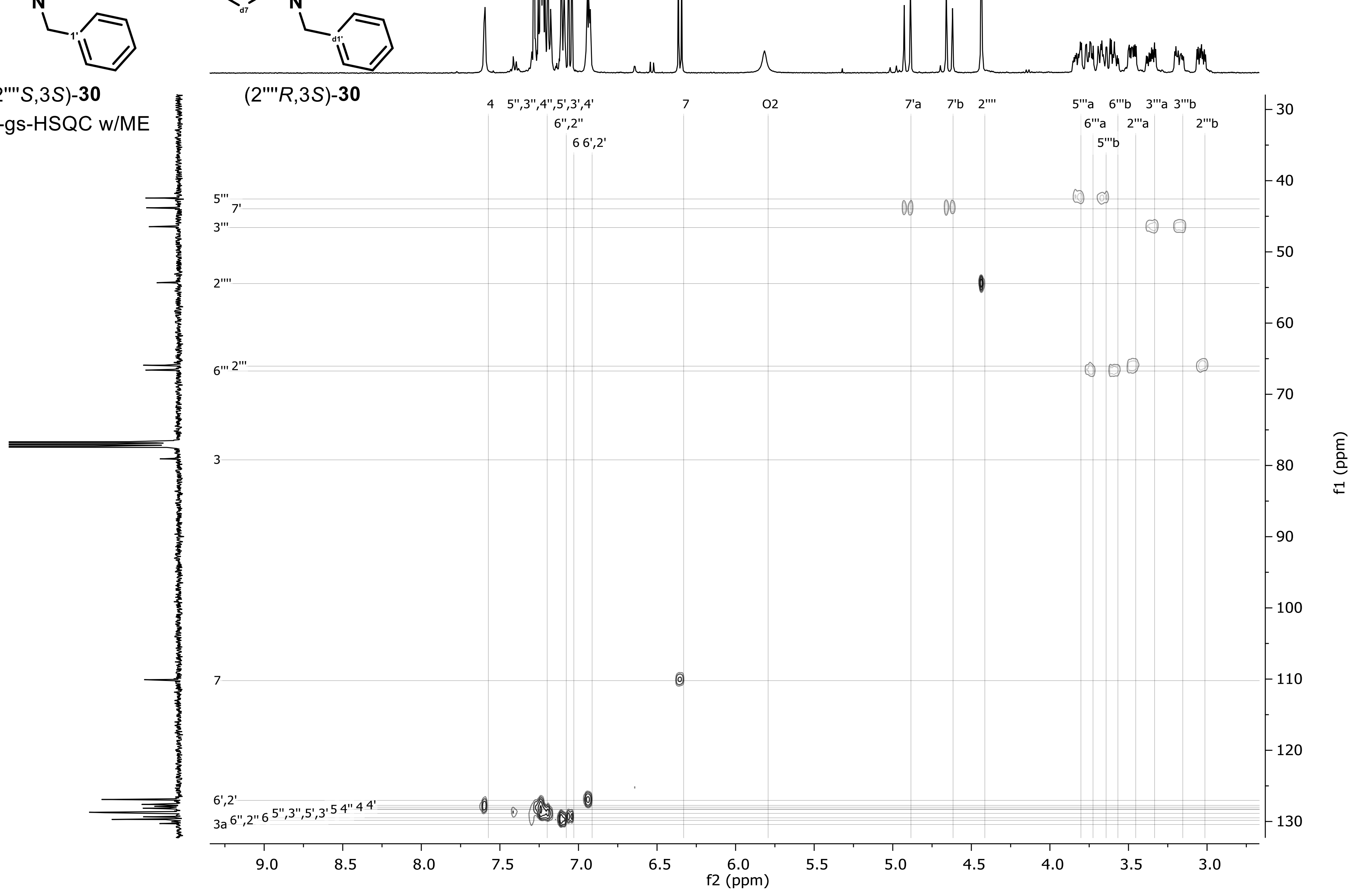

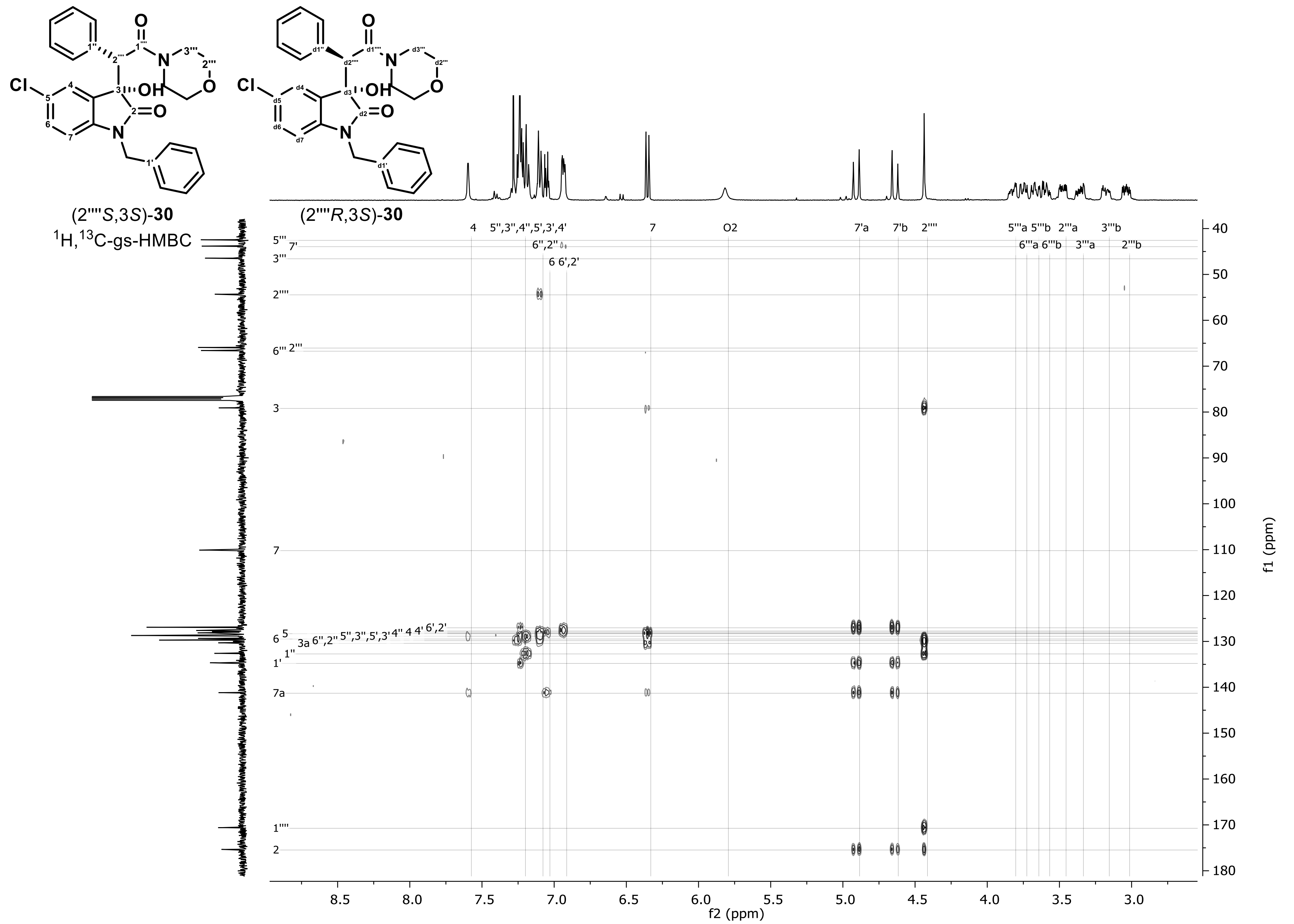
(2''''S,3S)-30
^{1}H,^{13}C-gs-HMBC
(2''''R,3S)-30
4 5'',3'',4'',5',3',4' 7 O2 7'a 7'b 2''''
5'''a 5'''b 2'''a 3'''b
6'''a 6'''b 3'''a 2'''b
6'',2'' 6 6',2'
5''' 7' 3''' 2'''' 6''' 2''' 3 7 6 5 3a 6'',2'' 5'',3'',5',3' 4'' 4 4' 6',2' 1'' 1' 7a 1'''' 2
f2 (ppm)
f1 (ppm)

y) 1-(*p*-^{t}Butylbenzyl)-3-hydroxy-3-(2-morpholino-2-oxo-1-phenylethyl)indolin-2-one (31)

To a 25 ml round bottomed flask was added phenylacetic anhydride (95.3 mg, 0.375 mmol), *N*-(*p-tert*-butylbenzyl)isatin (73.3 mg, 0.250 mmol), and (2*S*,3*R*)-HyperBTM (3.9 mg, 0.012 mmol). The mixture was cooled to 0 °C and CH_2Cl_2 (6.0 ml, 0.04 M) and Hünig's base (54 µl, 0.312 mmol) were added. The mixture was stirred at 0 °C for 3 h. Morpholine (65 µl, 0.750 mmol) was added and the reaction was left to be stirred overnight at room temperature. The solvent was removed under reduced pressure. Purification by column chromatography (Hexane:EtOAc 7:3 → 2:3) gave the title compound as yellow solid (62.1 mg, 0.125 mmol, 50%).

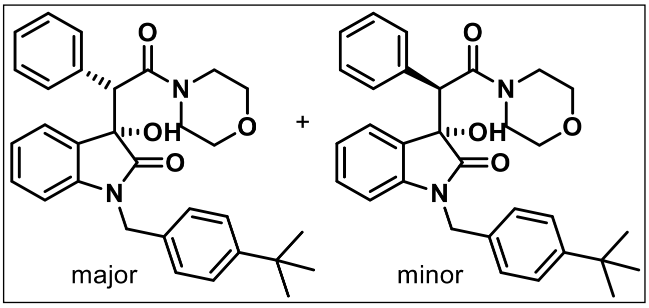

Major (2''''*S*,3*S*)-**31** and **minor** (2''''*R*,3*S*)-**31** analysed as an 87:13 mixture of diastereomers. **m.p.** 124 – 126 °C, **Chiral HPLC analysis** (Chiralcel® AD-H 85:15 Hexane:IPA, flow rate 2.0 ml·min^{-1}, 211 nm, 40 °C) t_R (2*R*,3*R*)-**31** 6.9 min, t_R (2*S*,3*S*)-**31** 16.4 min, <1:99 e.r.; t_R (2*S*,3*R*)-**31** 7.9 min, t_R (2*R*,3*S*)-**31** 36.3 min; signal not resolved; α_D^{20} = +66.8 (*c* 0.7, $CHCl_3$); ν_{max} (thin film) 3395 (s, br, OH), 3057 (w), 3030 (w), 2963 (m), 2907 (w), 2862 (w), 1717 (s, C=O), 1611 (s, C=O), 1516 (w), 1487 (m), 1468 (s), 1435 (m), 1416 (w), 1362 (m), 1300 (m), 1267 (m), 1256 (m), 1225 (m), 1175 (m), 1111 (s), 1069 (w), 1034 (w), 1018 (w), 970 (w), 908 (s), 862 (w), 843 (w), 812 (w), 802 (w), 754 (s); Not all NMR signals of the minor diastereomer could be resolved for **^{1}H NMR** (500 MHz, $CDCl_3$) δ_H 7.37 – 7.32 (0.39H, ArC*H*), 7.32 – 7.26 (ArC^4*H* major; ArC*H*), 7.26 – 7.23 (1.74H, m, ^{t}BuArC^{3,5}*H*), 7.23 – 7.19 (0.87H, m, PhC^4*H*), 7.17 – 7.10 (3.87H, m, ArC*H*), 7.08 (1H, app td, $^3J_{HH}$ = 7.7 Hz, $^4J_{HH}$ = 1.3 Hz, ArC^6*H*), 6.98 (1.74H, app d, $^3J_{HH}$ = 8.2 Hz, ^{t}BuArC^{2,6}*H*), 6.88 (1H, app td, $^3J_{HH}$ = 7.6 H, $^4J_{HH}$ = 1.1 Hz, ArC^5*H*, both), 6.66 (0.13H, s, O*H*), 6.65 (0.13H, d, $^3J_{HH}$ = 7.8 Hz, ArC^7*H*), 6.53 (0.87H, $^3J_{HH}$ = 7.6 Hz, ArC^7*H*), 6.07 (0.87H, s, O*H*), 4.94 (0.13H, d, $^2J_{HH}$ = 15.8 Hz, PhC^{1}C*H*$_a$H$_b$N), 4.84 (0.87H, d, $^2J_{HH}$ = 15.7 Hz, PhC^{1}C*H*$_a$H$_b$N), 4.64 (0.87H, d, $^2J_{HH}$ = 15.7 Hz, PhC^{1}CH$_a$*H*$_b$N), 4.64 (0.13H, d, $^2J_{HH}$ = 15.8 Hz, PhC^{1}CH$_a$*H*$_b$N), 4.43 (0.13H, s, PhC^{1}C*H*), 4.29 (0.87H, s, PhC^{1}C*H*), 3.77 (0.87H, ddd, $^2J_{HH}$ = 12.8 Hz, $^3J_{HH}$ = 5.7 Hz, 2.8 Hz, NC*H*$_c$H$_d$), 3.74 – 3.69 (1H, m, OC*H*$_c$H$_d$, major; ½ CH_2 minor), 3.66 (1H, ddd, $^2J_{HH}$ = 12.8 Hz, $^3J_{HH}$ = 6.9 Hz, 2.8 Hz, NCH$_c$*H*$_d$, major, ½ C*H*$_2$ minor), 3.55 (0.87H, ddd, $^2J_{HH}$ = 10.5Hz, $^3J_{HH}$ = 6.9 Hz, 2.8 Hz, OCH$_c$*H*$_d$), 3.46 (1.13 H, ddd, $^2J_{HH}$ = 11.6 Hz, $^3J_{HH}$ = 6.2 Hz, 3.0 Hz, OC*H*$_a$H$_b$, major; 2× ½ CH_2 minor), 3.42 – 3.37 (0.17H, m, OC*H*$_a$H$_b$), 3.36 (0.87H, ddd, $^2J_{HH}$ = 13.4 Hz, $^3J_{HH}$ = 7.1 Hz, 3.0 Hz, NC*H*$_a$H$_b$), 3.28 (0.13H, ddd, $^2J_{HH}$ = 13.4 Hz, $^3J_{HH}$ = 7.7 Hz, 3.1 Hz, NC*H*$_a$H$_b$), 3.19 (0.87H, ddd, $^2J_{HH}$ = 13.4 Hz, $^3J_{HH}$ = 6.2 Hz, 3.0 Hz, NCH$_a$*H*$_b$), 3.12 (0.13H, ddd, $^2J_{HH}$ = 13.4 Hz, $^3J_{HH}$ = 5.5 Hz, 3.1 Hz, NCH$_a$*H*$_b$), 3.04 (0.87H, ddd, $^2J_{HH}$ = 11.6 Hz, $^3J_{HH}$ = 7.1 Hz, 3.0 Hz, OCH$_a$*H*$_b$), 2.95 (0.13H, ddd, $^2J_{HH}$ = 11.1 Hz, $^3J_{HH}$ = 7.6 Hz, 2.9 Hz, OCH$_a$*H*$_b$), 1.28 (9H, s, C(C*H*$_3$)$_3$, both); **^{13}C {^{1}H} NMR** (125 MHz, $CDCl_3$) δ_C 176.1 (*C*(O)NCH$_2$^{t}BuAr, both), 171.0 (*C*(O)N(CH$_2$CH$_2$)$_2$O, major), 169.5 (*C*(O)N(CH$_2$CH$_2$)$_2$O, minor), 150.5 (ArC^{4t}Bu, major), 150.3 (Ar*C*^{4t}Bu, minor), 142.8 (Ar*C*^{7a}, major), 142.6 (Ar*C*^{7a}, minor), 133.5 (Ph*C*^{1}, minor), 133.1 (Ph*C*^{1}, major), 132.7 (^{t}BuAr*C*^{1}, minor), 132.4 (^{t}BuAr*C*^{1}, major), 130.2 (Ph*C*^{2,6}H, minor), 129.9 (Ph*C*^{2,6}H, major), 129.7 (Ar*C*^{3a}, minor), 129.6 (Ar*C*^{6}H, major), 128.6 (Ph*C*^{3,5}H, both), 128.5 (Ar*C*^{3a}, major), 128.3 (Ar*C*), 128.0 (Ph*C*^{4}H, major), 127.3 (Ar*C*^{4}H, major), 127.1 (^{t}BuAr*C*^{2,6}H, minor), 127.0 (^{t}BuAr*C*^{2,6}H, major), 125.6 (^{t}BuArC^{3,5}H, major), 124.6 (^{t}BuArC^{3,5}H, minor), 122.7 (Ar*C*^{5}H, major), 122.4 (Ar*C*^{5}H, minor), 109.5 (Ar*C*^{7}H, minor), 109.2 (Ar*C*^{7}H, major), 78.9 (*C*OH, major), 77.2 (*C*OH, minor), 66.7 (O*C*H$_c$H$_d$, both), 66.1 (O*C*H$_a$H$_b$, both), 55.4 (PhC^1*C*H, minor), 53.5 (PhC^1*C*H, major), 46.6 (N*C*H$_a$H$_b$, major), 46.4 (N*C*H$_a$H$_b$, minor), 43.7 (^{t}BuArC^1*C*H$_a$H$_b$, minor), 43.5 (^{t}BuArC^1*C*H$_a$H$_b$, major), 42.5 (N*C*H$_c$H$_d$, major), 42.2 (N*C*H$_c$H$_d$, minor), 34.6 (*C*(CH$_3$)$_3$, both), 31.4$_4$ (C(*C*H$_3$)$_3$, major), 31.4$_1$ (C(*C*H$_3$)$_3$, minor); ***m/z*** (ESI$^+$) 147 (10%), 198 (5%), 200 (5%), 230 (16%), 232 (16%), 361 ([M-C$_6$H$_4$^{t}Bu+2H]$^+$ 21%), 481 ([M-OH]$^+$ 19%), 499 ([M+H]$^+$ 100%), 500 ([M(^{13}C)+H]$^+$ 34%), 501 ([M(^{13}C$_2$)+H]$^+$ 6%), 521 ([M+Na]$^+$ 8%), 859 ([M-^{t}BuBn-H$_2$O]$^+$ 8%); **HRMS** (ESI$^+$) *m/z* calcd for [M+H]$^+$ $C_{31}H_{35}O_4N_2$ 499.2591, found 499.2584 (−1.5 ppm).

(±)-anti-**31** + (±)-syn-**31**

PDA Ch1 211nm

Peak#	Ret. Time	Area%
1	6.939	42.953
2	7.868	6.329
3	16.438	43.823
4	36.344	6.896
Total		100.000

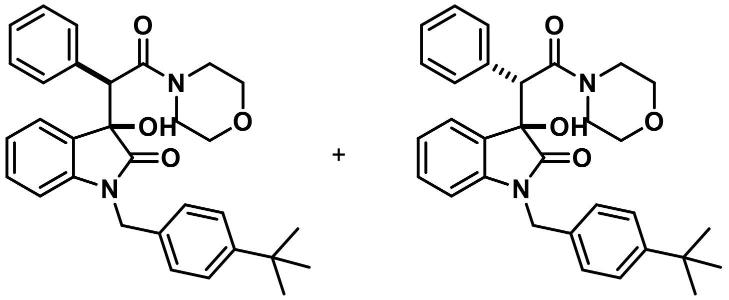

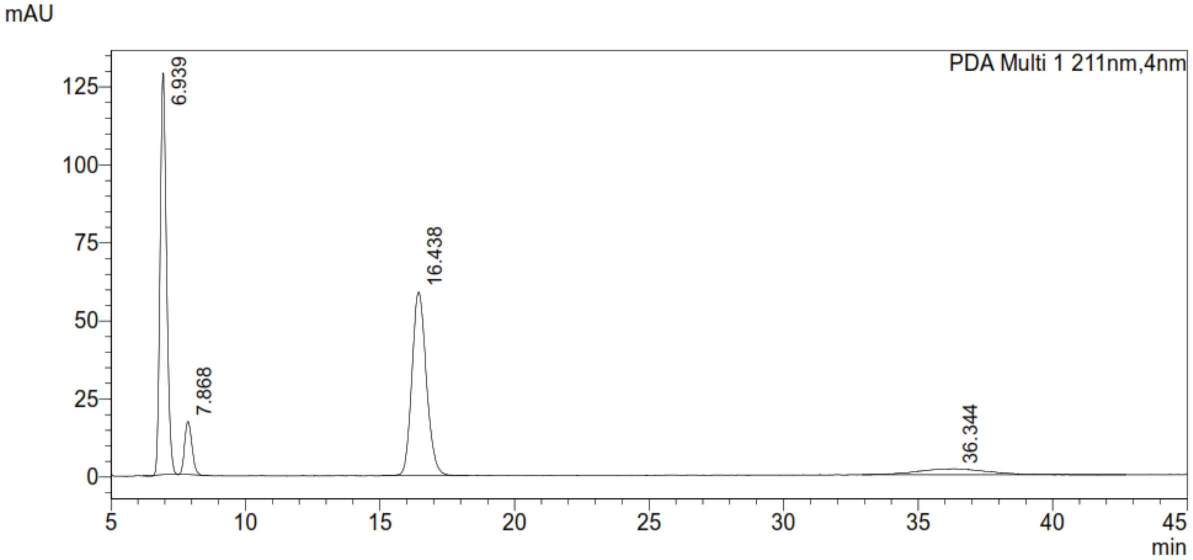

(2''''*S*,3*S*)-**31** + (2''''*R*,3*S*)-**31**

PDA Ch1 211nm

Peak#	Ret. Time	Area%
1	6.940	0.493
2	7.852	0.155
3	16.370	99.164
4	33.322	0.187
Total		100.000

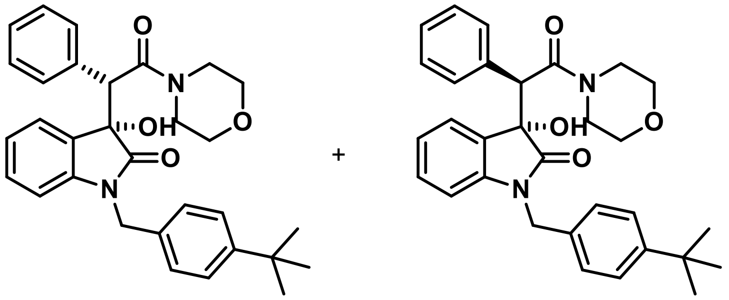

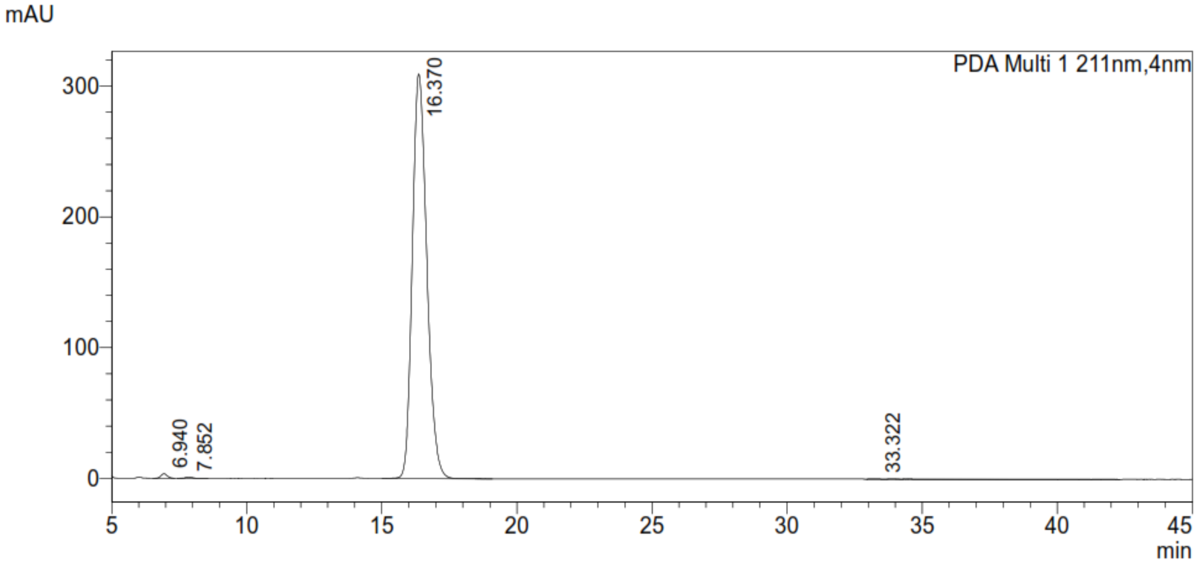

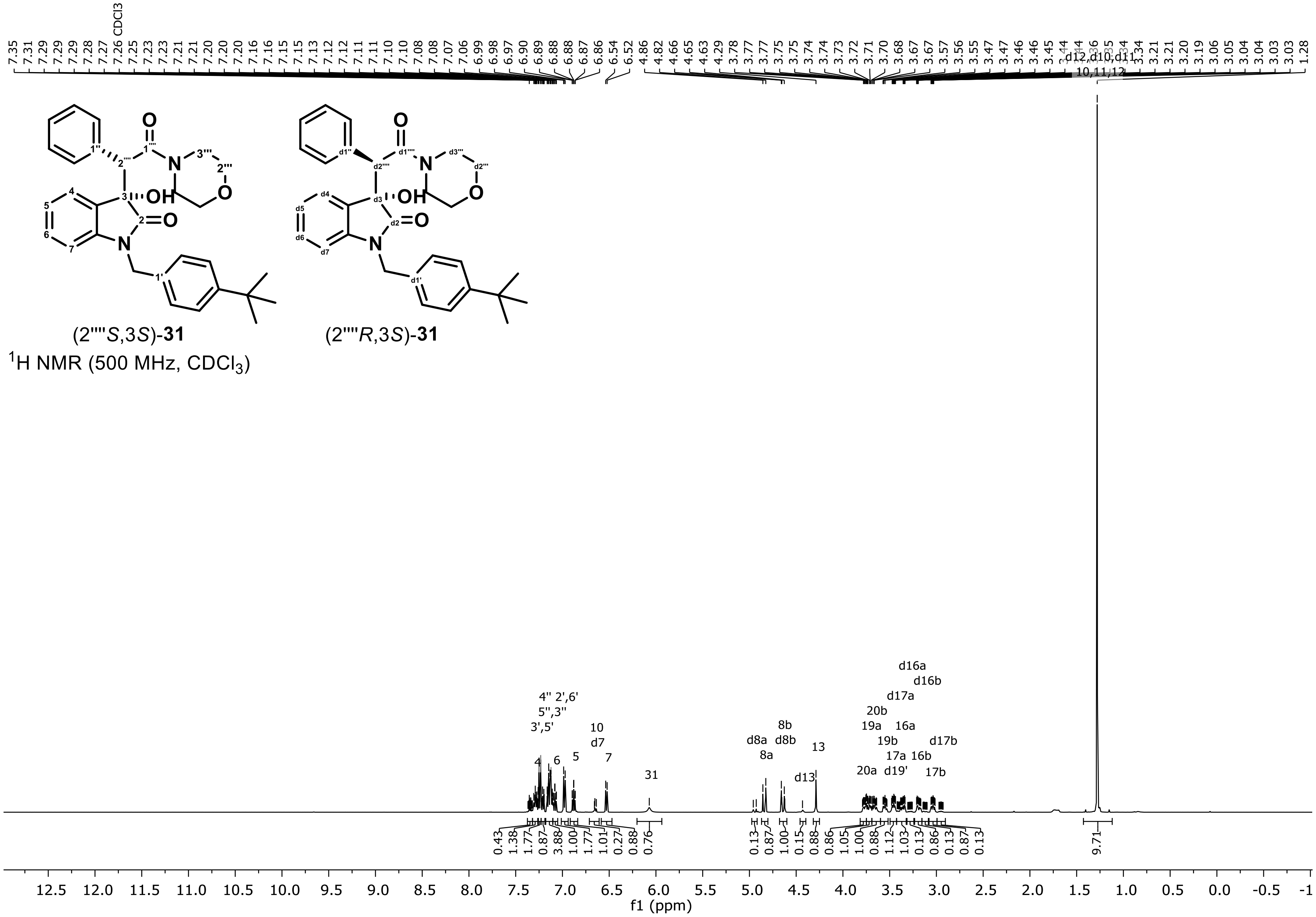
(2''''S,3S)-31
^{1}H NMR (500 MHz, CDCl$_3$)
(2''''R,3S)-31
f1 (ppm)

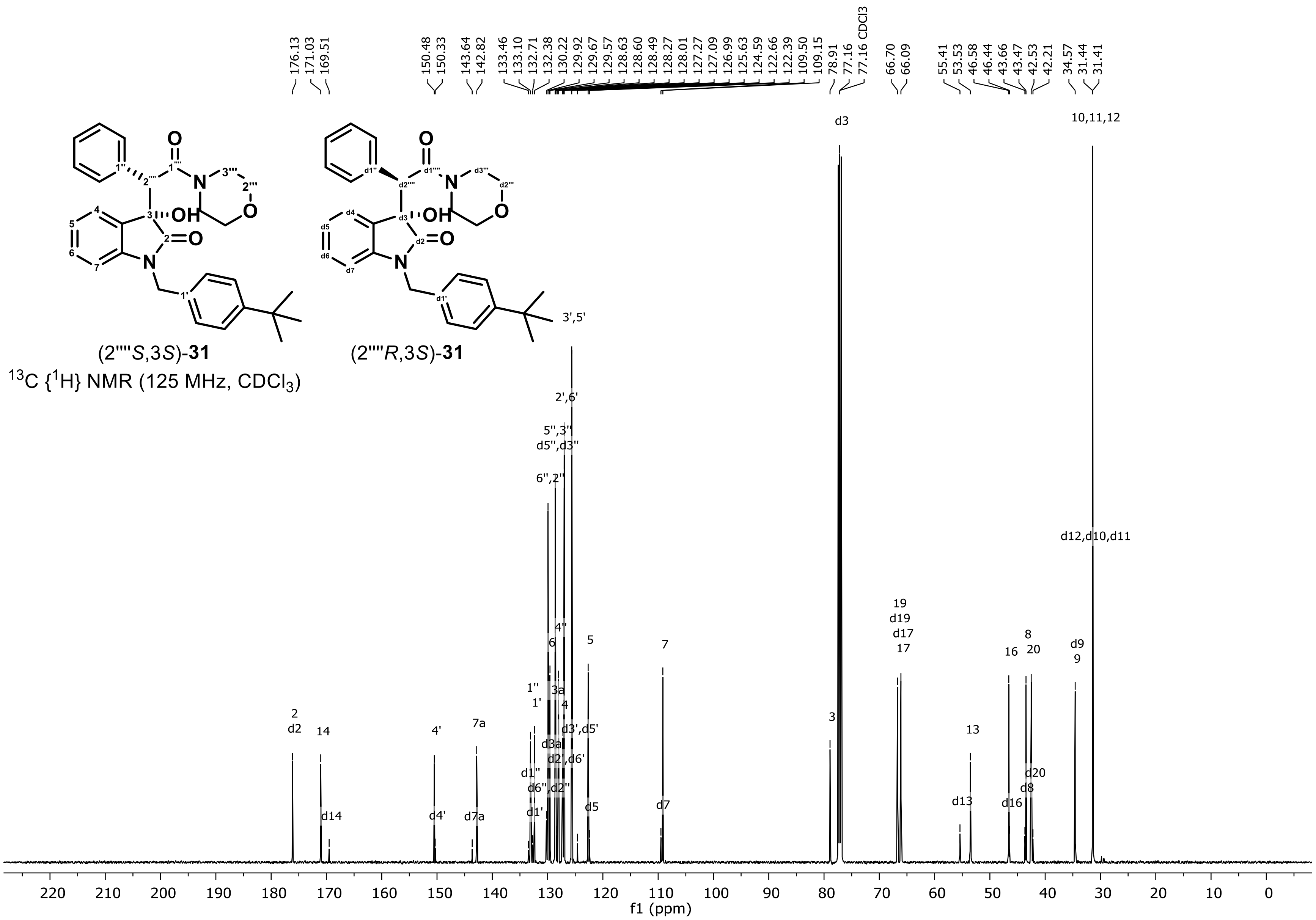

(2''''S,3S)-31 (2''''R,3S)-31

$^{13}C\ \{^{1}H\}$ NMR (125 MHz, $CDCl_3$)

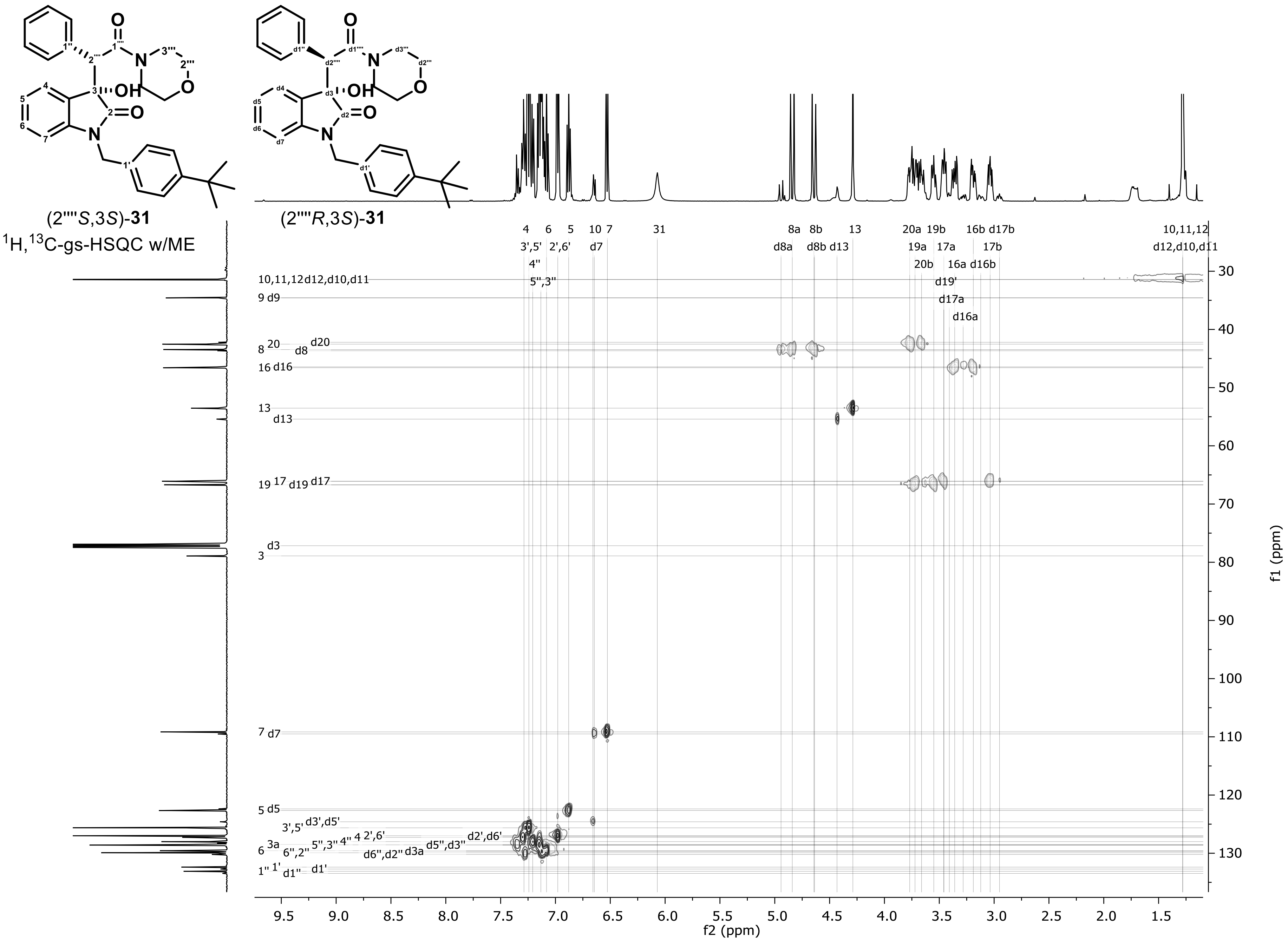

(2''''S,3S)-**31**

$^{1}H,^{13}C$-gs-HSQC w/ME

(2''''R,3S)-**31**

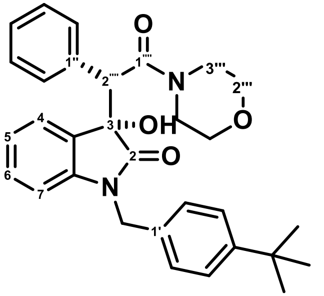

(2''''S,3S)-**31**

^{1}H,^{13}C-gs-HMBC

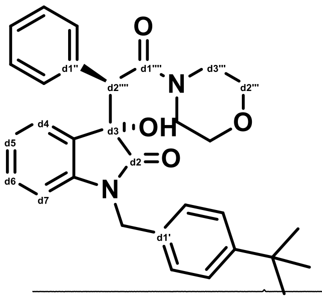

(2''''R,3S)-**31**

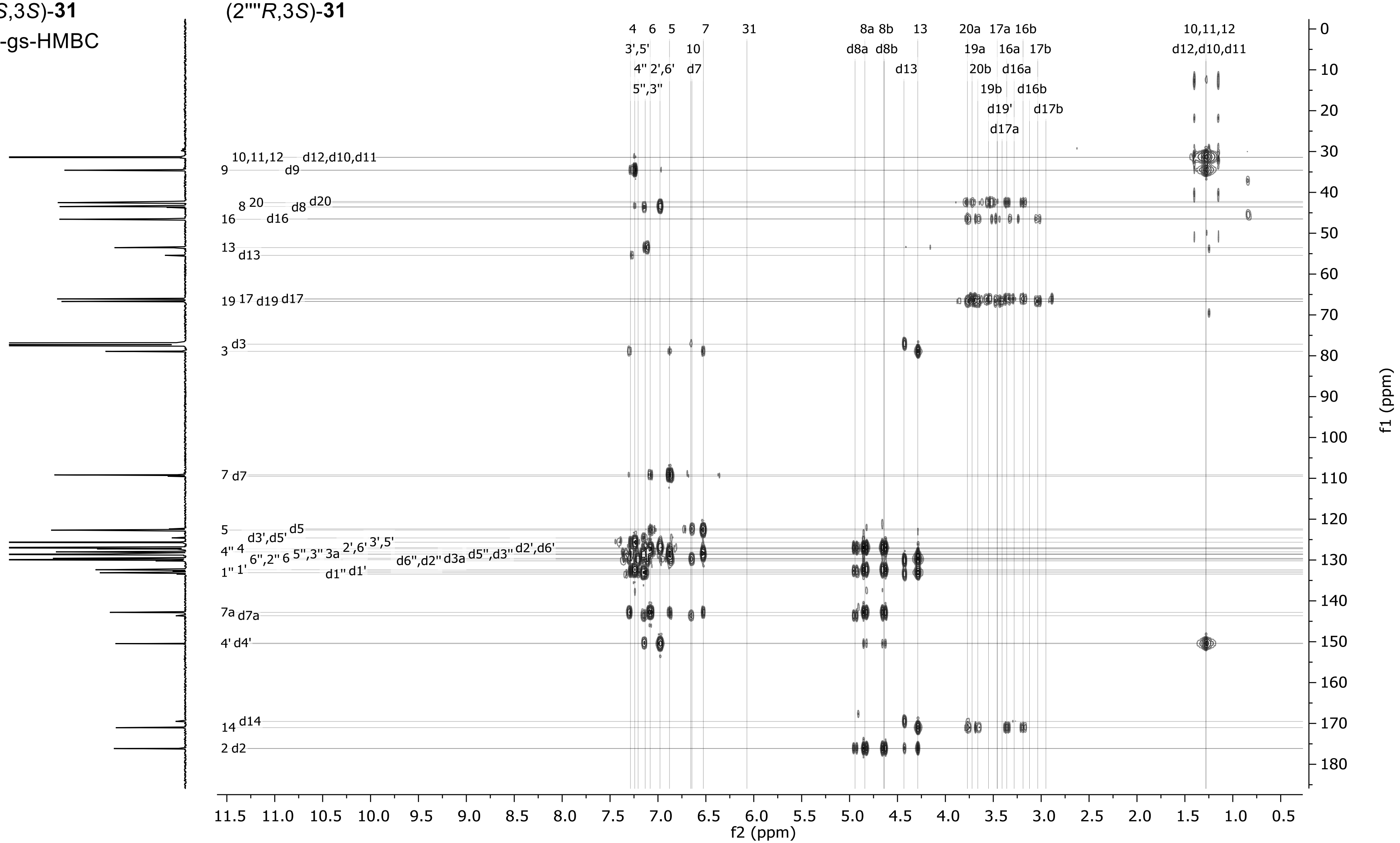

IV. Syntheses of Anhydrides

a) Phenylacetic anhydride (S1)

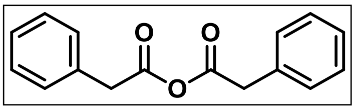

To a solution of 2-phenylacetic acid (680 mg, 5.00 mmol) in toluene (15.0 ml, 0.3 M) was added *N*,*N*′-dicyclohexylcarbodiimide (516 mg, 2.50 mmol). The mixture was stirred for 30 minutes at room temperature and was then filtered through Celite®. The solvent was removed and recrystallisation from Et_2O gave the title compound (447 mg, 1.76 mmol, 70%) as a white crystalline solid with data in accordance with the literature.[15] **m.p.** 68–70 °C (Et_2O) {Lit.[15b] 70-72 °C}; **^{1}H NMR** (500 MHz, $CDCl_3$) δ_H 7.37 – 7.28 (6H, m, 2 × $PhC^{3',4',5'}H$), 7.19 – 7.24 (4H, m, 2 × $PhC^{2',6'}H$), 3.73 (4H, s, 2 × CH_2).

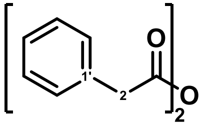

S1

^{1}H NMR (500 MHz, $CDCl_3$)

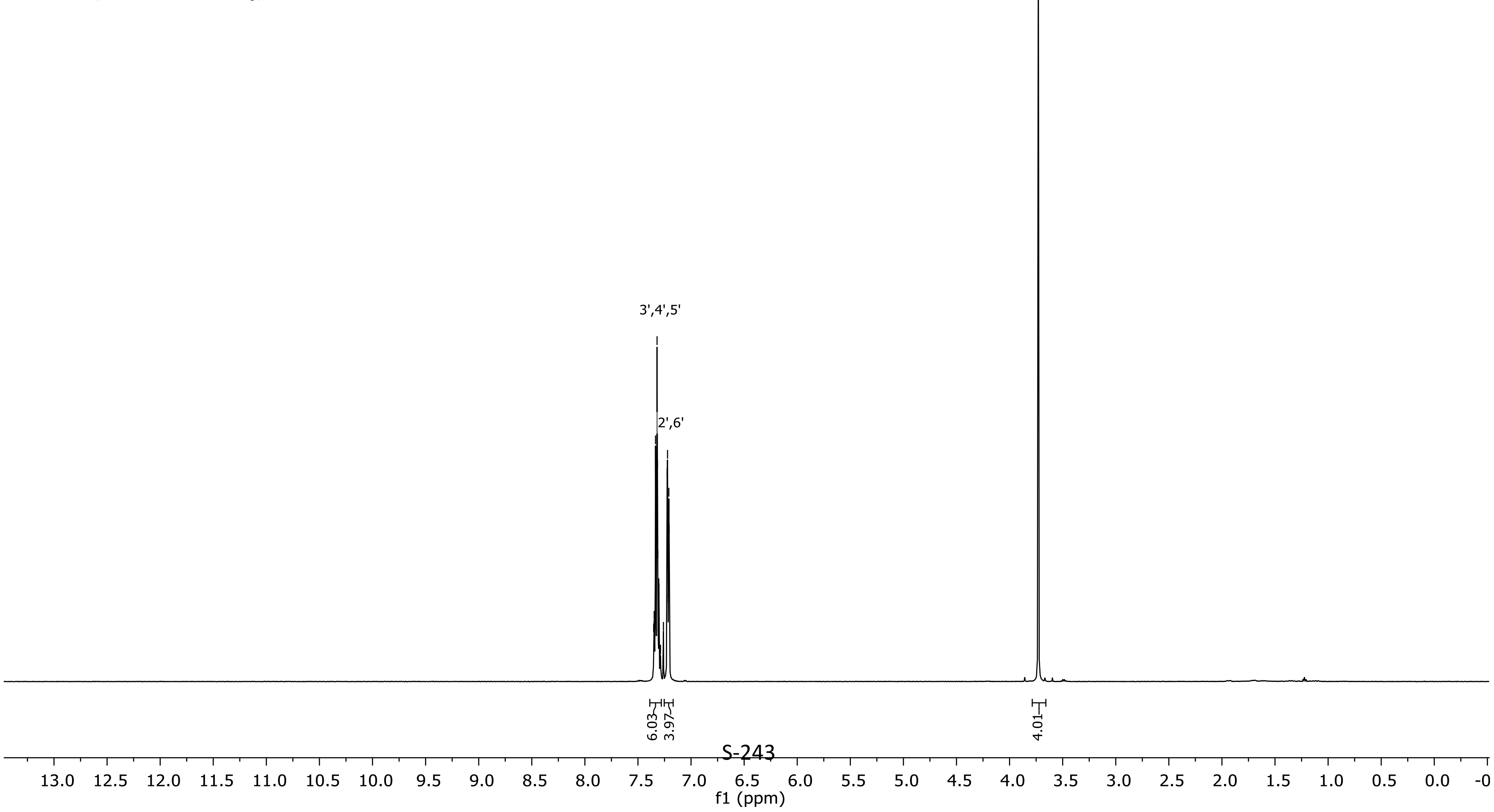

b) ***para*-Anisylacetic anhydride (S2)**

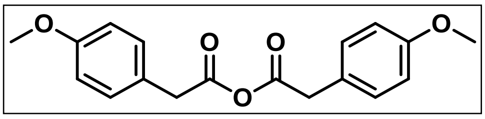

To a solution of 2-(*p*-anisyl)acetic acid (2.50 g, 15.0 mmol) in toluene (45.0 ml, 0.3 M) was added *N*,*N'*-dicyclohexylcarbodiimide (1.50 g, 7.50 mmol). The mixture was stirred for 30 minutes at room temperature and was then filtered through Celite®. The solvent was removed and recrystallisation from Et_2O gave the title compound (2.09 g, 6.65 mmol, 89%) as a white crystalline solid with data in accordance with the literature.[15b, 16] **m.p.** 64 °C (PhMe) {Lit.[15a] 60 – 62 °C}, {Lit.[15b] 61 – 62 (Et_2O)}, {Lit.[16b] 76 – 78 (Petrol)}, {Lit.[16a] 77 – 78 °C (Et_2O)}; **^{1}H NMR** (500 MHz, $CDCl_3$) δ_H 7.16 – 7.12 (4H, m, 2 × ArC$^{2',6'}$*H*) , 6.89 – 6.85 (4H, m, 2 × ArC$^{3',5'}$*H*), 3.83 (6H, s, 2 × OCH_3), 3.68 (4H, s, 2 × CH_2).

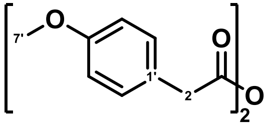

S2

^{1}H NMR (500 MHz, $CDCl_3$)

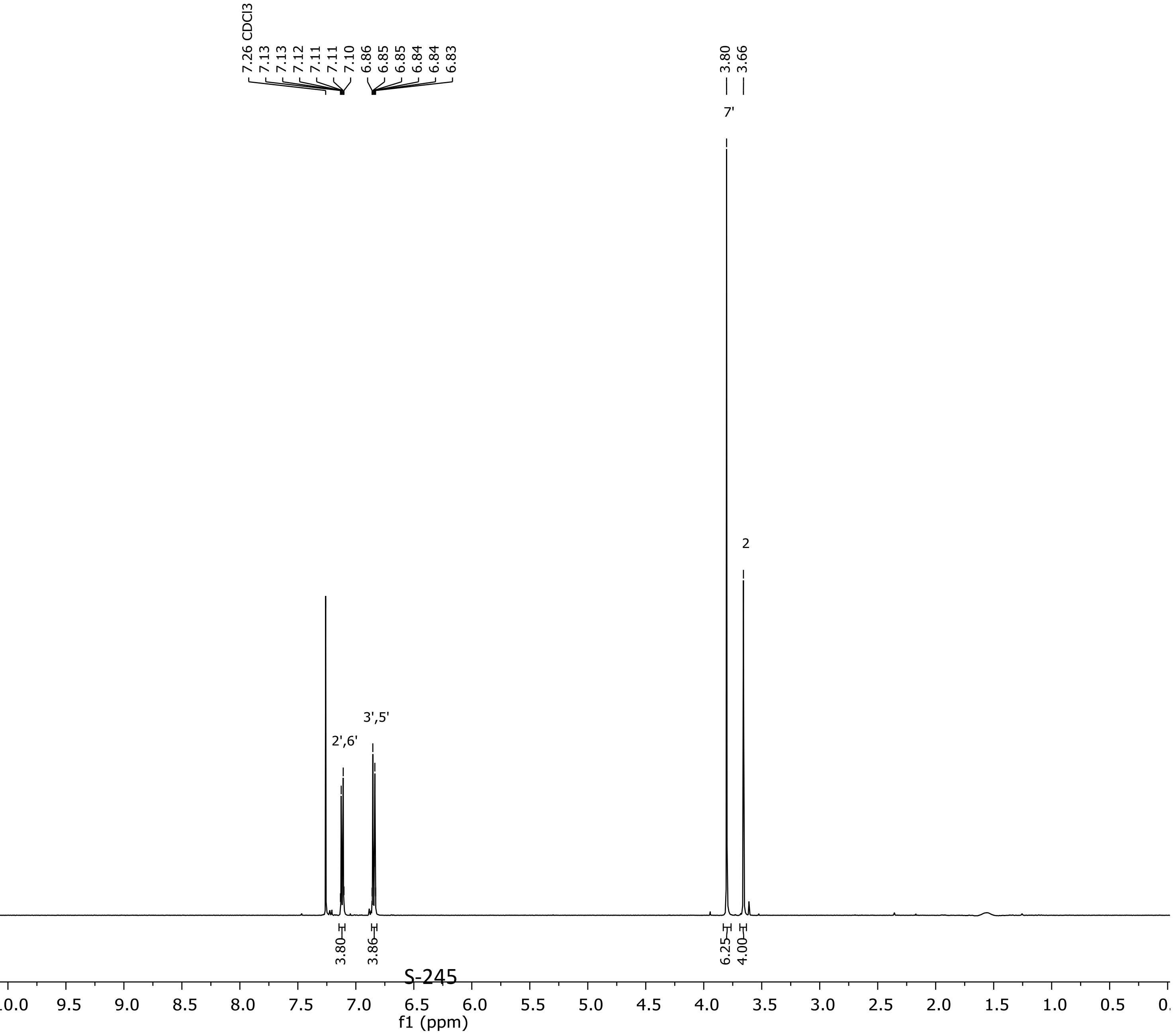

c) para-Tolylacetic anhydride (S3)

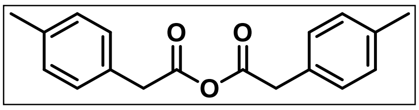

To a solution of 2-(*p*-tolyl)acetic acid (831 mg, 5.0 mmol) in toluene (15.0 ml, 0.3 M) was added *N*,*N*′-dicyclohexyl-carbodiimide (567 mg, 2.75 mmol) The mixture was stirred for 30 minutes at room temperature and was then filtered through Celite®. The solvent was removed and recrystallisation from toluene gave the title compound (708 mg, 2.51 mmol, quant.) as a white crystalline solid with data in accordance with the literature.[15a, 16c] **m.p.** 59 – 61 °C (PhMe) {Lit.[16b] 56 – 57 °C}; **^{1}H NMR** (400 MHz, $CDCl_3$) δ_H 7.14 – 7.11 (4H, m, 2 × ArC$^{2',6'}$*H*), 7.11 – 7.07 (4H, m, 2 × ArC$^{3',5'}$*H*), 3.68 (4H, s, 2 × CH_2), 2.34 (6H, s, 2 × CH_3).

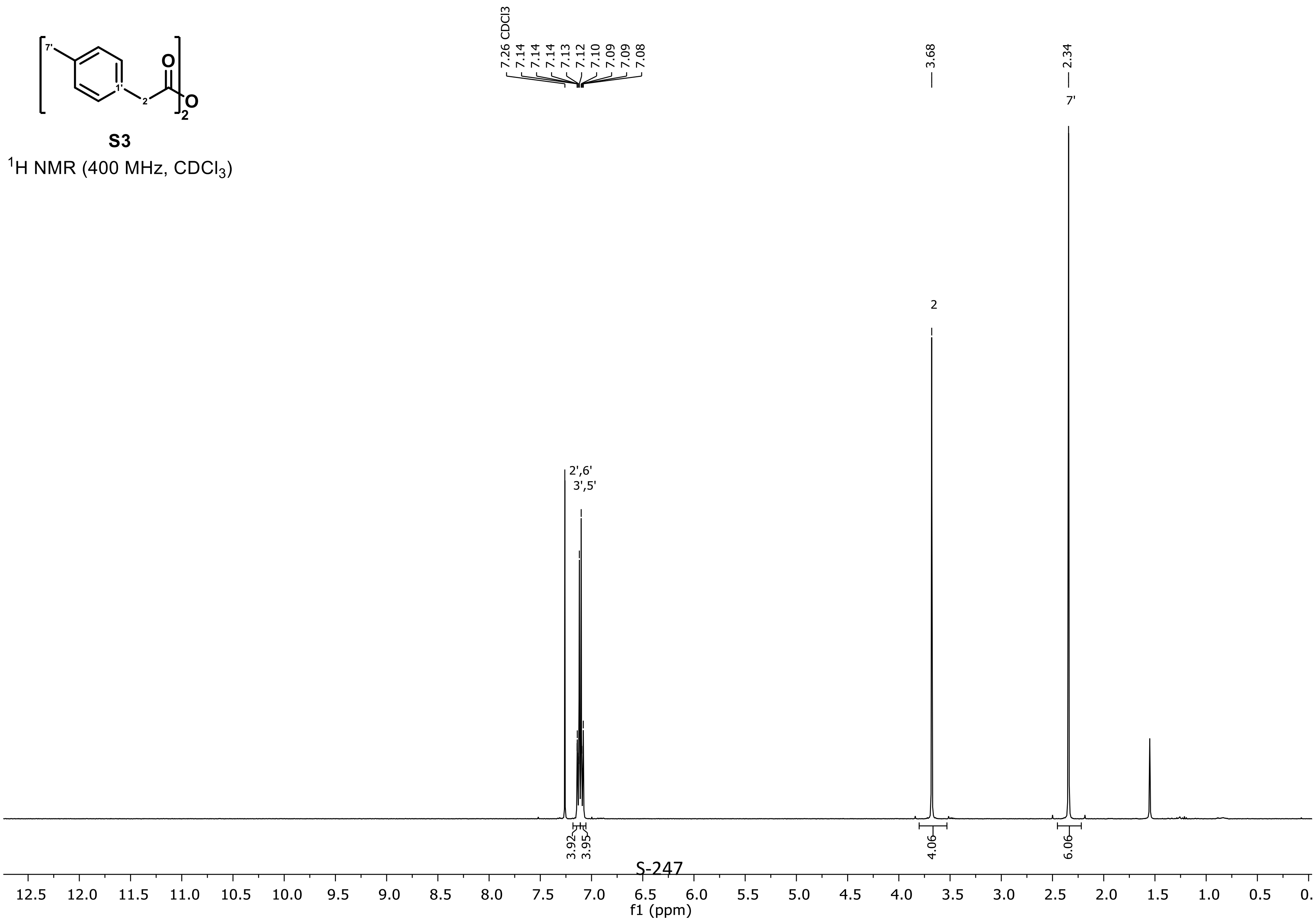

S3

^{1}H NMR (400 MHz, $CDCl_3$)

d) *para*-Chlorophenylacetic anhydride (S4)

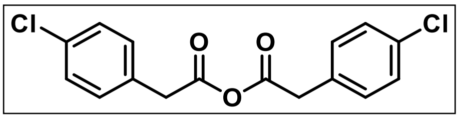

To a solution of 2-(*p*-chlorophenyl)acetic acid (853 mg, 2.75 mmol) in toluene (15.0 ml, 0.3 M) was added *N*,*N'*-dicyclohexylcarbodiimide (567 mg, 2.75 mmol) The mixture was stirred for 30 minutes at room temperature and was then filtered through Celite® to give the title compound (444 mg, 1.37 mmol, 55%) as a white crystalline solid with data in accordance with the literature.[15] **m.p.** 64 – 66 °C {Lit.[15b] 62 – 64 °C}; **^{1}H NMR** (400 MHz, $CDCl_3$) δ_H 7.32 – 7.27 (4H, m, 2 × ArC$^{3',5'}$*H*), 7.15 – 7.10 (4H, m, 2 × ArC$^{2',6'}$*H*), 3.70 (4H, s, 2 × CH_2).

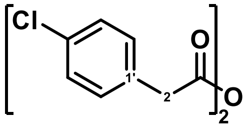

S4

^{1}H NMR (400 MHz, $CDCl_3$)

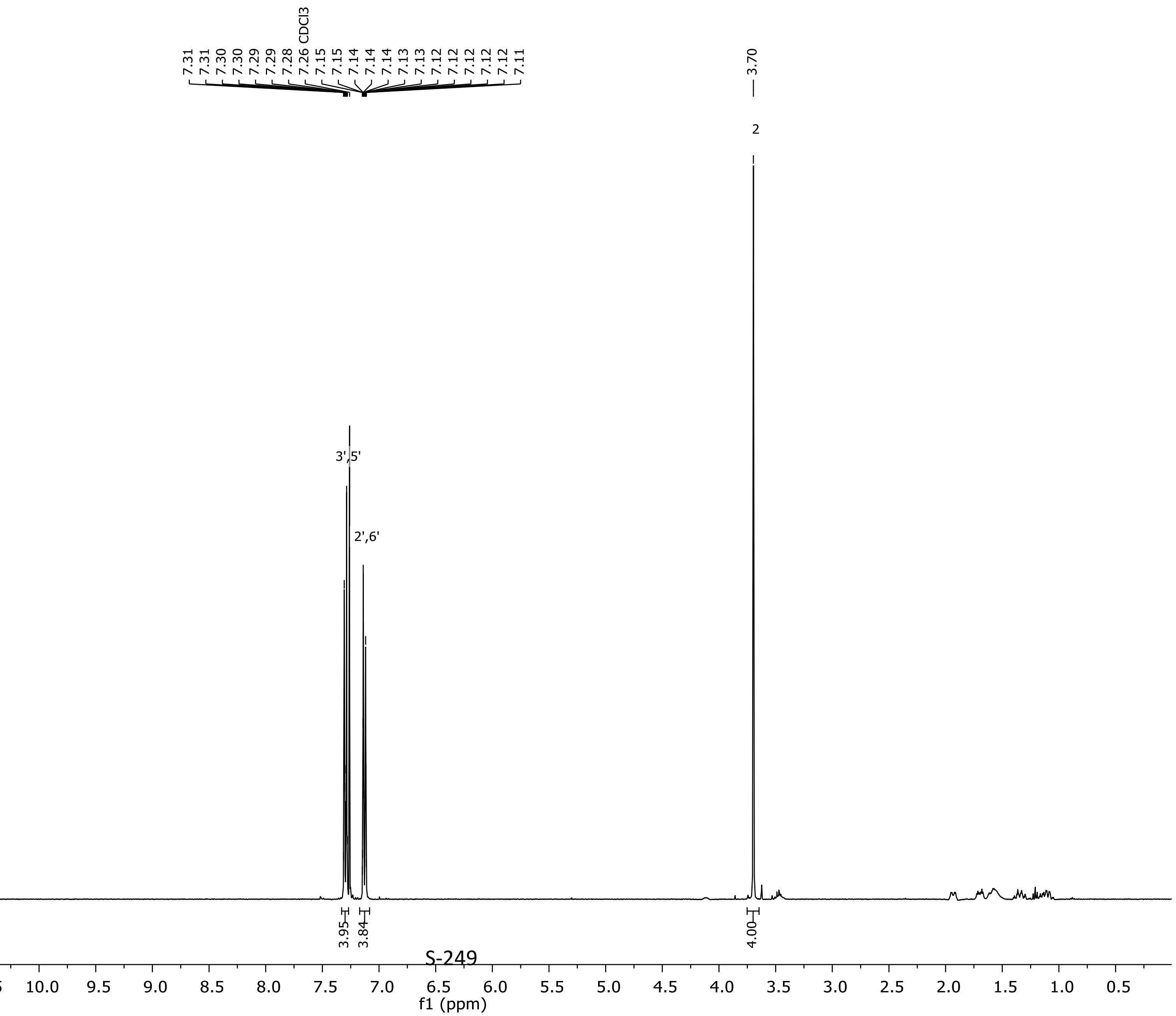

e) **(*para*-Bromophenyl)acetic anhydride (S5)**

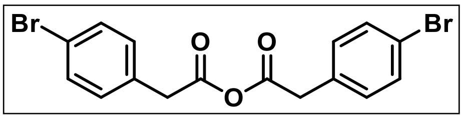

To a solution of 2-(*p*-bromophenyl)acetic acid (1.08 g, 5.0 mmol) in toluene (15.0 ml, 0.3 M) was added *N*,*N*′-dicyclohexylcarbodiimide (567 mg, 2.75 mmol) The mixture was stirred for 30 minutes at room temperature and was then filtered through Celite®. The solvent was removed and recrystallisation from toluene gave the title compound (1.03 g, 2.50 mmol, quant) as a white crystalline solid with data in accordance with the literature.[15] **m.p.** 80 – 82 °C (PhMe) {Lit.[15b] 75 – 77 °C}; **^{1}H NMR** (400 MHz, $CDCl_3$) δ_H 7.48 – 7.43 (4H, m, 2 × Ar$C^{3',5'}$*H*), 7.10 – 7.04 (4H, m, 2 × Ar$C^{2',6'}$*H*), 3.68 (4H, s, 2 × CH_2).

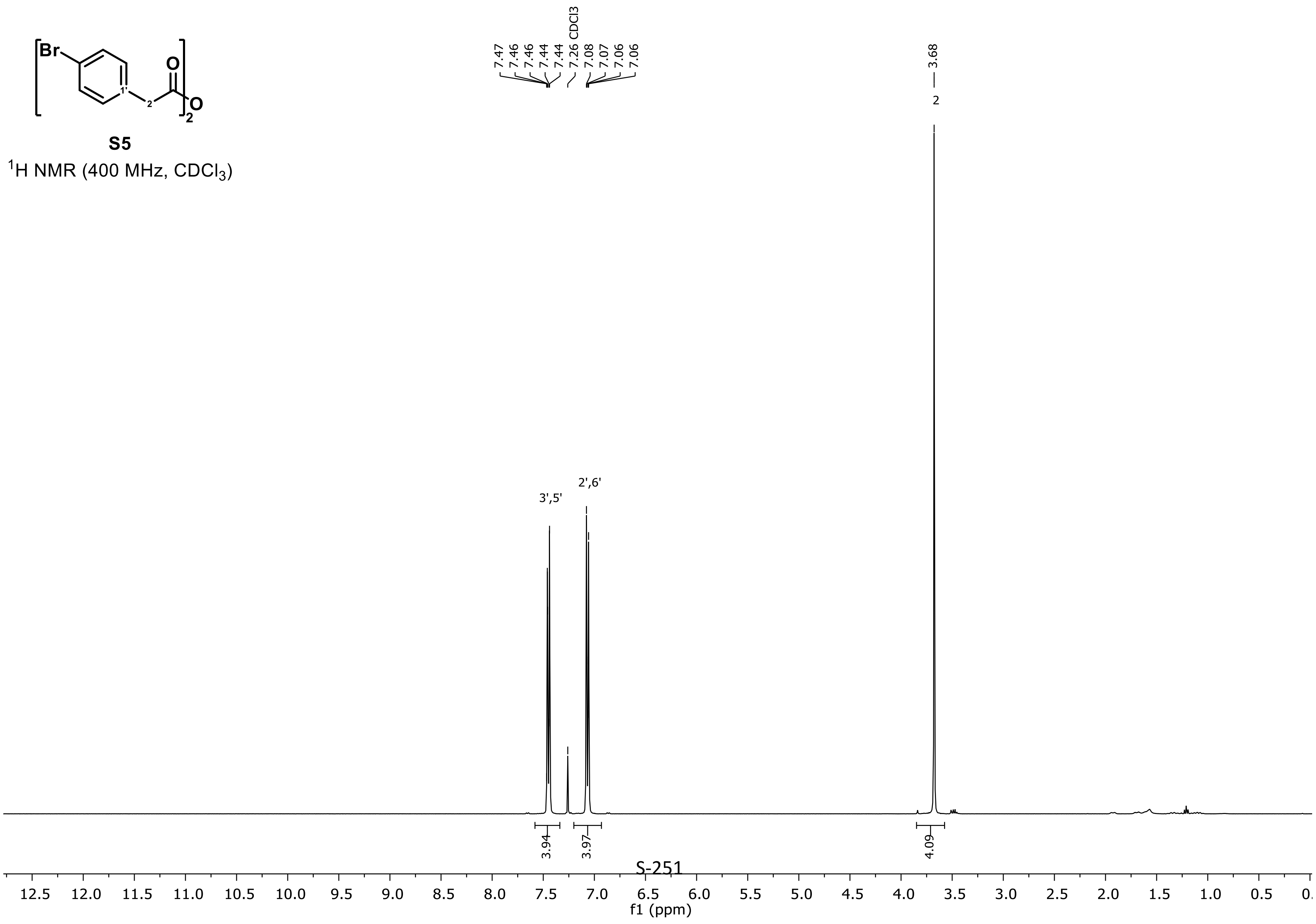
S5
^{1}H NMR (400 MHz, $CDCl_3$)
7.47
7.46
7.46
7.44
7.44
7.26 CDCl3
7.08
7.07
7.06
7.06
3.68
2
3',5'
2',6'
3.94
3.97
4.09
12.5
12.0
11.5
11.0
10.5
10.0
9.5
9.0
8.5
8.0
7.5
7.0
6.5
6.0
5.5
5.0
4.5
4.0
3.5
3.0
2.5
2.0
1.5
1.0
0.5
f1 (ppm)

f) *meta*-Bromophenylacetic anhydride (S6)

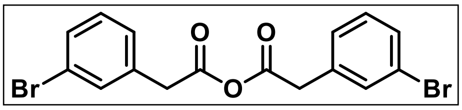

To a solution of 2-(m-bromophenyl)acetic acid (1.08 g, 5.0 mmol) in toluene (15.0 ml, 0.3 M) was added *N*,*N*'-dicyclohexylcarbodiimide (567 mg, 2.75 mmol) The mixture was stirred for 30 minutes at room temperature and was then filtered through Celite®. The solvent was removed and recrystallisation from toluene gave the title compound (1.05 g, 2.55 mmol, quant) as a white crystalline solid. **m.p.** 44 – 46 °C; **^{1}H NMR** (500 MHz, $CDCl_3$) δ_H 7.44 (2H, ddd, $^3J_{HH}$ = 7.9 Hz, $^4J_{HH}$ = 2.0 Hz, 1.2 Hz, ArC$^{4'}$*H*), 7.38 (2H, dd, $^4J_{HH}$ = 2.0 Hz, 1.6 Hz, ArC$^{2'}$*H*), 7.21 (2H, app t, $^3J_{HH}$ = 7.9 Hz, 2 × ArC$^{5'}$*H*), 7.15 (2H, ddd, $^3J_{HH}$ = 7.9 Hz, $^4J_{HH}$ = 1.6 Hz, 1.2 Hz, 2 × ArC$^{6'}$*H*), 3.70 (4H, s, 2 × CH_2).

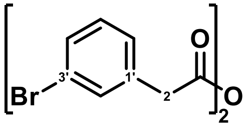

S6

^{1}H NMR (400 MHz, $CDCl_3$)

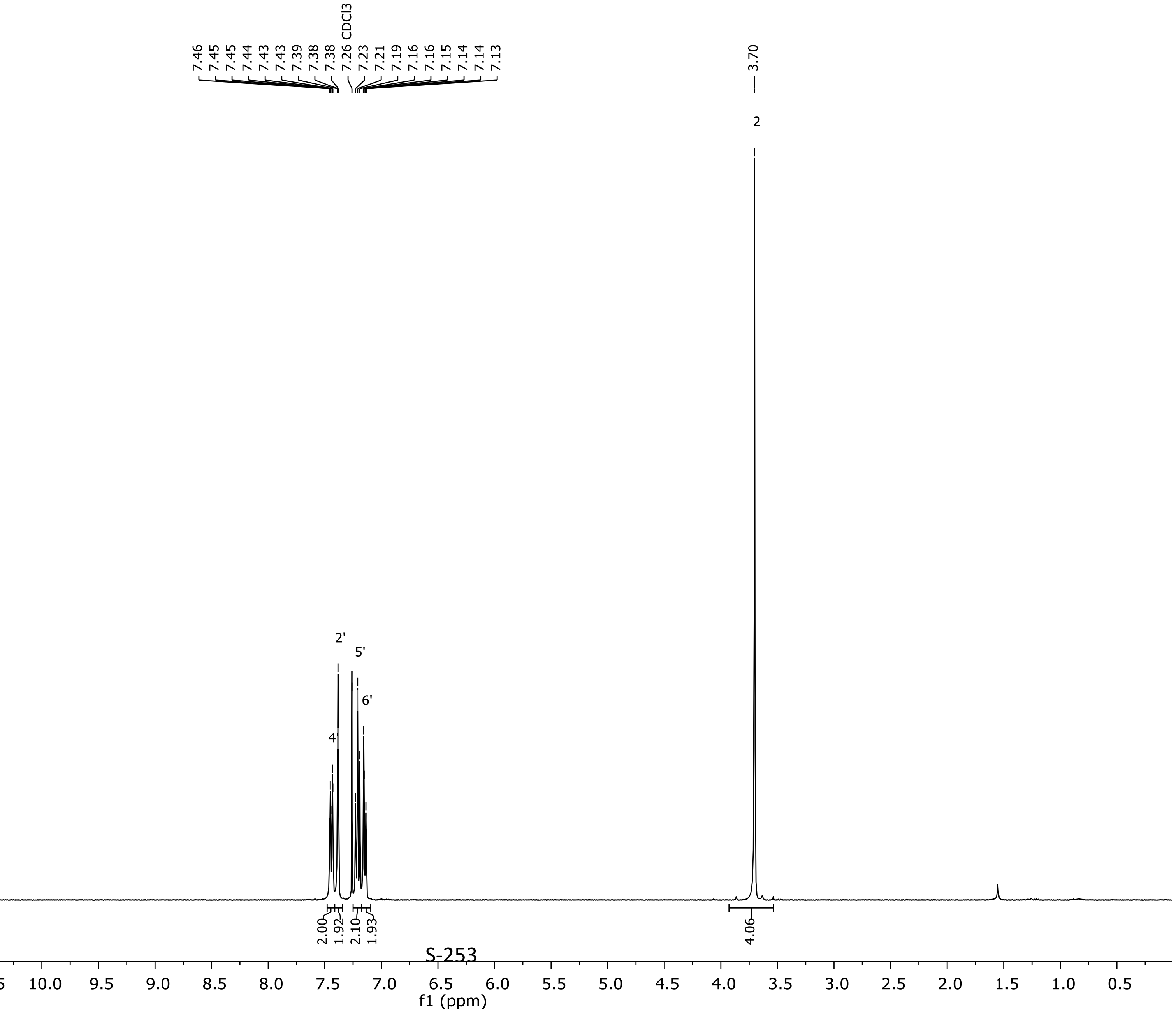

g) Hydratropic Anhydride (kk47-S7)

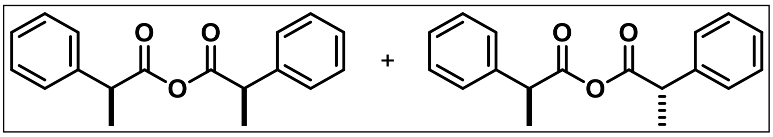

To a 250 ml round bottomed flask was added toluene (66.6 ml, 0.33 M), (±)-hydratropic acid (3.0 ml, 21.97 mmol) and *N*,*N'*-dicyclohexylcarbodiimide (2.49 g, 12.09 mmol). The mixture was stirred at room temperature for 30 minutes and was then filtered through Celite® eluting with toluene. The solvent was removed under reduced pressure and the crude material was purified by column chromatography (4/5 Geoffrey, Hexane:CH_2Cl_2 375:125 → 250:250 → 125:375) to give the title compound as a racemic 1:1 mixture of diastereomers and colourless oil (2.07 g, 7.34 mmol, 33%) with spectroscopic data in accordance with the literature.[17] **R*f*** 0.55 (Hexane:CH_2Cl_2 3:7); **^{1}H NMR** (400 MHz, $CDCl_3$) δ_H 7.24 – 7.32 (12H, m, 4 × PhC^{3,4,5}*H*), 7.08 – 7.15 (8H, m, 4 × PhC^{2,6}*H*), 3.70 (2H, q, $^3J_{HH}$ = 7.1 Hz, 2 × C*H*(Ph)Me), 3.68 (2H, q, $^3J_{HH}$ = 7.1 Hz, 2 × C*H*(Ph)Me), 1.45 (6H, d, $^3J_{HH}$ = 7.1 Hz, 2 × C*H*$_3$), 1.44 (6H, d, $^3J_{HH}$ = 7.1 Hz, 2 × C*H*$_3$).

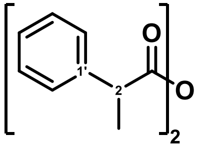

S7

^{1}H NMR (400 MHz, $CDCl_3$)

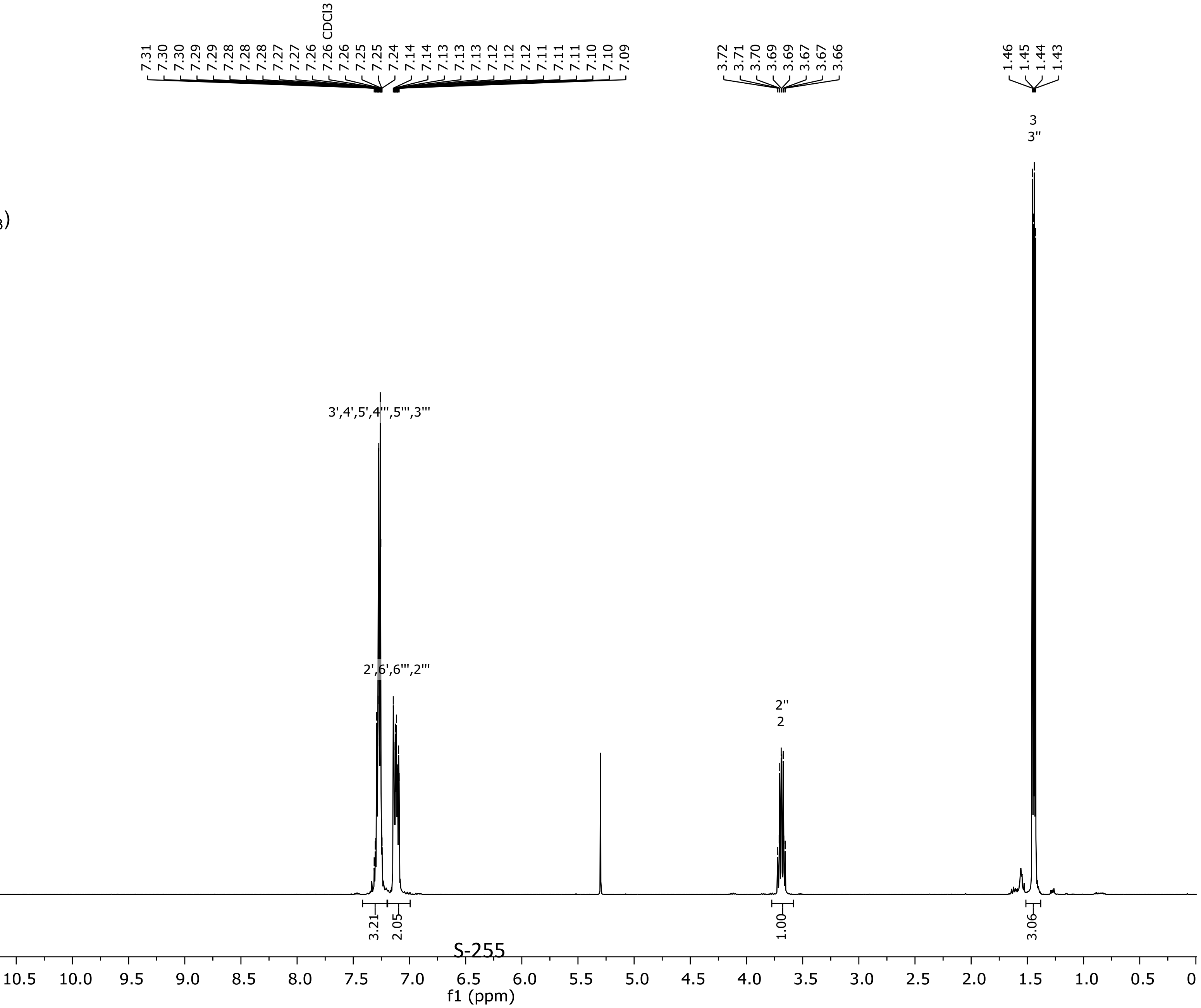

V. Syntheses of Isatins

a) N-Benzylisatin (S8)

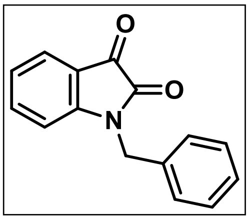

Following a literature procedure;[18] to a 250 ml round bottomed flask under N_2 was added DMF (20 ml, 0.25 M), isatin (736 mg, 5.00 mmol), NaH (60% dispersion in mineral oil, 260 mg, 6.50 mmol) at 0 °C. The reaction was stirred at this temperature for 0.5 h. BnBr (713 µl, 6.00 mmol) was added, and the reaction was left to be stirred at room temperature for 2 h. Water (100 ml) was added and the resulting precipitated was filtered off. The precipitated was dissolved in CH_2Cl_2 and washed with brine. The combined organic layers were dried over $MgSO_4$ anhydrous, filtered, and concentrated *in vacuo*. Purification by recrystallisation from EtOAc (evaporation or precipitation with hexane) gave the title compound as red crystalline solid (1210 mg, 5.10 mmol, quant) with spectroscopic data in accordance with the literature.[18] **m.p.** 121 °C {Lit.[18-19] 126 – 127°C}, {Lit.[20] 146 – 148 °C (EtOH)}, {Lit.[21] 134 – 136 °C (EtOH)}, {Lit.[22] 131 – 132 °C (PhH)}; **^{1}H NMR** (400 MHz, $CDCl_3$) δ_H 7.61 (1H, ddd, $^3J_{HH}$ = 7.5 Hz, $^4J_{HH}$ = 1.4 Hz, $^5J_{HH}$ = 0.6 Hz, ArC^4*H*), 7.48 (1H, ddd, $^3J_{HH}$ = 8.0 Hz, 7.6 Hz, $^4J_{HH}$ = 1.4 Hz, ArC^6*H*), 7.38 – 7.27 (5H, m, PhC^{2,3,4,5,6}*H*), 7.09 (1H, app td, $^3J_{HH}$ = 7.5 Hz, $^4J_{HH}$ = 0.8 Hz, ArC^5*H*), 6.78 (1H, ddd, $^3J_{HH}$ = 8.0 Hz, $^4J_{HH}$ = 0.8 Hz, $^5J_{HH}$ = 0.6 Hz, ArC^7*H*), 4.93 (2H, s, CH_2).

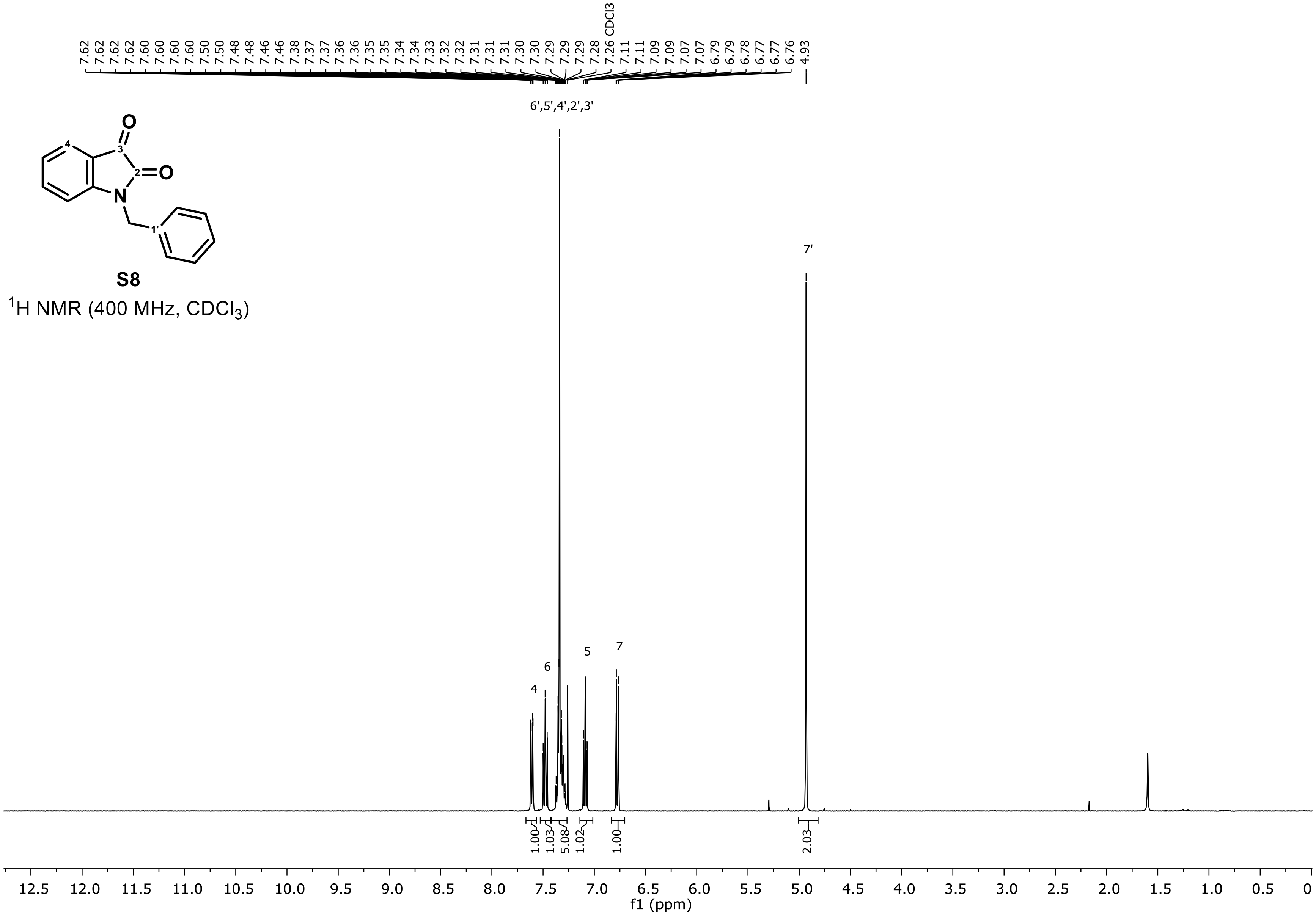

S8

^{1}H NMR (400 MHz, $CDCl_3$)

b) N-Benzyl-7-trifluoromethylisatin (S9)

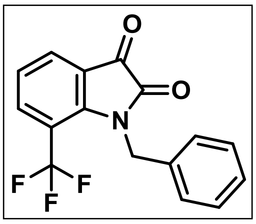

Following a literature procedure;[18] to a 250 ml round bottomed flask under N_2 was added DMF (19 ml, 0.25 M), 7-trifluoromethylisatin (1.00 g, 4.65 mmol), NaH (60% dispersion in mineral oil, 242 mg, 6.05 mmol) at 0 °C. The reaction was stirred at this temperature for 0.5 h. BnBr (660 µl, 5.55 mmol) was added, and the reaction was left to be stirred at room temperature for 2 h. Water (100 ml) was added and the resulting precipitated was filtered off. The precipitated was dissolved in CH_2Cl_2 and washed with brine. The combined organic layers were dried over $MgSO_4$ anhydrous, filtered, and concentrated *in vacuo*. Purification by recrystallisation from EtOAc (evaporation or precipitation with hexane) gave to give the title compound as bright yellow solid (1179.6 mg,3.86 mmol, 83%) with spectroscopic data in accordance with the literature.[23] **m.p.** 86-88 °C (EtOAc/Hexane); {Lit.[23c] 86-88 °C}, {Lit.[23a] 91-93 °C (PhMe/Hexane)}, {Lit.[24] 104 – 106 °C}; ***ν*** ν_{max} (thin film) 3034 (w, C-H), 2953 (w, C-H), 2924 (w, C-H), 2853 (w, C-H), 1746 (s, C=O), 1614 (s, C=O, amide), 1593 (m), 1497 (w), 1489 (w), 1450 (m), 1429 (s), 1341 (m), 1323 (s), 1277 (w), 1175 (m), 1150 (m), 1126 (s), 1101 (s), 1088 (m), 1074 (w), 1044 (w), 1028 (w), 959 (w), 833 (m), 779 (m); **^{1}H NMR** (400 MHz, $CDCl_3$) δ_H 7.88 (1H, dd, $^3J_{HH}$ = 7.5 Hz, $^4J_{HH}$ = 1.4 Hz, ArC$^{4'}$*H*), 7.86 (1H, dd, $^3J_{HH}$ = 8.2 Hz, $^4J_{HH}$ = 1.4 Hz, ArC^6*H*), 7.34 – 7.22 (4H, m, ArC^5*H*, PhC^{3,4,5}*H*), 7.19 – 7.13 (2H, m, PhC$^{2',6'}$*H*), 5.22 (2H, s, PhC^1-C*H*$_2$); **^{19}F{^{1}H} NMR** (377 MHz, $CDCl_3$) δ_F −55.58 (3F, s, CF_3).

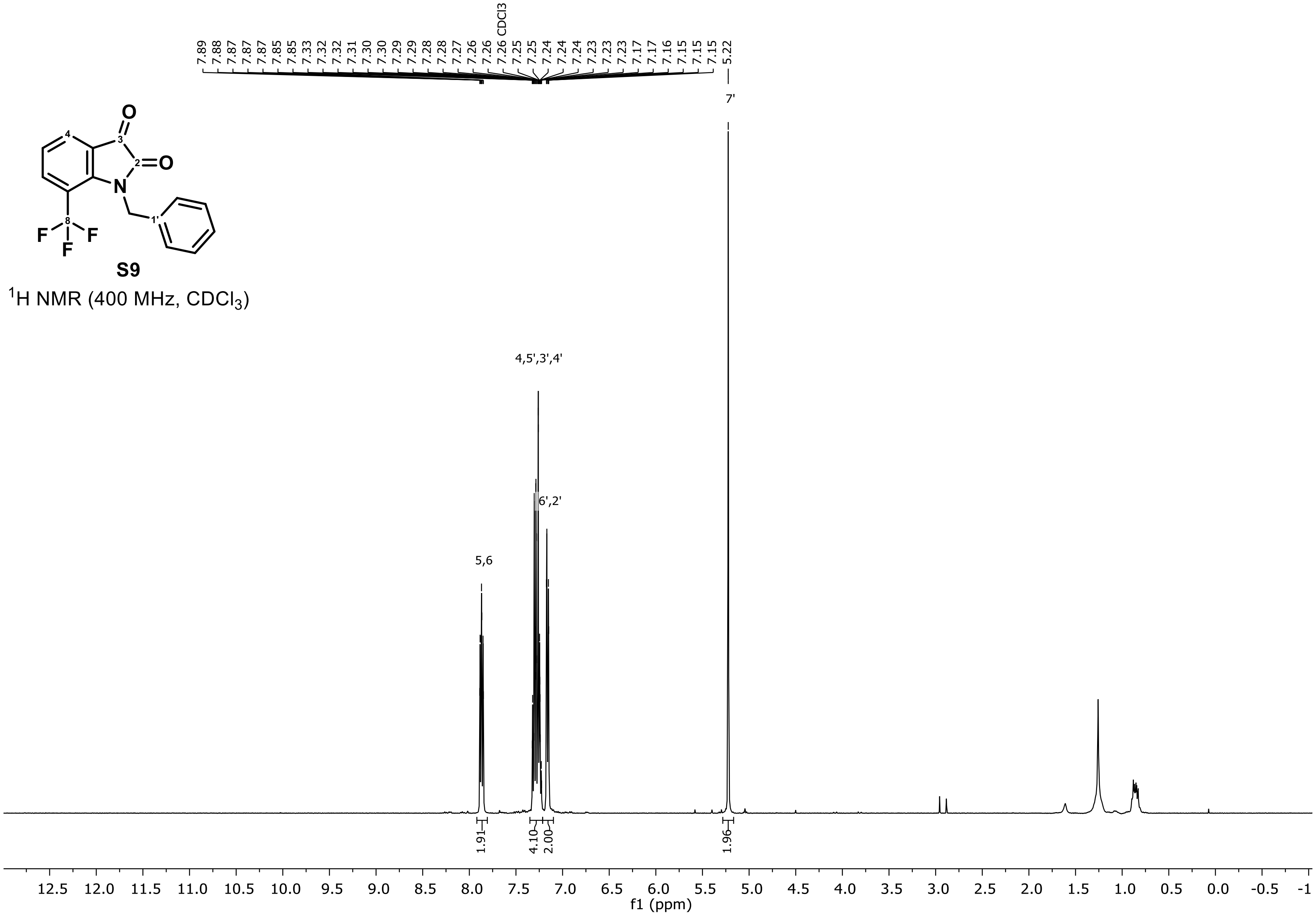
S9
^{1}H NMR (400 MHz, $CDCl_3$)
7.89 7.88 7.87 7.87 7.87 7.85 7.85 7.33 7.32 7.32 7.31 7.30 7.30 7.29 7.29 7.28 7.28 7.27 7.26 7.26 7.26 CDCl3 7.25 7.25 7.24 7.24 7.24 7.23 7.23 7.23 7.17 7.17 7.16 7.15 7.15 7.15 5.22
5,6
4,5',3',4'
6',2'
7'
1.91
4.10
2.00
1.96
f1 (ppm)

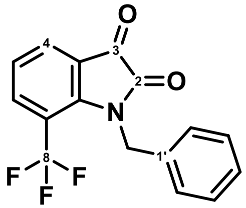

S9

^{19}F NMR (377 MHz, $CDCl_3$)

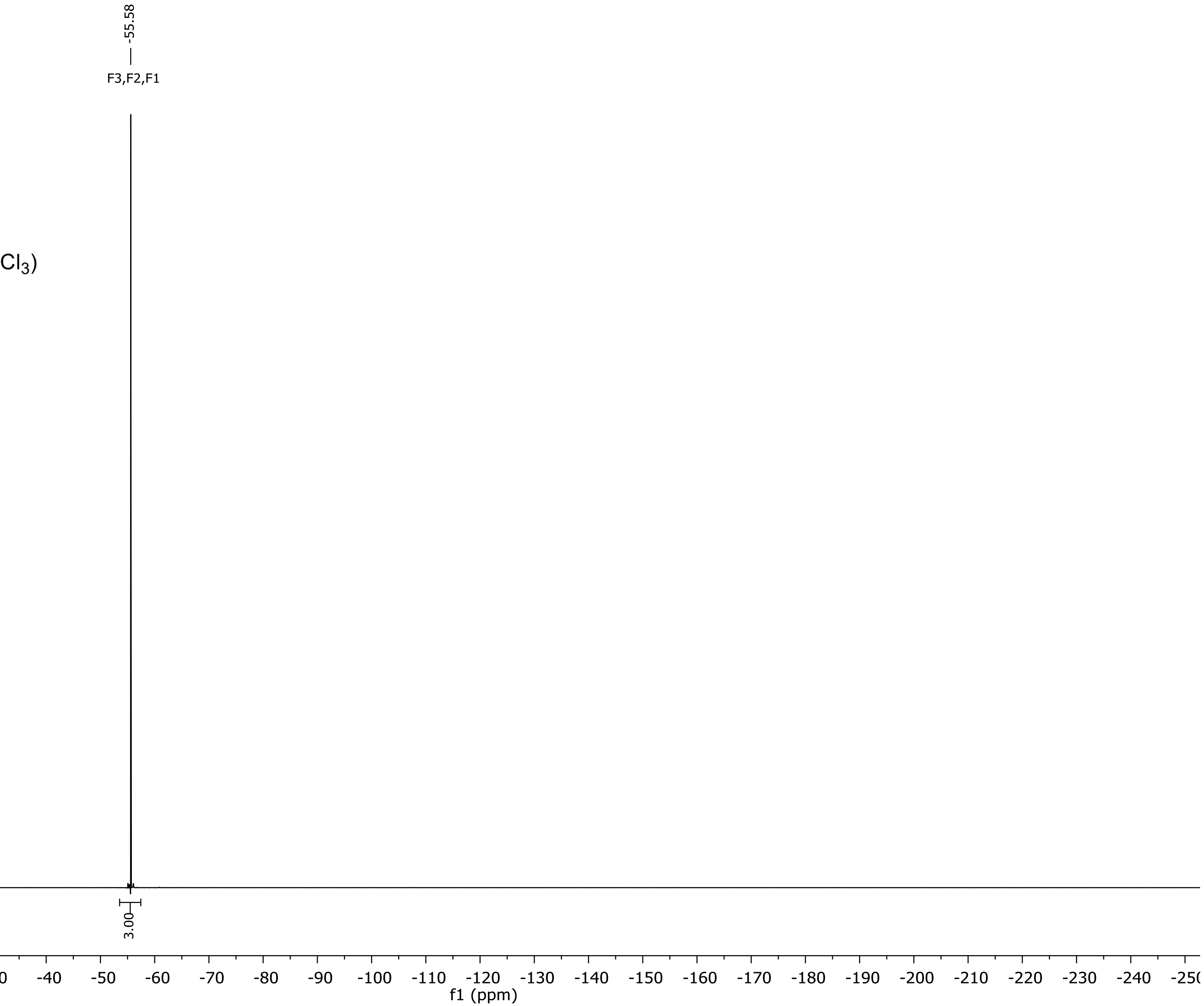

c) ***N*-Benzyl 7-Chloroisatin (S10)**

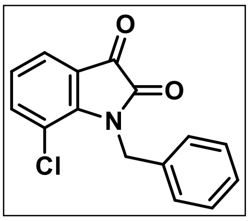

Following a literature procedure;[18] to a 50 ml round bottomed flask under N_2 was added DMF (9 ml, 0.25 M), 7-chloroisatin (410 mg, 2.25 mmol), NaH (60% dispersion in mineral oil, 117 mg, 2.90 mmol) at 0 °C. The reaction was stirred at this temperature for 0.5 h. BnBr (321 µL, 2.70 mmol) was added, and the reaction was left to be stirred at room temperature for 2 h. Water (25 ml) was added and the resulting precipitated was filtered off. The precipitated was dissolved in CH_2Cl_2 and washed with brine. The combined organic layers were dried over $MgSO_4$ anhydrous, filtered, and concentrated *in vacuo*. Purification by recrystallisation from EtOAc (evaporation or precipitation with hexane) gave to give the title compound as bright yellow solid (413 mg, 1.52 mmol, 68%) with spectroscopic data in accordance with the literature.[18, 25] **m.p.** 158 °C {Lit.[18] 152 – 155 °C}; **^{1}H NMR** (500 MHz, $CDCl_3$) δ_H 7.59 (1H, dd, $^3J_{HH}$ = 7.3 Hz, $^4J_{HH}$ = 1.3 Hz, ArC^4*H*), 7.48 (1H, dd, $^3J_{HH}$ = 8.2 Hz, $^4J_{HH}$ = 1.3 Hz, ArC^6*H*), 7.36 – 7.26 (5H, m, PhC^{2,3,4,5,6}*H*), 7.07 (1H, dd, $^3J_{HH}$ = 8.2 Hz, 7.3 Hz, ArC^5*H*), 5.38 (2H, s, C*H*$_2$).

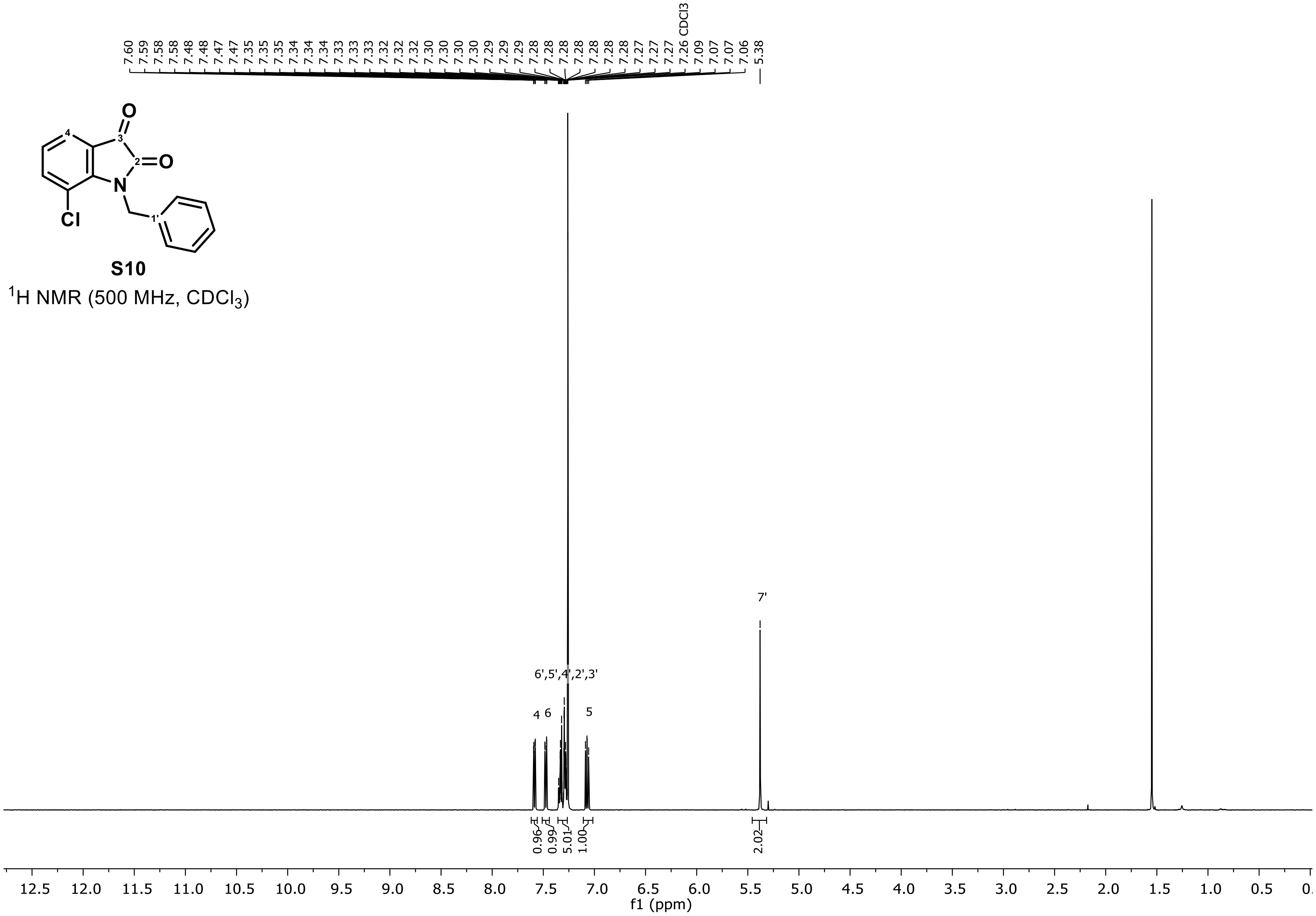
O
O
N
Cl
4
3
2
1'
S10
¹H NMR (500 MHz, CDCl3)
7.60
7.59
7.58
7.58
7.48
7.48
7.47
7.47
7.35
7.35
7.35
7.34
7.34
7.34
7.33
7.33
7.33
7.32
7.32
7.32
7.30
7.30
7.30
7.30
7.29
7.29
7.29
7.28
7.28
7.28
7.28
7.28
7.28
7.28
7.27
7.27
7.27
7.26 CDCl3
7.09
7.07
7.07
7.06
5.38
7'
6',5',4',2',3'
4
6
5
0.96
0.99
5.01
1.00
2.02
12.5
12.0
11.5
11.0
10.5
10.0
9.5
9.0
8.5
8.0
7.5
7.0
6.5
6.0
5.5
5.0
4.5
4.0
3.5
3.0
2.5
2.0
1.5
1.0
0.5
f1 (ppm)

d) *N*-Benzyl 6-Chloroisatin (S11)

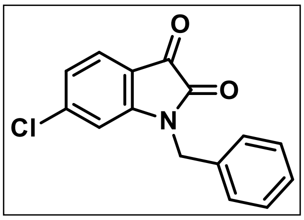

Following a literature procedure;[18] to a 250 ml round bottomed flask under N_2 was added DMF (20 ml, 0.25 M), 6-chloroisatin (908 mg, 5.00 mmol), NaH (60% dispersion in mineral oil, 260 mg, 6.50 mmol) at 0 °C. The reaction was stirred at this temperature for 0.5 h. BnBr (713 µL, 6.00 mmol) was added, and the reaction was left to be stirred at room temperature for 2 h. Water (100 ml) was added and the resulting precipitated was filtered off. The precipitated was dissolved in CH_2Cl_2 and washed with brine. The combined organic layers were dried over $MgSO_4$ anhydrous, filtered, and concentrated *in vacuo*. Purification by recrystallisation from EtOAc (evaporation or precipitation with hexane) gave to give the title compound as bright yellow solid (1060 mg, 3.90 mmol, 78%) with spectroscopic data in accordance with the literature.[26] **m.p.** 169 °C {Lit.[27] 175 – 176 °C (AcOH)}, {Lit.[26] 173 – 174°C}, {Lit.[28] 166 – 167 °C}; ^{1}**H NMR** (500 MHz, $CDCl_3$) δ_H 7.55 (1H, d, $^3J_{HH}$ = 8.0 Hz, Ar*H*), 7.30 – 7.45 (5H, m, PhC^{2,3,4,5,6}*H*), 7.08 (1H, dd, $^3J_{HH}$ = 8.0 Hz, $^4J_{HH}$ = 1.7 Hz, ArC^5*H*), 6.78 (1H, d, $^4J_{HH}$ = 1.6 Hz, ArC^7*H*), 4.91 (2H, s, C*H*$_2$).

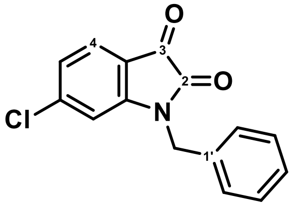

S11

^{1}H NMR (500 MHz, $CDCl_3$)

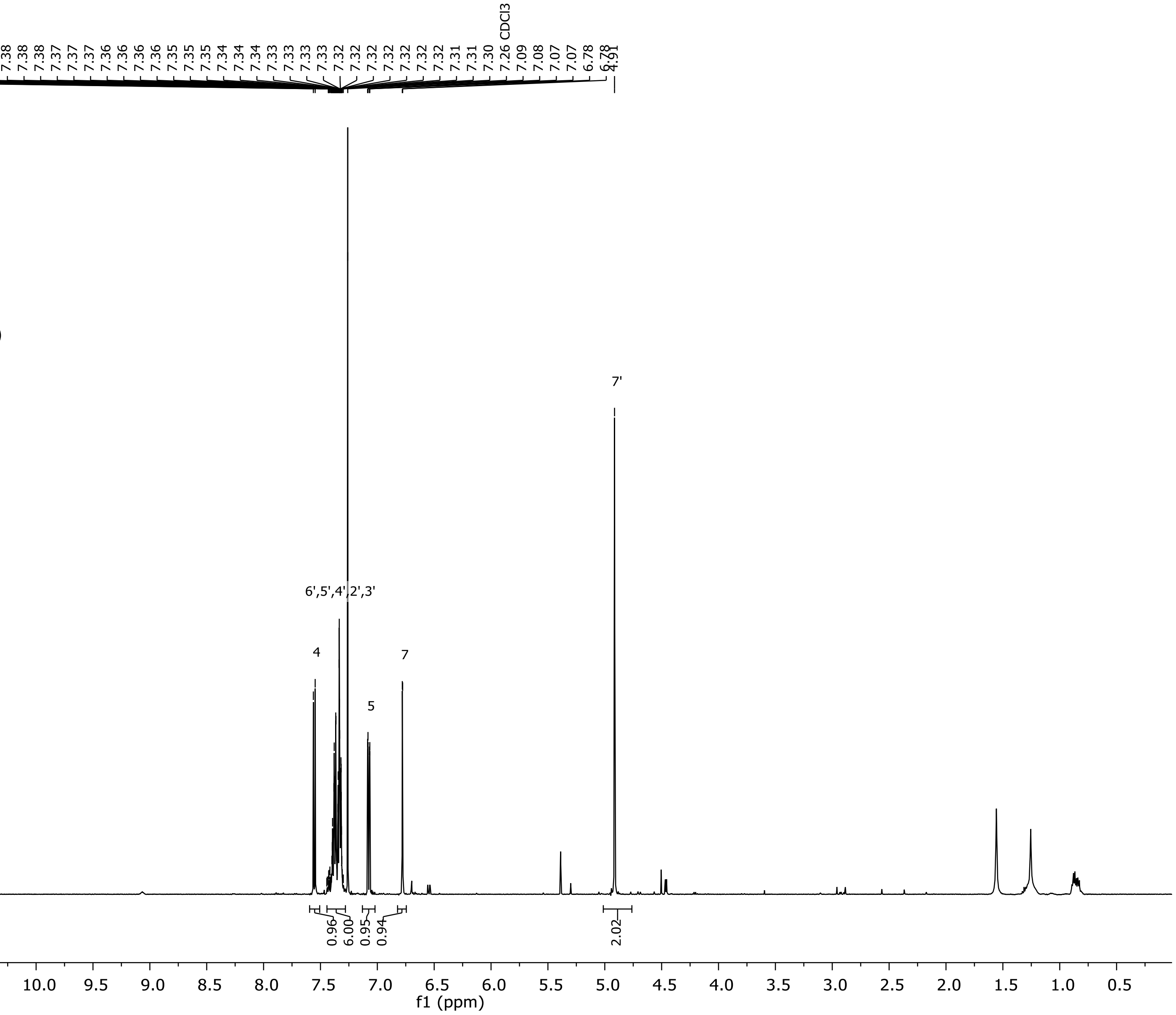

e) ***N*-Benzyl-5-chloroisatin (S12)**

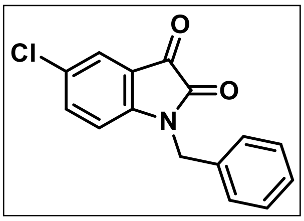

Following a literature procedure;[18] to a 250 ml round bottomed flask under N_2 was added DMF (45 ml, 0.25 M), 5-chloroisatin (2035 mg, 11.21 mmol), NaH (60% dispersion in mineral oil, 583 mg, 15.58 mmol) at 0 °C, the reaction was stirred at this temperature for 2 h. BnBr (1.6 ml, 13.45 mmol) was added, and the reaction was left to be stirred at room temperature for 2 h. Water (100 ml) was added and the resulting precipitated was filtered off. The precipitated was dissolved in CH_2Cl_2 and washed with brine. The combined organic layers were dried over $MgSO_4$ anhydrous, filtered, and concentrated *in vacuo* to give the title compound as red solid (2556.4 mg, 9.41 mmol, 84%). Further purification by recrystallisation from EtOAc (evaporation or precipitation with hexane) gave 1714.1 mg (6.31 mmol, 56%) with spectroscopic data in accordance with the literature.[18, 27, 29] m.p. 140 °C (EtOAc/Hexane), {Lit. 148 – 152 °C (no solvent given),[30] 142 – 144 °C (no solvent given),[29] 134 – 136 °C (no solvent given)[18]}; IR ν_{max} (film) 3065 (w, C-H), 2932 (w, C-H), 1738 (s, C=O), 1607 (s, C=O, amide), 1474 (s), 1445 (m), 1350 (m), 1331 (m), 1258 (w), 1188 (m), 1175 (m), 1126 (m), 1082 (w), 1069 (w), 1030 (w), 957 (w), 901 (w), 822 (m); **^{1}H NMR** (400 MHz, $CDCl_3$) δ_H 7.60 (1H, d, $^4J_{HH}$ = 2.2 Hz, ArC^4*H*), 7.46 (1H, dd, $^3J_{HH}$ = 8.4 Hz, $^4J_{HH}$ = 2.2 Hz, ArC^6*H*), 7.40 – 7.36 (2H, m, PhC^{3,5}*H*), 7.36 – 7.31 (3H, m, PhC^{2,4,6}*H*), 6.75 (1H, d, $^3J_{HH}$ = 8.4 Hz, ArC^7*H*), 4.95 (2H, s, PhC^1-C*H*$_2$).

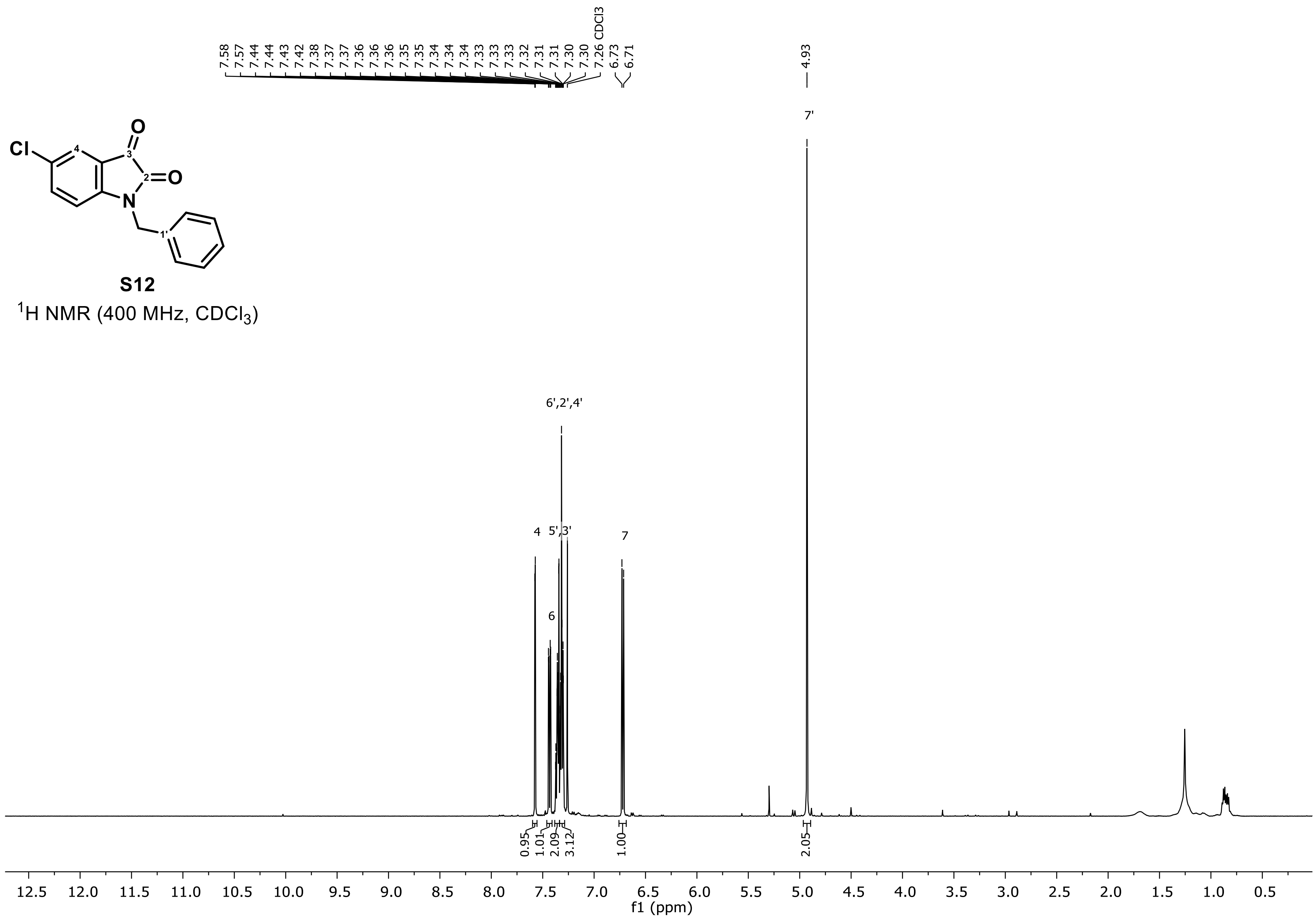

^{1}H NMR (400 MHz, $CDCl_3$)

f) *N*-Benzyl-4-chloroisatin (S13)

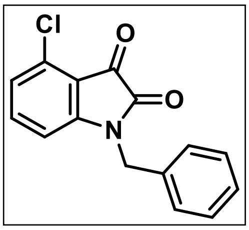

Following a literature procedure;[18] to a 250 ml round bottomed flask under N_2 was added DMF (45 ml, 0.25 M), 4-chloroisatin (2035 mg, 11.21 mmol), NaH (60% dispersion in mineral oil, 583 mg, 15.58 mmol) at 0 °C, the reaction was stirred at this temperature for 2 h. BnBr (1.6 ml, 13.45 mmol) was added, and the reaction was left to be stirred at room temperature for 2 h. Water (100 ml) was added and the resulting precipitated was filtered off. The precipitated was dissolved in CH_2Cl_2 and washed with brine. The combined organic layers were dried over $MgSO_4$ anhydrous, filtered, and concentrated *in vacuo* to give the title compound as red solid (2302.9 mg, 8.48 mmol, 76%). Further purification by recrystallisation from EtOAc (evaporation or precipitation with hexane) to give the title compound (1771.1 mg, 6.52 mmol, 58%) with spectroscopic data in accordance with literature.[18, 27] **m.p.** 162 °C (Hexane/EtOAc); $\boldsymbol{\nu}_{\mathbf{max}}$ (thin film) 3098 (w), 3032 (w), 1730 (s, C=O), 1601 (s, C=O), 1587 (s), 1495 (w), 1472 (w), 1452 (s), 1369 (w), 1352 (m), 1327 (m), 1300 (w), 1231 (s), 1167 (m), 1150 (m), 1076 (w), 1049 (m), 1028 (w), 1015 (w), 890 (w), 864 (m), 814 (w), 797 (s); **^{1}H NMR** (400 MHz, $CDCl_3$) δ_H 7.38 (1H, app t, $^3J_{HH}$ = 8.3 Hz, 7.9 Hz, ArC^6*H*), 7.38 – 7.33 (2H, m, PhC^{3,5}*H*), 7.33 – 7.29 (3H, m, PhC^{2,4,6}*H*), 7.03 (1H, d, $^3J_{HH}$ = 8.3 Hz, ArC^5*H*), 6.68 (1H, d, $^3J_{HH}$ = 7.9 Hz, ArC^7*H*), 4.94 (1H, s, PhC^1-CH_2).

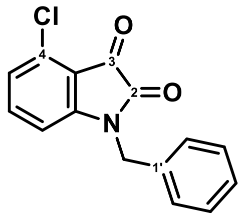

S13

^1H NMR (400 MHz, $CDCl_3$)

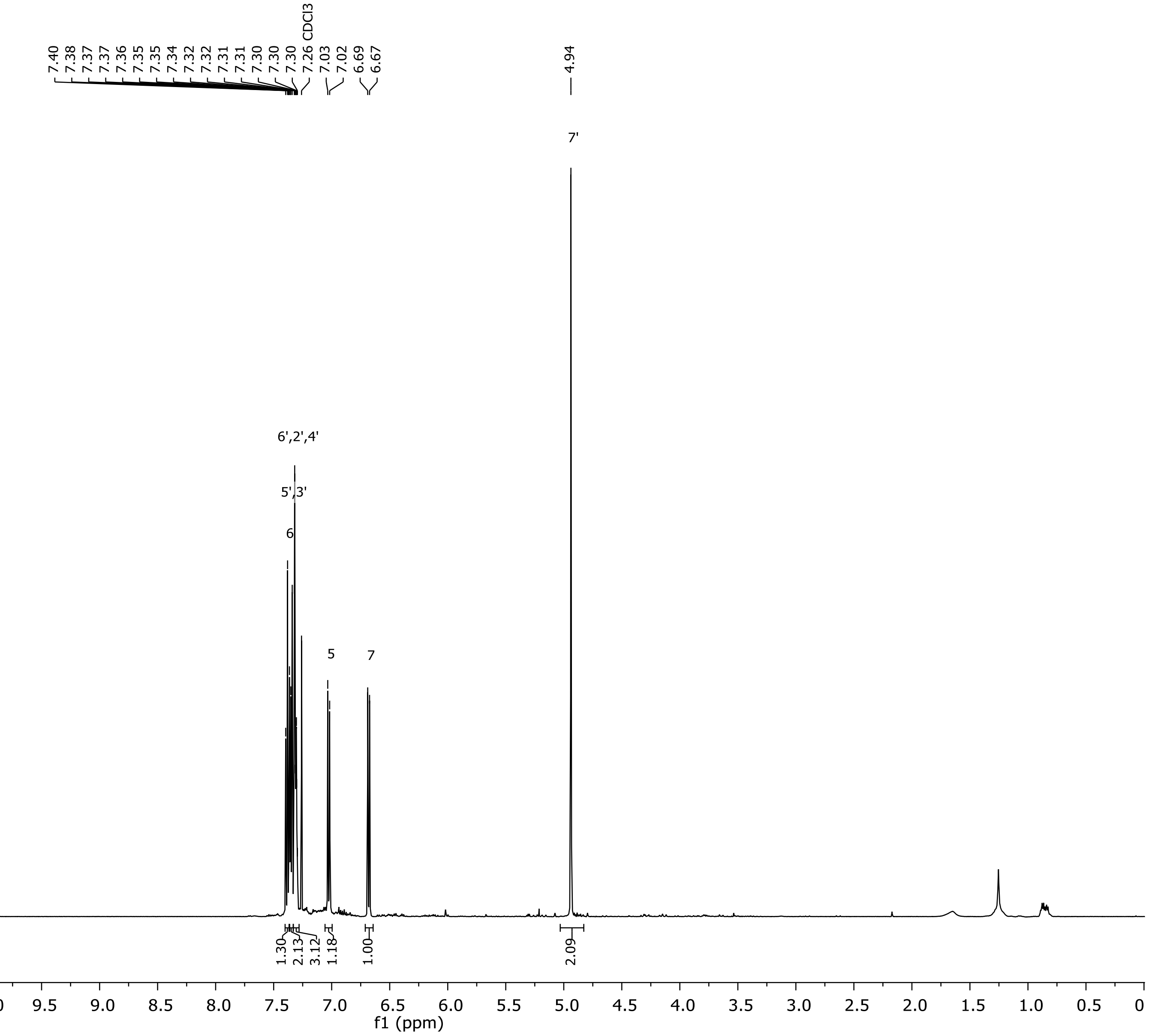

g) *N*-Benzyl-5-bromoisatin (S14)

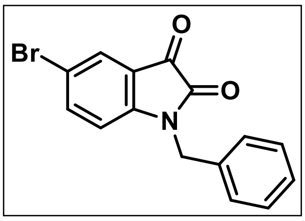

Following a literature procedure;[18] to a 250 ml round bottomed flask under N_2 was added DMF (30 ml, 0.25 M), 5-bromoisatin (1.69 g, 7.48 mmol), NaH (60% dispersion in mineral oil, 389 mg, 9.72 mmol) at 0 °C, the reaction was stirred at this temperature for 2 h. BnBr (1.07 ml, 8.98 mmol) was added, and the reaction was left to be stirred at room temperature for 2 h. Water (100 ml) was added and the resulting precipitated was filtered off. The precipitated was dissolved in CH_2Cl_2 and washed with brine. The combined organic layers were dried over $MgSO_4$ anhydrous, filtered, and concentrated *in vacuo.* Purification by recrystallisation from EtOAc (evaporation or precipitation with hexane) gave the title compound as red solid (1126.5 mg, 3.56 mmol, 48%). **m.p.** 148 – 150 °C (Hexane/EtOAc), {Lit.[20-21] 149 – 151 °C (EtOH)}, {Lit.[31] 147 – 149 °C}, {Lit.[18] 145 – 147 °C}, {Lit.[29] 106 – 108 °C}; $\boldsymbol{\nu}_{\mathbf{max}}$ (thin film) 3065 (w, C-H), 3034 (w, C-H), 2934 (w, C-H), 1738 (s, C=O ketone), 1605 (s, C=O amide), 1497 (m, C=C), 1497 (w), 1470 (m), 1454 (w), 1437 (m), 1350 (w), 1329 (m), 1260 (w), 1177 (m), 1126 (m), 1082 (w), 1059 (w), 1030 (w), 908 (w), 820 (m); **^{1}H NMR** (400 MHz, $CDCl_3$) δ_H 7.74 – 7.69 (1H, m, ArC^4*H*), 7.58 (1H, dd, $^3J_{HH}$ = 8.4 Hz, $^4J_{HH}$ = 2.1 Hz, ArC^6*H*), 7.40 – 7.28 (5H, m, PhC^{2,3,4,5,6}*H*), 6.67 (1H, d, $^3J_{HH}$ = 8.4 Hz, ArC^7*H*), 4.93 (2H, s, PhC^1-C*H*$_2$).

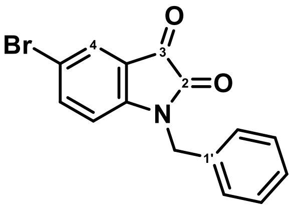

S14

^{1}H NMR (400 MHz, $CDCl_3$)

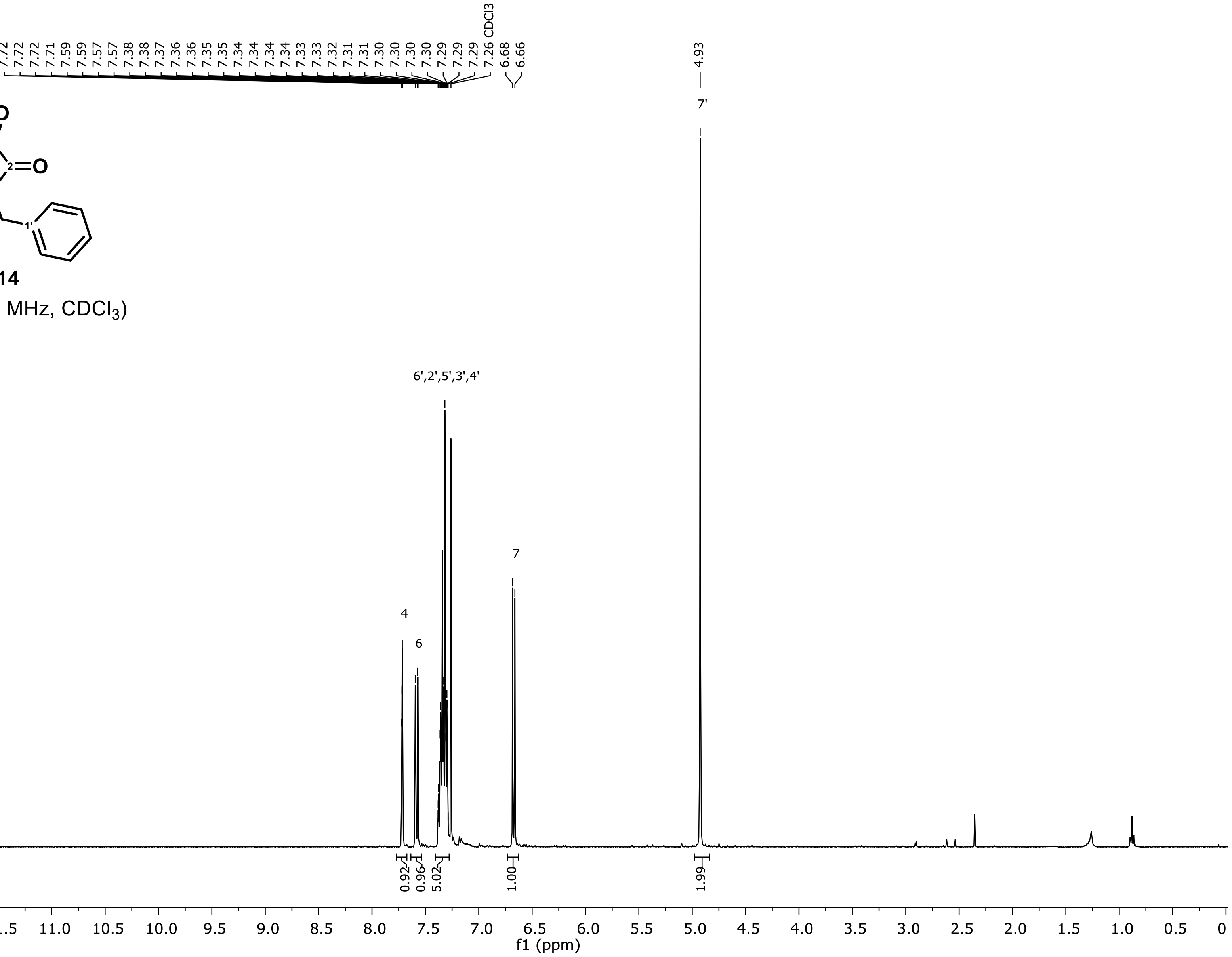

h) N-Benzyl-5-trifluoromethoxyisatin (S15)

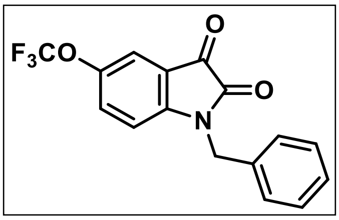

Following a literature procedure;[18] to a 100 ml round bottomed flask under Argon was added DMF (17.5 ml, 0.25 M), 5-trifluoromethoxyisatin (1.00 g, 4.33 mmol), NaH (60% dispersion in mineral oil, 225 mg, 5.63 mmol) at 0 °C. The reaction was stirred at this temperature for 2 h. BnBr (620 µl, 5.21 mmol) was added, and the reaction was left to be stirred at room temperature for 2 h. Water (100 ml) was added and the resulting precipitated was filtered off. The precipitated was dissolved in CH_2Cl_2 and washed with brine. The combined organic layers were dried over $MgSO_4$ anhydrous, filtered, and concentrated *in vacuo*. Purification by recrystallisation from EtOAc (evaporation or precipitation with hexane) gave to give the title compound as bright red solid (1087 mg, 3.38 mmol, 78%) with spectroscopic data in accordance with literature.[23a, 32] **m.p.** (EtOAc/Hexane) 102-104 °C; {Lit. 100-102 °C, no solvent given}[32], {106-107 °C, PhMe/Hexane}[23a] ***ν*****max** (thin film) 3063 (w, C-H), 3036 (w, C-H), 2926 (w, C-H), 2855 (w, C-H), 1740 (s, isatin ketone), 1622 (s, isatin amide), 1605 (w), 1485 (s, C=C), 1466 (w), 1456 (w), 1350 (w), 1333 (m), 1254 (s, C-O, C-F), 1213 (s, C-O, C-F), 1173 (s, C-O, C-F), 1115 (w), 1082 (w), 1030 (w), 978 (w), 903 (w), 831 (w), 793 (w); **^{1}H NMR** (400 MHz, $CDCl_3$) δ_H 7.49 (1H, dd, $^4J_{HH}$ = 2.5 Hz, $^5J_{HH}$ = 1.1 Hz, ArC$_4$*H*), 7.30 – 7.40 (m, 6H, ArC^6*H*, PhC$^{2',3',4',5',6'}$*H*), 6.80 (1H, d, $^3J_{HH}$ = 8.6 Hz, ArC^7*H*), 4.95 (s, 2H, PhC*H*$_2$); **^{19}F NMR** (377 MHz, $CDCl_3$) δ_F −58.55 (OC*F*$_3$).

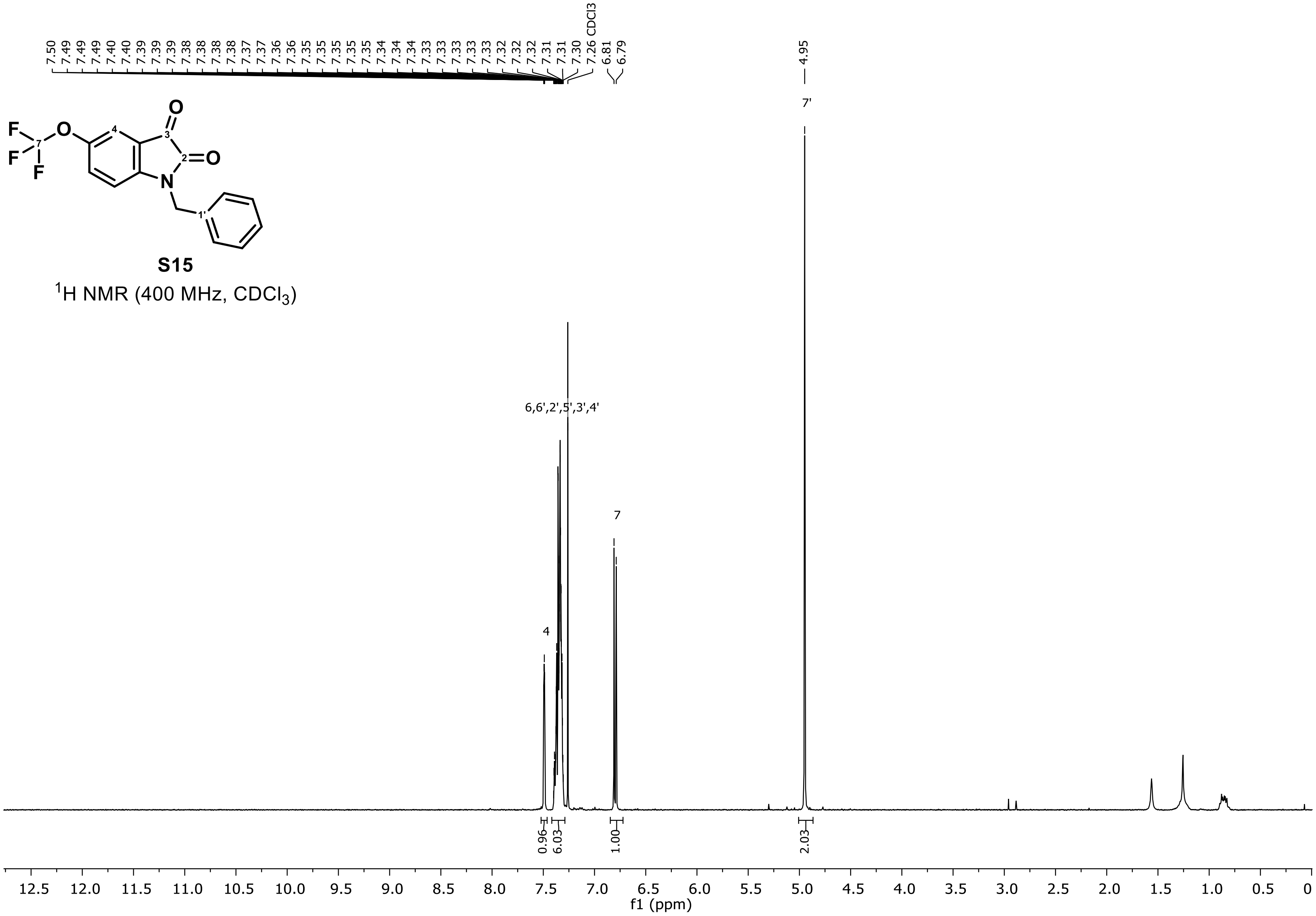
7.50 7.49 7.49 7.49 7.40 7.40 7.39 7.39 7.39 7.38 7.38 7.38 7.38 7.37 7.37 7.36 7.36 7.35 7.35 7.35 7.35 7.35 7.34 7.34 7.34 7.33 7.33 7.33 7.33 7.33 7.32 7.32 7.32 7.31 7.31 7.30 7.26 CDCl3 6.81 6.79
4.95
7'
S15
1H NMR (400 MHz, CDCl3)
6,6',2',5',3',4'
7
4
0.96
6.03
1.00
2.03
12.5 12.0 11.5 11.0 10.5 10.0 9.5 9.0 8.5 8.0 7.5 7.0 6.5 6.0 5.5 5.0 4.5 4.0 3.5 3.0 2.5 2.0 1.5 1.0 0.5 0
f1 (ppm)

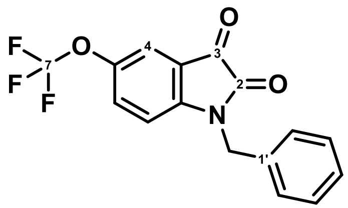

S15

^{19}F NMR (377 MHz, $CDCl_3$)

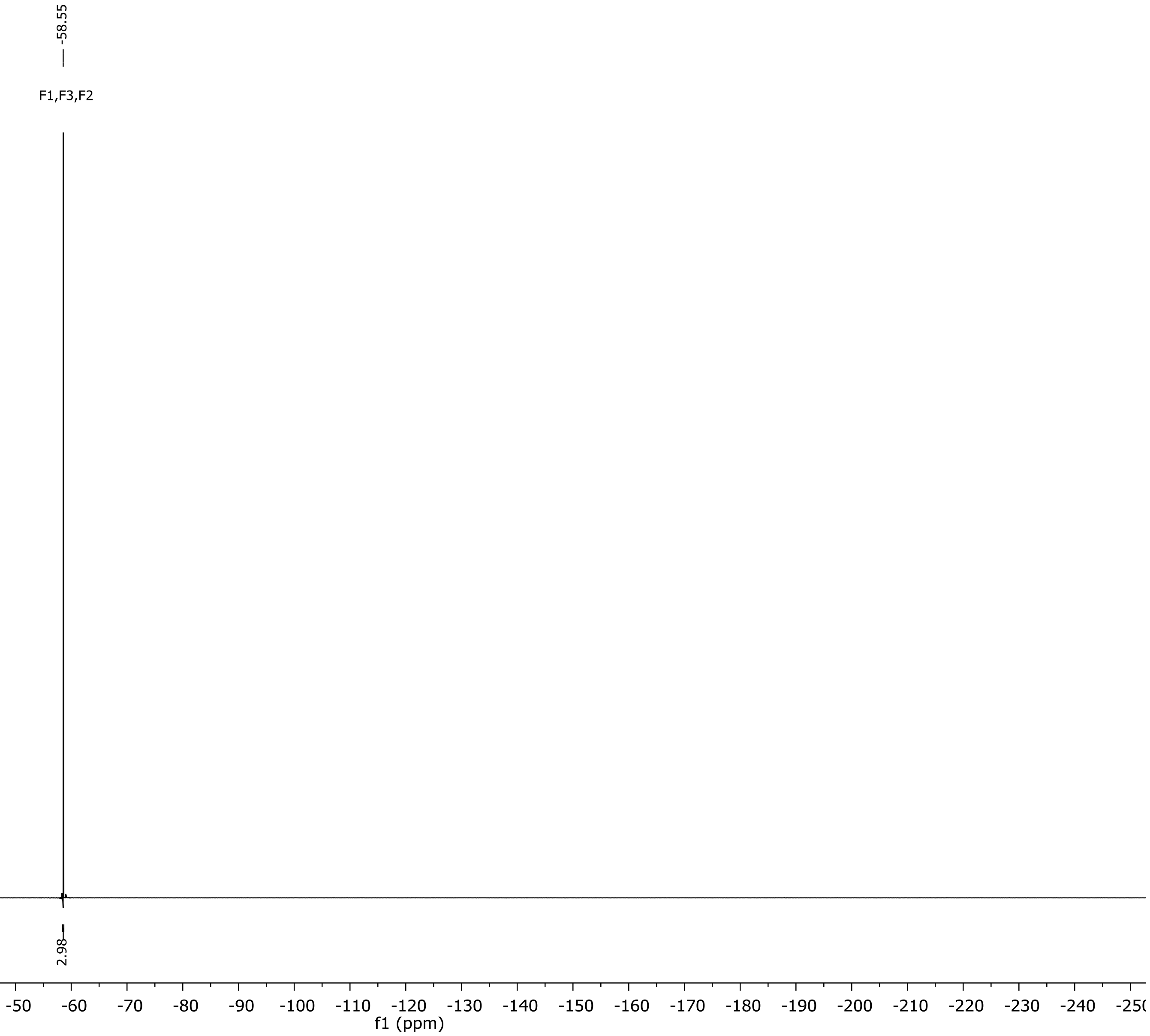

i) *N*-Benzyl-5-methoxyisatin (S16)

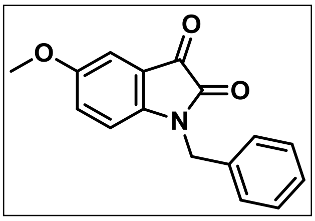

Following a literature procedure;[18] to a 250 ml round bottomed flask under N_2 was added DMF (23 ml, 0.25 M), 5-methoxyisatin (1.00 g, 5.65 mmol), NaH (60% dispersion in mineral oil, 293.6 mg, 7.34 mmol) at 0 °C, the reaction was stirred at this temperature for 2 h. BnBr (800 µl, 6.72 mmol) was added, and the reaction was left to be stirred at room temperature for 2 h. Water (100 ml) was added and the resulting precipitated was filtered off. The precipitated was dissolved in CH_2Cl_2 and washed with brine. The combined organic layers were dried over $MgSO_4$ anhydrous, filtered, and concentrated *in vacuo*. Purification by recrystallisation from EtOAc (evaporation or precipitation with hexane) gave to give the title compound as dark purple solid (887.9 mg, 3.32 mmol, 59%) with spectroscopic data in accordance with literature.[18, 23a, 33] m.p. 116 °C (hexane/EtOAc) {Lit. 127 -128 (no solvent given),[33a] 120 – 121 (toluene),[18] 117 – 118 (hexane),[33b] 115 – 116 (no solvent given)[18]}; ***ν*~max~** (thin film) 3067 (w), 3030 (w), 3011 (w), 2963 (w), 2835 (w), 1721 (s, C=O), 1620 (m), 1601 (m), 1493 (s), 1474 (m), 1452 (m), 1435 (m), 1348 (m), 1335 (m), 1312 (m), 1271 (m), 1144 (m), 1078 (w), 1045 (m), 1018 (m), 964 (w), 866 (w), 835 (s), 806 (w), 772 (s); **^{1}H NMR** (400 MHz, $CDCl_3$) δ_H 7.38 – 7.27 (5H, m, PhC^{2,3,4,5,6}*H*), 7.15 (1H, d, $^4J_{HH}$ = 2.7 Hz, ArC^4*H*), 7.02 (1H, dd, $^3J_{HH}$ = 8.6 Hz, $^4J_{HH}$ = 2.7 Hz, ArC^6*H*), 6.67 (1H, d, $^3J_{HH}$ = 8.6 Hz, ArC^7*H*), 4.90 (2H, s, PhC^1-CH_2), 3.77 (3H, s, OCH_3).

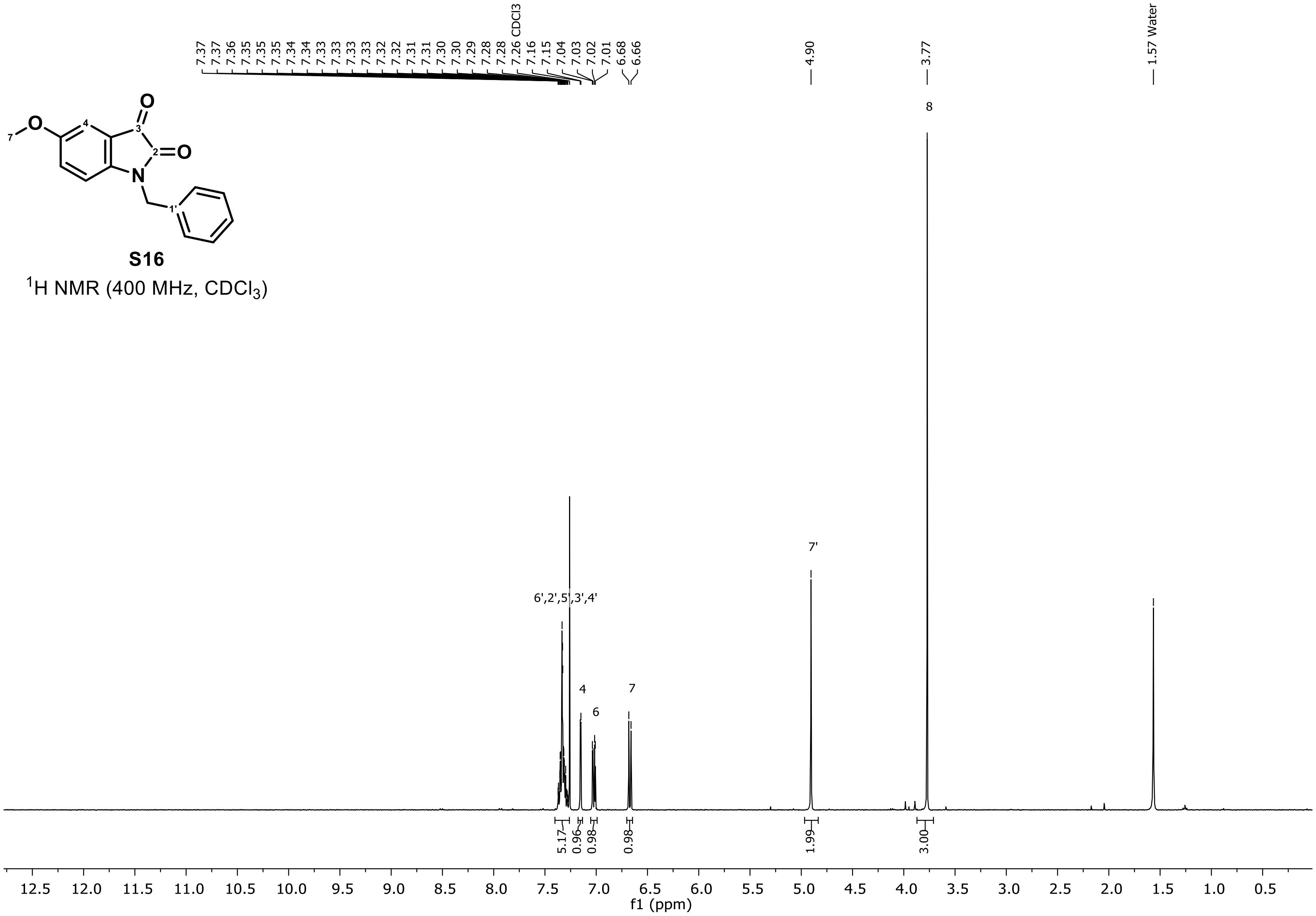

S16

^1H NMR (400 MHz, $CDCl_3$)

*j) N-(para-tert-*Butylbenzyl)isatin (S17)

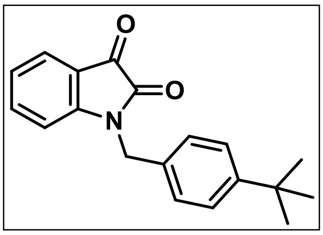

Following a literature procedure;[18] to a 250 ml round bottomed flask under Argon was added anhydrous DMF (40 ml, 0.25 M), isatin (1471.3 mg, 10.00 mmol), NaH (60% dispersion in mineral oil, 520 mg, 13.00 mmol) at 0 °C, the reaction was stirred at this temperature for 2 h. para-tert-Butylbenzyl bromide (2.2 ml, 12.00 mmol) was added, and the reaction was left to be stirred at room temperature for 2 h. Water (100 ml) was added and the resulting precipitated was filtered off. The precipitated was dissolved in CH_2Cl_2 and washed with brine. The combined organic layers were dried over $MgSO_4$ anhydrous, filtered, and concentrated *in vacuo*. The residue was redissolved in minimum volume of CH_2Cl_2 and hexane was added. The sample was left to partially evaporate overnight to give the title compound as red crystalline solid (1915.9 mg, 6.53 mmol, 63%) with spectroscopic data in accordance with literature.[23b, 23c] m.p. 188 °C (Hexane/EtOAc), {Lit.[23c] 189 – 191 °C}; $\boldsymbol{\nu}_{\mathbf{max}}$ (thin film) 2963 (m), 2907 (w), 2868 (w), 1732 (s), 1611 (s), 1516 (w), 1470 (s), 1439 (w), 1416 (w), 1372 (m), 1348 (s), 1315 (w), 1292 (w), 1269 (w), 1194 (w), 1177 (m), 1152 (w), 1123 (w), 1109 (w), 1096 (w), 1030 (w), 1018 (w), 1005 (w), 934 (w), 908 (w), 858 (m), 818 (w), 754 (s); **^{1}H NMR** (400 MHz, $CDCl_3$) δ_H 7.50 (1H, ddd, $^3J_{HH}$ = 8.0 Hz, 7.6 Hz, $^4J_{HH}$ = 1.4 Hz, ArC^6*H*), 7.40 – 7.33 (2H, m, ^{t}BuArC$^{2,\,6}$*H*), 7.30 – 7.24 (2H, m, ^{t}BuArC^{3,5}*H*), 7.09 (1H, ddd, $^3J_{HH}$ = 7.6 Hz, 7.4 Hz, $^4J_{HH}$ = 0.8 Hz, ArC^5*H*), 6.83 (1H, app d, $^3J_{HH}$ = 8.0 Hz, ArC^7*H*), 4.90 (2H, s, ^{t}BuArC^{1}C*H*$_2$), 1.29 (9H, s, C(C*H*$_3$)$_3$).

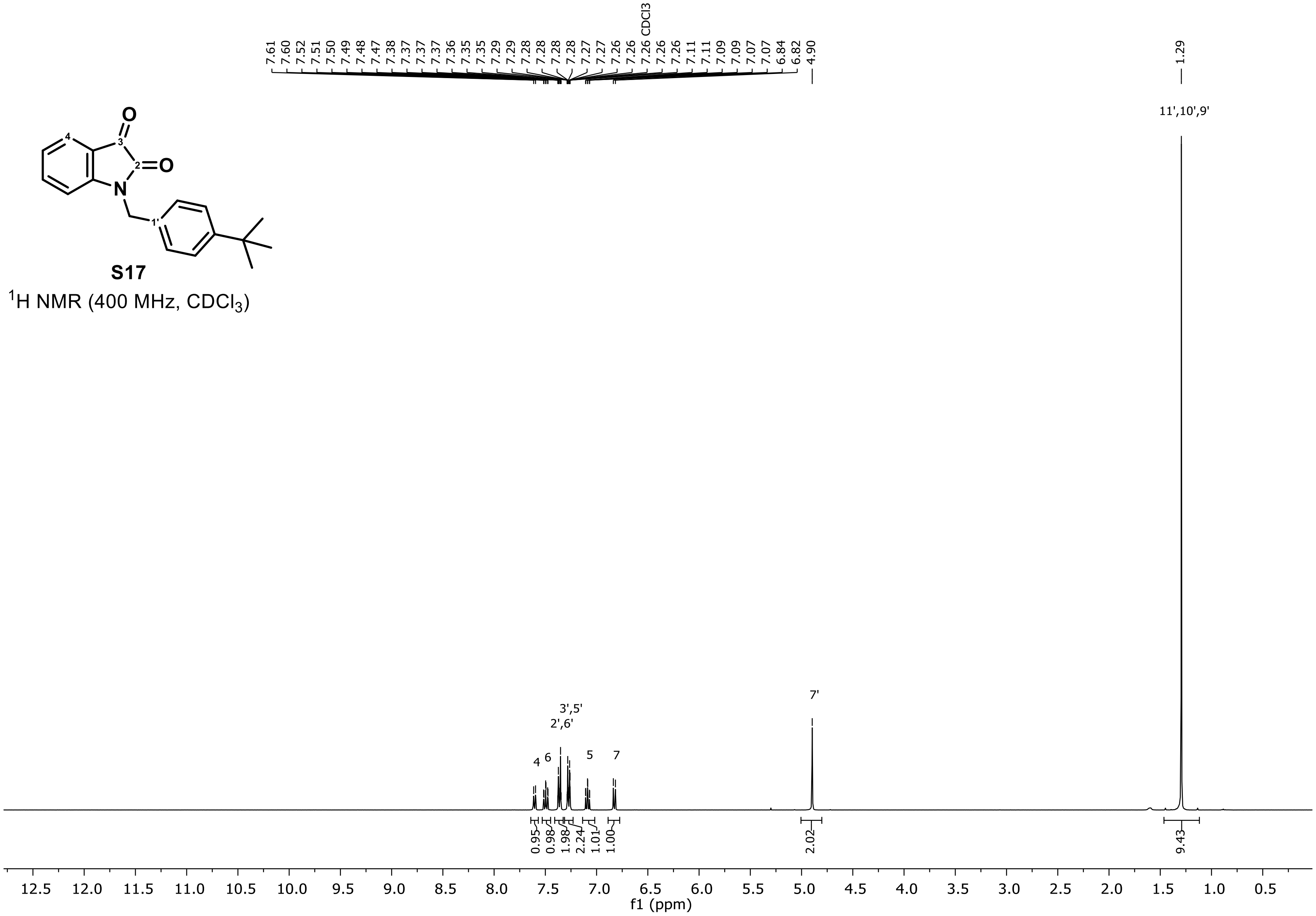
S17
^{1}H NMR (400 MHz, $CDCl_3$)
7.61
7.60
7.52
7.51
7.50
7.49
7.48
7.47
7.38
7.37
7.37
7.37
7.36
7.35
7.35
7.29
7.29
7.28
7.28
7.28
7.28
7.27
7.26
7.26 CDCl3
7.26
7.26
7.11
7.11
7.09
7.09
7.07
7.07
6.84
6.82
4.90
1.29
11',10',9'
7'
3',5'
2',6'
4
6
5
7
0.95
0.98
1.98
2.24
1.01
1.00
2.02
9.43
f1 (ppm)

k) *N*-Methylisatin (S18)

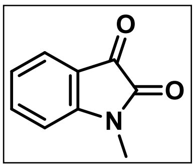

Following a literature procedure;[18] to a 250 ml round bottomed flask under N_2 was added DMF (80 ml, 0.25 M), isatin (2940 mg, 20.0 mmol), NaH (60% dispersion in mineral oil, 1040 mg, 26.0 mmol) at 0 °C, the reaction was stirred at this temperature for 2 h. MeI (1.49 ml, 24.0 mmol) was added, and the reaction was left to be stirred at room temperature for 2 h. Water (250 ml) was added and the resulting precipitated was filtered off. The precipitated was dissolved in CH_2Cl_2 and washed with brine. The combined organic layers were dried over $MgSO_4$ anhydrous, filtered, and concentrated *in vacuo*. Purification by recrystallisation from EtOAc (evaporation or precipitation with hexane) gave to give the title compound as dark purple solid (1090 mg, 6.76 mmol, 34%) with spectroscopic data in accordance with literature.[18] **m.p.** 102–106 °C {Lit.[18] 124 – 125 °C (EtOAc/Hexane)}, {Lit.[21] 126 – 127 (EtOH)}, {Lit.[20] 131 – 132 °C (EtOH)}; **^{1}H NMR** (500 MHz, $CDCl_3$) δ_H 7.61 (2H, 2 superimposed multiplets: dd, $^3J_{HH}$ = 7.5 Hz, $^4J_{HH}$ = 1.5 Hz, ArC^4*H* and ddd, $^3J_{HH}$ = 8.2 Hz, 7.5 Hz, $^4J_{HH}$ = 1.5 Hz, ArC^6*H*), 7.13 (1H, app td, $^3J_{HH}$ = 7.5 Hz, $^4J_{HH}$ = 0.8 Hz, ArC^5*H*), 6.89 (1H, dd, $^3J_{HH}$ = 8.2 Hz, $^4J_{HH}$ = 0.8 Hz, ArC^7*H*), 3.26 (3H, s, CH_3).

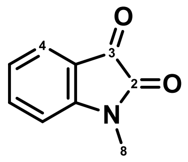

S18

^{1}H NMR (400 MHz, $CDCl_3$)

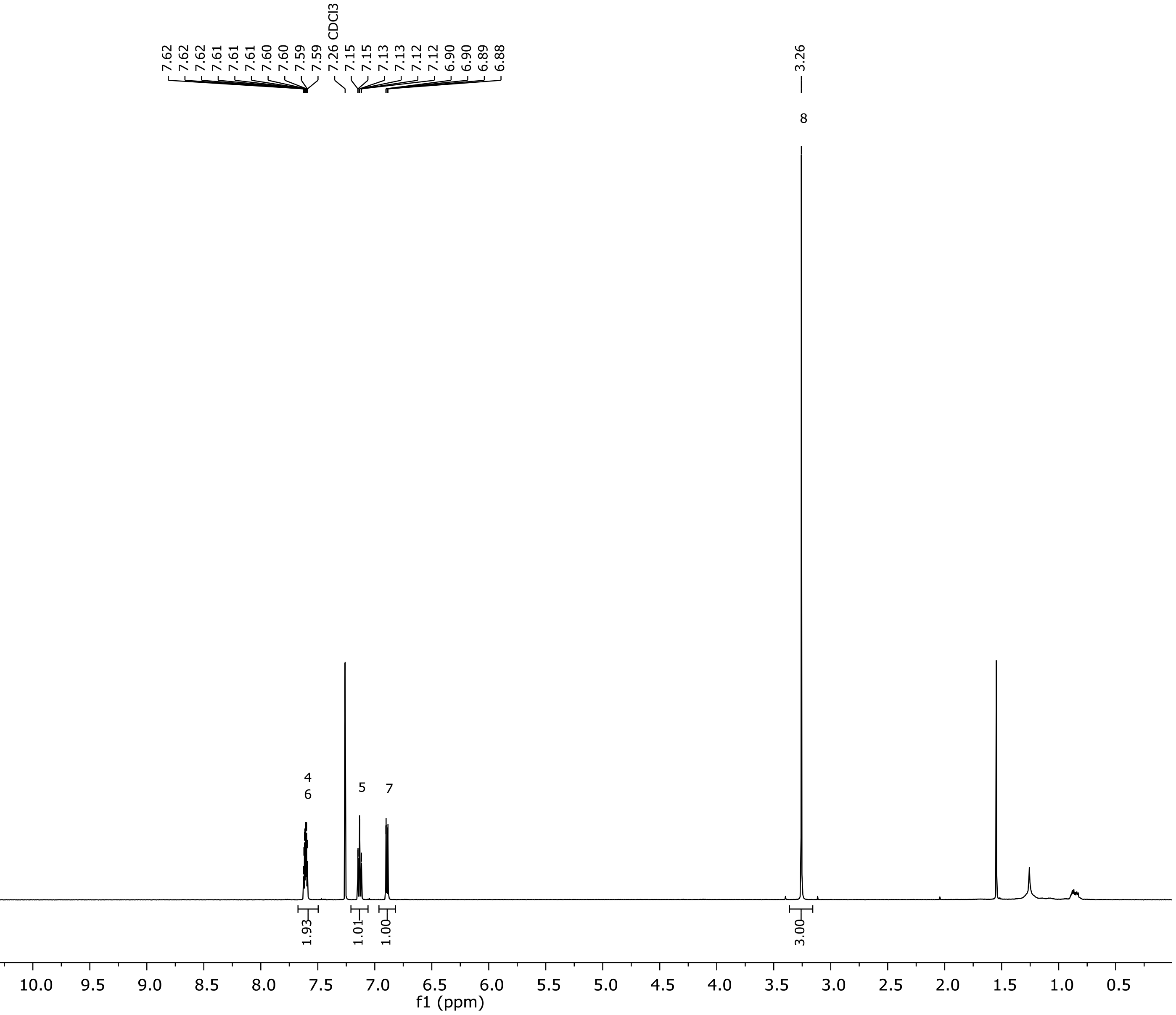

l) ***N*-Allylisatin (S19)**

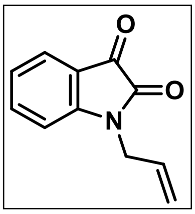

Cesium carbonate (8.96 g, 27.5 mmol) was added to a solution of isatin (3.68 g, 25 mmol) in anhydrous DMF (50 mL) at room temperature under a nitrogen atmosphere, and the reaction stirred for 30 minutes. Allyl bromide (2.60 ml, 30 mmol) was added, and the reaction stirred for a further 21 hours. Ethyl acetate (100 ml) and water (100 ml) were added and the layers separated. The aqueous layer was extracted using ethyl acetate (3 × 50 ml) and the combined organic fractions washed sequentially with water (2 × 100 ml) and brine (2 × 100 ml). The organic layer was dried ($MgSO_4$), filtered and concentrated in vacuo to give a red solid, which was re-dissolved in ethanol (50 mL), and then concentrated again in vacuo. This process was repeated three times (to remove residual DMF) to give the title compound as red solid (3.98 g, 21.3 mmol, 85%). **m.p.** 86-89 °C {Lit.[28] 85-87 °C}; **^{1}H NMR** (400 MHz, $CDCl_3$) δ_H 7.62 (1H, ddd, $^3J_{HH}$ = 7.4 Hz, $^4J_{HH}$ = 1.4 Hz, $^5J_{HH}$ = 0.6 Hz, ArC^4*H*), 7.57 (1H, ddd, $^3J_{HH}$ = 8.0 Hz, 7.6 Hz, $^4J_{HH}$ = 1.4 Hz, ArC^6*H*), 7.12 (1H, ddd, $^3J_{HH}$ = 7.6 Hz, 7.4 Hz, $^4J_{HH}$ = 0.8 Hz, ArC^5*H*), 6.89 (1H, ddd, $^3J_{HH}$ = 8.0 Hz, $^4J_{HH}$ = 0.8 Hz, $^5J_{HH}$ = 0.6 Hz, ArC^7*H*), 5.85 (1H, ddt, $^3J_{HHtrans}$ = 17.2 Hz, $^3J_{HHcis}$ = 10.5 Hz, $^3J_{HH}$ = 5.4 Hz, C*H*=CH_2), 5.33 (2H, dtd, $^3J_{HHtrans}$ = 17.2 Hz, $^4J_{HH}$ = 1.7 Hz, $^2J_{HH}$ = 0.9 Hz, CH=$CH_{cis}H_{trans}$), 5.30 (1H, dtd, $^3J_{HHcis}$ = 10.5 Hz, $^4J_{HH}$ = 1.5 Hz, $^2J_{HH}$ = 0.9 Hz, CH=$CH_{cis}H_{trans}$), 4.37 (2H, ddd, $^4J_{HH}$ = 5.3 Hz, $^4J_{HH}$ = 1.7 Hz, 1.54 Hz, NCH_2).

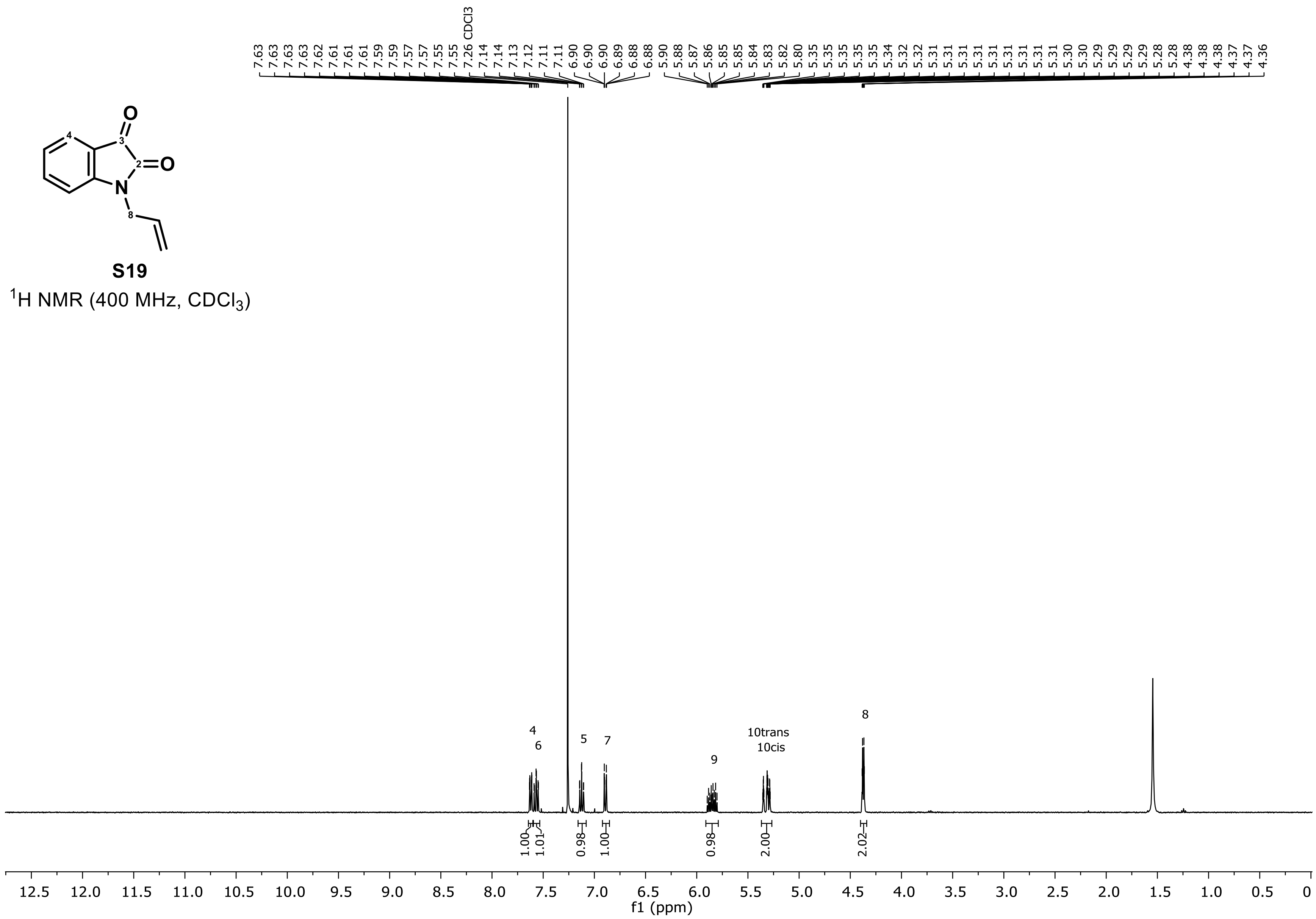
S19
^{1}H NMR (400 MHz, CDCl$_3$)
7.63 7.63 7.63 7.63 7.62 7.61 7.61 7.61 7.59 7.59 7.57 7.57 7.55 7.55 7.26 CDCl3 7.14 7.14 7.13 7.12 7.11 7.11 6.90 6.90 6.90 6.89 6.88 6.88 5.90 5.88 5.87 5.86 5.85 5.85 5.84 5.83 5.82 5.80 5.35 5.35 5.35 5.35 5.35 5.34 5.32 5.32 5.31 5.31 5.31 5.31 5.31 5.31 5.31 5.31 5.30 5.30 5.29 5.29 5.29 5.29 5.29 5.28 5.28 4.38 4.38 4.38 4.37 4.37 4.36
4
6
5
7
9
10trans
10cis
8
1.00
1.01
0.98
1.00
0.98
2.00
2.02
12.5 12.0 11.5 11.0 10.5 10.0 9.5 9.0 8.5 8.0 7.5 7.0 6.5 6.0 5.5 5.0 4.5 4.0 3.5 3.0 2.5 2.0 1.5 1.0 0.5 0
f1 (ppm)

m) ***N-tert*-Butoxycarbonylisatin (S20)**

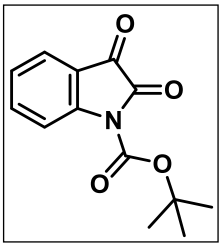

Isatin (1.47 g, 10 mmol) was added to a solution of DMAP (611 mg, 0.50 mmol) in anhydrous THF (50 mL). Di-*tert*-butyl-pyrocarbonate (2.40 g, 11.0 mmol) was added portionwise and the solution stirred under N_2 at r.t. for 6 hours. The reaction was quenched with water (25 mL) and concentrated *in vacuo*. The resulting solid filtered off, dissolved in CH_2Cl_2 and washed with brine. The solution was concentrated and the resulting crude solid recrystallised from hot CH_2Cl_2 and hexane to yield the product (1.60 g, 65%) as an orange solid with data in accordance with the literature.[18] **m.p** 109–110 °C {Lit.[18] 125–126 °C (CH_2Cl_2/Hexane)}, {Lit.[19] 130 °C}, {Lit.[34] 132 °C (THF/Petrol)}; **^{1}H NMR** (500 MHz, $CDCl_3$) δ_H 8.08 (1H, ddd, $^3J_{HH}$ = 8.3 Hz, $^4J_{HH}$ = 0.9 Hz, $^5J_{HH}$ = 0.6 Hz, ArC^4*H*), 7.74 (1H, ddd, $^3J_{HH}$ = 7.6 Hz, $^4J_{HH}$ = 1.5 Hz, $^5J_{HH}$ = 0.6 Hz, ArC^7*H*), 7.70 (1H, ddd, $^3J_{HH}$ = 8.2 Hz, 7.5 Hz, $^4J_{HH}$ = 1.5 Hz, ArC^5*H*), 7.28 (1H, ddd, $^3J_{HH}$ = 7.6 Hz, 7.5 Hz, $^4J_{HH}$ = 0.9 Hz, ArC^6*H*), 1.65 (9H, s, C(CH_3)$_3$).

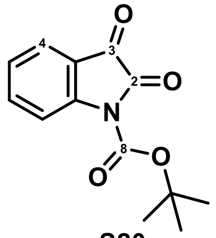

S20

^{1}H NMR (500 MHz, $CDCl_3$)

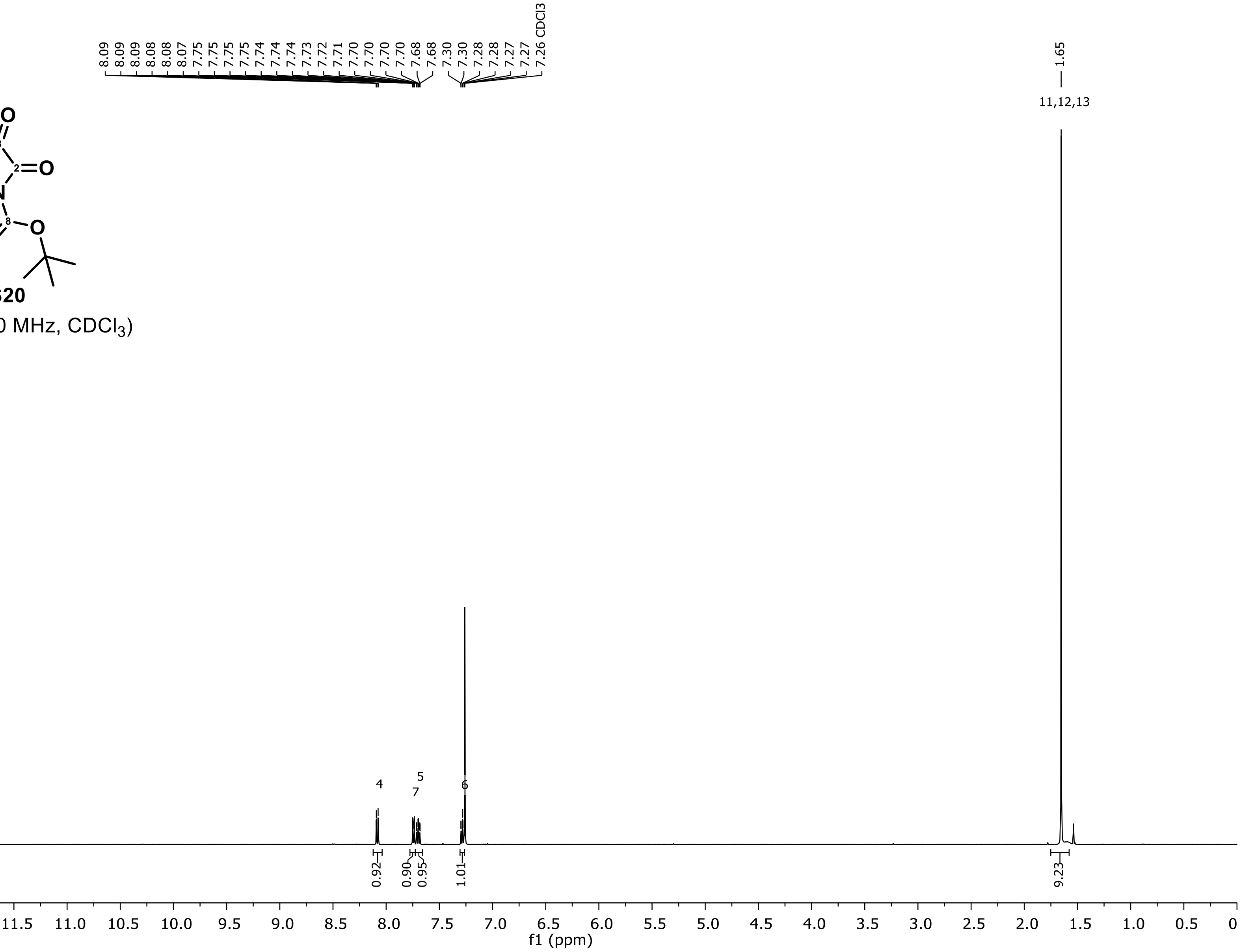

VI. Unsuccessful examples

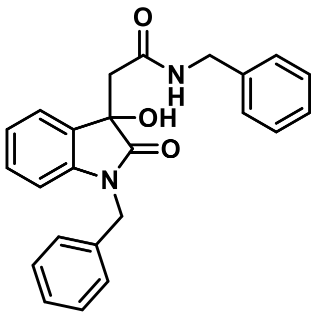

complex mixture
no product observed

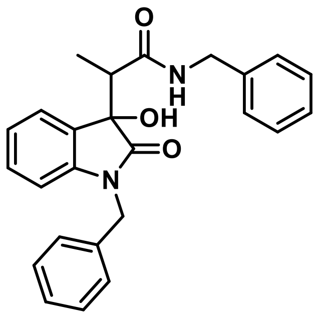

complex mixture
no product observed

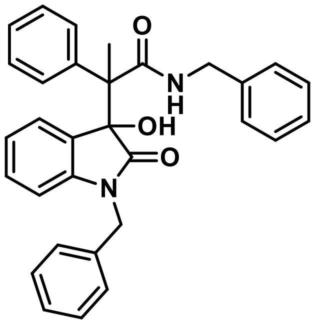

no reaction

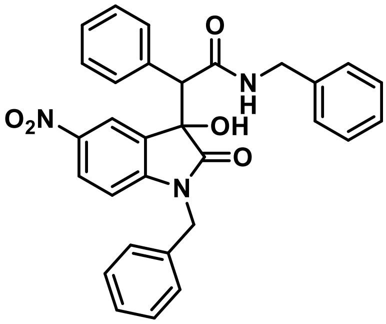

inseparable mixture

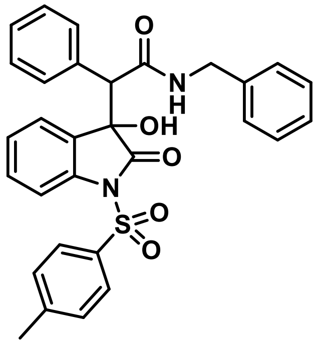

complex mixture
16% of ring-opened

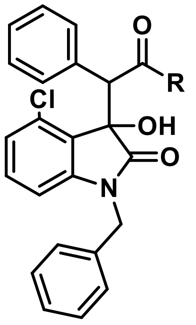

R = NHBn, morpholin-4-yl
inseparable mixture
<10% by NMR

Procedure for the attempted [2+2] with consecutive ring-opening are identical to those reported for compounds **8** – **31**.

VII. References

[1] a) A. Krasovskiy, P. Knochel, *Synthesis* **2006**, *2006*, 890-891; b) A. F. Burchat, J. M. Chong, N. Nielsen, *J. Organomet. Chem.* **1997**, *542*, 281-283.

[2] *Pure Appl. Chem.* **1974**, *37*, 445-462.

[3] W. C. Still, M. Kahn, A. Mitra, *J. Org. Chem.* **1978**, *43*, 2923-2925.

[4] a) G. R. Fulmer, A. J. M. Miller, N. H. Sherden, H. E. Gottlieb, A. Nudelman, B. M. Stoltz, J. E. Bercaw, K. I. Goldberg, *Organometallics* **2010**, *29*, 2176-2179; b) H. E. Gottlieb, V. Kotlyar, A. Nudelman, *J. Org. Chem.* **1997**, *62*, 7512-7515; c) R. K. Harris, E. D. Becker, S. M. Cabral de Menezes, P. Granger, R. E. Hoffman, K. W. Zilm, *Pure Appl. Chem.* **2008**, *80*, 59-84; d) R. K. Harris, E. D. Becker, S. M. Cabral de Menezes, R. Goodfellow, P. Granger, *Pure Appl. Chem.* **2001**, *73*, 1795-1818.

[5] a) *Pure Appl. Chem.* **1976**, *45*, 217-220; b) *Pure Appl. Chem.* **1972**, *29*, 625-628; c) R. K. Harris, J. Kowalewski, S. M. Cabral de Menezes, *Pure Appl. Chem.* **1997**, *69*, 2489-2496.

[6] a) T. R. Hoye, H. Zhao, *J. Org. Chem.* **2002**, *67*, 4014-4016; b) T. R. Hoye, P. R. Hanson, J. R. Vyvyan, *J. Org. Chem.* **1994**, *59*, 4096-4103.

[7] a) K. K. Murray, R. K. Boyd, M. N. Eberlin, G. J. Langley, L. Li, Y. Naito, *Pure Appl. Chem.* **2013**, *85*, 1515-1609; b) J. F. J. Todd, *Pure Appl. Chem.* **1991**, *63*, 1541-1566.

[8] a) H. A. Favre, W. H. Powell, *Nomenclature of Organic Chemistry: IUPAC Recommendations and Preferred Names 2013*, Royal Society of Chemistry, Cambridge, UK, **2014**; b) M. A. Brimble, D. S. Black, R. Hartshorn, A. P. Rauter, C.-K. Sha, L. K. Sydnes, *Pure Appl. Chem.* **2013**, *85*, 307-313; c) W. Mormann, K.-H. Hellwich, *Pure Appl. Chem.* **2008**, *80*, 201-232; d) J. Brecher, *Pure Appl. Chem.* **2008**, *80*, 277-410; e) J. Brecher, *Pure Appl. Chem.* **2006**, *78*, 1897-1970; f) G. P. Moss, *Pure Appl. Chem.* **1999**, *71*, 531-558; g) H. A. Favre, K.-H. Hellwich, G. P. Moss, W. H. Powell, J. G. Trynham, *Pure Appl. Chem.* **1999**, *71*, 1327-1330; h) G. P. Moss, *Pure Appl. Chem.* **1998**, *70*, 143-216; i) G. P. Moss, *Pure Appl. Chem.* **1996**, *68*, 2193-2222; j) W. H. Powell, *Pure Appl. Chem.* **1993**, *65*, 1357-1455; k) W. Drenth, *Pure Appl. Chem.* **1992**, *64*, 989-990; l) G. P. Moss, *Pure Appl. Chem.* **1989**, *61*, 1783-1822; m) J. S. Littler, *Pure Appl. Chem.* **1989**, *61*, 57-81; n) R. A. Y. Jones, J. F. Bunnett, *Pure Appl. Chem.* **1989**, *61*, 725-768; o) R. D. Guthrie, *Pure Appl. Chem.* **1989**, *61*, 23-56; p) W. H. Powell, *Pure Appl. Chem.* **1988**, *60*, 1395-1401; q) J. F. Bunnett, R. A. Y. Jones, *Pure Appl. Chem.* **1988**, *60*, 1115-1116; r) G. P. Moss, *Pure Appl. Chem.* **1987**, *59*, 779-832; s) N. Lozac'h, *Pure Appl. Chem.* **1986**, *58*, 1693-1696; t) W. H. Powell, *Pure Appl. Chem.* **1984**, *56*, 769-778; u) *Pure Appl. Chem.* **1984**, *56*, 595-624; v) W. H. Powell, *Pure Appl. Chem.* **1983**, *55*, 409-416; w) J. E. Merritt, K. L. Loening, *Pure Appl. Chem.* **1983**, *51*, 2251-2304; x) N. Lozac'h, *Pure Appl. Chem.* **1983**, *55*, 1463-1466; y) W. H. Powell, *Pure Appl. Chem.* **1982**, *54*, 217-228; z) *Pure Appl. Chem.* **1982**, *54*, 211-216; aa) *Pure Appl. Chem.* **1982**, *54*, 207-210; ab) J. F. Bunnett, *Pure Appl. Chem.* **1981**, *53*, 305-322; ac) *Pure Appl. Chem.* **1981**, *53*, 1901-1905; ad) *Pure Appl. Chem.* **1979**, *51*, 1995-2003; ae) *Pure Appl. Chem.* **1979**, *51*, 353-380; af) L. C. Cross, W. Klyne, *Pure Appl. Chem.* **1976**, *45*, 11-30; ag) *Pure Appl. Chem.* **1974**, *40*, 315-332; ah) *Pure Appl. Chem.* **1972**, *31*, 283-322; ai) *Pure Appl. Chem.* **1972**, *31*, 639-646; aj) *Pure Appl. Chem.* **1971**, *28*, 1-110; ak) *Pure Appl. Chem.* **1965**, *11*, 1-260.

[9] X.-H. Yang, K. Li, R.-J. Song, J.-H. Li, *Eur. J. Org. Chem.* **2014**, *2014*, 616-623.

[10] *CrystalClear-SM Expert*, v2.0; Rigaku Americas and Rigaku Corporation: The Woodlands, Texas, USA and Tokyo, Japan, **2011**.

[11] *CrysAlisPro*, v1.171.38.46; Rigaku Oxford Diffraction and Rigaku Corporation: Oxford, UK, **2015**.

[12] L. Palatinus, G. Chapuis, *J. Appl. Crystallogr.* **2007**, *40*, 786-790.

[13] G. Sheldrick, *Acta Crystallogr. Sect. C: Cryst. Struct. Commun.* **2015**, *71*, 3-8.

[14] O. V. Dolomanov, L. J. Bourhis, R. J. Gildea, J. A. K. Howard, H. Puschmann, *J. Appl. Crystallogr.* **2009**, *42*, 339-341.

[15] a) L. C. Morrill, L. A. Ledingham, J.-P. Couturier, J. Bickel, A. D. Harper, C. Fallan, A. D. Smith, *Organic & Biomolecular Chemistry* **2014**, *12*, 624-636; b) D. G. Stark, L. C. Morrill, D. B. Cordes, A. M. Z. Slawin, T. J. C. O'Riordan, A. D. Smith, *Chem. Asian J.* **2016**, *11*, 395-400.

[16] a) C. Rüchardt, O. Krätz, S. Eichler, *Chem. Ber.* **1969**, *102*, 3922-3946; b) A. A. M. Roof, H. F. van Woerden, H. Cerfontain, *Tetrahedron* **1976**, *32*, 2967-2971; c) S. R. Smith, C. Fallan, J. E. Taylor, R. McLennan, D. S. B. Daniels, L. C. Morrill, A. M. Z. Slawin, A. D. Smith, *Chem. Eur. J.* **2015**, *21*, 10530-10536.

[17] a) I. Tömösközi, T. Mohácsi, T. Zs, *Tetrahedron* **1971**, *27*, 4931-4938; b) Z. Yang, S. Chen, F. Yang, C. Zhang, Y. Dou, Q. Zhou, Y. Yan, L. Tang, *Eur. J. Org. Chem.* **2019**, *2019*, 5998-6002.

[18] M. D. Greenhalgh, S. M. Smith, D. M. Walden, J. E. Taylor, Z. Brice, E. R. T. Robinson, C. Fallan, D. B. Cordes, A. M. Z. Slawin, H. C. Richardson, M. A. Grove, P. H.-Y. Cheong, A. D. Smith, *Angew. Chem. Int. Ed.* **2018**, *57*, 3200-3206.

[19] C. M. Young, A. Elmi, D. J. Pascoe, R. K. Morris, C. McLaughlin, A. M. Woods, A. B. Frost, A. de la Houpliere, K. B. Ling, T. K. Smith, A. M. Z. Slawin, P. H. Willoughby, S. L. Cockroft, A. D. Smith, *Angew. Chem. Int. Ed.* **2020**, *59*, 3705-3710.

[20] W. M. Eldehna, M. A. El Hassab, M. F. Abo-Ashour, T. Al-Warhi, M. M. Elaasser, N. A. Safwat, H. Suliman, M. F. Ahmed, S. T. Al-Rashood, H. A. Abdel-Aziz, R. El-Haggar, *Bioorg. Chem.* **2021**, *110*, 104748.

[21] M. Farooq, Z. M. Al Marhoon, N. A. Taha, A. A. Baabbad, M. A. Al-Wadaan, A. El-Faham, *Biol. Pharm. Bull.* **2018**, *41*, 350-359.

[22] H. Hellmann, G. Hallmann, F. Lingens, *Chem. Ber.* **1953**, *86*, 1346-1361.

[23] a) C. S. Buxton, D. C. Blakemore, J. F. Bower, *Angew. Chem. Int. Ed.* **2017**, *56*, 13824-13828; b) V. Laina-Martín, J. Humbrías-Martín, J. A. Fernández-Salas, J. Alemán, *Chem. Commun.* **2018**, *54*, 2781-2784; c) F. Shi, Z.-L. Tao, S.-W. Luo, S.-J. Tu, L.-Z. Gong, *Chem. Eur. J.* **2012**, *18*, 6885-6894.

[24] S.-H. Cao, X.-C. Zhang, Y. Wei, M. Shi, *Eur. J. Org. Chem.* **2011**, *2011*, 2668-2672.

[25] D. J. Vyas, R. Fröhlich, M. Oestreich, *J. Org. Chem.* **2010**, *75*, 6720-6723.

[26] J. Itoh, S. B. Han, M. J. Krische, *Angew. Chem. Int. Ed.* **2009**, *48*, 6313-6316.

[27] H.-Q. Liu, D.-C. Wang, F. Wu, W. Tang, P.-K. Ouyang, *Chin. Chem. Lett.* **2013**, *24*, 929-933.

[28] P. Wang, X. Cao, S. Zhang, *Synthesis* **2021**, *53*, 3836-3846.

[29] A. Kamal, R. Mahesh, V. L. Nayak, K. S. Babu, G. B. Kumar, A. B. Shaik, J. S. Kapure, A. Alarifi, *Eur. J. Med. Chem.* **2016**, *108*, 476-485.

[30] K. Dhara, T. Mandal, J. Das, J. Dash, *Angew. Chem. Int. Ed.* **2015**, *54*, 15831-15835.

[31] G. Satish, A. Polu, T. Ramar, A. Ilangovan, *J. Org. Chem.* **2015**, *80*, 5167-5175.

[32] R. P. Singh, U. Majumder, J. n. M. Shreeve, *J. Org. Chem.* **2001**, *66*, 6263-6267.

[33] a) M. Gangar, N. Kashyap, K. Kumar, S. Goyal, V. A. Nair, *Tetrahedron Lett.* **2015**, *56*, 7074-7081; b) C. Fischer, C. Meyers, E. M. Carreira, *Helv. Chim. Acta* **2000**, *83*, 1175-1181.

[34] G. Wille, W. Steglich, *Synthesis* **2001**, *2001*, 759-762.
